# Transcriptional response of *Saccharomyces cerevisiae* to potassium starvation

**DOI:** 10.1186/1471-2164-15-1040

**Published:** 2014-11-29

**Authors:** Ida G Anemaet, G Paul H van Heusden

**Affiliations:** Institute of Biology, Leiden University, Sylviusweg 72, Leiden, 2333BE The Netherlands

**Keywords:** *Saccharomyces cerevisiae*, Cation homeostasis, SAGE tag sequencing, RNAseq, *PHO84*, Antisense RNA, Potassium starvation

## Abstract

**Background:**

Ion homeostasis is essential for every cell and aberrant cation homeostasis is related to diseases like Alzheimer’s disease and epilepsy. The mechanisms responsible for cation homeostasis are only partly understood. The yeast *Saccharomyces cerevisiae* is an excellent organism to study fundamental aspects of cation homeostasis. In this study we investigated the transcriptional response of this yeast to potassium starvation by using Serial Analysis of Gene Expression (SAGE)-tag sequencing.

**Results:**

Comparison of transcript levels in cells grown for 60 min in media without potassium with those in cells grown under standard potassium concentrations showed that the mRNA levels of 105 genes were significantly (P < 0.01) up-regulated more than 2.0-fold during potassium starvation and the mRNA levels of 172 genes significantly down-regulated. These genes belong to several functional categories. Genes involved in stress response including *HSP30*, *YRO2* and *TPO2* and phosphate metabolism including *PHO84*, *PHO5* and *SPL2* were highly up-regulated. Analysis of the promoter of *PHO84* encoding a high affinity phosphate transporter, revealed that increased *PHO84* RNA levels are caused by both increased Pho4-dependent transcription and decreased RNA turnover. In the latter process antisense transcription may be involved. Many genes involved in cell cycle control, and to a lesser extent genes involved in amino acid transport, were strongly down-regulated.

**Conclusions:**

Our study showed that yeast cells respond to potassium starvation in a complex way and reveals a direct link between potassium homeostasis and phosphate metabolism.

**Electronic supplementary material:**

The online version of this article (doi:10.1186/1471-2164-15-1040) contains supplementary material, which is available to authorized users.

## Background

Cation homeostasis is an indispensable process for every living cell. The intracellular concentration of H^+^, K^+^ and Na^+^ cations must be tightly regulated because H^+^ and K^+^ are involved in important processes and activities of many cellular systems. On the other hand, extreme Na^+^ concentrations are toxic for cells. Aberrant cellular cation homeostasis is related to human neurological diseases like Alzheimer’s disease [1, 2] and epilepsy [3]. Furthermore, cation homeostasis is critically important for apoptosis [4] and aberrant serum potassium levels are related to diseases like hypo- and hyperkalemia and Crohn's disease. Properties of ion homeostasis in plants determine their ability to grow in environments with very low or high concentrations of salts (for review see: [5]).

The yeast *Saccharomyces cerevisiae* is an excellent model organism to study cation homeostasis (for reviews see: [6–8]). In this yeast intracellular potassium concentration is relatively stable at 200 – 300 mM. On the other hand, the intracellular Na^+^ concentration is an order of magnitude lower, around 20 mM. Maintenance of high intracellular concentrations of potassium is facilitated by the generation of a membrane potential by the proton ATPase Pma1 [9] and two high affinity potassium transporters Trk1 and Trk2 [10, 11]. Sodium ions are mainly extruded by the Ena1-5 ATP-dependent sodium-potassium transporters [12, 13]. Potassium is extruded from the cells by the Nha1 and Tok1 transporters [14, 15]. The activity of these transporters is highly regulated by several protein kinases and protein phosphatases and by protein turnover. Regulation of ion homeostasis at the transcriptional level is less well understood. Genome-wide studies mainly addressed the transcriptional response to high sodium- or potassium levels rather than the response to cation starvation conditions [16–19].

To further understand cation homeostasis in *S. cerevisiae* a number of European research groups including ours collaborate in the Translucent consortium [8]. To allow studies on the response of yeast to different concentrations of potassium, a new YNB medium containing negligible concentrations of alkali metal cations has been developed by the consortium [20]. In a recent study this medium was used to study the short-term response to potassium starvation at the transcriptional level using DNA microarrays [21]. This study showed that the lack of potassium drastically activates sulfur metabolism (mainly methionine and cysteine metabolism), triggers an oxidative stress response and activate the retrograde pathway. The expression of genes encoding ribosomal proteins and proteins involved in the cell cycle was considerably lowered. In this study, we investigated the transcriptional response to potassium starvation using Serial Analysis of Gene Expression (SAGE)-tag sequencing. The expression of genes involved in several cellular processes was shown to be affected during potassium starvation including genes encoding proteins involved in stress response and phosphate metabolism. The effect of potassium starvation on the expression of *PHO84* encoding an high affinity phosphate transporter was shown to be caused by an activation of the *PHO84* promoter through the Pho4 transcription factor as well as at the level of RNA turnover.

## Results

### Transcriptome analysis by SAGE tag sequencing

To further understand the mechanisms of ion homeostasis we analyzed the effects of potassium starvation on the genome-wide transcription in *S. cerevisiae*. In our studies we used the Translucent YNB medium containing very low concentrations of alkali metal cations, allowing controlled addition of potassium. Exponential growing cells in four independent cultures were transferred to medium containing 50 mM KCl or lacking KCl and grown for 60 min. RNA was isolated for transcriptome analysis. Recent technological developments made RNA sequencing the method of choice over analysis using DNA microarrays for such studies. We used SAGE tag sequencing allowing accurate quantification of RNA levels. The SAGE tags were aligned to a virtual tag library corresponding to all *S. cerevisiae* annotated open reading frames (http://www.yeastgenome.org) including 200 bp downstream sequences. In this way, transcriptional information was obtained for 6053 loci [see Additional file 1].

Comparison of transcript levels isolated from cells grown in media without potassium with those isolated from cells grown under standard potassium concentrations showed that the mRNA levels of 105 genes were significantly (P < 0.01) up-regulated more than 2.0-fold during potassium starvation and the mRNA levels of 172 genes significantly down-regulated. The 25 most up-regulated genes are shown in Table 1, the 25 most down-regulated genes in Table 2. Classification according to GO-term biological process using FunSpec [22] of the genes up-regulated at least 2-fold (P < 0.01) showed that the up-regulated genes belong to many different categories and that especially genes involved in phosphate metabolism, cytogamy and stress response are enriched in this set of genes (Table 3). In addition, genes involved carbohydrate metabolism are affected and genes subject to glucose repression like *HXK1* are induced. Classification of the genes down-regulated at least 2-fold (P < 0.01) by potassium starvation shows a very clear enrichment of genes involved in cell cycle-related processes. In addition genes involved in ammonia assimilation and amino acid transport are enriched in this set of genes (Table 4).

**Table 1:** **The 25 most up-regulated genes after 60 min of potassium starvation (P < 0.01)**

Annotation	Description	RNA level 50 mM (reads per million) (± SD; n = 4)	RNA level 0 mM (reads per million) (± SD; n = 4)	Fold-change (0 mM/ 50 mM)	P-value
*SSN8*	Cyclin-like component of the RNA polymerase II holoenzyme	5.2 ± 2.5	71 ± 20	13.7	0.001
*YER138W-A*	Putative protein of unknown function	2.3 ± 2.3	29 ± 13	12.4	0.007
*CIT3*	Dual specificity mitochondrial citrate and methylcitrate synthase	6.6 ± 3.2	75 ± 26	11.4	0.002
*HSP82*	Hsp90 chaperone; redundant in function with Hsc82p	175 ± 52	1845 ± 333	10.6	0.000
*PDC5*	Minor isoform of pyruvate decarboxylase	21 ± 6	219 ± 43	10.5	0.000
*PHO84*	High-affinity inorganic phosphate (Pi) transporter	50 ± 27	516 ± 214	10.3	0.005
*YBR085C-A*	Protein of unknown function	46 ± 19	443 ± 180	9.6	0.005
*YGR161W-C*	Putative protein of unknown function	7.6 ± 3.4	72 ± 26	9.5	0.002
*YIL082W-A*	Retrotransposon TYA Gag and TYB Pol genes	1.2 ± 0.5	9.8 ± 2.7	8.1	0.001
*SPL2*	Protein with similarity to cyclin-dependent kinase inhibitors	31 ± 5	224 ± 70	7.3	0.001
*MCM10*	Essential chromatin-associated protein	2.1 ± 0.6	14 ± 4	6.9	0.001
*HSP104*	Disaggregase	107 ± 50	718 ± 287	6.7	0.006
*IES5*	Protein that associates with the Ino80 chromatin remodeling complex	31 ± 10	186 ± 66	6.1	0.003
*PHM6*	Protein of unknown function	11 ± 6	66 ± 25	5.9	0.005
*YNL146W*	Putative protein of unknown function	3.2 ± 1.6	19 ± 6	5.8	0.003
*HSP42*	Small heat shock protein with chaperone activity	57 ± 25	331 ± 95	5.8	0.001
*ECL1*	Protein of unknown function	99 ± 49	549 ± 169	5.5	0.002
*STI1*	Hsp90 cochaperone	157 ± 51	805 ± 230	5.1	0.002
*ARE2*	Acyl-CoA:sterol acyltransferase	4.7 ± 1.2	24 ± 10	5.1	0.010
*HXK1*	Hexokinase isoenzyme 1	202 ± 70	1027 ± 373	5.1	0.005
*FUS1*	Membrane protein localized to the shmoo tip	22 ± 13	107 ± 32	4.9	0.002
*SSA3*	ATPase involved in protein folding and the response to stress	7.2 ± 1.8	35 ± 15	4.9	0.010
*ESC2*	Sumo-like domain protein	2.4 ± 1.2	11 ± 5	4.8	0.009
*MET28*	bZIP transcriptional activator in the Cbf1p-Met4p-Met28p complex	11 ± 4	51 ± 20	4.8	0.008
*YBL005W-B*	Retrotransposon TYA Gag and TYB Pol genes	1495 ± 714	7106 ± 1942	4.7	0.002

**Table 2:** **The 25 most down-regulated genes after 60 min of potassium starvation (P < 0.01)**

Annotation	Description	RNA level 50 mM (reads per million) (± SD; n = 4)	RNA level 0 mM (reads per million) (± SD; n = 4)	Fold-change (50 mM/0 mM)	P-value
*YIL171W-A*	Dubious open reading frame	0.6 ± 0.2	0.0 ± 0.0	-	0.001
*RER1*	Protein involved in retention of membrane proteins	0.5 ± 0.2	0.0 ± 0.0	-	0.001
*CLB6*	B-type cyclin involved in DNA replication during S phase	23 ± 10	0.3 ± 0.4	71.2	0.004
*TOS6*	Glycosylphosphatidylinositol-dependent cell wall protein	395 ± 42	12 ± 9	32.5	0.000
*POL30*	Proliferating cell nuclear antigen	166 ± 46	5.7 ± 4.0	29.1	0.000
*PCL1*	Cyclin, interacts with cyclin-dependent kinase Pho85p	100 ± 30	4.1 ± 3.4	24.6	0.001
*TOS4*	Putative transcription factor, contains Forkhead Associated domain	54 ± 15	2.5 ± 2.4	21.3	0.001
*KCC4*	Protein kinase of the bud neck involved in the septin checkpoint	42 ± 14	2.1 ± 1.4	20.3	0.001
*YMR230W-A*	Putative protein of unknown function	23 ± 11	1.1 ± 1.0	20.1	0.007
*YOX1*	Homeobox transcriptional repressor	71 ± 21	4.1 ± 3.1	17.2	0.001
*HTA1*	Histone H2A	1248 ± 392	74 ± 31	17.0	0.001
*CDC45*	DNA replication initiation factor	27 ± 9	1.9 ± 1.3	14.1	0.002
*SWE1*	Protein kinase that regulates the G2/M transition	81 ± 35	6.8 ± 4.7	11.9	0.006
*CDC21*	Thymidylate synthase	98 ± 34	8.3 ± 5.9	11.8	0.002
*ALG14*	Component of UDP-GlcNAc transferase	76 ± 26	6.7 ± 4.6	11.4	0.002
*YIG1*	Protein that interacts with glycerol 3-phosphatase	1.5 ± 0.5	0.1 ± 0.3	10.7	0.003
*MCD1*	Essential alpha-kleisin subunit of the cohesin complex	87 ± 30	8.4 ± 5.2	10.4	0.002
*RPL18B*	Ribosomal 60S subunit protein L18B	774 ± 132	75 ± 13	10.4	0.000
*LSM4*	Lsm (Like Sm) protein	0.9 ± 0.2	0.1 ± 0.2	10.2	0.001
*GAP1*	General amino acid permease	146 ± 18	15 ± 6	9.6	0.000
*YER088C-A*	Dubious open reading frame	24 ± 5	2.6 ± 1.8	9.4	0.000
*YLR413W*	Putative protein of unknown function	207 ± 58	22 ± 11	9.4	0.001
*TOS2*	Protein involved in localization of Cdc24p to the site of bud growth	16 ± 1	1.7 ± 1.5	9.2	0.000
*PMI40*	Mannose-6-phosphate isomerase	361 ± 96	40 ± 29	9.1	0.001
*KIP1*	Kinesin-related motor protein	21 ± 7	2.3 ± 1.6	8.9	0.002

**Table 3:** **Functional analysis using FunSpec** [22] **of genes up-regulated at least 2-fold (P < 0.01) during potassium starvation**

Category	p-value (x10 ^−3^ )	In category from cluster	k ^a^	f ^a^
polyphosphate metabolic process [GO:0006797]	0.19	*VTC2 VTC4 PHO84*	3	8
cytogamy [GO:0000755]	0.19	*FUS1 FIG 2 STE2*	3	8
response to stress [GO:0006950]	0. 52	*SSA3 HSP26 AAD4 HSP42 MDJ1 POG1 HSP104 STI1 HSP82*	9	152
budding cell bud growth [GO:0007117]	2.46	*ROM1 NAP1 REH1*	3	18
carbohydrate metabolic process [GO:0005975]	3.18	*GLC3 HXK1 PGM3 GLC8 ZWF1 SOL1*	6	94
'de novo' protein folding [GO:0006458]	3.40	*MDJ1 HSP82*	2	6
biological_process unknown [GO:0008150]	3.70	*EDS1 YBR053C YBR085C-A PCS60 RTN2 PHM6 IES5 YER138W-A TMT1 YGR127W ECL1 YGR161W-C HUA1 AIM17 YIL102C-A IKS1 YJL127C-B YKL023W TTI1 YKR075C YLR149C YLR271W CMC4 PGM3 YNL058C YNL146W YNR014W AIM41 YOR289W UIP4*	30	1203
NADPH regeneration [GO:0006740]	4.72	*IDP3 ZWF1*	2	7
cell adhesion [GO:0007155]	4.72	*FIG 2 SAG1*	2	7
glucose metabolic process [GO:0006006]	5.06	*HXK1 PGM3 ZWF1*	3	23
branched chain family amino acid catabolic process [GO:0009083]	7.93	*BAT2 PDC5*	2	9
re-entry into mitotic cell cycle after pheromone arrest [GO:0000321]	7.93	*FAR7 POG1*	2	9
vacuole fusion, non-autophagic [GO:0042144]	9.77	*SWF1 VTC2 VTC4*	3	29
deadenylation-dependent decapping of nuclear-transcribed mRNA [GO:0000290]	9.81	*EDC2 DCS2*	2	10
microautophagy [GO:0016237]	9.81	*VTC2 VTC4*	2	10

a, k: number of genes from the input cluster in given category; f: number of genes total in given category.

**Table 4:** **Functional analysis using FunSpec** [22] **of genes down-regulated at least 2-fold (P < 0.01) during potassium starvation**

Category	p-value (x10 ^−3^ )	In category from cluster	k ^a^	f ^a^
cell division [GO:0051301]	0.007	*LTE1 KIP1 HSL7 KCC4 MCD1 UBC9 PDS1 SPC25 CLB1 CLB6 SWE1 PCL1 SGO1 NUD1 CLN2 CLB5 KAR3*	17	190
regulation of cyclin-dependent protein kinase activity [GO:0000079]	0.008	*CLB1 CLB6 SWE1 PCL1 CLN2 CLB5*	6	20
cell cycle [GO:0007049]	0.019	*LTE1 KIP1 HSL7 KCC4 MCD1 PSA1 UBC9 PDS1 SPC25 CLB1 CLB6 SWE1 SLD2 CDC45 YOX1 TOF1 PCL1 SGO1 NUD1 CLN2 CLB5 KAR3*	22	316
mitosis [GO:0007067]	0.15	*LTE1 KIP1 HSL7 MCD1 UBC9 PDS1 SPC25 CLB1 SWE1 SGO1 NUD1 KAR3*	12	132
ammonia assimilation cycle [GO:0019676]	0.16	*GAP1 PUT4 GDH1*	3	5
amino acid transmembrane transport [GO:0003333]	0.31	*GNP1 HNM1 GAP1 BIO5 PUT4*	5	24
double-strand break repair via break-induced replication [GO:0000727]	0.46	*RDH54 SLD5 SLD2 CDC45 CTF4*	5	26
DNA replication [GO:0006260]	0.66	*POL12 POL30 SLD5 SLD2 CDC45 TOF1 RFA2 CTF4*	8	75
G2/M transition of mitotic cell cycle [GO:0000086]	0.67	*HSL7 UBC9 CLB1 SWE1 CLB5*	5	28
positive regulation of DNA replication [GO:0045740]	0.67	*CLB6 CLB5*	2	2
GDP-mannose biosynthetic process [GO:0009298]	0.67	*PSA1 PMI40*	2	2
meiotic mismatch repair [GO:0000710]	0.87	*POL30 MSH6 PMS1*	3	8
budding cell bud growth [GO:0007117]	1.00	*KCC4 GIN4 TOS2 DFG5*	4	18
'de novo' IMP biosynthetic process [GO:0006189]	1.28	*ADE17 ADE4 ADE2*	3	9
microtubule nucleation [GO:0007020]	1.84	*SPC25 SPC97 STU2 SPC98*	4	21
gamma-aminobutyric acid transport [GO:0015812]	1.97	*GAP1 PUT4*	2	3
regulation of S phase of mitotic cell cycle [GO:0007090]	1.97	*CLB6 CLB5*	2	3
mitotic sister chromatid cohesion [GO:0007064]	3.08	*POL30 TOF1 CTF4 KAR3*	4	24
cell wall mannoprotein biosynthetic process [GO:0000032]	3.16	*PSA1 PMI40 KTR1*	3	12
glycine metabolic process [GO:0006544]	3.87	*SHM2 GCV2*	2	4
septin checkpoint [GO:0000135]	3.87	*KCC4 GIN4*	2	4
amino acid transport [GO:0006865]	4.30	*GNP1 HNM1 GAP1 BIO5 PUT4*	5	42
response to DNA damage stimulus [GO:0006974]	4.92	*RDH54 POL30 MCD1 DUN1 MSH6 HTA1 SRS2 TOS4 OGG1 PMS1 TOF1 SMC5*	12	197
telomere maintenance via telomerase [GO:0007004]	6.17	*EST1 RAP1 RFA2*	3	15
DNA repair [GO:0006281]	7.71	*RDH54 POL30 MSH6 HTA1 SRS2 OGG1 PMS1 TOF1 RFA2 SMC5 CTF4*	11	183
negative regulation of transcription from RNA polymerase II promoter, global [GO:0045816]	9.34	*HTB1 HTA1*	2	6
regulation of mitotic cell cycle [GO:0007346]	9.34	*SMI1 YOX1*	2	6
premeiotic DNA replication [GO:0006279]	9.34	*CLB6 CLB5*	2	6
replication fork protection [GO:0048478]	9.34	*DUN1 TOF1*	2	6

a, k: number of genes from the input cluster in given category; f: number of genes total in given category.

Our SAGE-tag sequencing showed that the stress response genes *HSP30*, *TPO2* and *YRO2* were highly up-regulated upon potassium starvation, but due to the variation in expression in the individual cultures the effect did not meet our criteria for significance, i.e. P < 0.01. By real time qRT-PCR we verified the effects of potassium starvation on these genes, using *ACT1* as control. As shown in Table 5 qRT-PCR clearly showed that *HSP30*, *TPO2* and *YRO2* were strongly up-regulated upon potassium starvation in agreement with the RNAseq data. As an additional control we verified the expression of *GAP1* and *ALG9* by qRT-PCR. Both by RNAseq and qRT-PCR we showed that *GAP1* was strongly down-regulated whereas the control gene *ALG9*[23] was only slightly affected by potassium starvation (Table 5).

**Table 5:** **Analysis of the effect of potassium starvation by qRT-PCR**

Gene	Fold change qRT-PCR		Fold change RNAseq
	Experiment 1	Experiment 2	
*TPO2*	4.5	5.1	4.6 (P = 0.025)
*HSP30*	11.1	18.0	19.7 (P = 0.011)
*YRO2*	10.4	40.9	23.1 (P = 0.075)
*GAP1*	- 12.9^a^	- 23.7	−9.6 (P < 0.001)
*ALG9*	1.2	1.7	−1.25

a, down-regulation is indicated with ‘-‘.

### Growth of selected deletion strains at low potassium concentrations

**Figure 1:**
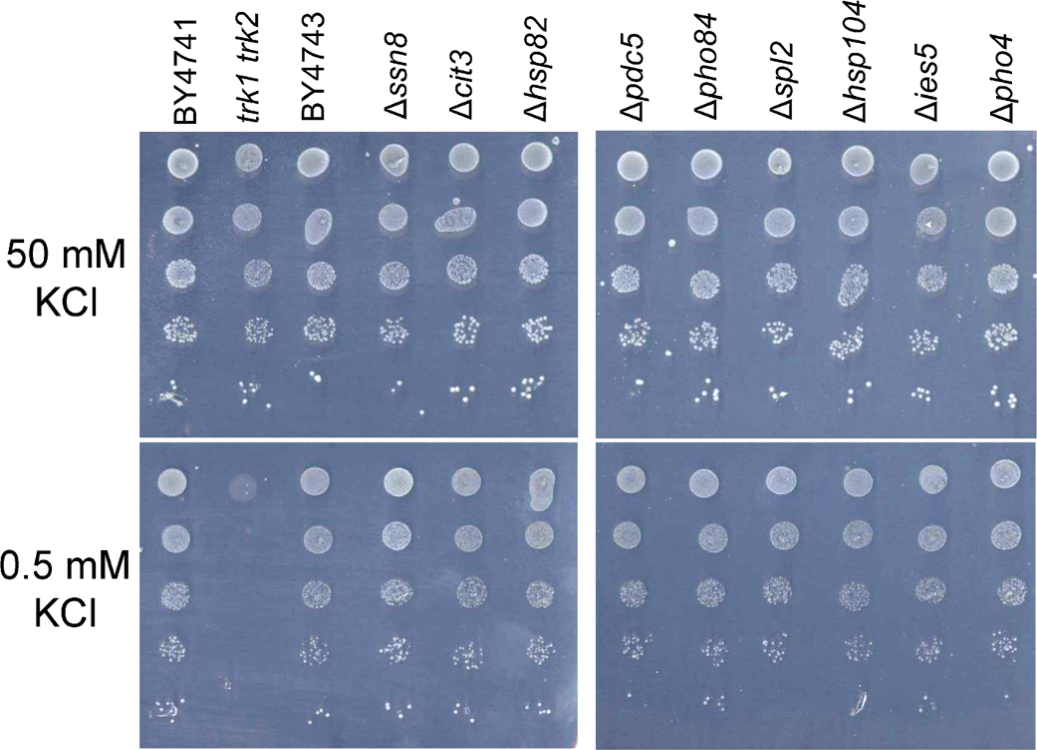
**Growth at 50 and 0.5 mM KCl of deletion strains lacking genes highly up-regulated during potassium starvation or lacking** ***PHO4*** **.** Ten-fold serial dilutions of the indicated strains are spotted on Translucent YNB plates supplemented with histidine, methionine, leucine and uracil containing 50 or 0.5 mM KCl. Plates were incubated for 3 days at 30°C.

An obvious question is whether the up-regulated genes are required for growth at low potassium concentrations. To this end strains deleted for the most up-regulated genes or deleted for *PHO4* (encoding a transcription factor regulating the expression of genes involved in phosphate metabolism, see below) were spotted on plates with 50 or 0.5 mM KCl. The *trk1trk2* double disruptant, lacking the high affinity potassium transporters Trk1 and Trk2, was used as a control. None of the selected disruptants (Δ*ssn8*, Δ*cit3*, Δ*hsp82*, Δ*pdc5*, Δ*pho84*, Δ*spl2*, Δ*hsp104*, Δ*ies5*, Δ*pho4*) showed an aberrant growth at 0.5 mM KCl, whereas the *trk1trk2* mutant (BYT12) did not grow at this KCl concentration (Figure 1). These results indicate that the most up-regulated genes are not by themselves required for growth at low potassium concentrations. To study the survival of the tested disruption strains at low potassium conditions, these strains were spotted on a plate lacking potassium and after six days the cells were transferred to plates with 50 mM KCl. No significant differences in growth compared to the wild type strain were observed, except for the *trk1 trk2* strain (data not shown).

### Promoter analysis

Using the information available in the Yeastract database (http://www.yeastract.com) it became clear that many different transcription factors are associated with the up-regulated genes. This suggests that the transcriptional response to potassium starvation is not linked to the activation of a single transcription factor. Two of the up-regulated genes related to stress-response, i.e. *YRO2* and *TPO2*, are also up-regulated upon weak acid stress [24]. The latter up-regulation is dependent on the transcription factor Haa1. However, in a *Δhaa1* mutant *YRO2* is still highly up-regulated upon potassium starvation (RM Läkamp and GPH van Heusden, unpublished results) making a role of Haa1 in the response to potassium starvation less likely. To further analyze the regulation of *YRO2* and *TPO2* the promoter sequences were placed upstream of the bacterial ß-galactosidase gene in plasmid pRUL302 and introduced in yeast strain BY4741. The resulting strains were cultivated in the presence of 50 mM KCl till mid-exponential growth, transferred to media with 50 mM KCl or to media lacking KCl and grown for 60 min. Then, cells were isolated and ß-galactosidase activity was determined. Using 510 bp of sequences of the *TPO2* promoter or 2141 bp of sequences of the *YRO2* promoter we found an only 1.3-fold or 2.1-fold, respectively, increase in β-galactosidase activity upon potassium starvation, whereas RNAseq showed 4.6-fold (P = 0.025) and 23-fold (P = 0.075), respectively, increase in mRNA level, suggesting that also other mechanisms than activation of the promoter are involved in the expression of *YRO2* and *TPO2* during potassium starvation.

### Regulation of PHO84 by the transcription factor Pho4

Our RNAseq data reveal that genes involved in phosphate metabolism are well represented in the set of genes that were significantly up-regulated. Many of these genes, including *PHO84*, are strongly regulated in response to external phosphate concentrations (for review see: [25]). The transcription factor Pho4 translocates into the nucleus at low phosphate concentrations and plays a prominent role in activation of *PHO84* expression [26]. To investigate the involvement of Pho4 in the activation of the *PHO84* promoter during potassium starvation we inserted 600 bp promoter sequences upstream of the ß-galactosidase gene in plasmid pRUL302 and introduced the resulting plasmid in BY4741 and the Δ*pho4* disruptant in the BY4741 background (each containing the pRS313 plasmid). The resulting strains were cultivated as described above to determine the effect of potassium starvation on the *PHO84* promoter. As shown in Table 6, in the wild type background (BY4741) there is a clear activation of the *PHO84* promoter, whereas in the Δ*pho4* strain hardly any activity can be detected, both after cultivation at 50 mM and 0 mM KCl. Introduction of an intact copy of *PHO4* (pRS313[PHO4]) in the wild type background gives a stimulation of the *PHO84* promoter, both at 50 and 0 mM KCl. Introduction of an intact copy of *PHO4* (pRS313[PHO4]) in the Δ*pho4* strain restores and activates the *PHO84* promoter. These results indicate that the Pho4 transcription factor is required for the expression of *PHO84* at low potassium concentrations.

**Table 6:** **Role of Pho4 in the transcriptional regulation of** ***PHO84*** **during potassium starvation**

Strain	Plasmid	ß-galactosidase activity (arbitrary units)		Fold change (0 mM/50 mM)
		50 mM KCl	0 mM KCl	
BY4741	pRS313	0.13	0.56	4.1
BY4741	pRS313[PHO4]	2.36	3.34	1.4
Δpho4	pRS313	0.00	0.00	-
Δpho4	pRS313[PHO4]	1.41	2.84	2.0

Results of a typical experiment are given. In each experiment two independent transformants were analyzed, the average is shown.

### Promoter replacement

To further investigate the regulation of gene expression during potassium starvation we replaced the promoter sequences of a number of genes (*HSP30*, *TPO2*, *YRO2*, *PHO84* and *ALG9*) by the *CYC1* promoter. This promoter is most likely not influenced by potassium starvation as the levels of *CYC1* RNA were hardly changed (1.2-fold lower levels after potassium starvation). The resulting strains were cultivated as above and RNA was isolated. Gene expression was determined by qRT-PCR using *ACT1* as a reference gene. In this way we found the following effects of potassium starvation on the RNA levels: *HSP30*, 0.9- and 1.3-fold increase in two independent experiments; *TPO2*, 0.8- and 2.2-fold increase; *YRO2*, 5.3- and 3.5-fold decrease; *PHO84*, 6.8- and 8.6--fold increase; *ALG9* (reference gene), 1.0- and 1.2-fold increase. These observations show that especially for *PHO84* other regulatory aspects are involved besides activation of the promoter. Surprisingly, the levels of *YRO2* RNA are reduced at 0 mM KCl.

### Antisense transcription

**Figure 2:**
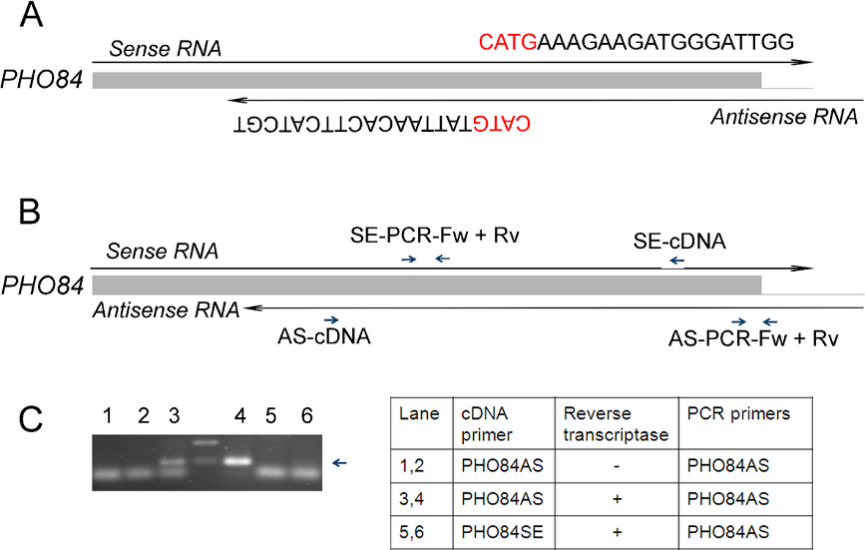
**Antisense transcription of** ***PHO84*** **. A**, scheme showing the position of SAGE tags corresponding to sense and antisense transcripts. **B**, primers used to detect *PHO84* antisense transcripts. **C**. Detection of antisense transcripts using strand specific cDNA synthesis and PCR. RNA was isolated from cells grown for 60 min in Translucent YNB medium lacking potassium. Arrow indicates the position of the expected PCR fragment.

Several studies have shown that *PHO84* is also transcribed in the opposite direction yielding antisense RNA [27, 28]. SAGE tag sequencing is able to identify antisense transcripts [29, 30]. This is illustrated for *PHO84* in Figure 2A. Alignment of the sequenced tags to the complementary strand of the *S. cerevisiae* open reading frames (+ 200 bp downstream sequences) reveals potential antisense transcripts corresponding to 5665 loci [see Additional file 2]. The total number of tags aligning to the complementary strand is 4 to 14 percent of the total number of tags aligning to the coding strand. Comparison of the antisense transcript levels in cells grown in medium without potassium with the levels in cells grown under standard potassium concentrations shows that antisense transcription of 34 genes was significantly (P < 0.01) up-regulated more than 2-fold, whereas antisense transcripts of 76 genes were significantly down-regulated [see Additional file 3]. Several tags aligned to the complementary strand of *PHO84*. Almost all tags were found at a higher level using RNA isolated from cells grown at 0 mM KCl compared to cells grown at 50 mM KCl [see Additional file 3]. The total number of tags corresponding to antisense transcripts of the *PHO84* gene is 8.7-fold (P = 0.07) increased after potassium starvation.

Antisense *PHO84* transcripts were further analyzed by RT-PCR using strand specific primers. cDNA synthesis using a primer specific for antisense RNA (primer AS-cDNA, Figure 2B) yields cDNA dependent on antisense transcripts. Using primers AS-PCR-Fw and AS-PCR-Rv (Figure 2B) this cDNA can be detected by PCR. As shown in Figure 2C (lanes 3 and 4) the expected PCR fragment was found. This band was not found when primer SE-cDNA (Figure 2B, lane 5 and 6) was used as primer for cDNA synthesis, which generates cDNA corresponding to sense transcripts. These results indicate that antisense *PHO84* RNA can be detected RT-PCR, in agreement with our RNAseq experiments and previous studies.

## Discussion and conclusion

The yeast *S. cerevisiae* is an excellent organism to study fundamental aspects of cation homeostasis. Although many components involved in the maintenance of cation homeostasis in this yeast have already been revealed, still many aspects remain unclear. Understanding the response of yeast cells to potassium starvation is essential for understanding the mechanisms involved in cation homeostasis. Therefore, the Translucent consortium developed a new medium, based on the widely used YNB-medium, containing negligible amounts of alkali metal cations [20]. This medium allowed potassium starvation studies. Studies have been conducted to address effects at the transcriptional [21] and proteomic level [31]. Using DNA microarrays it was shown that the lack of potassium affects the expression of more than one thousand genes including a strong activation of genes involved in sulfur metabolism, oxidative stress response and the retrograde pathway and a reduced expression of genes involved in ribosome biogenesis and cell cycle control. At the protein level a general decrease in protein content and an increased level of stress-related proteins were found.

In this study we used SAGE-tag sequencing to analyze the transcriptional response of *S. cerevisiae* to potassium starvation. We used four independent biological replicates and for reasons unknown the variation between these replicates is somewhat larger than expected. Despite this variation, similarly to the study with DNA microarrays we found a very pronounced effect of potassium starvation on gene expression and the expression of genes belonging to many functional categories was affected. The power of SAGE-tag sequencing is that also lowly expressed genes can be analyzed. Therefore, we were able to obtain information on the expression of 6053 different loci, much higher than what can be achieved by DNA microarrays. Both in this study and in the study with DNA microarrays a strong up-regulation of genes involved in stress response and a strong down-regulation of genes involved in various aspects of cell cycle control were found. Although the overall trend found in this and the previous study is very similar, also quantitative differences were found. One of the examples is the effect on the expression of genes involved in sulfur metabolism. In the previous study after 60 min of potassium starvation *SUL2*, *MUP3*, *MET3*, *STR3*, *MET14*, *MET28*, *SER3* and *MET10* were all up-regulated more than 4-fold. For these genes we also found up-regulation, although much less pronounced.

The effect of potassium starvation on the *S. cerevisiae* transcriptome has also been investigated using chemostat cultures [32]. In that study, only a limited number of genes were strongly affected by potassium limitation, and the great majority of these were genes known to be involved in nitrogen metabolism. Genes encoding ammonium ion and amino acid transporters (*MEP2* and *GAP1*) were down-regulated 30-fold and virtually all other nitrogen catabolite-repressed genes were strongly down-regulated as well. SPS-regulated amino acid permeases were strongly up-regulated. In our study we also found a strong down-regulation of *GAP1* and *MEP2* (9.6 and 7.4-fold, respectively). On the other hand, in our study genes encoding amino acid permeases were down-regulated instead of up-regulated (*GNP1* 5.5-fold, *DIP1* 2.6-fold, *TAT2* 1.1-fold, *AGP1* 9.8-fold, *PTR2* 3.0-fold, *BAP2* 3.5-fold and *MUP1* 1.3-fold-down-regulated, see Additional file 1). In this respect it has to be mentioned that the physiological state of yeast cells in chemostat cultures can be greatly different from that of cells in shake flask cultures and that different media were used in the two studies.

**Figure 3:**
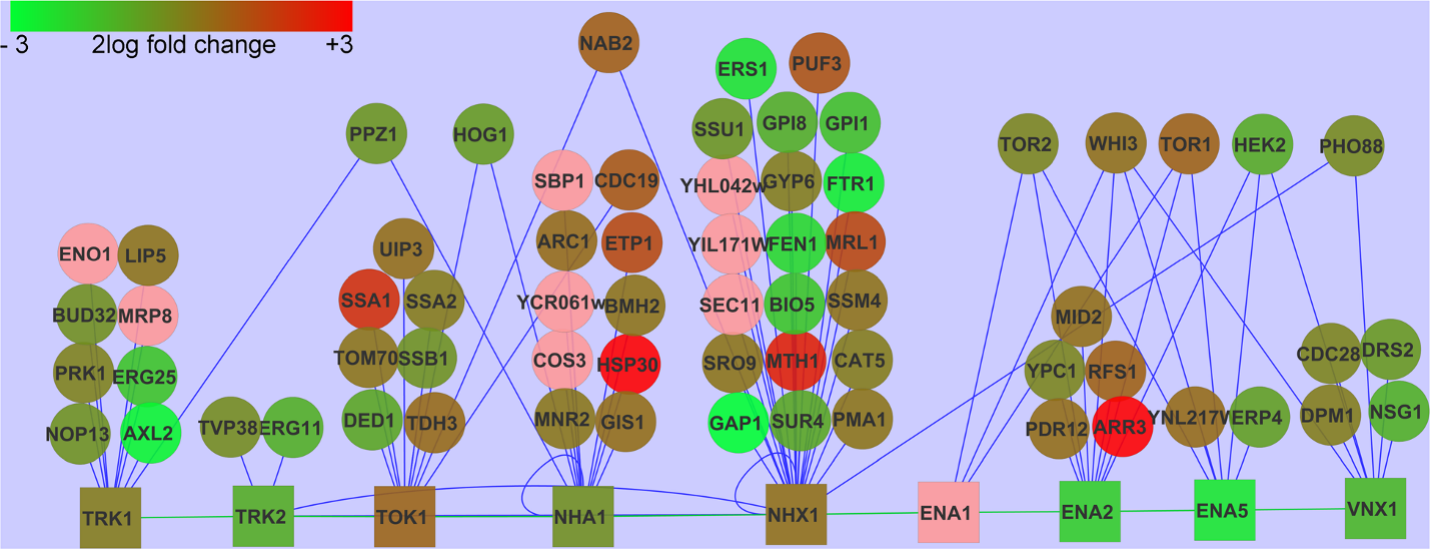
**The effect of potassium starvation on the RNA level of a number of** ***S. cerevisiae*** **ion transporters and their binding partners.** Protein binding partners (physical interactions) were taken from http://www.yeastgenome.org. Squares: ion transporters; circles: binding partners. Fold changes in RNA levels are indicated in a color scale, down-regulation in green and up-regulation in red; pink, RNAseq data not available. Protein-protein interactions are shown with blue lines. The figure was made using Cytoscape [33].

Potassium homeostasis is regulated by a complex interplay between transporters and regulatory proteins. Many proteins have been identified that bind to the various *S. cerevisiae* ion transports [8] (http://www.yeastgenome.org). The function of most of these interactions is still unknown and a role in the regulation of the ion transporters is imaginable. In Figure 3 a number of *S. cerevisiae* ion transporters and their binding partners is shown and the effect of potassium starvation on their transcript levels is indicated in a color scale, with down-regulation in green and up-regulation in red. It is clear that the RNA levels of some binding partners are increased and of some binding partners decreased. For example, *HSP30*, encoding a Nha1 binding partner, and *ARR3*, encoding an Ena2 binding partners are strongly up-regulated. The RNA levels of many, but not all, of the binding partners of the Nhx1 transporter are decreased. However, the significance of these observations still has to be shown.

Identification of transcription factors involved in the regulation of gene expression during potassium starvation is very complex. Using the information on transcription factor associations in the Yeastract database (http://www.yeastract.com) many different transcription factors were found associated with the up- or down-regulated genes. The transcription factor Pho4 may participate in gene regulation during potassium starvation. This transcription factor is found to be associated with seven of the 25 most up-regulated genes, i.e. *ARE2*, *FUS1*, *HSP104*, *PHM6*, *PHO84*, *SPL2* and *YNL146W* (http://www.yeastract.com). Deletion of *PHO4* strongly affected the activity of *PHO84* promoter. However, other transcription factors are most likely be involved as well. In addition, RNA turnover is important. After replacement of the *PHO84* promoter by the *CYC1* promoter *PHO84* RNA levels are still approximately five-fold increased.

In this study as well as in the study using DNA microarrays [21] a strong induction if genes involved in phosphate metabolism, including *PHO84*, was found. Thus, despite the presence of phosphate in the medium, these genes are not repressed, when potassium is absent. The transcription factor Pho4 plays a key role in the regulation of these genes. The subcellular localization of this transcription factor is regulated via phosphorylation by the Pho80-Pho85 cyclin-kinase complex [26]. When phosphate is available Pho4 is retained in the cytoplasm, whereas under phosphate limiting conditions it translocates into the nucleus, resulting in expression of genes involved in phosphate uptake. During potassium starvation we observed an increase in nuclear Pho4-GFP (data not shown). The expression of *PHO4*, *PHO80* and *PHO85* is only slightly and not significantly affected by potassium starvation (1.14-, 0.58- and 0.94-fold, respectively) [see Additional file 1], suggesting that transcriptional regulation of these genes does not play a role. However, the mechanism of the regulation of Pho4 during potassium starvation still has to be revealed.

Several studies have shown that *PHO84* is also transcribed in the opposite direction yielding antisense RNA [27, 28]. This antisense RNA induces transcriptional silencing of *PHO84* via histone deacetylation of the *PHO84* promoter when phosphate is available. Our SAGE tag sequencing revealed many tags corresponding to antisense transcripts corresponding to 5665 loci [see Additional file 2] and the levels of many of these tags were affected by potassium starvation, including those of *PHO84* [see Additional files 2 and 3]. By applying SAGE tag sequencing, tags corresponding to antisense transcripts were also found in other studies [29, 30]. However, it has been suggested that spurious synthesis of second-strand cDNA during reverse transcription reactions triggers antisense artifacts [34]. In our studies we performed cDNA synthesis on-the-bead making the formation of artifacts less likely [30]. Furthermore, several studies showed the widespread occurrence of antisense transcription in *S. cerevisiae* (eg. [35, 36]). By RT-PCR using antisense-specific primers during cDNA synthesis we were able to provide additional evidence for antisense transcription of *PHO84* (Figure 2). However, the role of antisense transcription in the regulation of *PHO84* during potassium starvation still has to be disclosed.

## Methods

### Yeast strains and growth conditions

In this study the yeast strain BY4741 and strains derived from BY4741 were used, as listed in Table 7. For analysis of growth at low potassium concentrations also a number of homozygous diploid deletion strains in the BY4743 background were used, obtained from Euroscarf (Frankfurt, Germany). Yeast was grown in YPD medium or selective MY medium supplemented, if required, with histidine, leucine, methionine and/or uracil to a final concentration of 20 mg/L [37]. For cultivation at defined potassium concentrations YNB medium containing very low concentrations of alkali metal cations, developed by the Translucent consortium, was used [20]. Yeast transformations were performed using the LiAc method [38]. Yeast strains carrying plasmids were obtained by transforming parental strains with the appropriate plasmids followed by selection for histidine, and/or uracil prototrophy.

**Table 7:** **Yeast strains used in this study**

Yeast strain	Genotype	Source/Reference
BY4741	*MATa his3Δ1 leu2Δ0 met15Δ0 ura3Δ0*	Euroscarf, Germany
P_CYC1_:: PHO84 (GG3425)	*MATa his3Δ1 leu2Δ0 met15Δ0 ura3Δ0* P_CYC1_::*PHO84* (KAN.MX)	This study
P_CYC1_:: YRO2 (GG3424)	*MATa his3Δ1 leu2Δ0 met15Δ0 ura3Δ0* P_CYC1_::*YRO2* (KAN.MX)	This study
P_CYC1_:: TPO2 (GG3423)	*MATa his3Δ1 leu2Δ0 met15Δ0 ura3Δ0* P_CYC1_::*TPO2* (KAN.MX)	This study
P_CYC1_:: HSP30 (GG3422)	*MATa his3Δ1 leu2Δ0 met15Δ0 ura3Δ0* P_CYC1_:: *HSP30* (KAN.MX)	This study
P_CYC1_:: ALG9 (GG3426)	*MATa his3Δ1 leu2Δ0 met15Δ0 ura3Δ0* P_CYC1_::A*LG9* (KAN.MX)	This study
BYT12	*MATa his3Δ1 leu2Δ0 met15Δ0 ura3Δ0 trk1*Δ::*loxP trk2*Δ::*loxP*	Hana Sychrova, Prague [39]
Δpho4	*MATa his3Δ1 leu2Δ0 met15Δ0 ura3Δ0 pho4*Δ::KAN.MX	Euroscarf, Germany

### Replacement of promoters by the CYC1 promoter

To replace the endogenous promoters of *YRO2*, *HSP30*, *TPO2*, *PHO84* or *ALG9* by the *CYC1* promoter a DNA fragment was generated by PCR on plasmid pYM-N10 [40] using the primer combinations YRO2-Fw-prom/YRO2-Rev-CYC1, HSP30-Fw-prom/HSP30-Rev-CYC1, TPO2-Fw-prom/TPO2-Rev-CYC1, PHO84-Fw-prom/PHO84-Rev-CYC1 or ALG9-Fw-prom/ALG9-Rev-CYC1, respectively. The primer sequences are shown in Table 8. These DNA fragments were used to transform BY4741 and transformants were selected on YPD plates containing 150 μg/ml G418. Correct integration was verified by PCR.

**Table 8:** **Primers used in this study**

Primer	Sequence (5’ – 3’)
YRO2-Fw-prom	TTTACGAAAAGTGTCTAGTTGCTCAATGCATATAAACTTAATCTA GCTTCGTACGCTGCAGGTCG
YRO2-Rev-CYC1	GGCTTCGTTACCACCTCTTTTCAATAGTTCAACATAATCAGACATTTAGTGTGTGTATTTGTGTTTGC
HSP30-Fw-prom	CCTTGCGTCTCCCTGCGCGATTTTGTTGGCCATTTTCCAGATCCT GCTTCGTACGCTGCAGGTCG
HSP30-Rev-CYC1	TAAAGCCTCGTTACGATTTAAAAAGCTTGATAGCGTATCGTTCAT TTAGTGTGTGTATTTGTGTTTGC
TPO2-Fw-prom	ACCGATTTCTCGAGATGATTCCATAGCCGTTAAATTCATCTCAAA GCTTCGTACGCTGCAGGTCG
TPO2-Rev-CYC1	AGTGTTTTGTGAGTTGAATGAAACAACAGATTCTTGATCACTCATTTAGTGTGTGTATTTGTGTTTGC
PHO84-Fw-prom	AATCAGTATTACGCACGTTGGTGCTGTTATAGGCGCCCTATACGT GCTTCGTACGCTGCAGGTCG
PHO84-Rev-CYC1	ACTTCTTTCAGCAACATGAATAGTATCTTTATTGACGGAACTCAT TTAGTGTGTGTATTTGTGTTTGC
ALG9-Fw-prom	TTTGATGAGA ACCGTTCTGC GATATTCAGA ACTGTCAATA CAAGCGCTTCGTACGCTGCAGGTCG
ALG9-Rev-CYC1	AAAATAACAACAGTAATAAACTAATGGTTACCGCCTTGCAATTCATTTAGTGTGTGTATTTGTGTTTGC
IGPf1	CGGAATTCAATCAGTATTACGCACGTTGGTGCTG
IGPr	CGCGGATCCTCCATTTGGATTGTATTCGTGGAGTT
IGpTf	CGGAATTCCGCATTTTACTGAACGAGTCATT
IGpTr	CGCGGATCCTCCATATTTGTTTTGTGTATTATTTT
IGpYf3	CGGAATTCCAATTATAGAATCTGTTGACCAAG
IGpYR	CGCGGATCCTCCATTTTGATGCTTTTTTTAAAAAA
PHO84-qPCR-Fw	ACAACCTTG TTGATCCCAG AA
PHO84-qPCR-Rv	TGCTTCATGTTGAAGTTGAGATG
YRO2-qPCR-Fw	TGCCATCTCCAGCTTCTTTC
YRO2-qPCR-Rv	TCCTCCTCTTCTTGGGCTTT
TPO2-qPCR-Fw	TCCATCGACAGTGTTGAGATG
TPO2-qPCR-Rv	TGTGGAAATTTGTTATTTTTGTA
HSP30-qPCR-Fw	CAACCAGACGGTGAGGCTAT
HSP30-qPCR-Rv	TCCGTAGCATGGTGATGAGA
ALG9-qPCR-Fw	ACATCGTCGCCCCAATAAAT
ALG9-qPCR-Rv	GATTGGCTCCGGTTACGTTGC
ACT1-qPCR-Fw	CTGCCGGTATTGACCAAACT
ACT1-qPCR-Rv	CGGTGATTTCCTTTTGCATT
IG-PHO4-Fw-EcoRI	GGGAATTCGTCTCTGTCTAATGCGGTCAC
IG-PHO4-Rv-BamHI	GGGGATCCGTTCTCTCAAATCTTCCAACTGATC
PHO84-AS-cDNA	CTTCCAGCCCATCTCAACTTC
PHO84-SE-cDNA	GAAGTTGAGATGGGCTGGAAG
PHO84-AS-PCR-Fw	GCATAAAAGCCTCAAAGATGC
PHO84-AS-PCR-Rv	TGGCAGAGAGATGTGAGGAA

### Transcriptome analysis by SAGE-tag sequencing

Strain BY4741 was grown overnight at 30°C in Translucent YNB medium containing 50 mM KCl, supplemented with leucine, histidine, methionine and uracil. This culture was used to inoculate two times 50 ml of supplemented YNB medium containing 50 mM KCl yielding A_600nm_ 0.1. These cultures were grown to A_600nm_ 0.5 and cells were isolated by centrifugation. Cells from one culture were washed twice with supplemented YNB medium containing 50 mM KCl and resuspended in 50 ml supplemented YNB medium containing 50 mM KCl. Cells from the other culture were washed twice with supplemented YNB medium lacking KCl and resuspended in 50 ml supplemented YNB medium lacking KCl. Both cultures were incubated at 180 rpm at 30°C. After 60 min the cultures were poured into plastic tubes and immediately frozen in liquid nitrogen. Prior to RNA isolation, the frozen cultures were thawed on ice and cells were collected by centrifugation and transferred to Eppendorf tubes using ice-cold water. RNA was isolated using the Ambion RiboPure yeast RNA Purification Kit (Life Technologies).

Sequence tags were prepared by the Leiden Genome Technology Center (Leiden, the Netherlands) as described [30]. Briefly, 1 μg of total RNA was incubated with oligo-dT beads to capture the polyadenlyated RNA fraction. First- and second-strand cDNA synthesis was performed while the RNA was bound to the beads. While on the beads, samples were digested with *Nla*III to retain a cDNA fragment from the most 3’ CATG to the poly(A)-tail. Subsequently, the GEX adapter 1 was ligated to the free 5’-end of the RNA, and a digestion with *Mme*I was performed, which cuts 17 bp downstream of the CATG site. At this point, the fragments detach from the beads. After dephosphorylation and phenol extraction, the GEX adapter 2 was ligated to the 3’-end of the tag. PCR amplification with 15 cycles using Phusion polymerase was performed with primers complementary to the adapter sequences to enrich the samples for the desired fragments. Sequencing was performed at the Leiden Genome Technology Center on Illumina Hiseq2000 sequencer (Illumina, San Diego, CA, USA). Sequence tags from six samples were bar-coded, mixed and analyzed in a single lane. Obtained sequences were aligned to a virtual tag library obtained by *in silico* digestion by *Nla*III, cutting at 5’-CATG-3’, of *S. cerevisiae* sequences corresponding to open reading frames plus 200 bp downstream sequences using the CLC genomic workbench (Aarhus, Denmark). For each open reading frame the number of aligned reads per million was calculated. This was done independently for each biological replicate. Then, the average expression level with standard deviation was determined for each condition (0 or 50 mM KCl) using the four biological replicates. Statistical analysis was done using the Student’s t-test.

The raw sequence data have been deposited in NCBI’s Gene Expression Omnibus (GEO) and is accessible through GEO Series accession number GSE57093.

### qRT-PCR analysis

qRT-PCR was performed essentially as described earlier [41]. Yeast strains were cultivated in Translucent YNB medium and RNA was isolated as described above. RNA (1 μg) was treated with DNase I (Ambion), according to the recommended protocol, with the addition of 0.5 μl RNasin (Promega) per reaction. From each sample, 0.5 μg was used for subsequent cDNA synthesis with the oligo-dT primer, using an iScript Select cDNA kit (BioRad). Several dilutions of this cDNA were prepared and qRT-PCR was performed in 25 μl reaction volumes using a standard PCR reaction mix for Phusion DNA polymerase (Thermo Scientific), with the addition of 1.25 μl 500× diluted SYBR Green (Life Technologies) in DMSO. PCR efficiency was determined using a series of dilutions of genomic BY4741 DNA. To measure transcript levels of *PHO84*, *YRO2*, *TPO2*, *HSP30* and *ALG9*, primer combinations PHO84-qPCR-Fw/PHO84-qPCR-Rv, YRO2-qPCR-Fw/YRO2-qPCR-Rv, TPO2-qPCR-Fw/TPO2-qPCR-Rv, HSP30-qPCR-Fw /HSP30-qPCR-Rv and ALG9-qPCR-Fw/ALG9-qPCR-Rv, respectively, were used. Transcript levels were normalized against expression of the *ACT1*, measured using the primer combination ACT1-qPCR-Fw/ACT1-qPCR-Rv. Experiments were performed on a Chromo4 Real-Time PCR Detection system controlled by the Opticon Monitor 3.1 software (Biorad).

### Promoter activity assays

Promoter activity was determined by fusion of selected promoter regions to the bacterial ß-galactosidase gene in plasmid pRUL302 as described [42]. Promoter regions of *PHO84*, *TPO2* and *YRO2* were amplified by PCR on chromosomal DNA isolated from BY4741 using the primer combinations IGPf1/IGPr, IGpTf/IGpTr or IGpYf3/IGpYR, respectively, yielding fragments of 600, 510 and 2141 bp, respectively. These fragments were digested with *Bam*H1 and *Eco*RI and ligated in pRUL302 digested with the same enzymes. These constructs were introduced in yeast and transformants were selected for uracil prototrophy. pRS313[PHO4] (pRUL1334), containing the *PHO4* coding region and 578 bp upstream sequences and 500 bp downstream sequences was made by ligating a PCR fragment into pRS313 [43] using the restriction enzymes *Eco*RI and *Bam*HI. The *PHO4* PCR fragment was obtained by PCR using primers IG-PHO4-Fw-EcoRI and IG-PHO4-Rv-BamHI with BY4741 genomic DNA as template. All PCR products were analyzed by DNA sequencing.

Yeast strains carrying the various pRUL302 plasmids were grown in Translucent YNB medium supplemented with histidine, methionine and leucine as described above. Yeast cells were isolated from the frozen cultures after thawing and centrifugation and resuspendend in water. ß-Galactosidase activity was measured using the Yeast β-Galactosidase Assay Kit (Thermo Scientific).

### Assays for growth at low potassium concentrations

Strains were taken from the collection of systematic diploid homozygous deletion strains (Euroscarf, Germany) and were cultivated overnight in Translucent YNB medium supplemented with 50 mM KCl and histidine, uracil, leucine and methionine. Aliquots of these cultures were centrifuged and cells were washed twice with Translucent YNB medium lacking potassium and subsequently resuspended in Translucent YNB medium lacking potassium till A_620_ of 0.5. Ten-fold serial dilutions were made in the same medium and 10 μl aliquots were spotted on plates with Translucent YNB medium with different concentrations of KCl supplemented with histidine, uracil, leucine and methionine. As controls BY4741 and *Δtrk1Δtrk2* in BY4741 were included. Plates were incubated at 30°C for 3 days.

## Electronic supplementary material

Additional file 1: **Effect of potassium starvation on RNA levels determined by SAGE tag sequencing.** (PDF 286 KB)

Additional file 2: **Effect of potassium starvation on antisense transcript levels determined by SAGE tag sequencing.** (PDF 283 KB)

Additional file 3: **Genes of which antisense transcripts are up‒ or down‒regulated more than 2‒fold upon potassium starvation (P<0.01).** (PDF 82 KB)

## Abbreviations

SAGE: Serial analysis of gene expression
qRT-PCR: Quantitative reverse transcriptase PCR.
