# Supplementary material for: Transcriptional response of Saccharomyces cerevisiae to potassium starvation
Source: BMC Genomics. 2014 Nov 29;15(1):1040. doi: 10.1186/1471-2164-15-1040 (PMC4289377; doi:10.1186/1471-2164-15-1040)
Supplement: Supplementary file 1 — Additional file 1: Effect of potassium starvation on RNA levels determined by SAGE tag sequencing. (PDF 286 KB) [file 12864_2014_6863_MOESM1_ESM.pdf]

Additional file 1.
Effect of potassium starvation on RNA levels determined by SAGE tag sequencing.

Annotation	Average 50 mM (reads per million)	SD 50 mM	Average 0 mM (reads per million)	SD 0 mM	Fold-change 0 mM/50 mM	P
AAC1	30,7	18,0	21,2	17,9	0,69	0,484
AAC3	10,6	9,5	25,5	30,0	2,40	0,382
AAD10	33,3	11,2	93,4	36,2	2,81	0,019
AAD14	34,5	11,1	28,4	13,1	0,82	0,504
AAD15	0,1	0,2	0,0	0,0	0,00	0,356
AAD16	9,4	1,6	15,6	11,4	1,66	0,324
AAD3	4,5	1,7	2,9	3,1	0,66	0,413
AAD4	10,1	3,0	21,4	1,8	2,13	0,001
AAD6	2,2	0,9	0,8	0,7	0,36	0,050
AAH1	66,3	31,1	49,8	15,3	0,75	0,378
AAP1	39,1	18,5	60,3	25,6	1,54	0,227
AAR2	0,7	0,5	3,2	2,3	4,39	0,084
AAT1	34,5	7,2	31,9	7,3	0,92	0,629
AAT2	539,6	388,8	240,9	87,4	0,45	0,185
ABD1	87,6	27,6	106,3	35,5	1,21	0,436
ABF1	227,1	97,7	140,5	51,5	0,62	0,168
ABM1	0,2	0,2	2,4	1,6	11,25	0,038
ABP1	107,4	46,0	154,9	91,0	1,44	0,387
ABP140	123,5	67,9	113,5	40,2	0,92	0,808
ABZ1	101,8	36,4	151,4	50,1	1,49	0,161
ABZ2	46,9	13,5	87,1	57,0	1,86	0,219
ACA1	14,2	3,3	17,4	12,0	1,23	0,619
ACB1	383,1	472,3	351,9	470,9	0,92	0,929
ACC1	615,4	117,5	288,9	101,8	0,47	0,006
ACE2	33,1	22,7	35,5	16,1	1,07	0,871
ACF2	25,9	5,3	43,9	9,6	1,70	0,016
ACF4	28,5	19,4	21,9	10,4	0,77	0,570
ACH1	60,0	37,5	209,0	89,0	3,49	0,022
ACK1	26,1	4,1	43,9	39,3	1,68	0,402
ACN9	28,2	10,9	45,1	32,3	1,60	0,360
ACO1	1396,7	969,5	918,4	723,8	0,66	0,459
ACO2	417,1	113,5	214,1	45,6	0,51	0,016
ACP1	513,6	200,5	687,9	245,0	1,34	0,313
ACS1	13,1	1,7	31,1	11,6	2,38	0,021
ACS2	742,6	481,2	283,2	143,2	0,38	0,117
ACT1	1869,8	675,7	2493,5	621,0	1,33	0,223
ADA2	12,4	5,8	5,9	4,0	0,48	0,117
ADD37	0,1	0,2	0,0	0,1	0,31	0,532
ADD66	41,7	26,8	38,7	32,7	0,93	0,889
ADE1	793,3	512,5	326,7	135,8	0,41	0,129
ADE12	1578,2	837,7	370,1	174,6	0,23	0,030
ADE13	1229,6	420,0	616,4	190,5	0,50	0,038

ADE16	117,1	27,4	140,4	47,9	1,20	0,431
ADE17	1852,8	408,1	534,2	250,6	0,29	0,002
ADE2	461,9	133,2	114,2	24,2	0,25	0,002
ADE3	667,1	284,9	796,9	320,6	1,19	0,567
ADE4	528,1	171,7	161,4	83,8	0,31	0,009
ADE5,7	1102,1	492,5	454,3	105,7	0,41	0,042
ADE6	310,5	174,2	211,9	32,7	0,68	0,309
ADE8	729,6	370,3	166,0	77,9	0,23	0,025
ADF1	76,5	48,3	80,7	32,4	1,05	0,891
ADH1	0,5	0,5	0,0	0,0	0,00	0,094
ADH2	5844,7	2612,4	6037,6	4414,0	1,03	0,943
ADH3	712,3	160,1	976,3	297,8	1,37	0,169
ADH4	43,7	14,6	37,8	12,9	0,86	0,567
ADH5	166,2	68,2	273,3	146,4	1,64	0,233
ADH6	208,7	256,5	391,1	504,2	1,87	0,543
ADH7	6,1	3,5	3,1	3,1	0,51	0,241
ADI1	20,4	7,0	54,3	29,3	2,66	0,066
ADK1	1117,5	679,0	1092,1	704,3	0,98	0,960
ADK2	1,2	0,5	0,3	0,3	0,24	0,023
ADO1	850,2	475,9	767,6	378,9	0,90	0,795
ADP1	50,7	16,0	41,9	14,0	0,83	0,439
ADR1	6,1	3,4	31,8	13,8	5,21	0,011
ADY2	0,8	0,3	0,6	0,7	0,71	0,567
ADY3	19,1	5,5	7,2	4,9	0,38	0,018
ADY4	40,7	20,9	54,3	23,4	1,34	0,418
AEP1	26,4	16,6	20,1	15,3	0,76	0,596
AEP2	25,7	11,6	18,2	2,0	0,71	0,250
AEP3	7,8	7,0	7,0	5,2	0,90	0,861
AFG1	38,2	21,5	14,1	11,1	0,37	0,093
AFG2	13,5	11,7	20,0	20,3	1,49	0,595
AFG3	128,3	71,6	104,6	69,8	0,82	0,652
AFI1	21,3	6,4	28,2	11,1	1,32	0,326
AFR1	28,2	9,8	157,4	98,3	5,58	0,040
AFT1	28,9	15,7	32,8	14,9	1,13	0,738
AFT2	3,5	3,2	2,7	2,2	0,78	0,705
AGA1	179,7	113,9	1743,6	1076,5	9,70	0,028
AGA2	50,9	30,0	61,6	17,3	1,21	0,559
AGC1	18,2	7,2	19,8	13,3	1,09	0,835
AGE1	42,2	27,9	18,8	13,5	0,44	0,181
AGE2	85,7	14,1	56,0	15,3	0,65	0,029
AGP1	247,9	153,6	25,2	3,8	0,10	0,027
AGP2	11,5	3,4	32,1	30,4	2,79	0,226
AGP3	0,4	0,3	10,9	20,0	30,53	0,332
AGX1	17,4	11,0	11,9	6,6	0,69	0,431
AHA1	84,3	28,2	224,5	87,2	2,66	0,022
AHC1	28,9	14,9	59,9	47,8	2,08	0,261
AHC2	26,3	9,4	4,8	4,2	0,18	0,006
AHP1	3865,5	2247,9	6134,5	3382,1	1,59	0,307
AHT1	0,4	0,7	0,0	0,1	0,11	0,413
AI1	2,4	2,8	1,9	1,7	0,81	0,787

AI2	1,8	3,3	0,5	0,8	0,28	0,460
AI3	0,3	0,3	0,0	0,1	0,17	0,226
AI4	2,7	0,6	1,8	1,6	0,67	0,324
AI5_ALPHA	22,9	17,2	16,2	15,0	0,71	0,580
AIF1	76,1	50,2	42,0	28,1	0,55	0,281
AIM1	43,1	6,9	27,7	12,8	0,64	0,079
AIM10	23,6	14,3	12,2	8,3	0,52	0,217
AIM11	15,8	7,8	18,5	12,6	1,17	0,727
AIM13	86,6	41,7	69,5	23,3	0,80	0,501
AIM14	57,0	22,4	31,4	18,3	0,55	0,127
AIM17	93,1	54,1	382,3	116,1	4,10	0,004
AIM18	9,6	2,5	4,4	3,1	0,47	0,042
AIM19	20,2	9,8	17,8	3,4	0,88	0,662
AIM2	35,5	14,1	30,9	11,4	0,87	0,632
AIM21	38,8	28,8	53,8	21,3	1,39	0,434
AIM22	11,3	9,4	17,2	19,0	1,52	0,598
AIM23	22,6	10,2	33,7	10,2	1,49	0,175
AIM24	89,3	29,9	85,9	22,8	0,96	0,864
AIM25	19,7	5,7	9,4	6,7	0,48	0,057
AIM26	0,1	0,2	0,0	0,0	0,00	0,356
AIM27	144,9	46,5	110,0	30,1	0,76	0,254
AIM29	138,2	72,7	161,5	92,3	1,17	0,705
AIM3	31,9	21,8	35,7	24,5	1,12	0,826
AIM31	99,5	28,2	101,9	35,8	1,02	0,918
AIM32	10,5	5,5	7,2	5,8	0,68	0,431
AIM33	14,4	8,0	12,7	9,0	0,88	0,783
AIM34	30,2	19,2	6,4	6,5	0,21	0,057
AIM36	27,4	9,6	27,4	4,5	1,00	0,992
AIM37	288,6	157,4	313,6	159,9	1,09	0,831
AIM38	109,9	42,4	77,7	25,6	0,71	0,240
AIM39	21,8	5,0	20,5	6,8	0,94	0,773
AIM4	62,4	25,3	106,0	56,9	1,70	0,211
AIM41	83,6	37,3	203,5	45,5	2,43	0,007
AIM43	31,5	36,4	22,8	18,8	0,73	0,689
AIM44	21,7	17,8	22,7	18,9	1,05	0,940
AIM45	121,7	70,4	66,7	40,6	0,55	0,225
AIM46	40,4	21,4	49,2	42,8	1,22	0,726
AIM5	64,1	33,2	101,9	47,1	1,59	0,238
AIM6	56,6	19,3	80,6	19,5	1,42	0,130
AIM7	288,2	128,6	315,9	157,7	1,10	0,794
AIM9	59,3	11,6	63,7	7,7	1,07	0,549
AIP1	57,3	49,4	105,1	103,5	1,83	0,437
AIR1	27,8	15,0	33,8	30,5	1,21	0,737
AIR2	52,2	40,1	32,6	32,2	0,62	0,474
AKL1	59,1	21,0	60,2	41,2	1,02	0,962
AKR1	174,8	53,9	169,6	24,6	0,97	0,865
AKR2	12,3	2,5	12,2	6,9	0,99	0,974
ALA1	261,0	48,7	176,9	32,8	0,68	0,029
ALB1	116,4	62,5	92,7	22,9	0,80	0,503
ALD2	0,2	0,2	0,3	0,6	2,01	0,616

ALD3	82,9	20,0	217,6	177,6	2,62	0,183
ALD4	355,1	95,0	349,8	35,4	0,99	0,920
ALD5	285,1	111,1	205,4	41,9	0,72	0,228
ALD6	554,5	536,8	214,7	98,1	0,39	0,259
ALE1	66,5	27,3	54,4	41,7	0,82	0,643
ALF1	9,4	3,0	20,8	6,0	2,22	0,014
ALG1	58,8	29,0	31,9	7,3	0,54	0,122
ALG11	39,3	17,3	37,8	11,8	0,96	0,893
ALG12	66,2	18,4	42,3	28,5	0,64	0,209
ALG13	32,9	14,8	58,4	31,2	1,77	0,191
ALG14	76,0	26,2	6,7	4,6	0,09	0,002
ALG2	81,3	34,0	38,3	11,3	0,47	0,053
ALG3	66,1	28,0	50,6	12,9	0,77	0,352
ALG5	47,8	42,6	18,5	12,9	0,39	0,236
ALG6	86,4	22,1	101,9	42,7	1,18	0,543
ALG7	63,5	23,7	10,1	7,6	0,16	0,005
ALG8	41,5	19,6	15,4	2,0	0,37	0,038
ALG9	43,9	11,0	35,2	24,5	0,80	0,544
ALK1	49,9	18,0	46,1	15,5	0,92	0,762
ALK2	80,4	54,4	17,2	12,5	0,21	0,064
ALO1	428,5	148,8	236,7	115,3	0,55	0,088
ALP1	3,8	3,7	0,9	1,1	0,24	0,185
ALR1	78,2	47,2	93,7	45,9	1,20	0,653
ALR2	5,2	2,3	12,1	14,5	2,33	0,381
ALT1	152,5	46,7	257,8	31,2	1,69	0,010
ALT2	44,2	32,8	6,5	7,0	0,15	0,065
ALY1	40,7	20,2	36,8	24,7	0,90	0,812
ALY2	19,1	5,8	32,0	5,7	1,68	0,020
AMA1	2,5	1,7	1,2	0,9	0,48	0,236
AMD1	66,8	30,2	72,4	5,5	1,08	0,730
AMD2	25,6	7,2	21,9	16,6	0,86	0,699
AME1	13,5	5,2	7,1	5,3	0,53	0,139
AMN1	181,0	95,2	219,7	73,1	1,21	0,543
AMS1	59,1	36,9	141,0	46,0	2,39	0,032
ANB1	37,0	29,4	170,3	133,8	4,60	0,100
ANP1	5,8	2,0	5,7	4,3	0,99	0,975
ANS1	1,3	1,6	1,7	1,9	1,32	0,753
ANT1	30,5	11,4	8,7	6,2	0,28	0,015
AOS1	111,0	21,3	85,8	24,8	0,77	0,174
APA1	252,9	146,9	434,3	141,2	1,72	0,125
APA2	26,3	15,2	94,0	57,6	3,57	0,064
APC1	18,3	4,9	21,8	13,6	1,19	0,647
APC11	48,3	14,5	44,0	11,8	0,91	0,658
APC2	20,0	4,0	20,5	13,8	1,03	0,942
APC4	39,8	7,7	18,0	12,7	0,45	0,026
APC5	17,6	9,3	14,3	10,4	0,81	0,650
APC9	50,7	17,4	52,9	10,2	1,04	0,829
APD1	88,4	39,3	110,5	70,9	1,25	0,606
APE2	289,1	185,8	271,7	172,9	0,94	0,895
APE3	535,8	230,2	372,0	158,3	0,69	0,286

API2	0,0	0,0	0,0	0,0	#DIV/0!	#DIV/0!
APJ1	64,1	45,4	125,6	19,6	1,96	0,047
APL1	27,9	10,4	65,8	4,0	2,36	0,000
APL2	38,8	25,1	66,4	29,8	1,71	0,207
APL3	20,8	5,2	35,3	14,6	1,70	0,110
APL4	13,9	7,5	10,8	7,3	0,78	0,576
APL5	91,9	67,8	76,1	55,6	0,83	0,730
APL6	190,2	11,7	145,8	42,3	0,77	0,090
APM1	24,9	4,9	37,5	13,6	1,50	0,132
APM2	34,7	9,2	50,3	21,8	1,45	0,234
APM3	185,5	76,4	143,0	26,3	0,77	0,333
APM4	148,1	49,1	130,8	73,6	0,88	0,709
APN1	79,4	32,7	82,9	50,7	1,04	0,910
APN2	16,1	8,3	20,5	6,6	1,27	0,446
APP1	32,5	9,4	46,9	24,9	1,44	0,321
APQ12	116,9	15,7	100,9	17,7	0,86	0,223
APQ13	17,4	19,1	9,2	7,7	0,53	0,454
APS1	14,8	4,6	22,7	16,8	1,53	0,398
APS2	27,0	9,6	20,1	13,7	0,75	0,442
APS3	63,2	11,1	82,0	18,0	1,30	0,126
APT2	24,2	18,8	31,1	24,3	1,29	0,665
AQR1	147,6	77,0	14,9	4,7	0,10	0,014
AQY1	2,1	1,3	0,9	0,7	0,41	0,154
AQY2	0,3	0,5	0,3	0,4	0,99	0,991
ARA1	99,7	37,9	93,1	53,9	0,93	0,848
ARA2	42,8	18,8	41,0	9,7	0,96	0,874
ARB1	196,1	99,0	153,2	53,8	0,78	0,475
ARC1	920,5	215,7	1311,9	292,4	1,43	0,075
ARC18	298,6	133,2	460,1	314,8	1,54	0,381
ARC19	837,9	317,1	921,2	227,1	1,10	0,684
ARC35	251,5	89,6	302,7	100,7	1,20	0,477
ARC40	97,8	31,9	133,0	34,3	1,36	0,185
ARD1	105,5	47,0	79,6	17,7	0,76	0,343
ARE1	56,0	28,3	27,0	12,9	0,48	0,111
ARE2	4,7	1,2	23,9	10,3	5,13	0,010
ARF1	967,6	507,9	1070,0	540,7	1,11	0,792
ARF2	1044,1	676,7	990,1	541,8	0,95	0,905
ARF3	47,6	16,2	39,1	20,6	0,82	0,541
ARG1	2709,5	1066,5	565,8	337,3	0,21	0,009
ARG2	44,9	19,9	66,9	36,3	1,49	0,329
ARG3	1442,7	892,5	214,4	120,8	0,15	0,034
ARG4	1197,1	788,7	989,4	353,4	0,83	0,648
ARG5,6	852,6	415,2	198,1	105,7	0,23	0,022
ARG7	433,3	324,6	799,0	292,6	1,84	0,145
ARG8	245,3	175,8	68,2	55,9	0,28	0,103
ARG80	60,6	34,7	79,2	53,8	1,31	0,583
ARG81	18,3	19,4	14,1	4,2	0,77	0,688
ARG82	47,8	9,9	16,8	11,2	0,35	0,006
ARH1	39,0	25,6	46,3	39,1	1,19	0,763
ARI1	43,4	60,3	86,4	122,3	1,99	0,551

ARK1	41,7	14,3	22,0	16,4	0,53	0,120
ARL1	122,5	21,0	31,1	4,5	0,25	0,000
ARL3	67,9	34,6	54,3	23,2	0,80	0,538
ARN1	1,4	1,4	1,0	1,0	0,71	0,652
ARN2	52,0	21,9	52,2	37,3	1,00	0,992
ARO1	605,8	482,4	290,6	202,5	0,48	0,274
ARO10	4,2	4,3	3,4	4,2	0,80	0,784
ARO2	462,7	230,7	893,9	586,8	1,93	0,220
ARO3	331,1	246,9	387,5	316,8	1,17	0,788
ARO4	320,9	118,6	194,5	126,1	0,61	0,195
ARO7	81,0	34,0	75,3	22,1	0,93	0,788
ARO8	263,8	62,0	301,3	94,5	1,14	0,532
ARO80	21,1	2,8	28,6	7,2	1,36	0,099
ARO9	1,8	1,0	2,4	2,5	1,35	0,662
ARP1	36,3	18,7	26,6	21,0	0,73	0,513
ARP10	4,4	1,5	2,3	1,7	0,53	0,118
ARP2	257,4	23,7	308,7	70,8	1,20	0,219
ARP3	143,3	50,9	166,9	35,9	1,17	0,476
ARP4	39,1	22,9	24,6	21,2	0,63	0,388
ARP5	97,0	37,1	97,9	44,9	1,01	0,975
ARP6	72,5	29,3	26,7	2,8	0,37	0,021
ARP7	129,9	44,6	112,3	30,3	0,86	0,539
ARP8	26,2	4,9	20,0	13,7	0,76	0,425
ARP9	238,2	94,5	191,7	132,8	0,81	0,590
ARR1	71,4	52,0	56,7	20,7	0,79	0,617
ARR2	3,0	2,4	6,0	6,0	1,98	0,399
ARR3	0,5	0,5	6,1	5,0	12,83	0,068
ART10	31,7	24,8	34,9	21,9	1,10	0,850
ART5	2,0	1,2	1,8	1,3	0,90	0,832
ARV1	26,9	21,2	43,1	49,6	1,60	0,570
ARX1	100,4	52,6	116,7	57,8	1,16	0,691
ASA1	30,2	17,9	80,0	28,5	2,65	0,025
ASC1	6467,0	2685,5	5044,7	2254,4	0,78	0,448
ASE1	8,2	4,8	14,2	13,4	1,74	0,431
ASF1	147,6	112,7	48,4	32,6	0,33	0,142
ASF2	49,1	8,0	20,5	8,3	0,42	0,003
ASG1	100,8	103,5	72,7	64,9	0,72	0,662
ASG7	41,3	19,6	160,3	95,5	3,88	0,050
ASH1	70,7	29,2	57,2	27,0	0,81	0,524
ASI1	40,4	19,5	33,7	22,6	0,83	0,667
ASI2	10,0	2,7	38,3	7,3	3,82	0,000
ASI3	73,7	48,8	46,5	13,3	0,63	0,323
ASK1	23,4	10,9	17,5	15,7	0,75	0,556
ASK10	36,3	17,7	65,7	28,1	1,81	0,127
ASM4	82,2	40,4	94,2	56,9	1,15	0,743
ASN1	944,0	548,9	476,7	240,1	0,50	0,170
ASN2	681,8	144,2	506,0	212,5	0,74	0,220
ASP1	234,0	175,3	215,8	182,8	0,92	0,890
ASP3-1	1170,6	623,1	665,5	394,4	0,57	0,220
ASR1	8,8	3,1	54,5	26,7	6,18	0,014

AST1	3,2	2,5	2,9	2,5	0,90	0,855
AST2	8,8	3,2	20,0	22,7	2,26	0,368
ATC1	39,5	20,2	56,3	45,9	1,42	0,529
ATE1	48,4	28,2	48,4	7,6	1,00	0,999
ATF1	41,2	17,1	24,9	23,4	0,60	0,305
ATF2	63,8	33,9	10,3	8,6	0,16	0,022
ATG1	9,1	4,4	14,4	9,8	1,59	0,354
ATG10	25,2	17,1	11,7	10,8	0,46	0,229
ATG11	12,4	10,9	14,5	12,2	1,17	0,804
ATG12	1,8	1,5	3,8	3,7	2,11	0,359
ATG13	137,5	52,4	107,9	36,5	0,78	0,389
ATG14	16,0	7,4	22,9	6,4	1,43	0,210
ATG15	0,7	0,5	16,8	21,7	23,45	0,188
ATG16	1,9	0,5	4,8	3,8	2,46	0,187
ATG17	19,3	9,0	32,6	15,7	1,69	0,194
ATG18	15,6	9,6	38,1	35,3	2,44	0,264
ATG19	19,1	13,0	37,6	39,5	1,98	0,406
ATG2	60,2	27,5	74,3	27,8	1,23	0,499
ATG20	11,1	6,0	47,6	39,4	4,30	0,116
ATG21	24,8	7,9	34,4	6,3	1,39	0,106
ATG22	27,2	15,9	30,6	5,0	1,13	0,696
ATG23	15,1	5,3	27,5	19,7	1,82	0,269
ATG26	38,4	14,5	69,3	5,1	1,80	0,007
ATG27	153,4	79,7	133,3	71,1	0,87	0,721
ATG3	17,1	9,5	19,8	3,7	1,15	0,625
ATG32	16,3	3,7	8,4	5,7	0,51	0,058
ATG33	50,9	28,1	79,4	13,6	1,56	0,118
ATG34	22,3	4,1	29,2	19,5	1,31	0,517
ATG4	6,3	6,2	5,4	5,9	0,86	0,843
ATG5	39,0	23,5	34,1	12,1	0,87	0,725
ATG7	13,8	7,8	21,6	21,0	1,57	0,512
ATG9	7,9	2,1	25,7	18,5	3,25	0,104
ATH1	18,6	13,4	18,8	2,9	1,01	0,987
ATM1	123,0	56,2	119,4	45,0	0,97	0,924
ATO2	1,3	1,4	8,0	6,6	6,35	0,094
ATO3	177,0	67,9	137,8	23,9	0,78	0,318
ATP1	454,8	355,2	425,3	210,5	0,94	0,891
ATP10	1,1	1,6	0,4	0,4	0,38	0,423
ATP11	64,3	45,3	117,3	118,2	1,82	0,435
ATP12	79,8	27,7	34,7	7,3	0,43	0,020
ATP14	162,4	24,4	208,3	24,6	1,28	0,038
ATP16	214,8	134,1	267,8	137,5	1,25	0,601
ATP17	235,0	159,1	322,9	107,1	1,37	0,395
ATP19	100,5	46,6	160,0	87,3	1,59	0,274
ATP2	937,4	287,1	1742,7	1619,8	1,86	0,365
ATP20	4,3	0,6	0,9	0,9	0,21	0,001
ATP22	10,2	5,8	6,5	4,5	0,63	0,343
ATP23	56,7	18,4	47,9	27,8	0,85	0,618
ATP25	35,9	9,7	27,9	5,0	0,78	0,192
ATP3	132,0	55,0	190,1	65,5	1,44	0,224

ATP4	579,5	170,0	681,6	103,4	1,18	0,345
ATP6	2,1	2,6	0,6	1,3	0,31	0,360
ATR1	131,0	54,3	74,5	26,4	0,57	0,111
ATS1	14,9	6,9	8,6	7,7	0,58	0,272
ATX1	166,6	66,8	146,5	71,3	0,88	0,696
ATX2	45,6	13,5	23,9	8,2	0,52	0,033
AUA1	2,8	2,4	4,4	4,6	1,56	0,569
AUR1	502,8	135,7	131,6	59,1	0,26	0,002
AUS1	25,2	4,2	32,4	11,6	1,29	0,288
AVL9	20,3	15,3	9,5	10,1	0,47	0,283
AVO1	25,6	10,8	18,1	12,7	0,71	0,403
AVO2	12,4	6,0	17,4	11,9	1,41	0,478
AVT1	53,4	70,9	20,0	35,4	0,38	0,433
AVT2	4,8	4,1	6,0	6,6	1,23	0,785
AVT3	80,5	41,7	75,5	14,0	0,94	0,826
AVT4	35,2	20,2	30,8	12,1	0,87	0,717
AVT5	24,3	8,9	29,7	12,4	1,22	0,506
AVT6	53,0	24,0	45,4	33,1	0,86	0,720
AVT7	105,3	62,4	149,3	48,0	1,42	0,307
AXL1	35,1	5,4	71,4	11,1	2,04	0,001
AXL2	70,3	37,1	10,1	7,0	0,14	0,019
AYR1	72,1	63,4	82,8	89,2	1,15	0,851
AYT1	37,0	9,7	36,6	20,1	0,99	0,969
AZF1	16,6	6,9	27,4	4,4	1,65	0,039
AZR1	0,7	0,5	5,6	8,9	8,39	0,310
BAG7	1,7	1,3	1,7	2,0	1,02	0,976
BAP2	239,8	280,9	69,0	19,8	0,29	0,271
BAP3	254,0	111,1	69,0	7,3	0,27	0,016
BAR1	247,7	178,2	541,2	215,4	2,18	0,081
BAS1	51,7	20,3	44,1	31,0	0,85	0,699
BAT1	1724,0	1419,2	1230,2	447,8	0,71	0,532
BAT2	550,4	231,6	1511,0	295,3	2,75	0,002
BBC1	28,8	27,8	27,4	20,0	0,95	0,937
BBP1	96,1	10,4	43,7	37,4	0,45	0,036
BCD1	84,8	24,0	72,2	42,8	0,85	0,626
BCH1	85,2	77,4	71,4	42,9	0,84	0,767
BCH2	18,9	4,0	16,5	11,1	0,87	0,694
BCK1	19,7	18,1	10,7	8,7	0,54	0,405
BCK2	52,7	21,2	92,4	24,3	1,75	0,049
BCP1	119,6	28,8	83,9	18,9	0,70	0,084
BCS1	18,4	2,8	21,1	12,5	1,15	0,681
BCY1	67,3	66,2	105,9	51,8	1,57	0,393
BDF1	127,3	112,4	95,2	66,0	0,75	0,640
BDF2	253,3	100,9	197,1	94,5	0,78	0,447
BDH1	358,4	203,0	376,3	140,6	1,05	0,889
BDH2	15,3	8,4	17,2	17,5	1,12	0,857
BDP1	42,7	26,5	19,9	14,7	0,47	0,183
BDS1	34,5	21,3	24,9	13,0	0,72	0,471
BEM1	32,1	26,3	22,6	24,7	0,70	0,618
BEM2	275,0	170,7	232,5	160,0	0,85	0,729

BEM3	50,1	12,3	25,0	18,7	0,50	0,067
BEM4	3,5	1,3	2,4	1,6	0,69	0,345
BER1	65,2	17,5	103,2	49,5	1,58	0,198
BET1	38,8	23,9	21,0	7,9	0,54	0,208
BET2	83,0	56,7	66,1	31,9	0,80	0,622
BET3	96,0	45,8	81,1	43,7	0,84	0,653
BET4	52,9	10,0	73,8	16,0	1,40	0,069
BET5	53,0	14,1	36,7	12,0	0,69	0,128
BFA1	0,8	0,6	1,3	1,0	1,72	0,372
BFR1	336,6	115,1	357,7	91,9	1,06	0,784
BFR2	79,6	17,2	93,9	42,4	1,18	0,556
BGL2	602,6	485,1	1159,0	926,8	1,92	0,328
BI2	0,5	0,4	0,2	0,2	0,33	0,226
BI3	0,0	0,0	0,1	0,3	#DIV/0!	0,356
BI4	0,0	0,0	0,0	0,1	#DIV/0!	0,356
BIG1	11,6	1,9	24,5	9,2	2,11	0,034
BIK1	58,6	37,6	59,9	44,6	1,02	0,966
BIM1	97,6	111,9	36,0	22,3	0,37	0,321
BIO2	41,4	7,8	65,1	26,9	1,57	0,141
BIO3	7,2	1,7	17,8	20,4	2,48	0,338
BIO4	39,3	31,7	33,0	22,3	0,84	0,758
BIO5	3,3	0,9	1,1	0,7	0,32	0,008
BIR1	33,3	13,8	42,4	9,5	1,27	0,316
BIT2	19,9	8,2	22,2	9,2	1,11	0,731
BIT61	2,7	1,7	3,6	3,4	1,35	0,642
BLI1	66,7	23,0	162,5	155,4	2,44	0,268
BLM10	33,9	16,0	41,3	10,2	1,22	0,463
BLS1	32,3	15,6	59,1	60,3	1,83	0,424
BMH1	4336,8	1873,5	2669,1	1385,9	0,62	0,202
BMH2	433,2	167,9	541,9	163,7	1,25	0,389
BMS1	134,7	85,1	118,2	109,1	0,88	0,820
BNA1	501,2	152,3	863,5	275,3	1,72	0,061
BNA2	26,3	13,2	54,5	21,6	2,08	0,067
BNA3	132,1	68,2	200,3	50,5	1,52	0,159
BNA4	27,7	4,7	15,2	10,9	0,55	0,081
BNA5	34,0	9,7	46,2	23,7	1,36	0,378
BNA6	42,9	19,2	40,8	12,5	0,95	0,856
BNA7	11,9	7,9	11,3	5,1	0,95	0,906
BNI1	69,7	17,1	79,8	11,8	1,15	0,366
BNI4	42,4	21,0	14,5	9,9	0,34	0,053
BNI5	37,7	24,9	20,8	12,6	0,55	0,271
BNR1	73,6	33,5	29,4	7,2	0,40	0,042
BNS1	31,5	12,6	40,0	15,4	1,27	0,428
BOI1	29,6	12,2	16,9	11,5	0,57	0,183
BOI2	51,9	21,7	27,6	13,5	0,53	0,106
BOP2	7,7	4,9	22,1	12,3	2,89	0,072
BOP3	21,5	20,6	25,8	13,0	1,20	0,739
BOR1	20,2	24,7	17,4	20,2	0,86	0,866
BOS1	181,1	84,9	133,2	30,2	0,74	0,329
BPH1	18,0	11,3	28,0	16,6	1,56	0,356

BPL1	25,3	9,2	22,7	5,5	0,90	0,641
BPT1	59,6	19,0	52,3	36,0	0,88	0,735
BRE1	100,0	51,6	67,5	45,3	0,67	0,380
BRE2	94,3	42,5	90,0	27,7	0,95	0,871
BRE4	39,9	24,3	17,5	13,6	0,44	0,158
BRE5	181,4	92,7	186,7	141,1	1,03	0,952
BRF1	67,3	31,4	64,5	27,6	0,96	0,898
BRL1	17,2	9,7	11,6	9,7	0,67	0,448
BRN1	2,4	1,3	0,9	0,8	0,39	0,110
BRO1	33,1	4,2	58,9	18,6	1,78	0,035
BRP1	0,5	0,4	1,7	2,1	3,87	0,268
BRR1	47,0	25,7	29,1	23,0	0,62	0,340
BRR2	49,9	24,4	60,0	13,7	1,20	0,498
BRR6	37,3	21,4	37,1	40,0	0,99	0,992
BRX1	264,2	88,6	165,1	32,5	0,63	0,081
BSC1	0,0	0,0	0,0	0,1	#DIV/0!	0,356
BSC2	8,8	6,6	6,0	5,0	0,67	0,514
BSC4	0,3	0,5	0,6	0,5	2,03	0,438
BSC5	1,0	0,5	2,0	2,1	1,98	0,390
BSC6	32,0	9,6	32,7	10,0	1,02	0,918
BSD2	46,7	33,6	59,1	37,6	1,27	0,640
BSP1	80,1	26,0	123,2	11,5	1,54	0,023
BST1	41,9	29,8	14,0	10,0	0,33	0,127
BTN2	78,5	29,1	207,0	58,5	2,64	0,008
BTS1	15,0	7,3	10,9	7,5	0,73	0,466
BTT1	1,5	0,8	4,3	2,9	2,86	0,111
BUB1	22,3	11,7	4,4	3,2	0,20	0,026
BUB2	20,7	3,7	13,8	3,4	0,67	0,032
BUB3	71,7	36,4	17,9	12,0	0,25	0,031
BUD13	15,2	4,3	17,6	14,3	1,16	0,750
BUD14	78,9	39,7	62,1	30,2	0,79	0,524
BUD16	8,8	8,0	3,6	3,7	0,41	0,280
BUD17	76,2	46,1	68,5	37,2	0,90	0,804
BUD2	31,0	4,5	19,5	3,9	0,63	0,009
BUD20	142,1	88,5	111,7	82,1	0,79	0,633
BUD21	94,1	52,9	120,4	65,0	1,28	0,553
BUD22	88,0	30,6	129,9	85,0	1,48	0,390
BUD23	65,1	24,3	45,0	5,4	0,69	0,158
BUD25	0,6	0,5	0,7	0,8	1,09	0,905
BUD26	64,5	11,9	62,7	14,1	0,97	0,847
BUD27	21,8	17,4	29,0	26,8	1,33	0,668
BUD3	21,7	9,1	13,6	6,1	0,63	0,191
BUD31	3,7	3,0	5,6	5,9	1,53	0,579
BUD32	57,9	13,7	45,0	17,0	0,78	0,283
BUD4	103,0	41,1	31,8	10,6	0,31	0,015
BUD5	23,6	15,2	46,2	41,2	1,96	0,343
BUD6	53,8	25,8	41,2	13,5	0,77	0,422
BUD7	27,5	14,8	30,0	7,1	1,09	0,773
BUD8	66,9	40,4	69,0	23,9	1,03	0,931
BUD9	85,3	69,7	29,3	16,8	0,34	0,169

BUG1	83,7	38,8	86,3	36,1	1,03	0,925
BUL1	22,2	15,6	18,8	12,6	0,85	0,746
BUL2	33,3	13,5	25,6	5,8	0,77	0,331
BUR2	24,3	14,5	19,7	13,7	0,81	0,661
BUR6	93,7	25,8	212,0	55,1	2,26	0,008
BYE1	53,5	16,8	39,4	15,0	0,74	0,257
BZZ1	78,7	25,2	41,5	8,0	0,53	0,031
CAB1	90,3	10,8	128,7	27,9	1,43	0,042
CAB2	70,1	31,9	137,0	86,8	1,95	0,198
CAB3	21,9	15,3	27,6	20,8	1,26	0,673
CAB4	56,3	30,6	70,4	66,6	1,25	0,713
CAB5	170,7	54,8	120,2	21,2	0,70	0,137
CAC2	73,1	36,2	11,9	8,2	0,16	0,016
CAD1	128,8	38,5	247,8	101,8	1,92	0,071
CAF120	20,0	7,3	7,6	5,6	0,38	0,037
CAF130	50,3	17,4	67,6	19,1	1,34	0,229
CAF16	48,8	44,6	53,6	33,2	1,10	0,870
CAF20	627,6	361,6	406,4	188,3	0,65	0,320
CAF4	29,7	19,5	30,6	20,0	1,03	0,950
CAF40	76,5	33,2	68,1	31,0	0,89	0,725
CAJ1	124,9	43,9	182,3	48,9	1,46	0,131
CAK1	15,0	7,9	19,3	12,3	1,29	0,577
CAM1	244,5	99,9	243,7	40,2	1,00	0,989
CAN1	80,2	54,4	19,9	21,4	0,25	0,085
CAP1	171,7	66,5	251,7	101,0	1,47	0,234
CAP2	162,8	80,7	247,3	134,3	1,52	0,322
CAR1	359,6	147,3	344,3	21,1	0,96	0,844
CAR2	22,0	6,5	61,5	21,3	2,80	0,012
CAT2	15,0	6,8	44,8	29,0	2,98	0,092
CAT5	105,0	44,6	111,7	30,4	1,06	0,813
CAT8	5,6	2,2	3,1	2,5	0,56	0,190
CAX4	4,9	2,1	6,9	5,1	1,39	0,508
CBC2	403,1	209,6	124,2	28,2	0,31	0,039
CBF1	47,7	27,6	52,3	37,2	1,10	0,846
CBF2	61,8	29,9	21,3	18,7	0,34	0,061
CBF5	597,6	520,2	737,8	649,2	1,23	0,747
CBK1	30,0	12,0	28,6	19,6	0,95	0,909
CBP1	47,4	11,8	42,6	13,8	0,90	0,612
CBP2	51,3	11,7	54,1	7,6	1,05	0,701
CBP3	20,5	19,9	24,7	30,2	1,20	0,824
CBR1	255,7	343,6	191,2	200,3	0,75	0,757
CBS1	100,3	39,1	84,3	36,2	0,84	0,570
CBS2	76,3	29,5	72,5	24,0	0,95	0,848
CBT1	34,3	20,1	52,9	30,1	1,54	0,343
CCA1	127,8	17,9	169,7	28,7	1,33	0,048
CCC1	86,2	41,3	84,5	17,6	0,98	0,940
CCC2	23,5	16,8	18,3	7,9	0,78	0,599
CCE1	35,6	21,0	34,2	14,2	0,96	0,919
CCH1	37,1	14,7	23,9	18,1	0,64	0,300
CCL1	30,5	12,0	24,8	2,5	0,81	0,388

CCM1	51,4	20,9	43,3	15,9	0,84	0,560
CCP1	132,6	10,7	177,4	103,1	1,34	0,421
CCR4	86,0	25,9	76,4	20,2	0,89	0,580
CCS1	318,5	177,0	244,3	65,4	0,77	0,461
CCT2	246,8	115,6	252,4	129,9	1,02	0,950
CCT3	138,0	38,1	93,2	6,6	0,68	0,060
CCT4	319,3	171,2	310,1	118,9	0,97	0,933
CCT5	239,9	134,4	164,7	99,1	0,69	0,402
CCT6	73,4	22,9	81,3	20,8	1,11	0,628
CCT7	271,1	123,8	182,2	66,9	0,67	0,253
CCT8	585,1	48,6	393,9	140,2	0,67	0,042
CCW14	855,9	327,1	673,9	234,6	0,79	0,401
CCZ1	11,0	7,9	9,6	6,4	0,87	0,786
CDA1	0,3	0,3	1,2	0,9	3,82	0,095
CDA2	10,1	3,1	6,4	2,2	0,63	0,104
CDC1	81,8	50,2	57,2	39,4	0,70	0,470
CDC10	55,4	13,5	69,4	25,4	1,25	0,370
CDC11	46,4	10,4	27,1	12,0	0,58	0,051
CDC12	51,6	23,8	46,4	21,0	0,90	0,756
CDC123	33,3	34,9	19,9	18,9	0,60	0,525
CDC13	19,8	8,0	9,3	7,1	0,47	0,098
CDC14	32,4	11,8	13,4	9,3	0,41	0,044
CDC15	29,7	3,6	35,1	6,8	1,18	0,218
CDC16	43,3	15,2	34,9	12,1	0,81	0,423
CDC19	4876,5	2096,2	9449,2	2997,5	1,94	0,047
CDC20	69,6	21,0	119,0	37,6	1,71	0,062
CDC21	98,3	34,2	8,3	5,9	0,08	0,002
CDC23	19,9	6,1	26,3	19,6	1,32	0,559
CDC24	25,2	13,5	20,8	11,0	0,83	0,636
CDC25	92,1	62,9	85,3	61,6	0,93	0,884
CDC26	25,8	13,8	8,8	5,9	0,34	0,063
CDC27	45,4	10,3	41,1	6,3	0,91	0,504
CDC28	38,5	15,2	36,5	16,6	0,95	0,865
CDC3	92,2	68,4	52,0	42,1	0,56	0,356
CDC33	402,1	141,6	222,8	50,8	0,55	0,054
CDC34	60,6	48,0	76,9	36,5	1,27	0,608
CDC36	41,8	15,0	17,6	12,6	0,42	0,048
CDC37	99,1	41,9	133,8	26,7	1,35	0,212
CDC39	69,5	39,7	80,5	37,1	1,16	0,700
CDC4	54,6	10,3	41,3	7,1	0,76	0,078
CDC40	29,8	11,4	18,1	7,2	0,61	0,133
CDC42	316,7	223,3	220,1	156,7	0,70	0,505
CDC43	21,6	9,6	17,9	13,1	0,83	0,667
CDC45	27,0	9,1	1,9	1,3	0,07	0,002
CDC48	390,4	175,6	389,5	125,5	1,00	0,994
CDC5	84,2	47,0	21,1	14,1	0,25	0,042
CDC50	7,9	4,5	9,7	6,8	1,22	0,690
CDC53	35,6	18,0	49,0	43,0	1,38	0,586
CDC55	46,4	50,3	46,0	17,2	0,99	0,988
CDC6	35,9	12,1	14,3	2,0	0,40	0,013

CDC60	328,4	60,6	216,4	30,4	0,66	0,016
CDC7	20,5	8,6	14,7	10,6	0,72	0,427
CDC73	5,3	3,6	11,5	8,7	2,18	0,237
CDC8	92,5	42,5	42,2	29,8	0,46	0,101
CDC9	73,0	37,7	30,1	5,2	0,41	0,065
CDD1	10,2	6,9	41,5	17,9	4,07	0,017
CDH1	14,9	7,6	24,9	13,3	1,67	0,241
CDS1	49,0	40,5	60,5	44,2	1,23	0,714
CEF1	56,6	28,4	63,5	28,6	1,12	0,744
CEG1	53,0	27,3	22,7	11,1	0,43	0,085
CEM1	25,4	25,7	27,2	24,7	1,07	0,924
CEP3	15,2	7,9	23,1	15,5	1,52	0,396
CET1	51,0	14,7	61,2	12,7	1,20	0,335
CEX1	12,6	4,3	9,9	5,3	0,79	0,460
CFD1	35,2	18,5	36,2	14,6	1,03	0,937
CFT1	13,6	3,8	32,2	11,2	2,37	0,020
CFT2	23,6	15,2	23,9	7,7	1,01	0,973
CGI121	11,4	3,6	14,3	9,1	1,26	0,571
CGR1	216,0	41,4	204,7	75,9	0,95	0,802
CHA1	49,8	32,8	21,9	18,3	0,44	0,188
CHA4	8,0	3,1	9,8	4,7	1,22	0,553
CHC1	97,6	22,9	151,2	46,1	1,55	0,083
CHD1	64,0	9,0	45,5	15,6	0,71	0,086
CHK1	19,1	6,1	16,1	12,3	0,84	0,677
CHL1	25,1	7,5	28,3	13,6	1,13	0,698
CHL4	17,1	9,4	15,1	6,7	0,88	0,730
CHO1	96,1	60,2	64,0	17,3	0,67	0,345
CHO2	98,6	27,5	66,6	35,0	0,68	0,201
CHS1	58,3	24,6	90,5	15,7	1,55	0,069
CHS2	63,3	26,7	82,5	27,4	1,30	0,354
CHS3	46,9	28,8	36,9	16,7	0,79	0,573
CHS5	293,7	109,2	267,4	114,3	0,91	0,751
CHS6	8,1	4,6	14,4	11,6	1,79	0,347
CHS7	56,8	28,5	40,7	21,7	0,72	0,404
CHZ1	53,1	13,4	19,8	13,9	0,37	0,014
CIA1	125,1	32,5	57,4	18,3	0,46	0,011
CIC1	129,7	67,6	110,4	52,4	0,85	0,668
CIK1	13,8	5,2	27,8	16,9	2,01	0,166
CIN1	8,9	5,6	5,4	3,6	0,60	0,327
CIN2	120,2	74,3	22,3	16,4	0,19	0,042
CIN5	18,7	19,2	59,7	64,3	3,20	0,267
CIN8	45,7	24,8	17,4	6,6	0,38	0,070
CIS1	23,8	15,7	41,2	28,3	1,73	0,324
CIS3	780,5	167,0	383,8	146,5	0,49	0,012
CIT1	236,0	26,6	190,1	73,1	0,81	0,283
CIT2	1724,8	722,9	2821,0	714,3	1,64	0,074
CIT3	6,6	3,2	75,3	26,4	11,38	0,002
CKA1	73,2	38,4	115,0	69,1	1,57	0,331
CKA2	165,2	111,3	150,4	108,4	0,91	0,855
CKB1	88,5	60,8	90,3	88,6	1,02	0,975

CKB2	127,5	31,9	95,3	19,9	0,75	0,138
CKI1	8,8	4,3	12,1	3,2	1,37	0,267
CKS1	174,5	75,7	51,5	29,1	0,30	0,023
CLA4	40,1	19,1	40,8	7,2	1,02	0,950
CLB1	40,5	12,1	11,3	9,9	0,28	0,010
CLB2	2,1	2,3	10,1	13,0	4,78	0,270
CLB3	43,4	22,0	34,0	12,2	0,78	0,481
CLB4	19,2	6,1	13,4	3,8	0,70	0,158
CLB5	97,0	16,9	38,9	4,7	0,40	0,001
CLB6	22,6	9,7	0,3	0,4	0,01	0,004
CLC1	133,3	153,6	121,8	121,8	0,91	0,911
CLD1	11,6	8,9	7,9	8,4	0,68	0,562
CLF1	32,3	21,8	40,7	27,5	1,26	0,650
CLG1	374,4	186,4	690,4	357,8	1,84	0,168
CLN1	87,2	21,0	30,2	36,0	0,35	0,034
CLN2	150,6	20,5	17,1	11,0	0,11	0,000
CLN3	84,7	55,5	112,8	94,1	1,33	0,625
CLP1	2,8	1,1	1,7	1,3	0,62	0,256
CLU1	260,2	131,3	208,0	69,7	0,80	0,509
CMC2	142,5	89,7	91,5	100,0	0,64	0,476
CMC4	27,9	8,6	97,6	15,5	3,50	0,000
CMK1	22,9	19,4	37,1	44,7	1,62	0,580
CMK2	93,8	51,3	122,5	27,5	1,31	0,362
CMP2	83,9	20,5	99,9	15,1	1,19	0,255
CMS1	118,8	86,5	83,5	57,1	0,70	0,521
CNA1	38,8	27,5	23,3	17,1	0,60	0,375
CNB1	47,8	28,1	76,0	55,8	1,59	0,402
CNE1	67,4	18,7	46,2	32,3	0,68	0,298
CNM67	36,4	17,5	17,7	5,2	0,49	0,087
CNN1	11,5	4,4	5,4	3,9	0,47	0,082
CNS1	63,4	4,9	106,6	10,2	1,68	0,000
COA1	47,0	20,7	57,4	32,8	1,22	0,611
COA2	33,9	17,3	16,0	11,9	0,47	0,139
COA3	90,1	70,4	117,0	88,7	1,30	0,651
COA4	52,8	29,9	115,1	93,9	2,18	0,253
COB	6,1	8,3	1,9	3,1	0,31	0,376
COF1	2021,5	1039,4	2482,1	941,8	1,23	0,536
COG1	39,9	26,5	35,7	25,3	0,89	0,825
COG2	33,2	20,6	39,3	29,0	1,18	0,743
COG3	52,4	21,3	46,3	5,2	0,88	0,601
COG4	14,9	6,9	11,4	8,2	0,76	0,535
COG5	26,4	7,8	8,4	6,1	0,32	0,011
COG6	61,8	26,5	43,8	30,6	0,71	0,408
COG7	149,2	56,5	230,6	61,0	1,55	0,098
COG8	122,7	75,9	80,6	51,6	0,66	0,394
COP1	386,9	129,2	251,3	120,5	0,65	0,176
COQ1	30,7	21,3	33,8	18,8	1,10	0,835
COQ10	48,1	21,7	47,2	7,3	0,98	0,941
COQ2	34,3	11,2	24,8	3,8	0,72	0,160
COQ3	71,1	17,6	34,1	13,2	0,48	0,015

COQ4	40,9	28,3	60,0	56,0	1,47	0,564
COQ5	254,4	55,9	342,6	173,5	1,35	0,371
COQ6	43,6	47,1	35,8	18,9	0,82	0,770
COQ8	15,1	4,5	11,1	5,3	0,73	0,293
COQ9	116,4	37,8	127,5	83,6	1,10	0,817
COR1	190,0	71,5	338,0	57,1	1,78	0,018
COS1	84,0	56,3	51,4	34,3	0,61	0,361
COS10	1,9	1,6	0,2	0,4	0,10	0,082
COS111	8,1	8,6	31,6	16,4	3,90	0,044
COS12	2,7	1,6	0,6	0,8	0,21	0,055
COS2	150,3	59,0	124,0	54,3	0,82	0,535
COS4	0,3	0,3	0,0	0,0	0,00	0,144
COS6	44,5	10,8	6,3	4,8	0,14	0,001
COS7	2,1	1,1	5,5	4,6	2,65	0,199
COS8	17,3	6,2	14,1	3,3	0,82	0,404
COS9	57,4	20,5	51,4	24,5	0,90	0,721
COT1	26,8	9,8	31,0	16,8	1,16	0,680
COX1	142,9	188,4	19,0	12,8	0,13	0,237
COX10	10,6	4,2	9,8	7,0	0,92	0,850
COX11	30,0	10,0	10,5	7,1	0,35	0,019
COX12	169,3	60,3	53,6	34,3	0,32	0,016
COX13	339,0	130,7	746,7	187,4	2,20	0,012
COX14	245,6	124,7	409,5	188,6	1,67	0,197
COX15	17,4	9,8	23,7	15,9	1,36	0,523
COX17	130,7	84,9	213,1	181,1	1,63	0,441
COX18	17,2	13,7	23,8	10,4	1,38	0,475
COX19	25,9	0,8	38,5	11,1	1,49	0,064
COX2	0,5	0,4	0,0	0,1	0,08	0,062
COX20	90,3	18,9	84,6	15,7	0,94	0,656
COX23	2,2	3,1	6,4	6,0	2,97	0,252
COX3	0,2	0,4	0,0	0,1	0,20	0,464
COX4	50,0	30,8	104,8	94,8	2,09	0,314
COX5A	119,4	65,1	168,8	52,9	1,41	0,283
COX5B	118,1	73,7	200,6	186,4	1,70	0,442
COX6	112,9	84,8	131,6	96,8	1,17	0,782
COX8	118,2	69,9	163,4	83,3	1,38	0,438
COX9	138,4	133,2	181,2	126,3	1,31	0,657
COY1	65,2	22,3	110,9	23,3	1,70	0,030
CPA1	147,9	23,7	161,9	134,4	1,10	0,844
CPA2	277,8	224,5	130,5	70,4	0,47	0,257
CPD1	5,6	2,3	5,9	4,9	1,06	0,900
CPR1	48,3	20,1	59,2	25,2	1,23	0,524
CPR2	69,4	42,9	108,7	62,6	1,56	0,341
CPR3	82,5	32,6	101,8	40,5	1,23	0,486
CPR4	211,4	111,0	417,6	197,9	1,98	0,119
CPR5	159,4	89,3	198,7	91,7	1,25	0,562
CPR6	127,5	85,6	380,3	253,9	2,98	0,108
CPR7	44,6	16,1	27,8	19,9	0,62	0,237
CPR8	36,5	13,2	17,4	14,0	0,48	0,094
CPS1	36,4	9,6	26,7	18,1	0,73	0,378

CPT1	159,0	90,2	90,1	25,8	0,57	0,192
CRC1	6,1	4,4	8,4	6,9	1,38	0,590
CRD1	35,6	15,4	31,9	5,6	0,90	0,667
CRF1	4,8	2,2	2,8	2,2	0,58	0,240
CRH1	312,4	124,6	73,5	14,6	0,24	0,009
CRM1	319,5	125,3	154,9	57,6	0,48	0,054
CRN1	113,6	43,7	70,3	29,8	0,62	0,153
CRP1	423,9	242,8	324,8	177,5	0,77	0,534
CRR1	5,5	3,6	3,8	2,7	0,69	0,476
CRS5	37,1	14,1	22,5	6,4	0,61	0,108
CRT10	45,2	27,0	62,8	53,3	1,39	0,577
CRZ1	26,1	7,0	16,2	10,9	0,62	0,177
CSE1	133,6	59,7	164,0	90,1	1,23	0,595
CSE4	35,6	15,5	42,9	38,6	1,20	0,738
CSF1	28,3	21,2	45,2	18,5	1,60	0,276
CSG2	92,6	26,7	85,3	39,4	0,92	0,771
CSH1	135,3	54,7	81,2	13,4	0,60	0,103
CSI1	4,6	3,4	4,0	2,7	0,86	0,776
CSI2	17,3	9,0	1,3	1,1	0,08	0,013
CSL4	23,3	28,7	26,8	43,7	1,15	0,899
CSM1	1,6	1,2	1,7	1,9	1,05	0,949
CSM2	2,3	1,6	1,3	1,1	0,53	0,300
CSM3	29,1	13,9	5,4	4,3	0,18	0,018
CSM4	0,0	0,0	0,0	0,0	#DIV/0!	#DIV/0!
CSN12	68,0	48,7	92,5	67,3	1,36	0,577
CSN9	16,2	7,6	6,2	4,4	0,38	0,062
CSR1	195,8	130,0	79,5	53,5	0,41	0,149
CSR2	9,8	3,0	24,4	9,3	2,50	0,025
CST26	68,5	33,8	25,4	10,9	0,37	0,051
CST6	39,7	12,3	57,6	26,1	1,45	0,261
CST9	34,9	23,2	37,1	27,2	1,06	0,904
CTA1	4,7	1,8	8,3	6,9	1,78	0,345
CTF13	6,2	3,5	15,4	9,1	2,48	0,108
CTF18	22,7	11,3	4,2	4,3	0,19	0,022
CTF19	0,9	0,8	0,5	0,6	0,56	0,448
CTF3	5,6	1,8	9,6	14,6	1,70	0,612
CTF4	47,0	11,7	10,9	7,6	0,23	0,002
CTH1	44,5	12,9	62,8	10,9	1,41	0,073
CTI6	25,8	3,4	22,4	15,9	0,87	0,687
CTK1	61,0	65,2	57,9	56,4	0,95	0,946
CTK2	56,0	31,1	39,2	3,8	0,70	0,324
CTK3	40,7	27,1	54,6	22,1	1,34	0,458
CTL1	70,8	19,2	119,4	22,5	1,69	0,017
CTM1	8,0	2,9	9,8	7,6	1,22	0,677
CTP1	265,0	99,7	61,5	15,9	0,23	0,007
CTR1	29,5	13,4	40,3	17,1	1,36	0,360
CTR2	100,1	35,3	83,6	22,4	0,83	0,459
CTR3	4,1	1,8	5,3	5,3	1,28	0,696
CTR86	25,3	3,1	18,6	4,4	0,74	0,048
CTR9	69,9	25,5	70,3	47,3	1,01	0,987

CTS1	1062,8	390,6	945,8	325,4	0,89	0,662
CTS2	23,1	5,0	20,0	8,3	0,86	0,545
CTT1	10,4	4,3	34,9	24,0	3,34	0,091
CUE1	193,9	38,6	140,3	11,3	0,72	0,037
CUE2	14,7	3,6	14,5	4,4	0,98	0,938
CUE3	44,8	29,6	58,7	33,3	1,31	0,556
CUE4	137,3	21,8	44,9	21,5	0,33	0,001
CUE5	132,2	80,6	344,0	204,6	2,60	0,102
CUL3	24,6	14,0	36,7	10,5	1,49	0,214
CUP1-1	2288,4	1096,9	2802,8	991,5	1,22	0,513
CUP2	20,2	9,1	11,2	9,8	0,56	0,227
CUP9	473,7	237,2	191,9	131,9	0,41	0,083
CUR1	44,7	17,1	87,9	32,7	1,97	0,057
CUS1	11,7	3,9	28,6	11,8	2,45	0,034
CUS2	26,6	13,9	11,6	8,3	0,44	0,114
CWC15	131,3	52,9	178,4	64,7	1,36	0,302
CWC2	25,4	11,9	22,9	15,9	0,90	0,816
CWC21	39,7	27,4	43,6	28,5	1,10	0,850
CWC22	37,1	17,8	29,3	12,2	0,79	0,495
CWC24	31,1	22,2	31,9	28,9	1,03	0,965
CWC25	6,7	2,5	2,4	1,6	0,36	0,027
CWC27	49,7	13,7	66,1	15,3	1,33	0,161
CWH41	82,6	33,0	35,2	20,3	0,43	0,050
CWH43	164,2	58,6	149,3	64,4	0,91	0,745
CWP1	253,0	92,4	159,5	128,4	0,63	0,282
CYB2	5,0	1,5	5,5	4,0	1,10	0,812
CYC1	73,5	27,9	60,2	34,5	0,82	0,571
CYC2	14,5	9,8	13,0	14,0	0,90	0,872
CYC3	24,7	11,5	86,9	59,7	3,52	0,087
CYC7	75,0	14,2	88,8	31,5	1,18	0,454
CYC8	176,6	4,9	81,9	18,4	0,46	0,000
CYK3	39,7	5,6	34,7	15,4	0,87	0,566
CYM1	28,5	8,8	30,3	20,8	1,06	0,879
CYR1	46,1	8,7	84,9	15,2	1,84	0,004
CYS3	368,4	214,6	543,4	183,6	1,48	0,261
CYS4	6,3	8,6	1,1	1,0	0,18	0,274
CYT1	40,1	44,2	38,6	42,0	0,96	0,962
CYT2	37,1	11,4	44,5	28,4	1,20	0,644
DAD4	83,3	39,6	111,5	70,5	1,34	0,512
DAK1	163,1	117,3	236,1	107,8	1,45	0,395
DAK2	0,8	0,9	5,8	4,8	7,60	0,085
DAL1	1,7	0,5	0,4	0,9	0,25	0,037
DAL2	29,0	6,7	31,0	5,7	1,07	0,664
DAL3	21,8	28,4	1,7	1,9	0,08	0,207
DAL4	1,4	1,3	1,2	1,3	0,85	0,819
DAL5	7,1	5,0	3,5	3,5	0,50	0,292
DAL7	23,3	11,6	20,4	5,7	0,88	0,672
DAL80	1,5	1,0	0,0	0,1	0,02	0,029
DAL81	61,6	40,3	55,8	21,3	0,91	0,808
DAL82	24,3	4,2	18,4	12,5	0,76	0,408

DAM1	34,6	26,3	19,0	15,0	0,55	0,340
DAN1	5,2	4,4	5,6	5,4	1,07	0,920
DAN4	9,6	3,5	4,4	3,0	0,46	0,067
DAP1	107,6	62,6	221,0	92,9	2,05	0,089
DAP2	56,8	25,8	46,4	34,3	0,82	0,647
DAS1	13,4	7,8	12,7	11,0	0,95	0,926
DAS2	56,2	29,2	41,7	18,3	0,74	0,431
DAT1	21,5	19,3	17,1	12,0	0,80	0,715
DBF2	90,3	24,0	85,5	25,8	0,95	0,797
DBF20	55,8	14,8	36,1	25,6	0,65	0,232
DBF4	98,6	76,8	47,4	18,9	0,48	0,243
DBP1	5,6	0,6	14,8	9,9	2,63	0,113
DBP10	83,6	61,5	104,7	70,8	1,25	0,669
DBP2	1451,8	937,5	133,9	82,3	0,09	0,031
DBP3	71,9	47,2	96,0	64,4	1,33	0,569
DBP5	99,2	35,1	62,9	12,1	0,63	0,098
DBP6	63,0	27,6	36,0	11,9	0,57	0,123
DBP7	46,1	15,5	33,7	11,9	0,73	0,250
DBP8	29,2	5,6	23,5	6,2	0,81	0,225
DBP9	119,2	38,1	76,2	17,0	0,64	0,085
DBR1	51,6	29,4	67,3	46,5	1,30	0,590
DCC1	7,0	2,8	4,2	3,8	0,60	0,280
DCD1	70,8	30,4	24,9	17,6	0,35	0,040
DCG1	13,4	7,8	2,2	1,8	0,17	0,032
DCN1	15,8	5,9	19,9	16,6	1,26	0,658
DCP1	86,5	61,4	42,2	19,4	0,49	0,217
DCP2	110,7	29,8	161,1	9,4	1,46	0,018
DCR2	35,4	15,7	39,1	20,1	1,10	0,782
DCS1	256,5	54,7	515,1	171,2	2,01	0,028
DCS2	50,4	14,2	183,7	64,2	3,65	0,007
DCW1	373,9	178,6	322,8	205,7	0,86	0,720
DDC1	19,4	9,0	20,6	10,0	1,06	0,872
DDI1	70,8	7,8	112,4	31,8	1,59	0,044
DDI2	2,5	1,8	3,8	3,1	1,51	0,499
DDP1	111,9	26,6	168,0	68,6	1,50	0,178
DED1	62,8	59,4	33,4	33,8	0,53	0,424
DED81	580,0	251,1	681,4	272,6	1,17	0,604
DEF1	389,0	304,6	189,3	148,2	0,49	0,283
DEG1	28,5	11,3	14,1	9,7	0,49	0,101
DEP1	25,1	2,6	31,4	6,5	1,25	0,119
DER1	21,6	11,2	15,6	11,2	0,73	0,483
DET1	45,6	11,2	59,8	51,0	1,31	0,606
DFG10	21,9	12,6	25,8	28,3	1,18	0,809
DFG16	2,4	0,3	6,3	5,2	2,59	0,191
DFG5	56,8	11,8	20,2	5,6	0,36	0,001
DFM1	65,3	9,6	95,6	83,2	1,46	0,496
DFR1	57,5	34,8	25,5	11,4	0,44	0,131
DGA1	28,2	14,2	24,4	9,8	0,86	0,674
DGK1	50,2	15,6	41,9	13,0	0,83	0,444
DGR1	0,1	0,1	0,2	0,2	1,83	0,563

DGR2	43,4	6,9	56,5	15,5	1,30	0,172
DHH1	21,1	17,4	38,1	19,7	1,81	0,242
DHR2	19,1	7,7	8,6	6,4	0,45	0,080
DIA1	33,6	22,1	64,3	45,1	1,91	0,268
DIA2	49,8	15,2	33,3	9,4	0,67	0,113
DIA3	5,4	4,0	9,9	7,0	1,81	0,312
DIA4	11,2	6,5	6,2	4,6	0,56	0,258
DIB1	42,3	15,8	50,0	10,1	1,18	0,444
DIC1	107,9	121,2	43,9	21,5	0,41	0,338
DID2	131,6	78,3	168,8	109,7	1,28	0,601
DID4	89,8	27,6	104,3	34,9	1,16	0,539
DIE2	26,4	9,5	43,2	5,7	1,64	0,023
DIG1	43,1	16,9	47,7	9,1	1,11	0,648
DIG2	58,0	11,8	59,1	8,6	1,02	0,885
DIM1	112,3	49,2	79,9	35,9	0,71	0,328
DIN7	7,2	3,8	1,4	1,9	0,20	0,034
DIP2	112,1	101,2	122,0	71,1	1,09	0,878
DIP5	172,9	77,5	65,3	32,9	0,38	0,043
DIS3	35,6	23,6	20,1	13,7	0,56	0,299
DIT1	1,8	0,9	2,2	1,6	1,21	0,688
DIT2	21,1	17,9	9,4	9,8	0,44	0,293
DJP1	109,5	47,6	142,5	18,5	1,30	0,245
DLD1	351,1	119,8	287,6	89,3	0,82	0,428
DLD2	76,7	103,1	88,3	140,5	1,15	0,899
DLD3	529,2	546,6	911,7	944,3	1,72	0,510
DLS1	63,7	6,0	62,2	36,2	0,98	0,935
DLT1	41,0	11,5	19,6	3,3	0,48	0,012
DMA1	54,7	23,0	31,9	7,2	0,58	0,107
DMA2	63,2	29,0	50,1	18,3	0,79	0,473
DMC1	4,2	3,8	4,5	3,4	1,07	0,907
DML1	26,1	12,8	17,0	12,1	0,65	0,345
DNA2	37,6	19,2	33,3	12,0	0,89	0,718
DNF1	93,3	18,9	99,7	32,1	1,07	0,744
DNF2	5,0	0,6	3,8	2,5	0,76	0,401
DNF3	70,1	22,6	52,4	17,5	0,75	0,262
DNL4	7,9	3,9	10,4	7,4	1,32	0,565
DNM1	23,2	7,7	27,6	22,3	1,19	0,727
DOA1	102,8	31,6	143,4	59,6	1,39	0,274
DOA4	9,5	6,3	35,4	3,9	3,73	0,000
DOC1	1,4	1,0	3,5	2,5	2,56	0,167
DOG1	1,5	1,5	0,1	0,3	0,09	0,121
DOG2	26,9	13,0	35,3	18,3	1,31	0,485
DOM34	82,7	15,0	59,1	3,4	0,71	0,022
DON1	8,2	3,0	2,7	2,0	0,33	0,022
DOP1	82,3	14,4	74,7	14,1	0,91	0,480
DOS2	111,5	78,3	160,0	115,5	1,43	0,513
DOT1	98,1	16,1	58,7	19,3	0,60	0,020
DOT6	309,0	234,2	348,8	183,7	1,13	0,798
DPB11	29,7	24,3	14,5	12,0	0,49	0,307
DPB2	45,1	43,0	53,2	62,2	1,18	0,838

DPB3	31,4	20,5	15,6	10,7	0,50	0,220
DPB4	35,9	12,0	14,2	11,0	0,40	0,037
DPH1	39,6	14,2	39,2	30,9	0,99	0,982
DPH2	105,8	50,9	82,6	27,8	0,78	0,454
DPH5	199,9	127,3	213,7	165,4	1,07	0,899
DPL1	130,2	20,3	183,9	30,9	1,41	0,027
DPM1	275,4	95,3	286,8	59,2	1,04	0,847
DPP1	290,1	38,7	222,5	58,7	0,77	0,103
DPS1	86,8	77,8	373,6	608,5	4,31	0,386
DRE2	45,8	20,5	69,5	9,2	1,52	0,080
DRS1	80,5	29,6	69,6	11,3	0,86	0,517
DRS2	45,3	28,0	30,2	18,9	0,67	0,405
DSD1	45,9	8,3	60,1	41,5	1,31	0,527
DSE1	28,8	23,6	31,9	18,5	1,10	0,847
DSE2	385,0	281,1	283,2	77,0	0,74	0,511
DSE3	170,5	45,2	102,3	46,1	0,60	0,079
DSE4	68,8	46,6	46,3	52,8	0,67	0,546
DSF1	1,5	0,7	2,0	1,6	1,27	0,658
DSF2	17,9	4,4	13,6	2,8	0,76	0,151
DSK2	433,7	172,2	657,1	209,0	1,52	0,150
DSL1	204,3	71,2	214,3	119,6	1,05	0,891
DSS1	58,6	10,8	68,9	34,9	1,18	0,592
DSS4	90,0	24,1	49,3	11,5	0,55	0,022
DST1	112,4	22,5	131,4	17,9	1,17	0,235
DTD1	152,4	47,6	63,6	64,0	0,42	0,068
DTR1	0,3	0,4	0,3	0,5	1,00	0,997
DUG1	558,1	93,9	790,8	16,2	1,42	0,003
DUG2	27,8	7,8	16,6	11,2	0,60	0,154
DUG3	39,5	18,2	49,3	29,8	1,25	0,598
DUN1	180,3	43,2	39,1	8,4	0,22	0,001
DUO1	41,3	11,7	27,7	20,2	0,67	0,288
DUR1,2	88,4	15,4	20,2	9,7	0,23	0,000
DUR3	25,9	6,2	5,8	4,9	0,22	0,002
DUS1	46,8	32,7	34,8	13,5	0,74	0,523
DUS3	117,2	69,0	116,5	91,7	0,99	0,990
DUS4	33,5	19,7	14,5	7,5	0,43	0,121
DUT1	1,0	0,9	0,0	0,0	0,00	0,086
DYN1	6,2	3,8	12,0	8,8	1,93	0,271
DYN2	60,0	30,4	39,6	10,0	0,66	0,250
DYN3	85,3	19,4	88,2	88,1	1,03	0,951
DYS1	220,7	59,0	265,4	62,2	1,20	0,337
EAF1	21,8	9,1	18,3	13,8	0,84	0,684
EAF3	66,7	17,1	54,0	5,5	0,81	0,205
EAF5	211,4	167,6	174,3	73,1	0,82	0,699
EAF6	57,3	14,3	73,1	32,3	1,28	0,404
EAF7	45,1	39,5	64,0	23,7	1,42	0,445
EAP1	112,5	40,5	135,4	69,1	1,20	0,589
EAR1	34,8	10,0	51,5	10,0	1,48	0,056
EBP2	148,5	65,0	181,7	98,5	1,22	0,594
EBS1	113,5	15,1	63,7	24,7	0,56	0,014

ECI1	11,0	4,4	3,7	2,7	0,34	0,031
ECL1	99,4	49,2	549,1	168,8	5,52	0,002
ECM1	93,0	87,6	71,4	11,7	0,77	0,642
ECM10	1,0	1,2	0,2	0,3	0,25	0,269
ECM11	13,6	11,7	3,5	3,8	0,26	0,152
ECM12	7,8	5,2	7,3	5,2	0,94	0,909
ECM13	1,0	0,7	2,1	1,7	2,01	0,296
ECM14	110,9	38,2	126,3	56,4	1,14	0,667
ECM15	420,6	234,0	572,0	366,3	1,36	0,512
ECM16	40,0	19,7	52,3	37,0	1,31	0,580
ECM18	3,3	0,8	10,2	7,4	3,12	0,112
ECM19	18,3	6,4	18,1	7,0	0,99	0,964
ECM2	8,0	7,0	27,2	12,8	3,39	0,039
ECM21	59,5	41,9	56,7	31,0	0,95	0,917
ECM22	13,8	7,6	14,6	4,4	1,05	0,872
ECM25	46,8	20,2	27,8	9,6	0,59	0,140
ECM27	2,5	1,9	3,0	2,9	1,19	0,794
ECM29	45,5	16,1	73,4	27,9	1,61	0,135
ECM3	101,0	47,4	67,5	30,0	0,67	0,277
ECM30	49,2	23,4	50,8	18,8	1,03	0,921
ECM31	30,2	27,1	30,4	35,8	1,00	0,996
ECM32	52,0	12,4	106,9	15,7	2,06	0,002
ECM33	1312,7	960,2	700,1	527,2	0,53	0,306
ECM34	0,3	0,6	1,4	1,5	5,16	0,201
ECM38	58,7	17,7	48,1	52,2	0,82	0,714
ECM4	9,8	2,7	13,8	4,5	1,41	0,173
ECM5	20,5	4,0	14,5	9,8	0,71	0,304
ECM7	24,9	11,3	20,4	12,0	0,82	0,604
ECM8	0,7	0,6	0,3	0,4	0,47	0,361
ECM9	19,2	2,4	15,0	10,3	0,78	0,460
ECO1	13,6	12,6	6,5	9,0	0,48	0,393
ECT1	87,9	83,7	112,5	110,3	1,28	0,734
EDC1	3,3	2,9	4,0	3,7	1,23	0,756
EDC2	36,7	9,1	73,9	14,1	2,02	0,004
EDC3	67,2	23,1	49,5	21,5	0,74	0,304
EDE1	121,6	52,8	194,3	19,9	1,60	0,042
EDS1	13,4	2,5	44,7	13,1	3,33	0,003
EEB1	14,0	9,3	8,2	4,4	0,59	0,303
EFB1	2239,7	1325,2	2642,3	1234,0	1,18	0,672
EFG1	94,4	18,5	85,9	28,4	0,91	0,633
EFM1	709,9	527,9	545,4	437,3	0,77	0,648
EFR3	103,6	19,8	58,0	24,7	0,56	0,028
EFT1	5,0	1,5	7,4	7,3	1,48	0,542
EFT2	3827,6	2275,9	3522,5	2065,3	0,92	0,849
EGD1	1637,0	648,2	2062,8	654,5	1,26	0,391
EGD2	1543,2	844,5	1624,1	902,9	1,05	0,900
EGT2	198,8	124,8	153,6	121,9	0,77	0,623
EHD3	66,4	49,1	51,4	34,9	0,77	0,636
EHT1	143,4	82,1	113,9	25,9	0,79	0,519
EKI1	39,8	8,6	37,3	34,7	0,94	0,891

ELA1	0,9	1,1	9,3	14,9	10,13	0,304
ELF1	157,8	61,1	218,0	69,2	1,38	0,240
ELG1	42,3	23,8	17,5	12,3	0,41	0,114
ELM1	20,7	21,6	22,2	22,0	1,08	0,923
ELO1	390,8	218,0	121,2	106,8	0,31	0,068
ELP2	126,0	53,2	80,5	39,3	0,64	0,218
ELP3	49,0	61,5	24,8	12,7	0,51	0,471
ELP4	95,5	28,6	60,6	32,5	0,63	0,158
ELP6	64,3	50,3	46,9	30,9	0,73	0,577
EMC1	79,3	33,3	57,5	10,0	0,72	0,256
EMC2	57,1	18,1	58,4	10,0	1,02	0,905
EMC4	192,5	63,1	173,7	44,2	0,90	0,642
EMC6	431,2	215,6	257,6	153,0	0,60	0,237
EMG1	175,6	198,8	189,9	248,7	1,08	0,931
EMI1	144,4	65,5	267,4	172,9	1,85	0,232
EMI2	117,8	29,9	325,5	124,0	2,76	0,017
EMI5	40,7	25,0	92,6	61,5	2,28	0,169
EMP24	462,6	262,6	280,2	218,4	0,61	0,327
EMP46	20,2	9,9	17,8	3,7	0,88	0,665
EMP47	160,3	69,1	166,8	26,5	1,04	0,866
EMP70	195,7	78,3	124,1	59,6	0,63	0,196
EMW1	123,9	28,0	43,7	15,1	0,35	0,002
ENA2	52,3	52,1	14,7	9,6	0,28	0,206
ENA5	17,7	14,4	3,3	3,0	0,19	0,097
ENB1	63,3	66,5	63,7	37,7	1,01	0,991
END3	314,4	245,6	465,2	303,5	1,48	0,469
ENO2	7041,6	1026,5	9540,8	1063,8	1,35	0,015
ENP1	88,3	46,5	101,9	50,3	1,15	0,705
ENP2	78,0	23,2	80,3	25,4	1,03	0,894
ENT1	31,2	26,3	26,0	24,0	0,83	0,780
ENT2	96,6	43,3	90,7	17,6	0,94	0,809
ENT3	42,0	31,1	76,7	61,5	1,82	0,353
ENT4	28,5	15,1	19,5	13,4	0,68	0,404
ENT5	35,9	27,2	30,7	22,7	0,86	0,782
EOS1	71,8	49,1	55,2	27,3	0,77	0,578
EPL1	35,3	23,5	36,7	24,7	1,04	0,939
EPS1	112,6	30,4	92,9	22,2	0,83	0,336
EPT1	22,1	19,9	14,1	10,7	0,64	0,506
ERB1	105,8	28,8	94,4	27,3	0,89	0,585
ERC1	1,0	0,6	0,7	0,9	0,66	0,538
ERD1	73,1	36,1	55,9	33,0	0,76	0,507
ERD2	197,4	96,0	85,5	38,7	0,43	0,074
ERF2	29,9	16,1	24,6	6,3	0,82	0,561
ERG1	258,4	177,8	250,0	186,0	0,97	0,950
ERG10	244,5	98,3	114,7	21,1	0,47	0,042
ERG11	346,6	316,5	158,7	118,9	0,46	0,309
ERG12	122,5	69,7	78,1	30,3	0,64	0,288
ERG13	191,8	73,3	127,9	71,3	0,67	0,258
ERG2	647,6	368,2	322,4	126,4	0,50	0,146
ERG20	565,5	294,3	576,4	188,3	1,02	0,952

ERG24	442,8	51,6	361,6	66,9	0,82	0,103
ERG25	1561,7	1297,9	511,5	282,4	0,33	0,165
ERG26	223,1	151,6	193,1	75,6	0,87	0,735
ERG27	237,2	158,7	149,9	114,9	0,63	0,408
ERG28	71,5	35,0	131,7	133,8	1,84	0,417
ERG3	1472,6	239,5	596,4	107,4	0,40	0,001
ERG4	245,3	95,7	80,4	3,4	0,33	0,014
ERG5	65,7	38,8	69,0	42,4	1,05	0,912
ERG6	278,2	146,3	128,9	87,1	0,46	0,130
ERG7	84,4	36,4	58,7	28,8	0,70	0,311
ERG8	269,1	93,0	143,1	29,8	0,53	0,042
ERG9	200,9	51,7	256,9	72,2	1,28	0,254
ERI1	251,4	190,7	168,9	102,4	0,67	0,475
ERJ5	136,0	52,1	60,4	42,4	0,44	0,065
ERO1	31,6	15,5	60,2	26,4	1,90	0,111
ERP1	187,7	88,8	108,9	62,2	0,58	0,196
ERP2	252,9	136,2	82,1	47,2	0,32	0,056
ERP3	134,5	109,3	10,2	8,6	0,08	0,064
ERP4	219,2	39,4	131,6	77,5	0,60	0,090
ERP5	211,4	56,9	109,1	27,6	0,52	0,018
ERP6	124,7	56,3	176,1	62,2	1,41	0,266
ERR1	0,0	0,0	0,0	0,0	#DIV/0!	#DIV/0!
ERR3	0,4	0,6	0,6	0,4	1,58	0,599
ERS1	62,4	43,2	12,1	10,4	0,19	0,064
ERT1	27,0	4,7	23,9	4,1	0,88	0,349
ERV1	87,7	40,1	57,4	33,8	0,65	0,291
ERV14	314,2	138,3	293,7	28,2	0,93	0,781
ERV2	100,7	57,1	37,8	19,7	0,38	0,083
ERV25	406,0	241,8	302,7	184,7	0,75	0,523
ERV29	117,4	38,7	138,4	56,5	1,18	0,564
ERV41	177,4	48,1	156,6	108,2	0,88	0,737
ERV46	64,4	7,7	53,2	15,5	0,83	0,245
ESA1	22,3	9,8	30,5	5,2	1,37	0,190
ESBP6	50,8	5,6	54,7	15,1	1,08	0,641
ESC1	35,9	11,6	62,6	14,3	1,75	0,027
ESC2	2,4	1,2	11,4	4,6	4,84	0,009
ESC8	41,3	12,0	19,4	2,3	0,47	0,011
ESF1	421,9	406,2	406,7	288,0	0,96	0,954
ESF2	31,5	15,3	26,9	7,6	0,85	0,609
ESP1	61,4	36,7	21,4	10,4	0,35	0,081
ESS1	32,2	17,5	54,0	51,5	1,68	0,454
EST1	23,4	7,9	4,9	3,7	0,21	0,005
EST2	20,1	8,5	17,9	2,4	0,89	0,636
EST3	75,0	52,7	136,7	59,9	1,82	0,173
ETP1	24,0	16,9	58,1	44,4	2,42	0,201
ETR1	53,0	35,4	61,0	44,7	1,15	0,789
ETT1	197,4	99,1	278,9	88,4	1,41	0,266
EUG1	134,2	72,2	74,9	23,3	0,56	0,169
EXG1	192,7	137,4	137,5	37,0	0,71	0,467
EXG2	79,7	52,6	68,5	30,9	0,86	0,726

EXO1	41,7	23,6	10,0	9,1	0,24	0,046
EXO5	15,9	7,0	13,3	9,2	0,84	0,666
EXO70	36,9	14,2	15,5	3,7	0,42	0,027
EXO84	43,1	28,3	49,0	24,4	1,14	0,763
FAA1	137,0	44,3	191,8	87,5	1,40	0,306
FAA2	9,7	3,6	11,9	2,3	1,22	0,359
FAA3	149,0	94,4	193,0	115,8	1,30	0,577
FAA4	348,4	230,6	42,5	11,5	0,12	0,038
FAB1	22,5	5,4	48,4	61,5	2,15	0,433
FAD1	83,4	54,8	84,1	53,0	1,01	0,985
FAF1	36,8	18,7	36,9	9,1	1,00	0,992
FAL1	32,5	35,5	21,2	23,6	0,65	0,616
FAP1	38,3	8,1	51,6	26,8	1,35	0,379
FAP7	77,2	13,6	25,2	17,7	0,33	0,003
FAR1	42,9	8,2	73,8	51,6	1,72	0,281
FAR10	96,0	13,7	120,5	21,2	1,25	0,101
FAR11	30,6	19,8	37,3	13,7	1,22	0,599
FAR3	9,1	6,2	22,3	15,1	2,46	0,156
FAR7	13,1	4,1	34,6	8,5	2,64	0,004
FAR8	60,0	13,1	32,5	15,2	0,54	0,034
FAS1	915,1	769,7	183,2	159,3	0,20	0,112
FAS2	602,3	353,2	365,5	281,9	0,61	0,335
FAT1	41,9	14,1	45,8	7,7	1,09	0,652
FAU1	79,8	24,5	92,4	18,5	1,16	0,444
FBA1	6403,3	3383,2	9306,7	3004,4	1,45	0,247
FBP1	1,0	0,8	1,4	1,4	1,44	0,624
FBP26	25,8	10,8	17,0	18,5	0,66	0,444
FCF1	38,1	6,6	5,1	3,8	0,13	0,000
FCF2	68,9	54,4	77,7	4,5	1,13	0,759
FCJ1	49,5	41,7	88,2	74,8	1,78	0,402
FCP1	43,6	25,6	33,4	28,6	0,77	0,614
FCY1	362,7	128,3	451,4	169,0	1,24	0,435
FCY2	1868,6	1367,5	789,9	521,1	0,42	0,191
FCY21	1,2	1,0	2,1	1,7	1,74	0,401
FCY22	1,2	0,3	2,6	2,0	2,15	0,210
FDC1	61,8	18,9	77,2	9,1	1,25	0,195
FDH1	0,5	0,4	0,2	0,4	0,43	0,348
FDH2	0,2	0,4	0,1	0,2	0,51	0,678
FEN1	867,8	430,9	203,0	115,5	0,23	0,025
FEN2	25,2	7,3	63,5	6,4	2,52	0,000
FES1	58,5	22,8	201,4	39,8	3,44	0,001
FET3	943,0	596,3	330,0	145,7	0,35	0,093
FET4	77,7	10,1	143,7	26,9	1,85	0,004
FET5	36,8	16,5	26,7	5,4	0,73	0,288
FHL1	11,4	4,2	16,3	17,8	1,43	0,612
FHN1	23,3	16,7	6,5	5,6	0,28	0,105
FIG1	2,7	0,7	13,5	9,2	4,99	0,059
FIG2	40,7	21,5	148,7	43,3	3,65	0,004
FIG4	14,1	3,4	20,9	16,1	1,49	0,438
FIN1	6,2	1,6	17,9	13,4	2,91	0,132

FIP1	69,7	28,4	90,9	34,0	1,30	0,374
FIR1	48,8	26,2	43,8	17,7	0,90	0,760
FIS1	27,8	33,7	35,7	48,0	1,28	0,796
FIT1	3,3	1,2	1,4	0,9	0,43	0,051
FIT3	50,6	29,6	54,8	24,7	1,08	0,834
FKH1	30,2	7,9	3,7	2,6	0,12	0,001
FKH2	25,8	6,2	25,3	16,9	0,98	0,955
FKS1	531,5	304,1	204,8	88,1	0,39	0,085
FKS3	5,9	1,4	10,6	10,6	1,80	0,412
FLC1	193,7	94,7	198,3	76,7	1,02	0,942
FLC2	73,7	31,7	42,1	30,9	0,57	0,203
FLC3	28,0	25,0	21,6	8,3	0,77	0,645
FLD1	36,4	11,0	16,3	7,1	0,45	0,022
FLO1	0,6	0,3	1,1	1,1	1,88	0,410
FLO10	1,9	0,6	0,9	0,6	0,49	0,074
FLO5	0,2	0,2	0,0	0,0	0,00	0,144
FLO8	3,3	1,3	4,9	1,1	1,50	0,105
FLO9	51,3	22,3	38,3	27,1	0,75	0,486
FLP1	118,8	66,1	42,4	20,4	0,36	0,069
FLR1	28,8	17,1	17,6	8,4	0,61	0,283
FLX1	16,8	9,3	13,9	5,2	0,82	0,599
FMC1	32,2	6,2	44,7	19,2	1,39	0,260
FMN1	26,0	15,4	43,4	24,6	1,67	0,275
FMO1	40,6	14,9	40,6	18,7	1,00	0,999
FMP10	82,2	40,5	191,5	133,4	2,33	0,168
FMP16	12,9	4,0	53,5	23,7	4,15	0,015
FMP21	51,9	31,7	10,1	8,7	0,19	0,044
FMP23	42,1	15,4	57,1	8,0	1,35	0,137
FMP25	70,1	29,5	47,9	8,9	0,68	0,199
FMP27	41,2	17,9	48,9	11,9	1,19	0,498
FMP30	26,7	10,6	27,9	17,5	1,04	0,911
FMP32	89,0	26,3	62,4	20,8	0,70	0,163
FMP33	9,7	3,5	19,6	5,4	2,01	0,023
FMP37	139,6	53,0	141,5	43,9	1,01	0,958
FMP40	29,2	39,7	44,9	67,5	1,54	0,703
FMP41	240,8	63,3	397,2	196,2	1,65	0,180
FMP43	29,7	23,5	4,9	5,1	0,16	0,085
FMP45	12,9	1,8	54,1	35,3	4,18	0,059
FMP46	17,0	11,6	38,2	31,0	2,25	0,246
FMP48	9,7	4,8	57,7	40,3	5,97	0,056
FMP52	198,2	53,6	198,9	69,2	1,00	0,988
FMS1	26,0	14,4	53,1	43,5	2,04	0,282
FMT1	0,4	0,6	0,5	0,6	1,17	0,868
FOB1	25,4	11,1	22,2	5,5	0,87	0,623
FOL1	69,8	28,7	49,0	8,2	0,70	0,212
FOL2	191,3	60,7	341,3	101,8	1,78	0,045
FOL3	23,9	9,8	35,2	21,6	1,47	0,376
FOX2	36,0	10,9	69,3	27,8	1,92	0,068
FPK1	15,2	0,9	18,3	12,3	1,21	0,627
FPR2	222,6	103,3	232,2	74,0	1,04	0,884

FPR3	543,7	213,8	903,1	367,9	1,66	0,142
FPR4	164,2	62,0	248,9	53,1	1,52	0,083
FPS1	105,4	63,3	85,1	46,6	0,81	0,625
FRA1	79,9	18,0	87,4	53,2	1,09	0,798
FRA2	92,9	34,2	152,4	39,2	1,64	0,063
FRE1	12,8	8,1	21,2	7,0	1,66	0,167
FRE2	2,5	1,5	2,6	2,5	1,04	0,947
FRE3	3,8	2,6	8,8	4,8	2,31	0,115
FRE4	2,6	1,2	2,6	2,0	1,00	0,995
FRE5	0,1	0,1	0,0	0,0	0,00	0,356
FRE6	23,2	4,2	45,4	14,3	1,95	0,025
FRE7	1,4	1,1	1,0	0,9	0,72	0,596
FRE8	1,6	1,6	4,5	3,2	2,75	0,161
FRK1	15,9	4,8	13,9	9,6	0,87	0,719
FRM2	2,4	2,5	11,7	9,6	4,93	0,109
FRQ1	108,5	13,3	105,3	28,5	0,97	0,846
FRS1	239,0	159,0	315,1	128,6	1,32	0,485
FRS2	253,8	118,5	210,7	59,7	0,83	0,541
FRT1	9,9	5,8	4,0	3,3	0,40	0,128
FRT2	15,7	5,9	38,1	15,9	2,42	0,039
FSF1	180,1	135,4	158,2	32,5	0,88	0,764
FSH1	128,2	78,6	138,2	103,6	1,08	0,883
FSH2	43,8	5,8	46,2	28,8	1,05	0,876
FSH3	43,9	16,5	26,7	3,5	0,61	0,088
FTH1	38,9	17,1	34,1	5,2	0,87	0,605
FTR1	222,5	113,2	36,8	34,0	0,17	0,020
FUI1	159,8	87,9	130,5	75,4	0,82	0,631
FUM1	160,5	64,1	229,8	78,3	1,43	0,219
FUN12	1485,5	936,5	2465,1	1419,6	1,66	0,293
FUN14	75,7	50,7	121,1	69,1	1,60	0,329
FUN19	19,9	10,7	72,0	50,0	3,62	0,088
FUN26	30,9	7,4	16,1	6,1	0,52	0,021
FUN30	164,0	118,0	146,8	88,3	0,89	0,823
FUR1	467,1	185,3	254,1	32,5	0,54	0,064
FUR4	89,8	70,9	238,1	183,2	2,65	0,182
FUS1	21,8	13,1	107,4	31,7	4,93	0,002
FUS2	9,6	4,7	18,3	13,9	1,91	0,280
FUS3	49,3	22,1	180,4	107,6	3,66	0,054
FYV1	0,5	0,8	0,0	0,1	0,06	0,321
FYV10	40,9	18,4	61,9	34,9	1,51	0,329
FYV12	0,0	0,0	0,0	0,1	#DIV/0!	0,356
FYV4	38,1	11,2	75,7	44,2	1,99	0,150
FYV5	1,2	0,6	0,8	0,7	0,67	0,437
FYV7	2,8	3,8	1,5	1,6	0,55	0,555
FYV8	24,4	7,9	16,2	11,2	0,66	0,275
FZF1	2,3	0,9	25,6	29,3	11,36	0,162
FZO1	13,3	3,2	15,7	1,2	1,18	0,206
GAA1	138,9	56,9	75,8	37,8	0,55	0,115
GAB1	45,3	28,8	42,3	29,4	0,93	0,886
GAC1	2,6	0,9	2,7	3,2	1,04	0,955

GAD1	61,1	10,7	324,8	203,6	5,31	0,041
GAL1	12,1	4,9	11,2	11,5	0,92	0,880
GAL10	0,3	0,4	0,2	0,5	0,96	0,977
GAL11	9,9	4,5	10,6	2,2	1,07	0,790
GAL2	3,4	1,7	3,3	2,3	0,98	0,955
GAL3	6,6	2,5	2,0	1,8	0,30	0,024
GAL4	3,9	3,0	8,7	6,3	2,25	0,211
GAL7	4,4	3,1	8,8	6,6	1,97	0,280
GAL80	52,9	28,2	41,4	12,0	0,78	0,483
GAL83	29,1	18,3	26,1	12,6	0,89	0,792
GAP1	145,6	17,7	15,1	6,3	0,10	0,000
GAR1	549,8	201,3	454,3	200,7	0,83	0,527
GAS1	493,2	219,7	140,3	74,2	0,28	0,023
GAS2	10,2	4,7	1,9	1,3	0,18	0,015
GAS3	205,1	89,0	38,0	27,6	0,19	0,012
GAS4	2,0	2,4	5,8	5,9	2,83	0,287
GAS5	203,5	192,3	129,4	105,2	0,64	0,524
GAT1	159,3	48,2	46,9	26,5	0,29	0,006
GAT2	18,3	2,6	24,7	11,8	1,35	0,333
GAT4	0,9	0,6	0,1	0,2	0,10	0,060
GBP2	231,2	81,7	142,4	54,3	0,62	0,120
GCD1	364,7	157,5	206,1	56,7	0,57	0,107
GCD10	35,7	19,8	31,9	27,3	0,89	0,830
GCD11	21,1	12,5	32,6	13,5	1,54	0,260
GCD14	36,0	6,7	35,5	5,0	0,99	0,911
GCD2	81,0	26,0	75,8	20,5	0,93	0,761
GCD6	312,5	118,3	284,2	80,7	0,91	0,706
GCD7	76,7	50,0	130,9	76,5	1,71	0,280
GCN1	146,0	81,1	133,2	62,2	0,91	0,811
GCN2	30,9	10,3	20,1	13,8	0,65	0,256
GCN20	64,3	26,4	57,4	16,8	0,89	0,676
GCN3	166,7	54,0	156,4	52,1	0,94	0,793
GCN4	386,6	114,8	369,0	95,2	0,95	0,821
GCN5	23,5	13,9	48,9	60,5	2,08	0,445
GCR1	14,8	4,8	28,1	4,4	1,90	0,006
GCR2	63,9	24,9	83,6	22,3	1,31	0,285
GCS1	129,3	59,1	40,9	13,0	0,32	0,027
GCV1	321,4	222,4	155,4	137,7	0,48	0,251
GCV2	447,0	127,7	95,5	46,7	0,21	0,002
GCV3	975,9	701,2	436,3	263,0	0,45	0,200
GCY1	28,1	4,2	150,2	84,0	5,35	0,027
GDA1	295,2	120,8	175,9	77,8	0,60	0,148
GDB1	44,6	30,1	63,1	61,9	1,41	0,611
GDE1	100,3	68,0	108,2	70,8	1,08	0,876
GDH1	1071,8	254,5	227,3	70,3	0,21	0,001
GDH2	20,3	5,3	72,9	40,1	3,60	0,041
GDH3	14,4	6,3	24,4	13,8	1,70	0,231
GDI1	120,6	54,8	189,4	159,8	1,57	0,447
GDS1	84,9	20,5	48,1	5,4	0,57	0,013
GDT1	126,0	146,8	30,7	25,8	0,24	0,248

GEA1	23,2	15,6	15,3	4,4	0,66	0,363
GEA2	67,9	16,7	48,5	8,2	0,71	0,083
GEF1	31,5	13,5	16,8	11,2	0,53	0,146
GEM1	0,8	0,8	2,6	1,7	3,25	0,109
GEP3	111,9	17,1	215,0	54,2	1,92	0,011
GEP4	28,2	16,4	15,2	11,1	0,54	0,237
GEP5	7,2	4,5	3,3	2,8	0,46	0,190
GEP7	19,4	2,8	23,4	21,0	1,21	0,719
GET2	163,7	54,7	159,7	50,7	0,98	0,918
GET3	286,4	51,0	237,8	62,8	0,83	0,275
GET4	141,3	58,3	135,8	50,6	0,96	0,891
GFA1	214,0	61,7	205,3	117,4	0,96	0,899
GFD1	119,2	65,8	207,8	112,8	1,74	0,224
GFD2	39,7	14,5	73,1	27,3	1,84	0,074
GGA1	9,8	5,7	16,5	11,2	1,68	0,330
GGA2	85,8	41,6	60,4	8,5	0,70	0,277
GGC1	305,7	170,7	315,3	124,6	1,03	0,930
GIC1	23,2	10,8	6,6	6,1	0,28	0,037
GIC2	185,3	49,1	161,0	33,5	0,87	0,446
GID7	43,7	18,9	69,2	27,9	1,58	0,182
GID8	102,5	45,2	104,8	21,6	1,02	0,929
GIM3	171,1	64,9	158,5	29,0	0,93	0,735
GIM4	199,0	24,5	117,1	48,6	0,59	0,024
GIM5	36,0	13,2	24,2	6,9	0,67	0,165
GIN4	168,7	34,6	20,5	5,0	0,12	0,000
GIP1	3,2	1,8	16,2	23,5	5,08	0,311
GIP2	18,1	4,9	16,9	12,1	0,94	0,868
GIP3	30,1	23,2	13,1	10,0	0,44	0,227
GIP4	25,6	6,3	31,3	9,9	1,22	0,374
GIR2	124,8	29,7	190,4	72,1	1,53	0,143
GIS1	203,5	148,8	274,3	221,3	1,35	0,614
GIS2	720,4	432,4	1092,2	727,9	1,52	0,414
GIS3	39,6	52,6	69,0	86,3	1,74	0,581
GIS4	22,3	9,0	21,2	9,6	0,95	0,877
GIT1	3,7	2,1	3,6	2,4	0,98	0,967
GLC3	39,4	12,3	153,8	20,4	3,91	0,000
GLC7	150,3	29,3	163,4	80,6	1,09	0,771
GLC8	175,3	19,2	411,5	32,3	2,35	0,000
GLE1	39,7	21,8	31,9	11,2	0,81	0,552
GLE2	61,3	61,9	32,7	23,8	0,53	0,422
GLG1	17,6	9,1	19,1	5,9	1,09	0,788
GLG2	14,3	4,5	5,0	3,4	0,35	0,016
GLK1	367,7	114,9	827,6	352,9	2,25	0,048
GLN1	4162,8	2340,7	1415,9	645,0	0,34	0,064
GLN3	13,9	11,2	10,4	9,5	0,75	0,658
GLN4	174,4	62,0	187,6	93,1	1,08	0,821
GLO1	75,0	21,9	173,2	76,5	2,31	0,049
GLO2	145,8	96,3	297,7	256,9	2,04	0,311
GLO3	154,8	25,6	120,1	63,1	0,78	0,347
GLO4	32,0	18,9	84,7	44,5	2,65	0,072

GLR1	184,3	13,9	210,3	92,5	1,14	0,599
GLT1	106,2	66,4	29,4	15,0	0,28	0,065
GLY1	98,5	54,3	149,5	74,5	1,52	0,311
GNA1	126,6	37,9	77,1	13,8	0,61	0,049
GND1	1926,1	1231,7	1429,3	591,0	0,74	0,494
GND2	6,8	2,1	17,2	12,0	2,53	0,139
GNP1	1126,2	337,2	206,1	100,8	0,18	0,002
GNT1	131,2	80,4	42,8	22,6	0,33	0,079
GON7	79,8	45,1	58,5	26,3	0,73	0,447
GOR1	43,7	17,7	156,8	68,3	3,58	0,019
GOS1	60,8	29,7	70,2	28,5	1,16	0,661
GOT1	60,7	17,4	7,9	5,5	0,13	0,001
GPA1	112,6	35,4	203,2	102,1	1,80	0,145
GPA2	84,7	9,9	107,9	21,1	1,27	0,094
GPB1	22,9	6,6	26,2	17,5	1,14	0,738
GPB2	77,5	24,2	78,3	15,7	1,01	0,959
GPD1	240,3	133,8	420,3	375,3	1,75	0,401
GPD2	337,8	183,9	387,5	102,8	1,15	0,654
GPH1	16,6	12,6	55,5	35,1	3,34	0,082
GPI1	28,1	12,4	9,8	5,0	0,35	0,034
GPI10	50,1	17,2	30,9	14,7	0,62	0,141
GPI11	43,4	16,9	31,4	16,2	0,72	0,345
GPI12	43,8	32,8	42,3	34,0	0,97	0,951
GPI13	219,9	53,6	154,3	32,5	0,70	0,081
GPI14	81,1	39,2	56,0	15,3	0,69	0,276
GPI15	79,6	46,7	28,9	25,1	0,36	0,105
GPI16	74,8	26,7	70,3	38,9	0,94	0,856
GPI17	169,4	27,0	146,8	29,6	0,87	0,304
GPI18	79,5	38,3	67,7	19,3	0,85	0,603
GPI19	0,3	0,3	0,1	0,2	0,43	0,471
GPI2	6,7	4,7	16,6	11,5	2,48	0,162
GPI8	69,1	8,1	33,1	15,7	0,48	0,006
GPM1	3719,4	2121,5	4814,8	1831,0	1,29	0,464
GPM2	49,0	8,4	45,4	25,1	0,93	0,795
GPM3	30,3	21,1	43,3	33,7	1,43	0,535
GPR1	78,2	39,5	160,9	60,0	2,06	0,061
GPT2	68,4	43,6	86,0	62,1	1,26	0,658
GPX1	7,9	3,9	43,9	29,0	5,58	0,049
GPX2	310,8	195,0	181,5	47,6	0,58	0,245
GRC3	57,0	22,5	71,7	33,0	1,26	0,489
GRE1	5,6	2,8	9,9	7,3	1,77	0,316
GRE2	57,7	21,1	103,6	54,9	1,79	0,170
GRE3	365,1	45,3	1330,6	607,7	3,64	0,019
GRH1	69,5	30,5	74,7	23,6	1,07	0,798
GRR1	22,4	7,7	28,5	3,2	1,27	0,193
GRS1	789,9	279,3	561,7	202,3	0,71	0,234
GRS2	12,6	9,8	19,0	9,8	1,51	0,392
GRX1	766,0	310,4	700,6	274,3	0,91	0,763
GRX3	251,0	37,1	240,7	48,0	0,96	0,746
GRX4	114,4	35,7	52,6	18,8	0,46	0,022

GRX5	262,1	112,3	375,2	78,5	1,43	0,150
GRX6	63,8	26,9	38,7	7,9	0,61	0,122
GRX7	96,3	30,8	69,7	30,4	0,72	0,266
GSC2	67,2	30,5	44,0	29,1	0,65	0,314
GSF2	147,4	67,8	157,8	88,9	1,07	0,858
GSG1	40,4	20,8	49,8	30,0	1,23	0,624
GSH1	21,0	8,9	38,7	19,0	1,84	0,143
GSH2	209,0	55,4	138,0	15,2	0,66	0,048
GSM1	5,1	2,5	13,8	9,5	2,68	0,128
GSP1	585,1	223,3	365,4	112,1	0,62	0,129
GSP2	48,6	31,4	70,3	52,5	1,45	0,505
GSY1	21,5	3,8	47,8	15,7	2,22	0,017
GSY2	94,7	26,1	231,7	139,0	2,45	0,101
GTB1	15,9	10,5	50,4	23,5	3,16	0,036
GTO1	14,6	5,5	24,5	18,2	1,68	0,339
GTO3	0,2	0,4	0,3	0,2	1,64	0,596
GTR1	46,7	16,5	34,9	23,4	0,75	0,441
GTR2	59,8	31,8	56,1	52,9	0,94	0,908
GTS1	36,7	15,3	40,9	22,9	1,12	0,768
GTT2	8,5	2,7	14,7	4,9	1,72	0,070
GTT3	50,3	23,0	21,3	17,4	0,42	0,091
GUA1	729,9	488,7	819,2	466,8	1,12	0,800
GUD1	5,9	3,9	35,3	26,0	5,99	0,067
GUF1	9,8	1,9	19,8	24,7	2,02	0,450
GUK1	342,5	134,4	300,5	65,9	0,88	0,594
GUP1	114,7	90,6	108,9	97,0	0,95	0,933
GUP2	6,2	3,5	2,6	1,9	0,42	0,119
GUS1	394,1	118,4	314,2	108,2	0,80	0,357
GUT1	47,2	25,1	30,1	10,4	0,64	0,253
GUT2	47,0	27,7	28,9	20,7	0,61	0,336
GVP36	247,2	29,7	361,1	73,1	1,46	0,028
GWT1	130,0	85,6	64,7	49,0	0,50	0,234
GYL1	62,1	28,1	38,7	25,9	0,62	0,266
GYP1	34,7	5,9	38,5	11,5	1,11	0,578
GYP5	9,8	7,1	19,3	17,8	1,98	0,356
GYP6	121,3	24,1	125,4	14,3	1,03	0,775
GYP7	19,7	5,5	24,8	8,8	1,26	0,371
GYP8	99,1	46,9	231,6	90,3	2,34	0,040
GZF3	73,9	19,0	62,8	23,1	0,85	0,487
HAA1	58,7	8,8	81,4	29,6	1,39	0,193
HAC1	386,9	302,4	347,4	226,1	0,90	0,841
HAL1	5,7	1,6	17,8	9,0	3,15	0,037
HAL5	85,9	16,8	122,9	30,2	1,43	0,076
HAL9	38,6	19,0	36,0	29,8	0,93	0,890
HAM1	69,8	96,5	35,6	25,7	0,51	0,519
HAP1	83,3	44,6	67,5	10,4	0,81	0,516
HAP2	37,4	17,8	64,7	23,4	1,73	0,113
HAP3	30,8	30,1	26,6	20,1	0,86	0,825
HAP4	70,8	63,7	118,0	74,6	1,67	0,373
HAP5	17,5	7,7	21,3	15,8	1,22	0,680

HAS1	88,5	61,2	158,1	50,1	1,79	0,129
HAT2	37,8	43,5	80,0	128,5	2,12	0,557
HBS1	45,0	19,3	41,1	15,8	0,91	0,765
HBT1	13,4	8,7	10,1	6,8	0,75	0,572
HCA4	107,7	73,2	100,8	47,4	0,94	0,880
HCH1	306,3	337,5	506,4	594,6	1,65	0,580
HCM1	17,6	10,5	7,1	5,5	0,40	0,127
HCR1	180,8	64,5	144,5	40,1	0,80	0,376
HCS1	19,7	6,8	13,2	9,2	0,67	0,299
HDA1	22,1	7,3	34,5	23,9	1,56	0,359
HDA2	26,6	7,0	37,7	9,5	1,42	0,111
HDA3	15,0	11,6	12,8	6,2	0,85	0,752
HEF3	1,6	1,2	0,7	0,6	0,42	0,235
HEH2	44,8	9,3	49,5	9,8	1,10	0,518
HEK2	423,2	104,9	211,1	49,4	0,50	0,011
HEM1	70,0	23,8	151,5	66,7	2,16	0,061
HEM12	81,0	32,3	66,9	34,2	0,83	0,570
HEM13	75,6	55,8	41,5	28,1	0,55	0,318
HEM14	22,2	21,9	11,1	5,9	0,50	0,366
HEM15	75,0	32,1	81,2	27,9	1,08	0,779
HEM2	200,2	45,0	203,9	7,8	1,02	0,876
HEM3	9,0	7,0	8,7	7,1	0,97	0,958
HEM4	37,6	11,5	40,2	39,9	1,07	0,905
HER1	28,1	16,3	13,5	9,5	0,48	0,171
HER2	93,5	56,0	35,9	15,8	0,38	0,095
HES1	1,6	0,5	0,6	0,6	0,40	0,043
HFA1	81,7	34,5	60,2	26,2	0,74	0,358
HFD1	17,2	11,2	27,6	10,7	1,60	0,230
HFI1	28,5	15,0	37,9	34,5	1,33	0,635
HFM1	2,1	2,6	7,6	7,6	3,70	0,215
HGH1	67,8	16,6	63,5	29,4	0,94	0,806
HHF2	2667,4	2240,3	311,4	220,6	0,12	0,081
HHO1	152,1	46,9	27,0	12,6	0,18	0,002
HHT2	316,6	138,1	168,9	54,3	0,53	0,094
HHY1	0,0	0,0	0,5	0,5	#DIV/0!	0,075
HIF1	42,0	22,5	8,7	7,1	0,21	0,030
HIM1	4,9	2,0	8,2	5,0	1,70	0,255
HIP1	49,3	42,0	60,5	48,4	1,23	0,738
HIR1	19,7	6,4	18,0	4,5	0,91	0,678
HIR2	42,1	8,9	21,9	5,2	0,52	0,008
HIR3	36,9	21,2	34,7	16,4	0,94	0,876
HIS1	590,0	310,5	332,9	92,9	0,56	0,164
HIS2	306,5	111,8	264,3	108,1	0,86	0,607
HIS3	19,3	9,9	45,5	39,2	2,36	0,243
HIS4	316,6	139,0	167,4	97,5	0,53	0,129
HIS5	323,1	122,2	251,0	95,4	0,78	0,388
HIS6	22,2	17,7	34,7	26,0	1,57	0,456
HIS7	108,1	53,8	118,8	57,2	1,10	0,794
HIT1	3,5	2,8	7,3	4,3	2,07	0,195
HKR1	26,9	11,1	28,8	16,7	1,07	0,859

HLJ1	30,5	23,2	50,4	24,6	1,65	0,285
HLR1	54,2	25,5	11,3	12,3	0,21	0,023
HMF1	328,8	170,1	578,1	186,6	1,76	0,096
HMG1	136,4	37,4	54,7	14,7	0,40	0,007
HMG2	62,9	22,7	23,1	15,5	0,37	0,027
HMI1	11,1	1,8	7,1	5,1	0,64	0,194
HMLALPHA1	0,0	0,0	0,0	0,0	#DIV/0!	#DIV/0!
HMO1	467,7	130,5	571,4	111,0	1,22	0,271
HMS1	1,8	0,9	1,9	1,5	1,07	0,894
HMS2	32,8	10,4	28,8	8,3	0,88	0,567
HMT1	408,1	171,8	323,7	67,7	0,79	0,396
HMX1	36,8	17,2	54,5	9,6	1,48	0,123
HNM1	144,9	10,1	58,5	36,2	0,40	0,004
HNT1	975,2	132,6	253,0	79,4	0,26	0,000
HNT2	50,3	7,8	33,6	25,1	0,67	0,249
HNT3	78,1	15,3	60,3	14,3	0,77	0,140
HO	81,4	70,1	10,2	15,0	0,13	0,094
HOC1	203,1	169,3	192,5	139,8	0,95	0,926
HOF1	8,7	2,2	16,6	6,2	1,90	0,053
HOG1	262,6	145,0	171,5	118,0	0,65	0,367
HOL1	92,9	5,8	98,0	34,7	1,05	0,785
HOM2	407,9	158,5	707,0	451,3	1,73	0,258
HOM3	209,8	176,1	327,9	231,9	1,56	0,448
HOM6	426,2	199,8	536,6	238,7	1,26	0,505
HOP2	30,4	7,7	40,4	8,9	1,33	0,142
HOR2	24,3	16,4	27,4	14,6	1,13	0,790
HOS1	27,2	5,0	16,5	5,9	0,60	0,032
HOS2	3,1	1,1	7,3	4,8	2,33	0,147
HOS3	47,5	20,2	16,6	3,0	0,35	0,023
HOS4	119,7	36,8	214,6	49,6	1,79	0,022
HOT1	17,8	9,4	6,2	4,6	0,35	0,068
HOT13	133,4	42,0	121,8	73,7	0,91	0,794
HPA3	55,1	27,9	46,9	13,2	0,85	0,613
HPC2	22,8	5,0	31,8	4,7	1,39	0,040
HPF1	12,4	9,7	7,2	2,0	0,58	0,332
HPM1	93,1	65,5	82,9	38,9	0,89	0,797
HPR1	5,1	3,7	16,5	6,2	3,26	0,019
HPT1	882,7	324,0	108,0	42,6	0,12	0,003
HRB1	42,1	24,1	67,2	34,1	1,60	0,275
HRD1	60,6	31,7	48,2	24,1	0,80	0,557
HRD3	18,3	4,2	26,2	11,3	1,43	0,237
HRK1	32,1	12,5	90,0	42,5	2,80	0,040
HRP1	936,8	213,7	150,7	33,6	0,16	0,000
HRQ1	42,6	12,6	32,9	11,5	0,77	0,300
HRR25	113,5	56,8	132,0	53,3	1,16	0,652
HRT1	107,9	50,1	205,2	77,2	1,90	0,079
HRT3	33,4	6,9	31,7	8,3	0,95	0,764
HSC82	1950,6	1294,0	5213,9	3818,5	2,67	0,157
HSE1	43,0	11,9	81,0	27,3	1,89	0,043
HSF1	57,0	37,1	78,7	41,0	1,38	0,462

HSH155	22,9	9,3	42,5	12,3	1,85	0,044
HSH49	61,9	22,2	140,2	51,7	2,26	0,032
HSK3	50,7	31,7	67,6	56,6	1,33	0,622
HSL1	195,8	93,9	36,3	6,5	0,19	0,015
HSL7	119,1	36,8	44,7	3,4	0,38	0,007
HSM3	23,8	12,4	16,9	11,3	0,71	0,444
HSP104	106,9	49,6	718,3	287,2	6,72	0,006
HSP12	1264,7	547,9	973,2	422,0	0,77	0,432
HSP26	49,6	24,8	144,6	24,8	2,92	0,002
HSP30	12,5	8,2	247,6	129,0	19,74	0,011
HSP31	25,1	11,8	15,9	12,2	0,63	0,319
HSP42	56,9	25,3	330,6	94,8	5,81	0,001
HSP60	539,3	293,9	1174,7	465,7	2,18	0,060
HSP78	91,8	66,0	443,0	304,1	4,83	0,065
HSP82	174,9	51,8	1845,1	333,2	10,55	0,000
HST1	239,1	147,6	233,6	180,9	0,98	0,964
HST2	87,8	24,5	77,5	50,3	0,88	0,724
HST3	59,4	26,5	36,4	13,2	0,61	0,172
HST4	38,5	20,2	38,6	13,1	1,00	0,996
HSV2	39,4	22,3	37,1	18,9	0,94	0,878
HTA1	1248,2	392,2	73,6	30,6	0,06	0,001
HTB1	1924,8	516,1	304,5	154,3	0,16	0,001
HTB2	868,2	571,7	83,5	54,6	0,10	0,034
HTD2	30,3	5,1	12,3	4,3	0,41	0,002
HTS1	877,0	640,3	1509,4	1131,7	1,72	0,368
HTZ1	149,4	63,0	91,5	31,3	0,61	0,151
HUA1	38,6	13,8	154,5	54,7	4,01	0,006
HUB1	97,0	69,2	114,7	119,6	1,18	0,807
HUG1	10,8	2,4	20,5	17,3	1,91	0,305
HUL4	12,0	4,2	38,7	37,8	3,22	0,210
HUL5	29,9	16,2	65,6	32,0	2,20	0,093
HUR1	20,0	15,0	10,4	10,0	0,52	0,331
HUT1	36,5	13,5	24,4	2,9	0,67	0,130
HVG1	12,2	5,5	12,7	7,9	1,04	0,919
HXK1	201,5	70,3	1026,8	372,9	5,10	0,005
HXK2	1080,4	553,8	1762,7	833,6	1,63	0,222
HXT1	171,5	63,5	121,6	29,9	0,71	0,205
HXT10	0,9	1,9	0,2	0,2	0,20	0,455
HXT11	0,3	0,3	0,1	0,2	0,27	0,238
HXT12	2,6	2,1	3,6	3,3	1,41	0,607
HXT13	0,1	0,2	0,0	0,0	0,00	0,356
HXT14	0,0	0,0	0,2	0,2	#DIV/0!	0,205
HXT15	0,3	0,3	0,1	0,1	0,18	0,128
HXT17	0,6	0,5	0,4	0,5	0,60	0,539
HXT2	186,4	177,0	64,1	26,8	0,34	0,221
HXT3	142,2	67,0	287,0	119,2	2,02	0,079
HXT4	33,1	12,1	255,1	196,2	7,70	0,065
HXT5	10,8	9,3	5,4	3,7	0,50	0,320
HXT6	0,2	0,3	0,1	0,3	0,53	0,574
HXT7	432,1	127,7	625,9	336,9	1,45	0,323

HXT8	0,1	0,2	0,4	0,3	2,96	0,297
HXT9	0,6	0,4	0,8	0,7	1,44	0,568
HYM1	13,8	6,6	34,9	28,7	2,53	0,201
HYP2	1731,1	1769,9	1130,6	992,9	0,65	0,576
HYR1	362,4	365,8	438,8	418,3	1,21	0,793
IAH1	52,7	24,1	39,4	15,9	0,75	0,391
IBD2	52,5	23,5	30,9	8,3	0,59	0,133
ICE2	126,2	70,4	89,7	19,3	0,71	0,356
ICL1	39,2	15,1	59,2	37,6	1,51	0,361
ICL2	54,3	28,7	50,9	7,3	0,94	0,824
ICP55	32,7	10,6	39,2	10,6	1,20	0,420
ICS2	49,6	28,1	41,2	17,5	0,83	0,629
ICS3	4,6	2,7	2,3	1,6	0,49	0,187
ICT1	6,0	6,3	1,7	1,8	0,28	0,233
ICY1	123,8	83,6	209,7	81,5	1,69	0,192
ICY2	64,1	28,5	123,4	63,6	1,93	0,140
IDH1	922,7	876,2	407,0	317,9	0,44	0,311
IDH2	465,5	122,9	263,5	183,9	0,57	0,118
IDI1	86,0	36,2	80,9	7,9	0,94	0,793
IDP1	388,9	122,2	590,2	149,5	1,52	0,082
IDP2	8,3	2,9	4,8	3,6	0,58	0,186
IDP3	9,7	2,3	24,4	7,4	2,50	0,009
IDS2	70,7	22,1	34,2	14,3	0,48	0,032
IES1	65,6	15,2	84,5	3,4	1,29	0,051
IES3	63,9	16,0	62,9	50,4	0,98	0,971
IES5	30,5	9,9	186,3	65,6	6,10	0,003
IFA38	58,2	40,5	32,2	6,4	0,55	0,253
IFH1	74,3	60,9	117,3	87,9	1,58	0,452
IFM1	15,6	6,7	9,8	6,6	0,63	0,263
IGO1	110,5	34,8	164,4	47,4	1,49	0,117
IGO2	72,1	26,3	77,3	19,5	1,07	0,762
IKI1	24,7	6,7	27,0	12,3	1,10	0,747
IKI3	121,5	45,2	96,8	6,9	0,80	0,321
IKS1	33,5	20,0	92,8	23,9	2,77	0,009
ILM1	214,7	32,5	60,3	14,9	0,28	0,000
ILS1	364,6	209,1	517,5	224,7	1,42	0,358
ILV1	896,3	165,6	330,2	53,8	0,37	0,001
ILV2	692,2	215,5	1119,0	375,6	1,62	0,096
ILV3	681,6	357,9	495,6	142,7	0,73	0,372
ILV5	1466,1	1029,1	947,3	639,3	0,65	0,425
ILV6	479,4	72,8	413,6	204,2	0,86	0,566
IMA1	3,8	3,8	7,3	5,2	1,91	0,320
IMA2	21,7	8,2	41,7	11,5	1,93	0,030
IMA3	5,1	4,6	2,8	3,1	0,55	0,439
IMA5	7,3	4,1	1,4	1,2	0,19	0,033
IMD1	38,7	25,0	8,3	7,1	0,22	0,058
IMD2	1,4	1,7	0,0	0,0	0,00	0,155
IMD3	196,0	66,0	66,9	46,0	0,34	0,018
IMD4	174,7	154,4	94,5	59,0	0,54	0,369
IME1	2,2	0,7	1,8	1,2	0,82	0,588

IME2	6,5	1,4	4,0	2,9	0,61	0,163
IME4	3,2	2,0	2,7	2,9	0,84	0,776
IMG1	35,7	34,7	42,5	43,1	1,19	0,814
IMG2	161,5	60,1	68,9	21,8	0,43	0,027
IMH1	185,4	169,7	164,3	139,1	0,89	0,853
IML1	29,3	16,4	24,3	20,1	0,83	0,713
IML2	31,8	4,9	95,4	61,5	2,99	0,085
IML3	2,4	1,3	4,3	3,5	1,75	0,367
IMP1	86,2	18,6	70,0	14,1	0,81	0,215
IMP2	15,1	5,5	19,8	2,4	1,31	0,167
IMP2'	61,7	46,3	144,2	112,3	2,34	0,223
IMP3	30,8	14,4	35,3	15,7	1,15	0,685
IMP4	64,5	30,9	67,7	24,2	1,05	0,874
INH1	104,5	104,9	209,1	226,2	2,00	0,433
INM1	43,9	5,4	149,9	45,9	3,42	0,004
INM2	39,5	10,2	53,2	24,9	1,35	0,349
INN1	33,2	5,9	32,5	4,9	0,98	0,856
INO1	7,3	11,4	1,5	1,2	0,21	0,353
INO2	14,2	2,0	12,7	8,7	0,89	0,747
INO4	64,4	31,1	81,1	49,6	1,26	0,589
INO80	36,8	4,8	46,0	11,2	1,25	0,182
INP1	14,2	7,3	10,8	7,5	0,76	0,537
INP2	33,0	14,1	14,6	10,3	0,44	0,079
INP51	36,4	17,2	27,9	9,0	0,77	0,417
INP52	31,6	9,8	34,1	8,8	1,08	0,722
INP53	35,4	4,2	35,4	13,0	1,00	0,998
INP54	19,1	14,9	25,9	20,9	1,35	0,616
IOC2	98,8	67,4	108,1	51,4	1,09	0,833
IOC3	59,0	18,6	47,7	18,8	0,81	0,426
IOC4	62,3	28,9	59,2	28,6	0,95	0,886
IPI1	33,5	14,7	41,5	22,7	1,24	0,577
IPI3	57,1	65,8	37,7	32,5	0,66	0,615
IPK1	26,7	11,7	12,6	2,7	0,47	0,056
IPL1	19,4	9,0	6,0	5,0	0,31	0,041
IPP1	3199,1	1415,6	3802,1	1480,0	1,19	0,577
IPT1	58,7	8,5	23,3	16,3	0,40	0,008
IQG1	29,1	11,5	32,9	7,6	1,13	0,596
IRA1	75,9	56,5	82,2	66,0	1,08	0,891
IRA2	114,7	25,0	135,4	60,2	1,18	0,549
IRC10	7,1	1,5	2,3	1,8	0,32	0,006
IRC11	13,8	4,3	21,3	7,8	1,55	0,141
IRC14	8,2	9,0	12,7	5,2	1,55	0,419
IRC15	11,3	2,5	25,2	19,2	2,23	0,202
IRC16	1,3	1,3	0,3	0,3	0,27	0,232
IRC18	0,0	0,0	0,0	0,0	#DIV/0!	#DIV/0!
IRC19	14,0	5,0	9,9	5,2	0,71	0,300
IRC2	3,2	1,7	0,2	0,3	0,07	0,013
IRC20	34,4	9,0	37,8	21,2	1,10	0,776
IRC21	40,2	19,5	23,6	17,1	0,59	0,248
IRC22	132,4	34,2	105,5	21,5	0,80	0,231

IRC23	43,3	11,9	45,9	26,5	1,06	0,863
IRC24	40,2	27,0	52,5	37,3	1,31	0,613
IRC25	49,1	15,1	38,4	22,5	0,78	0,460
IRC3	12,5	3,2	32,1	33,2	2,57	0,285
IRC5	40,0	19,3	20,2	18,6	0,51	0,190
IRC6	18,2	6,6	19,2	7,1	1,06	0,840
IRC7	68,1	20,9	97,6	28,8	1,43	0,148
IRC8	8,8	2,7	9,4	6,9	1,07	0,876
IRC9	40,0	26,7	30,7	19,2	0,77	0,591
IRE1	52,1	31,3	45,6	23,8	0,87	0,752
IRR1	77,1	60,1	26,1	9,1	0,34	0,144
IRS4	19,2	13,4	12,3	9,1	0,64	0,427
ISA1	133,2	56,5	80,5	19,4	0,60	0,128
ISA2	144,8	64,0	212,6	84,5	1,47	0,248
ISC1	23,7	6,2	8,2	5,7	0,35	0,010
ISD11	7,1	6,9	4,1	4,3	0,58	0,491
ISF1	4,3	2,5	9,7	10,0	2,22	0,341
ISM1	21,3	8,1	19,9	13,3	0,93	0,855
ISN1	32,9	21,7	54,9	14,1	1,67	0,139
ISR1	31,6	12,5	16,5	8,8	0,52	0,096
IST1	28,8	15,8	46,6	25,8	1,62	0,285
IST2	125,0	58,9	81,1	33,1	0,65	0,241
IST3	21,7	12,8	18,1	8,3	0,83	0,649
ISU1	19,3	9,8	28,6	21,0	1,48	0,453
ISU2	13,8	12,8	7,1	3,0	0,51	0,349
ISW1	115,6	53,3	75,5	43,5	0,65	0,288
ISW2	60,2	40,1	51,2	21,3	0,85	0,705
ISY1	43,2	21,3	58,8	37,1	1,36	0,495
ITC1	69,4	44,3	79,2	46,8	1,14	0,770
ITR1	164,3	40,1	122,7	52,6	0,75	0,255
ITR2	141,6	41,5	89,9	46,1	0,63	0,146
ITT1	15,7	4,7	36,9	11,0	2,35	0,012
IVY1	47,0	24,5	60,1	24,1	1,28	0,473
IWR1	31,7	11,0	60,2	48,9	1,90	0,300
IXR1	47,9	21,7	28,1	19,0	0,59	0,219
IZH1	41,0	33,7	17,5	18,6	0,43	0,270
IZH2	64,4	37,5	54,6	10,6	0,85	0,632
IZH3	40,9	34,9	48,9	15,1	1,20	0,687
IZH4	7,0	10,6	1,1	1,2	0,16	0,311
JAC1	14,2	10,0	29,1	25,1	2,05	0,312
JEM1	69,7	23,8	23,5	6,1	0,34	0,009
JEN1	56,5	18,0	46,2	16,5	0,82	0,428
JHD1	22,7	15,5	27,1	22,7	1,19	0,761
JHD2	21,0	9,3	25,9	8,5	1,23	0,468
JID1	8,9	3,5	11,6	10,3	1,31	0,632
JIP3	1,8	1,4	1,3	1,6	0,74	0,683
JIP4	30,0	10,3	41,8	16,8	1,39	0,277
JIP5	261,6	59,7	354,7	79,1	1,36	0,109
JJJ1	160,7	73,8	161,4	84,1	1,00	0,990
JJJ2	73,0	59,8	41,1	22,7	0,56	0,357

JJJ3	45,8	16,6	34,1	22,8	0,75	0,440
JLP1	4,5	0,8	10,6	5,6	2,36	0,074
JLP2	12,9	9,4	11,5	9,9	0,89	0,844
JNM1	30,6	5,2	34,5	10,4	1,13	0,533
JSN1	6,8	5,4	6,4	4,9	0,94	0,920
KAE1	142,7	16,8	78,9	11,0	0,55	0,001
KAP104	66,5	17,7	91,7	9,2	1,38	0,045
KAP114	57,1	12,8	47,1	32,6	0,82	0,586
KAP120	41,6	20,9	22,0	3,9	0,53	0,114
KAP122	26,5	17,5	9,7	8,1	0,37	0,133
KAP123	109,5	45,3	82,4	24,5	0,75	0,332
KAP95	56,3	39,5	42,4	29,9	0,75	0,594
KAR1	29,3	6,5	13,7	9,2	0,47	0,033
KAR2	411,5	227,2	535,6	128,1	1,30	0,378
KAR3	45,3	9,8	12,9	10,1	0,28	0,004
KAR4	27,0	11,9	72,1	11,3	2,67	0,002
KAR5	20,6	7,1	52,1	24,1	2,53	0,046
KAR9	78,0	17,3	29,9	10,7	0,38	0,003
KCC4	42,0	14,0	2,1	1,4	0,05	0,001
KCS1	28,8	13,6	17,1	11,8	0,59	0,241
KDX1	4,3	2,6	2,1	2,1	0,49	0,239
KEG1	31,6	19,3	18,7	9,5	0,59	0,275
KEI1	68,1	30,2	17,0	14,2	0,25	0,022
KEL1	91,2	42,4	52,4	21,0	0,57	0,152
KEL2	61,3	57,6	38,6	23,6	0,63	0,493
KEL3	69,2	18,3	61,9	8,3	0,89	0,493
KEM1	248,9	150,6	199,1	104,3	0,80	0,606
KES1	35,9	19,8	36,9	27,9	1,03	0,956
KEX1	59,3	24,9	33,8	20,3	0,57	0,163
KEX2	35,4	33,1	19,1	12,0	0,54	0,392
KGD1	126,5	116,8	281,3	302,8	2,22	0,377
KGD2	90,4	13,9	163,1	38,2	1,80	0,012
KHA1	23,7	5,0	19,1	2,0	0,81	0,139
KIC1	16,7	10,4	24,9	18,4	1,49	0,467
KIN1	38,0	16,7	45,8	17,2	1,20	0,542
KIN2	44,1	20,5	49,6	7,2	1,12	0,631
KIN28	22,6	14,0	14,9	11,1	0,66	0,421
KIN3	13,0	7,6	15,2	13,5	1,17	0,782
KIN4	50,6	48,2	17,2	5,4	0,34	0,218
KIN82	23,8	3,4	65,1	22,7	2,74	0,011
KIP1	20,8	6,9	2,3	1,6	0,11	0,002
KIP2	21,8	8,7	12,8	3,9	0,59	0,110
KIP3	40,6	32,5	15,5	10,8	0,38	0,193
KKQ8	51,7	19,3	81,5	21,5	1,58	0,085
KNH1	11,0	4,8	5,9	4,3	0,54	0,167
KNS1	1,0	1,0	2,1	1,7	2,01	0,340
KOG1	20,6	10,7	13,5	3,5	0,65	0,252
KRE1	172,5	65,6	151,1	77,7	0,88	0,689
KRE11	22,8	15,6	22,1	16,6	0,97	0,957
KRE2	123,7	49,8	212,7	62,6	1,72	0,068

KRE27	131,3	34,8	115,6	26,1	0,88	0,497
KRE28	10,1	2,1	13,3	8,0	1,31	0,472
KRE29	1,0	1,2	2,9	2,9	2,83	0,281
KRE33	86,9	47,4	61,9	11,7	0,71	0,345
KRE5	50,7	11,4	40,1	10,1	0,79	0,211
KRE6	118,5	46,6	47,2	13,6	0,40	0,026
KRE9	131,5	54,3	145,5	74,7	1,11	0,773
KRI1	285,1	173,8	566,8	351,0	1,99	0,200
KRR1	228,4	42,2	219,1	91,0	0,96	0,859
KRS1	122,3	88,4	417,1	651,2	3,41	0,404
KSH1	138,6	70,8	197,9	94,0	1,43	0,352
KSP1	65,5	27,7	149,4	21,5	2,28	0,003
KSS1	5,6	2,2	4,3	3,0	0,76	0,498
KTI11	3,1	2,7	3,5	3,7	1,12	0,880
KTI12	154,6	47,5	170,7	59,3	1,10	0,685
KTR1	146,1	43,3	52,4	7,9	0,36	0,005
KTR2	18,1	19,3	11,0	4,6	0,60	0,497
KTR3	9,5	7,5	6,3	6,4	0,67	0,551
KTR4	107,2	54,1	110,6	56,5	1,03	0,933
KTR5	135,9	91,8	29,7	31,5	0,22	0,071
KTR6	112,7	68,2	73,4	77,7	0,65	0,476
KTR7	61,8	22,7	62,8	23,5	1,02	0,956
KXD1	31,5	5,3	34,4	9,5	1,09	0,614
LAA1	41,2	34,9	53,5	48,9	1,30	0,696
LAC1	53,0	8,6	27,0	14,6	0,51	0,022
LAG1	341,7	71,4	231,8	91,6	0,68	0,107
LAG2	12,0	4,1	10,4	4,5	0,87	0,628
LAP2	107,3	41,5	127,9	26,6	1,19	0,436
LAP3	143,8	102,5	265,0	209,6	1,84	0,339
LAP4	80,3	39,4	220,8	77,0	2,75	0,017
LAS1	134,7	84,7	156,0	70,4	1,16	0,712
LAS17	112,3	23,4	114,5	51,9	1,02	0,940
LAS21	44,0	16,4	39,3	5,2	0,89	0,605
LAT1	559,6	141,7	692,5	105,8	1,24	0,184
LCB1	170,5	48,3	134,9	28,5	0,79	0,251
LCB2	355,4	292,3	304,5	162,0	0,86	0,771
LCB3	276,5	182,0	164,4	130,1	0,59	0,355
LCB4	66,5	7,7	82,5	13,1	1,24	0,081
LCB5	52,9	23,7	43,1	18,2	0,81	0,535
LCD1	27,3	6,9	15,8	2,2	0,58	0,019
LCL3	71,4	17,4	40,5	13,7	0,57	0,031
LCP5	45,2	8,0	50,4	2,4	1,12	0,257
LDB16	85,4	37,8	59,6	28,9	0,70	0,321
LDB17	35,4	7,1	20,0	9,0	0,57	0,037
LDB18	120,2	56,0	53,3	12,2	0,44	0,058
LDB19	58,7	20,3	84,6	9,8	1,44	0,061
LDB7	200,4	129,6	121,9	80,9	0,61	0,344
LEA1	12,9	2,5	25,9	18,5	2,01	0,211
LEE1	3,3	1,4	5,7	3,8	1,73	0,283
LEM3	37,4	24,3	30,3	19,5	0,81	0,665

LEO1	106,2	101,3	103,1	70,2	0,97	0,962
LEU1	518,6	170,5	475,3	130,1	0,92	0,700
LEU3	18,4	8,5	23,5	15,9	1,28	0,593
LEU4	363,2	135,9	259,7	72,1	0,72	0,227
LEU5	21,1	5,6	12,3	8,2	0,58	0,129
LEU9	260,0	89,3	106,6	38,9	0,41	0,020
LGE1	26,7	9,6	34,1	18,3	1,28	0,501
LHP1	282,8	222,2	206,3	131,0	0,73	0,575
LHS1	109,0	31,4	72,9	19,8	0,67	0,100
LIA1	145,9	109,1	163,9	138,6	1,12	0,845
LIF1	5,9	3,4	11,3	9,6	1,91	0,332
LIN1	37,8	22,0	161,0	106,1	4,26	0,063
LIP1	75,8	36,3	112,9	90,3	1,49	0,475
LIP2	75,4	15,0	93,7	13,6	1,24	0,119
LIP5	94,7	31,5	117,9	22,6	1,25	0,276
LOC1	17,9	9,8	1,7	1,4	0,09	0,017
LOH1	2,2	1,5	7,6	11,0	3,45	0,369
LOS1	71,8	29,8	41,2	11,0	0,57	0,102
LOT5	16,0	7,5	17,8	5,0	1,11	0,712
LOT6	7,2	6,8	15,0	7,6	2,07	0,179
LPD1	483,9	194,5	546,0	281,9	1,13	0,729
LPP1	16,0	12,6	14,4	14,7	0,90	0,873
LPX1	41,6	29,5	19,8	12,3	0,48	0,221
LRE1	72,7	32,1	55,3	25,2	0,76	0,426
LRG1	20,5	10,9	18,5	4,1	0,90	0,740
LRO1	19,4	15,4	12,0	10,1	0,62	0,449
LRP1	91,1	39,6	92,2	51,2	1,01	0,973
LSB3	54,9	82,9	53,4	79,7	0,97	0,980
LSB5	45,9	34,1	49,8	43,3	1,08	0,892
LSB6	19,8	6,1	44,8	19,5	2,27	0,049
LSC1	198,0	105,5	73,3	17,0	0,37	0,058
LSC2	216,0	43,3	114,1	28,4	0,53	0,008
LSG1	93,1	59,2	62,4	26,3	0,67	0,381
LSM1	148,1	94,2	151,9	87,8	1,03	0,955
LSM12	186,4	46,0	114,1	18,9	0,61	0,027
LSM2	306,4	403,7	232,3	299,8	0,76	0,778
LSM4	0,9	0,2	0,1	0,2	0,10	0,001
LSM5	270,2	137,7	156,0	31,7	0,58	0,157
LSM6	187,2	74,9	238,0	24,5	1,27	0,245
LSM8	54,5	21,5	35,3	20,8	0,65	0,247
LSP1	495,9	197,9	667,1	308,9	1,35	0,387
LST4	103,8	31,0	57,5	15,0	0,55	0,036
LST7	2,0	1,6	3,0	3,3	1,47	0,626
LST8	16,1	7,2	43,1	17,9	2,68	0,031
LTE1	30,0	6,5	8,7	5,9	0,29	0,003
LTP1	55,1	17,2	41,7	5,9	0,76	0,190
LTV1	156,1	57,6	151,5	93,9	0,97	0,936
LYP1	163,1	87,8	143,3	107,9	0,88	0,785
LYS1	387,3	121,6	228,6	52,5	0,59	0,054
LYS12	421,6	96,9	298,0	175,7	0,71	0,264

LYS14	102,7	31,1	128,7	53,9	1,25	0,434
LYS2	385,9	226,7	313,1	192,4	0,81	0,642
LYS20	3105,9	1262,4	2100,0	596,2	0,68	0,200
LYS21	638,2	477,7	429,3	280,1	0,67	0,479
LYS4	138,9	82,9	111,8	71,6	0,80	0,638
LYS5	29,2	11,3	85,8	57,0	2,94	0,100
LYS9	1179,3	490,5	589,2	154,8	0,50	0,062
MAC1	33,2	15,8	67,7	65,5	2,04	0,346
MAD1	65,7	9,7	63,8	12,0	0,97	0,823
MAD2	88,5	50,1	50,7	10,0	0,57	0,189
MAD3	13,7	10,5	2,2	2,2	0,16	0,077
MAE1	736,5	578,7	366,2	281,8	0,50	0,294
MAF1	68,0	48,9	93,6	78,2	1,38	0,598
MAG1	78,9	16,3	130,9	46,0	1,66	0,077
MAG2	21,9	7,2	23,7	16,2	1,08	0,845
MAK10	15,9	9,7	9,1	8,8	0,58	0,343
MAK11	170,4	110,4	136,5	29,7	0,80	0,576
MAK16	56,8	26,1	62,2	21,8	1,10	0,760
MAK21	169,3	106,7	118,3	68,2	0,70	0,451
MAK3	63,4	22,8	17,8	9,2	0,28	0,010
MAK31	176,0	41,9	312,3	23,6	1,77	0,001
MAK32	22,7	2,8	21,4	2,8	0,94	0,502
MAK5	62,2	29,1	76,8	43,0	1,23	0,595
MAL11	4,2	3,4	2,2	1,7	0,52	0,332
MAL12	0,4	0,3	0,0	0,1	0,07	0,068
MAL13	1,8	0,6	4,9	3,9	2,70	0,169
MAL31	5,3	4,0	4,8	2,9	0,91	0,845
MAL32	5,4	2,3	14,2	7,4	2,63	0,065
MAL33	23,1	5,9	25,5	17,9	1,10	0,807
MAM3	48,0	7,1	79,5	32,9	1,66	0,110
MAM33	224,8	97,8	40,5	14,1	0,18	0,010
MAP1	155,5	204,0	134,3	123,9	0,86	0,865
MAP2	298,0	66,7	293,7	44,4	0,99	0,919
MAS1	49,2	8,6	50,9	34,5	1,04	0,925
MAS2	68,4	11,0	109,4	25,1	1,60	0,024
MBA1	96,6	30,8	44,0	28,8	0,46	0,047
MBB1	4,9	2,2	1,6	1,6	0,33	0,053
MBF1	827,4	511,5	1430,4	978,3	1,73	0,316
MBP1	48,6	22,8	38,6	31,4	0,79	0,622
MBR1	10,3	6,3	8,1	6,9	0,79	0,653
MCA1	281,7	85,3	259,6	46,0	0,92	0,665
MCD1	87,3	29,7	8,4	5,2	0,10	0,002
MCD4	111,8	48,8	15,8	10,5	0,14	0,009
MCH1	29,7	16,9	22,2	21,3	0,75	0,601
MCH2	0,9	1,2	0,7	0,6	0,78	0,780
MCH4	503,6	270,3	720,6	276,5	1,43	0,305
MCH5	52,5	37,8	91,7	54,0	1,74	0,280
MCK1	78,0	17,7	82,9	26,8	1,06	0,769
MCM1	100,4	36,9	55,9	40,5	0,56	0,155
MCM10	2,1	0,6	14,5	3,8	6,89	0,001

MCM16	14,8	3,9	13,8	6,7	0,93	0,790
MCM2	56,9	37,5	40,3	23,4	0,71	0,482
MCM21	11,9	5,5	7,1	5,5	0,59	0,259
MCM22	7,9	2,9	9,7	5,4	1,23	0,574
MCM3	34,3	28,0	19,3	10,3	0,56	0,353
MCM4	20,8	7,9	27,3	11,9	1,32	0,392
MCM5	39,4	26,9	55,3	39,5	1,40	0,531
MCM6	36,6	12,6	21,2	14,6	0,58	0,162
MCM7	37,0	9,9	33,9	12,2	0,92	0,712
MCR1	372,2	300,3	534,8	148,3	1,44	0,369
MCT1	25,8	12,1	116,3	24,0	4,51	0,001
MCX1	66,6	17,1	53,3	21,2	0,80	0,365
MDE1	168,3	109,1	130,9	57,4	0,78	0,567
MDG1	23,4	6,4	28,1	14,1	1,20	0,567
MDH1	490,9	321,1	820,6	508,6	1,67	0,315
MDH2	32,6	22,2	69,6	9,9	2,14	0,023
MDH3	66,7	55,7	64,6	48,8	0,97	0,958
MDJ1	162,1	88,9	418,7	76,5	2,58	0,005
MDJ2	2,6	0,9	9,2	6,9	3,52	0,107
MDL1	68,9	40,5	74,1	33,1	1,08	0,847
MDL2	58,2	31,5	56,2	14,7	0,96	0,910
MDM1	42,6	7,6	11,1	9,1	0,26	0,002
MDM10	23,7	3,0	23,6	3,9	1,00	0,983
MDM12	7,7	4,0	6,4	4,9	0,83	0,690
MDM20	97,0	55,6	59,5	14,6	0,61	0,240
MDM30	25,3	21,9	22,1	5,1	0,88	0,788
MDM31	19,3	8,4	12,0	10,4	0,62	0,315
MDM32	59,7	7,0	141,7	53,0	2,37	0,022
MDM34	95,1	33,0	98,9	60,0	1,04	0,915
MDM35	233,7	87,5	468,2	106,7	2,00	0,015
MDM36	4,3	3,2	7,6	6,3	1,79	0,377
MDM38	349,7	216,1	546,3	385,8	1,56	0,408
MDN1	99,0	54,4	65,0	47,2	0,66	0,381
MDR1	28,4	11,6	42,7	5,2	1,50	0,066
MDS3	19,2	10,6	41,9	5,9	2,19	0,009
MDV1	35,8	14,9	54,3	32,5	1,52	0,340
MDY2	111,5	108,1	91,1	68,5	0,82	0,760
MEC1	14,2	3,3	13,1	9,1	0,92	0,824
MEC3	26,7	10,7	21,9	10,0	0,82	0,536
MED1	25,4	11,3	40,0	32,3	1,58	0,426
MED11	36,0	11,4	34,4	11,5	0,96	0,851
MED2	102,1	32,6	127,1	24,6	1,25	0,265
MED4	13,4	9,1	8,6	7,4	0,64	0,442
MED6	32,1	30,9	56,0	30,6	1,75	0,312
MED7	29,8	9,0	49,4	37,9	1,66	0,355
MED8	19,4	13,6	35,6	24,5	1,84	0,292
MEF1	50,1	2,7	44,0	16,1	0,88	0,483
MEF2	7,8	3,9	9,1	6,5	1,18	0,729
MEI4	1,0	0,9	1,2	1,9	1,25	0,819
MEI5	0,1	0,2	0,0	0,0	0,00	0,356

MEK1	0,1	0,2	0,0	0,0	0,00	0,356
MEP1	26,9	8,8	6,5	5,5	0,24	0,008
MEP2	21,4	15,7	2,9	1,0	0,13	0,056
MEP3	32,3	13,5	7,1	5,0	0,22	0,013
MER1	0,2	0,3	0,1	0,3	0,72	0,830
MES1	218,0	52,1	346,3	62,6	1,59	0,020
MET1	17,2	10,8	54,9	24,3	3,19	0,030
MET10	38,3	9,2	99,2	54,4	2,59	0,070
MET12	39,8	38,7	37,9	37,3	0,95	0,945
MET13	13,6	12,3	13,7	14,6	1,00	0,996
MET14	51,0	31,1	171,8	141,5	3,37	0,146
MET16	34,4	9,7	114,7	18,1	3,34	0,000
MET18	30,3	12,9	25,6	1,2	0,84	0,494
MET2	27,9	29,3	68,4	32,2	2,45	0,113
MET22	129,1	48,5	118,3	32,9	0,92	0,726
MET28	10,6	3,7	51,0	20,2	4,80	0,008
MET3	22,5	6,6	43,5	21,9	1,93	0,116
MET30	25,2	11,3	28,3	6,6	1,12	0,649
MET31	33,9	1,9	31,0	9,4	0,91	0,559
MET32	4,0	1,8	6,8	6,2	1,69	0,422
MET4	82,9	15,7	94,0	27,5	1,13	0,507
MET5	30,0	11,3	82,3	34,0	2,74	0,027
MET7	64,6	30,9	28,3	12,8	0,44	0,073
MET8	17,3	5,5	40,7	22,2	2,35	0,087
MEU1	178,7	89,5	119,8	49,9	0,67	0,294
MEX67	70,9	34,4	114,6	55,9	1,62	0,231
MF(ALPHA)1	0,7	0,7	0,5	0,7	0,72	0,673
MF(ALPHA)2	0,6	0,6	0,7	0,5	1,22	0,745
MFA1	4881,9	1074,9	17405,4	6583,3	3,57	0,009
MFA2	78,9	38,7	230,8	147,1	2,93	0,093
MFB1	36,9	8,9	19,9	13,7	0,54	0,083
MFM1	10,0	2,7	14,3	6,8	1,43	0,285
MFT1	22,3	10,3	42,5	26,7	1,91	0,206
MGA1	0,9	0,3	2,7	2,4	3,08	0,186
MGA2	79,8	32,2	42,1	29,4	0,53	0,135
MGE1	258,7	78,6	155,0	22,7	0,60	0,044
MGM1	76,1	35,4	53,1	14,1	0,70	0,272
MGM101	156,5	108,0	70,4	47,4	0,45	0,194
MGR1	31,7	4,0	40,2	16,6	1,27	0,357
MGR2	74,2	63,4	29,8	26,9	0,40	0,246
MGR3	22,6	3,4	18,3	2,4	0,81	0,081
MGS1	12,4	7,2	25,8	23,0	2,07	0,311
MGT1	77,5	36,2	75,4	26,8	0,97	0,931
MHP1	113,5	29,8	92,1	14,9	0,81	0,246
MHR1	99,0	26,0	144,1	63,6	1,46	0,238
MHT1	1,1	0,4	2,1	2,0	2,04	0,317
MIA40	57,9	31,9	153,2	65,3	2,65	0,039
MIC14	81,7	50,6	61,2	30,3	0,75	0,512
MIC17	559,5	206,6	344,4	189,2	0,62	0,175
MID1	54,3	12,5	19,6	2,4	0,36	0,002

MID2	55,0	35,6	72,6	45,4	1,32	0,563
MIF2	78,9	17,1	40,5	11,4	0,51	0,010
MIG1	34,1	17,9	27,1	1,9	0,79	0,465
MIG2	19,6	9,4	15,8	12,6	0,81	0,649
MIG3	14,8	7,7	12,7	9,1	0,86	0,736
MIH1	10,5	7,0	4,5	4,6	0,43	0,204
MIM1	79,3	60,5	103,1	90,1	1,30	0,677
MIP1	54,3	38,3	36,8	34,9	0,68	0,524
MIP6	5,1	1,8	9,3	8,0	1,83	0,343
MIR1	180,1	108,0	503,1	76,8	2,79	0,003
MIS1	131,1	18,1	67,2	14,9	0,51	0,002
MKC7	66,7	36,2	20,8	9,4	0,31	0,050
MKK1	34,5	10,6	33,8	22,6	0,98	0,957
MKK2	46,6	32,6	12,2	8,2	0,26	0,086
MKS1	31,2	5,9	34,8	3,1	1,12	0,314
MKT1	292,1	80,3	252,0	126,8	0,86	0,612
MLC1	403,4	299,3	490,5	331,9	1,22	0,710
MLC2	71,2	42,2	85,1	44,5	1,19	0,667
MLF3	43,4	18,6	38,5	15,2	0,89	0,698
MLH1	6,8	4,2	8,1	6,7	1,20	0,749
MLH2	9,3	3,4	24,7	22,7	2,66	0,228
MLH3	37,7	22,3	35,6	19,0	0,95	0,893
MLP1	97,1	81,6	70,3	52,6	0,72	0,600
MLP2	140,0	94,2	183,0	46,9	1,31	0,445
MLS1	6,2	2,1	12,7	9,9	2,06	0,245
MMF1	323,4	88,1	440,3	122,4	1,36	0,172
MMM1	17,3	12,7	58,9	31,8	3,41	0,051
MMP1	8,8	3,9	25,8	20,3	2,94	0,151
MMR1	43,7	16,5	54,4	12,9	1,24	0,346
MMS1	3,9	1,0	10,0	6,9	2,58	0,132
MMS2	102,6	39,9	76,2	16,9	0,74	0,269
MMS21	0,2	0,3	1,7	1,5	7,19	0,106
MMS22	43,3	17,0	32,9	24,6	0,76	0,513
MMS4	24,6	7,0	9,5	7,8	0,39	0,028
MMT1	16,6	2,9	7,4	5,1	0,44	0,019
MMT2	14,9	5,8	40,1	13,9	2,68	0,016
MND1	1,3	1,0	0,9	0,7	0,71	0,530
MND2	13,4	8,2	7,0	5,7	0,52	0,246
MNE1	13,9	7,1	30,0	23,6	2,16	0,238
MNL1	72,4	32,6	40,5	25,6	0,56	0,174
MNN1	86,9	53,2	19,8	11,5	0,23	0,049
MNN10	38,6	25,0	40,9	43,3	1,06	0,931
MNN11	123,7	32,6	77,7	27,4	0,63	0,074
MNN2	92,8	30,1	60,2	17,9	0,65	0,112
MNN4	18,9	11,2	54,8	23,6	2,90	0,034
MNN5	174,1	74,2	124,5	39,2	0,71	0,282
MNN9	744,1	333,7	554,2	273,4	0,74	0,413
MNP1	487,6	199,6	677,7	121,2	1,39	0,155
MNR2	43,8	24,1	51,3	23,2	1,17	0,673
MNS1	17,5	6,0	11,2	7,6	0,64	0,243

MNT2	68,9	24,4	53,4	21,4	0,77	0,376
MNT3	38,0	10,6	32,3	8,8	0,85	0,442
MNT4	39,8	19,3	48,0	47,8	1,21	0,762
MOB1	47,5	23,6	49,7	32,6	1,05	0,919
MOB2	53,5	67,9	61,1	81,1	1,14	0,891
MOD5	20,3	6,2	18,2	14,3	0,90	0,798
MOG1	113,2	78,2	72,0	11,2	0,64	0,338
MON1	140,4	38,5	109,0	20,6	0,78	0,201
MON2	32,8	16,5	34,6	27,5	1,06	0,913
MOT1	24,1	13,5	46,0	69,4	1,91	0,557
MOT2	112,6	34,0	90,6	10,1	0,80	0,261
MOT3	38,6	14,0	21,7	14,5	0,56	0,144
MPA43	18,5	7,5	18,3	15,5	0,99	0,975
MPC54	8,2	3,0	19,6	13,0	2,40	0,137
MPD1	9,8	8,4	7,3	11,2	0,75	0,737
MPD2	38,2	14,8	27,0	19,1	0,71	0,391
MPE1	32,8	14,0	74,5	102,4	2,27	0,450
MPH1	42,3	10,1	37,3	16,6	0,88	0,619
MPH2	11,0	6,2	5,5	3,9	0,49	0,178
MPM1	66,6	21,6	138,2	62,4	2,08	0,073
MPP10	92,1	20,0	98,5	9,9	1,07	0,582
MPP6	20,3	7,3	20,8	17,8	1,02	0,964
MPS1	41,5	15,2	33,6	20,3	0,81	0,557
MPS2	6,2	1,8	1,6	1,3	0,27	0,007
MPS3	1,8	1,3	0,5	0,5	0,27	0,101
MPT5	37,7	19,3	79,1	54,3	2,10	0,201
MRC1	83,3	35,8	27,4	21,2	0,33	0,036
MRD1	48,3	25,7	66,5	33,6	1,38	0,424
MRE11	55,0	9,3	59,8	9,5	1,09	0,494
MRF1	34,7	17,7	29,3	21,9	0,84	0,712
MRH1	932,3	480,4	2558,0	1376,6	2,74	0,067
MRH4	36,1	9,7	38,9	29,2	1,08	0,863
MRI1	148,5	40,1	74,2	23,1	0,50	0,018
MRK1	6,5	2,3	3,4	2,8	0,52	0,139
MRL1	24,8	16,1	63,2	58,1	2,54	0,251
MRM1	68,1	10,5	66,8	20,6	0,98	0,910
MRM2	31,9	7,6	35,6	6,7	1,12	0,492
MRN1	119,4	65,9	78,3	44,3	0,66	0,341
MRP1	72,4	37,2	104,9	75,2	1,45	0,468
MRP10	124,3	103,4	153,0	108,0	1,23	0,715
MRP13	33,9	7,9	35,4	5,6	1,04	0,767
MRP17	138,2	50,7	179,8	22,4	1,30	0,184
MRP2	1371,2	940,3	513,3	204,5	0,37	0,125
MRP20	30,5	8,6	55,5	18,0	1,82	0,046
MRP21	68,8	34,9	55,0	23,7	0,80	0,536
MRP4	74,3	35,8	70,4	35,4	0,95	0,882
MRP49	38,7	16,8	55,2	12,1	1,42	0,163
MRP51	81,6	32,8	88,7	28,5	1,09	0,754
MRP7	99,8	62,4	57,7	59,3	0,58	0,365
MRPL1	221,8	107,8	180,4	117,0	0,81	0,622

MRPL10	61,4	21,3	65,2	17,5	1,06	0,792
MRPL11	52,8	16,3	69,0	12,3	1,31	0,164
MRPL13	55,7	17,5	60,1	17,7	1,08	0,740
MRPL15	29,3	14,0	29,5	12,2	1,01	0,982
MRPL16	116,4	75,2	199,5	171,2	1,71	0,409
MRPL17	147,6	77,4	139,4	31,2	0,94	0,850
MRPL20	119,0	25,8	80,8	25,7	0,68	0,081
MRPL22	57,9	18,3	65,1	21,0	1,12	0,622
MRPL23	184,7	86,4	107,5	25,6	0,58	0,137
MRPL25	145,7	70,7	154,1	105,6	1,06	0,899
MRPL27	164,2	56,2	83,0	41,2	0,51	0,059
MRPL28	9,2	6,6	2,1	1,7	0,23	0,082
MRPL3	30,3	3,4	41,1	27,2	1,36	0,461
MRPL31	180,9	93,3	69,1	9,1	0,38	0,054
MRPL32	76,0	24,4	109,2	89,5	1,44	0,501
MRPL33	30,7	36,6	44,7	52,8	1,46	0,678
MRPL35	21,4	15,8	17,1	13,0	0,80	0,693
MRPL37	102,0	83,5	88,8	99,4	0,87	0,846
MRPL38	63,5	24,6	69,7	33,6	1,10	0,776
MRPL39	228,2	49,9	209,7	40,5	0,92	0,586
MRPL4	54,1	12,8	47,9	35,8	0,89	0,755
MRPL40	15,1	16,5	43,0	66,6	2,84	0,448
MRPL44	155,3	71,1	150,0	62,7	0,97	0,915
MRPL49	320,2	206,7	276,1	168,7	0,86	0,752
MRPL50	157,3	93,5	194,3	61,4	1,23	0,533
MRPL51	193,5	64,8	184,4	45,3	0,95	0,824
MRPL6	81,2	56,1	91,2	24,5	1,12	0,755
MRPL7	101,1	86,3	108,0	118,3	1,07	0,928
MRPL8	51,4	16,2	49,9	21,2	0,97	0,914
MRPL9	145,0	51,3	92,7	34,5	0,64	0,142
MRPS12	289,7	75,5	254,7	129,9	0,88	0,658
MRPS16	95,1	97,1	127,1	128,7	1,34	0,705
MRPS17	215,6	130,5	158,2	98,2	0,73	0,508
MRPS18	147,0	25,0	130,3	8,6	0,89	0,252
MRPS28	73,6	53,3	53,2	4,9	0,72	0,476
MRPS35	102,8	71,6	56,4	22,9	0,55	0,263
MRPS5	65,0	17,4	86,6	6,7	1,33	0,060
MRPS8	128,5	74,2	186,6	100,5	1,45	0,388
MRPS9	112,0	44,5	153,5	101,5	1,37	0,482
MRS1	53,4	15,2	38,0	25,5	0,71	0,340
MRS2	30,7	6,7	18,8	2,7	0,61	0,017
MRS3	83,7	11,8	37,1	17,8	0,44	0,005
MRS4	14,8	6,9	26,5	17,7	1,79	0,264
MRS6	244,3	97,1	147,3	75,6	0,60	0,166
MRT4	265,5	162,9	250,9	170,2	0,95	0,906
MSA1	109,5	61,7	118,3	71,8	1,08	0,859
MSB1	39,0	16,8	28,8	12,0	0,74	0,361
MSB2	66,6	15,4	42,5	16,0	0,64	0,073
MSB3	12,8	8,8	16,4	7,4	1,28	0,551
MSB4	32,4	6,1	15,3	10,4	0,47	0,030

MSC1	26,6	11,2	93,1	67,4	3,50	0,099
MSC2	7,3	3,5	17,1	7,7	2,35	0,060
MSC3	25,0	22,4	26,1	14,8	1,04	0,937
MSC6	22,7	10,4	9,8	8,5	0,43	0,103
MSC7	70,5	38,4	47,5	28,9	0,67	0,374
MSD1	21,0	11,7	32,4	16,9	1,54	0,311
MSE1	40,4	8,6	27,4	5,1	0,68	0,040
MSF1	24,5	13,9	52,6	33,6	2,15	0,172
MSG5	21,0	7,6	18,4	17,0	0,88	0,791
MSH1	56,6	37,8	56,0	30,8	0,99	0,980
MSH2	89,5	51,1	21,5	14,8	0,24	0,043
MSH3	29,7	14,8	35,4	10,6	1,19	0,558
MSH4	2,4	0,9	5,7	3,9	2,42	0,144
MSH5	9,0	5,7	10,6	8,7	1,18	0,763
MSH6	141,5	50,6	37,8	15,0	0,27	0,008
MSI1	101,5	61,5	77,8	34,2	0,77	0,526
MSK1	47,6	25,7	34,3	16,0	0,72	0,413
MSL1	21,4	7,1	27,9	5,5	1,30	0,197
MSL5	12,6	3,7	10,1	9,6	0,80	0,648
MSM1	27,0	10,5	20,4	2,8	0,75	0,271
MSN1	25,7	11,8	23,2	15,6	0,90	0,800
MSN2	71,6	41,5	73,4	25,6	1,03	0,942
MSN4	19,5	10,4	21,4	14,6	1,10	0,836
MSN5	122,2	32,4	138,2	53,0	1,13	0,626
MSO1	45,9	36,3	24,5	17,6	0,53	0,330
MSP1	29,4	5,1	48,9	26,6	1,66	0,201
MSR1	100,9	56,4	69,7	15,2	0,69	0,328
MSS1	23,5	9,0	20,4	6,1	0,87	0,586
MSS11	9,0	4,5	15,2	3,5	1,69	0,075
MSS116	71,0	37,1	76,0	23,2	1,07	0,828
MSS18	30,6	20,8	26,6	16,1	0,87	0,769
MSS2	33,2	22,8	54,6	62,3	1,65	0,542
MSS4	65,3	15,0	62,2	17,3	0,95	0,795
MSS51	102,4	39,9	100,9	72,6	0,99	0,973
MST1	33,4	9,0	32,2	3,4	0,97	0,819
MST28	28,1	13,6	41,4	23,7	1,47	0,367
MSW1	20,1	8,8	29,1	7,3	1,45	0,166
MSY1	30,6	20,5	11,6	8,2	0,38	0,135
MTC1	183,5	33,6	365,8	144,0	1,99	0,049
MTC2	79,6	42,6	54,0	42,3	0,68	0,427
MTC4	41,1	24,5	28,3	11,7	0,69	0,382
MTC5	12,3	7,1	26,8	14,7	2,18	0,125
MTC6	19,8	10,6	22,2	7,8	1,12	0,726
MTC7	2,4	2,1	0,5	0,6	0,23	0,152
MTD1	398,2	201,2	82,0	68,9	0,21	0,025
MTF1	33,0	14,8	41,0	12,0	1,24	0,432
MTF2	30,9	15,9	27,8	20,0	0,90	0,819
MTG1	49,5	28,6	87,4	56,3	1,77	0,275
MTG2	12,0	1,6	12,3	7,7	1,02	0,954
MTH1	11,7	11,3	55,8	48,0	4,77	0,124

MTL1	7,4	4,3	20,8	13,9	2,82	0,116
MTM1	78,0	19,7	77,4	36,2	0,99	0,979
MTO1	71,2	67,8	54,9	26,2	0,77	0,670
MTQ1	32,7	18,6	50,5	27,6	1,54	0,326
MTQ2	26,6	15,8	50,9	60,3	1,92	0,465
MTR10	34,7	17,0	41,6	4,5	1,20	0,461
MTR2	82,2	40,1	100,6	23,2	1,22	0,458
MTR3	39,5	11,0	25,8	9,5	0,65	0,109
MTR4	145,9	44,5	124,8	35,3	0,86	0,485
MTW1	16,5	9,0	6,6	4,5	0,40	0,093
MUB1	29,1	23,0	51,1	66,7	1,76	0,556
MUC1	8,3	1,0	2,4	1,7	0,29	0,001
MUD1	19,0	6,1	14,4	3,3	0,76	0,231
MUD2	36,1	30,1	25,1	9,1	0,70	0,511
MUK1	18,5	7,4	32,5	4,9	1,76	0,020
MUM2	78,2	74,9	34,0	34,4	0,43	0,325
MUM3	50,3	36,4	92,5	72,7	1,84	0,339
MUP1	237,8	181,4	187,1	48,9	0,79	0,609
MUP3	44,8	28,1	65,2	51,4	1,45	0,513
MUS81	15,2	6,0	7,0	5,4	0,46	0,091
MVB12	18,4	9,6	20,4	11,2	1,11	0,791
MVD1	99,7	29,7	120,5	39,4	1,21	0,431
MVP1	57,1	32,7	65,2	40,3	1,14	0,764
MXR1	140,5	50,0	150,6	93,7	1,07	0,855
MXR2	123,5	31,2	163,0	19,6	1,32	0,076
MYO1	50,3	21,8	44,1	14,1	0,88	0,651
MYO2	161,5	83,0	102,7	21,5	0,64	0,219
MYO3	21,9	3,9	70,5	55,3	3,22	0,130
MYO4	54,1	27,5	41,5	20,0	0,77	0,488
MYO5	37,1	15,0	35,3	23,6	0,95	0,899
MZM1	61,0	33,0	45,6	27,3	0,75	0,499
NAB2	189,5	112,8	328,4	185,0	1,73	0,247
NAB3	118,7	56,3	112,2	25,8	0,95	0,841
NAB6	3,0	0,8	37,2	43,1	12,56	0,163
NAF1	26,2	13,2	30,6	18,9	1,17	0,717
NAG1	0,1	0,1	0,1	0,2	2,33	0,618
NAM2	32,9	10,8	24,6	16,6	0,75	0,432
NAM7	4,1	2,3	6,3	5,5	1,53	0,490
NAM8	35,3	7,1	52,7	22,0	1,49	0,183
NAM9	43,6	16,6	41,6	26,8	0,95	0,901
NAN1	67,4	38,7	63,3	39,8	0,94	0,886
NAP1	293,9	78,5	769,2	182,4	2,62	0,003
NAR1	26,9	9,6	33,7	8,9	1,25	0,339
NAS2	35,1	18,2	53,3	34,3	1,52	0,384
NAS6	49,9	18,4	49,4	11,4	0,99	0,961
NAT1	226,9	127,2	152,0	29,5	0,67	0,295
NAT2	122,6	86,6	97,5	7,0	0,80	0,585
NAT3	83,6	38,4	59,7	11,3	0,71	0,278
NAT4	4,7	3,7	1,0	1,2	0,21	0,102
NAT5	239,5	195,2	178,7	112,2	0,75	0,609

NBA1	51,8	14,3	56,2	13,2	1,08	0,669
NBL1	2,1	0,9	2,4	2,4	1,13	0,834
NBP1	53,6	20,6	51,8	37,5	0,97	0,937
NBP2	15,4	22,7	20,6	29,0	1,34	0,788
NBP35	48,2	30,4	44,5	26,3	0,92	0,863
NCA2	34,8	15,7	13,5	9,8	0,39	0,061
NCA3	23,6	7,0	88,5	50,8	3,76	0,045
NCB2	132,9	75,0	148,9	64,1	1,12	0,756
NCE102	570,3	368,2	948,8	643,0	1,66	0,346
NCE103	87,4	58,9	132,5	147,5	1,51	0,591
NCL1	262,4	162,1	212,8	106,7	0,81	0,628
NCP1	255,4	123,3	130,4	70,7	0,51	0,129
NCR1	73,1	35,4	66,1	28,3	0,90	0,767
NCS2	39,2	11,4	58,8	27,4	1,50	0,235
NCS6	79,2	40,5	67,3	27,0	0,85	0,643
NDC1	122,9	47,8	136,6	104,0	1,11	0,819
NDD1	101,5	78,8	26,4	22,0	0,26	0,116
NDE1	97,6	14,3	44,6	8,8	0,46	0,001
NDE2	4,0	1,2	5,1	3,8	1,28	0,590
NDI1	39,6	11,6	100,1	20,2	2,53	0,002
NDJ1	18,5	14,1	24,3	7,2	1,31	0,492
NDL1	29,3	11,6	50,7	29,5	1,73	0,226
NDT80	1,7	1,2	2,4	2,1	1,39	0,601
NEJ1	64,1	31,6	47,6	38,5	0,74	0,534
NEM1	17,6	3,1	19,6	14,2	1,12	0,788
NEO1	35,1	9,3	23,2	9,5	0,66	0,123
NET1	176,6	12,7	142,7	39,9	0,81	0,156
NEW1	142,0	8,4	158,3	26,3	1,11	0,282
NFI1	4,7	1,9	4,9	4,0	1,03	0,949
NFS1	66,9	10,6	92,1	11,6	1,38	0,018
NFT1	13,5	11,9	12,7	2,4	0,94	0,899
NFU1	71,9	41,3	105,1	28,0	1,46	0,231
NGG1	33,1	13,4	41,0	13,5	1,24	0,442
NGL1	4,2	2,1	16,0	10,4	3,79	0,068
NGL2	22,5	7,7	26,2	7,9	1,16	0,530
NGL3	2,4	0,9	11,5	8,3	4,87	0,070
NGR1	36,1	25,0	31,6	25,8	0,87	0,808
NHA1	43,3	25,8	33,6	27,7	0,78	0,627
NHP6A	239,9	57,5	249,6	22,3	1,04	0,764
NHX1	44,3	15,8	58,3	38,0	1,32	0,522
NIC96	43,7	19,0	23,2	16,9	0,53	0,159
NIF3	75,7	51,1	87,9	83,3	1,16	0,811
NIP1	108,7	43,4	115,5	44,5	1,06	0,834
NIP100	10,2	4,8	12,6	7,2	1,24	0,593
NIP7	372,0	165,9	263,7	96,0	0,71	0,302
NIS1	116,0	71,5	95,2	67,5	0,82	0,687
NIT1	2,9	1,9	6,1	7,0	2,06	0,425
NIT2	16,7	10,8	5,5	4,7	0,33	0,106
NIT3	92,3	100,9	90,4	68,4	0,98	0,977
NKP1	206,2	205,7	172,9	187,7	0,84	0,819

NKP2	0,2	0,3	0,3	0,5	1,16	0,900
NMA1	102,8	32,6	94,0	54,4	0,91	0,790
NMA111	43,7	18,3	82,6	26,3	1,89	0,051
NMA2	40,2	17,6	35,4	16,6	0,88	0,705
NMD2	52,0	20,2	72,1	13,8	1,39	0,152
NMD3	170,7	58,7	43,5	20,8	0,25	0,006
NMD4	34,1	15,8	34,2	15,0	1,00	0,995
NMD5	44,2	20,8	52,1	14,8	1,18	0,561
NMT1	102,7	69,7	94,2	70,5	0,92	0,870
NNF1	41,6	6,4	34,7	11,4	0,83	0,334
NNF2	109,0	39,7	68,5	51,8	0,63	0,261
NNK1	19,5	6,5	45,4	60,0	2,33	0,424
NNT1	121,5	36,4	85,4	38,8	0,70	0,224
NOB1	119,2	77,2	107,9	49,4	0,91	0,813
NOC2	178,7	81,1	146,8	84,3	0,82	0,605
NOC3	79,6	47,8	70,9	50,0	0,89	0,810
NOC4	73,9	28,1	72,6	36,8	0,98	0,956
NOG1	118,4	78,9	139,3	87,7	1,18	0,735
NOG2	131,1	63,6	114,1	35,2	0,87	0,657
NOP1	613,1	118,7	400,3	90,4	0,65	0,029
NOP12	142,1	27,7	87,2	36,4	0,61	0,053
NOP13	310,3	107,4	234,3	79,5	0,76	0,299
NOP14	82,6	11,8	84,0	18,4	1,02	0,902
NOP15	68,7	35,7	64,4	37,3	0,94	0,876
NOP2	289,7	156,2	332,0	190,3	1,15	0,743
NOP4	235,6	116,3	243,4	129,9	1,03	0,931
NOP53	196,1	63,2	166,2	49,3	0,85	0,483
NOP56	1018,5	401,8	1493,9	717,5	1,47	0,292
NOP58	401,6	177,4	329,4	67,5	0,82	0,476
NOP6	232,9	137,3	227,3	125,9	0,98	0,954
NOP7	59,6	29,7	60,9	27,9	1,02	0,951
NOP8	50,6	19,2	65,8	39,7	1,30	0,516
NOP9	51,3	30,9	40,3	27,9	0,79	0,619
NOT3	45,4	21,8	39,2	28,0	0,86	0,740
NOT5	74,0	15,2	68,2	9,9	0,92	0,548
NPA3	139,3	92,0	117,7	85,7	0,85	0,743
NPC2	56,3	39,0	45,2	20,2	0,80	0,630
NPL3	146,5	59,3	620,5	331,3	4,23	0,030
NPL4	23,6	17,1	43,7	27,5	1,85	0,262
NPL6	65,4	40,5	44,7	15,3	0,68	0,376
NPP1	33,9	2,8	39,7	19,0	1,17	0,573
NPP2	33,7	23,9	41,5	14,0	1,23	0,595
NPR1	59,7	9,3	60,4	21,4	1,01	0,954
NPR2	17,2	2,5	22,5	4,1	1,31	0,072
NPR3	36,8	15,8	38,2	6,1	1,04	0,882
NPT1	223,8	32,1	240,8	23,2	1,08	0,424
NPY1	63,9	17,4	34,1	23,3	0,53	0,087
NQM1	12,8	2,9	188,8	144,0	14,77	0,050
NRD1	141,3	62,7	44,2	20,8	0,31	0,026
NRG1	53,8	36,1	97,4	86,0	1,81	0,387

NRG2	66,8	56,3	150,2	104,8	2,25	0,210
NRK1	79,4	23,6	80,0	58,6	1,01	0,985
NRM1	30,0	13,2	4,2	3,6	0,14	0,009
NRP1	105,1	37,1	103,4	36,8	0,98	0,949
NRT1	13,4	5,1	28,8	18,9	2,14	0,168
NSA1	89,1	48,1	77,0	45,7	0,86	0,728
NSA2	76,3	39,3	124,3	40,2	1,63	0,139
NSE1	52,0	28,9	26,6	9,6	0,51	0,147
NSE3	136,5	81,8	225,2	57,7	1,65	0,127
NSE4	14,4	6,4	13,9	9,8	0,97	0,935
NSE5	20,3	16,5	13,4	9,6	0,66	0,498
NSG1	202,1	88,6	92,3	10,4	0,46	0,049
NSG2	57,6	70,5	89,0	109,3	1,55	0,646
NSL1	17,9	5,8	12,4	3,5	0,69	0,151
NSP1	0,2	0,5	0,2	0,3	1,04	0,973
NSR1	416,7	115,9	361,9	184,6	0,87	0,633
NST1	232,5	118,9	264,5	116,0	1,14	0,713
NTA1	14,5	6,2	45,0	15,8	3,10	0,011
NTE1	102,6	45,3	38,6	24,6	0,38	0,048
NTF2	692,1	397,7	703,7	400,0	1,02	0,968
NTG1	45,9	16,3	45,1	19,7	0,98	0,954
NTG2	27,7	6,4	31,4	16,4	1,13	0,693
NTH1	129,4	47,8	280,2	109,8	2,17	0,045
NTH2	79,3	3,1	47,8	16,2	0,60	0,009
NTO1	40,0	20,6	6,9	5,0	0,17	0,020
NTR2	137,1	74,5	138,0	62,4	1,01	0,985
NUC1	163,4	137,9	138,2	105,1	0,85	0,781
NUD1	17,5	3,5	2,2	1,8	0,13	0,000
NUF2	22,6	9,1	18,1	6,5	0,80	0,451
NUG1	105,8	49,5	93,1	61,1	0,88	0,756
NUM1	66,1	10,2	97,6	35,8	1,48	0,142
NUP1	92,9	30,7	124,7	35,2	1,34	0,223
NUP100	121,9	117,3	117,8	104,4	0,97	0,960
NUP116	41,8	10,3	32,7	12,0	0,78	0,293
NUP120	136,3	49,7	57,0	27,9	0,42	0,032
NUP133	30,4	18,3	38,2	19,1	1,25	0,580
NUP145	116,2	37,5	95,2	33,9	0,82	0,439
NUP157	51,6	12,5	49,1	16,7	0,95	0,817
NUP159	172,7	15,5	145,3	39,1	0,84	0,240
NUP170	148,2	24,3	99,6	44,3	0,67	0,102
NUP188	147,9	109,0	154,3	85,0	1,04	0,929
NUP192	118,5	78,3	110,4	52,8	0,93	0,870
NUP2	109,6	54,1	71,6	21,9	0,65	0,240
NUP42	22,5	5,1	16,6	4,0	0,74	0,117
NUP49	164,4	152,8	99,8	51,7	0,61	0,453
NUP53	48,2	14,2	62,2	51,7	1,29	0,620
NUP57	198,5	103,3	132,5	50,7	0,67	0,295
NUP60	50,5	31,3	35,8	3,7	0,71	0,387
NUP82	59,2	21,1	112,4	37,2	1,90	0,047
NUP84	88,4	50,0	69,4	37,8	0,79	0,567

NUP85	83,2	24,1	73,1	16,1	0,88	0,513
NUR1	52,9	40,0	43,7	14,0	0,83	0,679
NUS1	59,5	31,6	52,9	21,4	0,89	0,744
NUT1	143,0	90,0	116,5	50,1	0,82	0,626
NUT2	2,1	1,7	6,7	6,9	3,18	0,240
NVJ1	51,7	45,7	127,6	102,8	2,47	0,226
NYV1	131,5	20,8	177,7	55,1	1,35	0,168
OAC1	135,9	56,9	231,3	114,2	1,70	0,185
OAF1	14,1	3,9	18,6	13,6	1,32	0,548
OAF3	18,6	9,6	18,7	14,7	1,01	0,989
OAR1	1,4	1,2	6,5	5,0	4,74	0,091
OAZ1	18,0	14,7	19,4	7,1	1,08	0,865
OCA1	22,7	12,6	34,4	25,8	1,51	0,449
OCA4	139,6	73,2	112,0	58,9	0,80	0,578
OCA5	47,8	13,0	43,1	21,1	0,90	0,715
OCA6	26,4	5,8	36,9	4,8	1,40	0,032
OCH1	67,3	25,0	15,2	10,2	0,23	0,008
OCT1	20,5	5,1	24,5	5,6	1,19	0,336
ODC1	31,0	11,3	52,9	27,0	1,70	0,187
ODC2	98,8	14,4	23,6	8,1	0,24	0,000
OGG1	39,4	15,1	7,2	3,7	0,18	0,006
OKP1	50,6	18,0	40,5	13,6	0,80	0,406
OLA1	1377,1	664,4	1186,3	275,2	0,86	0,615
OLE1	251,0	159,9	54,7	14,6	0,22	0,050
OM45	62,7	26,1	197,6	153,7	3,15	0,134
OMA1	44,9	24,3	39,4	19,5	0,88	0,737
OMS1	24,5	5,3	35,4	5,3	1,44	0,027
OPI1	51,6	14,1	46,9	12,2	0,91	0,636
OPI10	18,3	9,4	64,3	54,4	3,52	0,147
OPI3	296,9	259,3	793,6	755,3	2,67	0,260
OPI6	0,7	0,7	0,2	0,2	0,23	0,188
OPI8	13,5	13,0	9,6	9,9	0,71	0,649
OPI9	0,1	0,1	0,2	0,2	2,56	0,476
OPT1	20,6	15,3	17,9	17,0	0,87	0,824
OPT2	101,9	69,0	39,1	26,5	0,38	0,140
OPY1	42,1	35,6	17,2	15,4	0,41	0,245
OPY2	65,9	20,6	39,8	3,4	0,60	0,047
ORC1	48,3	15,7	26,9	7,8	0,56	0,051
ORC2	27,3	13,8	11,9	8,0	0,44	0,103
ORC3	37,1	11,6	34,7	7,2	0,94	0,740
ORC4	17,8	19,1	67,7	113,7	3,81	0,419
ORC5	23,0	3,0	32,9	6,8	1,43	0,037
ORC6	18,9	4,6	24,8	13,2	1,31	0,427
ORM1	32,5	14,9	25,9	20,6	0,80	0,622
ORM2	11,8	14,5	122,7	232,9	10,41	0,379
ORT1	112,1	18,9	97,2	40,5	0,87	0,530
OSH2	76,2	17,8	72,1	19,3	0,95	0,764
OSH3	218,2	185,3	203,0	170,0	0,93	0,908
OSH6	77,8	37,7	76,1	29,7	0,98	0,947
OSH7	57,7	82,4	62,1	69,9	1,08	0,937

OSM1	77,5	19,2	159,4	41,4	2,06	0,011
OST1	277,6	65,1	150,8	26,5	0,54	0,011
OST2	245,0	84,1	165,9	78,7	0,68	0,219
OST3	54,0	35,0	39,3	21,8	0,73	0,505
OST4	361,1	78,4	416,1	152,0	1,15	0,544
OST5	36,6	17,4	6,8	4,6	0,19	0,016
OST6	99,9	23,5	83,3	49,7	0,83	0,567
OSW1	0,8	0,9	0,9	0,9	1,19	0,811
OSW2	4,3	1,4	5,6	4,2	1,30	0,583
OSW5	36,6	13,4	37,9	3,8	1,04	0,851
OTU1	47,0	14,6	88,5	13,2	1,88	0,006
OTU2	49,3	13,6	28,4	8,5	0,58	0,040
OXA1	108,0	66,5	62,6	38,5	0,58	0,282
OXP1	21,3	3,9	7,0	4,9	0,33	0,004
OXR1	17,0	4,0	20,4	8,8	1,20	0,515
OYE2	683,3	330,6	928,1	403,0	1,36	0,384
OYE3	7,2	4,8	4,4	3,0	0,60	0,346
PAA1	389,4	30,9	365,5	42,8	0,94	0,400
PAB1	552,4	207,7	657,5	202,9	1,19	0,496
PAC1	15,0	8,3	13,3	9,1	0,89	0,799
PAC11	17,3	15,5	3,4	2,7	0,19	0,127
PAC2	17,9	5,1	7,6	5,1	0,42	0,029
PAD1	67,0	21,1	69,0	10,4	1,03	0,874
PAF1	45,1	30,0	54,0	24,5	1,20	0,661
PAH1	14,6	10,8	24,3	20,3	1,66	0,433
PAM1	231,8	61,8	148,9	69,2	0,64	0,124
PAM16	197,3	165,3	170,8	126,9	0,87	0,808
PAM17	60,9	29,4	68,4	49,5	1,12	0,804
PAM18	75,9	7,9	90,1	17,4	1,19	0,189
PAN1	118,4	33,0	171,6	92,9	1,45	0,322
PAN2	66,5	53,9	44,9	22,1	0,68	0,487
PAN3	46,0	8,2	29,5	19,7	0,64	0,172
PAN5	75,9	91,4	30,8	32,7	0,41	0,389
PAP1	88,4	28,3	86,8	12,1	0,98	0,921
PAP2	19,2	5,2	13,0	11,0	0,67	0,341
PAR32	129,5	46,0	164,6	50,5	1,27	0,343
PAT1	153,3	59,3	126,6	27,8	0,83	0,446
PAU10	0,4	0,4	0,1	0,2	0,24	0,227
PAU13	0,1	0,1	0,4	0,4	4,17	0,250
PAU15	2,6	0,9	1,4	1,0	0,55	0,116
PAU17	10,1	3,4	27,3	27,9	2,70	0,267
PAU18	0,7	1,4	0,0	0,0	0,00	0,356
PAU19	0,5	0,5	0,3	0,4	0,54	0,491
PAU20	14,4	2,5	4,7	3,5	0,33	0,004
PAU23	13,6	10,3	18,9	11,3	1,39	0,514
PAU24	0,0	0,0	0,0	0,0	#DIV/0!	#DIV/0!
PAU3	3,6	1,8	12,0	13,7	3,38	0,268
PAU5	1,3	0,9	2,2	1,9	1,66	0,429
PAU7	0,3	0,5	0,1	0,1	0,21	0,412
PAU8	0,4	0,7	0,0	0,0	0,00	0,223

PAU9	16,3	6,5	9,2	6,6	0,56	0,175
PBA1	47,5	30,4	35,0	14,4	0,74	0,484
PBN1	98,7	46,3	129,4	60,4	1,31	0,451
PBP1	295,6	60,1	270,7	34,9	0,92	0,501
PBP2	37,3	23,3	22,4	19,1	0,60	0,360
PBS2	31,9	10,8	24,8	16,8	0,78	0,498
PBY1	44,0	25,6	32,6	25,0	0,74	0,546
PCA1	10,6	3,4	10,7	8,0	1,01	0,984
PCC1	117,2	48,6	101,5	28,3	0,87	0,596
PCD1	16,4	5,9	13,4	3,6	0,82	0,420
PCF11	24,2	11,2	12,0	8,2	0,49	0,128
PCH2	4,6	3,1	0,9	0,7	0,21	0,062
PCI8	17,4	12,3	23,4	7,9	1,35	0,444
PCK1	34,5	17,1	33,1	16,0	0,96	0,908
PCL1	99,9	29,6	4,1	3,4	0,04	0,001
PCL10	11,7	3,7	13,7	4,2	1,17	0,508
PCL2	70,3	31,9	97,2	68,2	1,38	0,502
PCL5	140,5	68,1	226,6	67,9	1,61	0,124
PCL6	86,0	13,6	114,7	45,0	1,33	0,268
PCL7	23,2	2,3	46,5	16,4	2,00	0,031
PCL8	17,3	12,3	11,0	9,6	0,64	0,454
PCL9	27,7	5,8	19,7	8,2	0,71	0,165
PCM1	77,3	69,9	104,5	80,8	1,35	0,629
PCP1	31,9	17,7	51,8	32,2	1,62	0,321
PCS60	23,8	5,9	101,6	39,4	4,27	0,008
PCT1	61,2	43,7	33,0	21,3	0,54	0,289
PDA1	856,2	140,9	1052,7	166,2	1,23	0,121
PDB1	725,6	475,5	746,3	456,9	1,03	0,952
PDC1	23692,4	6231,4	25923,1	6831,2	1,09	0,647
PDC2	37,9	8,0	34,0	12,2	0,90	0,613
PDC5	20,8	5,9	219,0	42,7	10,53	0,000
PDC6	1,3	1,0	3,5	3,4	2,75	0,254
PDE1	92,2	51,1	246,2	87,7	2,67	0,023
PDE2	30,1	13,2	22,5	16,6	0,75	0,503
PDH1	62,0	25,2	104,8	11,5	1,69	0,022
PDI1	205,0	104,9	284,9	109,1	1,39	0,332
PDR1	45,2	14,5	19,8	14,2	0,44	0,046
PDR10	27,0	10,3	17,1	13,1	0,63	0,278
PDR11	8,2	3,5	9,1	6,2	1,11	0,812
PDR12	15,9	7,7	21,4	22,2	1,34	0,657
PDR15	19,3	5,3	19,0	1,7	0,99	0,933
PDR16	193,5	69,5	102,7	36,3	0,53	0,060
PDR17	93,8	65,5	113,2	37,7	1,21	0,627
PDR18	1,3	1,2	3,2	2,3	2,40	0,203
PDR3	16,9	6,8	19,6	6,5	1,16	0,584
PDR5	134,2	52,6	63,8	1,9	0,48	0,037
PDR8	29,5	7,9	63,0	45,5	2,13	0,198
PDS1	35,9	11,8	6,2	4,3	0,17	0,003
PDS5	162,8	104,6	16,5	11,4	0,10	0,032
PDX1	66,8	32,1	110,6	70,7	1,66	0,302

PDX3	143,8	39,7	141,0	35,8	0,98	0,921
PEA2	45,0	13,5	24,3	9,1	0,54	0,045
PEF1	42,2	25,9	45,4	26,0	1,07	0,869
PEP1	73,8	38,7	106,7	45,0	1,45	0,310
PEP12	17,9	4,5	24,9	17,1	1,39	0,463
PEP3	25,3	13,4	42,7	11,0	1,68	0,092
PEP4	252,6	91,3	409,7	147,9	1,62	0,121
PEP5	25,6	10,0	23,4	6,0	0,91	0,721
PEP7	14,2	8,7	9,3	7,1	0,65	0,413
PEP8	39,3	28,7	48,6	39,6	1,24	0,718
PER1	192,3	88,4	222,0	68,3	1,15	0,614
PER33	337,0	191,5	631,8	358,7	1,87	0,197
PES4	1,1	0,7	12,7	20,2	11,53	0,294
PET100	182,7	43,4	381,6	83,9	2,09	0,006
PET111	4,5	2,6	5,4	3,9	1,21	0,698
PET112	17,6	10,0	23,0	10,7	1,31	0,488
PET117	37,9	15,1	47,8	13,4	1,26	0,366
PET122	0,0	0,0	0,0	0,0	#DIV/0!	#DIV/0!
PET123	73,4	32,0	46,1	3,1	0,63	0,140
PET127	15,7	7,4	8,7	6,0	0,56	0,194
PET130	39,2	11,7	56,7	21,0	1,45	0,197
PET18	11,3	2,9	6,5	4,7	0,58	0,138
PET191	104,6	52,4	52,0	25,3	0,50	0,120
PET20	16,3	3,7	38,8	13,8	2,38	0,020
PET309	42,9	20,2	32,8	16,0	0,76	0,461
PET494	30,3	7,0	34,7	4,7	1,14	0,344
PET54	13,5	4,1	6,2	4,4	0,46	0,052
PET8	50,3	11,9	60,4	32,6	1,20	0,583
PET9	285,1	224,0	226,7	72,0	0,80	0,637
PEX1	21,1	5,9	24,0	10,1	1,13	0,645
PEX10	12,0	6,6	12,0	8,6	1,00	0,999
PEX11	253,7	85,9	92,6	25,3	0,36	0,011
PEX12	27,2	25,0	22,6	24,2	0,83	0,799
PEX13	58,7	26,5	37,3	16,5	0,64	0,220
PEX14	36,9	12,2	52,4	17,2	1,42	0,190
PEX15	18,7	3,2	17,4	4,1	0,93	0,654
PEX17	31,8	8,6	31,5	7,4	0,99	0,962
PEX18	1,0	0,8	0,7	0,7	0,73	0,623
PEX19	57,9	36,6	61,8	41,4	1,07	0,892
PEX2	42,1	16,4	24,4	8,1	0,58	0,101
PEX21	19,6	8,8	23,8	21,6	1,21	0,735
PEX22	5,0	8,5	5,8	11,1	1,15	0,917
PEX25	72,2	38,9	25,3	20,2	0,35	0,075
PEX27	29,4	9,3	36,5	20,6	1,24	0,555
PEX28	26,3	18,1	32,6	13,2	1,24	0,591
PEX29	35,6	8,9	55,1	19,7	1,55	0,121
PEX3	27,2	16,5	34,5	21,1	1,27	0,603
PEX30	32,3	15,7	28,3	23,7	0,88	0,788
PEX31	31,9	17,3	18,5	9,3	0,58	0,220
PEX32	103,3	20,2	93,0	37,6	0,90	0,646

PEX4	29,2	20,0	37,7	20,0	1,29	0,568
PEX5	50,4	25,9	19,6	13,9	0,39	0,081
PEX6	26,6	17,6	25,4	11,8	0,96	0,914
PEX7	27,1	16,2	35,3	23,4	1,30	0,584
PEX8	25,7	12,9	32,8	10,4	1,28	0,420
PFA3	47,4	16,3	43,7	20,2	0,92	0,784
PFA4	62,4	9,9	86,0	49,7	1,38	0,388
PFA5	75,9	16,4	47,7	21,8	0,63	0,084
PFD1	63,3	57,9	40,2	37,0	0,64	0,526
PFK1	308,5	158,4	457,8	194,7	1,48	0,279
PFK2	850,1	162,1	1338,0	211,9	1,57	0,011
PFK26	62,4	23,7	50,9	16,1	0,82	0,454
PFK27	34,5	15,7	54,2	30,5	1,57	0,295
PFS1	2,3	1,9	0,5	0,6	0,21	0,112
PFS2	10,3	10,9	4,8	3,6	0,46	0,372
PFY1	1230,9	695,0	1161,3	806,4	0,94	0,900
PGA1	1,0	0,3	1,0	0,9	1,01	0,980
PGA2	183,0	80,8	318,5	117,5	1,74	0,106
PGA3	292,3	133,8	206,7	52,6	0,71	0,279
PGC1	116,1	56,5	53,7	29,2	0,46	0,098
PGD1	37,9	14,5	55,1	49,0	1,45	0,527
PGI1	3664,8	1943,8	3003,1	892,4	0,82	0,559
PGK1	8705,4	4854,0	9568,9	3047,8	1,10	0,773
PGM1	70,5	48,9	16,0	16,5	0,23	0,079
PGM2	16,0	12,8	41,8	32,1	2,60	0,186
PGM3	23,9	9,5	73,1	17,3	3,06	0,003
PGS1	24,5	15,4	27,2	19,2	1,11	0,829
PGU1	0,3	0,3	0,6	0,8	2,36	0,426
PHA2	33,2	15,1	23,3	12,8	0,70	0,356
PHB1	51,9	28,1	115,7	60,9	2,23	0,106
PHB2	173,9	64,9	231,6	33,2	1,33	0,165
PHD1	41,0	20,5	75,1	33,3	1,83	0,131
PHM6	11,3	5,7	66,4	25,4	5,88	0,005
PHM7	4,1	2,3	2,2	1,9	0,54	0,254
PHM8	16,2	8,6	82,2	64,1	5,08	0,087
PHO11	13,4	13,0	114,3	75,0	8,50	0,038
PHO12	0,1	0,1	0,4	0,6	6,08	0,313
PHO13	181,1	55,7	97,1	27,0	0,54	0,035
PHO2	29,4	19,4	40,3	11,8	1,37	0,374
PHO23	23,0	5,8	39,3	15,1	1,71	0,089
PHO3	66,1	40,7	64,4	6,6	0,97	0,933
PHO4	19,9	17,2	22,8	17,7	1,14	0,823
PHO5	3,7	4,2	102,2	58,2	27,82	0,015
PHO8	31,4	14,5	260,3	147,3	8,30	0,021
PHO80	20,9	11,1	12,1	9,1	0,58	0,266
PHO81	34,5	28,1	138,4	83,0	4,01	0,055
PHO84	50,1	27,2	516,1	214,4	10,29	0,005
PHO85	74,0	47,4	69,4	28,3	0,94	0,874
PHO86	220,9	96,8	311,3	30,9	1,41	0,126
PHO87	46,3	26,7	65,5	40,3	1,41	0,458

PHO88	876,2	458,6	746,8	407,6	0,85	0,688
PHO89	15,9	18,6	29,0	13,0	1,83	0,290
PHO90	95,7	33,3	81,2	32,3	0,85	0,556
PHO91	49,1	22,8	56,0	8,4	1,14	0,591
PHR1	4,2	2,9	16,9	13,6	4,05	0,118
PHS1	107,3	19,2	63,3	4,9	0,59	0,004
PIB1	18,1	13,7	30,4	21,1	1,68	0,367
PIB2	64,0	13,7	48,9	18,8	0,76	0,244
PIC2	68,2	18,9	90,7	29,5	1,33	0,246
PIF1	31,0	10,3	14,8	2,7	0,48	0,022
PIG1	13,2	8,1	6,3	4,4	0,48	0,187
PIG2	14,5	1,8	26,7	11,1	1,84	0,073
PIH1	38,0	10,1	46,2	29,0	1,22	0,609
PIK1	52,1	10,8	54,1	8,5	1,04	0,775
PIL1	334,9	105,9	227,0	41,5	0,68	0,107
PIM1	129,8	87,9	121,3	67,6	0,94	0,884
PIN2	12,2	8,3	13,1	10,5	1,07	0,903
PIN4	86,1	39,4	101,3	36,6	1,18	0,591
PIP2	21,7	8,6	62,7	44,0	2,89	0,117
PIR1	348,1	149,1	259,8	111,8	0,75	0,380
PIR3	2,6	2,7	3,1	2,2	1,21	0,767
PIS1	303,5	128,8	376,5	105,0	1,24	0,414
PKC1	28,5	17,6	27,5	4,3	0,96	0,911
PKH1	63,8	9,4	57,2	10,7	0,90	0,390
PKH2	15,3	7,6	17,9	13,2	1,17	0,743
PKH3	59,1	22,5	84,9	19,1	1,44	0,131
PKP1	22,5	10,4	27,1	15,4	1,21	0,637
PKP2	34,8	15,3	56,8	19,1	1,63	0,123
PLB1	13,1	8,9	23,1	18,4	1,77	0,363
PLB2	27,0	19,5	2,9	2,2	0,11	0,050
PLB3	79,6	33,4	42,9	50,5	0,54	0,271
PLC1	47,1	8,7	51,8	22,8	1,10	0,713
PLM2	13,0	7,7	2,8	3,0	0,21	0,047
PLP1	52,5	17,5	31,8	7,0	0,61	0,070
PLP2	16,7	8,2	24,1	8,4	1,45	0,252
PMA1	2049,1	923,3	2488,3	1255,9	1,21	0,593
PMA2	0,6	0,8	0,0	0,0	0,00	0,188
PMC1	41,1	12,6	58,5	26,6	1,42	0,281
PMD1	35,3	22,6	48,1	12,6	1,36	0,361
PMI40	360,5	96,4	39,8	28,8	0,11	0,001
PML1	32,1	14,1	28,6	10,9	0,89	0,713
PML39	14,9	6,2	19,3	8,5	1,29	0,436
PMP1	93,9	60,1	250,0	105,8	2,66	0,043
PMP3	1519,0	1169,1	2092,1	1586,1	1,38	0,582
PMR1	398,8	269,5	166,2	77,6	0,42	0,148
PMS1	56,6	20,5	9,9	7,2	0,17	0,005
PMT1	178,7	65,5	67,6	13,9	0,38	0,016
PMT2	196,8	52,9	121,9	17,8	0,62	0,036
PMT3	77,7	34,3	34,9	24,3	0,45	0,088
PMT4	133,9	71,7	46,5	12,8	0,35	0,053

PMT5	68,5	44,2	17,1	13,3	0,25	0,068
PMT6	113,9	63,4	93,4	24,2	0,82	0,568
PMU1	173,4	75,4	174,9	38,6	1,01	0,973
PNC1	120,4	82,7	549,1	475,4	4,56	0,126
PNG1	45,5	5,3	61,3	31,5	1,35	0,359
PNO1	53,1	12,6	52,1	21,2	0,98	0,938
PNP1	34,6	18,7	14,5	11,4	0,42	0,116
PNS1	75,4	51,4	45,0	27,7	0,60	0,339
PNT1	29,8	12,1	17,3	3,5	0,58	0,095
POA1	42,7	19,9	45,5	42,5	1,07	0,908
POB3	103,2	30,3	79,3	15,0	0,77	0,206
POC4	55,5	26,1	20,2	16,3	0,36	0,061
POG1	18,2	3,4	41,4	8,7	2,27	0,003
POL1	104,0	56,5	30,3	14,4	0,29	0,045
POL12	26,9	6,3	7,2	6,1	0,27	0,004
POL2	325,3	161,4	387,5	169,4	1,19	0,614
POL3	151,1	22,3	79,9	3,2	0,53	0,001
POL30	165,6	45,7	5,7	4,0	0,03	0,000
POL31	116,5	89,0	31,7	15,0	0,27	0,109
POL32	50,4	21,1	30,5	7,4	0,61	0,125
POL4	29,3	19,7	14,7	10,1	0,50	0,234
POL5	158,7	65,1	115,7	17,4	0,73	0,249
POM152	21,7	9,5	20,9	14,7	0,96	0,923
POM33	56,6	10,4	96,8	24,8	1,71	0,024
POM34	85,0	44,2	64,7	43,4	0,76	0,536
POP1	66,5	33,6	61,0	39,0	0,92	0,840
POP2	111,7	58,8	65,8	40,8	0,59	0,247
POP3	3,6	2,2	5,3	4,3	1,44	0,528
POP4	26,0	4,1	22,8	15,7	0,88	0,710
POP5	84,1	35,3	31,9	19,2	0,38	0,041
POP6	63,0	45,7	56,4	21,2	0,90	0,802
POP7	35,9	24,3	49,2	37,2	1,37	0,571
POP8	168,7	106,8	89,1	15,3	0,53	0,191
POR1	837,3	157,4	2897,8	243,6	3,46	0,000
POS5	7,7	4,9	24,9	11,7	3,23	0,035
POT1	1,6	1,0	0,5	0,4	0,32	0,094
POX1	0,7	1,1	2,3	1,6	3,34	0,153
PPA2	36,4	29,8	22,2	17,0	0,61	0,442
PPE1	16,5	8,9	14,7	4,5	0,89	0,739
PPG1	71,5	17,7	100,6	19,0	1,41	0,066
PPH21	156,5	51,5	174,7	95,2	1,12	0,749
PPH22	59,2	33,0	25,5	17,0	0,43	0,119
PPH3	20,4	14,4	18,4	16,5	0,90	0,861
PPM1	75,1	25,8	60,9	30,9	0,81	0,507
PPM2	0,0	0,0	0,0	0,1	#DIV/0!	0,356
PPN1	238,2	56,3	311,4	71,4	1,31	0,158
PPQ1	184,1	95,4	79,4	13,8	0,43	0,073
PPR1	32,2	15,4	27,8	10,9	0,86	0,659
PPS1	84,4	26,6	57,5	11,5	0,68	0,114
PPT1	70,1	11,7	69,4	7,0	0,99	0,923

PPT2	12,9	10,6	7,9	6,5	0,61	0,453
PPX1	62,5	14,0	63,5	41,5	1,02	0,962
PPZ1	53,5	11,8	37,8	14,8	0,71	0,148
PPZ2	54,0	26,1	87,4	39,3	1,62	0,206
PRB1	68,1	27,4	209,3	15,3	3,07	0,000
PRC1	376,8	168,2	570,3	194,0	1,51	0,182
PRD1	58,5	26,3	67,9	22,7	1,16	0,609
PRE1	98,2	15,5	106,1	37,0	1,08	0,709
PRE10	236,1	43,3	233,7	11,2	0,99	0,919
PRE2	367,5	41,6	342,3	203,9	0,93	0,817
PRE3	301,0	138,0	380,0	91,5	1,26	0,377
PRE4	66,9	88,3	120,4	144,2	1,80	0,550
PRE5	72,9	41,6	72,9	44,4	1,00	0,998
PRE6	284,4	171,7	470,0	217,8	1,65	0,229
PRE7	257,9	129,5	243,9	133,2	0,95	0,885
PRE8	102,6	32,6	126,2	27,9	1,23	0,313
PRE9	298,9	153,1	384,8	171,4	1,29	0,483
PRI1	60,5	26,0	30,2	21,2	0,50	0,120
PRI2	50,4	24,3	11,7	10,1	0,23	0,026
PRK1	17,3	9,1	16,9	3,5	0,97	0,928
PRM1	4,6	3,1	20,4	17,1	4,44	0,120
PRM10	14,3	9,8	39,8	13,1	2,79	0,021
PRM2	8,2	4,3	36,2	16,9	4,41	0,018
PRM3	4,5	2,1	180,8	139,1	40,07	0,044
PRM4	22,8	13,5	59,2	27,9	2,59	0,058
PRM5	61,0	97,3	131,1	177,5	2,15	0,515
PRM6	2,3	2,0	60,6	55,8	26,07	0,082
PRM7	25,2	24,1	28,4	12,3	1,13	0,819
PRM8	20,2	9,2	46,5	28,2	2,30	0,127
PRM9	18,5	9,6	27,7	15,0	1,49	0,344
PRO1	107,3	47,5	58,4	22,2	0,54	0,111
PRO2	205,1	93,4	269,4	139,1	1,31	0,472
PRO3	211,3	121,6	173,2	115,4	0,82	0,665
PRP11	6,8	2,7	21,9	13,2	3,20	0,067
PRP16	67,8	9,9	84,3	47,1	1,24	0,518
PRP18	1,3	0,3	12,7	6,9	9,71	0,017
PRP19	63,0	26,3	34,9	24,3	0,55	0,168
PRP2	13,1	3,4	18,7	6,3	1,43	0,170
PRP21	43,8	5,5	29,0	5,4	0,66	0,009
PRP22	23,2	11,9	18,1	3,1	0,78	0,434
PRP24	9,8	1,8	8,8	6,1	0,90	0,757
PRP28	10,8	2,6	23,6	20,7	2,17	0,268
PRP3	36,2	17,9	50,0	22,9	1,38	0,380
PRP31	17,5	7,6	36,3	33,9	2,07	0,320
PRP38	5,9	4,1	5,7	6,3	0,97	0,961
PRP39	11,1	5,3	15,3	10,4	1,38	0,498
PRP4	30,2	12,7	33,7	24,3	1,12	0,805
PRP40	215,7	46,6	301,4	91,0	1,40	0,145
PRP42	26,4	3,6	24,7	6,1	0,94	0,652
PRP43	72,4	66,3	86,8	71,1	1,20	0,778

PRP45	12,0	6,2	9,2	6,9	0,77	0,572
PRP46	33,4	20,1	40,9	20,9	1,22	0,623
PRP5	18,5	4,9	46,5	42,5	2,51	0,239
PRP6	38,7	15,0	41,1	22,4	1,06	0,868
PRP8	60,1	22,9	85,6	27,6	1,42	0,205
PRP9	20,6	10,6	30,9	18,2	1,50	0,367
PRR1	38,1	16,5	20,0	18,0	0,53	0,188
PRR2	3,4	2,2	3,6	2,8	1,07	0,898
PRS1	103,7	64,0	52,4	75,3	0,51	0,339
PRS2	124,8	76,2	113,2	50,3	0,91	0,808
PRS3	155,3	115,9	226,2	96,2	1,46	0,383
PRS4	124,0	71,9	173,7	55,6	1,40	0,316
PRS5	133,4	90,5	110,9	43,5	0,83	0,671
PRT1	315,6	201,9	295,4	214,9	0,94	0,895
PRX1	63,2	16,1	314,4	147,2	4,98	0,015
PRY1	247,2	131,1	99,0	16,0	0,40	0,066
PRY2	177,6	76,9	140,1	59,8	0,79	0,470
PRY3	56,0	43,8	44,5	42,8	0,80	0,722
PSA1	3686,1	1105,8	952,8	239,4	0,26	0,003
PSD1	40,8	7,6	21,5	4,4	0,53	0,005
PSD2	27,4	15,4	40,6	10,0	1,48	0,200
PSE1	234,6	191,2	121,6	85,1	0,52	0,322
PSF1	26,9	10,6	10,9	7,9	0,41	0,052
PSF2	77,3	23,7	52,7	13,6	0,68	0,122
PSF3	40,4	24,5	16,1	9,7	0,40	0,115
PSH1	36,4	15,5	25,7	6,4	0,70	0,245
PSK1	37,8	21,2	45,2	14,2	1,19	0,584
PSK2	95,5	46,4	81,0	44,6	0,85	0,670
PSO2	11,0	6,6	9,4	9,2	0,85	0,782
PSP1	23,9	17,7	25,7	19,3	1,08	0,895
PSP2	11,6	7,4	22,1	10,1	1,91	0,145
PSR1	103,4	58,8	110,4	52,2	1,07	0,864
PSR2	48,3	24,5	27,4	14,9	0,57	0,195
PST1	117,9	47,1	304,6	124,5	2,58	0,031
PST2	584,7	380,9	589,2	260,6	1,01	0,985
PSY2	28,2	11,6	44,3	15,0	1,57	0,138
PSY3	20,3	9,8	4,7	3,1	0,23	0,023
PSY4	37,1	18,2	16,7	12,0	0,45	0,111
PTA1	49,7	9,8	31,1	20,8	0,63	0,157
PTC1	23,9	10,2	24,5	8,8	1,03	0,931
PTC2	93,0	41,5	75,5	40,0	0,81	0,567
PTC3	185,3	31,2	199,6	29,6	1,08	0,530
PTC4	31,5	7,5	46,5	24,4	1,48	0,284
PTC5	15,8	8,8	18,6	8,3	1,18	0,651
PTC6	7,7	4,0	6,9	4,7	0,90	0,801
PTC7	77,0	55,1	74,4	44,0	0,97	0,945
PTH1	0,9	0,4	0,9	0,7	1,05	0,921
PTH2	58,5	59,9	31,5	20,6	0,54	0,426
PTI1	65,3	18,0	44,0	39,8	0,67	0,368
PTK1	6,2	3,3	12,2	10,2	1,96	0,304

PTK2	100,4	78,4	266,7	229,2	2,66	0,219
PTM1	70,8	36,7	80,0	23,8	1,13	0,688
PTP1	26,3	3,5	37,4	4,8	1,42	0,009
PTP2	25,9	4,5	26,3	7,4	1,02	0,929
PTP3	31,2	10,8	54,3	41,0	1,74	0,317
PTR2	29,5	6,1	9,7	6,9	0,33	0,005
PTR3	22,4	12,6	19,0	4,1	0,85	0,621
PUB1	406,1	122,6	414,8	143,1	1,02	0,929
PUF2	13,8	4,8	28,0	18,7	2,02	0,193
PUF3	2,6	0,9	5,4	1,6	2,06	0,024
PUF4	73,1	29,6	104,1	35,8	1,42	0,230
PUF6	106,0	57,6	111,1	39,1	1,05	0,888
PUG1	5,9	2,8	2,8	3,0	0,47	0,178
PUN1	70,9	24,2	109,1	54,6	1,54	0,248
PUP1	199,3	46,6	226,6	35,6	1,14	0,387
PUP2	305,6	169,0	422,9	225,4	1,38	0,437
PUP3	268,4	205,6	242,7	170,1	0,90	0,853
PUS1	53,9	22,8	40,9	12,8	0,76	0,358
PUS2	1,8	1,7	4,7	5,3	2,58	0,340
PUS4	73,1	20,3	51,1	14,5	0,70	0,129
PUS5	16,2	6,3	16,5	11,7	1,02	0,969
PUS6	25,1	25,0	13,8	12,0	0,55	0,448
PUS7	46,2	26,0	52,7	22,3	1,14	0,715
PUS9	26,2	6,7	23,4	16,6	0,89	0,761
PUT1	7,6	3,9	12,9	2,0	1,70	0,052
PUT2	95,8	55,2	163,5	152,3	1,71	0,435
PUT3	9,8	7,5	17,1	15,5	1,74	0,432
PUT4	10,9	3,4	1,6	1,8	0,15	0,003
PWP1	156,5	46,1	194,7	58,5	1,24	0,345
PWP2	123,1	37,8	150,2	39,0	1,22	0,355
PXA1	5,5	1,9	7,2	4,8	1,30	0,548
PXA2	13,5	5,2	30,6	24,4	2,27	0,218
PXL1	30,8	5,3	10,6	8,0	0,34	0,006
PXR1	203,9	118,3	188,6	158,6	0,92	0,882
PYC1	203,9	130,8	765,1	490,7	3,75	0,069
PYC2	368,8	124,1	210,1	36,4	0,57	0,050
PYK2	45,8	12,2	83,2	15,9	1,82	0,010
PZF1	16,9	6,5	19,3	10,0	1,15	0,694
Q0144	1,0	1,4	1,9	3,6	1,94	0,648
Q0255	2,0	2,6	0,3	0,6	0,15	0,264
QCR10	111,4	14,5	277,1	103,6	2,49	0,019
QCR2	157,2	86,3	268,9	119,8	1,71	0,181
QCR6	112,6	53,2	205,6	74,3	1,83	0,088
QCR7	260,2	188,0	387,6	246,4	1,49	0,443
QCR8	399,8	190,1	762,8	427,5	1,91	0,172
QCR9	178,2	40,2	221,2	105,4	1,24	0,475
QDR1	3,0	3,1	0,8	0,7	0,26	0,218
QDR2	114,3	68,5	77,1	42,5	0,67	0,391
QDR3	126,0	63,5	43,2	16,5	0,34	0,045
QNS1	79,3	59,3	54,1	26,0	0,68	0,465

QRI1	206,4	78,3	146,0	37,4	0,71	0,214
QRI5	126,9	56,3	242,5	177,4	1,91	0,261
QRI7	19,1	9,3	45,6	10,9	2,39	0,010
RAD1	49,7	21,6	42,1	20,9	0,85	0,633
RAD10	7,5	3,4	8,1	5,8	1,08	0,866
RAD14	16,3	5,0	23,7	6,6	1,46	0,121
RAD16	42,0	11,6	50,6	14,0	1,20	0,383
RAD17	522,8	449,2	324,9	347,3	0,62	0,512
RAD18	10,7	8,5	12,1	10,0	1,13	0,835
RAD2	18,2	9,4	38,5	19,9	2,12	0,114
RAD23	144,7	52,2	194,4	52,4	1,34	0,227
RAD24	28,5	8,5	16,4	11,1	0,58	0,135
RAD26	21,8	7,8	41,9	15,1	1,92	0,055
RAD27	68,6	42,4	14,6	11,4	0,21	0,049
RAD28	10,7	4,5	19,3	9,1	1,80	0,142
RAD3	30,8	9,1	18,1	12,4	0,59	0,150
RAD30	9,6	3,9	10,3	8,4	1,08	0,877
RAD33	86,7	67,7	39,6	16,8	0,46	0,226
RAD34	2,6	2,0	7,7	5,8	3,02	0,142
RAD4	8,6	2,1	11,4	5,5	1,32	0,378
RAD5	34,9	14,0	16,1	6,6	0,46	0,052
RAD50	25,8	13,9	51,3	15,9	1,99	0,052
RAD51	27,1	6,3	13,8	9,4	0,51	0,058
RAD52	41,5	16,6	41,1	8,9	0,99	0,965
RAD53	25,2	14,3	3,5	2,9	0,14	0,024
RAD54	48,4	30,8	56,4	28,7	1,16	0,718
RAD57	23,3	9,3	19,8	10,5	0,85	0,634
RAD59	32,6	17,6	44,1	30,1	1,35	0,536
RAD6	110,5	59,7	153,7	6,0	1,39	0,200
RAD61	14,6	5,4	24,8	12,8	1,70	0,194
RAD7	11,8	0,6	22,3	15,4	1,89	0,223
RAD9	38,8	10,1	32,3	9,0	0,83	0,377
RAI1	279,3	229,4	311,6	191,0	1,12	0,836
RAM1	39,5	15,2	36,1	17,6	0,91	0,779
RAM2	156,2	101,9	126,1	97,4	0,81	0,684
RAP1	53,6	9,3	15,6	4,7	0,29	0,000
RAS1	61,0	28,4	65,3	37,0	1,07	0,858
RAS2	182,2	37,3	252,2	28,7	1,38	0,025
RAT1	54,5	33,8	59,0	46,9	1,08	0,881
RAV1	30,8	15,3	39,7	16,9	1,29	0,467
RAV2	26,4	11,0	32,2	8,2	1,22	0,429
RAX1	2,0	1,5	3,0	2,1	1,49	0,466
RAX2	15,0	2,9	17,1	11,5	1,14	0,739
RBA50	120,3	68,9	58,7	7,0	0,49	0,125
RBD2	193,8	92,2	171,2	85,4	0,88	0,732
RBG1	293,4	162,8	354,2	175,0	1,21	0,629
RBG2	143,6	35,4	104,5	16,1	0,73	0,091
RBK1	120,3	33,4	175,0	48,9	1,45	0,114
RBL2	100,7	8,5	105,8	69,5	1,05	0,888
RBS1	37,7	14,9	30,6	6,0	0,81	0,407

RCE1	28,8	23,5	10,1	11,1	0,35	0,202
RCK1	1,3	0,7	4,2	3,7	3,29	0,165
RCK2	43,0	3,4	59,3	33,9	1,38	0,377
RCL1	82,1	30,3	65,5	24,2	0,80	0,428
RCN1	19,3	11,1	33,0	25,4	1,71	0,360
RCN2	44,0	21,6	55,2	2,9	1,25	0,344
RCO1	38,9	30,7	41,5	33,2	1,07	0,913
RCR1	14,2	7,0	25,8	19,1	1,81	0,299
RCR2	38,2	15,8	58,1	23,3	1,52	0,206
RCY1	17,0	13,8	12,0	9,4	0,70	0,567
RDH54	108,9	23,5	35,8	13,9	0,33	0,002
RDI1	115,2	43,5	126,2	13,1	1,10	0,645
RDL1	358,8	137,1	573,9	60,7	1,60	0,028
RDR1	5,0	2,3	3,5	2,3	0,69	0,376
RDS1	3,5	1,1	4,2	3,6	1,21	0,719
RDS2	20,0	9,1	12,6	11,5	0,63	0,352
RDS3	36,9	21,5	33,3	5,8	0,90	0,755
REB1	4,3	2,7	5,7	5,0	1,33	0,638
REC102	2,4	1,4	1,3	1,1	0,53	0,247
REC104	9,9	3,5	6,8	4,9	0,69	0,341
REC107	9,8	12,8	4,3	4,1	0,43	0,437
REC114	4,2	2,7	1,5	1,1	0,36	0,110
REC8	2,8	1,3	2,6	2,3	0,96	0,933
RED1	21,2	10,2	20,8	14,5	0,98	0,967
REE1	70,9	32,4	767,1	378,8	10,81	0,011
REF2	97,9	26,4	120,7	29,0	1,23	0,287
REG1	63,6	54,8	56,3	48,5	0,89	0,848
REG2	1,9	0,5	2,7	2,0	1,40	0,476
REH1	85,5	19,8	204,6	46,6	2,39	0,003
REI1	82,6	9,1	43,1	11,3	0,52	0,002
REP1	199,8	131,3	141,2	84,7	0,71	0,481
REP2	246,3	135,5	234,8	55,3	0,95	0,880
RER1	0,5	0,2	0,0	0,0	0,00	0,001
RER2	65,7	63,0	81,4	51,0	1,24	0,712
RET1	68,0	18,2	70,6	33,8	1,04	0,898
RET2	130,7	52,9	91,6	52,3	0,70	0,334
RET3	142,3	85,1	24,7	5,0	0,17	0,033
REV1	137,1	79,1	107,0	59,6	0,78	0,565
REV3	17,4	7,0	39,5	17,5	2,27	0,057
REV7	16,0	9,5	12,9	12,1	0,80	0,695
REX2	148,0	31,2	180,0	70,6	1,22	0,439
REX3	95,3	37,9	88,8	21,7	0,93	0,778
REX4	36,9	19,6	16,6	14,2	0,45	0,145
RFA1	27,5	21,1	5,4	4,0	0,20	0,085
RFA2	27,5	11,7	4,6	3,3	0,17	0,009
RFC1	28,6	13,0	21,5	7,0	0,75	0,375
RFC2	30,0	13,4	28,8	7,3	0,96	0,880
RFC3	145,8	48,3	80,6	59,5	0,55	0,140
RFC4	26,7	8,2	32,3	16,0	1,21	0,555
RFC5	18,5	14,3	8,2	7,7	0,45	0,253

RFM1	15,0	6,3	19,7	18,5	1,32	0,646
RFS1	109,3	59,3	182,4	83,3	1,67	0,203
RFT1	37,6	1,0	29,5	9,2	0,79	0,132
RFU1	17,5	8,1	8,8	6,6	0,50	0,145
RFX1	23,9	9,5	26,9	10,9	1,12	0,696
RGA1	20,2	14,9	26,2	12,2	1,30	0,553
RGA2	37,3	11,6	33,8	22,8	0,90	0,791
RGC1	13,7	9,5	18,0	6,7	1,32	0,484
RGD1	35,4	17,9	29,0	6,9	0,82	0,528
RGD2	26,3	15,3	37,2	22,6	1,41	0,455
RGI1	43,0	31,9	373,1	239,8	8,69	0,034
RGI2	1,5	1,9	1,1	0,7	0,71	0,679
RGM1	7,1	6,8	8,1	4,7	1,14	0,814
RGP1	35,1	17,0	24,7	6,9	0,70	0,297
RGR1	39,8	11,9	48,4	24,2	1,22	0,547
RGS2	0,9	0,5	3,1	3,2	3,45	0,228
RGT1	44,7	24,2	56,9	23,1	1,27	0,492
RGT2	36,4	21,9	38,3	10,6	1,05	0,883
RHB1	5,7	1,7	11,7	8,1	2,05	0,200
RHO1	399,2	196,8	310,1	142,8	0,78	0,492
RHO2	164,5	72,4	90,3	57,8	0,55	0,160
RHO3	149,0	90,0	43,6	31,6	0,29	0,069
RHO4	57,4	28,7	35,1	18,6	0,61	0,240
RHO5	482,6	426,4	381,3	310,0	0,79	0,714
RHR2	3183,0	1744,7	3450,4	1396,9	1,08	0,819
RIA1	81,5	39,5	48,4	25,6	0,59	0,210
RIB1	18,5	1,3	44,1	6,1	2,38	0,000
RIB2	182,3	113,7	284,2	171,7	1,56	0,360
RIB3	538,0	234,4	546,2	200,9	1,02	0,959
RIB4	565,2	173,6	1117,7	523,0	1,98	0,092
RIB5	218,2	159,3	206,1	158,5	0,94	0,918
RIB7	28,9	27,6	26,3	6,3	0,91	0,863
RIC1	42,7	18,0	72,2	25,9	1,69	0,111
RIF1	23,0	8,8	9,9	3,7	0,43	0,034
RIF2	9,5	3,6	6,9	4,7	0,73	0,411
RIM1	316,0	114,4	351,4	126,4	1,11	0,692
RIM101	17,5	4,0	57,8	22,9	3,31	0,013
RIM11	85,5	52,3	110,7	30,1	1,30	0,434
RIM13	16,8	11,6	19,5	21,1	1,16	0,826
RIM15	37,3	20,6	38,2	9,5	1,02	0,941
RIM2	62,1	25,1	57,2	10,1	0,92	0,729
RIM20	49,3	22,5	74,0	10,3	1,50	0,093
RIM21	92,0	18,7	158,6	14,0	1,72	0,001
RIM4	3,4	2,0	1,8	1,2	0,54	0,237
RIM8	53,5	11,8	51,6	9,9	0,96	0,811
RIM9	0,5	0,5	0,9	0,6	1,76	0,365
RIO1	44,0	33,4	66,3	53,4	1,51	0,506
RIO2	137,0	48,2	65,9	43,4	0,48	0,071
RIP1	6,5	5,0	11,8	9,7	1,81	0,370
RIT1	18,9	3,0	23,7	5,3	1,25	0,167

RIX1	22,9	8,6	26,5	10,4	1,15	0,622
RIX7	105,9	40,9	40,2	27,4	0,38	0,037
RKI1	191,6	112,8	144,6	49,9	0,75	0,475
RKM1	62,6	31,2	8,5	7,3	0,14	0,015
RKM2	62,2	4,4	28,0	9,6	0,45	0,001
RKM3	65,2	21,8	49,5	14,7	0,76	0,279
RKM4	35,7	1,8	51,9	7,5	1,45	0,006
RKR1	80,8	19,1	55,3	27,2	0,68	0,175
RLF2	85,3	42,7	49,4	24,0	0,58	0,193
RLI1	160,2	33,7	129,4	15,2	0,81	0,146
RLM1	30,1	26,8	47,7	41,8	1,58	0,505
RLP24	189,2	52,9	166,9	64,6	0,88	0,612
RLP7	191,9	52,5	180,7	29,0	0,94	0,722
RMA1	20,3	8,1	17,8	6,7	0,88	0,650
RMD1	43,4	10,1	34,9	12,7	0,80	0,332
RMD5	7,6	1,8	26,7	23,6	3,50	0,158
RMD6	3,0	1,5	6,6	5,6	2,23	0,252
RMD8	36,6	13,0	35,5	6,6	0,97	0,881
RMD9	84,6	26,9	91,8	31,4	1,08	0,742
RME1	235,9	75,6	356,1	126,4	1,51	0,154
RMI1	57,8	19,0	19,7	14,9	0,34	0,020
RML2	206,8	96,0	209,1	73,2	1,01	0,971
RMR1	16,0	6,3	19,9	16,2	1,24	0,673
RMT2	51,8	38,3	27,3	16,3	0,53	0,283
RNA1	328,9	180,8	247,7	64,2	0,75	0,430
RNA14	85,6	23,5	59,5	26,7	0,69	0,191
RNA15	29,5	9,5	25,5	10,0	0,87	0,586
RNH1	32,4	24,8	20,0	16,1	0,62	0,432
RNH201	103,5	18,1	32,5	16,1	0,31	0,001
RNH202	26,4	9,8	16,9	11,8	0,64	0,262
RNH203	54,0	31,2	17,3	11,2	0,32	0,069
RNH70	32,8	17,9	17,7	12,1	0,54	0,212
RNP1	0,3	0,4	12,8	8,3	40,96	0,024
RNQ1	150,0	50,2	132,6	9,9	0,88	0,520
RNR1	146,4	115,3	2,6	2,2	0,02	0,047
RNR2	381,5	119,8	379,1	145,8	0,99	0,980
RNR3	18,5	11,0	25,0	21,8	1,35	0,614
RNR4	569,0	333,3	773,9	436,2	1,36	0,483
RNT1	66,4	23,3	56,9	26,5	0,86	0,612
RNY1	13,0	4,0	39,0	6,9	2,99	0,001
ROD1	15,4	7,9	26,8	6,0	1,73	0,063
ROG1	20,6	3,0	18,1	5,6	0,88	0,466
ROG3	23,3	7,5	46,4	24,1	1,99	0,117
ROK1	155,7	33,3	120,6	66,3	0,77	0,381
ROM1	26,4	10,3	67,0	7,2	2,54	0,001
ROM2	33,1	20,2	37,1	27,7	1,12	0,825
ROT1	133,3	49,7	119,5	31,0	0,90	0,653
ROT2	60,1	38,9	57,1	25,2	0,95	0,904
ROX1	10,8	13,5	10,6	14,8	0,98	0,984
ROX3	119,5	48,7	96,7	65,8	0,81	0,599

RPA12	194,8	79,8	148,4	39,4	0,76	0,337
RPA135	100,8	19,4	81,2	7,9	0,81	0,110
RPA14	40,0	17,0	49,8	7,4	1,25	0,332
RPA190	477,2	266,0	343,9	134,3	0,72	0,405
RPA34	213,4	60,7	181,1	60,1	0,85	0,478
RPA43	176,0	90,4	85,6	22,9	0,49	0,101
RPB10	156,6	81,8	183,7	135,8	1,17	0,745
RPB2	144,7	17,6	113,0	16,9	0,78	0,040
RPB3	278,9	19,4	261,8	34,8	0,94	0,423
RPB4	199,3	82,5	200,3	72,1	1,00	0,986
RPB5	229,5	81,5	256,8	98,3	1,12	0,685
RPB7	306,4	67,3	228,1	13,9	0,74	0,063
RPB8	430,9	338,9	490,7	302,3	1,14	0,801
RPB9	95,4	59,1	87,8	56,9	0,92	0,860
RPC11	222,7	71,7	101,7	41,5	0,46	0,027
RPC17	142,4	61,7	80,2	22,1	0,56	0,107
RPC19	202,4	104,2	185,2	52,7	0,92	0,779
RPC25	51,2	45,5	29,9	17,0	0,58	0,413
RPC31	91,5	24,2	70,7	21,7	0,77	0,247
RPC34	70,5	37,9	64,1	48,0	0,91	0,840
RPC37	126,1	99,4	139,1	50,0	1,10	0,823
RPC40	219,0	135,4	165,4	58,0	0,76	0,494
RPC53	49,8	8,6	52,3	7,1	1,05	0,666
RPC82	48,1	22,2	36,0	18,1	0,75	0,429
RPD3	70,5	40,8	81,4	25,7	1,16	0,666
RPE1	150,5	123,6	89,6	98,4	0,60	0,470
RPF1	52,6	32,7	48,9	10,7	0,93	0,838
RPF2	61,1	51,6	27,6	22,7	0,45	0,280
RPG1	229,5	84,1	200,0	69,6	0,87	0,607
RPH1	62,9	24,1	134,1	38,1	2,13	0,020
RPI1	30,4	12,8	35,2	12,4	1,16	0,607
RPL10	3145,1	1832,8	1531,3	1174,2	0,49	0,189
RPL11A	4,8	2,9	8,9	7,4	1,87	0,338
RPL11B	2135,5	2320,2	1192,8	1154,3	0,56	0,494
RPL12A	605,0	173,3	652,0	248,8	1,08	0,767
RPL12B	1954,7	1363,6	1460,1	1080,9	0,75	0,590
RPL13A	9,6	6,0	2,8	2,8	0,29	0,085
RPL13B	9,6	4,2	5,1	3,9	0,54	0,177
RPL14A	3601,7	1161,1	1743,4	1046,4	0,48	0,055
RPL14B	13,3	7,5	10,5	13,5	0,79	0,731
RPL16A	1646,1	1015,0	823,3	354,1	0,50	0,177
RPL16B	1426,7	212,6	1138,6	116,1	0,80	0,055
RPL17B	23,8	12,1	9,1	6,2	0,38	0,074
RPL18B	774,1	131,7	74,7	13,0	0,10	0,000
RPL19A	3387,9	1641,0	2427,6	914,3	0,72	0,346
RPL19B	2800,6	803,8	1454,8	303,6	0,52	0,020
RPL1A	6120,4	6215,2	4074,0	3610,1	0,67	0,590
RPL1B	446,1	276,5	188,3	133,8	0,42	0,144
RPL20A	1102,8	349,9	952,9	335,4	0,86	0,559
RPL20B	3063,2	859,2	1595,2	460,2	0,52	0,024

RPL21A	2508,4	1501,1	1483,5	861,9	0,59	0,281
RPL21B	297,8	163,1	237,4	4,8	0,80	0,487
RPL22B	454,1	320,0	161,1	122,8	0,35	0,138
RPL23A	1298,5	845,5	1018,3	576,2	0,78	0,604
RPL23B	1230,9	516,3	881,6	178,1	0,72	0,248
RPL25	809,7	95,1	932,6	72,5	1,15	0,085
RPL26A	6,1	4,2	2,3	1,8	0,37	0,141
RPL26B	43,6	4,0	20,1	6,7	0,46	0,001
RPL27A	2714,3	1123,4	1450,7	510,0	0,53	0,086
RPL27B	27,5	6,6	8,7	6,5	0,32	0,007
RPL28	7728,1	1087,7	7133,6	1090,7	0,92	0,469
RPL29	5755,5	3413,3	3345,6	1513,0	0,58	0,244
RPL2A	3978,9	2307,4	3572,3	1962,4	0,90	0,797
RPL2B	113,4	19,4	79,0	14,8	0,70	0,031
RPL3	4970,9	1422,8	2792,8	155,2	0,56	0,023
RPL30	65,2	31,5	35,8	29,2	0,55	0,220
RPL31A	6039,3	5498,0	3957,6	3086,6	0,66	0,534
RPL31B	2804,0	2474,2	990,1	754,8	0,35	0,210
RPL32	5271,9	2310,4	4045,6	1944,4	0,77	0,448
RPL33B	10,6	1,7	4,6	3,5	0,44	0,022
RPL34A	2146,2	1124,7	1432,7	737,1	0,67	0,329
RPL34B	15,0	7,6	4,2	5,0	0,28	0,056
RPL35B	17,4	14,0	17,9	22,7	1,03	0,972
RPL36A	576,2	174,2	285,8	80,8	0,50	0,023
RPL36B	3738,0	3269,0	3404,0	2277,5	0,91	0,872
RPL37A	3591,4	1532,6	2309,6	1176,0	0,64	0,233
RPL37B	2326,3	3462,9	1893,2	2676,6	0,81	0,850
RPL40A	21,3	10,7	7,6	3,1	0,36	0,048
RPL41A	20167,0	11227,1	16091,7	7493,6	0,80	0,568
RPL42A	14,4	8,5	5,4	4,3	0,38	0,108
RPL42B	58,4	33,4	35,1	22,9	0,60	0,293
RPL43A	18,1	11,2	7,5	5,1	0,42	0,136
RPL43B	3807,3	2317,2	2587,6	1132,3	0,68	0,381
RPL4A	1571,0	533,8	812,7	322,8	0,52	0,051
RPL4B	830,4	339,9	555,4	152,3	0,67	0,190
RPL5	2123,2	945,8	1410,3	768,3	0,66	0,286
RPL6A	3450,9	678,3	2314,2	755,0	0,67	0,066
RPL6B	19,6	13,7	9,3	8,6	0,48	0,249
RPL7A	2979,5	952,6	1892,9	659,2	0,64	0,110
RPL7B	26,7	22,3	14,5	7,1	0,54	0,335
RPL8A	21,2	37,2	12,0	18,0	0,56	0,670
RPL8B	2795,5	760,4	2840,7	692,8	1,02	0,933
RPL9A	1410,4	879,4	774,1	568,8	0,55	0,270
RPL9B	1093,7	1078,8	885,3	831,4	0,81	0,770
RPM2	47,5	12,3	95,7	24,0	2,02	0,012
RPN1	129,4	127,9	124,0	74,9	0,96	0,945
RPN11	77,7	22,0	113,8	60,5	1,47	0,304
RPN12	104,6	30,0	157,6	21,4	1,51	0,028
RPN13	268,3	152,1	246,6	119,8	0,92	0,830
RPN14	15,2	4,6	10,2	7,5	0,67	0,303

RPN2	449,3	250,3	555,2	258,6	1,24	0,578
RPN3	192,4	92,9	274,2	136,2	1,43	0,359
RPN4	62,9	19,6	110,5	40,4	1,76	0,079
RPN5	350,1	99,8	577,4	140,1	1,65	0,038
RPN6	145,9	61,8	331,7	128,3	2,27	0,040
RPN7	148,5	73,3	243,5	141,5	1,64	0,278
RPN8	586,1	162,4	397,4	66,9	0,68	0,075
RPN9	125,1	41,1	255,4	188,1	2,04	0,225
RPO21	150,0	151,5	146,0	111,9	0,97	0,967
RPO26	213,8	269,3	160,7	205,2	0,75	0,765
RPO31	100,5	38,5	43,2	18,6	0,43	0,036
RPO41	28,5	12,4	36,8	11,2	1,29	0,353
RPP0	2601,7	1246,3	2381,7	848,1	0,92	0,780
RPP1	72,6	34,0	24,8	16,8	0,34	0,045
RPP1A	4624,5	4328,5	4996,3	3667,4	1,08	0,900
RPP1B	4784,4	4632,7	4563,7	3020,0	0,95	0,939
RPP2A	1580,1	760,5	1506,0	1116,6	0,95	0,916
RPP2B	5332,3	4420,2	5561,4	3353,4	1,04	0,937
RPR2	0,8	0,4	0,8	0,7	0,93	0,883
RPS0A	194,3	74,9	61,9	44,3	0,32	0,023
RPS0B	3492,8	903,8	2508,3	467,8	0,72	0,101
RPS10A	1121,6	325,7	1090,2	190,5	0,97	0,873
RPS10B	1093,7	293,3	820,0	39,9	0,75	0,114
RPS11A	7,4	4,6	2,0	2,5	0,27	0,081
RPS11B	24,8	20,3	7,1	6,8	0,29	0,149
RPS12	1537,1	727,0	930,6	250,0	0,61	0,166
RPS13	2502,9	754,6	1351,3	207,7	0,54	0,026
RPS14A	4796,9	1052,7	4805,9	808,8	1,00	0,990
RPS14B	1052,8	577,9	460,9	271,8	0,44	0,113
RPS15	2984,1	1809,6	2864,8	1833,0	0,96	0,929
RPS16A	3161,0	2085,2	1826,9	1101,8	0,58	0,301
RPS16B	3861,7	2990,3	1745,4	1202,4	0,45	0,237
RPS17A	12,7	8,1	4,3	4,7	0,33	0,122
RPS17B	5243,3	1340,3	2903,2	954,0	0,55	0,029
RPS18A	6,3	0,5	2,4	1,8	0,38	0,006
RPS18B	1223,1	213,5	638,6	72,4	0,52	0,002
RPS19A	2142,4	937,3	1250,1	491,0	0,58	0,143
RPS19B	2946,7	2049,1	1979,2	1166,0	0,67	0,443
RPS1A	3992,3	474,4	2598,4	318,6	0,65	0,003
RPS1B	3565,8	1366,4	1657,5	446,5	0,46	0,038
RPS2	2937,5	1073,5	1946,9	362,9	0,66	0,131
RPS21A	4,3	1,8	3,4	2,4	0,79	0,567
RPS21B	3614,5	1381,3	3262,5	1183,3	0,90	0,712
RPS22A	2097,6	1831,0	1282,1	1030,5	0,61	0,467
RPS22B	1141,8	1046,2	313,5	215,8	0,27	0,172
RPS23A	1514,8	1456,9	1111,2	1007,3	0,73	0,665
RPS23B	1,5	2,5	0,2	0,3	0,12	0,347
RPS24A	23,4	4,6	9,2	6,4	0,39	0,012
RPS25A	722,7	137,8	858,6	174,6	1,19	0,267
RPS25B	353,7	86,9	301,4	71,5	0,85	0,388

RPS26B	31,3	21,7	4,6	3,2	0,15	0,051
RPS27A	29,2	10,5	10,7	8,2	0,37	0,032
RPS27B	546,9	435,9	672,9	1034,2	1,23	0,830
RPS30A	4409,7	3101,7	4080,9	2604,1	0,93	0,876
RPS30B	5965,2	2939,1	5252,0	2224,2	0,88	0,712
RPS31	28,5	13,9	16,0	7,9	0,56	0,171
RPS4A	2016,9	594,6	1784,0	257,2	0,88	0,499
RPS4B	1789,0	624,4	1578,5	410,7	0,88	0,594
RPS6A	9,5	6,9	5,9	5,9	0,62	0,455
RPS7A	1837,3	1115,1	1444,1	833,4	0,79	0,593
RPS7B	919,4	312,0	534,9	90,8	0,58	0,056
RPS8A	23,7	20,7	11,9	12,4	0,50	0,366
RPS8B	2903,6	1497,3	2393,8	1223,1	0,82	0,617
RPS9A	1647,9	701,4	1002,9	419,6	0,61	0,166
RPS9B	2,0	1,8	0,1	0,3	0,07	0,087
RPT2	157,1	84,8	256,3	179,8	1,63	0,357
RPT3	120,8	110,9	189,1	158,0	1,57	0,505
RPT4	414,8	84,7	743,2	134,4	1,79	0,006
RPT5	202,8	87,5	263,3	19,0	1,30	0,225
RPT6	204,3	120,1	157,1	98,4	0,77	0,565
RRB1	103,4	19,8	86,6	53,7	0,84	0,580
RRD1	41,3	20,3	28,4	20,7	0,69	0,407
RRD2	41,4	6,7	56,9	12,5	1,37	0,073
RRF1	57,0	21,0	52,3	15,1	0,92	0,730
RRG1	5,6	1,8	15,8	14,8	2,84	0,219
RRG7	51,5	25,7	51,1	24,1	0,99	0,983
RRG8	0,2	0,4	0,7	0,5	3,33	0,175
RRG9	52,7	17,0	29,1	19,8	0,55	0,121
RRI1	19,7	2,7	26,1	14,1	1,33	0,404
RRI2	6,4	1,5	18,4	12,3	2,88	0,100
RRM3	44,7	36,9	23,7	16,3	0,53	0,338
RRN10	38,7	8,4	39,1	12,9	1,01	0,959
RRN11	59,1	16,7	40,5	33,9	0,69	0,363
RRN3	62,6	33,3	72,6	53,9	1,16	0,761
RRN5	0,8	1,1	0,3	0,3	0,41	0,440
RRN6	11,6	8,8	10,8	10,7	0,93	0,912
RRN7	19,7	12,1	9,7	7,9	0,49	0,217
RRN9	22,8	9,0	21,8	8,0	0,96	0,875
RRP1	53,6	24,5	38,1	5,5	0,71	0,261
RRP12	102,2	82,0	69,8	60,7	0,68	0,549
RRP14	52,2	33,8	67,3	57,5	1,29	0,667
RRP15	73,5	46,3	95,9	90,9	1,31	0,675
RRP17	3,3	2,5	1,5	1,0	0,44	0,217
RRP3	68,7	41,7	86,5	44,6	1,26	0,582
RRP36	39,3	26,6	47,0	14,7	1,19	0,633
RRP4	178,0	93,4	174,9	78,1	0,98	0,961
RRP40	66,3	30,9	97,2	60,6	1,46	0,399
RRP42	57,6	17,4	25,6	1,2	0,44	0,011
RRP43	65,1	40,8	38,5	35,3	0,59	0,361
RRP45	90,4	37,2	83,7	25,7	0,93	0,779

RRP46	48,6	27,4	35,8	14,3	0,74	0,438
RRP5	122,2	49,2	142,3	44,7	1,16	0,568
RRP6	34,9	6,1	23,6	15,8	0,68	0,231
RRP7	103,6	10,3	131,5	4,2	1,27	0,002
RRP8	84,2	44,1	59,3	23,3	0,70	0,358
RRP9	41,9	20,1	32,9	6,1	0,78	0,422
RRT1	0,8	0,4	1,6	1,2	2,04	0,238
RRT12	1,5	0,8	1,3	1,1	0,87	0,776
RRT13	27,3	3,8	16,4	4,2	0,60	0,008
RRT14	51,1	18,5	29,1	20,2	0,57	0,160
RRT15	4,6	4,5	3,9	4,5	0,84	0,819
RRT16	0,1	0,2	0,6	0,7	4,96	0,242
RRT2	44,2	24,2	36,4	32,7	0,82	0,715
RRT5	0,8	0,4	0,2	0,3	0,26	0,041
RRT6	2,8	1,0	2,4	2,2	0,85	0,747
RRT7	0,0	0,0	0,0	0,0	#DIV/0!	#DIV/0!
RRT8	29,1	17,8	53,3	54,3	1,83	0,430
RSA1	35,9	7,1	48,5	12,6	1,35	0,133
RSA3	132,1	37,4	107,8	41,3	0,82	0,416
RSA4	49,1	25,0	53,4	14,9	1,09	0,779
RSB1	3,3	1,4	2,3	2,1	0,71	0,479
RSC1	80,0	27,7	151,9	64,9	1,90	0,087
RSC2	41,1	25,6	46,7	16,3	1,13	0,727
RSC3	29,7	8,1	36,4	22,5	1,23	0,596
RSC30	2,0	2,2	1,4	1,2	0,70	0,633
RSC4	77,2	13,8	90,2	21,9	1,17	0,351
RSC58	141,0	54,5	197,1	64,2	1,40	0,231
RSC6	75,3	25,0	54,0	17,8	0,72	0,214
RSC8	305,3	95,8	327,1	29,6	1,07	0,679
RSC9	44,1	25,1	39,3	37,8	0,89	0,841
RSE1	39,1	18,2	44,2	9,3	1,13	0,634
RSF1	13,5	3,9	16,7	2,9	1,24	0,235
RSF2	55,5	21,9	49,9	8,2	0,90	0,651
RSM18	54,3	30,3	57,9	22,2	1,07	0,855
RSM19	200,7	124,9	339,5	213,5	1,69	0,305
RSM22	52,9	26,2	117,1	78,0	2,21	0,169
RSM23	61,4	43,0	87,2	48,4	1,42	0,456
RSM24	39,7	17,2	41,4	15,0	1,04	0,886
RSM25	177,3	80,5	255,4	67,7	1,44	0,188
RSM26	74,5	41,3	36,5	26,9	0,49	0,175
RSM27	91,6	45,7	102,6	29,6	1,12	0,701
RSM28	28,5	12,6	41,8	26,7	1,47	0,402
RSM7	95,9	14,7	76,0	18,9	0,79	0,148
RSN1	92,4	54,9	68,3	10,6	0,74	0,422
RSP5	19,3	14,8	37,9	28,7	1,97	0,291
RTA1	1,8	0,9	9,1	12,2	4,96	0,281
RTC1	43,5	12,1	44,7	30,7	1,03	0,944
RTC2	78,4	40,4	72,8	21,0	0,93	0,813
RTC3	94,1	81,6	1517,4	1402,2	16,12	0,089
RTC4	3,2	1,3	4,0	3,1	1,24	0,654

RTC5	39,8	32,4	29,5	20,4	0,74	0,609
RTC6	15,8	1,8	9,6	6,7	0,61	0,128
RTF1	111,5	16,5	110,6	26,5	0,99	0,959
RTG2	224,3	93,3	112,9	76,2	0,50	0,114
RTG3	9,3	7,2	9,9	10,3	1,06	0,928
RTK1	35,6	16,7	51,8	35,4	1,46	0,440
RTN1	195,8	74,9	132,4	44,1	0,68	0,195
RTN2	14,3	5,5	50,4	3,6	3,52	0,000
RTR1	3,2	2,1	5,8	2,4	1,80	0,155
RTR2	9,3	3,8	8,5	6,6	0,91	0,836
RTS1	81,7	39,0	109,1	36,1	1,34	0,341
RTS2	2,7	0,7	4,3	2,9	1,63	0,309
RTS3	36,2	23,3	44,5	30,1	1,23	0,676
RTT10	60,1	27,7	52,3	22,0	0,87	0,673
RTT101	16,6	3,8	26,4	2,4	1,59	0,005
RTT102	63,4	39,3	43,9	10,5	0,69	0,374
RTT103	51,5	14,3	29,0	12,5	0,56	0,055
RTT105	10,5	10,9	7,8	4,1	0,75	0,663
RTT106	84,4	42,5	55,7	15,2	0,66	0,250
RTT107	68,6	63,0	5,3	4,5	0,08	0,092
RTT109	7,7	3,6	0,9	0,9	0,12	0,010
RUD3	36,9	18,4	33,4	22,1	0,90	0,815
RUP1	27,7	11,8	35,6	22,8	1,28	0,563
RVB1	108,3	23,6	110,6	40,5	1,02	0,925
RVB2	189,2	50,4	226,2	56,7	1,20	0,367
RVS161	107,7	77,0	211,4	79,2	1,96	0,110
RVS167	218,8	147,8	249,5	134,3	1,14	0,769
RXT2	34,1	20,6	58,1	10,7	1,70	0,085
RXT3	58,2	26,7	85,4	42,7	1,47	0,322
SAC1	128,2	93,8	97,1	65,3	0,76	0,607
SAC3	52,6	33,6	44,2	25,8	0,84	0,704
SAC6	164,8	45,5	217,2	34,8	1,32	0,117
SAC7	33,9	29,3	25,5	17,8	0,75	0,642
SAD1	30,3	7,4	34,8	11,9	1,15	0,540
SAF1	14,4	4,3	19,7	13,2	1,37	0,472
SAG1	20,4	8,6	63,6	11,9	3,11	0,001
SAH1	1929,6	963,7	2091,3	643,9	1,08	0,790
SAK1	79,2	47,6	71,7	26,1	0,91	0,792
SAL1	6,4	1,9	39,3	31,9	6,17	0,085
SAM1	352,2	205,1	319,3	47,5	0,91	0,765
SAM2	158,8	176,6	377,6	174,6	2,38	0,128
SAM3	67,4	30,5	150,9	82,0	2,24	0,105
SAM35	39,6	27,1	56,4	42,2	1,43	0,527
SAM4	1992,9	245,3	742,4	184,0	0,37	0,000
SAM50	52,6	16,5	65,2	21,7	1,24	0,390
SAP1	23,1	7,6	19,3	15,8	0,83	0,678
SAP155	253,8	115,3	218,6	113,7	0,86	0,679
SAP185	64,1	24,9	62,8	26,4	0,98	0,945
SAP190	45,8	12,8	48,9	25,5	1,07	0,834
SAP4	86,1	51,2	95,3	65,4	1,11	0,830

SAR1	633,9	166,2	273,8	51,0	0,43	0,006
SAS10	86,6	62,7	133,4	97,7	1,54	0,451
SAS2	25,3	17,3	15,2	4,8	0,60	0,305
SAS3	174,1	84,0	115,3	74,3	0,66	0,335
SAS4	66,1	38,5	90,3	66,9	1,37	0,554
SAS5	50,9	18,6	35,7	9,5	0,70	0,196
SAT4	158,0	75,9	145,7	11,5	0,92	0,759
SAW1	17,9	3,6	19,0	4,7	1,06	0,726
SAY1	22,6	13,4	6,3	8,1	0,28	0,083
SBA1	318,4	164,7	475,2	283,3	1,49	0,376
SBE2	23,3	9,3	39,7	21,8	1,70	0,216
SBE22	18,5	11,3	13,3	5,2	0,72	0,441
SBH1	554,1	326,1	310,4	162,7	0,56	0,229
SCC2	21,8	6,2	18,6	14,2	0,85	0,694
SCC4	47,8	23,4	34,5	18,1	0,72	0,400
SCD5	38,2	33,4	50,5	20,6	1,32	0,556
SCEI	29,8	44,5	27,6	45,7	0,93	0,948
SCH9	57,9	47,3	65,1	33,3	1,13	0,810
SCJ1	59,2	18,8	67,1	36,5	1,13	0,715
SCL1	152,8	85,1	198,2	90,9	1,30	0,494
SCM3	11,3	6,2	30,1	47,8	2,66	0,466
SCM4	32,4	6,6	17,5	8,6	0,54	0,034
SCO1	36,4	9,1	26,9	4,8	0,74	0,112
SCO2	74,6	42,3	54,0	39,1	0,72	0,502
SCP160	372,6	226,1	254,4	153,1	0,68	0,420
SCS2	498,6	227,2	437,1	179,7	0,88	0,686
SCS22	119,3	56,3	71,7	21,1	0,60	0,164
SCS3	118,0	27,4	32,8	19,3	0,28	0,002
SCS7	557,0	294,1	699,1	184,3	1,26	0,444
SCT1	39,5	4,8	38,9	14,9	0,98	0,940
SCW10	483,6	132,7	156,0	55,3	0,32	0,004
SCW11	96,7	22,0	116,7	49,4	1,21	0,487
SCW4	809,3	457,7	834,7	453,0	1,03	0,940
SCY1	9,6	5,2	5,6	4,6	0,58	0,292
SDA1	105,6	50,6	94,9	43,7	0,90	0,758
SDC1	154,1	86,4	237,3	103,9	1,54	0,264
SDC25	6,4	2,5	15,7	14,8	2,44	0,264
SDH1	57,4	19,7	99,3	25,0	1,73	0,039
SDH2	57,5	20,0	102,0	49,5	1,77	0,146
SDH4	58,1	12,7	165,0	22,0	2,84	0,000
SDL1	15,8	5,5	10,9	5,4	0,69	0,249
SDO1	110,2	58,0	88,9	43,9	0,81	0,579
SDP1	3,6	2,2	1,3	1,0	0,36	0,099
SDS22	33,3	14,3	46,3	21,3	1,39	0,350
SDS23	35,6	16,7	67,1	36,5	1,88	0,168
SDS24	132,9	65,5	369,6	311,6	2,78	0,188
SDS3	2,3	1,1	2,5	1,8	1,10	0,826
SDT1	64,1	33,4	31,5	25,5	0,49	0,172
SEC1	102,3	43,7	71,3	45,7	0,70	0,365
SEC10	87,2	29,3	72,1	10,2	0,83	0,369

SEC12	47,3	31,8	58,4	35,8	1,23	0,658
SEC13	121,1	56,7	107,1	81,7	0,88	0,788
SEC14	123,7	83,2	210,2	166,9	1,70	0,389
SEC15	35,9	15,7	31,5	12,7	0,88	0,679
SEC16	214,3	149,1	239,9	167,1	1,12	0,827
SEC17	116,1	37,9	139,6	53,4	1,20	0,499
SEC18	129,4	60,7	109,4	33,1	0,85	0,584
SEC2	55,0	14,5	35,5	10,8	0,64	0,074
SEC20	54,2	33,3	21,5	14,5	0,40	0,122
SEC21	189,1	66,3	173,5	25,3	0,92	0,676
SEC22	71,9	24,5	45,4	29,8	0,63	0,218
SEC23	170,4	35,0	143,9	43,7	0,84	0,380
SEC24	160,1	92,4	108,1	53,9	0,68	0,368
SEC26	647,4	368,7	515,9	236,7	0,80	0,570
SEC27	246,9	194,7	208,5	122,9	0,84	0,750
SEC28	305,6	87,5	161,1	44,2	0,53	0,026
SEC3	43,3	16,6	57,3	39,6	1,32	0,539
SEC31	197,6	126,3	156,8	99,7	0,79	0,630
SEC39	36,6	6,0	25,8	3,5	0,70	0,021
SEC4	195,5	106,6	154,5	57,8	0,79	0,524
SEC5	30,2	14,5	42,4	18,5	1,41	0,338
SEC59	1,6	1,4	0,3	0,3	0,16	0,097
SEC6	64,5	26,9	53,9	38,0	0,84	0,665
SEC61	225,5	109,0	145,4	76,0	0,64	0,273
SEC62	68,7	21,7	25,8	17,2	0,38	0,021
SEC63	1051,7	869,2	598,6	457,7	0,57	0,392
SEC65	84,7	26,0	63,9	42,0	0,75	0,432
SEC66	42,8	12,7	27,0	18,4	0,63	0,206
SEC7	132,4	49,2	119,4	9,0	0,90	0,621
SEC72	126,7	111,3	134,8	123,8	1,06	0,926
SEC8	45,7	26,4	58,4	12,8	1,28	0,418
SEC9	50,9	9,6	33,7	11,5	0,66	0,062
SED1	1596,0	481,6	2674,7	432,3	1,68	0,016
SED4	58,4	14,0	88,2	36,7	1,51	0,180
SED5	28,4	6,3	38,9	15,9	1,37	0,266
SEE1	56,9	34,8	25,4	9,0	0,45	0,131
SEF1	4,0	1,8	16,2	8,3	4,10	0,028
SEH1	67,9	22,8	36,5	20,9	0,54	0,089
SEM1	272,9	108,6	448,2	206,0	1,64	0,183
SEN1	139,7	63,6	72,4	10,5	0,52	0,082
SEN15	20,1	5,1	33,3	19,0	1,65	0,230
SEN2	29,0	6,9	20,0	10,5	0,69	0,201
SEN34	35,9	51,4	27,5	48,9	0,77	0,820
SEN54	28,7	9,7	28,6	20,8	1,00	0,991
SEO1	3,0	1,4	5,2	4,9	1,73	0,419
SER1	1303,2	869,4	604,4	214,6	0,46	0,170
SER2	325,4	58,6	119,5	42,6	0,37	0,001
SER3	706,4	602,5	1314,1	891,9	1,86	0,302
SER33	4,7	2,2	4,9	4,2	1,04	0,943
SES1	616,2	335,6	998,8	567,5	1,62	0,290

SET1	20,7	7,4	10,6	7,4	0,51	0,102
SET2	119,1	90,4	92,3	57,7	0,77	0,635
SET3	27,3	7,9	31,7	24,2	1,16	0,740
SET4	4,0	3,9	4,9	4,4	1,23	0,763
SET5	45,8	11,8	31,7	6,4	0,69	0,082
SET6	1,0	1,2	1,5	1,5	1,54	0,596
SEY1	77,2	43,0	67,8	37,6	0,88	0,755
SFA1	231,5	97,2	462,0	143,6	2,00	0,038
SFB2	100,0	81,8	137,0	88,5	1,37	0,562
SFB3	151,8	118,9	127,7	60,7	0,84	0,731
SFC1	0,0	0,1	0,1	0,3	3,14	0,540
SFG1	17,7	7,0	12,4	9,8	0,70	0,422
SFH1	52,1	45,6	36,9	30,2	0,71	0,599
SFH5	127,3	17,9	155,1	41,0	1,22	0,260
SFI1	72,8	27,2	56,8	42,7	0,78	0,550
SFK1	68,7	58,6	59,1	47,4	0,86	0,807
SFL1	12,9	9,9	24,3	17,0	1,88	0,291
SFP1	13,2	3,0	39,9	19,5	3,02	0,036
SFT1	71,6	36,8	29,4	5,2	0,41	0,064
SFT2	266,5	137,0	201,9	109,8	0,76	0,489
SGA1	1,5	0,6	1,5	1,0	0,98	0,953
SGD1	65,8	28,8	56,2	22,2	0,85	0,615
SGE1	86,1	49,6	48,5	17,1	0,56	0,202
SGF11	0,9	1,3	11,0	9,5	11,58	0,082
SGF29	14,9	9,7	10,3	8,0	0,69	0,494
SGF73	44,1	13,9	75,9	58,9	1,72	0,334
SGM1	232,9	58,5	149,0	52,5	0,64	0,077
SGN1	42,2	19,2	52,8	17,4	1,25	0,444
SGO1	69,4	10,6	14,6	8,6	0,21	0,000
SGS1	15,9	8,9	10,5	7,6	0,66	0,390
SGT1	76,6	17,1	98,4	19,8	1,29	0,145
SGT2	445,6	175,8	782,1	276,7	1,76	0,086
SGV1	113,5	71,0	119,7	68,7	1,05	0,904
SHC1	3,9	1,7	9,8	7,5	2,48	0,178
SHE1	335,7	154,3	65,5	31,8	0,20	0,014
SHE10	90,9	24,1	105,7	23,8	1,16	0,414
SHE2	50,0	63,3	74,9	56,7	1,50	0,580
SHE3	35,3	13,3	17,3	11,6	0,49	0,086
SHE4	21,3	6,9	52,0	39,5	2,45	0,177
SHE9	60,3	40,7	59,9	46,4	0,99	0,990
SHG1	13,1	7,2	29,6	8,8	2,26	0,027
SHM1	294,4	149,5	296,1	126,2	1,01	0,987
SHM2	2692,2	741,2	1067,1	386,9	0,40	0,008
SHO1	58,0	55,1	40,1	42,7	0,69	0,625
SHP1	79,3	43,1	96,1	27,5	1,21	0,538
SHQ1	14,6	6,4	10,3	7,7	0,70	0,419
SHR3	206,9	71,8	114,4	35,7	0,55	0,060
SHR5	34,9	19,5	18,3	14,0	0,52	0,215
SHS1	71,1	49,1	74,1	25,1	1,04	0,917
SHU1	6,3	3,7	5,5	4,0	0,87	0,780

SHU2	29,2	15,3	45,4	21,6	1,56	0,266
SHY1	11,4	2,8	17,6	12,0	1,54	0,354
SIA1	13,0	5,8	28,1	8,5	2,17	0,026
SIC1	56,4	44,2	26,6	28,4	0,47	0,299
SIF2	33,0	9,9	31,3	12,5	0,95	0,836
SIL1	20,7	14,2	14,5	13,6	0,70	0,553
SIM1	356,8	105,4	93,1	24,3	0,26	0,003
SIN3	55,7	30,9	61,1	10,6	1,10	0,755
SIN4	44,0	12,7	39,1	18,9	0,89	0,678
SIP1	18,6	7,7	48,2	61,6	2,59	0,378
SIP18	17,3	6,0	51,8	49,3	3,00	0,214
SIP2	17,1	10,9	12,0	8,4	0,70	0,487
SIP3	45,1	27,7	48,6	12,0	1,08	0,825
SIP4	2,2	1,6	6,2	5,7	2,84	0,219
SIP5	21,7	14,3	44,6	35,3	2,06	0,273
SIR1	27,8	10,5	13,1	9,8	0,47	0,087
SIR2	47,9	23,7	34,5	23,6	0,72	0,452
SIR3	66,9	43,4	77,0	53,5	1,15	0,781
SIR4	56,7	38,0	46,4	31,0	0,82	0,689
SIS1	116,6	45,1	304,6	129,5	2,61	0,034
SIS2	91,9	55,6	111,0	56,0	1,21	0,647
SIT1	244,2	124,6	22,6	15,4	0,09	0,012
SIT4	140,6	54,3	160,0	34,2	1,14	0,569
SIW14	995,1	267,6	577,0	102,0	0,58	0,027
SIZ1	23,0	4,6	23,9	17,6	1,04	0,923
SKG1	19,0	11,1	10,5	7,7	0,56	0,257
SKG3	33,9	14,9	20,4	19,6	0,60	0,313
SKG6	33,6	14,7	4,5	3,3	0,13	0,008
SKI2	141,5	34,3	135,8	47,2	0,96	0,853
SKI3	65,4	40,9	40,5	27,1	0,62	0,348
SKI6	126,6	38,7	158,1	73,2	1,25	0,475
SKI7	55,4	21,9	87,3	19,7	1,58	0,074
SKI8	172,5	81,2	107,5	57,8	0,62	0,240
SKM1	29,0	14,7	18,2	9,2	0,63	0,260
SKN1	48,6	19,0	85,6	26,2	1,76	0,062
SKN7	22,8	6,9	14,3	9,9	0,63	0,211
SKO1	39,4	20,1	30,8	20,7	0,78	0,575
SKP1	341,9	197,0	582,6	291,0	1,70	0,220
SKP2	17,6	9,2	31,8	9,0	1,80	0,070
SKS1	40,3	33,3	36,7	21,6	0,91	0,862
SKT5	153,4	134,8	312,5	268,4	2,04	0,330
SKY1	39,9	37,5	23,7	9,7	0,59	0,434
SLA1	52,6	36,9	39,6	23,5	0,75	0,573
SLA2	109,2	53,6	146,4	54,7	1,34	0,368
SLC1	78,9	66,1	36,5	30,9	0,46	0,289
SLD2	31,2	9,1	8,3	7,3	0,27	0,008
SLD3	18,3	13,2	17,8	7,8	0,97	0,942
SLD5	54,7	3,6	20,7	2,9	0,38	0,000
SLF1	51,7	10,8	58,8	19,7	1,14	0,555
SLG1	19,9	13,5	30,4	22,6	1,52	0,458

SLH1	33,7	10,3	29,1	20,8	0,86	0,708
SLI1	4,8	1,9	5,2	3,9	1,09	0,840
SLI15	24,8	6,7	23,5	3,3	0,95	0,740
SLK19	77,3	65,4	33,4	16,7	0,43	0,241
SLM1	84,2	59,9	95,9	51,8	1,14	0,779
SLM2	10,0	3,6	25,2	23,9	2,52	0,255
SLM3	53,3	7,0	17,7	11,9	0,33	0,002
SLM4	87,5	35,9	80,8	26,0	0,92	0,772
SLM5	6,8	3,8	5,3	4,1	0,78	0,610
SLM6	2,6	2,9	2,8	3,2	1,05	0,951
SLN1	34,4	16,5	31,8	21,8	0,92	0,852
SLO1	0,8	0,7	2,2	1,7	2,68	0,195
SLP1	17,4	11,7	14,4	11,5	0,83	0,730
SLS1	27,6	13,2	11,1	7,7	0,40	0,074
SLT2	38,3	21,6	70,4	39,0	1,84	0,200
SLU7	59,3	35,0	49,4	33,5	0,83	0,695
SLX1	20,0	1,8	22,7	4,0	1,14	0,255
SLX4	28,4	9,8	35,5	6,1	1,25	0,263
SLX5	64,6	23,8	57,1	33,5	0,88	0,730
SLX8	13,0	4,5	19,6	10,8	1,51	0,299
SLX9	50,4	15,7	62,0	23,2	1,23	0,439
SLY1	80,5	39,7	53,4	39,5	0,66	0,370
SLY41	27,5	7,5	31,0	9,9	1,13	0,597
SLZ1	0,8	1,0	0,5	0,5	0,56	0,527
SMA1	0,2	0,3	0,0	0,0	0,00	0,356
SMA2	0,0	0,0	0,1	0,1	#DIV/0!	0,356
SMB1	120,3	54,3	53,4	26,7	0,44	0,069
SMC1	97,2	38,8	26,0	8,6	0,27	0,012
SMC2	23,2	6,5	11,5	8,4	0,50	0,070
SMC3	88,9	38,2	17,7	4,4	0,20	0,010
SMC4	106,4	76,2	72,5	41,9	0,68	0,465
SMC5	73,0	10,2	31,1	3,9	0,43	0,000
SMC6	87,6	34,8	37,3	21,0	0,43	0,048
SMD2	32,3	12,9	29,0	22,1	0,90	0,804
SMD3	55,2	27,3	53,5	28,0	0,97	0,934
SME1	114,5	25,9	169,9	51,8	1,48	0,104
SMF1	80,5	38,4	44,4	7,2	0,55	0,114
SMF2	83,7	25,6	20,1	12,1	0,24	0,004
SMF3	56,0	24,8	43,4	23,3	0,77	0,486
SMI1	496,6	157,1	186,7	24,4	0,38	0,008
SMK1	1,5	0,6	0,7	0,4	0,42	0,052
SML1	231,5	128,1	240,7	140,3	1,04	0,926
SMM1	84,5	56,2	49,4	33,2	0,58	0,323
SMP1	1,9	1,4	4,1	3,2	2,15	0,260
SMP3	11,8	6,9	16,8	7,4	1,42	0,358
SMT3	520,4	237,4	432,2	210,8	0,83	0,598
SMX3	0,0	0,0	0,0	0,0	#DIV/0!	#DIV/0!
SMY1	36,9	12,7	28,1	5,8	0,76	0,253
SMY2	52,0	30,7	70,0	57,6	1,34	0,603
SNA2	48,8	31,1	119,3	65,3	2,44	0,099

SNA3	178,3	159,8	306,5	312,3	1,72	0,492
SNA4	19,6	8,0	35,0	11,9	1,78	0,076
SNC1	76,1	24,8	74,9	27,0	0,98	0,949
SNF1	41,2	10,0	45,3	7,0	1,10	0,533
SNF11	12,6	4,0	13,9	13,9	1,10	0,867
SNF12	20,7	8,2	9,0	6,2	0,44	0,065
SNF2	40,2	30,5	34,4	18,5	0,85	0,753
SNF3	17,1	5,1	15,4	12,4	0,90	0,808
SNF4	69,5	7,6	21,9	14,6	0,31	0,001
SNF5	55,1	6,7	42,8	6,5	0,78	0,039
SNF6	202,2	30,3	97,4	23,3	0,48	0,002
SNF7	328,1	81,2	478,8	31,1	1,46	0,013
SNF8	64,8	26,4	82,4	45,2	1,27	0,525
SNG1	15,9	9,0	30,8	21,3	1,94	0,244
SNM1	15,0	6,3	24,8	17,1	1,65	0,323
SNN1	35,4	36,5	94,4	80,6	2,67	0,231
SNO1	64,9	9,7	102,3	27,7	1,58	0,044
SNO3	11,3	2,3	6,1	4,9	0,54	0,099
SNO4	0,1	0,1	0,8	0,6	8,40	0,045
SNQ2	88,6	46,4	63,0	15,5	0,71	0,335
SNT1	113,8	79,3	113,4	78,9	1,00	0,994
SNT2	11,6	6,2	23,7	5,6	2,04	0,028
SNT309	61,5	26,9	86,1	31,9	1,40	0,283
SNU114	38,0	17,3	50,9	12,4	1,34	0,271
SNU13	0,7	0,8	0,7	0,9	0,97	0,973
SNU23	23,2	11,5	11,1	6,6	0,48	0,117
SNU56	19,5	5,7	15,8	1,7	0,81	0,262
SNU66	26,8	14,9	19,9	13,9	0,74	0,523
SNU71	24,1	18,8	41,4	24,2	1,72	0,302
SNX3	131,0	36,5	105,4	24,2	0,80	0,286
SNX4	25,3	14,9	55,9	47,5	2,21	0,266
SNX41	17,1	7,3	28,0	9,9	1,63	0,128
SNZ1	628,9	314,8	895,0	481,3	1,42	0,390
SNZ2	0,5	0,3	0,8	0,9	1,72	0,521
SNZ3	36,8	15,3	69,1	54,3	1,88	0,296
SOD1	952,1	306,1	1899,2	795,5	1,99	0,068
SOD2	104,0	48,4	380,1	76,5	3,65	0,001
SOF1	51,0	16,4	28,0	23,0	0,55	0,155
SOG2	70,3	58,9	86,6	84,6	1,23	0,762
SOH1	25,4	12,9	35,4	32,5	1,39	0,588
SOK1	73,6	18,3	100,8	14,6	1,37	0,059
SOK2	143,0	43,4	105,4	29,7	0,74	0,203
SOL1	30,9	6,7	123,9	32,7	4,01	0,001
SOL2	77,8	17,7	136,3	71,3	1,75	0,162
SOL3	458,4	297,8	650,0	490,9	1,42	0,529
SOL4	12,2	4,7	58,4	51,3	4,80	0,123
SOP4	282,8	124,5	275,6	64,9	0,97	0,922
SOR2	26,9	5,3	18,2	12,5	0,68	0,248
SOV1	13,3	2,3	14,8	1,4	1,11	0,314
SPA2	61,4	47,9	25,3	22,0	0,41	0,219

SPB1	195,1	168,0	210,0	150,8	1,08	0,899
SPB4	74,3	17,7	32,9	22,3	0,44	0,027
SPC1	10,0	5,1	9,0	7,7	0,90	0,839
SPC105	35,1	17,0	23,1	12,6	0,66	0,303
SPC110	51,4	34,1	16,2	11,7	0,31	0,099
SPC19	63,6	26,5	56,2	23,8	0,88	0,693
SPC24	0,3	0,6	0,0	0,0	0,00	0,295
SPC25	40,4	14,0	9,8	8,0	0,24	0,009
SPC29	9,5	5,2	1,4	1,4	0,15	0,024
SPC3	311,5	144,5	439,1	211,4	1,41	0,358
SPC34	37,1	12,2	17,6	2,0	0,47	0,020
SPC42	72,5	28,7	29,4	15,0	0,41	0,037
SPC72	19,5	9,9	25,2	7,4	1,29	0,389
SPC97	29,4	11,5	5,8	4,0	0,20	0,008
SPC98	45,0	16,1	10,2	4,4	0,23	0,006
SPE1	81,7	15,4	85,3	31,8	1,04	0,845
SPE2	48,7	10,5	43,5	6,9	0,89	0,440
SPE3	467,8	382,2	429,8	210,7	0,92	0,868
SPE4	31,5	27,1	13,5	2,8	0,43	0,235
SPF1	151,2	41,2	76,8	20,4	0,51	0,018
SPG1	2,1	0,6	7,7	10,3	3,69	0,321
SPG3	33,4	15,6	21,0	14,3	0,63	0,282
SPG4	9,8	6,5	14,9	14,9	1,52	0,554
SPG5	86,1	28,6	71,6	34,9	0,83	0,545
SPH1	31,4	23,6	6,0	5,6	0,19	0,082
SPI1	98,7	90,4	608,7	387,1	6,17	0,043
SPL2	30,6	4,7	224,4	69,5	7,34	0,001
SPN1	79,2	8,7	70,2	9,4	0,89	0,209
SPO1	11,5	11,3	73,2	101,9	6,38	0,274
SPO11	2,3	1,1	1,4	1,6	0,61	0,389
SPO12	71,5	10,4	87,3	26,0	1,22	0,302
SPO13	1,3	0,8	1,0	0,9	0,78	0,655
SPO14	37,3	16,4	34,9	6,5	0,94	0,797
SPO20	0,3	0,4	0,1	0,1	0,22	0,367
SPO21	12,5	4,4	8,2	6,6	0,66	0,321
SPO22	2,7	2,4	32,8	58,6	12,24	0,344
SPO23	8,8	3,2	7,0	5,5	0,80	0,591
SPO7	61,6	49,5	36,2	30,5	0,59	0,415
SPO71	18,0	5,7	24,5	15,3	1,36	0,458
SPO73	23,4	20,7	20,2	15,5	0,86	0,811
SPO74	13,1	5,8	5,3	3,7	0,41	0,064
SPO75	3,7	1,7	1,8	1,4	0,49	0,139
SPO77	0,5	0,7	6,3	10,5	12,50	0,315
SPP1	38,1	18,7	43,8	11,7	1,15	0,625
SPP2	273,4	189,2	117,7	44,2	0,43	0,160
SPP381	0,6	0,4	0,3	0,4	0,58	0,445
SPP382	9,5	8,1	11,1	11,4	1,16	0,832
SPP41	110,1	61,3	93,8	67,4	0,85	0,733
SPR1	6,3	4,5	5,1	3,9	0,80	0,683
SPR28	0,0	0,0	0,0	0,1	#DIV/0!	0,356

SPR3	1,5	2,1	1,9	2,7	1,25	0,830
SPR6	58,0	43,8	76,2	86,9	1,31	0,722
SPS1	1,5	0,3	0,6	0,6	0,38	0,043
SPS100	3,9	4,3	9,9	11,0	2,53	0,351
SPS18	0,0	0,1	0,0	0,0	0,00	0,356
SPS19	9,2	4,8	11,7	8,3	1,27	0,627
SPS2	0,2	0,3	0,2	0,4	1,17	0,917
SPS22	9,2	5,5	31,7	12,0	3,45	0,015
SPS4	4,8	1,6	1,6	1,2	0,33	0,019
SPT10	1,2	1,6	1,4	1,0	1,17	0,837
SPT14	24,7	19,6	12,6	8,8	0,51	0,305
SPT15	159,3	56,2	156,6	25,2	0,98	0,933
SPT16	97,9	61,9	103,6	43,7	1,06	0,886
SPT2	33,1	9,7	41,2	6,3	1,24	0,213
SPT20	39,1	15,1	41,0	31,9	1,05	0,919
SPT21	113,7	62,8	24,0	10,9	0,21	0,031
SPT23	95,4	27,8	169,0	46,2	1,77	0,034
SPT3	49,4	25,5	59,1	29,9	1,20	0,640
SPT4	169,8	125,3	287,7	173,9	1,69	0,314
SPT5	3,8	0,6	4,7	3,4	1,22	0,635
SPT6	149,5	27,3	106,7	49,3	0,71	0,180
SPT7	33,5	21,5	28,6	21,1	0,85	0,757
SPT8	48,6	16,5	21,4	16,7	0,44	0,060
SQS1	48,6	19,2	96,3	46,6	1,98	0,107
SQT1	144,6	21,8	118,2	24,7	0,82	0,159
SRB2	48,3	18,3	23,1	9,1	0,48	0,048
SRB4	13,2	8,5	5,8	4,2	0,44	0,169
SRB5	27,3	14,9	37,5	18,4	1,37	0,425
SRB6	99,1	54,4	166,6	71,1	1,68	0,182
SRB7	61,8	28,8	45,5	14,2	0,74	0,348
SRB8	22,8	11,4	35,5	15,1	1,56	0,228
SRC1	45,1	11,6	68,7	7,1	1,53	0,013
SRD1	50,0	21,4	46,6	26,7	0,93	0,847
SRL1	256,6	168,3	302,6	109,4	1,18	0,663
SRL2	0,8	0,7	1,3	1,0	1,59	0,475
SRL3	23,2	41,0	44,8	71,0	1,93	0,617
SRL4	6,3	5,5	14,5	12,5	2,29	0,278
SRM1	26,8	9,0	31,6	11,7	1,18	0,542
SRN2	43,1	23,6	40,9	17,7	0,95	0,888
SRO7	25,7	7,6	30,1	21,5	1,17	0,712
SRO77	15,1	4,0	8,3	5,7	0,55	0,100
SRO9	32,3	15,6	38,7	19,0	1,20	0,620
SRP1	47,8	13,7	23,8	3,8	0,50	0,015
SRP101	36,8	16,8	24,8	14,2	0,67	0,317
SRP102	110,2	38,5	72,0	25,8	0,65	0,151
SRP14	265,4	111,6	172,4	80,2	0,65	0,225
SRP21	250,4	50,3	209,7	28,2	0,84	0,209
SRP40	191,1	71,4	112,2	47,0	0,59	0,114
SRP54	236,4	66,8	228,1	51,0	0,96	0,850
SRP68	90,3	39,3	62,2	7,2	0,69	0,209

SRP72	117,0	45,1	126,1	22,4	1,08	0,729
SRS2	84,1	23,7	21,8	8,3	0,26	0,003
SRT1	3,3	2,3	14,8	20,2	4,53	0,299
SRV2	270,0	162,1	173,2	92,6	0,64	0,340
SRX1	14,8	14,3	88,0	84,1	5,95	0,137
SRY1	59,9	41,1	67,8	30,8	1,13	0,768
SSA1	855,6	440,7	3237,5	1556,1	3,78	0,026
SSA2	2686,5	1626,6	2945,0	1255,1	1,10	0,810
SSA3	7,2	1,8	35,2	14,9	4,92	0,010
SSA4	24,4	23,2	84,2	70,8	3,45	0,159
SSB1	3461,0	714,1	2583,8	274,2	0,75	0,062
SSB2	3104,8	571,3	2959,6	510,6	0,95	0,718
SSC1	155,8	103,5	652,6	344,2	4,19	0,033
SSD1	96,5	29,6	155,8	53,8	1,62	0,101
SSE1	924,4	39,4	1812,2	178,8	1,96	0,000
SSE2	51,3	19,4	260,6	130,5	5,08	0,019
SSF1	22,8	2,6	6,2	4,2	0,27	0,001
SSF2	82,3	36,6	115,2	60,1	1,40	0,386
SSH1	323,7	131,9	63,9	56,8	0,20	0,011
SSH4	14,4	5,5	13,7	11,2	0,95	0,912
SSK1	7,0	4,2	15,5	8,0	2,23	0,107
SSK2	87,8	67,7	63,6	51,2	0,72	0,589
SSK22	18,5	4,2	41,4	26,0	2,24	0,133
SSL1	30,1	12,5	26,5	4,9	0,88	0,611
SSL2	65,4	19,7	134,0	87,9	2,05	0,178
SSM4	60,6	19,1	77,3	18,5	1,28	0,253
SSN2	14,0	2,7	29,8	20,1	2,13	0,172
SSN3	10,9	4,8	15,3	7,9	1,40	0,382
SSN8	5,2	2,5	71,4	19,7	13,72	0,001
SSO1	94,5	39,9	103,8	23,5	1,10	0,702
SSO2	167,1	87,6	198,7	102,0	1,19	0,655
SSP1	19,9	10,3	5,7	3,8	0,29	0,042
SSP120	586,1	308,1	776,4	261,3	1,32	0,382
SSP2	10,0	0,4	2,5	1,8	0,25	0,000
SSQ1	41,3	19,1	67,9	45,9	1,64	0,326
SST2	91,3	57,6	210,2	78,4	2,30	0,050
SSU1	251,8	151,1	181,1	83,2	0,72	0,443
SSU72	54,4	22,5	45,6	31,3	0,84	0,662
SSY1	42,6	25,8	56,1	12,5	1,32	0,382
SSY5	2,1	0,7	2,3	2,1	1,07	0,893
SSZ1	2523,2	1651,9	4097,0	2742,6	1,62	0,364
STB1	12,3	1,5	4,6	3,1	0,37	0,004
STB2	6,5	0,9	13,6	3,9	2,08	0,013
STB3	20,6	22,9	52,4	79,3	2,54	0,471
STB4	27,3	7,1	27,4	9,4	1,00	0,989
STB5	26,9	5,8	40,4	35,0	1,50	0,476
STB6	24,9	6,1	28,3	5,0	1,14	0,424
STD1	30,1	10,9	48,9	23,2	1,63	0,192
STE11	12,6	5,6	16,1	11,2	1,28	0,596
STE12	50,7	33,9	75,1	68,1	1,48	0,545

STE13	61,9	42,3	67,0	53,1	1,08	0,886
STE14	179,7	72,7	267,4	90,7	1,49	0,182
STE18	182,0	157,1	213,7	158,4	1,17	0,786
STE2	519,1	99,1	1446,8	349,9	2,79	0,002
STE20	54,7	13,4	59,0	15,7	1,08	0,691
STE23	179,9	67,1	179,5	13,3	1,00	0,992
STE24	224,3	22,7	346,9	112,1	1,55	0,076
STE3	4,7	1,8	2,9	2,0	0,61	0,227
STE4	127,7	42,8	210,3	74,7	1,65	0,104
STE5	37,6	28,0	57,6	35,7	1,53	0,413
STE50	61,4	18,9	90,8	14,8	1,48	0,050
STE6	130,5	79,0	200,7	86,1	1,54	0,274
STE7	43,4	13,5	49,6	15,2	1,14	0,565
STF1	304,5	101,3	841,2	394,1	2,76	0,039
STF2	424,5	218,8	1882,1	1028,3	4,43	0,032
STH1	29,8	21,8	16,8	11,7	0,56	0,335
STI1	156,6	50,5	805,3	230,1	5,14	0,002
STL1	0,8	0,7	1,5	1,1	1,99	0,274
STM1	2696,0	1301,6	1953,0	1063,6	0,72	0,411
STN1	17,5	8,8	10,9	7,6	0,62	0,296
STO1	68,1	29,6	57,6	26,9	0,85	0,618
STP1	145,8	83,2	127,2	86,3	0,87	0,767
STP2	30,8	9,7	54,8	20,4	1,78	0,078
STP22	62,9	6,0	81,3	16,5	1,29	0,082
STP3	37,3	13,1	35,0	28,0	0,94	0,890
STP4	11,9	9,9	25,4	17,0	2,13	0,219
STR2	46,4	23,5	31,5	22,0	0,68	0,391
STR3	2,6	2,5	5,5	2,3	2,09	0,141
STS1	40,9	20,0	48,9	28,5	1,20	0,662
STT3	120,8	75,0	67,0	17,7	0,55	0,212
STT4	58,4	33,1	29,6	20,4	0,51	0,189
STU1	20,4	8,6	12,5	9,3	0,61	0,254
STU2	39,8	7,1	12,8	3,2	0,32	0,000
STV1	38,6	34,4	32,3	45,1	0,84	0,830
SUA5	104,9	15,5	36,8	25,9	0,35	0,004
SUA7	119,7	87,2	129,0	55,2	1,08	0,862
SUB1	130,1	73,0	237,1	95,0	1,82	0,125
SUB2	473,3	83,6	313,3	132,4	0,66	0,087
SUC2	40,7	9,1	54,4	27,9	1,34	0,387
SUE1	3,0	2,4	4,9	6,7	1,63	0,617
SUI1	1429,0	677,5	1633,4	267,6	1,14	0,595
SUI2	354,3	267,8	321,2	243,7	0,91	0,861
SUL1	8,8	5,1	16,0	18,1	1,82	0,473
SUL2	21,6	16,2	92,4	78,1	4,27	0,126
SUM1	290,6	190,5	212,5	90,9	0,73	0,487
SUN4	244,8	99,0	131,4	49,6	0,54	0,086
SUP35	298,9	144,9	330,1	112,8	1,10	0,745
SUP45	444,5	168,2	417,8	129,6	0,94	0,811
SUR1	2,2	1,0	2,0	1,8	0,92	0,861
SUR2	88,2	49,5	52,1	23,1	0,59	0,234

SUR4	852,7	261,9	450,9	63,7	0,53	0,025
SUR7	119,5	52,4	67,0	24,3	0,56	0,119
SUS1	138,2	57,4	80,1	11,8	0,58	0,095
SUT1	64,4	29,4	59,4	46,9	0,92	0,863
SUT2	59,8	20,0	12,6	9,1	0,21	0,005
SUV3	22,9	7,5	33,9	34,5	1,48	0,555
SVF1	294,0	114,3	197,7	58,4	0,67	0,184
SVL3	176,9	53,3	205,9	34,4	1,16	0,396
SVP26	164,1	79,3	126,3	63,4	0,77	0,484
SWA2	22,0	8,1	44,3	33,7	2,01	0,245
SWC3	24,6	14,4	18,8	16,6	0,76	0,618
SWC4	17,1	8,3	63,7	21,2	3,72	0,006
SWC5	31,5	28,8	9,3	6,7	0,30	0,184
SWC7	0,0	0,0	0,0	0,0	#DIV/0!	#DIV/0!
SWD1	10,3	4,4	42,7	18,3	4,14	0,014
SWD2	58,0	29,1	43,5	16,4	0,75	0,417
SWD3	30,4	18,7	23,3	18,1	0,77	0,607
SWE1	80,9	34,8	6,8	4,7	0,08	0,006
SWF1	11,6	4,7	30,7	9,0	2,64	0,009
SWH1	75,2	11,9	150,0	18,1	2,00	0,000
SWI1	12,2	6,7	2,0	1,8	0,17	0,026
SWI3	20,5	9,4	41,2	28,6	2,01	0,218
SWI4	150,5	85,7	23,4	15,7	0,16	0,027
SWI5	45,8	15,6	27,8	6,5	0,61	0,076
SWI6	60,5	21,5	41,9	9,0	0,69	0,161
SWM1	1,7	0,7	6,0	8,0	3,45	0,329
SWM2	16,5	8,6	34,4	30,7	2,09	0,303
SWP1	200,6	45,2	117,4	16,1	0,59	0,013
SWP82	67,8	24,0	27,2	10,6	0,40	0,021
SWR1	27,1	13,2	36,4	12,9	1,35	0,350
SWS2	93,4	55,6	87,2	19,5	0,93	0,839
SWT1	23,2	6,4	20,2	1,4	0,87	0,400
SWT21	6,0	3,8	6,9	2,2	1,14	0,718
SXM1	133,9	71,8	132,1	30,2	0,99	0,966
SYC1	6,8	3,3	7,1	4,9	1,04	0,922
SYF1	35,5	6,4	40,6	33,8	1,14	0,775
SYG1	111,0	53,3	85,3	42,4	0,77	0,480
SYH1	142,8	46,6	135,6	73,3	0,95	0,875
SYM1	15,0	7,7	53,8	15,1	3,60	0,004
SYN8	34,4	20,8	26,9	25,8	0,78	0,664
SYP1	54,2	24,9	64,8	33,3	1,20	0,627
SYS1	42,0	22,4	46,2	20,5	1,10	0,791
SYT1	35,9	14,0	29,9	15,9	0,83	0,594
TAD1	28,9	12,7	15,2	11,1	0,52	0,154
TAD2	25,0	14,0	7,6	5,1	0,30	0,058
TAD3	37,5	13,2	32,6	13,2	0,87	0,618
TAE1	81,0	57,0	68,8	39,0	0,85	0,737
TAE2	301,8	183,8	355,1	174,3	1,18	0,689
TAF1	51,1	19,3	59,9	28,3	1,17	0,627
TAF10	47,2	35,3	29,6	16,9	0,63	0,404

TAF11	261,7	103,1	128,5	78,4	0,49	0,085
TAF2	33,3	11,2	45,0	12,5	1,35	0,212
TAF3	35,7	6,8	29,5	16,5	0,82	0,508
TAF4	71,1	30,4	70,9	14,6	1,00	0,989
TAF5	63,0	32,2	86,7	41,5	1,38	0,402
TAF6	323,7	100,3	206,4	33,8	0,64	0,068
TAF7	14,5	4,5	19,2	13,9	1,32	0,542
TAF8	24,4	10,7	19,4	13,0	0,79	0,573
TAF9	284,9	132,5	255,7	140,1	0,90	0,772
TAH1	68,9	28,2	258,9	148,7	3,76	0,046
TAH11	49,7	21,9	104,6	44,9	2,10	0,071
TAH18	19,8	7,2	15,0	5,5	0,76	0,335
TAL1	807,0	437,4	635,9	198,5	0,79	0,503
TAM41	22,9	10,3	31,1	29,0	1,36	0,613
TAN1	77,2	38,9	41,0	29,1	0,53	0,187
TAO3	42,6	11,2	24,9	16,7	0,58	0,128
TAP42	20,7	10,7	34,1	27,6	1,64	0,401
TAR1	5,9	5,8	4,6	4,4	0,78	0,736
TAT1	86,4	49,8	23,0	7,9	0,27	0,045
TAT2	63,4	24,1	54,5	20,8	0,86	0,596
TAX4	4,9	1,3	8,5	5,7	1,73	0,265
TAZ1	34,9	18,9	27,5	23,7	0,79	0,642
TBF1	30,4	3,3	22,4	6,3	0,74	0,068
TBS1	11,3	3,3	9,3	6,3	0,82	0,593
TCB1	103,0	19,1	68,7	21,0	0,67	0,052
TCB2	212,6	92,0	98,8	42,1	0,46	0,065
TCB3	290,3	179,6	141,2	74,9	0,49	0,176
TCM62	24,9	20,6	27,2	14,1	1,10	0,856
TCO89	31,2	5,3	28,0	15,9	0,90	0,713
TCP1	373,6	274,7	363,3	198,6	0,97	0,953
TDH1	190,5	161,8	1329,1	835,5	6,98	0,037
TDH2	4086,6	317,8	4823,8	862,8	1,18	0,160
TDH3	11592,7	3820,8	16763,1	8521,3	1,45	0,311
TDP1	6,7	1,0	11,2	4,1	1,68	0,076
TEA1	24,6	9,0	49,1	23,6	1,99	0,101
TEC1	175,5	46,9	339,5	35,6	1,93	0,001
TED1	72,2	15,9	61,6	16,5	0,85	0,390
TEF2	25063,7	17867,4	21298,8	14039,5	0,85	0,752
TEF4	4381,7	2188,3	5020,1	2458,4	1,15	0,711
TEL1	19,5	5,1	12,6	8,8	0,64	0,220
TEL2	29,2	13,3	11,8	6,2	0,40	0,055
TEM1	53,8	19,9	51,0	9,4	0,95	0,808
TEN1	26,8	14,9	28,2	21,8	1,06	0,914
TEP1	3,8	2,3	19,5	32,5	5,12	0,372
TES1	17,9	24,3	8,4	5,7	0,47	0,475
TEX1	53,8	17,7	44,0	28,5	0,82	0,582
TFA1	111,8	84,2	146,9	118,2	1,31	0,645
TFA2	77,9	42,7	128,4	88,6	1,65	0,345
TFB1	79,8	65,9	79,4	68,5	1,00	0,995
TFB2	49,3	7,4	32,9	9,6	0,67	0,036

TFB3	126,3	32,8	161,2	23,5	1,28	0,135
TFB4	73,5	16,4	40,2	14,8	0,55	0,023
TFB5	119,6	57,8	80,4	34,8	0,67	0,290
TFC1	28,3	12,4	20,5	6,8	0,73	0,314
TFC3	39,7	15,9	29,0	20,7	0,73	0,443
TFC4	50,3	11,4	44,0	9,2	0,88	0,428
TFC6	29,1	4,5	23,7	3,5	0,81	0,107
TFC7	34,4	26,2	29,0	23,8	0,84	0,770
TFC8	22,3	12,1	12,1	3,2	0,54	0,153
TFG1	146,9	45,2	146,0	39,3	0,99	0,976
TFG2	48,0	20,0	31,1	21,3	0,65	0,291
TFS1	62,2	53,5	103,9	122,9	1,67	0,557
TGL1	81,4	20,4	36,1	24,2	0,44	0,028
TGL2	14,4	9,1	35,1	21,7	2,43	0,130
TGL3	22,4	8,0	36,8	8,0	1,64	0,044
TGL4	81,2	6,5	152,6	97,4	1,88	0,194
TGL5	88,5	39,2	94,8	19,7	1,07	0,782
TGS1	16,0	6,0	21,7	14,8	1,36	0,500
THG1	282,3	130,8	287,2	87,6	1,02	0,952
THI11	42,5	30,2	58,3	19,5	1,37	0,414
THI12	0,2	0,3	0,0	0,0	0,00	0,356
THI13	1,3	0,7	1,4	1,3	1,09	0,875
THI2	10,5	5,7	13,6	6,5	1,29	0,506
THI20	19,4	12,3	23,0	9,9	1,19	0,661
THI21	24,9	3,6	8,6	6,0	0,35	0,004
THI22	26,0	11,4	37,2	30,8	1,43	0,520
THI3	27,3	7,0	31,0	10,6	1,14	0,581
THI4	7,3	9,1	11,1	15,7	1,52	0,690
THI6	27,8	22,4	32,4	17,3	1,17	0,754
THI7	24,4	5,8	28,9	5,7	1,18	0,315
THI72	0,6	0,6	0,4	0,7	0,65	0,649
THI73	3,1	0,8	1,3	0,9	0,42	0,023
THI74	8,1	4,4	3,9	2,8	0,48	0,158
THI80	15,3	7,3	2,4	1,9	0,16	0,014
THO1	303,8	90,2	412,5	108,8	1,36	0,175
THO2	41,4	29,7	49,7	22,8	1,20	0,673
THP1	22,3	13,8	47,5	25,8	2,13	0,135
THP2	12,3	9,5	1,6	2,4	0,13	0,074
THP3	15,5	8,9	32,7	20,4	2,11	0,173
THR1	235,5	113,5	301,7	151,3	1,28	0,511
THR4	293,3	155,6	175,4	123,6	0,60	0,280
THS1	859,4	542,7	778,8	207,1	0,91	0,791
TID3	86,7	49,4	58,5	33,6	0,67	0,381
TIF2	1289,3	794,1	1822,2	976,5	1,41	0,430
TIF3	575,2	275,3	362,3	249,9	0,63	0,296
TIF34	669,4	275,8	859,8	264,5	1,28	0,357
TIF35	219,2	107,6	190,7	91,6	0,87	0,701
TIF4631	106,0	45,6	182,5	41,0	1,72	0,047
TIF4632	79,2	12,2	63,1	20,6	0,80	0,227
TIF5	325,8	297,8	313,4	219,2	0,96	0,949

TIF6	109,3	39,6	164,4	70,4	1,50	0,222
TIM10	808,1	540,2	574,5	376,4	0,71	0,505
TIM12	57,5	24,9	23,4	16,2	0,41	0,062
TIM17	283,2	134,6	308,6	148,7	1,09	0,809
TIM18	41,6	31,1	66,8	57,0	1,61	0,467
TIM21	39,4	9,5	47,9	23,4	1,22	0,527
TIM22	18,5	5,5	44,5	5,6	2,41	0,001
TIM23	178,3	86,4	244,5	155,6	1,37	0,485
TIM44	78,6	47,8	106,1	36,8	1,35	0,396
TIM50	532,0	67,5	526,3	168,3	0,99	0,952
TIM54	180,4	12,4	144,7	36,1	0,80	0,111
TIM9	214,4	148,4	236,0	133,5	1,10	0,836
TIP1	468,2	345,0	1439,2	739,9	3,07	0,055
TIP20	84,7	23,9	41,2	24,2	0,49	0,043
TIP41	27,6	9,1	21,4	3,7	0,77	0,249
TIR1	17,8	8,4	4,4	3,6	0,25	0,026
TIR2	18,7	9,3	10,0	8,1	0,54	0,210
TIR3	45,6	19,4	14,8	10,2	0,32	0,031
TIR4	4,8	1,3	8,4	10,6	1,75	0,526
TIS11	45,4	35,3	56,2	10,8	1,24	0,580
TKL1	824,7	580,7	998,7	677,5	1,21	0,710
TKL2	6,1	2,2	23,7	17,5	3,90	0,093
TLG1	158,3	51,1	152,3	42,3	0,96	0,863
TLG2	59,8	41,9	55,2	25,3	0,92	0,859
TMA10	84,2	27,3	283,6	150,6	3,37	0,040
TMA108	89,4	50,2	85,6	32,8	0,96	0,904
TMA17	68,3	15,0	173,4	55,1	2,54	0,010
TMA19	5104,5	1658,0	6243,3	2244,9	1,22	0,446
TMA20	358,9	233,9	307,3	174,6	0,86	0,736
TMA22	147,3	74,0	310,2	138,5	2,11	0,083
TMA23	54,2	15,0	100,9	38,0	1,86	0,062
TMA46	139,0	22,7	156,3	34,0	1,12	0,429
TMA64	34,2	10,0	15,1	1,4	0,44	0,009
TMN2	24,5	15,1	23,4	8,4	0,96	0,903
TMN3	32,2	16,6	39,7	41,9	1,23	0,752
TMS1	34,6	13,9	20,8	12,1	0,60	0,185
TMT1	46,4	8,7	142,8	45,6	3,08	0,006
TNA1	32,6	22,9	45,1	21,6	1,38	0,459
TOA1	61,8	15,7	44,7	14,5	0,72	0,160
TOA2	52,8	24,4	247,9	135,7	4,70	0,030
TOD6	52,6	23,0	40,0	17,4	0,76	0,415
TOF1	34,1	7,2	9,5	4,4	0,28	0,001
TOF2	53,0	27,6	41,8	17,3	0,79	0,515
TOK1	29,6	12,4	46,4	39,2	1,57	0,445
TOM1	94,0	19,5	67,0	4,5	0,71	0,036
TOM20	111,6	55,4	119,8	72,1	1,07	0,862
TOM22	181,2	55,5	157,0	73,7	0,87	0,618
TOM40	135,5	104,6	184,1	197,9	1,36	0,679
TOM6	202,1	51,4	154,4	75,0	0,76	0,335
TOM7	862,3	316,0	744,8	176,8	0,86	0,540

TOM70	316,3	200,0	378,9	194,9	1,20	0,670
TOM71	91,0	47,8	57,2	18,9	0,63	0,237
TOP1	264,5	132,2	110,6	16,1	0,42	0,060
TOP2	124,8	106,0	22,4	17,6	0,18	0,105
TOP3	1,4	1,0	0,3	0,4	0,24	0,085
TOR1	22,9	14,6	37,9	10,1	1,65	0,143
TOR2	43,5	15,4	40,0	15,1	0,92	0,754
TOS1	682,6	397,2	537,8	256,5	0,79	0,563
TOS2	15,9	1,4	1,7	1,5	0,11	0,000
TOS3	23,8	15,5	21,0	18,3	0,88	0,825
TOS4	53,7	15,0	2,5	2,4	0,05	0,001
TOS6	394,6	41,8	12,2	8,6	0,03	0,000
TOS8	12,1	8,1	13,4	9,8	1,11	0,843
TPA1	146,3	67,0	121,7	34,8	0,83	0,539
TPC1	29,7	9,4	32,2	16,2	1,08	0,798
TPD3	103,3	40,8	164,2	99,4	1,59	0,300
TPK1	27,7	5,5	52,7	10,5	1,90	0,006
TPK2	19,6	7,4	20,6	14,1	1,05	0,910
TPK3	150,7	10,7	134,8	53,7	0,89	0,584
TPM1	710,2	337,5	1058,3	335,3	1,49	0,194
TPM2	116,9	49,0	103,3	28,9	0,88	0,649
TPN1	99,6	53,2	58,8	31,7	0,59	0,236
TPO1	235,7	91,2	132,2	74,7	0,56	0,130
TPO2	39,7	33,3	183,5	90,5	4,62	0,025
TPO3	23,9	19,0	50,6	41,2	2,11	0,285
TPO4	37,6	11,3	54,2	24,0	1,44	0,258
TPO5	17,4	7,7	17,7	7,3	1,02	0,947
TPP1	17,6	10,6	23,1	16,2	1,31	0,593
TPS1	71,5	31,1	283,7	231,8	3,96	0,120
TPS2	91,3	39,3	302,0	287,8	3,31	0,197
TPS3	30,7	20,2	54,4	25,4	1,77	0,194
TPT1	73,4	32,6	36,8	7,7	0,50	0,072
TRA1	81,3	42,2	63,7	35,6	0,78	0,548
TRE1	36,1	21,8	27,8	2,0	0,77	0,476
TRE2	34,0	28,6	29,6	17,5	0,87	0,798
TRF5	33,2	26,0	19,6	16,8	0,59	0,411
TRI1	26,0	13,2	34,1	12,0	1,31	0,400
TRK1	61,9	20,9	66,2	24,4	1,07	0,799
TRK2	15,5	5,7	7,5	5,3	0,49	0,088
TRL1	91,9	32,1	37,3	14,5	0,41	0,021
TRM1	177,7	71,8	148,7	63,8	0,84	0,568
TRM10	83,4	19,1	38,3	25,7	0,46	0,031
TRM11	90,1	46,9	16,2	3,5	0,18	0,020
TRM112	210,7	45,9	123,5	64,5	0,59	0,070
TRM12	70,2	21,2	80,7	15,9	1,15	0,461
TRM13	63,7	23,2	96,2	70,6	1,51	0,416
TRM2	20,2	11,0	22,5	7,2	1,11	0,735
TRM3	105,4	24,1	103,8	80,4	0,99	0,972
TRM44	81,6	63,1	62,8	46,3	0,77	0,649
TRM5	30,5	20,1	49,1	23,6	1,61	0,275

TRM7	36,4	8,0	26,9	9,6	0,74	0,181
TRM8	53,0	6,5	39,9	10,3	0,75	0,075
TRM82	118,7	68,5	118,2	49,2	1,00	0,991
TRM9	44,0	10,7	22,1	15,0	0,50	0,055
TRP1	40,7	16,3	43,5	21,6	1,07	0,841
TRP2	125,0	25,5	148,9	30,2	1,19	0,273
TRP3	378,0	189,2	470,8	290,0	1,25	0,611
TRP4	83,0	27,0	38,4	26,3	0,46	0,056
TRP5	643,6	413,6	911,2	280,7	1,42	0,326
TRR1	400,0	57,5	766,7	202,7	1,92	0,013
TRS120	51,0	25,0	42,8	15,4	0,84	0,595
TRS130	15,3	9,2	20,7	5,5	1,35	0,351
TRS20	91,1	65,4	45,8	36,2	0,50	0,271
TRS23	20,4	3,6	33,3	6,9	1,63	0,016
TRS31	54,3	47,7	34,4	45,2	0,63	0,566
TRS33	96,9	28,5	49,8	11,6	0,51	0,022
TRX1	507,9	345,0	409,7	251,8	0,81	0,662
TRX2	1195,2	502,8	2143,6	1052,2	1,79	0,155
TRX3	12,6	19,5	16,1	28,9	1,28	0,847
TRZ1	88,5	16,3	48,3	21,5	0,55	0,025
TSA1	2864,2	1019,0	4472,8	979,7	1,56	0,063
TSA2	36,7	25,5	113,0	103,3	3,08	0,201
TSC10	86,7	42,2	62,3	22,9	0,72	0,349
TSC11	58,0	30,9	71,2	32,4	1,23	0,577
TSC13	181,2	57,3	153,0	26,6	0,84	0,406
TSC3	208,3	90,1	174,0	21,4	0,84	0,486
TSL1	58,1	19,1	184,2	95,4	3,17	0,041
TSR1	73,0	34,9	104,7	25,6	1,43	0,193
TSR2	14,8	5,2	9,8	8,7	0,66	0,360
TSR3	153,2	52,9	113,3	47,9	0,74	0,306
TSR4	62,6	31,1	66,3	43,3	1,06	0,893
TTI1	5,5	2,7	16,7	4,3	3,06	0,004
TTI2	29,1	9,3	12,3	8,5	0,42	0,037
TUB1	144,9	87,4	46,5	38,7	0,32	0,085
TUB2	515,0	188,4	212,8	119,7	0,41	0,035
TUB3	146,6	58,8	171,3	177,4	1,17	0,800
TUB4	81,5	67,9	16,6	12,1	0,20	0,109
TUF1	293,9	129,1	167,6	50,9	0,57	0,119
TUL1	16,6	6,3	15,7	10,8	0,94	0,888
TUM1	11,6	4,6	6,6	4,5	0,57	0,170
TUS1	16,9	4,3	15,8	8,7	0,93	0,819
TVP15	193,2	114,6	315,0	183,1	1,63	0,303
TVP23	109,2	52,5	76,4	36,3	0,70	0,342
TVP38	46,0	23,9	38,0	13,4	0,83	0,581
TWF1	57,9	35,0	95,1	59,7	1,64	0,324
TYE7	133,2	117,2	135,1	88,8	1,01	0,980
TYR1	91,3	39,9	83,8	27,2	0,92	0,768
TYS1	33,7	25,0	58,6	53,2	1,74	0,428
TYW1	73,9	45,1	94,1	48,0	1,27	0,561
TYW3	41,2	12,4	32,9	27,4	0,80	0,598

UAF30	72,4	41,1	60,8	13,9	0,84	0,612
UBA1	263,8	57,7	375,5	63,9	1,42	0,041
UBA2	89,3	13,0	101,5	25,1	1,14	0,422
UBA3	4,6	1,2	9,1	6,9	1,99	0,241
UBA4	66,6	10,8	120,0	22,5	1,80	0,005
UBC1	89,2	33,3	64,8	24,6	0,73	0,283
UBC12	27,1	15,2	36,0	35,5	1,33	0,658
UBC13	82,6	42,1	42,6	14,2	0,52	0,122
UBC4	855,2	931,9	787,6	744,2	0,92	0,913
UBC5	16,7	17,9	13,9	17,7	0,83	0,829
UBC6	33,9	21,1	31,5	15,8	0,93	0,861
UBC7	157,6	97,3	99,3	87,3	0,63	0,406
UBC8	46,9	25,9	75,5	30,2	1,61	0,201
UBC9	76,0	14,2	33,5	10,7	0,44	0,003
UBI4	0,2	0,2	0,1	0,3	0,78	0,837
UBP1	62,3	28,0	39,3	12,6	0,63	0,185
UBP10	86,6	35,1	61,1	41,0	0,71	0,382
UBP11	20,8	7,0	15,1	10,5	0,73	0,400
UBP12	114,5	81,4	92,7	58,0	0,81	0,679
UBP13	46,1	16,8	33,0	12,2	0,72	0,256
UBP14	50,6	10,1	60,4	18,9	1,19	0,397
UBP15	36,5	12,6	101,5	71,5	2,78	0,124
UBP16	10,9	9,7	10,3	9,5	0,95	0,940
UBP2	73,0	46,5	89,4	78,9	1,22	0,732
UBP3	119,9	42,3	145,7	40,7	1,22	0,413
UBP5	43,7	17,8	23,3	1,6	0,53	0,063
UBP6	98,2	20,4	102,0	52,6	1,04	0,898
UBP7	11,9	6,2	10,1	9,3	0,85	0,756
UBP8	9,8	5,3	17,0	6,8	1,73	0,147
UBP9	15,2	4,7	38,3	23,3	2,52	0,099
UBR1	43,7	33,8	68,5	21,3	1,57	0,261
UBR2	5,2	1,1	17,0	3,2	3,27	0,000
UBS1	66,0	30,6	43,3	13,3	0,66	0,223
UBX2	53,0	34,4	91,9	55,8	1,73	0,280
UBX3	29,1	11,0	30,9	12,9	1,06	0,839
UBX4	49,3	18,6	50,0	36,8	1,01	0,974
UBX5	19,2	6,9	44,8	28,0	2,33	0,127
UBX6	11,6	4,1	22,0	7,5	1,90	0,050
UBX7	11,2	2,8	29,2	8,0	2,61	0,005
UFD1	19,9	21,7	32,3	24,9	1,62	0,481
UFD2	21,7	20,1	14,5	12,1	0,67	0,562
UFD4	45,2	9,7	50,7	16,5	1,12	0,588
UFE1	101,7	27,7	90,4	37,3	0,89	0,643
UFO1	31,1	13,6	36,1	8,2	1,16	0,556
UGA1	87,4	28,4	155,5	74,1	1,78	0,137
UGA2	73,9	52,0	334,0	180,1	4,52	0,032
UGA3	22,0	8,1	18,4	17,5	0,83	0,716
UGA4	3,1	1,8	5,7	4,2	1,84	0,298
UGO1	37,7	21,2	34,8	20,4	0,92	0,853
UGP1	92,1	55,5	153,8	27,7	1,67	0,094

UGX2	12,1	5,4	21,8	1,2	1,80	0,013
UIP3	96,0	64,0	129,8	91,2	1,35	0,566
UIP4	4,2	1,6	16,5	3,1	3,95	0,000
UIP5	22,0	6,2	16,6	11,9	0,75	0,448
ULA1	10,5	7,7	2,5	1,8	0,24	0,091
ULI1	12,2	1,9	16,8	3,4	1,38	0,056
ULP2	27,2	12,8	44,0	11,9	1,62	0,103
ULS1	23,8	7,0	36,3	20,3	1,53	0,287
UME1	19,7	9,0	23,9	13,9	1,21	0,629
UME6	211,5	92,9	129,3	65,0	0,61	0,197
UMP1	82,8	18,6	111,7	22,9	1,35	0,098
UNG1	38,6	13,5	12,3	13,3	0,32	0,032
UPC2	9,4	5,0	5,9	4,9	0,63	0,352
UPF3	53,1	24,7	87,7	40,3	1,65	0,195
UPS1	71,7	44,1	126,4	108,5	1,76	0,387
UPS2	84,4	46,9	48,0	10,5	0,57	0,180
UPS3	6,4	5,4	1,4	1,0	0,22	0,124
URA1	609,9	192,5	930,9	179,5	1,53	0,051
URA10	24,3	13,0	105,5	57,7	4,34	0,033
URA2	351,4	119,7	570,0	263,7	1,62	0,182
URA3	0,6	1,0	0,0	0,0	0,00	0,285
URA4	326,1	118,8	338,0	97,3	1,04	0,881
URA5	492,2	231,6	498,0	196,5	1,01	0,971
URA6	276,2	90,8	226,9	52,5	0,82	0,383
URA7	321,2	203,0	157,2	74,2	0,49	0,180
URA8	54,2	17,6	83,3	15,7	1,54	0,048
URB1	27,2	22,9	35,9	20,1	1,32	0,587
URB2	64,7	35,4	40,0	29,7	0,62	0,325
URC2	32,8	6,9	58,2	38,6	1,77	0,243
URE2	36,6	28,3	63,8	31,3	1,74	0,246
URH1	195,0	97,2	76,7	32,0	0,39	0,060
URK1	19,9	11,4	18,1	11,8	0,91	0,834
URM1	140,5	153,6	173,0	186,2	1,23	0,797
USA1	22,2	18,2	45,5	56,3	2,05	0,461
USE1	18,6	11,4	51,0	6,8	2,74	0,003
USO1	113,9	60,7	53,8	36,3	0,47	0,140
USV1	7,6	3,9	5,1	3,8	0,67	0,395
UTH1	1679,1	689,8	4276,4	1975,7	2,55	0,048
UTP10	40,5	10,3	49,0	11,4	1,21	0,311
UTP11	201,1	137,7	84,7	19,8	0,42	0,145
UTP13	30,9	23,1	18,4	15,1	0,60	0,401
UTP14	129,5	54,4	142,5	38,9	1,10	0,710
UTP15	25,8	12,9	23,3	18,6	0,90	0,832
UTP18	43,3	32,6	80,3	67,5	1,86	0,361
UTP20	151,8	111,8	76,6	46,6	0,50	0,261
UTP21	97,3	71,5	79,3	60,4	0,81	0,713
UTP22	88,3	43,7	74,6	45,0	0,84	0,677
UTP23	97,2	63,5	35,5	12,8	0,36	0,105
UTP25	76,6	55,0	88,3	22,8	1,15	0,709
UTP30	31,7	14,0	12,3	3,5	0,39	0,036

UTP4	38,1	17,3	30,2	22,8	0,79	0,598
UTP5	135,8	20,7	107,0	68,9	0,79	0,454
UTP6	60,9	36,9	44,8	24,7	0,74	0,496
UTP7	121,0	90,3	115,6	83,0	0,96	0,932
UTP8	109,8	46,6	121,1	34,9	1,10	0,712
UTP9	130,0	50,3	59,6	45,4	0,46	0,083
UTR1	115,2	62,8	149,2	81,2	1,30	0,532
UTR2	351,2	182,6	232,7	115,6	0,66	0,315
UTR4	14,7	5,5	14,5	5,9	0,98	0,952
UTR5	0,0	0,0	0,4	0,4	#DIV/0!	0,052
VAB2	41,7	13,1	63,1	14,3	1,51	0,069
VAC14	124,6	24,2	85,5	15,7	0,69	0,035
VAC17	12,0	3,8	3,9	2,7	0,33	0,013
VAC7	24,0	4,4	37,4	19,0	1,56	0,218
VAC8	58,1	39,9	40,6	24,7	0,70	0,483
VAM10	0,3	0,3	0,2	0,2	0,69	0,691
VAM3	43,6	21,7	36,7	20,7	0,84	0,663
VAM6	27,7	11,9	27,8	17,1	1,00	0,997
VAM7	1,8	0,9	1,1	1,0	0,60	0,340
VAN1	127,0	81,3	169,8	114,7	1,34	0,565
VAS1	549,7	248,3	380,6	125,4	0,69	0,270
VBA1	65,9	35,1	57,6	6,6	0,87	0,658
VBA2	21,1	6,5	35,6	9,5	1,69	0,045
VBA3	0,0	0,0	0,2	0,2	#DIV/0!	0,157
VBA4	150,3	66,1	43,5	29,1	0,29	0,025
VBA5	0,8	0,7	0,1	0,3	0,17	0,121
VCX1	118,7	39,5	75,5	28,0	0,64	0,125
VEL1	1,1	0,8	0,9	1,0	0,79	0,724
VHR1	30,6	14,3	52,5	24,3	1,72	0,171
VHS1	9,6	6,8	14,5	10,1	1,51	0,450
VHS2	21,6	1,6	24,8	2,7	1,15	0,087
VHS3	51,4	48,1	116,5	86,0	2,26	0,235
VHT1	303,8	249,8	275,6	245,0	0,91	0,877
VID22	66,4	53,8	31,7	17,0	0,48	0,264
VID24	70,5	31,4	69,3	22,6	0,98	0,955
VID27	43,5	8,8	19,6	5,2	0,45	0,003
VID28	39,1	15,3	73,2	21,8	1,87	0,043
VID30	55,6	30,1	85,1	32,3	1,53	0,229
VIK1	23,7	10,3	13,1	8,8	0,55	0,170
VIP1	75,9	76,4	95,7	88,8	1,26	0,747
VMA1	912,7	341,0	1327,2	387,2	1,45	0,159
VMA10	0,5	0,5	0,6	0,5	1,22	0,763
VMA11	828,9	421,9	644,9	266,4	0,78	0,488
VMA13	557,9	252,0	821,4	406,7	1,47	0,313
VMA16	224,5	84,1	179,3	54,5	0,80	0,403
VMA2	1558,2	231,6	1838,1	320,6	1,18	0,207
VMA22	16,8	9,0	7,8	7,1	0,46	0,164
VMA4	413,7	212,9	490,3	209,0	1,19	0,626
VMA5	251,3	61,4	171,0	85,9	0,68	0,179
VMA6	398,1	229,6	179,9	112,0	0,45	0,138

VMA8	21,8	11,9	2,5	1,8	0,12	0,019
VMA9	927,0	326,9	651,2	67,3	0,70	0,150
VMR1	48,3	46,4	49,1	21,8	1,02	0,975
VMS1	23,1	8,0	27,2	17,1	1,18	0,676
VNX1	37,3	9,7	15,2	10,6	0,41	0,022
VOA1	248,5	167,5	216,1	113,2	0,87	0,760
VPH1	435,9	251,4	407,6	272,5	0,94	0,884
VPH2	182,5	55,3	89,4	43,6	0,49	0,038
VPS1	208,3	128,9	128,3	34,0	0,62	0,275
VPS13	92,4	28,7	78,9	23,0	0,85	0,491
VPS15	21,4	2,9	32,3	3,3	1,51	0,002
VPS16	31,4	9,9	40,1	7,1	1,28	0,204
VPS17	116,0	28,6	117,3	62,2	1,01	0,972
VPS20	41,8	12,0	56,5	11,6	1,35	0,129
VPS21	82,5	17,8	138,3	9,2	1,68	0,001
VPS24	61,2	15,5	84,2	23,2	1,38	0,150
VPS25	57,5	53,7	44,1	36,3	0,77	0,692
VPS27	40,0	10,8	50,3	8,6	1,26	0,189
VPS28	36,3	24,7	50,4	45,9	1,39	0,609
VPS29	78,8	32,3	25,3	5,7	0,32	0,017
VPS3	25,8	21,0	24,7	12,4	0,96	0,928
VPS30	21,5	11,1	26,5	14,5	1,23	0,605
VPS33	27,0	13,1	29,6	4,3	1,10	0,714
VPS34	33,2	10,7	28,6	8,5	0,86	0,526
VPS35	66,4	53,9	113,4	90,2	1,71	0,405
VPS36	48,4	45,5	59,1	52,0	1,22	0,767
VPS38	61,3	19,4	55,0	9,6	0,90	0,585
VPS4	140,5	119,8	197,5	175,5	1,41	0,611
VPS41	58,3	35,3	67,9	17,3	1,16	0,643
VPS45	25,5	8,5	31,6	7,9	1,24	0,326
VPS5	38,8	14,9	64,0	36,6	1,65	0,249
VPS52	63,8	37,1	58,2	19,2	0,91	0,798
VPS53	89,0	8,0	64,2	22,6	0,72	0,084
VPS54	56,2	28,1	46,0	14,9	0,82	0,545
VPS55	26,4	12,9	11,2	3,4	0,42	0,063
VPS60	86,9	29,1	80,8	21,6	0,93	0,749
VPS61	0,3	0,5	0,0	0,1	0,13	0,302
VPS62	62,9	31,4	60,5	43,2	0,96	0,933
VPS63	131,1	68,7	99,4	38,1	0,76	0,450
VPS64	28,4	15,4	26,8	9,9	0,95	0,871
VPS65	2,1	1,0	1,5	1,5	0,70	0,514
VPS66	72,1	30,0	52,4	15,2	0,73	0,285
VPS68	127,1	70,2	152,4	78,3	1,20	0,648
VPS69	2,5	1,4	2,5	2,7	0,99	0,990
VPS70	31,9	12,8	75,6	55,2	2,37	0,175
VPS71	62,4	33,9	69,2	23,0	1,11	0,750
VPS72	40,2	24,7	41,7	27,8	1,04	0,940
VPS73	122,7	66,1	121,4	55,3	0,99	0,977
VPS74	125,8	39,6	102,1	19,8	0,81	0,325
VPS75	48,7	19,3	48,6	5,9	1,00	0,998

VPS8	31,5	10,6	37,1	20,4	1,18	0,646
VPS9	34,6	12,2	36,9	8,3	1,07	0,764
VRG4	261,4	68,1	115,7	60,2	0,44	0,018
VRP1	23,1	11,5	41,7	30,4	1,81	0,297
VTA1	43,7	16,1	45,5	20,2	1,04	0,893
VTC1	712,8	436,6	896,0	290,5	1,26	0,511
VTC2	260,2	48,4	551,8	79,6	2,12	0,001
VTC3	107,3	77,5	530,8	297,4	4,95	0,033
VTC4	74,0	37,2	256,5	70,4	3,46	0,004
VTH1	5,2	4,6	17,9	13,6	3,43	0,127
VTI1	92,1	33,4	189,2	56,4	2,05	0,025
VTS1	33,3	15,2	17,8	1,6	0,53	0,089
WAR1	21,7	4,6	33,8	4,5	1,56	0,009
WBP1	174,9	73,5	182,0	80,5	1,04	0,902
WHI2	80,3	41,1	32,8	19,7	0,41	0,082
WHI3	47,8	6,1	62,2	55,8	1,30	0,626
WHI4	157,8	26,9	214,5	36,6	1,36	0,047
WHI5	2,2	1,7	1,9	1,3	0,87	0,802
WRS1	383,1	100,7	362,2	86,7	0,95	0,764
WSC2	216,6	93,0	43,6	6,6	0,20	0,010
WSC3	62,7	30,9	24,4	1,3	0,39	0,048
WSC4	7,4	2,6	7,6	5,8	1,02	0,967
WSS1	17,2	5,5	19,5	3,4	1,14	0,499
WTM1	258,0	119,3	367,7	212,0	1,43	0,402
WTM2	11,8	12,4	15,0	7,5	1,27	0,675
WWM1	163,3	79,6	198,3	24,9	1,21	0,432
XBP1	8,7	3,3	10,4	9,6	1,19	0,755
XDJ1	36,1	22,7	31,1	17,2	0,86	0,737
XKS1	61,2	3,7	62,4	26,0	1,02	0,930
XPT1	109,5	50,1	134,0	95,7	1,22	0,667
XRS2	7,9	6,2	8,4	6,2	1,06	0,911
XYL2	4,9	0,2	8,0	5,2	1,66	0,271
YAE1	35,1	5,4	25,8	17,4	0,73	0,346
YAF9	34,0	9,8	45,0	6,1	1,33	0,103
YAH1	170,9	107,4	102,6	65,3	0,60	0,319
YAK1	32,4	8,1	46,4	17,1	1,43	0,190
YAL004W	6,6	4,7	10,4	9,0	1,59	0,475
YAL016C-A	1,2	0,8	0,6	0,4	0,45	0,183
YAL018C	0,1	0,1	0,2	0,4	1,87	0,620
YAL019W-A	0,4	0,4	0,5	0,6	1,30	0,751
YAL026C-A	0,9	0,7	0,7	0,9	0,84	0,806
YAL031W-A	1,2	0,4	1,6	1,8	1,32	0,690
YAL034C-B	6,5	5,8	5,1	4,9	0,79	0,734
YAL037C-A	617,0	362,4	937,7	558,6	1,52	0,373
YAL037C-B	111,9	117,8	109,5	93,6	0,98	0,976
YAL042C-A	1,1	0,7	0,7	1,0	0,64	0,528
YAL044W-A	120,6	77,9	134,5	68,9	1,12	0,798
YAL045C	2,4	2,2	0,8	1,3	0,32	0,239
YAL056C-A	4,8	3,1	5,1	4,5	1,06	0,920
YAL059C-A	3,7	2,4	1,9	2,0	0,51	0,288

YAL063C-A	3,9	0,9	2,3	1,9	0,58	0,154
YAL064C-A	0,0	0,0	0,2	0,5	#DIV/0!	0,356
YAL064W	0,4	0,4	0,2	0,3	0,40	0,414
YAL064W-B	1,1	0,9	0,4	0,3	0,39	0,205
YAL065C	0,1	0,2	0,2	0,2	2,00	0,553
YAL066W	0,5	0,8	0,1	0,2	0,22	0,426
YAP1	66,6	33,2	67,3	25,0	1,01	0,973
YAP1801	63,0	18,8	77,9	14,2	1,23	0,255
YAP1802	85,6	27,9	66,2	31,0	0,77	0,388
YAP3	32,2	14,1	46,7	27,9	1,45	0,390
YAP5	11,6	5,6	32,4	7,3	2,79	0,004
YAP6	12,3	8,0	52,3	15,7	4,26	0,004
YAP7	3,4	0,9	2,4	1,7	0,70	0,329
YAR009C	18202,6	12792,7	25336,0	13041,0	1,39	0,465
YAR010C	280,1	181,9	994,0	527,7	3,55	0,043
YAR023C	8,1	7,7	13,2	3,7	1,63	0,280
YAR028W	38,8	32,7	217,7	176,2	5,61	0,093
YAR030C	5,5	3,5	7,7	9,9	1,40	0,691
YAR035C-A	0,2	0,3	0,1	0,3	0,53	0,603
YAR047C	0,7	0,6	0,4	0,3	0,56	0,391
YAR053W	5,0	1,6	4,2	2,8	0,84	0,629
YAR060C	0,0	0,1	0,0	0,0	0,00	0,356
YAR061W	0,0	0,0	0,0	0,0	#DIV/0!	#DIV/0!
YAR062W	0,0	0,0	0,0	0,0	#DIV/0!	#DIV/0!
YAR068W	35,1	21,6	7,8	6,2	0,22	0,051
YAR069C	0,7	1,3	10,8	17,3	15,78	0,288
YAR070C	0,0	0,0	0,1	0,1	#DIV/0!	0,356
YAR075W	27,8	7,5	22,8	7,9	0,82	0,395
YAR1	217,7	28,5	355,1	102,6	1,63	0,042
YAT1	7,7	6,1	3,9	3,4	0,51	0,321
YAT2	22,4	10,0	79,2	27,4	3,54	0,008
YBL005W-A	8,8	6,6	15,3	13,3	1,75	0,413
YBL005W-B	1495,6	714,5	7106,1	1942,6	4,75	0,002
YBL006W-A	6,1	4,8	9,6	6,7	1,58	0,425
YBL008W-A	0,0	0,0	0,0	0,0	#DIV/0!	#DIV/0!
YBL010C	38,9	21,2	13,7	10,7	0,35	0,079
YBL012C	0,8	1,0	0,0	0,0	0,00	0,156
YBL028C	84,1	38,8	103,5	37,2	1,23	0,497
YBL029W	17,5	7,9	13,7	10,0	0,78	0,574
YBL036C	80,7	53,5	98,4	68,3	1,22	0,697
YBL039C-A	0,4	0,5	0,0	0,0	0,00	0,142
YBL039W-B	40,5	29,6	65,7	31,3	1,62	0,287
YBL044W	5,9	5,6	1,3	1,2	0,22	0,158
YBL053W	2,0	1,8	1,3	0,9	0,63	0,479
YBL055C	25,4	27,3	12,7	9,2	0,50	0,411
YBL059W	25,2	23,0	42,6	58,5	1,69	0,598
YBL062W	16,2	10,7	18,4	11,4	1,14	0,782
YBL065W	0,4	0,6	0,8	0,8	2,30	0,401
YBL068W-A	26,8	11,1	33,0	3,6	1,23	0,331
YBL070C	5,7	5,0	3,1	3,3	0,55	0,421

YBL071C	1,1	0,9	0,6	0,7	0,52	0,351
YBL073W	0,0	0,0	6,3	12,6	#DIV/0!	0,353
YBL077W	56,4	31,1	30,8	10,9	0,55	0,172
YBL081W	78,3	19,2	44,7	16,9	0,57	0,039
YBL086C	7,0	5,0	6,1	4,5	0,87	0,798
YBL094C	0,6	0,6	0,0	0,0	0,00	0,091
YBL095W	41,5	14,2	91,9	33,6	2,21	0,033
YBL096C	0,0	0,0	0,0	0,0	#DIV/0!	#DIV/0!
YBL100W-A	18,2	5,9	83,7	28,4	4,60	0,004
YBL100W-B	3719,0	1437,8	6173,6	1363,3	1,66	0,048
YBL104C	42,3	7,8	38,6	20,9	0,91	0,746
YBL107C	20,8	10,2	19,2	13,0	0,92	0,853
YBL108W	0,7	1,0	0,0	0,0	0,00	0,211
YBL109W	0,9	0,8	0,0	0,1	0,05	0,060
YBL111C	45,4	26,5	20,1	13,5	0,44	0,140
YBL112C	27,9	23,2	11,4	10,4	0,41	0,244
YBL113C	18,0	13,3	17,3	18,3	0,96	0,950
YBL113W-A	1,2	1,5	0,2	0,3	0,13	0,224
YBP1	41,3	16,5	60,5	4,9	1,46	0,068
YBP2	20,8	11,5	36,2	29,4	1,74	0,367
YBR012C	136,5	141,0	173,8	143,5	1,27	0,724
YBR012W-A	7,5	2,1	5,8	2,7	0,77	0,353
YBR012W-B	9,1	3,7	34,2	13,4	3,75	0,011
YBR013C	43,6	19,9	19,3	9,1	0,44	0,067
YBR016W	361,0	89,1	242,1	57,5	0,67	0,066
YBR027C	2,1	2,2	0,3	0,4	0,17	0,182
YBR028C	106,6	77,9	56,8	32,8	0,53	0,284
YBR032W	2,9	3,9	0,4	0,3	0,15	0,252
YBR051W	0,3	0,3	0,2	0,4	0,81	0,846
YBR053C	230,2	77,6	640,1	91,5	2,78	0,000
YBR056W	66,7	62,9	146,1	147,7	2,19	0,361
YBR056W-A	12,7	8,0	8,2	5,6	0,64	0,392
YBR062C	48,6	9,6	94,9	63,9	1,95	0,202
YBR063C	23,2	13,1	66,1	67,4	2,85	0,258
YBR064W	0,5	0,3	0,9	0,8	1,84	0,389
YBR071W	21,8	8,9	4,5	3,9	0,21	0,012
YBR074W	56,1	29,7	74,6	49,7	1,33	0,546
YBR076C-A	20,4	12,8	7,7	5,1	0,38	0,114
YBR085C-A	46,4	18,8	442,6	180,4	9,55	0,005
YBR089W	17,0	19,8	0,7	0,8	0,04	0,149
YBR090C	16,6	22,7	4,3	3,7	0,26	0,327
YBR096W	61,7	15,8	40,3	11,7	0,65	0,071
YBR099C	14,5	3,5	8,0	2,8	0,55	0,028
YBR103C-A	1,6	1,0	1,0	1,2	0,64	0,501
YBR116C	0,2	0,3	0,1	0,2	0,49	0,666
YBR124W	1,6	1,4	0,5	0,5	0,28	0,166
YBR126W-A	2071,7	616,3	2268,7	1034,1	1,10	0,755
YBR134W	0,0	0,0	0,2	0,4	#DIV/0!	0,275
YBR137W	35,0	10,0	38,0	1,7	1,09	0,567
YBR138C	9,9	3,4	5,8	4,7	0,58	0,202

YBR139W	39,0	32,4	74,5	63,4	1,91	0,357
YBR141C	25,8	14,3	20,8	14,5	0,81	0,645
YBR141W-A	6,0	2,9	2,7	1,9	0,45	0,103
YBR144C	1,7	0,7	7,6	7,9	4,37	0,190
YBR174C	1,8	1,1	2,4	2,0	1,33	0,621
YBR178W	0,2	0,2	0,9	1,1	5,58	0,217
YBR182C-A	0,1	0,2	0,1	0,2	0,74	0,844
YBR184W	14,9	9,2	9,9	9,8	0,66	0,481
YBR190W	0,1	0,1	0,1	0,3	2,35	0,616
YBR191W-A	1,0	0,8	0,3	0,4	0,28	0,158
YBR196C-B	4,4	3,4	1,1	1,5	0,25	0,128
YBR197C	13,8	9,0	18,3	12,8	1,32	0,588
YBR201C-A	5,9	1,4	3,5	2,5	0,60	0,151
YBR204C	17,7	12,3	26,5	20,2	1,50	0,484
YBR206W	86,4	51,0	143,3	63,2	1,66	0,211
YBR209W	1,9	3,5	0,1	0,1	0,03	0,328
YBR219C	67,8	19,6	32,9	11,1	0,49	0,021
YBR220C	1,0	0,9	0,5	0,9	0,44	0,374
YBR221W-A	15,7	10,1	13,0	10,0	0,83	0,715
YBR223W-A	0,1	0,1	0,1	0,1	1,07	0,963
YBR224W	0,8	0,3	0,8	0,7	1,06	0,915
YBR225W	26,0	11,7	64,2	46,7	2,47	0,164
YBR226C	0,4	0,4	0,2	0,4	0,61	0,579
YBR230W-A	143,2	58,9	286,2	32,8	2,00	0,005
YBR232C	0,0	0,0	0,3	0,2	#DIV/0!	0,044
YBR235W	46,6	6,5	31,6	11,0	0,68	0,057
YBR238C	3,1	2,3	4,4	3,5	1,43	0,557
YBR241C	19,2	5,2	22,6	4,2	1,18	0,348
YBR242W	41,3	25,4	23,3	9,3	0,56	0,231
YBR255C-A	64,2	18,6	34,7	3,3	0,54	0,020
YBR259W	17,6	1,1	32,8	17,5	1,86	0,135
YBR271W	72,2	19,1	26,7	18,4	0,37	0,014
YBR277C	1,4	2,5	0,3	0,4	0,23	0,430
YBR284W	9,5	5,3	6,7	8,0	0,70	0,575
YBR285W	6,1	4,9	32,7	42,9	5,34	0,264
YBR287W	98,9	53,5	216,1	43,9	2,18	0,015
YBR292C	22,7	18,7	36,6	22,4	1,61	0,379
YBR298C-A	0,8	1,6	0,0	0,0	0,00	0,356
YBT1	916,6	300,0	405,7	170,7	0,44	0,025
YCF1	103,6	40,2	85,0	53,9	0,82	0,601
YCG1	49,7	26,4	24,3	5,0	0,49	0,109
YCH1	20,5	4,7	28,0	16,1	1,36	0,408
YCK1	67,1	25,1	44,8	18,8	0,67	0,205
YCK2	79,3	36,4	56,6	35,0	0,71	0,404
YCK3	41,1	12,4	28,2	19,3	0,69	0,305
YCL001W-B	0,6	0,5	5,3	9,0	8,45	0,340
YCL002C	42,5	20,0	21,5	6,1	0,51	0,092
YCL007C	19,4	11,0	9,8	10,2	0,51	0,251
YCL019W	5,7	4,7	4,9	5,6	0,87	0,844
YCL021W-A	12,1	7,9	8,5	4,1	0,70	0,446

YCL022C	4,6	3,6	0,3	0,3	0,06	0,056
YCL023C	3,9	6,1	0,7	0,8	0,17	0,332
YCL041C	3,7	1,6	2,9	2,2	0,78	0,573
YCL047C	52,9	44,2	69,9	59,0	1,32	0,661
YCL048W-A	0,8	0,7	1,2	2,4	1,56	0,742
YCL049C	5,6	2,5	1,8	1,2	0,33	0,039
YCL057C-A	328,0	259,8	463,5	322,7	1,41	0,537
YCL065W	0,3	0,5	0,0	0,0	0,00	0,356
YCL068C	0,1	0,2	0,2	0,4	1,88	0,653
YCL073C	0,1	0,2	0,0	0,0	0,00	0,356
YCL074W	0,0	0,0	0,2	0,4	#DIV/0!	0,356
YCP4	319,1	113,2	335,0	105,1	1,05	0,843
YCR001W	0,3	0,3	0,7	0,8	2,10	0,420
YCR006C	0,5	0,9	1,1	1,0	2,01	0,444
YCR007C	7,8	4,1	23,1	7,8	2,97	0,013
YCR013C	197,8	165,2	394,1	253,5	1,99	0,242
YCR015C	16,8	11,1	40,2	24,2	2,39	0,130
YCR018C-A	17,6	7,9	6,7	5,8	0,38	0,067
YCR022C	1,1	0,8	0,3	0,3	0,24	0,103
YCR023C	109,9	19,6	63,6	15,0	0,58	0,010
YCR024C-B	1376,6	399,8	2624,3	562,7	1,91	0,011
YCR041W	2,4	1,5	1,6	1,6	0,66	0,487
YCR043C	58,0	24,8	24,0	18,3	0,41	0,069
YCR045W-A	1,5	2,3	0,2	0,4	0,16	0,333
YCR047W-A	0,8	0,9	0,5	0,6	0,71	0,683
YCR049C	0,2	0,3	0,0	0,0	0,00	0,134
YCR050C	2,2	1,3	0,6	0,6	0,28	0,068
YCR051W	102,7	88,3	153,2	120,9	1,49	0,525
YCR061W	2,0	1,5	14,1	13,7	6,95	0,132
YCR064C	0,8	0,3	0,4	0,6	0,45	0,252
YCR076C	63,5	17,1	56,2	36,1	0,88	0,726
YCR081C-A	0,3	0,3	3,8	6,6	12,05	0,333
YCR087C-A	45,4	9,3	41,1	12,8	0,91	0,606
YCR087W	3,1	2,0	2,3	2,8	0,74	0,645
YCR090C	152,6	69,9	121,0	102,2	0,79	0,628
YCR095W-A	0,7	0,8	1,5	1,1	2,07	0,300
YCR099C	7,4	2,9	9,2	6,7	1,24	0,648
YCR100C	0,2	0,4	0,2	0,4	0,81	0,874
YCR101C	0,1	0,2	0,4	0,6	3,59	0,384
YCR102C	3,0	1,3	3,7	2,6	1,23	0,651
YCR102W-A	0,1	0,1	0,7	0,6	8,29	0,096
YCS4	45,1	27,5	12,8	9,0	0,28	0,067
YCT1	21,9	3,3	38,4	9,4	1,75	0,016
YDC1	73,9	44,7	176,3	48,6	2,38	0,021
YDJ1	358,2	203,1	316,7	97,2	0,88	0,725
YDL009C	4,2	4,0	2,0	2,3	0,48	0,375
YDL012C	131,0	42,0	106,7	12,3	0,81	0,311
YDL016C	0,6	0,7	0,3	0,3	0,55	0,511
YDL022C-A	0,0	0,0	0,0	0,0	#DIV/0!	#DIV/0!
YDL023C	0,1	0,2	0,0	0,0	0,00	0,356

YDL026W	0,7	0,8	5,6	8,9	8,24	0,316
YDL027C	69,3	21,4	117,3	22,2	1,69	0,021
YDL034W	1,6	1,5	1,0	1,1	0,59	0,510
YDL041W	0,0	0,0	0,0	0,0	#DIV/0!	#DIV/0!
YDL050C	17,7	16,2	10,8	11,4	0,61	0,515
YDL057W	21,9	13,1	42,2	28,6	1,93	0,244
YDL063C	9,8	7,4	5,2	5,3	0,53	0,351
YDL068W	1,3	1,2	0,4	0,3	0,34	0,222
YDL073W	49,7	20,2	37,0	19,6	0,74	0,402
YDL085C-A	44,0	30,4	88,2	50,7	2,00	0,186
YDL086C-A	6,1	4,6	6,5	5,9	1,07	0,918
YDL086W	97,6	84,8	124,3	77,6	1,27	0,659
YDL094C	48,0	29,2	10,6	7,2	0,22	0,047
YDL109C	0,5	0,7	0,3	0,3	0,57	0,596
YDL114W	0,3	0,4	0,2	0,5	0,74	0,799
YDL118W	0,0	0,1	0,0	0,1	0,87	0,928
YDL119C	24,5	10,3	37,3	16,6	1,52	0,239
YDL121C	126,7	88,6	99,7	55,6	0,79	0,624
YDL124W	176,4	111,5	592,3	601,3	3,36	0,223
YDL129W	12,0	4,1	10,9	7,8	0,91	0,808
YDL133W	57,0	29,6	40,8	25,1	0,72	0,436
YDL144C	52,5	9,0	85,4	62,3	1,63	0,336
YDL156W	15,4	7,9	8,7	6,1	0,57	0,228
YDL157C	63,4	24,5	36,0	12,1	0,57	0,092
YDL158C	79,3	35,8	21,9	4,8	0,28	0,019
YDL159C-B	0,2	0,3	0,6	0,9	3,62	0,404
YDL159W-A	0,0	0,1	0,1	0,1	1,75	0,723
YDL160C-A	57,5	21,3	43,4	14,5	0,76	0,317
YDL162C	0,0	0,0	0,0	0,0	#DIV/0!	#DIV/0!
YDL163W	0,3	0,7	0,0	0,0	0,00	0,356
YDL172C	9,5	10,1	11,4	12,0	1,20	0,820
YDL176W	28,2	3,9	34,2	18,0	1,21	0,535
YDL177C	23,2	5,4	35,3	19,0	1,52	0,264
YDL180W	40,8	28,9	43,0	30,7	1,05	0,920
YDL183C	112,2	63,6	109,2	48,5	0,97	0,943
YDL186W	0,1	0,3	1,0	1,2	7,38	0,212
YDL199C	21,9	8,5	36,3	24,5	1,65	0,310
YDL206W	22,6	30,9	12,3	5,2	0,54	0,535
YDL211C	11,5	6,4	1,1	0,8	0,09	0,017
YDL218W	1,4	0,3	3,3	2,3	2,30	0,155
YDL221W	1,8	1,3	0,3	0,3	0,15	0,060
YDL228C	216,1	203,3	198,9	170,6	0,92	0,901
YDL233W	22,1	26,2	23,0	25,9	1,04	0,965
YDL241W	31,6	8,4	36,1	21,1	1,14	0,705
YDL242W	0,0	0,0	0,0	0,0	#DIV/0!	#DIV/0!
YDL247W-A	1,5	1,3	0,0	0,1	0,03	0,071
YDR008C	5,5	1,7	1,2	0,8	0,22	0,004
YDR010C	1,2	0,6	0,6	0,8	0,48	0,289
YDR018C	5,5	2,1	12,7	7,5	2,30	0,117
YDR026C	58,6	26,2	65,2	35,1	1,11	0,774

YDR029W	8,5	7,7	6,3	5,6	0,75	0,673
YDR034C-A	0,1	0,3	4,0	2,8	30,35	0,036
YDR048C	0,9	0,6	0,4	0,9	0,50	0,444
YDR053W	0,2	0,3	0,1	0,3	0,56	0,656
YDR056C	164,5	71,7	83,3	50,3	0,51	0,113
YDR061W	78,0	27,3	55,0	26,7	0,71	0,274
YDR089W	175,7	48,6	35,1	12,0	0,20	0,001
YDR090C	2,3	1,4	2,4	1,7	1,04	0,940
YDR094W	116,3	75,9	61,0	54,7	0,52	0,282
YDR095C	4,4	4,6	0,7	0,8	0,17	0,170
YDR102C	0,4	0,6	0,3	0,6	0,97	0,981
YDR109C	66,0	30,6	67,1	53,0	1,02	0,973
YDR114C	0,7	0,5	0,6	0,7	0,85	0,817
YDR115W	33,6	18,4	9,2	5,6	0,28	0,045
YDR119W-A	5,7	3,5	13,3	4,2	2,33	0,033
YDR124W	2,6	0,9	2,3	1,8	0,88	0,771
YDR131C	16,8	5,0	21,2	3,9	1,27	0,205
YDR132C	13,8	2,6	14,0	4,0	1,02	0,924
YDR149C	0,1	0,3	0,3	0,3	2,32	0,426
YDR154C	5362,3	668,3	7325,1	466,9	1,37	0,003
YDR157W	2,7	2,6	1,1	1,0	0,41	0,297
YDR161W	46,8	12,1	37,9	17,8	0,81	0,442
YDR169C-A	0,0	0,0	0,0	0,0	#DIV/0!	#DIV/0!
YDR179W-A	12,7	4,7	4,4	3,1	0,35	0,026
YDR182W-A	7,0	2,1	6,2	9,2	0,89	0,874
YDR183C-A	2,6	1,5	1,2	1,0	0,45	0,161
YDR186C	106,2	35,5	125,1	34,6	1,18	0,475
YDR187C	0,8	0,9	0,1	0,3	0,17	0,218
YDR193W	0,5	0,6	0,5	0,6	1,10	0,909
YDR199W	2,9	2,1	1,0	0,8	0,36	0,146
YDR210W-B	0,6	0,9	1,2	1,1	1,89	0,451
YDR215C	17,2	10,8	20,7	2,1	1,21	0,543
YDR222W	50,4	31,5	73,6	8,7	1,46	0,204
YDR230W	1,4	1,0	10,2	14,3	7,07	0,268
YDR239C	16,2	5,3	24,4	8,5	1,51	0,150
YDR246W-A	0,4	0,3	0,2	0,4	0,44	0,365
YDR248C	66,8	23,6	114,3	62,7	1,71	0,206
YDR249C	44,4	16,9	54,3	28,2	1,22	0,570
YDR250C	0,5	0,4	0,2	0,4	0,44	0,404
YDR261C-D	766,6	547,9	733,5	481,8	0,96	0,931
YDR261W-A	0,2	0,4	0,0	0,0	0,00	0,356
YDR261W-B	3,8	3,2	3,3	2,9	0,86	0,813
YDR262W	87,2	29,2	123,9	59,6	1,42	0,312
YDR266C	67,3	31,4	52,7	24,9	0,78	0,492
YDR269C	0,0	0,0	0,0	0,0	#DIV/0!	#DIV/0!
YDR271C	0,3	0,4	0,7	0,8	2,12	0,454
YDR274C	0,9	1,1	0,9	1,1	0,98	0,979
YDR278C	0,3	0,5	0,1	0,2	0,29	0,420
YDR282C	8,1	5,9	8,6	3,8	1,06	0,887
YDR286C	15,0	6,3	15,2	7,9	1,02	0,960

YDR290W	0,0	0,0	0,0	0,0	#DIV/0!	#DIV/0!
YDR306C	32,3	6,1	63,1	9,5	1,96	0,002
YDR307W	32,1	17,1	6,8	5,5	0,21	0,030
YDR319C	23,3	6,7	46,6	39,4	2,00	0,289
YDR320W-B	1,8	0,7	1,2	0,8	0,66	0,295
YDR327W	8,8	6,9	7,1	7,1	0,80	0,733
YDR333C	38,4	15,1	58,0	52,3	1,51	0,500
YDR336W	20,5	15,0	17,4	19,3	0,85	0,809
YDR338C	18,4	2,1	16,7	11,8	0,91	0,787
YDR340W	0,0	0,0	0,0	0,0	#DIV/0!	#DIV/0!
YDR341C	527,3	304,5	444,6	265,8	0,84	0,697
YDR344C	0,8	0,7	0,1	0,2	0,13	0,109
YDR348C	31,3	21,0	20,7	18,1	0,66	0,472
YDR352W	54,0	24,7	23,2	20,6	0,43	0,104
YDR354C-A	3,9	4,5	2,8	3,5	0,72	0,719
YDR355C	0,2	0,3	0,1	0,3	0,55	0,589
YDR370C	43,3	21,1	33,9	14,0	0,78	0,488
YDR371C-A	0,0	0,0	0,1	0,2	#DIV/0!	0,356
YDR374W-A	180,9	137,4	112,0	76,9	0,62	0,415
YDR379C-A	27,6	10,3	52,9	21,1	1,92	0,075
YDR387C	20,1	9,0	52,1	44,5	2,60	0,208
YDR391C	43,2	9,9	129,8	87,0	3,00	0,095
YDR401W	0,1	0,2	6,7	13,0	59,87	0,347
YDR415C	47,8	13,3	54,0	8,9	1,13	0,466
YDR426C	2,6	1,7	2,0	2,0	0,80	0,708
YDR442W	0,1	0,2	0,2	0,4	1,62	0,756
YDR444W	30,7	10,5	47,7	25,5	1,55	0,265
YDR445C	4,0	2,7	2,9	2,0	0,72	0,530
YDR455C	1,7	2,1	0,9	1,0	0,51	0,514
YDR461C-A	222,1	88,3	444,5	191,2	2,00	0,079
YDR467C	3,7	4,2	3,9	3,9	1,05	0,954
YDR476C	99,0	40,3	80,0	29,4	0,81	0,474
YDR491C	0,2	0,2	0,0	0,0	0,00	0,144
YDR506C	12,1	9,3	30,5	22,3	2,53	0,178
YDR509W	8,8	10,0	0,8	1,0	0,09	0,162
YDR510C-A	97,4	114,1	43,3	41,4	0,44	0,407
YDR514C	19,3	8,1	16,1	11,3	0,83	0,663
YDR535C	0,4	0,3	0,1	0,1	0,14	0,061
YDR537C	0,3	0,3	0,1	0,3	0,40	0,384
YDR541C	41,1	35,8	26,3	24,1	0,64	0,518
YDR543C	1,0	0,4	0,3	0,4	0,27	0,035
YDR544C	2,0	0,8	0,5	0,4	0,26	0,019
YEA4	7,0	4,5	14,8	14,1	2,12	0,332
YEA6	40,0	16,8	50,1	36,4	1,25	0,634
YEF1	4,2	3,9	2,8	3,5	0,68	0,626
YEF3	1605,3	502,9	1971,3	344,0	1,23	0,275
YEH1	22,1	7,2	5,5	4,9	0,25	0,009
YEH2	63,3	29,8	47,2	18,8	0,75	0,396
YEL007W	256,8	81,1	129,3	98,4	0,50	0,092
YEL008C-A	0,2	0,3	0,8	0,8	3,28	0,236

YEL008W	0,8	0,4	5,9	4,0	7,13	0,043
YEL009C-A	6,5	3,5	1,8	1,5	0,28	0,048
YEL010W	0,4	0,6	5,0	8,6	11,20	0,328
YEL018C-A	1,4	1,4	0,4	0,5	0,30	0,254
YEL020C	69,5	48,0	70,1	39,4	1,01	0,984
YEL020C-B	1,4	1,1	0,8	0,9	0,57	0,430
YEL023C	35,8	16,0	11,8	9,0	0,33	0,040
YEL025C	64,8	18,1	59,3	9,0	0,91	0,604
YEL028W	11,9	5,3	22,7	19,0	1,91	0,315
YEL030C-A	0,2	0,2	0,5	0,4	2,21	0,292
YEL034C-A	84,5	79,3	43,4	41,5	0,51	0,393
YEL043W	41,5	21,5	49,6	24,9	1,19	0,642
YEL045C	0,5	0,4	0,5	0,4	1,00	0,995
YEL047C	161,6	49,9	214,9	42,2	1,33	0,154
YEL050W-A	0,6	0,6	1,1	1,2	1,79	0,501
YEL057C	2,4	1,3	7,0	5,2	2,90	0,142
YEL067C	0,8	0,7	0,5	0,4	0,56	0,381
YEL068C	21,9	10,4	23,5	7,1	1,07	0,806
YEL073C	14,0	12,8	16,7	7,3	1,19	0,728
YEL074W	1,9	0,8	0,5	0,4	0,24	0,018
YEL075C	0,0	0,0	0,0	0,1	#DIV/0!	0,356
YEL075W-A	4,3	2,2	2,4	1,7	0,56	0,229
YEL076C	0,3	0,2	0,0	0,0	0,00	0,036
YEL076C-A	0,0	0,0	0,0	0,0	#DIV/0!	#DIV/0!
YEL077C	2,0	2,1	0,2	0,2	0,09	0,142
YEL1	42,4	23,3	69,8	60,3	1,65	0,430
YEN1	8,3	4,0	7,1	5,3	0,85	0,723
YER006C-A	2,6	2,5	2,8	1,7	1,10	0,868
YER010C	33,2	13,0	15,7	12,5	0,47	0,101
YER023C-A	5,4	4,1	2,3	2,3	0,44	0,246
YER034W	14,5	11,0	29,5	22,2	2,03	0,273
YER038W-A	0,5	0,5	0,2	0,3	0,33	0,324
YER039C-A	0,4	0,5	0,9	0,7	2,06	0,328
YER046W-A	0,7	0,6	0,7	1,2	1,02	0,982
YER053C-A	9,8	1,9	297,1	316,0	30,33	0,119
YER064C	15,2	12,1	18,9	5,1	1,25	0,587
YER067C-A	2,3	1,2	10,9	11,0	4,76	0,173
YER071C	217,7	50,8	46,2	23,4	0,21	0,001
YER076C	14,6	7,7	29,1	33,1	2,00	0,425
YER076W-A	1,1	0,6	1,2	1,1	1,06	0,924
YER077C	22,1	6,7	28,3	18,1	1,28	0,541
YER079C-A	8,0	3,1	3,1	2,2	0,39	0,042
YER079W	67,1	41,7	282,9	109,9	4,22	0,010
YER084W	11,0	6,8	2,7	2,2	0,24	0,059
YER084W-A	0,1	0,1	0,5	0,4	5,69	0,072
YER085C	0,9	1,1	7,6	14,5	8,33	0,396
YER087C-A	6,7	5,1	1,6	1,4	0,24	0,104
YER088C-A	24,0	4,9	2,6	1,8	0,11	0,000
YER088W-B	4,5	4,1	3,5	3,7	0,78	0,729
YER091C-A	0,0	0,1	0,1	0,1	1,79	0,714

YER107W-A	0,0	0,0	0,0	0,0	#DIV/0!	#DIV/0!
YER119C-A	1,6	1,7	1,3	1,4	0,81	0,798
YER121W	10,5	4,5	21,9	24,6	2,09	0,395
YER128W	70,0	46,9	80,9	51,3	1,16	0,763
YER130C	36,1	7,8	56,0	10,6	1,55	0,024
YER135C	0,6	0,5	0,6	0,7	1,02	0,979
YER137C	28,9	21,5	23,8	16,4	0,82	0,721
YER137W-A	0,8	0,6	1,2	1,2	1,45	0,600
YER138C	59,3	51,1	93,4	25,6	1,57	0,278
YER138W-A	2,3	2,3	28,7	13,0	12,26	0,007
YER140W	11,9	2,3	26,7	8,3	2,24	0,014
YER145C-A	20,6	19,6	9,9	9,8	0,48	0,364
YER147C-A	50,1	23,2	86,4	41,8	1,73	0,179
YER148W-A	1,7	0,4	0,5	0,6	0,31	0,013
YER152C	59,3	24,1	35,8	22,3	0,60	0,203
YER152W-A	0,3	0,4	0,0	0,0	0,00	0,134
YER156C	138,6	117,8	110,2	101,0	0,80	0,727
YER158C	9,3	5,9	8,0	9,2	0,85	0,812
YER158W-A	1,9	2,2	3,3	3,4	1,72	0,519
YER160C	354,0	201,1	424,6	337,1	1,20	0,731
YER163C	29,1	17,0	67,6	41,2	2,33	0,134
YER165C-A	0,0	0,0	0,1	0,2	#DIV/0!	0,356
YER172C-A	0,3	0,3	1,1	1,5	3,85	0,332
YER175W-A	1,0	0,7	1,6	1,9	1,59	0,584
YER184C	4,9	1,9	5,5	4,9	1,11	0,841
YER186C	46,6	11,8	28,5	9,0	0,61	0,050
YER187W	2,0	2,5	1,6	1,1	0,83	0,816
YER188C-A	0,1	0,2	0,1	0,2	1,33	0,848
YER188W	10,9	4,7	3,0	3,9	0,28	0,041
YER189W	1,1	0,5	0,3	0,3	0,31	0,057
YER190C-A	2,5	0,5	0,9	0,8	0,34	0,015
YET1	279,0	109,3	313,3	142,3	1,12	0,716
YET2	14,7	19,3	28,9	37,4	1,97	0,525
YET3	126,1	43,6	257,0	101,5	2,04	0,056
YFH1	76,6	29,8	63,2	46,4	0,82	0,644
YFH7	12,4	6,1	11,5	9,2	0,93	0,875
YFL012W	0,6	0,1	0,9	0,6	1,52	0,356
YFL012W-A	0,0	0,0	0,0	0,0	#DIV/0!	#DIV/0!
YFL013W-A	4,9	4,8	5,0	4,9	1,02	0,979
YFL021C-A	3,4	1,6	1,8	1,5	0,53	0,188
YFL034W	39,4	18,6	33,7	4,3	0,85	0,571
YFL040W	0,1	0,2	0,1	0,1	0,48	0,653
YFL042C	28,9	11,7	52,5	15,4	1,82	0,050
YFL051C	0,0	0,0	0,2	0,2	#DIV/0!	0,243
YFL052W	0,2	0,2	0,0	0,1	0,24	0,294
YFL054C	11,5	6,2	36,3	32,1	3,16	0,180
YFL063W	0,0	0,0	0,0	0,0	#DIV/0!	#DIV/0!
YFL065C	1,2	0,6	1,0	0,7	0,84	0,691
YFL066C	0,0	0,1	0,0	0,0	0,00	0,356
YFR006W	56,7	61,6	63,0	56,2	1,11	0,885

YFR009W-A	7,0	5,4	3,2	3,4	0,45	0,274
YFR012W	0,6	0,6	0,1	0,2	0,15	0,144
YFR016C	110,1	89,5	117,7	82,7	1,07	0,905
YFR017C	20,9	10,5	57,1	25,8	2,73	0,041
YFR018C	78,5	30,0	79,7	36,7	1,02	0,959
YFR020W	22,6	7,8	55,3	24,8	2,45	0,046
YFR032C-B	2,0	1,5	1,1	1,5	0,56	0,433
YFR034W-A	181,7	107,5	95,3	30,8	0,52	0,173
YFR035C	6,0	6,5	2,5	2,9	0,41	0,361
YFR036W-A	39,2	27,2	21,6	16,1	0,55	0,308
YFR039C	31,9	11,1	35,6	28,3	1,12	0,816
YFR045W	19,3	10,1	30,9	16,4	1,60	0,274
YFR052C-A	9,0	5,2	15,2	6,9	1,69	0,201
YFR054C	1,2	0,4	2,0	1,4	1,72	0,275
YFR056C	0,0	0,1	0,0	0,0	0,00	0,356
YGK3	19,5	9,7	7,4	6,8	0,38	0,087
YGL010W	45,5	15,0	64,3	32,5	1,41	0,335
YGL014C-A	5,7	4,0	7,7	7,6	1,36	0,653
YGL015C	1,3	1,3	10,9	8,6	8,49	0,069
YGL034C	0,2	0,2	0,0	0,0	0,00	0,077
YGL036W	50,8	26,3	85,2	18,6	1,67	0,077
YGL041C	4,8	2,7	2,2	2,0	0,46	0,171
YGL041C-B	5,4	2,4	1,5	1,1	0,27	0,025
YGL041W-A	66,4	47,8	88,3	73,7	1,33	0,635
YGL042C	2,8	2,5	4,8	5,5	1,71	0,532
YGL052W	0,8	0,5	1,2	1,3	1,53	0,545
YGL069C	2,3	2,1	1,9	1,4	0,82	0,754
YGL072C	27,4	17,9	24,2	22,7	0,88	0,832
YGL074C	0,2	0,5	0,2	0,3	1,08	0,946
YGL081W	5,1	2,8	15,5	24,0	3,04	0,422
YGL082W	98,5	58,6	143,5	37,1	1,46	0,243
YGL088W	21,9	29,3	10,7	13,3	0,49	0,513
YGL101W	41,7	13,5	12,8	8,8	0,31	0,012
YGL102C	297,3	210,2	160,6	128,3	0,54	0,309
YGL108C	20,1	9,6	37,3	21,2	1,86	0,190
YGL109W	3,2	1,3	3,4	2,9	1,06	0,916
YGL114W	47,2	10,8	72,6	30,4	1,54	0,167
YGL117W	203,7	103,3	135,6	77,6	0,67	0,332
YGL118C	0,7	0,8	0,1	0,2	0,12	0,153
YGL123C-A	188,3	226,0	100,0	112,0	0,53	0,510
YGL138C	0,5	0,3	0,3	0,6	0,73	0,723
YGL140C	31,0	16,7	24,6	6,6	0,79	0,506
YGL149W	0,8	0,3	1,4	1,2	1,64	0,409
YGL152C	2,5	2,2	2,0	1,8	0,80	0,731
YGL159W	27,6	10,6	51,4	46,9	1,86	0,362
YGL165C	0,4	0,4	0,8	1,1	2,23	0,468
YGL176C	13,3	7,3	13,1	1,2	0,98	0,959
YGL177W	0,6	0,9	1,1	0,9	1,83	0,451
YGL185C	23,4	10,1	58,5	26,1	2,51	0,046
YGL188C	35,8	8,1	21,2	6,9	0,59	0,034

YGL188C-A	16,2	4,3	4,2	3,3	0,26	0,004
YGL199C	1,5	1,0	1,0	0,9	0,66	0,464
YGL204C	35,4	9,1	23,5	3,9	0,67	0,054
YGL214W	8,4	3,8	3,1	2,2	0,36	0,049
YGL217C	2,7	1,9	1,2	1,4	0,43	0,247
YGL218W	0,3	0,2	0,4	0,6	1,09	0,930
YGL230C	6,8	2,9	3,3	3,0	0,49	0,144
YGL235W	0,0	0,1	4,8	3,4	119,53	0,030
YGL239C	0,0	0,0	0,0	0,0	#DIV/0!	#DIV/0!
YGL242C	85,6	14,6	87,7	29,4	1,03	0,898
YGL262W	0,3	0,3	0,1	0,3	0,46	0,448
YGP1	37,9	12,9	560,6	322,5	14,81	0,018
YGR001C	207,0	270,7	187,6	261,2	0,91	0,921
YGR012W	56,2	13,0	58,5	27,5	1,04	0,884
YGR015C	6,4	3,0	5,5	3,8	0,86	0,734
YGR016W	1,9	1,1	3,5	2,8	1,87	0,316
YGR017W	134,2	81,7	110,5	53,5	0,82	0,645
YGR018C	3,5	2,6	2,4	2,3	0,68	0,540
YGR021W	4,9	3,1	11,8	10,4	2,42	0,249
YGR022C	0,2	0,3	0,3	0,2	1,33	0,727
YGR026W	289,6	140,4	257,2	108,9	0,89	0,727
YGR031W	4,4	3,8	1,6	1,3	0,36	0,207
YGR035C	18,3	5,9	10,7	10,5	0,58	0,252
YGR035W-A	6,1	2,1	1,7	1,2	0,28	0,010
YGR042W	40,4	14,0	36,8	3,5	0,91	0,639
YGR045C	2,3	0,9	3,6	3,0	1,59	0,428
YGR050C	2,6	2,4	1,2	1,3	0,47	0,353
YGR051C	0,0	0,0	0,2	0,4	#DIV/0!	0,356
YGR053C	13,3	11,8	25,3	34,3	1,90	0,535
YGR054W	118,9	62,7	146,1	15,5	1,23	0,432
YGR064W	11,9	7,1	6,2	5,9	0,52	0,255
YGR066C	9,6	4,8	36,3	26,2	3,76	0,093
YGR067C	4,8	2,0	7,1	4,9	1,48	0,422
YGR069W	1,9	0,8	1,3	1,0	0,67	0,351
YGR071C	15,5	9,0	17,2	4,4	1,11	0,756
YGR073C	21,8	6,0	13,9	9,8	0,64	0,221
YGR079W	73,5	29,6	22,7	1,3	0,31	0,014
YGR093W	15,5	4,4	4,7	4,2	0,30	0,012
YGR102C	147,0	24,8	122,4	23,5	0,83	0,199
YGR107W	1,5	0,4	4,8	8,0	3,26	0,432
YGR109W-A	2,9	1,9	26,9	17,2	9,13	0,032
YGR109W-B	144,5	70,6	567,3	430,7	3,93	0,101
YGR111W	37,3	9,9	50,4	6,8	1,35	0,070
YGR114C	1,6	1,4	0,4	0,5	0,24	0,147
YGR115C	7,0	5,8	8,8	6,0	1,25	0,692
YGR117C	62,3	12,9	70,6	2,5	1,13	0,255
YGR121W-A	0,1	0,2	0,0	0,0	0,00	0,134
YGR122W	21,8	13,4	33,1	16,6	1,52	0,330
YGR125W	96,2	22,4	78,0	26,3	0,81	0,332
YGR126W	0,0	0,1	10,6	16,1	215,96	0,238

YGR127W	23,8	11,9	75,2	7,9	3,17	0,000
YGR130C	83,3	49,9	136,3	85,6	1,64	0,326
YGR137W	361,7	202,6	860,9	325,1	2,38	0,040
YGR139W	0,1	0,2	0,1	0,1	0,89	0,939
YGR149W	72,0	36,0	56,9	18,9	0,79	0,486
YGR151C	239,0	140,4	37,8	26,0	0,16	0,030
YGR153W	41,2	15,9	14,3	10,4	0,35	0,030
YGR161W-C	7,6	3,4	72,4	25,5	9,47	0,002
YGR164W	1,3	2,6	0,2	0,4	0,15	0,430
YGR168C	33,4	11,4	36,0	11,9	1,08	0,759
YGR169C-A	238,0	84,2	330,1	44,8	1,39	0,102
YGR176W	0,0	0,1	0,0	0,1	0,58	0,731
YGR201C	21,6	25,1	19,4	18,6	0,90	0,892
YGR205W	16,9	8,3	26,4	25,1	1,57	0,498
YGR207C	340,8	265,4	197,5	152,4	0,58	0,385
YGR210C	111,5	71,6	98,0	78,8	0,88	0,809
YGR219W	16,2	8,1	4,4	3,7	0,27	0,038
YGR226C	0,2	0,3	0,0	0,0	0,00	0,356
YGR228W	35,4	27,5	22,2	16,9	0,63	0,447
YGR235C	90,7	31,2	107,6	43,9	1,19	0,554
YGR237C	10,7	3,4	21,0	15,3	1,96	0,239
YGR240C-A	3,3	1,0	3,7	2,8	1,11	0,806
YGR250C	63,6	48,0	139,2	43,6	2,19	0,059
YGR251W	15,5	14,6	42,1	24,9	2,71	0,115
YGR259C	1,1	1,2	1,3	1,2	1,11	0,890
YGR265W	1,1	0,8	0,6	0,6	0,54	0,378
YGR266W	74,9	41,3	50,8	20,3	0,68	0,335
YGR270C-A	1,0	1,1	0,4	0,4	0,43	0,373
YGR273C	0,0	0,0	0,3	0,4	#DIV/0!	0,146
YGR283C	69,0	25,9	39,6	20,2	0,57	0,124
YGR290W	0,1	0,2	0,0	0,0	0,00	0,356
YHB1	134,2	40,3	157,6	109,2	1,17	0,701
YHC1	35,8	20,2	31,2	16,6	0,87	0,738
YHC3	31,8	11,8	25,6	21,7	0,80	0,630
YHI9	155,1	64,7	126,4	6,3	0,81	0,410
YHK8	13,7	7,1	9,9	7,7	0,72	0,487
YHL002C-A	5,2	1,6	6,2	5,3	1,19	0,734
YHL006W-A	6,5	2,5	10,7	10,3	1,65	0,457
YHL008C	44,7	13,0	59,4	6,2	1,33	0,087
YHL009W-A	0,5	0,6	13,5	16,2	27,45	0,161
YHL009W-B	49,6	40,4	65,7	45,8	1,32	0,619
YHL012W	14,5	10,9	10,5	10,8	0,72	0,621
YHL017W	33,5	17,6	44,8	33,1	1,33	0,572
YHL018W	1,0	1,0	0,3	0,2	0,30	0,246
YHL019W-A	1,0	0,6	0,2	0,2	0,19	0,048
YHL026C	33,9	3,9	80,3	69,0	2,37	0,228
YHL030W-A	0,7	0,2	0,3	0,5	0,51	0,265
YHL037C	0,7	0,6	0,5	0,4	0,70	0,614
YHL042W	4,2	2,5	35,4	28,2	8,37	0,070
YHL044W	16,1	10,0	13,8	4,5	0,86	0,696

YHL045W	0,6	0,2	0,7	0,5	1,20	0,677
YHL046W-A	1,7	1,3	0,2	0,2	0,09	0,059
YHL048C-A	24,1	8,4	13,8	11,6	0,57	0,201
YHL049C	0,5	0,2	0,3	0,2	0,54	0,174
YHL050C	18,7	14,3	3,6	2,9	0,19	0,085
YHM2	459,8	288,8	217,0	87,4	0,47	0,159
YHP1	57,6	44,1	12,3	9,9	0,21	0,091
YHR003C	44,4	9,1	46,3	18,8	1,04	0,864
YHR009C	128,5	56,0	160,9	87,5	1,25	0,555
YHR020W	340,4	287,8	290,0	166,7	0,85	0,772
YHR022C	2,0	2,1	12,7	5,8	6,45	0,013
YHR022C-A	0,3	0,5	0,2	0,4	0,71	0,818
YHR028W-A	2,6	1,9	3,3	3,9	1,26	0,766
YHR032C-A	1,1	1,3	1,6	1,6	1,53	0,608
YHR032W-A	61,0	22,9	58,3	13,7	0,96	0,849
YHR033W	48,7	23,9	66,2	28,0	1,36	0,381
YHR035W	13,7	3,5	46,3	40,3	3,38	0,158
YHR045W	73,2	66,2	57,5	47,4	0,78	0,712
YHR050W-A	1,5	1,0	2,0	1,3	1,29	0,613
YHR052W-A	99,7	72,8	134,5	119,3	1,35	0,637
YHR054C	73,7	30,9	90,9	15,2	1,23	0,356
YHR056W-A	0,7	1,1	0,7	0,5	0,96	0,961
YHR063W-A	46,9	41,1	68,9	65,0	1,47	0,588
YHR069C-A	0,6	0,8	0,4	0,3	0,61	0,564
YHR070C-A	1,5	2,0	2,1	2,2	1,34	0,739
YHR071C-A	1,0	2,0	2,7	1,6	2,57	0,240
YHR078W	86,1	46,0	69,2	7,9	0,80	0,496
YHR080C	37,5	28,2	32,1	22,0	0,85	0,771
YHR086W-A	0,8	1,0	0,1	0,3	0,17	0,252
YHR095W	0,5	0,8	0,3	0,4	0,60	0,635
YHR097C	53,8	25,0	133,4	76,6	2,48	0,095
YHR112C	60,6	31,0	195,0	84,7	3,22	0,025
YHR113W	151,8	163,0	198,9	193,0	1,31	0,722
YHR122W	259,5	104,2	721,5	315,3	2,78	0,032
YHR125W	0,0	0,0	0,1	0,2	#DIV/0!	0,356
YHR127W	30,7	13,9	8,7	7,6	0,28	0,032
YHR130C	20,4	12,8	18,1	4,8	0,88	0,738
YHR131C	2,9	1,4	5,9	5,4	2,08	0,311
YHR131W-A	0,2	0,3	0,0	0,0	0,00	0,356
YHR138C	80,5	73,5	158,2	124,6	1,96	0,324
YHR139C-A	0,3	0,3	0,2	0,3	0,83	0,843
YHR140W	11,4	3,4	2,6	2,0	0,23	0,005
YHR145C	55,8	36,2	53,5	54,3	0,96	0,948
YHR159W	9,0	5,1	10,9	9,6	1,20	0,748
YHR165W-A	0,5	0,3	1,0	0,8	2,10	0,273
YHR173C	0,9	1,3	0,7	0,5	0,75	0,750
YHR175W-A	1,3	0,9	0,9	1,2	0,72	0,641
YHR177W	0,3	0,3	0,1	0,3	0,48	0,516
YHR180W	7,6	3,3	8,7	10,3	1,15	0,844
YHR180W-A	3,6	2,0	2,4	2,5	0,66	0,474

YHR182C-A	3,0	3,3	1,4	1,6	0,46	0,418
YHR182W	52,6	17,4	28,9	17,2	0,55	0,101
YHR192W	197,6	141,9	125,3	108,5	0,63	0,449
YHR193C-A	0,7	0,3	1,4	1,0	2,15	0,207
YHR202W	15,4	6,6	18,0	16,0	1,17	0,771
YHR210C	6,0	2,0	5,4	4,1	0,91	0,814
YHR217C	0,3	0,2	0,2	0,2	0,50	0,347
YHR219C-A	15,9	15,7	4,5	4,6	0,28	0,212
YIA6	13,0	6,9	19,8	17,6	1,52	0,498
YIF1	76,5	34,1	38,5	30,4	0,50	0,147
YIG1	1,5	0,5	0,1	0,3	0,09	0,003
YIH1	176,8	100,4	166,6	71,9	0,94	0,875
YIL001W	14,3	2,9	21,8	12,8	1,52	0,295
YIL002W-A	19,4	9,4	9,3	7,1	0,48	0,136
YIL012W	1,6	1,8	0,3	0,4	0,21	0,218
YIL014C-A	19,0	14,2	24,6	23,7	1,29	0,703
YIL020C-A	131,9	40,1	49,5	20,4	0,37	0,010
YIL021C-A	2,8	3,1	1,1	1,2	0,41	0,360
YIL024C	24,9	9,3	10,8	6,9	0,43	0,051
YIL028W	0,2	0,3	0,0	0,1	0,20	0,319
YIL029C	46,5	16,3	53,1	19,0	1,14	0,619
YIL029W-A	0,0	0,1	0,4	0,5	12,87	0,176
YIL032C	2,3	4,2	0,0	0,1	0,01	0,318
YIL046W-A	0,0	0,0	0,0	0,0	#DIV/0!	#DIV/0!
YIL055C	17,5	12,1	36,3	12,9	2,07	0,078
YIL058W	0,2	0,3	0,2	0,2	0,77	0,828
YIL060W	0,8	0,6	0,9	1,0	1,15	0,848
YIL066W-A	3,5	1,1	2,0	1,7	0,57	0,181
YIL067C	17,2	12,0	21,4	12,4	1,24	0,644
YIL068W-A	5,9	4,4	3,0	2,3	0,51	0,288
YIL071W-A	1,2	0,8	1,1	0,8	0,93	0,880
YIL077C	16,3	10,1	21,4	16,2	1,32	0,607
YIL080W	0,3	0,7	4,0	3,0	11,93	0,055
YIL082W-A	1,2	0,5	9,8	2,7	8,10	0,001
YIL086C	0,0	0,0	0,0	0,0	#DIV/0!	#DIV/0!
YIL092W	20,3	10,0	30,2	9,7	1,49	0,205
YIL096C	94,3	47,0	72,3	35,5	0,77	0,483
YIL100C-A	0,2	0,2	0,2	0,2	0,99	0,989
YIL100W	0,0	0,0	0,0	0,1	#DIV/0!	0,356
YIL102C	0,2	0,4	0,3	0,5	1,53	0,706
YIL102C-A	4,7	1,5	13,8	4,5	2,90	0,009
YIL105W-A	4,3	2,9	3,9	3,9	0,91	0,873
YIL108W	26,9	12,1	45,3	22,3	1,69	0,196
YIL115W-A	8,1	5,8	2,6	2,3	0,32	0,123
YIL141W	0,6	0,5	0,0	0,0	0,00	0,039
YIL142C-A	1,4	1,1	0,9	1,0	0,67	0,575
YIL151C	28,4	11,8	24,4	17,7	0,86	0,718
YIL152W	74,2	48,7	82,2	59,9	1,11	0,842
YIL156W-A	0,8	1,0	0,5	0,5	0,62	0,614
YIL156W-B	160,9	166,1	136,2	140,7	0,85	0,828

YIL161W	24,2	8,5	35,7	18,7	1,47	0,308
YIL163C	0,1	0,2	0,1	0,3	1,09	0,955
YIL165C	29,5	12,9	48,9	24,9	1,66	0,216
YIL166C	16,1	6,0	10,3	7,9	0,64	0,284
YIL168W	0,6	0,4	2,2	1,7	3,78	0,126
YIL169C	0,9	0,3	5,4	5,7	5,77	0,165
YIL171W-A	0,6	0,2	0,0	0,0	0,00	0,001
YIL174W	0,3	0,4	0,6	0,7	1,66	0,581
YIM1	20,5	5,8	22,0	10,2	1,07	0,806
YIM2	2,1	0,8	5,6	4,9	2,62	0,211
YIP1	110,9	124,1	104,1	121,8	0,94	0,940
YIP3	92,7	48,5	55,3	19,4	0,60	0,201
YIP4	32,1	28,1	16,3	5,1	0,51	0,313
YIP5	190,4	82,0	125,4	45,2	0,66	0,214
YIR007W	17,3	1,8	15,7	6,5	0,91	0,665
YIR014W	5,7	3,3	10,6	7,5	1,86	0,274
YIR016W	30,3	13,6	45,8	11,2	1,51	0,129
YIR017W-A	2,8	2,6	17,4	15,4	6,31	0,110
YIR018C-A	2,6	0,4	2,5	1,9	0,97	0,930
YIR020C	2,9	1,6	6,4	4,3	2,19	0,181
YIR020W-A	0,1	0,2	0,1	0,2	1,17	0,917
YIR021W-A	65,2	78,3	60,9	67,6	0,93	0,936
YIR024C	52,3	26,5	117,3	70,6	2,24	0,135
YIR030W-A	0,9	0,4	0,2	0,2	0,20	0,015
YIR035C	57,3	29,8	287,6	182,1	5,02	0,047
YIR036W-A	10,7	8,4	13,0	8,3	1,22	0,707
YIR042C	1,9	1,4	11,0	16,5	5,65	0,315
YIR043C	0,9	0,8	0,1	0,2	0,09	0,085
YJL007C	0,0	0,0	0,0	0,0	#DIV/0!	#DIV/0!
YJL009W	5,4	4,7	6,9	7,6	1,27	0,756
YJL015C	1,8	1,2	0,8	0,7	0,45	0,206
YJL016W	57,1	24,3	57,1	24,0	1,00	0,998
YJL020W-A	0,3	0,3	0,6	0,4	2,41	0,235
YJL022W	0,0	0,0	0,0	0,0	#DIV/0!	#DIV/0!
YJL026C-A	6,4	5,6	3,9	4,1	0,61	0,500
YJL027C	0,2	0,5	0,3	0,5	1,12	0,937
YJL028W	0,1	0,2	0,0	0,0	0,00	0,356
YJL032W	3,9	3,8	4,1	4,5	1,04	0,963
YJL043W	2,8	1,4	2,1	1,4	0,72	0,463
YJL045W	2,7	1,7	5,7	3,2	2,11	0,152
YJL047C-A	0,3	0,3	0,0	0,0	0,00	0,135
YJL049W	124,6	27,5	76,1	29,7	0,61	0,053
YJL052C-A	0,0	0,1	0,0	0,1	0,87	0,928
YJL055W	253,6	97,0	394,9	114,1	1,56	0,108
YJL064W	0,7	0,7	0,3	0,5	0,40	0,376
YJL067W	0,3	0,3	0,5	0,7	2,01	0,503
YJL068C	114,1	77,6	131,8	78,1	1,16	0,758
YJL070C	14,9	9,1	21,9	20,2	1,46	0,555
YJL077W-A	35,8	8,4	23,6	17,4	0,66	0,255
YJL077W-B	16,2	6,0	34,5	15,0	2,12	0,064

YJL086C	0,2	0,3	0,0	0,0	0,00	0,256
YJL107C	0,3	0,3	0,2	0,2	0,58	0,496
YJL113W	0,0	0,0	0,4	0,3	#DIV/0!	0,026
YJL114W	0,0	0,0	0,1	0,3	#DIV/0!	0,356
YJL118W	48,7	25,8	9,5	8,7	0,19	0,028
YJL119C	5,8	3,8	4,3	3,1	0,73	0,552
YJL120W	0,9	0,9	0,4	0,6	0,42	0,360
YJL127C-B	110,3	56,7	388,9	119,0	3,53	0,006
YJL132W	15,4	5,1	25,3	14,6	1,65	0,246
YJL133C-A	1258,2	318,6	1034,2	529,3	0,82	0,496
YJL135W	0,1	0,2	0,0	0,0	0,00	0,356
YJL144W	25,4	16,6	102,7	69,0	4,03	0,072
YJL150W	0,0	0,0	0,2	0,2	#DIV/0!	0,204
YJL152W	5,5	4,0	5,1	4,8	0,93	0,906
YJL156W-A	0,5	0,5	0,7	0,8	1,57	0,584
YJL160C	5,4	2,6	5,8	3,9	1,08	0,857
YJL163C	10,8	0,9	9,0	6,8	0,83	0,617
YJL169W	7,9	6,9	6,7	5,6	0,86	0,810
YJL171C	49,6	29,7	51,6	21,9	1,04	0,914
YJL175W	0,0	0,0	0,0	0,0	#DIV/0!	#DIV/0!
YJL181W	41,9	18,9	12,1	8,7	0,29	0,029
YJL182C	5,1	4,8	1,7	2,4	0,34	0,255
YJL185C	12,0	5,7	9,2	6,4	0,77	0,538
YJL193W	30,7	15,3	27,9	23,4	0,91	0,847
YJL195C	0,0	0,1	0,0	0,0	0,00	0,356
YJL202C	4,5	1,9	2,5	1,9	0,56	0,180
YJL206C	5,0	1,7	15,1	11,4	3,04	0,129
YJL211C	0,7	0,5	0,4	0,6	0,56	0,464
YJL213W	18,0	8,3	101,8	55,7	5,66	0,025
YJL215C	0,0	0,1	0,1	0,2	1,75	0,723
YJL218W	11,0	3,7	18,2	12,7	1,65	0,320
YJR003C	27,7	12,8	45,2	7,6	1,63	0,057
YJR008W	22,2	2,7	61,6	21,9	2,78	0,012
YJR011C	16,7	9,6	1,8	1,4	0,11	0,021
YJR012C	55,6	30,6	56,4	21,8	1,01	0,968
YJR015W	127,5	35,1	89,4	36,6	0,70	0,183
YJR018W	0,3	0,2	0,0	0,0	0,00	0,035
YJR020W	0,8	0,4	0,3	0,4	0,36	0,100
YJR023C	0,1	0,2	0,0	0,0	0,00	0,356
YJR030C	27,0	8,3	4,6	4,0	0,17	0,003
YJR037W	0,0	0,0	0,1	0,1	#DIV/0!	0,356
YJR038C	0,1	0,2	0,0	0,1	0,31	0,532
YJR039W	13,1	4,2	17,4	1,2	1,33	0,094
YJR054W	35,7	11,4	27,5	2,0	0,77	0,207
YJR056C	47,0	22,9	40,0	20,0	0,85	0,659
YJR061W	11,1	4,9	4,1	2,8	0,37	0,048
YJR071W	1,8	1,7	1,1	1,0	0,60	0,475
YJR079W	2,1	2,2	1,8	1,8	0,89	0,877
YJR085C	155,1	82,6	177,0	96,9	1,14	0,742
YJR087W	71,8	32,0	94,1	46,8	1,31	0,460

YJR096W	25,1	10,3	69,8	41,5	2,78	0,081
YJR098C	23,9	8,7	9,2	7,7	0,38	0,043
YJR107W	34,1	18,8	16,2	11,0	0,48	0,153
YJR111C	40,3	17,1	70,1	34,0	1,74	0,169
YJR112W-A	102,3	78,0	45,6	35,6	0,45	0,233
YJR114W	3,9	2,4	1,9	1,3	0,48	0,191
YJR115W	15,8	9,3	20,1	10,7	1,28	0,562
YJR116W	38,8	10,5	26,3	8,6	0,68	0,112
YJR124C	60,3	34,2	42,1	20,1	0,70	0,394
YJR129C	19,4	3,6	31,5	3,6	1,63	0,003
YJR141W	28,2	29,5	6,8	5,6	0,24	0,203
YJR142W	38,0	7,2	48,1	14,7	1,27	0,264
YJR146W	1,3	1,1	1,4	1,3	1,10	0,884
YJR149W	4,9	1,7	3,9	2,6	0,80	0,556
YJR154W	0,8	0,8	0,9	1,0	1,19	0,822
YJR162C	0,9	0,8	0,1	0,2	0,14	0,108
YJU2	0,5	0,4	1,3	0,9	2,68	0,149
YJU3	80,5	24,0	91,2	67,5	1,13	0,776
YKE2	66,6	37,6	25,2	9,4	0,38	0,076
YKE4	37,2	34,2	33,1	18,6	0,89	0,841
YKL018C-A	107,2	50,8	138,8	87,3	1,30	0,553
YKL023W	34,6	7,1	80,0	17,8	2,31	0,003
YKL027W	60,7	4,6	42,5	18,0	0,70	0,098
YKL030W	5,1	5,0	4,2	3,8	0,83	0,797
YKL033W-A	302,1	292,2	183,6	163,9	0,61	0,506
YKL036C	11,7	9,0	11,1	6,3	0,95	0,911
YKL044W	3,4	4,4	1,5	1,9	0,43	0,448
YKL047W	40,5	15,4	41,7	28,9	1,03	0,945
YKL050C	6,6	4,2	8,1	3,2	1,23	0,589
YKL053W	1,8	1,1	0,9	0,9	0,47	0,222
YKL063C	295,3	75,7	153,2	63,0	0,52	0,028
YKL066W	1,1	0,4	0,4	0,4	0,32	0,044
YKL069W	122,2	44,0	174,0	52,8	1,42	0,183
YKL070W	2,5	1,1	2,2	1,5	0,88	0,759
YKL071W	1,4	0,7	7,7	9,0	5,34	0,213
YKL075C	8,6	4,6	13,8	5,4	1,60	0,190
YKL077W	109,0	53,4	105,8	41,2	0,97	0,928
YKL091C	68,1	20,9	30,4	16,1	0,45	0,029
YKL100C	110,3	36,8	166,1	58,3	1,51	0,156
YKL102C	0,6	0,4	0,2	0,3	0,38	0,184
YKL105C	33,7	19,8	36,9	22,6	1,10	0,838
YKL106C-A	0,1	0,2	0,2	0,4	2,31	0,568
YKL107W	1,1	1,1	0,8	0,7	0,74	0,676
YKL111C	0,9	0,8	0,0	0,0	0,00	0,072
YKL118W	0,0	0,0	0,0	0,0	#DIV/0!	#DIV/0!
YKL123W	48,0	18,7	32,7	15,6	0,68	0,257
YKL133C	12,3	3,3	19,8	3,5	1,61	0,021
YKL136W	0,9	0,7	0,7	0,9	0,80	0,764
YKL147C	0,4	0,3	0,8	0,5	1,93	0,268
YKL151C	60,0	30,9	489,5	420,3	8,16	0,088

YKL153W	95,8	97,1	110,7	107,7	1,16	0,843
YKL162C	5,7	1,0	8,6	5,6	1,51	0,347
YKL169C	5,1	4,3	3,4	3,7	0,67	0,577
YKL177W	1,2	1,2	7,3	12,6	6,32	0,370
YKL183C-A	3,0	2,0	1,0	0,9	0,33	0,123
YKL187C	2,7	1,2	1,9	1,3	0,68	0,367
YKL202W	2,8	3,2	4,6	4,8	1,63	0,560
YKL222C	7,8	5,0	8,8	7,8	1,14	0,825
YKR005C	8,1	4,6	9,0	4,0	1,12	0,770
YKR011C	44,4	14,4	114,9	37,2	2,59	0,012
YKR012C	4,7	1,3	1,4	1,1	0,30	0,007
YKR015C	1,1	0,6	0,5	0,4	0,45	0,124
YKR017C	17,2	5,3	11,7	2,5	0,68	0,111
YKR018C	54,8	8,3	89,7	16,8	1,64	0,010
YKR023W	39,6	12,6	45,5	30,9	1,15	0,735
YKR033C	0,1	0,1	0,0	0,0	0,00	0,137
YKR040C	14,1	15,9	3,0	2,2	0,21	0,219
YKR041W	5,1	4,0	3,9	3,0	0,76	0,645
YKR043C	285,7	118,5	183,2	97,0	0,64	0,229
YKR045C	17,2	12,0	35,8	16,4	2,08	0,117
YKR047W	62,6	30,7	33,9	30,1	0,54	0,230
YKR051W	16,4	15,0	9,3	9,6	0,57	0,454
YKR070W	49,0	18,4	53,8	40,7	1,10	0,837
YKR073C	1,0	0,9	0,6	0,6	0,57	0,455
YKR075C	13,9	4,1	28,4	5,4	2,05	0,005
YKR075W-A	0,1	0,2	0,1	0,2	1,08	0,958
YKR078W	16,3	8,0	16,7	4,1	1,03	0,930
YKR096W	17,0	7,6	24,4	13,8	1,43	0,389
YKR104W	11,5	5,4	1,5	1,8	0,13	0,013
YKR106W	5,5	3,5	5,7	4,1	1,05	0,924
YKT6	183,2	55,5	174,8	72,9	0,95	0,861
YKU70	15,3	3,3	42,0	34,4	2,75	0,173
YKU80	10,9	4,7	15,1	10,9	1,38	0,506
YLF2	29,8	5,8	23,3	8,4	0,78	0,248
YLH47	45,9	32,4	62,2	63,5	1,36	0,662
YLL006W-A	0,0	0,1	0,1	0,1	1,75	0,723
YLL007C	8,9	5,1	14,8	14,0	1,66	0,460
YLL017W	0,3	0,3	0,3	0,3	0,77	0,716
YLL020C	33,6	14,1	77,4	51,6	2,31	0,152
YLL032C	11,1	4,2	13,7	5,9	1,23	0,505
YLL047W	0,1	0,2	4,9	6,6	46,41	0,194
YLL053C	6,4	4,7	5,9	4,3	0,92	0,881
YLL054C	14,2	6,9	17,1	11,8	1,20	0,685
YLL056C	1,7	0,8	1,9	1,4	1,10	0,843
YLL058W	22,8	11,7	25,0	10,4	1,10	0,791
YLL059C	0,2	0,2	4,0	4,5	24,21	0,143
YLL066C	662,7	135,3	316,7	23,9	0,48	0,002
YLL066W-B	27,9	17,7	12,6	12,4	0,45	0,208
YLR001C	34,9	18,6	42,1	19,5	1,21	0,612
YLR012C	0,8	0,8	1,0	1,0	1,22	0,781

YLR030W	1,7	1,1	0,7	1,1	0,39	0,208
YLR031W	3,6	1,2	19,3	24,2	5,32	0,243
YLR040C	26,9	16,1	34,7	31,5	1,29	0,674
YLR041W	3,4	1,8	3,7	2,7	1,09	0,850
YLR042C	28,0	11,8	21,3	19,3	0,76	0,572
YLR046C	19,5	14,4	5,0	4,3	0,26	0,102
YLR049C	119,1	58,8	18,9	5,9	0,16	0,015
YLR050C	64,9	31,8	26,3	19,6	0,40	0,084
YLR053C	12,5	4,6	41,7	29,0	3,33	0,094
YLR057W	28,4	16,6	50,1	40,2	1,77	0,357
YLR063W	3,2	1,6	3,4	2,4	1,08	0,866
YLR065C	1437,7	712,9	508,7	251,3	0,35	0,049
YLR072W	237,7	47,5	171,7	40,4	0,72	0,079
YLR076C	76,7	69,2	38,0	40,9	0,50	0,373
YLR099W-A	0,3	0,3	0,4	0,5	1,29	0,750
YLR101C	1,1	0,7	5,5	10,3	5,10	0,428
YLR108C	42,1	18,9	75,1	29,8	1,78	0,111
YLR111W	0,0	0,0	0,1	0,1	#DIV/0!	0,356
YLR112W	49,5	27,8	28,6	23,1	0,58	0,291
YLR118C	54,1	31,2	41,4	22,1	0,77	0,532
YLR122C	5,7	2,2	4,2	3,2	0,74	0,475
YLR123C	0,0	0,0	0,0	0,0	#DIV/0!	#DIV/0!
YLR125W	4,9	3,0	4,4	4,2	0,89	0,840
YLR126C	25,8	11,5	15,3	12,7	0,59	0,265
YLR132C	17,0	10,6	10,7	9,9	0,63	0,415
YLR137W	23,1	7,4	4,5	3,5	0,20	0,004
YLR140W	1,3	0,8	0,3	0,5	0,23	0,068
YLR143W	17,8	9,2	25,7	7,8	1,45	0,237
YLR146W-A	48,7	31,7	91,6	78,9	1,88	0,352
YLR149C	15,3	6,4	50,6	17,1	3,31	0,008
YLR152C	65,0	36,7	83,8	31,8	1,29	0,469
YLR154C-G	38,1	41,4	41,5	13,6	1,09	0,881
YLR154C-H	8,7	4,8	6,0	8,0	0,69	0,579
YLR154W-A	2,5	1,6	3,5	3,3	1,39	0,607
YLR162W	9,5	5,2	18,5	20,0	1,95	0,416
YLR169W	12,1	8,0	21,2	10,2	1,75	0,213
YLR171W	7,9	3,7	3,1	3,0	0,40	0,093
YLR173W	1,1	1,4	13,1	14,3	11,53	0,147
YLR177W	10,2	3,3	57,0	32,1	5,59	0,027
YLR179C	691,9	566,5	482,3	369,7	0,70	0,558
YLR198C	73,9	47,0	101,5	73,6	1,37	0,551
YLR202C	1,7	0,5	0,5	0,7	0,29	0,033
YLR211C	1,8	0,8	0,9	0,7	0,48	0,142
YLR217W	7,8	8,2	20,7	22,6	2,67	0,321
YLR222C-A	61,1	17,2	50,4	38,9	0,83	0,634
YLR224W	35,1	12,0	33,9	13,2	0,97	0,900
YLR225C	35,6	4,3	75,4	24,3	2,12	0,018
YLR227W-B	0,7	0,2	2,4	1,9	3,64	0,124
YLR232W	0,5	0,8	0,2	0,4	0,53	0,653
YLR235C	0,0	0,1	0,0	0,1	1,31	0,856

YLR236C	0,1	0,2	0,0	0,0	0,00	0,223
YLR241W	19,0	15,9	20,3	13,3	1,06	0,910
YLR243W	33,1	14,5	41,5	28,6	1,25	0,620
YLR252W	8,1	1,6	5,0	4,7	0,62	0,257
YLR253W	26,9	5,8	40,5	23,8	1,50	0,310
YLR255C	0,0	0,1	0,0	0,1	1,07	0,963
YLR257W	288,0	144,0	278,1	52,5	0,97	0,902
YLR264C-A	311,9	80,7	154,7	37,9	0,50	0,012
YLR269C	1,2	1,1	0,8	0,7	0,67	0,564
YLR271W	18,7	3,9	64,5	9,6	3,44	0,000
YLR278C	23,5	11,5	28,7	22,5	1,22	0,694
YLR279W	0,7	0,9	0,0	0,0	0,00	0,168
YLR280C	10,2	3,8	3,3	2,9	0,32	0,029
YLR281C	0,2	0,3	0,0	0,1	0,17	0,447
YLR282C	0,5	0,5	0,0	0,1	0,06	0,122
YLR283W	33,1	9,0	46,2	45,7	1,40	0,591
YLR285C-A	11,2	5,7	12,3	12,3	1,10	0,877
YLR286W-A	8,1	6,7	8,8	9,7	1,08	0,916
YLR287C	142,8	26,8	129,8	47,9	0,91	0,651
YLR290C	61,3	32,5	74,1	38,8	1,21	0,629
YLR294C	0,5	1,0	0,1	0,2	0,17	0,447
YLR296W	0,7	0,1	0,6	0,5	0,92	0,838
YLR297W	10,5	3,3	13,5	5,2	1,29	0,364
YLR299C-A	1,1	1,3	0,3	0,5	0,30	0,319
YLR302C	0,3	0,4	0,1	0,2	0,40	0,425
YLR307C-A	0,6	0,7	1,2	0,8	2,00	0,322
YLR312C	4,4	3,0	1,5	1,3	0,34	0,131
YLR317W	19,9	5,8	11,2	7,8	0,56	0,124
YLR326W	28,4	15,1	44,6	16,2	1,57	0,192
YLR339C	10,6	9,4	14,9	9,1	1,41	0,531
YLR342W-A	1,5	1,1	0,7	0,5	0,47	0,211
YLR345W	25,1	6,4	40,6	17,0	1,62	0,141
YLR346C	9,3	2,6	9,8	8,3	1,06	0,909
YLR349W	1,8	1,3	6,4	9,6	3,57	0,378
YLR352W	17,1	6,9	16,0	5,2	0,94	0,810
YLR358C	8,1	7,3	7,1	7,6	0,88	0,857
YLR361C-A	35,8	19,8	67,1	42,9	1,87	0,233
YLR364C-A	0,1	0,2	0,1	0,3	0,97	0,982
YLR365W	0,4	0,3	0,2	0,4	0,48	0,444
YLR374C	0,0	0,0	0,3	0,6	#DIV/0!	0,356
YLR379W	1,0	1,0	0,5	0,6	0,50	0,418
YLR399W-A	1,3	1,2	0,5	0,7	0,42	0,303
YLR400W	4,8	1,9	4,4	3,4	0,92	0,850
YLR407W	47,7	19,9	29,6	13,1	0,62	0,179
YLR413W	206,7	58,1	22,1	11,4	0,11	0,001
YLR415C	3,2	3,5	1,5	1,5	0,46	0,402
YLR416C	0,0	0,1	0,4	0,8	7,68	0,422
YLR419W	12,7	9,0	17,8	16,0	1,40	0,598
YLR422W	30,4	15,3	45,4	9,4	1,49	0,146
YLR426W	30,3	6,5	15,9	7,4	0,53	0,027

YLR428C	3,9	2,3	2,7	1,9	0,70	0,465
YLR434C	0,1	0,1	0,2	0,4	3,53	0,516
YLR437C-A	1,4	1,9	1,8	2,2	1,26	0,808
YLR444C	0,5	0,6	0,0	0,0	0,00	0,180
YLR445W	0,7	1,1	0,3	0,4	0,36	0,474
YLR446W	2,0	1,3	2,4	1,8	1,22	0,707
YLR455W	37,3	19,3	27,5	13,2	0,74	0,432
YLR456W	29,3	13,2	28,1	5,2	0,96	0,871
YLR458W	0,1	0,1	0,2	0,2	2,52	0,482
YLR460C	2,5	1,2	4,3	4,0	1,72	0,424
YLR462W	0,0	0,0	0,0	0,0	#DIV/0!	#DIV/0!
YMC1	127,3	68,1	112,1	27,4	0,88	0,692
YMC2	241,8	18,5	128,2	24,7	0,53	0,000
YMD8	114,8	82,5	106,5	71,5	0,93	0,883
YME1	7,8	4,7	6,2	6,0	0,80	0,702
YME2	31,7	13,2	54,5	46,9	1,72	0,385
YML002W	12,6	6,1	26,8	8,6	2,13	0,036
YML003W	0,2	0,2	0,3	0,5	1,78	0,644
YML007C-A	3,2	3,7	7,5	8,4	2,36	0,384
YML009C-A	3,8	3,2	3,0	3,6	0,78	0,741
YML009W-B	82,5	25,9	103,9	18,8	1,26	0,230
YML012C-A	0,2	0,2	0,7	0,7	3,49	0,223
YML018C	124,7	36,1	83,2	11,1	0,67	0,070
YML020W	39,3	13,0	21,9	10,3	0,56	0,080
YML034C-A	0,5	0,7	5,4	8,3	11,47	0,285
YML037C	1,0	1,0	0,7	0,6	0,72	0,657
YML047W-A	0,0	0,0	0,8	0,5	#DIV/0!	0,027
YML053C	69,3	27,0	97,7	34,2	1,41	0,240
YML054C-A	0,5	0,6	0,0	0,0	0,00	0,111
YML079W	100,9	6,9	117,8	34,9	1,17	0,378
YML081W	95,9	31,7	48,2	10,6	0,50	0,029
YML082W	17,7	2,8	13,3	8,9	0,75	0,384
YML083C	0,7	0,9	0,4	0,3	0,50	0,463
YML084W	0,3	0,7	0,0	0,1	0,12	0,418
YML089C	0,2	0,3	0,5	0,6	3,04	0,363
YML094C-A	1,5	1,2	0,0	0,0	0,00	0,047
YML096W	18,3	9,4	10,2	9,3	0,56	0,269
YML099W-A	2,6	2,1	2,5	2,9	0,96	0,954
YML101C-A	2,4	2,7	0,2	0,2	0,08	0,153
YML108W	71,2	67,4	70,4	48,7	0,99	0,986
YML116W-A	43,8	22,2	60,4	48,6	1,38	0,556
YML119W	14,0	6,2	19,7	15,2	1,40	0,515
YML122C	1,4	0,7	1,0	0,9	0,70	0,486
YML131W	12,8	6,4	30,9	18,3	2,42	0,111
YML6	33,1	7,0	29,6	14,0	0,90	0,674
YMR007W	7,4	5,5	8,6	7,9	1,16	0,811
YMR010W	50,4	18,6	28,2	1,5	0,56	0,055
YMR013C-A	35,8	25,7	12,0	8,2	0,33	0,128
YMR018W	6,3	1,7	3,2	2,7	0,50	0,096
YMR027W	46,2	10,7	49,2	11,9	1,06	0,723

YMR031C	75,7	43,9	93,1	31,1	1,23	0,542
YMR034C	5,5	3,2	5,3	2,2	0,95	0,897
YMR045C	10,9	5,1	13,6	10,0	1,25	0,649
YMR046W-A	0,2	0,5	0,1	0,1	0,24	0,487
YMR057C	1,0	0,8	1,4	1,1	1,42	0,557
YMR074C	0,4	0,4	0,1	0,2	0,19	0,140
YMR082C	2,6	4,7	0,1	0,1	0,02	0,325
YMR084W	0,0	0,0	0,1	0,1	#DIV/0!	0,356
YMR085W	5,1	2,9	3,0	2,2	0,59	0,300
YMR086C-A	8,1	4,4	6,5	5,1	0,80	0,641
YMR086W	59,1	40,8	85,0	66,8	1,44	0,531
YMR087W	34,8	17,3	27,5	19,7	0,79	0,597
YMR090W	26,1	8,5	37,8	15,0	1,45	0,222
YMR099C	91,7	52,4	107,6	38,6	1,17	0,642
YMR1	10,7	6,4	9,0	7,9	0,84	0,750
YMR102C	8,3	7,5	11,6	19,5	1,40	0,762
YMR103C	29,8	22,2	74,2	43,7	2,49	0,120
YMR105W-A	0,9	0,8	1,2	1,0	1,31	0,679
YMR111C	26,9	9,5	52,5	26,0	1,95	0,114
YMR114C	25,0	10,9	58,2	23,4	2,33	0,042
YMR119W-A	29,4	28,7	7,4	8,2	0,25	0,190
YMR122C	0,3	0,5	0,1	0,1	0,24	0,394
YMR122W-A	91,8	56,4	142,9	58,1	1,56	0,253
YMR124W	23,4	10,6	25,8	17,3	1,11	0,816
YMR130W	59,0	17,9	25,7	9,9	0,44	0,018
YMR134W	68,5	15,0	70,1	30,0	1,02	0,927
YMR135W-A	0,1	0,2	0,0	0,0	0,00	0,356
YMR141W-A	0,5	0,4	0,2	0,2	0,37	0,269
YMR144W	20,8	8,3	3,3	2,7	0,16	0,007
YMR147W	4,8	5,4	3,7	3,4	0,76	0,735
YMR155W	14,8	5,3	8,6	6,2	0,58	0,181
YMR158C-A	16,9	7,3	43,8	53,4	2,59	0,357
YMR158W-B	0,1	0,1	0,0	0,0	0,00	0,356
YMR160W	7,9	4,2	19,0	9,3	2,39	0,074
YMR166C	9,1	3,1	13,0	4,0	1,43	0,178
YMR175W-A	0,0	0,0	0,3	0,5	#DIV/0!	0,266
YMR178W	106,5	54,4	117,7	61,3	1,11	0,792
YMR181C	35,0	22,0	56,7	35,3	1,62	0,338
YMR182W-A	10,1	5,9	10,5	8,0	1,04	0,943
YMR185W	44,9	11,0	28,8	21,2	0,64	0,227
YMR187C	6,7	4,7	4,8	4,0	0,71	0,555
YMR193C-A	1,3	1,3	0,3	0,5	0,20	0,176
YMR194C-A	1,8	1,2	2,7	2,3	1,50	0,522
YMR196W	31,8	16,9	40,3	3,6	1,27	0,360
YMR206W	10,5	4,0	8,1	6,2	0,78	0,551
YMR209C	113,0	18,6	120,0	20,9	1,06	0,634
YMR210W	9,9	3,5	9,1	3,3	0,92	0,745
YMR221C	23,0	14,4	9,3	8,4	0,40	0,150
YMR226C	770,3	371,8	930,0	405,1	1,21	0,583
YMR230W-A	22,5	10,7	1,1	1,0	0,05	0,007

YMR244C-A	167,9	95,8	191,9	57,4	1,14	0,682
YMR244W	12,1	3,6	13,2	7,4	1,10	0,788
YMR245W	3,3	2,1	1,2	1,4	0,37	0,144
YMR252C	29,3	7,1	24,8	10,2	0,85	0,494
YMR253C	6,6	4,5	23,5	14,0	3,59	0,060
YMR254C	0,0	0,1	0,0	0,0	0,00	0,356
YMR258C	36,0	14,8	65,0	41,6	1,81	0,237
YMR259C	16,2	9,1	22,9	22,5	1,42	0,599
YMR262W	23,4	7,9	22,6	5,4	0,96	0,865
YMR265C	12,8	2,2	10,4	7,3	0,81	0,549
YMR272W-A	1,3	1,0	0,4	0,4	0,32	0,168
YMR272W-B	5,1	2,8	2,9	2,4	0,56	0,264
YMR279C	1,2	0,5	0,3	0,3	0,22	0,020
YMR290W-A	0,1	0,2	0,0	0,1	0,27	0,506
YMR291W	55,8	35,8	57,6	26,1	1,03	0,940
YMR294W-A	6,4	6,6	4,9	4,9	0,76	0,729
YMR295C	426,6	261,5	326,6	114,6	0,77	0,510
YMR304C-A	0,9	1,0	1,0	1,1	1,08	0,927
YMR306C-A	0,5	0,5	0,1	0,2	0,21	0,206
YMR307C-A	24,0	20,3	6,2	5,9	0,26	0,145
YMR31	127,2	92,7	198,6	104,7	1,56	0,346
YMR310C	63,4	42,8	80,3	56,0	1,27	0,648
YMR315W	282,1	40,9	319,1	134,4	1,13	0,617
YMR315W-A	0,0	0,0	0,0	0,0	#DIV/0!	#DIV/0!
YMR316C-A	2,6	1,4	3,2	2,7	1,20	0,739
YMR316C-B	2,8	1,1	3,0	2,3	1,08	0,866
YMR317W	5,0	2,4	15,3	11,6	3,04	0,132
YMR320W	0,5	0,7	0,7	0,7	1,38	0,691
YMR321C	1,1	0,3	1,1	0,8	1,07	0,876
YMR324C	0,7	0,5	0,1	0,2	0,16	0,069
YND1	36,4	21,9	22,0	24,1	0,60	0,410
YNG1	22,5	4,1	20,6	13,1	0,92	0,792
YNG2	70,3	25,7	92,7	13,4	1,32	0,174
YNK1	556,1	174,5	575,4	227,3	1,03	0,897
YNL010W	428,0	152,9	443,0	188,3	1,04	0,906
YNL011C	21,5	8,5	20,3	4,2	0,94	0,810
YNL013C	0,1	0,2	0,0	0,0	0,00	0,356
YNL018C	0,8	0,8	2,2	2,9	2,56	0,414
YNL019C	4,7	3,2	3,9	3,1	0,83	0,727
YNL022C	92,3	46,7	177,8	70,9	1,93	0,091
YNL024C	21,5	23,3	24,1	22,8	1,12	0,880
YNL028W	0,0	0,0	0,1	0,2	#DIV/0!	0,356
YNL034W	0,1	0,1	0,3	0,5	4,15	0,488
YNL035C	44,1	21,8	43,1	17,3	0,98	0,946
YNL040W	90,9	41,5	119,3	52,8	1,31	0,430
YNL042W-B	0,1	0,2	0,1	0,2	0,71	0,824
YNL043C	7,0	4,3	2,3	2,0	0,33	0,095
YNL046W	105,4	40,9	83,9	21,7	0,80	0,389
YNL050C	24,7	20,5	21,2	21,5	0,86	0,825
YNL054W-B	5,6	5,3	10,0	7,3	1,78	0,373

YNL058C	23,8	6,4	56,9	14,6	2,39	0,006
YNL067W-B	0,1	0,3	0,0	0,1	0,21	0,471
YNL089C	2,2	1,9	4,4	6,9	2,01	0,564
YNL092W	12,6	6,1	28,7	10,8	2,29	0,041
YNL095C	39,0	28,5	27,8	18,7	0,71	0,535
YNL103W-A	1,5	0,5	0,4	0,3	0,25	0,009
YNL108C	143,2	41,5	128,3	9,4	0,90	0,511
YNL109W	0,2	0,2	0,1	0,2	0,74	0,799
YNL115C	32,0	12,0	32,4	21,9	1,01	0,980
YNL122C	124,8	65,0	153,7	67,6	1,23	0,560
YNL134C	48,6	28,5	210,8	149,0	4,34	0,076
YNL143C	16,2	8,4	20,2	6,0	1,25	0,472
YNL144C	660,8	316,5	572,8	152,5	0,87	0,634
YNL144W-A	0,2	0,3	0,6	0,8	3,38	0,408
YNL146W	3,2	1,6	18,7	6,0	5,84	0,003
YNL150W	7,1	5,5	7,5	8,6	1,05	0,944
YNL155W	65,0	11,6	97,0	38,2	1,49	0,160
YNL162W-A	24,3	2,7	8,9	6,0	0,37	0,003
YNL165W	26,3	20,0	11,1	13,1	0,42	0,251
YNL170W	0,1	0,2	0,0	0,0	0,00	0,152
YNL171C	1,5	0,9	1,2	1,4	0,80	0,714
YNL174W	12,9	14,2	17,7	20,2	1,37	0,711
YNL176C	48,1	27,7	41,6	7,4	0,87	0,667
YNL179C	0,6	0,5	1,2	0,9	1,85	0,336
YNL181W	48,8	13,4	12,3	8,5	0,25	0,004
YNL184C	66,2	10,7	61,6	49,2	0,93	0,862
YNL190W	92,1	90,2	119,1	79,4	1,29	0,670
YNL193W	38,7	10,7	17,0	11,1	0,44	0,031
YNL194C	1,4	1,6	16,1	27,1	11,57	0,321
YNL195C	5,6	2,6	8,5	7,3	1,52	0,479
YNL200C	37,4	13,7	31,9	5,2	0,85	0,483
YNL203C	1,7	1,3	0,2	0,3	0,10	0,073
YNL205C	3,9	2,4	1,3	1,2	0,32	0,094
YNL211C	35,4	16,1	48,8	18,1	1,38	0,310
YNL217W	81,4	26,7	115,0	26,7	1,41	0,126
YNL226W	0,0	0,1	0,0	0,1	0,87	0,928
YNL228W	25,4	22,2	34,1	24,4	1,34	0,618
YNL234W	8,5	3,5	6,9	5,0	0,81	0,614
YNL235C	2,9	1,9	4,1	4,4	1,39	0,649
YNL247W	172,9	61,1	177,9	54,5	1,03	0,906
YNL277W-A	0,0	0,0	0,1	0,3	#DIV/0!	0,356
YNL284C-B	0,0	0,0	0,0	0,0	#DIV/0!	#DIV/0!
YNL285W	0,1	0,2	0,0	0,0	0,00	0,356
YNL295W	35,2	23,2	41,1	23,2	1,17	0,731
YNL296W	0,0	0,0	0,0	0,0	#DIV/0!	#DIV/0!
YNL303W	7,0	4,2	3,4	2,6	0,49	0,195
YNL305C	84,2	31,3	134,9	64,2	1,60	0,205
YNL320W	34,7	14,6	28,8	4,0	0,83	0,464
YNL324W	1,1	1,0	3,2	4,4	2,99	0,385
YNL337W	0,0	0,1	0,0	0,0	0,00	0,356

YNL338W	14,1	2,3	6,8	5,0	0,48	0,038
YNR003W-A	1,8	1,3	0,8	0,8	0,44	0,236
YNR005C	0,6	0,8	0,1	0,2	0,22	0,313
YNR014W	33,8	9,2	77,8	15,2	2,30	0,003
YNR021W	460,5	170,4	336,9	108,2	0,73	0,267
YNR029C	38,3	21,5	43,2	29,8	1,13	0,798
YNR034W-A	28,4	10,7	227,8	206,4	8,03	0,102
YNR040W	60,9	30,2	99,8	35,4	1,64	0,146
YNR042W	0,5	0,6	0,6	0,6	1,23	0,800
YNR048W	22,5	13,2	32,4	28,5	1,44	0,553
YNR061C	202,6	96,3	147,3	80,5	0,73	0,412
YNR062C	0,5	0,8	1,0	0,7	1,82	0,443
YNR063W	0,7	0,5	1,4	1,1	2,02	0,306
YNR064C	1,8	0,6	7,7	11,0	4,21	0,325
YNR065C	8,8	2,2	24,8	19,2	2,83	0,148
YNR066C	0,3	0,2	1,2	0,9	4,14	0,086
YNR068C	7,3	5,1	15,5	8,0	2,13	0,134
YNR071C	1,2	0,9	0,5	0,7	0,46	0,318
YNR073C	0,0	0,0	0,0	0,0	#DIV/0!	#DIV/0!
YNR075C-A	0,0	0,0	0,0	0,0	#DIV/0!	#DIV/0!
YOL013W-A	93,5	90,0	36,1	15,1	0,39	0,255
YOL013W-B	0,0	0,0	0,0	0,0	#DIV/0!	#DIV/0!
YOL014W	28,8	14,0	45,7	13,0	1,58	0,129
YOL019W	61,5	34,1	6,4	4,8	0,10	0,019
YOL019W-A	0,0	0,1	0,0	0,0	0,00	0,356
YOL024W	1,5	1,3	0,5	0,4	0,33	0,179
YOL029C	30,0	13,3	22,3	14,9	0,74	0,469
YOL035C	2,1	2,1	3,3	3,1	1,59	0,543
YOL036W	118,3	42,1	155,2	31,9	1,31	0,212
YOL037C	1,1	0,8	2,4	2,3	2,18	0,316
YOL038C-A	267,3	312,2	245,3	281,5	0,92	0,920
YOL046C	0,5	0,2	0,1	0,1	0,15	0,018
YOL047C	4,6	3,7	9,0	6,1	1,97	0,263
YOL050C	0,9	0,8	0,4	0,4	0,46	0,308
YOL057W	146,0	66,9	112,2	28,2	0,77	0,388
YOL073C	20,4	20,3	40,5	59,8	1,98	0,549
YOL075C	66,7	23,0	55,7	28,3	0,84	0,570
YOL079W	2,4	2,8	0,5	0,5	0,22	0,224
YOL085C	0,2	0,5	0,4	0,3	1,63	0,627
YOL085W-A	0,2	0,2	0,3	0,5	1,28	0,846
YOL086W-A	99,5	43,7	67,1	23,6	0,67	0,239
YOL087C	35,7	17,2	41,6	13,7	1,16	0,612
YOL092W	584,5	255,7	422,2	257,9	0,72	0,406
YOL097W-A	3,2	1,1	8,0	8,7	2,47	0,320
YOL098C	125,1	45,5	127,1	61,5	1,02	0,960
YOL107W	14,4	4,4	8,1	5,4	0,56	0,119
YOL118C	1,0	1,7	8,8	17,2	8,60	0,405
YOL131W	0,5	0,2	2,4	1,7	5,20	0,059
YOL134C	1,7	1,8	1,2	1,6	0,70	0,682
YOL153C	7,4	4,1	40,9	40,7	5,55	0,152

YOL155W-A	0,3	0,4	0,5	0,5	1,47	0,639
YOL159C-A	1,0	0,3	0,4	0,5	0,47	0,153
YOL160W	3,6	1,0	0,9	0,7	0,26	0,005
YOL162W	18,5	6,7	22,3	7,1	1,20	0,473
YOL163W	0,0	0,0	0,2	0,2	#DIV/0!	0,157
YOL164W-A	0,0	0,0	0,0	0,0	#DIV/0!	#DIV/0!
YOL166C	3,6	3,0	0,4	0,3	0,12	0,074
YOP1	3,8	3,8	2,1	1,5	0,57	0,454
YOR011W-A	0,1	0,2	0,2	0,4	2,02	0,668
YOR012W	0,2	0,2	0,4	0,6	1,96	0,576
YOR015W	43,5	3,0	8,7	6,6	0,20	0,000
YOR019W	2,8	1,5	2,7	2,3	0,96	0,944
YOR022C	15,8	9,8	17,6	3,7	1,11	0,751
YOR029W	0,0	0,1	0,2	0,4	5,42	0,376
YOR032W-A	2,6	0,6	0,8	1,2	0,30	0,030
YOR041C	0,1	0,2	0,6	0,4	4,02	0,111
YOR050C	1,9	0,7	0,8	0,6	0,42	0,057
YOR052C	71,2	83,5	281,2	327,4	3,95	0,260
YOR053W	0,5	0,6	0,7	0,6	1,62	0,533
YOR055W	0,0	0,0	0,2	0,4	#DIV/0!	0,356
YOR059C	12,7	6,9	16,7	4,7	1,31	0,377
YOR060C	3,4	1,2	13,3	10,2	3,96	0,100
YOR062C	28,7	5,0	89,3	60,1	3,10	0,092
YOR072W	0,4	0,5	0,1	0,1	0,16	0,253
YOR072W-A	4,3	3,0	1,5	1,8	0,34	0,151
YOR072W-B	0,9	0,7	0,6	0,7	0,67	0,549
YOR082C	1,4	1,8	0,6	0,7	0,40	0,413
YOR093C	57,2	22,2	26,1	6,5	0,46	0,036
YOR097C	41,0	27,6	60,3	30,5	1,47	0,383
YOR1	33,0	17,0	25,8	5,4	0,78	0,454
YOR102W	0,6	0,3	0,7	0,7	1,10	0,870
YOR105W	36,1	22,2	35,6	5,2	0,99	0,966
YOR108C-A	1,7	1,3	0,9	1,1	0,55	0,412
YOR111W	41,4	16,3	55,0	28,6	1,33	0,440
YOR114W	17,1	9,0	2,7	2,3	0,16	0,021
YOR131C	76,1	7,7	99,2	55,4	1,30	0,441
YOR139C	0,5	0,4	0,2	0,4	0,49	0,386
YOR161C-C	1,9	2,5	0,6	1,1	0,30	0,368
YOR161W-B	0,5	0,6	0,4	0,5	0,76	0,776
YOR169C	4,6	5,3	4,7	5,5	1,03	0,969
YOR170W	2,3	1,8	2,4	2,4	1,05	0,945
YOR186C-A	0,1	0,2	0,0	0,0	0,00	0,356
YOR186W	2,0	1,0	1,3	0,9	0,65	0,345
YOR192C-B	0,1	0,2	0,0	0,0	0,00	0,356
YOR192C-C	255,5	170,0	228,3	66,8	0,89	0,776
YOR199W	7,3	5,5	4,2	3,8	0,58	0,396
YOR200W	0,2	0,3	0,0	0,0	0,00	0,356
YOR203W	1004,8	713,8	3042,4	2312,1	3,03	0,143
YOR214C	0,1	0,2	0,1	0,1	0,60	0,740
YOR218C	2,2	1,2	1,0	0,7	0,43	0,122

YOR223W	49,5	19,4	44,6	19,1	0,90	0,729
YOR225W	3,9	1,5	1,6	1,7	0,41	0,085
YOR228C	20,9	9,0	29,6	8,6	1,41	0,213
YOR238W	38,4	22,0	21,8	16,3	0,57	0,274
YOR246C	87,0	22,6	53,4	2,8	0,61	0,025
YOR248W	1572,0	293,7	491,0	95,6	0,31	0,000
YOR262W	32,7	4,1	25,8	18,6	0,79	0,492
YOR263C	0,5	1,0	0,3	0,3	0,68	0,775
YOR268C	0,0	0,0	0,2	0,2	#DIV/0!	0,205
YOR277C	7,5	3,0	2,7	2,3	0,37	0,047
YOR283W	89,6	38,0	100,0	31,3	1,12	0,688
YOR289W	17,2	6,0	52,9	13,9	3,07	0,003
YOR292C	51,6	13,4	84,5	37,2	1,64	0,147
YOR293C-A	3,8	4,1	7,2	8,4	1,91	0,488
YOR296W	37,6	24,8	27,4	9,0	0,73	0,469
YOR300W	0,0	0,0	0,0	0,0	#DIV/0!	#DIV/0!
YOR304C-A	62,5	64,1	57,6	60,7	0,92	0,915
YOR314W-A	0,4	0,7	0,0	0,0	0,00	0,223
YOR316C-A	0,0	0,1	0,3	0,4	5,85	0,250
YOR318C	51,6	25,7	33,3	22,6	0,65	0,326
YOR325W	6,3	2,2	2,2	1,6	0,35	0,023
YOR329W-A	1,1	1,0	7,4	5,3	6,89	0,059
YOR331C	38,1	38,7	34,2	38,6	0,90	0,891
YOR333C	1,4	1,9	1,0	0,9	0,69	0,686
YOR338W	5,2	0,9	11,5	7,8	2,21	0,161
YOR342C	67,2	36,7	31,2	24,9	0,46	0,156
YOR343C	1,5	1,0	4,0	3,0	2,61	0,173
YOR345C	0,4	0,4	0,1	0,1	0,15	0,191
YOR352W	41,0	14,6	32,5	21,6	0,79	0,539
YOR356W	58,0	37,0	29,0	19,1	0,50	0,213
YOR364W	0,4	0,3	0,9	0,8	2,34	0,294
YOR365C	1,5	1,0	1,4	1,3	0,95	0,934
YOR366W	0,2	0,3	0,1	0,3	0,59	0,686
YOR378W	4,6	1,1	1,0	0,7	0,23	0,002
YOR379C	0,0	0,1	0,0	0,0	0,00	0,356
YOR381W-A	4,6	3,4	2,2	3,5	0,48	0,364
YOR385W	33,6	14,2	41,8	23,3	1,24	0,572
YOR387C	1,2	0,9	0,4	0,3	0,29	0,140
YOR389W	32,0	10,4	59,6	53,5	1,86	0,349
YOR390W	41,2	4,9	11,2	8,0	0,27	0,001
YOR392W	0,0	0,1	0,4	0,6	10,84	0,327
YOR394C-A	6,5	2,7	0,7	1,5	0,11	0,009
YOS1	36,2	11,2	5,6	4,1	0,16	0,002
YOS9	29,2	15,0	38,2	34,7	1,31	0,652
YOX1	71,1	20,7	4,1	3,1	0,06	0,001
YPC1	28,4	6,5	25,9	8,7	0,91	0,661
YPD1	148,5	65,6	178,5	76,4	1,20	0,574
YPI1	23,3	17,0	55,9	27,8	2,40	0,091
YPK1	135,8	103,6	76,6	63,1	0,56	0,367
YPK2	1,7	1,4	4,4	4,5	2,63	0,292

YPK9	134,2	75,4	70,8	34,0	0,53	0,176
YPL014W	20,6	4,9	21,7	14,3	1,05	0,888
YPL025C	5,0	1,8	2,3	1,7	0,46	0,070
YPL034W	30,6	9,4	26,9	18,7	0,88	0,736
YPL035C	2,4	2,4	2,2	1,8	0,94	0,924
YPL038W-A	1,6	1,2	0,6	0,6	0,38	0,179
YPL039W	56,2	22,9	40,5	14,2	0,72	0,288
YPL041C	7,7	1,0	9,7	11,0	1,27	0,723
YPL044C	5,9	5,4	4,0	4,1	0,67	0,588
YPL060C-A	0,1	0,2	0,2	0,4	2,00	0,670
YPL062W	0,7	0,8	0,6	0,5	0,92	0,919
YPL066W	52,2	24,9	44,2	13,5	0,85	0,595
YPL067C	68,5	21,6	15,0	12,1	0,22	0,005
YPL068C	21,3	13,0	7,3	5,1	0,34	0,093
YPL071C	51,6	31,4	43,6	37,0	0,85	0,754
YPL073C	0,0	0,0	4,2	8,4	#DIV/0!	0,356
YPL077C	0,7	0,5	0,3	0,7	0,48	0,410
YPL088W	92,3	29,4	133,4	60,2	1,45	0,266
YPL102C	0,0	0,0	0,0	0,0	#DIV/0!	#DIV/0!
YPL107W	26,5	6,7	40,3	20,8	1,52	0,253
YPL108W	32,3	19,4	47,4	17,0	1,47	0,285
YPL109C	5,8	2,2	3,6	2,6	0,62	0,246
YPL113C	8,5	1,0	5,6	4,2	0,65	0,220
YPL114W	1,0	0,7	1,2	0,9	1,20	0,743
YPL135C-A	2,2	2,5	3,0	3,6	1,36	0,728
YPL150W	15,1	3,7	21,1	15,3	1,40	0,475
YPL152W-A	0,0	0,1	0,0	0,0	0,00	0,356
YPL162C	24,4	6,2	41,5	18,6	1,70	0,131
YPL168W	11,1	4,1	8,0	5,4	0,72	0,398
YPL182C	0,6	0,8	0,1	0,1	0,12	0,231
YPL185W	0,9	0,8	1,9	1,5	2,20	0,265
YPL191C	24,8	9,7	32,2	1,7	1,30	0,182
YPL199C	64,5	47,5	46,6	33,0	0,72	0,557
YPL216W	18,0	3,7	18,1	12,3	1,01	0,987
YPL225W	515,6	329,9	573,6	222,0	1,11	0,780
YPL229W	21,4	10,9	67,4	33,5	3,14	0,040
YPL236C	22,7	13,9	26,1	14,7	1,15	0,745
YPL245W	25,5	12,6	27,2	25,3	1,06	0,911
YPL247C	17,2	13,3	68,4	67,8	3,97	0,189
YPL251W	6,9	5,9	6,5	6,4	0,94	0,933
YPL257W	6,5	2,1	10,1	6,9	1,55	0,361
YPL260W	70,7	61,5	131,0	99,0	1,85	0,341
YPL264C	24,6	12,1	29,2	10,7	1,19	0,583
YPL272C	9,3	6,4	3,9	3,2	0,42	0,181
YPL276W	0,5	0,8	0,0	0,0	0,00	0,225
YPL277C	0,2	0,2	0,1	0,2	0,82	0,873
YPL278C	0,8	0,9	0,4	0,5	0,48	0,461
YPL279C	0,1	0,1	0,0	0,0	0,00	0,356
YPP1	43,6	21,7	38,9	6,6	0,89	0,693
YPR003C	20,1	9,6	14,9	11,1	0,74	0,501

YPR010C-A	195,8	86,0	207,5	106,7	1,06	0,871
YPR011C	39,9	15,7	27,8	3,7	0,70	0,182
YPR012W	0,1	0,2	0,4	0,3	3,08	0,263
YPR013C	8,0	4,0	16,6	5,0	2,08	0,035
YPR015C	43,4	29,5	66,5	59,1	1,53	0,511
YPR016W-A	9,2	6,7	3,3	2,4	0,36	0,147
YPR022C	82,2	23,3	42,7	5,4	0,52	0,016
YPR027C	1,6	1,0	1,9	1,9	1,20	0,777
YPR039W	1,2	0,8	1,5	1,2	1,26	0,672
YPR050C	2,3	2,2	1,0	1,3	0,44	0,363
YPR059C	2,4	2,3	2,8	3,2	1,17	0,840
YPR063C	58,0	6,9	52,0	8,2	0,90	0,308
YPR064W	1,8	0,4	1,5	1,1	0,81	0,569
YPR071W	20,1	1,7	36,5	14,4	1,81	0,065
YPR074W-A	0,0	0,1	0,0	0,1	0,87	0,928
YPR076W	0,7	0,9	0,0	0,0	0,00	0,203
YPR077C	2,7	2,3	4,4	2,9	1,62	0,400
YPR078C	0,0	0,0	0,0	0,1	#DIV/0!	0,356
YPR084W	96,8	40,1	57,6	12,1	0,60	0,111
YPR089W	27,3	9,7	30,7	10,4	1,12	0,652
YPR091C	50,5	22,3	63,3	31,0	1,25	0,526
YPR097W	41,3	19,2	38,0	10,2	0,92	0,768
YPR099C	3,4	0,7	2,0	1,4	0,59	0,111
YPR1	102,9	50,2	204,0	108,5	1,98	0,142
YPR108W-A	58,1	53,9	8,0	5,9	0,14	0,114
YPR109W	52,1	32,8	58,4	36,9	1,12	0,806
YPR114W	85,0	17,0	120,9	43,5	1,42	0,175
YPR117W	43,7	13,8	81,9	20,1	1,88	0,020
YPR123C	0,7	0,7	0,9	1,0	1,21	0,808
YPR126C	3,0	2,2	1,5	1,5	0,50	0,297
YPR127W	74,7	30,6	129,0	57,0	1,73	0,145
YPR136C	1,8	2,0	0,1	0,2	0,07	0,159
YPR142C	24,0	18,5	16,0	13,9	0,67	0,516
YPR145C-A	7,3	8,4	4,1	4,5	0,56	0,524
YPR146C	0,2	0,4	0,0	0,0	0,00	0,356
YPR147C	113,8	62,8	92,5	29,9	0,81	0,563
YPR148C	151,4	85,0	212,3	159,5	1,40	0,525
YPR150W	0,7	0,5	0,9	0,8	1,20	0,773
YPR153W	9,7	1,2	4,4	3,2	0,45	0,020
YPR157W	4,7	2,7	31,9	21,4	6,87	0,045
YPR159C-A	1,0	0,6	0,9	1,1	0,92	0,905
YPR160C-A	0,7	0,7	0,0	0,1	0,06	0,120
YPR160W-A	0,2	0,3	0,3	0,2	1,85	0,519
YPR169W-A	14,3	8,4	8,6	7,8	0,61	0,364
YPR170C	6,0	4,4	4,6	3,5	0,76	0,623
YPR170W-A	686,0	392,1	292,2	252,3	0,43	0,142
YPR172W	65,4	8,4	104,5	16,1	1,60	0,005
YPR174C	20,9	7,0	16,6	11,6	0,79	0,545
YPR177C	0,1	0,1	0,0	0,1	0,54	0,697
YPR196W	1,6	0,3	2,0	1,5	1,25	0,629

YPR197C	1,3	1,1	0,7	0,8	0,56	0,452
YPS1	24,4	5,4	55,9	26,6	2,29	0,059
YPS3	21,8	7,3	8,8	7,1	0,41	0,045
YPS6	3,5	1,0	6,8	4,7	1,94	0,218
YPS7	105,1	45,9	108,0	27,4	1,03	0,917
YPT1	352,5	171,7	382,1	115,7	1,08	0,785
YPT10	23,9	17,6	34,9	36,9	1,46	0,610
YPT11	17,1	7,2	4,3	3,9	0,25	0,020
YPT31	205,5	152,0	190,6	169,2	0,93	0,900
YPT32	71,9	25,0	29,6	17,0	0,41	0,031
YPT35	14,6	8,4	17,1	11,8	1,17	0,745
YPT52	147,9	104,9	162,4	91,8	1,10	0,843
YPT53	1,4	1,1	1,8	2,0	1,27	0,749
YPT6	13,9	6,6	33,3	13,6	2,40	0,042
YPT7	186,3	128,1	109,5	64,1	0,59	0,325
YRA1	95,0	35,4	140,4	87,9	1,48	0,375
YRA2	103,7	59,1	76,1	32,1	0,73	0,444
YRB1	1242,2	561,0	1139,9	571,4	0,92	0,807
YRB30	29,5	14,6	42,8	16,1	1,45	0,268
YRF1-1	10,5	3,8	10,6	13,2	1,00	0,996
YRF1-2	0,2	0,3	0,2	0,5	1,03	0,981
YRM1	28,0	29,8	41,5	37,8	1,48	0,595
YRO2	49,6	26,5	1147,5	1019,0	23,16	0,075
YRR1	12,7	11,4	24,4	26,4	1,92	0,446
YSA1	166,6	87,0	391,6	236,2	2,35	0,124
YSC83	41,3	19,9	36,4	21,7	0,88	0,752
YSC84	55,4	27,4	82,6	48,0	1,49	0,363
YSF3	52,5	10,3	45,3	14,6	0,86	0,451
YSH1	41,9	14,7	38,8	13,4	0,93	0,764
YSP1	22,8	13,6	25,2	17,7	1,11	0,833
YSP2	36,0	7,6	53,3	17,4	1,48	0,117
YSP3	34,1	12,0	38,9	19,0	1,14	0,682
YSR3	15,8	4,8	5,0	3,8	0,32	0,013
YSW1	7,6	6,1	3,6	3,0	0,48	0,286
YSY6	2,2	1,1	0,2	0,3	0,07	0,012
YTA12	191,0	38,2	246,8	59,5	1,29	0,165
YTA6	28,1	8,7	32,5	11,2	1,16	0,560
YTA7	151,9	52,6	174,3	85,7	1,15	0,672
YTH1	51,2	22,3	52,7	31,9	1,03	0,944
YTM1	103,4	68,6	68,2	31,0	0,66	0,385
YTP1	2,1	0,7	1,2	1,5	0,58	0,332
YUR1	12,4	4,0	6,8	6,0	0,55	0,170
YVC1	53,2	9,8	45,3	9,4	0,85	0,290
YVH1	68,1	33,9	62,9	31,7	0,92	0,830
ZAP1	41,2	30,7	63,5	42,9	1,54	0,430
ZDS1	20,7	9,1	16,8	2,4	0,81	0,438
ZDS2	33,8	21,1	5,1	4,6	0,15	0,038
ZIM17	81,5	46,4	47,3	17,4	0,58	0,217
ZIP1	10,2	4,7	19,7	5,1	1,93	0,033
ZIP2	4,7	2,4	15,3	10,4	3,23	0,093

ZPR1	72,8	16,9	151,6	42,2	2,08	0,013
ZPS1	13,6	2,1	11,6	3,7	0,85	0,367
ZRC1	162,9	49,5	144,0	43,4	0,88	0,587
ZRG17	31,5	11,7	33,4	12,7	1,06	0,831
ZRG8	26,0	5,3	40,6	18,5	1,56	0,180
ZRT1	84,5	31,3	108,6	63,0	1,28	0,519
ZRT2	133,0	59,4	66,0	56,5	0,50	0,153
ZRT3	78,1	35,7	100,4	51,0	1,29	0,500
ZTA1	21,0	11,3	43,0	35,2	2,05	0,279
ZUO1	1618,2	1113,0	1992,3	1071,7	1,23	0,645
ZWF1	66,1	34,4	283,0	91,5	4,28	0,004
