# Supplementary material for: Transcriptional response of Saccharomyces cerevisiae to potassium starvation
Source: BMC Genomics. 2014 Nov 29;15(1):1040. doi: 10.1186/1471-2164-15-1040 (PMC4289377; doi:10.1186/1471-2164-15-1040)
Supplement: Supplementary file 2 — Additional file 2: Effect of potassium starvation on antisense transcript levels determined by SAGE tag sequencing. (PDF 283 KB) [file 12864_2014_6863_MOESM2_ESM.pdf]

Additional file 2. Effect of potassium starvation on antisense transcript levels determined by SAGE tag sequencing

Annotation	Average 50 mM (reads per million)	SD 50 mM	Average 0 mM (reads per million)	SD 0 mM	Fold change 0 mM/50 mM	P-value
AAC1	14,3	4,8	8,3	7,6	0,6	0,2327
AAC3	22,8	19,5	54,9	52,1	2,4	0,2933
AAD10	19,6	4,8	35,8	29,0	1,8	0,3112
AAD14	23,8	14,8	40,5	36,4	1,7	0,4293
AAD15	9,8	4,5	8,0	6,6	0,8	0,6751
AAD16	1,7	3,4	3,6	4,6	2,1	0,5222
AAD3	10,7	8,2	8,2	5,7	0,8	0,6377
AAD4	2,3	2,7	6,3	4,8	2,8	0,1962
AAD6	1,1	2,3	0,0	0,0	0,0	0,3559
AAH1	519,3	397,4	157,4	173,4	0,3	0,1461
AAP1	46,7	26,0	160,9	94,5	3,4	0,0586
AAR2	1,1	2,1	0,0	0,0	0,0	0,3559
AAT1	10,9	4,2	40,5	35,1	3,7	0,1451
AAT2	89,8	45,1	49,6	44,2	0,6	0,2498
ABD1	7,8	4,6	9,2	11,2	1,2	0,8193
ABF1	31,2	14,3	18,4	19,7	0,6	0,3338
ABM1	308,4	84,9	2948,9	1422,1	9,6	0,0100
ABP1	235,3	201,1	269,8	193,0	1,1	0,8128
ABP140	199,2	129,3	191,8	130,6	1,0	0,9388
ABZ1	11,1	7,3	28,6	23,1	2,6	0,1994
ABZ2	4137,4	2006,6	925,8	693,9	0,2	0,0232
ACA1	66,8	15,9	84,8	57,1	1,3	0,5661
ACB1	130,0	81,5	54,5	42,2	0,4	0,1512
ACC1	618,0	521,3	263,5	180,9	0,4	0,2462
ACE2	30,6	7,1	52,5	19,1	1,7	0,0750
ACF2	90,7	42,4	58,3	64,5	0,6	0,4343
ACF4	46,2	18,9	60,2	41,3	1,3	0,5595
ACH1	106,7	33,8	389,1	90,2	3,6	0,0011
ACK1	13,4	8,4	6,1	6,2	0,5	0,2120
ACN9	12,5	13,8	3,0	4,5	0,2	0,2402
ACO1	253,3	186,5	144,0	131,6	0,6	0,3754
ACO2	222,6	132,5	171,7	142,8	0,8	0,6199
ACP1	192,5	98,3	390,2	40,2	2,0	0,0098
ACS1	74,5	44,8	79,9	114,1	1,1	0,9326
ACS2	84,3	66,6	37,3	33,9	0,4	0,2550
ACT1	1478,0	393,9	1740,1	576,5	1,2	0,4811
ADA2	94,0	69,7	13,1	18,7	0,1	0,0662
ADD37	3,9	2,8	3,0	4,5	0,8	0,7624
ADE1	133,6	70,2	59,7	60,5	0,4	0,1618
ADE12	2320,8	1325,2	1122,3	544,4	0,5	0,1453
ADE13	540,4	325,4	257,8	129,3	0,5	0,1575
ADE16	552,7	82,1	179,7	120,6	0,3	0,0022

ADE17	615,4	431,7	167,0	167,6	0,3	0,1009
ADE2	44,2	41,3	10,6	12,6	0,2	0,1701
ADE3	363,9	236,5	479,6	363,6	1,3	0,6127
ADE4	398,1	70,9	149,0	40,8	0,4	0,0009
ADE5,7	262,9	185,5	140,0	104,7	0,5	0,2924
ADE6	163,9	88,3	96,4	72,6	0,6	0,2826
ADE8	181,3	87,2	84,5	57,0	0,5	0,1125
ADH1	4901,3	5434,2	5135,6	5021,5	1,0	0,9516
ADH2	168,8	101,5	149,6	33,5	0,9	0,7320
ADH3	523,6	344,3	500,3	429,9	1,0	0,9354
ADH4	21,5	9,7	15,2	16,9	0,7	0,5422
ADH5	122,0	97,4	179,7	198,9	1,5	0,6206
ADH6	124,8	49,9	292,5	76,3	2,3	0,0104
ADH7	35,7	25,3	58,3	45,4	1,6	0,4180
ADI1	9,8	7,0	17,6	20,6	1,8	0,4976
ADK1	440,0	221,2	545,5	234,2	1,2	0,5368
ADK2	124,0	29,6	61,9	41,8	0,5	0,0516
ADO1	151,7	67,5	124,5	110,7	0,8	0,6893
ADP1	72,6	41,9	51,5	37,8	0,7	0,4828
ADR1	27,1	18,1	28,8	20,0	1,1	0,9040
ADY2	7,9	5,4	11,0	7,6	1,4	0,5318
ADY3	66,4	15,4	18,1	22,4	0,3	0,0119
ADY4	162,5	15,6	147,3	98,5	0,9	0,7700
AEP1	5,5	1,6	7,3	6,0	1,3	0,5843
AEP2	22,9	14,4	15,8	12,0	0,7	0,4797
AEP3	2,8	2,4	3,2	4,8	1,1	0,8991
AFG1	14,8	6,2	10,7	10,1	0,7	0,5196
AFG2	12,5	12,3	17,6	18,5	1,4	0,6626
AFG3	20,5	6,9	7,1	8,2	0,3	0,0468
AFI1	80,9	40,4	91,6	39,7	1,1	0,7185
AFR1	72,5	58,2	154,9	32,6	2,1	0,0485
AFT1	49,1	16,0	49,3	35,4	1,0	0,9928
AFT2	62,8	15,0	32,1	31,9	0,5	0,1324
AGA1	45,9	23,1	441,2	79,5	9,6	0,0001
AGC1	12,5	8,0	7,2	8,6	0,6	0,4074
AGE1	17,9	7,7	141,2	227,9	7,9	0,3212
AGE2	17,9	13,3	12,6	12,4	0,7	0,5851
AGP1	82,4	35,3	128,8	145,1	1,6	0,5568
AGP2	100,8	26,3	48,5	33,4	0,5	0,0491
AGP3	19,7	9,6	35,7	26,0	1,8	0,2909
AGX1	43,0	26,5	73,6	78,8	1,7	0,4904
AHA1	99,6	75,7	107,4	80,0	1,1	0,8921
AHC1	25,2	16,6	28,9	30,8	1,1	0,8395
AHP1	1187,2	362,6	2405,8	612,1	2,0	0,0140
AHT1	5,4	2,1	18,9	14,6	3,5	0,1174
AIF1	37,3	11,9	20,2	18,6	0,5	0,1733
AIM1	17,6	20,8	6,8	5,9	0,4	0,3540
AIM10	3,0	4,4	5,9	7,1	2,0	0,5134
AIM11	9,8	1,7	14,0	10,1	1,4	0,4494
AIM13	2,1	2,8	0,0	0,0	0,0	0,1901

AIM14	23,6	19,7	19,7	17,0	0,8	0,7708
AIM17	90,1	17,7	331,9	48,9	3,7	0,0001
AIM18	77,3	19,4	39,6	27,0	0,5	0,0639
AIM19	11,2	3,1	34,3	23,7	3,1	0,1023
AIM2	9,7	5,6	10,4	9,4	1,1	0,9105
AIM21	19,6	9,5	12,7	13,7	0,7	0,4441
AIM22	23,3	7,2	16,9	11,6	0,7	0,3813
AIM23	48,6	28,4	98,8	65,9	2,0	0,2115
AIM24	11,0	8,7	12,7	19,0	1,2	0,8752
AIM25	25,3	7,8	12,0	9,3	0,5	0,0697
AIM26	7,7	7,8	11,2	15,6	1,5	0,7005
AIM27	71,9	37,3	51,1	44,9	0,7	0,5034
AIM29	106,8	40,0	95,7	28,3	0,9	0,6663
AIM3	7,3	5,3	15,4	10,8	2,1	0,2310
AIM31	29,9	13,8	27,5	23,2	0,9	0,8648
AIM32	43,9	29,0	31,3	28,3	0,7	0,5555
AIM33	75,8	45,0	48,7	44,0	0,6	0,4220
AIM34	46,2	23,2	81,2	85,6	1,8	0,4600
AIM36	24,2	9,2	95,0	95,3	3,9	0,1896
AIM37	873,2	686,9	399,8	284,5	0,5	0,2499
AIM38	54,3	41,8	33,8	34,6	0,6	0,4790
AIM39	8,2	5,4	3,7	3,1	0,5	0,1984
AIM41	310,8	78,7	389,2	261,5	1,3	0,5868
AIM43	21,4	23,8	15,8	11,2	0,7	0,6822
AIM44	42,8	28,5	39,7	27,2	0,9	0,8811
AIM45	20,9	15,8	17,7	14,4	0,8	0,7698
AIM46	23,7	21,0	31,2	26,0	1,3	0,6674
AIM5	16,4	6,6	15,7	14,3	1,0	0,9315
AIM6	23,5	21,0	38,7	29,1	1,6	0,4298
AIM7	151,7	40,3	110,0	77,8	0,7	0,3782
AIM9	38,2	32,7	38,9	29,4	1,0	0,9769
AIP1	30,4	24,1	29,7	27,6	1,0	0,9697
AIR1	33,3	16,7	169,7	238,3	5,1	0,2968
AIR2	13,7	2,2	5,0	5,5	0,4	0,0254
AKL1	23,0	19,0	84,5	93,4	3,7	0,2445
AKR1	133,8	91,8	219,2	36,5	1,6	0,1347
AKR2	36,2	6,7	78,7	122,4	2,2	0,5138
ALA1	164,5	122,9	92,6	62,1	0,6	0,3368
ALD2	63,7	32,9	575,0	812,0	9,0	0,2551
ALD3	72,7	36,7	52,8	55,2	0,7	0,5717
ALD4	280,6	154,1	248,1	169,9	0,9	0,7865
ALD5	167,3	57,4	186,8	60,9	1,1	0,6588
ALD6	255,9	137,5	184,2	10,0	0,7	0,3384
ALE1	48,6	29,4	25,6	18,8	0,5	0,2348
ALF1	8,6	3,6	6,2	7,3	0,7	0,5726
ALG1	191,9	44,4	120,9	90,0	0,6	0,2069
ALG11	36,5	22,9	17,3	14,5	0,5	0,2067
ALG12	32,2	11,3	14,8	19,2	0,5	0,1703
ALG13	6,5	7,6	1,8	3,6	0,3	0,3046
ALG14	67,4	34,3	144,6	280,8	2,1	0,6047

ALG2	264,8	149,0	101,3	72,6	0,4	0,0961
ALG3	6,4	7,4	5,4	9,1	0,8	0,8734
ALG5	27,3	14,9	6,9	5,2	0,3	0,0417
ALG6	100,5	51,0	176,3	47,1	1,8	0,0719
ALG7	62,0	19,7	18,7	14,3	0,3	0,0119
ALG8	59,9	65,2	155,2	256,1	2,6	0,4977
ALG9	65,3	32,0	46,5	34,6	0,7	0,4564
ALK1	34,1	4,8	46,6	33,3	1,4	0,4862
ALK2	37,6	11,5	34,2	23,3	0,9	0,8044
ALO1	310,7	117,8	325,5	125,3	1,0	0,8685
ALP1	0,8	1,5	1,3	2,6	1,7	0,7273
ALR1	29,8	13,0	30,0	24,6	1,0	0,9909
ALR2	3,1	2,8	7,9	5,8	2,6	0,1834
ALT1	301,7	235,8	643,0	755,5	2,1	0,4215
ALT2	52,2	30,8	10,1	8,3	0,2	0,0382
ALY1	17,2	6,9	13,2	13,1	0,8	0,6060
ALY2	67,7	75,7	35,2	23,9	0,5	0,4433
AMA1	0,0	0,0	0,0	0,0	#DIV/0!	#DIV/0!
AMD1	14,5	8,1	22,8	15,4	1,6	0,3754
AMD2	121,4	52,0	59,0	43,7	0,5	0,1158
AME1	13,0	11,9	6,9	5,7	0,5	0,3965
AMN1	127,8	69,2	94,3	65,0	0,7	0,5077
AMS1	93,3	44,7	256,2	176,1	2,7	0,1232
ANB1	27,0	9,4	83,3	82,4	3,1	0,2233
ANP1	30,9	19,6	31,9	31,0	1,0	0,9590
ANS1	1,2	2,4	4,1	2,9	3,5	0,1647
ANT1	33,8	26,2	7,5	5,4	0,2	0,0968
AOS1	170,8	117,2	138,7	102,2	0,8	0,6939
APA1	427,7	36,5	545,8	93,0	1,3	0,0561
APA2	12,3	9,0	32,2	33,5	2,6	0,2944
APC1	9,2	7,0	3,8	6,0	0,4	0,2892
APC11	78,2	51,2	39,8	42,9	0,5	0,2941
APC2	116,1	80,4	39,2	31,5	0,3	0,1253
APC4	11,4	5,2	4,9	5,7	0,4	0,1410
APC5	10,5	10,5	21,4	22,2	2,0	0,4095
APC9	3,8	4,5	4,3	4,0	1,1	0,8741
APD1	54,2	24,1	67,3	54,4	1,2	0,6751
APE2	63,1	23,2	62,1	42,4	1,0	0,9666
APE3	136,7	108,4	122,1	98,5	0,9	0,8480
API2	1,5	2,9	6,7	5,5	4,5	0,1471
APJ1	68,1	20,0	41,9	37,4	0,6	0,2626
APL1	15,5	16,3	39,2	30,0	2,5	0,2146
APL2	80,1	68,3	206,4	295,0	2,6	0,4359
APL3	18,2	16,9	11,2	12,1	0,6	0,5304
APL4	120,5	33,1	56,0	38,7	0,5	0,0444
APL5	242,5	145,6	251,4	168,1	1,0	0,9393
APL6	5,7	6,6	3,0	4,5	0,5	0,5237
APM1	48,0	7,6	26,8	18,7	0,6	0,0796
APM2	22,5	15,3	12,2	10,5	0,5	0,3104
APM3	27,5	10,5	17,2	17,0	0,6	0,3435

APM4	67,5	16,7	56,0	38,4	0,8	0,6029
APN1	138,2	49,6	209,6	171,9	1,5	0,4552
APN2	106,9	50,7	32,3	27,1	0,3	0,0411
APP1	27,5	15,6	26,6	28,0	1,0	0,9580
APQ12	138,9	52,3	82,4	57,4	0,6	0,1962
APS2	32,0	24,8	19,5	22,7	0,6	0,4866
APS3	74,7	42,5	171,5	139,8	2,3	0,2333
APT1	79,6	75,3	50,1	55,2	0,6	0,5505
APT2	92,7	51,1	45,6	33,0	0,5	0,1730
AQR1	448,1	492,1	120,0	112,4	0,3	0,2413
AQY1	89,3	45,1	98,9	50,2	1,1	0,7856
AQY2	14,7	11,9	8,4	9,1	0,6	0,4285
ARA1	87,2	22,3	59,4	42,1	0,7	0,2882
ARA2	61,2	23,0	80,1	76,7	1,3	0,6528
ARB1	94,9	49,5	54,8	50,8	0,6	0,3006
ARC1	153,4	121,1	237,3	119,5	1,5	0,3617
ARC18	46,9	53,9	53,3	49,5	1,1	0,8662
ARC19	114,0	65,7	214,1	61,7	1,9	0,0681
ARC35	93,3	46,0	69,1	62,0	0,7	0,5541
ARC40	152,5	38,7	68,2	51,8	0,4	0,0402
ARD1	46,6	33,8	37,9	39,8	0,8	0,7482
ARE1	5,6	8,4	6,2	5,7	1,1	0,9142
ARE2	3,1	2,1	12,3	11,4	4,0	0,1620
ARF1	656,3	290,1	405,4	316,7	0,6	0,2870
ARF2	732,1	131,1	488,6	310,8	0,7	0,1990
ARF3	221,1	72,0	59,8	41,3	0,3	0,0081
ARG1	556,1	353,8	137,6	145,1	0,2	0,0712
ARG2	11,1	8,7	10,1	8,3	0,9	0,8664
ARG3	656,4	526,0	88,9	49,3	0,1	0,0753
ARG4	142,5	98,8	163,8	173,4	1,1	0,8381
ARG5,6	944,0	497,2	178,9	126,4	0,2	0,0245
ARG7	463,4	365,0	252,1	166,8	0,5	0,3329
ARG8	495,1	292,0	79,1	66,2	0,2	0,0320
ARG80	116,2	134,0	84,4	85,4	0,7	0,7029
ARG81	2,1	2,8	0,7	1,3	0,3	0,3972
ARG82	28,1	16,8	23,0	19,2	0,8	0,7002
ARH1	36,6	13,0	19,4	13,5	0,5	0,1155
ARI1	18,3	13,1	5,0	4,1	0,3	0,1012
ARK1	353,1	59,3	233,8	227,5	0,7	0,3490
ARL1	205,2	101,2	68,6	48,9	0,3	0,0510
ARL3	9,4	8,2	2,4	4,7	0,3	0,1882
ARN1	37,9	16,5	611,2	518,9	16,1	0,0693
ARN2	26,0	11,1	21,1	16,5	0,8	0,6388
ARO1	57,6	40,1	102,3	92,0	1,8	0,4073
ARO10	296,4	221,4	114,4	90,5	0,4	0,1788
ARO2	71,0	33,5	77,0	66,5	1,1	0,8767
ARO3	209,0	129,4	282,5	198,7	1,4	0,5585
ARO4	308,5	243,4	80,9	58,0	0,3	0,1187
ARO7	21,8	19,3	26,0	20,7	1,2	0,7745
ARO8	934,6	383,6	823,3	264,0	0,9	0,6498

ARO80	9,8	6,0	29,1	25,4	3,0	0,1900
ARP1	22,1	9,9	6,0	6,5	0,3	0,0343
ARP10	13,7	6,6	27,3	33,4	2,0	0,4563
ARP2	187,9	126,0	173,4	152,9	0,9	0,8882
ARP3	156,7	49,8	257,3	187,8	1,6	0,3405
ARP4	61,2	26,4	23,2	19,4	0,4	0,0598
ARP5	179,6	137,5	189,0	148,4	1,1	0,9291
ARP6	31,4	11,0	46,1	33,6	1,5	0,4372
ARP7	32,8	11,2	28,1	24,8	0,9	0,7365
ARP8	95,0	55,2	88,1	63,5	0,9	0,8751
ARR1	264,0	47,7	155,7	138,2	0,6	0,1890
ARR2	18,4	12,7	2,9	3,4	0,2	0,0566
ARR3	20,8	23,6	18,0	17,4	0,9	0,8528
ART10	14,2	7,4	8,8	7,5	0,6	0,3418
ART5	4,3	3,2	3,6	5,6	0,8	0,8422
ARV1	14,9	3,5	15,0	18,2	1,0	0,9854
ARX1	289,2	137,3	158,4	110,8	0,5	0,1890
ASA1	42,1	24,4	19,1	16,2	0,5	0,1662
ASC1	4464,9	1462,1	3639,5	502,2	0,8	0,3267
ASE1	46,2	23,7	53,0	51,8	1,1	0,8205
ASF1	116,8	85,8	38,0	39,6	0,3	0,1465
ASF2	36,4	17,8	5,2	6,0	0,1	0,0160
ASG1	39,2	16,1	28,2	29,5	0,7	0,5361
ASG7	74,4	50,6	232,4	268,9	3,1	0,2921
ASH1	76,0	19,4	144,4	68,7	1,9	0,1039
ASI1	79,9	34,7	66,4	53,6	0,8	0,6858
ASI2	27,6	10,0	24,0	20,0	0,9	0,7592
ASI3	121,7	98,1	136,5	122,5	1,1	0,8561
ASK1	105,5	32,0	128,7	91,8	1,2	0,6506
ASK10	28,0	10,2	32,6	30,2	1,2	0,7830
ASM4	124,8	69,0	137,6	53,6	1,1	0,7795
ASN1	400,8	308,1	198,5	175,6	0,5	0,2974
ASN2	380,3	287,6	187,2	84,5	0,5	0,2450
ASP1	70,1	38,3	23,8	18,9	0,3	0,0732
ASP3-1	966,8	272,1	1684,2	1095,2	1,7	0,2506
ASR1	8,1	9,7	18,5	15,6	2,3	0,3018
AST2	5,1	1,2	5,8	6,8	1,1	0,8413
ATC1	34,3	34,0	53,1	61,8	1,5	0,6148
ATE1	11,4	8,2	22,3	17,9	2,0	0,3070
ATF1	17,4	4,8	8,8	7,5	0,5	0,1028
ATF2	10,3	13,8	23,1	23,9	2,2	0,3878
ATG1	13,1	7,5	8,8	10,4	0,7	0,5250
ATG10	21,1	9,2	26,1	19,7	1,2	0,6623
ATG11	19,1	7,3	20,0	21,0	1,0	0,9416
ATG12	21,8	15,9	24,3	21,1	1,1	0,8543
ATG13	55,3	39,2	44,8	31,0	0,8	0,6895
ATG14	85,9	85,3	90,1	72,7	1,0	0,9429
ATG15	0,0	0,0	2,0	4,0	#DIV/0!	0,3559
ATG17	25,3	19,0	18,2	13,6	0,7	0,5652
ATG18	13,2	7,9	29,0	23,4	2,2	0,2453

ATG19	49,2	26,0	40,5	27,1	0,8	0,6579
ATG2	137,8	212,5	36,5	25,5	0,3	0,3803
ATG20	6,9	5,9	3,1	3,7	0,4	0,3190
ATG21	13,2	10,6	15,3	16,1	1,2	0,8348
ATG22	25,2	33,2	14,4	9,6	0,6	0,5569
ATG23	26,9	11,2	34,9	29,0	1,3	0,6253
ATG26	17,2	9,9	12,9	8,9	0,8	0,5473
ATG27	63,4	49,2	50,2	46,0	0,8	0,7080
ATG3	7,2	7,6	9,9	10,6	1,4	0,6888
ATG32	52,4	17,5	26,2	20,4	0,5	0,0997
ATG33	22,7	14,2	41,2	31,3	1,8	0,3225
ATG34	3,0	2,1	2,4	4,7	0,8	0,8037
ATG4	0,0	0,0	0,0	0,0	#DIV/0!	#DIV/0!
ATG5	63,7	31,2	29,6	20,1	0,5	0,1155
ATG7	13,4	10,2	2,3	2,6	0,2	0,0780
ATG9	34,1	7,8	36,7	32,3	1,1	0,8799
ATH1	12,5	7,2	6,7	7,3	0,5	0,3008
ATM1	36,3	19,4	24,1	26,7	0,7	0,4870
ATO2	130,7	108,8	130,7	109,5	1,0	0,9999
ATO3	77,7	50,6	196,0	249,4	2,5	0,3884
ATP1	380,6	361,6	382,9	383,3	1,0	0,9934
ATP10	9,9	7,5	10,3	12,8	1,0	0,9639
ATP11	42,2	16,4	62,8	42,4	1,5	0,3982
ATP12	8,3	2,2	0,7	1,3	0,1	0,0010
ATP14	41,5	18,5	52,9	48,8	1,3	0,6783
ATP16	37,6	32,5	104,9	76,9	2,8	0,1581
ATP17	342,7	176,1	668,2	263,2	1,9	0,0856
ATP18	85,2	58,5	98,5	73,6	1,2	0,7869
ATP19	60,3	62,4	69,5	64,7	1,2	0,8441
ATP2	766,6	200,6	715,2	116,4	0,9	0,6730
ATP20	94,3	28,3	42,0	34,3	0,4	0,0569
ATP22	37,2	21,6	22,9	19,4	0,6	0,3644
ATP23	139,7	79,7	90,0	76,0	0,6	0,4010
ATP25	23,8	6,9	27,6	18,7	1,2	0,7139
ATP3	192,6	75,8	418,4	135,5	2,2	0,0270
ATP4	239,5	141,9	286,9	191,3	1,2	0,7045
ATP5	47,9	38,7	32,4	29,6	0,7	0,5466
ATP6	5,2	6,0	0,0	0,0	0,0	0,1340
ATR1	111,2	41,7	93,7	80,8	0,8	0,7131
ATS1	6,8	5,2	8,4	6,0	1,2	0,7000
ATX1	22,1	18,6	11,7	12,0	0,5	0,3818
ATX2	19,4	9,9	10,9	8,2	0,6	0,2300
AUA1	55,0	41,1	11,6	13,8	0,2	0,0922
AUR1	367,0	200,0	94,9	65,0	0,3	0,0414
AUS1	24,3	16,4	23,1	23,1	1,0	0,9363
AVL9	81,5	54,7	53,4	42,4	0,7	0,4476
AVO1	8,6	5,1	16,0	14,1	1,9	0,3651
AVO2	4,1	5,6	4,2	6,8	1,0	0,9814
AVT1	78,5	23,3	30,7	22,6	0,4	0,0258
AVT2	17,5	3,5	41,5	28,3	2,4	0,1439

AVT3	23,7	21,2	25,1	19,8	1,1	0,9280
AVT4	41,3	19,5	29,5	21,7	0,7	0,4471
AVT5	23,1	5,5	142,6	118,5	6,2	0,0905
AVT6	22,7	12,3	8,3	13,3	0,4	0,1609
AVT7	189,4	58,5	193,7	179,4	1,0	0,9653
AXL1	56,1	30,2	53,0	38,9	0,9	0,9031
AXL2	27,0	11,6	18,5	36,9	0,7	0,6741
AYR1	88,8	47,9	76,6	53,7	0,9	0,7465
AYT1	9,8	11,8	20,8	15,5	2,1	0,3032
AZF1	122,5	126,9	49,0	37,6	0,4	0,3087
AZR1	183,0	108,0	135,1	156,8	0,7	0,6333
BAG7	138,7	47,2	287,2	302,9	2,1	0,3700
BAP2	274,5	120,7	204,9	132,7	0,7	0,4675
BAP3	379,0	201,2	180,4	160,4	0,5	0,1735
BAR1	197,3	45,0	699,1	411,2	3,5	0,0514
BAS1	126,2	17,3	83,6	64,4	0,7	0,2488
BAT1	565,4	363,4	710,6	499,0	1,3	0,6545
BAT2	107,4	41,4	362,7	180,3	3,4	0,0328
BBC1	11,7	12,6	17,4	16,3	1,5	0,6015
BBP1	17,0	6,3	4,1	4,8	0,2	0,0171
BCD1	6,7	7,9	6,8	7,9	1,0	0,9981
BCH1	78,8	21,5	56,3	37,9	0,7	0,3423
BCH2	5,0	2,2	6,7	10,9	1,3	0,7660
BCK1	20,0	16,6	5,9	6,8	0,3	0,1682
BCK2	76,2	50,2	120,7	80,5	1,6	0,3846
BCP1	51,6	24,7	28,6	20,2	0,6	0,2006
BCS1	7,2	8,1	13,6	13,2	1,9	0,4417
BCY1	89,9	22,0	245,6	223,0	2,7	0,2142
BDF1	194,5	139,0	230,7	162,8	1,2	0,7469
BDH1	154,2	105,7	159,0	144,7	1,0	0,9590
BDH2	71,1	59,6	186,1	137,9	2,6	0,1766
BDP1	43,8	27,2	16,9	13,8	0,4	0,1279
BDS1	4,3	3,7	7,9	6,6	1,8	0,3859
BEM1	6,5	10,9	9,1	10,6	1,4	0,7474
BEM2	114,7	37,5	135,0	94,1	1,2	0,7032
BEM3	31,1	17,4	14,6	12,3	0,5	0,1702
BEM4	199,2	103,8	36,8	27,5	0,2	0,0233
BER1	302,2	77,4	141,0	101,8	0,5	0,0452
BET1	34,8	15,0	10,8	8,7	0,3	0,0327
BET2	41,1	14,1	13,9	12,6	0,3	0,0279
BET3	104,5	45,1	63,2	44,4	0,6	0,2387
BET4	6,3	9,8	0,0	0,0	0,0	0,2468
BET5	25,2	14,2	25,7	29,8	1,0	0,9753
BFA1	6,5	7,0	0,0	0,0	0,0	0,1156
BFR1	751,3	539,6	613,9	415,4	0,8	0,7006
BFR2	48,3	18,2	41,4	31,0	0,9	0,7130
BGL2	201,5	159,4	343,6	199,0	1,7	0,3076
BIG1	0,0	0,0	0,0	0,0	#DIV/0!	#DIV/0!
BIK1	15,0	8,0	71,0	118,4	4,7	0,3823
BIM1	30,7	32,3	74,1	125,7	2,4	0,5284

BIO2	17,0	16,0	25,3	31,0	1,5	0,6498
BIO3	12,4	7,9	3,0	5,9	0,2	0,1028
BIO4	9,1	7,0	33,2	33,2	3,6	0,2053
BIO5	233,3	52,2	379,7	106,8	1,6	0,0490
BIR1	25,1	15,0	176,5	251,0	7,0	0,2740
BIT2	44,4	45,1	66,3	52,3	1,5	0,5493
BIT61	6,6	4,0	3,2	4,8	0,5	0,3132
BLI1	112,1	32,6	65,7	45,9	0,6	0,1501
BLM10	44,5	16,5	99,2	118,4	2,2	0,3951
BLS1	20,2	12,6	29,9	23,3	1,5	0,4943
BMH1	3191,8	616,8	3339,0	760,9	1,0	0,7739
BMH2	647,6	519,2	833,1	297,3	1,3	0,5580
BMS1	333,7	73,3	170,1	119,9	0,5	0,0587
BNA1	178,6	66,1	218,0	164,5	1,2	0,6724
BNA2	2,3	4,6	5,5	6,4	2,4	0,4456
BNA3	87,0	15,8	94,5	63,1	1,1	0,8255
BNA4	14,3	12,0	3,0	3,6	0,2	0,1203
BNA5	24,2	15,6	13,8	12,6	0,6	0,3402
BNA6	109,5	106,5	114,0	122,7	1,0	0,9576
BNA7	63,7	44,0	14,5	18,3	0,2	0,0845
BNI1	68,1	42,3	60,1	40,4	0,9	0,7937
BNI4	31,9	25,6	14,6	10,4	0,5	0,2565
BNI5	64,5	50,4	20,7	24,1	0,3	0,1684
BNR1	27,7	10,8	9,4	8,4	0,3	0,0368
BNS1	20,4	21,4	31,4	29,2	1,5	0,5681
BOI1	38,6	13,6	82,2	102,2	2,1	0,4302
BOI2	31,3	22,9	20,9	19,6	0,7	0,5134
BOP2	32,6	28,3	11,3	11,2	0,3	0,2115
BOP3	22,1	8,8	56,4	37,7	2,5	0,1274
BOR1	7,9	7,3	23,0	19,2	2,9	0,1914
BOS1	3,5	2,8	4,2	5,6	1,2	0,8236
BPH1	32,0	15,6	42,2	50,1	1,3	0,7114
BPL1	24,9	27,9	89,7	143,2	3,6	0,4090
BPT1	74,3	18,6	98,7	41,3	1,3	0,3227
BRE1	169,9	55,5	199,2	80,1	1,2	0,5693
BRE2	43,2	34,3	34,4	25,6	0,8	0,6928
BRE4	7,5	4,2	2,3	2,6	0,3	0,0765
BRE5	796,5	706,5	948,9	823,9	1,2	0,7882
BRF1	210,1	168,3	270,0	123,2	1,3	0,5869
BRL1	15,1	16,1	116,2	226,2	7,7	0,4072
BRN1	10,8	5,4	10,7	8,3	1,0	0,9857
BRO1	67,9	21,2	20,3	13,9	0,3	0,0095
BRP1	14,3	7,0	38,9	34,2	2,7	0,2081
BRR1	39,0	6,4	36,7	27,3	0,9	0,8734
BRR2	42,7	11,7	105,9	66,5	2,5	0,1107
BRR6	31,1	20,2	9,2	8,6	0,3	0,0936
BRX1	71,3	62,5	52,7	57,8	0,7	0,6768
BSC1	0,0	0,0	0,7	1,3	#DIV/0!	0,3559
BSC2	100,2	56,1	40,3	30,5	0,4	0,1095
BSC4	1,1	2,1	3,7	4,6	3,5	0,3405

BSC5	1,8	2,8	8,8	6,0	5,0	0,0781
BSC6	54,7	31,7	31,1	27,5	0,6	0,3020
BSD2	92,1	19,1	46,6	34,2	0,5	0,0590
BSP1	583,2	165,4	362,9	89,3	0,6	0,0576
BST1	4989,4	2768,8	2069,1	1294,2	0,4	0,1046
BTN2	107,7	78,4	427,8	148,2	4,0	0,0088
BTS1	4,2	3,4	1,3	2,5	0,3	0,2158
BTT1	105,7	53,2	47,5	32,3	0,4	0,1107
BUB1	13,6	5,9	3,8	5,0	0,3	0,0440
BUB2	24,6	15,2	11,3	9,4	0,5	0,1863
BUB3	115,2	65,9	40,9	50,7	0,4	0,1242
BUD13	57,2	21,9	18,2	12,9	0,3	0,0220
BUD14	55,8	27,7	52,9	48,0	0,9	0,9208
BUD16	8,7	10,2	6,0	7,9	0,7	0,6913
BUD17	41,4	21,2	49,9	39,9	1,2	0,7190
BUD19	45,6	31,4	5,6	5,0	0,1	0,0457
BUD2	26,4	19,8	20,1	14,6	0,8	0,6296
BUD20	53,3	38,7	155,9	220,1	2,9	0,3938
BUD21	118,1	76,8	152,2	120,4	1,3	0,6508
BUD22	386,4	386,4	681,5	531,6	1,8	0,4038
BUD23	151,1	71,6	124,3	91,7	0,8	0,6617
BUD26	26,1	22,6	21,4	17,6	0,8	0,7529
BUD27	46,9	36,5	51,5	44,2	1,1	0,8780
BUD3	20,7	13,6	15,2	15,6	0,7	0,6192
BUD31	3,2	4,0	4,9	5,9	1,5	0,6549
BUD32	37,1	23,5	38,6	40,3	1,0	0,9520
BUD4	35,5	13,0	23,3	19,7	0,7	0,3394
BUD5	7,7	7,7	16,5	16,2	2,2	0,3629
BUD6	0,9	1,8	1,8	3,6	2,0	0,6679
BUD7	17,1	10,2	20,9	15,2	1,2	0,6965
BUD8	31,8	22,8	25,1	21,9	0,8	0,6839
BUD9	194,6	21,2	124,4	110,1	0,6	0,2568
BUG1	145,2	91,8	206,2	156,7	1,4	0,5272
BUL1	12,0	10,5	13,7	14,3	1,1	0,8503
BUL2	57,5	27,4	32,2	22,7	0,6	0,2039
BUR2	14,1	3,9	17,2	15,8	1,2	0,7112
BUR6	181,5	143,3	301,1	331,1	1,7	0,5319
BYE1	63,9	11,3	26,5	19,0	0,4	0,0147
BZZ1	8,1	6,4	9,9	9,4	1,2	0,7653
CAB1	58,2	56,4	41,9	35,9	0,7	0,6421
CAB2	89,9	17,1	354,4	435,4	3,9	0,2702
CAB3	18,5	9,2	23,1	21,2	1,2	0,7039
CAB4	17,1	9,9	15,1	11,4	0,9	0,7954
CAC2	21,9	21,1	11,3	14,8	0,5	0,4404
CAD1	15,9	8,7	21,3	17,1	1,3	0,5902
CAF120	18,6	8,9	88,3	106,9	4,8	0,2415
CAF130	27,5	15,8	23,0	15,7	0,8	0,7010
CAF16	52,3	31,5	77,5	72,0	1,5	0,5446
CAF4	25,4	13,8	24,5	25,7	1,0	0,9533
CAF40	112,6	18,4	238,0	283,9	2,1	0,4122

CAJ1	262,0	155,0	300,4	213,8	1,1	0,7810
CAK1	39,3	8,0	148,8	104,3	3,8	0,0811
CAM1	143,5	88,8	141,3	138,0	1,0	0,9789
CAN1	143,3	63,5	37,0	27,8	0,3	0,0220
CAP1	18,6	13,5	12,7	9,3	0,7	0,5039
CAP2	277,7	107,8	199,0	41,6	0,7	0,2221
CAR1	51,0	32,7	77,8	54,8	1,5	0,4341
CAR2	0,0	0,0	1,2	2,4	#DIV/0!	0,3559
CAT2	19,7	10,0	25,8	20,5	1,3	0,6135
CAT5	96,8	20,3	79,0	53,9	0,8	0,5606
CAT8	235,5	81,3	77,1	66,6	0,3	0,0236
CAX4	26,6	30,6	12,9	11,7	0,5	0,4363
CBC2	7,0	2,9	9,9	13,2	1,4	0,6813
CBF1	40,6	29,2	66,6	64,8	1,6	0,4922
CBF2	45,1	15,1	64,5	43,4	1,4	0,4314
CBF5	963,3	797,9	884,6	676,7	0,9	0,8854
CBK1	35,0	18,6	20,9	21,1	0,6	0,3526
CBP1	118,2	48,2	27,0	21,7	0,2	0,0136
CBP2	14,4	12,6	4,2	5,0	0,3	0,1812
CBP3	14,4	17,2	11,8	13,0	0,8	0,8119
CBP4	4,7	3,4	23,8	20,4	5,1	0,1150
CBR1	72,0	40,8	147,6	91,5	2,0	0,1821
CBS2	6,3	5,4	10,8	10,7	1,7	0,4786
CBT1	4,1	6,0	9,8	6,6	2,4	0,2523
CCA1	37,2	13,0	18,0	13,6	0,5	0,0879
CCC1	208,2	110,7	200,0	81,9	1,0	0,9088
CCC2	25,9	16,9	12,1	21,1	0,5	0,3471
CCH1	84,4	18,7	36,3	24,2	0,4	0,0200
CCL1	108,1	37,1	53,9	41,1	0,5	0,0978
CCM1	11,7	15,0	15,6	19,2	1,3	0,7607
CCP1	121,6	105,7	109,1	114,0	0,9	0,8776
CCR4	22,9	13,3	44,5	31,7	1,9	0,2564
CCS1	314,8	83,5	313,7	125,6	1,0	0,9889
CCT2	92,8	50,5	105,2	91,3	1,1	0,8209
CCT3	51,4	17,9	26,5	18,1	0,5	0,0983
CCT4	128,6	66,6	104,3	74,2	0,8	0,6432
CCT5	132,8	85,6	88,5	77,8	0,7	0,4733
CCT6	91,3	46,6	101,2	71,1	1,1	0,8220
CCT7	107,4	59,7	74,5	67,6	0,7	0,4944
CCT8	8,0	2,6	9,6	10,0	1,2	0,7557
CCW14	92,4	87,5	76,8	80,7	0,8	0,8015
CCZ1	0,0	0,0	0,0	0,0	#DIV/0!	#DIV/0!
CDA1	1,2	2,4	0,7	1,3	0,6	0,7150
CDA2	3,9	5,8	0,0	0,0	0,0	0,2210
CDC1	19,6	13,5	17,3	13,7	0,9	0,8182
CDC10	17,0	16,7	16,4	19,4	1,0	0,9665
CDC11	39,7	28,6	23,5	20,4	0,6	0,3926
CDC12	19,7	14,6	15,3	16,5	0,8	0,6994
CDC123	18,4	6,8	21,9	18,4	1,2	0,7298
CDC13	12,4	11,7	21,7	19,5	1,7	0,4475

CDC14	33,4	29,0	13,0	17,9	0,4	0,2778
CDC15	595,1	294,1	1408,1	862,4	2,4	0,1246
CDC16	330,8	82,7	190,5	141,2	0,6	0,1371
CDC19	0,0	0,0	0,0	0,0	#DIV/0!	#DIV/0!
CDC20	14,7	5,8	27,7	30,6	1,9	0,4358
CDC21	36,6	23,7	0,0	0,0	0,0	0,0216
CDC23	14,5	10,5	10,6	12,2	0,7	0,6480
CDC24	18,0	14,3	14,2	12,3	0,8	0,7043
CDC25	35,7	21,0	28,9	24,9	0,8	0,6930
CDC26	37,0	15,7	17,8	12,2	0,5	0,1021
CDC27	42,7	19,0	27,6	20,1	0,6	0,3160
CDC28	19,7	14,8	90,1	142,5	4,6	0,3634
CDC3	137,9	96,0	206,2	108,1	1,5	0,3816
CDC31	26,2	23,2	22,9	28,6	0,9	0,8631
CDC33	473,6	311,0	195,0	191,7	0,4	0,1780
CDC34	231,2	114,8	215,5	66,4	0,9	0,8208
CDC36	22,9	15,6	14,6	10,4	0,6	0,4129
CDC37	261,6	311,4	299,0	381,4	1,1	0,8842
CDC39	97,5	55,0	86,9	58,4	0,9	0,7995
CDC4	42,3	25,9	32,1	27,6	0,8	0,6078
CDC40	274,8	163,6	90,8	125,9	0,3	0,1250
CDC43	10,0	5,8	5,0	5,5	0,5	0,2558
CDC45	20,2	18,2	1,3	2,6	0,1	0,0857
CDC48	112,6	50,2	267,1	236,3	2,4	0,2482
CDC5	92,6	36,8	36,2	28,8	0,4	0,0521
CDC50	6,3	4,8	8,0	9,3	1,3	0,7653
CDC53	45,3	27,8	66,7	56,8	1,5	0,5230
CDC55	120,5	69,5	121,7	92,3	1,0	0,9836
CDC6	25,4	8,1	10,8	8,6	0,4	0,0483
CDC60	247,7	90,9	280,0	176,1	1,1	0,7557
CDC7	16,2	5,8	9,1	6,5	0,6	0,1541
CDC73	1,9	2,2	9,2	9,1	4,9	0,1687
CDC8	22,8	16,8	12,8	14,8	0,6	0,4087
CDC9	5,3	4,7	1,6	3,3	0,3	0,2521
CDD1	6,5	3,7	11,0	7,7	1,7	0,3388
CDH1	15,6	9,0	17,6	11,8	1,1	0,7942
CDS1	34,1	16,9	109,1	115,9	3,2	0,2478
CEF1	54,4	34,6	74,0	56,4	1,4	0,5765
CEG1	12,1	7,1	8,2	10,6	0,7	0,5643
CEM1	3,5	3,4	5,0	4,1	1,4	0,5843
CEP3	79,8	25,5	182,0	101,5	2,3	0,0986
CET1	8,6	6,7	3,6	4,6	0,4	0,2718
CEX1	11,9	8,1	4,4	4,3	0,4	0,1522
CFD1	38,6	25,2	54,3	39,5	1,4	0,5257
CFT1	59,3	22,5	74,9	51,3	1,3	0,5982
CFT2	25,7	27,6	22,5	21,0	0,9	0,8580
CGI121	5,7	6,9	3,0	5,9	0,5	0,5643
CGR1	308,7	182,5	389,6	100,3	1,3	0,4666
CHA1	15,6	14,1	9,7	10,8	0,6	0,5357
CHA4	24,2	16,5	21,8	16,6	0,9	0,8462

CHC1	47,3	32,1	38,6	32,0	0,8	0,7149
CHD1	113,1	26,5	168,2	48,4	1,5	0,0931
CHK1	3,6	4,7	9,3	13,1	2,6	0,4434
CHL1	32,0	10,7	20,0	13,5	0,6	0,2110
CHL4	143,1	49,0	313,3	208,4	2,2	0,1629
CHO1	585,6	219,4	452,4	389,5	0,8	0,5731
CHO2	215,5	160,1	125,4	164,9	0,6	0,4625
CHS1	100,4	62,6	174,3	77,1	1,7	0,1876
CHS2	44,8	22,0	50,9	48,4	1,1	0,8253
CHS3	45,7	26,5	91,4	124,4	2,0	0,4996
CHS5	767,5	141,0	868,2	409,3	1,1	0,6581
CHS6	14,6	13,9	37,3	25,0	2,6	0,1638
CHS7	193,6	113,3	241,5	188,5	1,2	0,6788
CHZ1	0,0	0,0	1,2	2,4	#DIV/0!	0,3559
CIA1	131,5	42,4	128,9	35,7	1,0	0,9266
CIC1	40,4	30,4	26,9	23,7	0,7	0,5116
CIK1	13,5	9,4	15,6	13,9	1,2	0,8144
CIN1	67,8	18,6	13,0	17,0	0,2	0,0048
CIN2	234,5	147,5	404,3	263,2	1,7	0,3036
CIN5	29,6	9,5	50,9	34,6	1,7	0,2793
CIN8	41,4	27,6	11,0	14,6	0,3	0,0990
CIS1	14,0	12,4	16,9	11,9	1,2	0,7498
CIS3	2921,1	1742,1	422,1	164,6	0,1	0,0289
CIT1	1045,3	610,8	2010,6	1266,3	1,9	0,2188
CIT2	1366,1	1019,3	1676,8	636,1	1,2	0,6235
CIT3	72,9	38,2	222,6	277,7	3,1	0,3265
CKA1	236,0	143,4	238,5	160,0	1,0	0,9823
CKA2	22,3	25,7	19,9	19,6	0,9	0,8902
CKB1	39,6	17,7	42,3	38,3	1,1	0,9018
CKB2	45,6	13,1	32,2	24,4	0,7	0,3722
CKI1	18,5	12,0	29,9	25,0	1,6	0,4408
CKS1	45,1	34,1	18,7	13,4	0,4	0,1995
CLA4	16,6	15,1	43,5	38,5	2,6	0,2424
CLB1	82,1	43,9	25,7	22,4	0,3	0,0619
CLB2	48,8	27,6	40,3	37,5	0,8	0,7270
CLB3	64,3	27,5	59,6	47,0	0,9	0,8683
CLB4	19,2	13,0	57,8	108,8	3,0	0,5080
CLB5	52,7	34,7	13,1	14,0	0,2	0,0785
CLB6	29,5	11,1	0,7	1,3	0,0	0,0021
CLD1	50,8	21,0	9,7	10,3	0,2	0,0124
CLF1	27,5	11,7	26,6	24,0	1,0	0,9467
CLG1	1239,0	662,5	2167,8	1277,1	1,7	0,2442
CLN1	59,7	25,8	27,3	29,9	0,5	0,1514
CLN2	262,1	127,6	15,9	11,1	0,1	0,0085
CLN3	87,9	42,0	77,6	57,0	0,9	0,7802
CLP1	5,4	3,9	2,4	3,4	0,4	0,2856
CLU1	271,4	141,7	161,3	129,3	0,6	0,2947
CMC4	11,9	8,8	22,4	20,3	1,9	0,3760
CMK1	9,4	7,4	7,1	6,9	0,7	0,6575
CMK2	10,0	7,7	10,3	8,3	1,0	0,9632

CMP2	22,9	0,9	14,7	14,2	0,6	0,2917
CMS1	46,9	19,8	32,9	22,4	0,7	0,3861
CNA1	54,4	34,3	25,2	21,1	0,5	0,1974
CNB1	65,6	33,4	74,7	59,4	1,1	0,7966
CNE1	2,8	4,8	7,7	6,8	2,8	0,2827
CNL1	22,9	6,6	4,9	5,9	0,2	0,0065
CNM67	59,5	29,8	34,1	25,0	0,6	0,2397
CNN1	0,0	0,0	1,9	3,8	#DIV/0!	0,3559
CNS1	60,2	28,0	72,0	52,1	1,2	0,7031
COA1	19,1	13,1	13,3	11,8	0,7	0,5352
COA2	33,0	4,8	39,1	32,2	1,2	0,7181
COA3	176,3	164,9	52,8	45,3	0,3	0,1989
COA4	24,3	7,7	65,3	39,9	2,7	0,0906
COB	21,2	23,8	3,6	7,3	0,2	0,2069
COF1	292,6	147,2	319,8	275,9	1,1	0,8675
COG1	76,0	33,4	98,1	65,4	1,3	0,5690
COG2	82,9	26,0	127,2	91,0	1,5	0,3855
COG3	53,3	38,0	30,0	20,3	0,6	0,3207
COG4	13,4	8,8	5,6	5,0	0,4	0,1705
COG5	16,4	12,8	29,0	25,2	1,8	0,4064
COG6	71,6	12,8	48,8	39,2	0,7	0,3119
COG7	74,5	26,3	62,7	53,0	0,8	0,7020
COG8	157,2	88,9	119,2	83,1	0,8	0,5551
COP1	169,4	40,9	105,1	72,0	0,6	0,1714
COQ1	18,3	7,0	81,9	94,2	4,5	0,2270
COQ10	231,5	37,1	20,8	15,5	0,1	0,0000
COQ2	6,8	4,3	4,2	5,0	0,6	0,4669
COQ3	61,9	14,4	93,8	89,9	1,5	0,5096
COQ4	11,9	10,7	14,2	16,0	1,2	0,8236
COQ5	578,1	164,4	1287,4	722,2	2,2	0,1039
COQ6	47,9	23,9	192,7	276,4	4,0	0,3368
COQ8	7,5	7,5	7,4	7,1	1,0	0,9872
COQ9	63,0	18,4	47,7	42,3	0,8	0,5310
COR1	194,7	142,1	241,3	198,1	1,2	0,7158
COS1	0,0	0,0	0,0	0,0	#DIV/0!	#DIV/0!
COS10	11,6	12,2	0,7	1,3	0,1	0,1247
COS111	318,3	212,9	209,2	150,0	0,7	0,4341
COS12	2,0	2,4	0,7	1,3	0,3	0,3485
COS2	76,3	46,1	88,2	61,2	1,2	0,7663
COS4	126,4	84,1	22,4	17,4	0,2	0,0518
COS6	16,9	7,4	2,6	3,0	0,2	0,0114
COS7	30,9	16,7	15,2	11,2	0,5	0,1699
COS9	20,3	12,6	73,4	90,1	3,6	0,2872
COT1	13,8	7,3	5,6	7,2	0,4	0,1604
COX1	109,8	54,7	79,9	94,3	0,7	0,6023
COX10	18,4	23,2	7,9	8,5	0,4	0,4294
COX11	94,4	50,5	58,7	50,6	0,6	0,3568
COX12	194,5	71,8	309,3	230,9	1,6	0,3793
COX13	118,7	68,6	226,5	97,7	1,9	0,1212
COX14	86,2	73,1	110,2	97,3	1,3	0,7072

COX15	61,0	21,7	26,9	19,7	0,4	0,0588
COX17	95,3	55,1	235,4	98,1	2,5	0,0472
COX18	41,5	21,2	14,2	10,1	0,3	0,0591
COX19	14,2	15,5	34,0	34,4	2,4	0,3334
COX2	7,2	14,3	1,3	2,6	0,2	0,4529
COX23	0,0	0,0	0,0	0,0	#DIV/0!	#DIV/0!
COX3	15,5	27,2	1,0	2,0	0,1	0,3286
COX4	197,7	116,9	313,8	162,9	1,6	0,2905
COX5A	63,2	25,3	125,4	34,6	2,0	0,0272
COX5B	118,8	52,4	182,9	34,9	1,5	0,0880
COX7	1,7	3,4	0,0	0,0	0,0	0,3559
COX8	38,3	18,1	129,7	152,9	3,4	0,2798
COX9	124,4	64,3	214,1	130,0	1,7	0,2621
COY1	22,5	4,3	19,4	13,1	0,9	0,6741
CPA1	513,4	157,2	307,0	40,6	0,6	0,0438
CPA2	296,6	138,7	183,6	123,5	0,6	0,2696
CPD1	10,6	5,4	7,8	6,3	0,7	0,5307
CPR1	458,6	437,2	906,4	1021,0	2,0	0,4508
CPR2	47,9	36,9	69,8	68,1	1,5	0,5911
CPR3	140,2	53,8	136,4	114,6	1,0	0,9535
CPR4	28,6	19,7	111,6	174,1	3,9	0,3799
CPR5	540,1	331,9	1374,0	1258,8	2,5	0,2474
CPR6	13,9	8,6	54,3	41,6	3,9	0,1057
CPR7	36,2	23,4	29,9	21,9	0,8	0,7069
CPR8	27,1	10,4	21,9	21,5	0,8	0,6787
CPS1	4,4	5,7	6,2	5,0	1,4	0,6571
CPT1	111,2	60,3	50,7	45,4	0,5	0,1600
CRC1	2,0	2,4	1,2	2,4	0,6	0,6368
CRD1	2,2	1,6	3,9	5,6	1,8	0,5669
CRF1	47,7	27,0	41,1	38,9	0,9	0,7891
CRH1	60,7	37,2	23,0	16,8	0,4	0,1140
CRM1	16,4	11,2	80,1	130,7	4,9	0,3693
CRN1	236,8	130,5	87,0	58,2	0,4	0,0809
CRP1	437,0	327,9	332,2	226,7	0,8	0,6181
CRR1	14,7	15,6	13,1	12,6	0,9	0,8789
CRS5	163,7	51,2	219,0	188,0	1,3	0,5909
CRT10	22,6	11,2	8,9	7,6	0,4	0,0895
CRZ1	73,9	25,7	77,8	74,8	1,1	0,9253
CSE1	322,2	148,3	158,4	113,6	0,5	0,1301
CSE2	3,7	3,4	1,3	2,6	0,4	0,3080
CSE4	41,8	23,4	26,8	25,9	0,6	0,4237
CSF1	83,5	16,1	20,0	15,1	0,2	0,0012
CSG2	41,4	38,8	35,0	31,8	0,8	0,8082
CSH1	4281,6	2193,1	621,8	443,8	0,1	0,0170
CSI1	77,1	15,6	52,3	74,5	0,7	0,5397
CSI2	37,4	13,8	17,4	18,7	0,5	0,1354
CSL4	27,7	10,0	16,6	15,8	0,6	0,2793
CSM2	18,1	4,9	11,6	13,8	0,6	0,4118
CSM3	4,8	5,7	2,4	2,8	0,5	0,4874
CSM4	2,5	3,0	0,0	0,0	0,0	0,1359

CSN12	37,9	24,5	51,3	42,4	1,4	0,6044
CSN9	38,5	29,9	14,7	15,2	0,4	0,2049
CSR1	29,3	20,9	8,5	10,4	0,3	0,1249
CSR2	80,7	49,8	125,7	100,5	1,6	0,4525
CST26	15,5	11,7	36,2	30,2	2,3	0,2501
CST6	1043,2	772,0	639,1	564,5	0,6	0,4304
CST9	4,2	4,9	6,9	4,9	1,7	0,4565
CTA1	273,2	153,3	125,5	72,0	0,5	0,1316
CTF13	75,0	53,7	87,8	96,2	1,2	0,8233
CTF18	61,3	27,4	140,0	146,8	2,3	0,3323
CTF19	38,4	48,1	31,4	54,8	0,8	0,8537
CTF3	41,9	29,1	7,5	5,0	0,2	0,0592
CTF4	64,5	15,6	166,5	211,8	2,6	0,3737
CTH1	22,1	14,0	17,8	16,5	0,8	0,7047
CTI6	71,9	12,9	27,5	19,9	0,4	0,0096
CTK1	10,9	2,5	8,2	5,6	0,8	0,4111
CTK2	20,3	14,1	7,4	6,9	0,4	0,1523
CTK3	150,3	80,2	119,2	133,0	0,8	0,7030
CTL1	21,3	11,1	15,9	15,5	0,7	0,5962
CTM1	51,5	22,9	33,3	33,1	0,6	0,4001
CTP1	85,0	57,6	107,8	124,9	1,3	0,7518
CTR1	211,4	107,1	227,4	75,3	1,1	0,8153
CTR2	29,7	19,6	6,8	7,9	0,2	0,0733
CTR3	36,3	16,0	95,1	63,7	2,6	0,1232
CTR86	6,5	3,5	80,4	139,7	12,3	0,3309
CTR9	63,5	34,0	75,7	54,4	1,2	0,7176
CTS1	7,8	4,7	10,3	9,6	1,3	0,6617
CTS2	7,0	4,3	2,4	4,7	0,3	0,2019
CTT1	3,0	5,9	0,0	0,0	0,0	0,3559
CUE1	158,1	93,1	98,1	82,2	0,6	0,3712
CUE2	51,2	17,1	72,8	74,6	1,4	0,5926
CUE3	44,1	22,5	33,1	30,6	0,8	0,5862
CUE4	17,1	17,7	3,0	3,6	0,2	0,1706
CUE5	66,0	29,7	81,9	56,9	1,2	0,6380
CUL3	12,2	10,4	21,4	15,4	1,8	0,3590
CUP2	0,0	0,0	0,0	0,0	#DIV/0!	#DIV/0!
CUP9	37,9	22,1	33,0	29,2	0,9	0,7971
CUR1	13,6	11,8	9,7	11,6	0,7	0,6497
CUS1	77,9	66,2	69,0	63,0	0,9	0,8525
CUS2	15,8	2,9	19,6	15,3	1,2	0,6431
CWC15	22,5	17,3	11,5	10,2	0,5	0,3142
CWC2	39,1	32,8	48,2	39,9	1,2	0,7376
CWC21	35,1	13,4	137,0	212,4	3,9	0,3751
CWC22	1678,7	573,2	338,5	53,2	0,2	0,0035
CWC24	4,1	3,0	1,9	2,4	0,5	0,2989
CWC25	366,7	335,4	75,6	83,8	0,2	0,1432
CWC27	69,1	40,1	79,0	57,2	1,1	0,7863
CWH41	72,7	19,9	31,3	23,0	0,4	0,0346
CWH43	83,8	63,7	70,9	60,7	0,8	0,7785
CWP1	193,3	35,6	157,5	165,1	0,8	0,6860

CYB2	27,7	19,5	26,5	23,7	1,0	0,9401
CYB5	16,2	9,4	3,1	3,7	0,2	0,0409
CYC1	232,2	187,1	57,9	55,6	0,2	0,1244
CYC2	11,8	9,3	7,9	9,2	0,7	0,5724
CYC3	56,4	21,3	98,0	78,2	1,7	0,3441
CYC7	3,4	4,6	5,9	4,9	1,7	0,4938
CYC8	39,5	24,4	36,9	36,9	0,9	0,9121
CYK3	17,8	9,4	19,1	22,2	1,1	0,9165
CYM1	18,5	12,1	41,2	51,3	2,2	0,4219
CYR1	24,5	13,9	42,8	34,1	1,7	0,3580
CYS3	26,7	25,5	61,3	66,9	2,3	0,3714
CYS4	15,6	9,8	18,5	17,2	1,2	0,7807
CYT1	181,7	62,5	124,5	114,9	0,7	0,4148
CYT2	39,4	33,8	24,8	19,4	0,6	0,4797
DAD3	15,0	27,1	1,3	2,6	0,1	0,3531
DAK1	46,2	28,9	48,5	36,0	1,1	0,9234
DAK2	1,1	2,1	26,8	18,7	25,1	0,0341
DAL1	5,2	4,2	72,4	108,7	13,9	0,2631
DAL2	18,5	11,0	21,4	17,5	1,2	0,7871
DAL3	9,1	3,8	3,8	2,5	0,4	0,0585
DAL4	50,4	44,9	279,4	421,4	5,5	0,3213
DAL5	8,3	4,5	11,0	7,3	1,3	0,5505
DAL7	8,9	8,2	1,2	2,4	0,1	0,1221
DAL80	2,2	2,6	1,2	2,4	0,5	0,5686
DAL81	9,0	6,1	73,1	107,7	8,1	0,2797
DAL82	6,4	5,7	3,0	5,9	0,5	0,4381
DAM1	126,2	67,7	82,1	55,9	0,7	0,3535
DAN1	72,6	29,5	29,5	43,3	0,4	0,1510
DAN4	15,0	8,6	11,7	10,1	0,8	0,6383
DAP1	30,7	25,7	39,8	34,5	1,3	0,6867
DAP2	57,7	19,6	51,4	34,3	0,9	0,7603
DAS1	8,0	3,3	8,5	10,0	1,1	0,9327
DAS2	98,9	41,5	119,8	68,3	1,2	0,6191
DAT1	31,0	16,4	28,8	31,8	0,9	0,9058
DBF2	64,4	49,4	137,6	112,6	2,1	0,2786
DBF20	2,3	3,2	2,4	2,8	1,1	0,9421
DBF4	99,1	91,9	40,2	28,3	0,4	0,2663
DBP1	8,7	7,2	5,7	5,2	0,6	0,5159
DBP10	107,8	48,8	136,5	121,1	1,3	0,6758
DBP2	230,7	58,6	214,5	49,7	0,9	0,6886
DBP3	156,8	99,7	179,9	163,1	1,1	0,8171
DBP5	98,7	46,3	132,2	52,5	1,3	0,3759
DBP6	11,5	14,4	8,7	7,3	0,8	0,7390
DBP7	23,2	19,7	20,2	19,7	0,9	0,8404
DBP8	12,7	8,4	3,2	6,3	0,2	0,1191
DBP9	142,4	103,0	115,8	109,3	0,8	0,7354
DBR1	30,6	30,2	21,9	19,4	0,7	0,6424
DCC1	3,8	5,3	1,8	3,6	0,5	0,5468
DCD1	24,9	8,7	10,8	9,7	0,4	0,0728
DCG1	4,0	7,9	4,9	5,7	1,2	0,8545

DCN1	10,4	5,5	15,0	16,5	1,4	0,6142
DCP1	81,5	47,8	27,1	19,1	0,3	0,0789
DCP2	49,9	20,1	52,0	45,8	1,0	0,9349
DCR2	28,4	19,6	5,3	10,6	0,2	0,0836
DCS1	242,9	30,9	936,7	486,5	3,9	0,0293
DCS2	6,8	6,0	110,9	128,6	16,4	0,1571
DCW1	222,8	123,5	265,0	180,5	1,2	0,7127
DDC1	14,0	9,8	20,7	17,8	1,5	0,5390
DDI1	20,8	13,3	15,2	11,0	0,7	0,5423
DDI2	9,9	8,9	8,5	5,8	0,9	0,7970
DDP1	13,4	11,2	19,6	22,7	1,5	0,6426
DDR2	30,0	12,6	56,3	46,2	1,9	0,3145
DED1	47,6	44,5	18,9	17,4	0,4	0,2742
DED81	363,6	209,9	356,9	338,5	1,0	0,9744
DEF1	646,1	517,6	328,9	293,6	0,5	0,3275
DEG1	22,1	12,3	14,1	12,5	0,6	0,3958
DEP1	26,0	19,0	27,3	32,5	1,1	0,9457
DER1	21,0	13,4	18,7	15,4	0,9	0,8246
DET1	27,7	21,7	14,6	17,6	0,5	0,3833
DFG10	26,5	9,8	38,9	26,1	1,5	0,4077
DFG16	16,9	26,6	72,6	117,4	4,3	0,3908
DFG5	152,1	94,0	200,7	185,2	1,3	0,6557
DFM1	54,4	44,6	131,5	74,4	2,4	0,1256
DFR1	15,1	12,5	12,8	13,7	0,8	0,8138
DGA1	48,2	21,3	46,9	24,3	1,0	0,9387
DGK1	27,7	19,2	16,7	16,8	0,6	0,4217
DGR1	25,7	19,5	29,5	44,9	1,1	0,8806
DGR2	43,1	26,4	39,0	26,2	0,9	0,8303
DHH1	11,0	9,5	20,8	21,3	1,9	0,4328
DHR2	40,5	40,4	172,0	216,9	4,2	0,2783
DIA1	17,9	13,1	15,0	10,3	0,8	0,7335
DIA2	104,5	5,3	26,7	21,4	0,3	0,0004
DIA3	12,3	5,4	11,9	8,3	1,0	0,9432
DIA4	24,7	8,7	16,3	14,8	0,7	0,3647
DIB1	38,8	5,7	58,6	41,1	1,5	0,3760
DIC1	77,8	38,3	117,7	136,0	1,5	0,5933
DID4	53,3	37,9	71,8	70,2	1,3	0,6608
DIE2	22,2	11,4	23,7	25,4	1,1	0,9180
DIG1	24,2	19,0	71,3	99,9	3,0	0,3896
DIG2	54,1	46,4	16,2	23,0	0,3	0,1940
DIM1	223,0	126,9	255,1	170,7	1,1	0,7731
DIN7	27,2	11,8	13,1	14,5	0,5	0,1799
DIP2	51,1	35,7	90,0	16,3	1,8	0,0947
DIP5	672,9	283,4	185,3	126,1	0,3	0,0200
DIS3	24,0	15,0	12,9	11,8	0,5	0,2891
DIT1	2,1	2,9	10,6	15,6	5,0	0,3241
DIT2	2,5	2,9	1,9	2,4	0,8	0,7704
DJP1	89,4	35,1	63,2	46,7	0,7	0,4049
DLD1	363,6	89,7	388,8	216,1	1,1	0,8363
DLD2	32,5	23,5	20,5	19,3	0,6	0,4620

DLD3	290,7	203,8	495,2	155,3	1,7	0,1616
DLT1	26,6	19,6	25,9	25,9	1,0	0,9669
DMA1	56,0	15,0	17,7	13,6	0,3	0,0093
DMA2	46,6	14,7	42,5	32,1	0,9	0,8226
DMC1	55,0	36,2	148,6	118,5	2,7	0,1816
DML1	18,7	10,0	11,5	9,8	0,6	0,3421
DNA2	13,2	10,2	33,6	23,3	2,5	0,1592
DNF1	41,0	20,4	97,8	82,2	2,4	0,2285
DNF2	17,0	7,0	4,4	3,3	0,3	0,0178
DNF3	1002,5	606,6	362,4	290,8	0,4	0,1057
DNL4	61,8	33,5	232,2	206,3	3,8	0,1541
DNM1	21,2	18,8	9,9	8,1	0,5	0,3149
DOA1	13,4	12,8	29,8	32,5	2,2	0,3835
DOA4	17,8	9,7	19,3	14,1	1,1	0,8615
DOC1	24,7	23,6	10,8	9,8	0,4	0,3206
DOG1	18,8	9,5	42,8	33,7	2,3	0,2205
DOG2	28,1	10,8	120,4	67,5	4,3	0,0355
DOM34	29,6	24,4	20,1	17,1	0,7	0,5441
DON1	5,7	6,8	1,8	2,3	0,3	0,3261
DOP1	22,4	7,4	19,6	14,3	0,9	0,7377
DOS2	555,3	104,2	1107,9	898,6	2,0	0,2676
DOT1	717,5	265,4	539,3	411,9	0,8	0,4945
DOT5	15,8	14,0	12,9	18,7	0,8	0,8067
DOT6	278,6	120,1	331,5	221,6	1,2	0,6890
DPB11	28,2	14,5	9,8	10,2	0,3	0,0840
DPB2	16,6	4,9	3,8	2,5	0,2	0,0035
DPB4	69,7	30,0	30,1	30,4	0,4	0,1135
DPH1	283,0	76,4	153,8	137,5	0,5	0,1515
DPH2	17,4	17,4	9,7	12,4	0,6	0,4965
DPH5	24,1	2,3	77,0	96,2	3,2	0,3133
DPL1	48,6	18,7	95,1	40,9	2,0	0,0838
DPM1	98,2	49,2	118,2	116,1	1,2	0,7619
DPP1	256,4	66,9	109,3	78,9	0,4	0,0294
DPS1	249,7	136,1	499,5	234,1	2,0	0,1146
DRE2	29,4	17,8	55,7	39,8	1,9	0,2744
DRS1	103,0	56,0	133,2	101,5	1,3	0,6210
DRS2	61,8	42,8	58,0	47,8	0,9	0,9091
DSD1	22,6	7,2	8,2	9,2	0,4	0,0486
DSE1	25,6	9,0	25,2	20,7	1,0	0,9724
DSE2	1160,9	373,8	637,5	432,3	0,5	0,1168
DSE3	47,2	18,4	35,3	29,2	0,7	0,5163
DSE4	87,1	29,6	226,0	169,0	2,6	0,1567
DSF1	2,4	3,0	7,3	7,5	3,0	0,2679
DSF2	29,7	4,1	23,3	16,4	0,8	0,4826
DSK2	129,6	43,3	173,9	125,5	1,3	0,5294
DSL1	208,4	27,2	165,1	120,2	0,8	0,5085
DSS1	31,1	25,2	42,6	32,2	1,4	0,5928
DSS4	20,1	14,2	11,1	14,5	0,6	0,4123
DST1	8,2	5,7	7,8	11,2	1,0	0,9575
DTD1	47,2	18,0	6,0	5,2	0,1	0,0046

DTR1	20,0	14,2	10,5	16,3	0,5	0,4135
DUG1	24,2	15,8	29,9	25,7	1,2	0,7147
DUG2	11,6	10,6	2,6	3,6	0,2	0,1575
DUG3	10,3	9,3	12,1	12,7	1,2	0,8249
DUN1	10,7	8,5	1,9	2,4	0,2	0,0946
DUO1	40,1	26,6	41,4	33,4	1,0	0,9563
DUR1,2	5,6	4,3	15,8	20,8	2,8	0,3770
DUR3	24,4	15,5	61,1	106,5	2,5	0,5196
DUS1	9,6	3,8	2,6	3,0	0,3	0,0273
DUS3	4,1	4,2	7,5	5,0	1,8	0,3361
DUS4	235,0	263,1	44,8	53,7	0,2	0,2065
DUT1	0,0	0,0	0,0	0,0	#DIV/0!	#DIV/0!
DYN1	45,4	21,0	53,2	53,6	1,2	0,7946
DYN2	92,8	26,4	39,5	28,4	0,4	0,0335
DYS1	163,5	120,1	148,6	116,2	0,9	0,8639
EAF1	35,7	20,8	32,4	22,1	0,9	0,8347
EAF3	104,6	45,3	162,1	84,4	1,6	0,2748
EAF5	23317,7	10562,2	34457,1	20559,3	1,5	0,3723
EAF6	77,8	32,5	200,7	87,2	2,6	0,0384
EAF7	43,2	33,2	62,0	64,2	1,4	0,6203
EAP1	174,6	48,2	320,9	116,7	1,8	0,0596
EAR1	28,8	15,3	20,3	18,6	0,7	0,5054
EBP2	383,8	257,2	388,1	379,0	1,0	0,9854
EBS1	25,0	13,8	24,7	20,5	1,0	0,9789
ECI1	5,9	2,4	0,0	0,0	0,0	0,0025
ECL1	43,2	27,4	170,4	115,9	3,9	0,0765
ECM1	100,9	125,9	32,2	24,9	0,3	0,3256
ECM12	74,2	46,2	501,2	454,2	6,8	0,1106
ECM13	4,2	3,0	5,7	4,4	1,4	0,5856
ECM14	219,0	32,3	246,0	102,0	1,1	0,6313
ECM15	186,9	181,1	528,5	303,2	2,8	0,1012
ECM16	35,1	25,5	55,7	39,8	1,6	0,4169
ECM18	108,9	104,1	896,5	1034,9	8,2	0,1806
ECM19	28,3	17,5	32,8	27,1	1,2	0,7890
ECM2	23,9	18,9	73,8	70,6	3,1	0,2217
ECM21	17,5	19,3	13,0	11,3	0,7	0,7009
ECM22	21,4	10,4	19,6	20,0	0,9	0,8747
ECM25	19,1	11,0	14,0	10,4	0,7	0,5293
ECM27	23,1	11,1	27,7	20,1	1,2	0,7004
ECM29	23,6	14,7	10,9	8,2	0,5	0,1806
ECM3	185,8	32,1	116,7	62,2	0,6	0,0959
ECM30	109,9	42,8	151,7	93,3	1,4	0,4467
ECM31	22,1	8,3	10,6	12,1	0,5	0,1700
ECM32	149,2	48,3	197,6	133,9	1,3	0,5224
ECM33	336,5	213,3	280,3	173,3	0,8	0,6969
ECM34	45,9	29,7	368,7	322,7	8,0	0,0935
ECM38	18,6	14,9	13,9	18,3	0,7	0,7069
ECM4	14,7	12,8	11,8	16,7	0,8	0,7966
ECM5	262,4	161,4	212,3	142,0	0,8	0,6571
ECM7	3,1	2,8	3,1	3,7	1,0	0,9895

ECM8	29,5	24,1	13,3	10,8	0,5	0,2666
ECM9	119,3	40,3	84,0	60,0	0,7	0,3665
ECO1	15,0	4,8	4,3	8,6	0,3	0,0712
ECT1	10,6	11,1	7,7	8,0	0,7	0,6834
EDC1	9,9	6,9	52,2	37,9	5,3	0,0704
EDC2	61,0	15,6	63,9	56,4	1,0	0,9239
EDC3	18,4	16,2	19,1	19,2	1,0	0,9556
EDE1	28,4	14,3	66,7	59,0	2,4	0,2531
EDS1	56,4	60,8	29,9	21,8	0,5	0,4433
EEB1	67,3	39,7	173,2	150,2	2,6	0,2219
EFB1	370,1	194,2	541,3	403,3	1,5	0,4732
EFG1	145,6	149,2	140,9	147,4	1,0	0,9653
EFM1	19,1	16,3	22,7	23,3	1,2	0,8089
EFR3	19,6	11,2	19,5	15,6	1,0	0,9854
EFT1	0,8	1,5	0,0	0,0	0,0	0,3559
EFT2	477,3	249,7	457,3	238,9	1,0	0,9117
EGD1	142,8	56,7	190,0	100,2	1,3	0,4432
EGD2	600,6	585,9	572,2	465,8	1,0	0,9420
EGT2	39,6	36,0	20,3	14,0	0,5	0,3550
EHD3	314,3	235,5	105,9	77,0	0,3	0,1437
EHT1	8,9	9,4	7,3	8,4	0,8	0,8022
EKI1	87,4	33,8	45,3	38,0	0,5	0,1487
ELA1	12,3	2,2	11,8	8,9	1,0	0,9204
ELC1	684,9	274,2	1124,2	360,5	1,6	0,1004
ELF1	97,3	57,0	84,6	73,1	0,9	0,7940
ELG1	30,7	9,0	7,2	8,3	0,2	0,0086
ELM1	44,3	31,9	53,7	36,4	1,2	0,7095
ELO1	179,1	80,8	54,0	41,7	0,3	0,0333
ELP2	51,1	18,8	35,7	29,4	0,7	0,4109
ELP3	62,0	17,7	53,5	48,2	0,9	0,7508
ELP4	14,4	12,6	10,2	8,6	0,7	0,6015
ELP6	34,5	18,8	25,0	23,1	0,7	0,5447
EMC1	22,6	15,0	13,9	11,6	0,6	0,3919
EMC2	162,5	114,8	40,5	48,4	0,2	0,0982
EMC4	50,1	31,1	77,3	79,8	1,5	0,5499
EMC6	1396,9	625,9	627,8	467,0	0,4	0,0964
EMG1	83,2	44,9	41,0	31,5	0,5	0,1752
EMI1	24,8	19,0	14,9	19,1	0,6	0,4896
EMI2	15,2	10,1	34,8	25,5	2,3	0,2015
EMI5	22,2	9,4	86,1	99,2	3,9	0,2471
EMP24	157,5	108,9	144,8	51,9	0,9	0,8400
EMP46	39,6	15,8	141,5	121,9	3,6	0,1482
EMP47	22,3	22,0	14,9	13,1	0,7	0,5820
EMP70	1525,6	396,2	1218,8	244,2	0,8	0,2354
EMW1	11,1	3,3	2,3	2,6	0,2	0,0058
ENA1	14,9	7,7	3,1	3,7	0,2	0,0327
ENA2	15,5	13,7	0,7	1,3	0,0	0,0742
ENA5	37,3	15,3	46,9	31,7	1,3	0,6050
ENB1	37,4	24,1	50,7	46,2	1,4	0,6288
END3	276,6	33,2	194,7	131,2	0,7	0,2716

ENO1	0,0	0,0	1,3	2,5	#DIV/0!	0,3559
ENO2	3105,0	2760,0	4375,1	4097,1	1,4	0,6255
ENP1	7,5	2,4	8,4	6,0	1,1	0,8020
ENP2	78,8	61,5	116,7	101,6	1,5	0,5471
ENT1	21,5	11,7	16,7	13,9	0,8	0,6132
ENT2	145,1	58,7	94,0	68,4	0,6	0,3004
ENT3	63,6	40,7	242,3	272,1	3,8	0,2418
ENT4	7,3	5,9	15,8	16,7	2,2	0,3759
ENT5	45,8	13,0	45,6	32,2	1,0	0,9928
EOS1	163,4	72,6	229,3	233,7	1,4	0,6092
EPL1	137,8	79,0	209,1	143,1	1,5	0,4161
EPS1	98,6	37,3	61,6	41,7	0,6	0,2336
EPT1	20,5	5,5	12,7	11,3	0,6	0,2612
ERB1	88,1	67,5	66,5	66,5	0,8	0,6650
ERC1	2,3	4,5	3,6	4,6	1,6	0,6857
ERD1	46,9	23,9	202,4	165,2	4,3	0,1116
ERD2	136,8	67,1	54,2	42,5	0,4	0,0829
ERF2	7,9	2,7	7,6	7,5	1,0	0,9327
ERG1	115,5	108,5	72,4	74,8	0,6	0,5374
ERG10	392,9	94,7	310,0	303,8	0,8	0,6208
ERG11	487,5	491,9	278,5	268,5	0,6	0,4841
ERG12	15,6	8,4	10,1	11,3	0,6	0,4598
ERG13	144,0	108,8	66,4	65,0	0,5	0,2668
ERG2	139,0	42,0	56,5	41,0	0,4	0,0307
ERG20	423,8	104,9	353,8	22,7	0,8	0,2401
ERG24	45,4	27,4	18,5	12,4	0,4	0,1237
ERG25	488,1	188,7	180,4	124,0	0,4	0,0344
ERG26	38,1	9,9	26,1	17,4	0,7	0,2732
ERG27	25,4	22,1	23,9	22,5	0,9	0,9271
ERG28	93,6	34,8	68,5	46,1	0,7	0,4186
ERG3	1541,6	464,9	629,0	154,4	0,4	0,0098
ERG4	118,3	113,5	37,4	35,0	0,3	0,2223
ERG5	32,8	22,7	24,9	29,3	0,8	0,6881
ERG6	176,2	128,6	69,3	72,3	0,4	0,1973
ERG7	92,8	43,9	38,4	28,1	0,4	0,0816
ERG8	129,6	95,7	186,5	51,7	1,4	0,3351
ERG9	70,1	45,7	222,7	278,5	3,2	0,3209
ERJ5	228,3	123,9	84,9	57,2	0,4	0,0803
ERO1	60,7	24,3	97,5	93,2	1,6	0,4737
ERP1	201,1	150,9	116,8	126,8	0,6	0,4254
ERP2	231,1	111,8	68,9	60,6	0,3	0,0435
ERP3	190,0	72,5	17,3	14,6	0,1	0,0034
ERP4	61,7	51,7	34,3	33,7	0,6	0,4093
ERP5	9,2	7,5	4,1	8,3	0,5	0,4034
ERP6	141,3	22,0	231,4	204,6	1,6	0,4144
ERR3	5,2	6,1	3,8	4,3	0,7	0,7183
ERS1	1,5	3,0	0,7	1,3	0,4	0,6212
ERT1	139,8	79,6	263,2	343,3	1,9	0,5097
ERV1	63,0	19,2	178,0	167,2	2,8	0,2209
ERV14	278,7	127,8	234,5	109,8	0,8	0,6187

ERV29	50,2	37,4	41,8	40,6	0,8	0,7706
ERV41	173,0	25,7	79,7	57,3	0,5	0,0249
ERV46	20,6	12,9	9,6	9,3	0,5	0,2168
ESA1	30,4	19,0	20,1	17,1	0,7	0,4528
ESBP6	60,2	47,3	37,5	29,6	0,6	0,4461
ESC1	92,4	104,2	104,8	92,8	1,1	0,8645
ESC2	12,0	9,5	33,7	31,7	2,8	0,2359
ESC8	23,6	11,2	70,4	109,8	3,0	0,4282
ESF1	1113,2	732,2	975,8	660,6	0,9	0,7898
ESF2	85,6	40,3	143,1	124,0	1,7	0,4117
ESP1	310,7	78,1	112,3	86,3	0,4	0,0143
ESS1	74,4	16,2	243,3	34,0	3,3	0,0001
EST1	40,1	18,2	140,1	96,0	3,5	0,0866
EST2	10,0	13,5	88,0	108,1	8,8	0,2019
EST3	6,8	5,7	9,2	10,6	1,3	0,7089
ETP1	13,5	7,0	37,7	27,4	2,8	0,1385
ETR1	8,4	13,0	15,2	18,0	1,8	0,5617
ETT1	99,0	19,1	194,9	8,5	2,0	0,0001
EUG1	52,4	14,8	27,4	21,3	0,5	0,1014
EXG1	590,8	488,1	335,6	212,7	0,6	0,3747
EXG2	102,1	23,2	207,5	242,5	2,0	0,4203
EXO1	38,8	13,6	13,7	9,4	0,4	0,0231
EXO5	50,0	11,4	75,8	115,4	1,5	0,6716
EXO70	71,2	27,3	17,0	16,0	0,2	0,0139
EXO84	13,7	9,3	8,0	10,2	0,6	0,4361
FAA1	72,3	15,6	95,2	71,8	1,3	0,5552
FAA2	2,7	5,5	3,1	3,7	1,1	0,9224
FAA3	206,9	58,0	160,9	120,4	0,8	0,5171
FAA4	1104,1	462,9	529,7	325,0	0,5	0,0885
FAB1	17,4	16,7	15,8	14,7	0,9	0,8889
FAD1	4,2	4,1	8,9	9,1	2,1	0,3811
FAF1	35,5	24,4	21,7	17,3	0,6	0,3928
FAL1	12,6	6,3	6,7	8,4	0,5	0,3028
FAP1	71,0	85,1	22,0	16,9	0,3	0,3019
FAP7	70,2	23,5	40,2	31,8	0,6	0,1786
FAR1	91,2	48,8	58,3	47,5	0,6	0,3714
FAR10	55,9	21,9	52,8	38,9	0,9	0,8948
FAR11	28,6	12,3	74,9	70,6	2,6	0,2437
FAR3	23,7	12,2	21,8	19,6	0,9	0,8785
FAR7	17,7	20,2	13,1	13,3	0,7	0,7170
FAR8	16,6	9,0	8,2	8,6	0,5	0,2216
FAS1	79,5	46,4	89,3	124,9	1,1	0,8878
FAS2	117,0	38,8	154,8	218,9	1,3	0,7454
FAT1	15,3	11,6	38,9	32,8	2,5	0,2240
FAU1	11,4	4,0	10,9	7,5	1,0	0,9224
FBA1	3133,9	2649,5	4859,7	4446,1	1,6	0,5296
FBP1	33,8	13,0	35,4	27,4	1,0	0,9191
FBP26	8,2	5,8	8,4	6,2	1,0	0,9559
FCF1	90,1	31,9	26,4	38,3	0,3	0,0431
FCF2	87,9	59,2	123,3	122,5	1,4	0,6222

FCJ1	28,4	27,3	64,3	66,5	2,3	0,3561
FCP1	144,2	91,4	86,2	67,1	0,6	0,3456
FCY1	235,8	105,3	231,3	194,5	1,0	0,9686
FCY2	241,6	129,5	84,0	80,9	0,3	0,0846
FCY21	64,4	12,5	45,2	30,1	0,7	0,2827
FCY22	10,8	13,3	6,3	6,4	0,6	0,5628
FDC1	22,2	10,8	5,5	4,9	0,2	0,0306
FDH1	2,7	3,1	0,7	1,3	0,2	0,2845
FDH2	6,2	5,2	0,0	0,0	0,0	0,0553
FEN1	42,0	17,9	20,9	14,1	0,5	0,1135
FEN2	21,8	25,4	23,1	25,8	1,1	0,9437
FES1	14,1	14,1	41,7	45,1	3,0	0,2873
FET3	1820,8	1244,0	1249,4	895,3	0,7	0,4840
FET4	234,3	119,6	298,1	131,3	1,3	0,4992
FET5	28,5	22,3	16,0	14,7	0,6	0,3838
FHL1	28,7	15,9	8,1	10,8	0,3	0,0757
FHN1	2765,8	1543,7	1456,0	550,3	0,5	0,1611
FIG1	0,8	1,5	10,7	10,7	14,1	0,1133
FIG2	9,7	4,4	40,5	31,7	4,2	0,1021
FIG4	37,0	9,0	134,9	34,7	3,6	0,0016
FIN1	25,3	5,8	114,9	153,8	4,5	0,2887
FIP1	40,5	34,9	29,1	31,4	0,7	0,6442
FIR1	153,5	21,8	58,2	46,7	0,4	0,0101
FIS1	20,4	11,3	18,4	21,5	0,9	0,8745
FIT1	4,6	5,4	1,2	2,4	0,3	0,2923
FIT3	49,8	14,4	45,0	34,7	0,9	0,8057
FKH1	36,8	16,1	4,8	6,8	0,1	0,0106
FKH2	12,4	8,3	85,2	76,5	6,9	0,1073
FKS1	210,9	112,4	153,6	86,8	0,7	0,4501
FKS3	72,8	26,4	25,8	21,7	0,4	0,0333
FLC1	101,0	25,5	58,8	44,7	0,6	0,1516
FLC2	230,8	128,1	122,3	83,0	0,5	0,2052
FLC3	17,4	9,5	10,9	10,7	0,6	0,4027
FLD1	9,0	11,6	1,3	2,5	0,1	0,2413
FLO1	61,9	51,4	72,1	59,8	1,2	0,8046
FLO10	0,9	1,8	3,7	3,2	4,1	0,1774
FLO5	2,4	2,8	0,7	1,3	0,3	0,3132
FLO8	8,1	3,9	1,9	3,8	0,2	0,0637
FLO9	267,8	163,7	127,4	144,8	0,5	0,2463
FLP1	725,1	303,3	2349,0	2587,7	3,2	0,2590
FLR1	60,5	35,9	21,6	18,5	0,4	0,1021
FLX1	10,9	2,9	13,3	9,8	1,2	0,6633
FMC1	38,0	32,7	32,9	38,3	0,9	0,8471
FMN1	60,4	71,6	129,0	132,0	2,1	0,3962
FMO1	38,4	9,9	25,6	19,2	0,7	0,2775
FMP10	4,2	5,0	8,5	10,4	2,0	0,4832
FMP16	29,9	23,1	87,9	68,8	2,9	0,1611
FMP21	524,3	157,0	512,1	271,2	1,0	0,9406
FMP23	14,0	8,3	25,8	23,7	1,8	0,3831
FMP25	8,9	6,6	2,9	3,4	0,3	0,1566

FMP27	224,1	121,1	185,8	162,2	0,8	0,7186
FMP30	3,2	3,7	2,8	3,4	0,9	0,8795
FMP32	21,6	15,7	14,7	17,0	0,7	0,5681
FMP33	6,8	5,3	10,8	8,7	1,6	0,4579
FMP37	97,0	46,3	85,0	66,8	0,9	0,7765
FMP40	5,2	2,3	1,8	3,6	0,3	0,1600
FMP41	31,4	15,6	21,2	18,6	0,7	0,4326
FMP45	6,3	7,4	93,1	158,9	14,7	0,3174
FMP46	4,4	4,0	4,9	4,4	1,1	0,8553
FMP48	3,9	4,7	12,8	13,1	3,3	0,2465
FMP52	17,9	14,1	20,9	22,7	1,2	0,8300
FMS1	50,6	20,0	36,9	27,6	0,7	0,4501
FMT1	4,6	7,6	0,0	0,0	0,0	0,2771
FOB1	136,1	103,8	40,8	41,7	0,3	0,1393
FOL1	40,6	16,8	66,3	58,7	1,6	0,4319
FOL2	382,2	143,7	783,2	107,7	2,0	0,0043
FOL3	9,3	1,7	8,2	10,2	0,9	0,8500
FOX2	62,6	16,6	107,5	84,7	1,7	0,3380
FPK1	8,7	11,8	16,4	13,1	1,9	0,4157
FPR2	160,2	65,8	153,8	105,5	1,0	0,9211
FPR3	1452,2	1086,1	2459,5	1967,1	1,7	0,4045
FPR4	299,6	155,6	453,4	172,2	1,5	0,2333
FPS1	63,9	23,1	54,1	49,7	0,8	0,7317
FRA1	46,2	17,5	63,5	44,6	1,4	0,4972
FRA2	51,1	46,7	34,9	32,8	0,7	0,5915
FRE1	21,8	6,4	109,6	92,7	5,0	0,1078
FRE2	16,6	7,8	11,0	12,7	0,7	0,4825
FRE3	28,3	15,2	49,8	36,8	1,8	0,3230
FRE4	163,5	72,6	189,3	65,4	1,2	0,6166
FRE5	13,7	15,5	10,3	11,2	0,7	0,7295
FRE6	18,7	9,0	20,4	17,6	1,1	0,8675
FRE7	73,0	70,6	44,5	85,6	0,6	0,6253
FRE8	8,6	9,3	3,9	4,9	0,4	0,4029
FRK1	33,1	19,3	13,3	13,1	0,4	0,1401
FRM2	15,2	13,6	9,3	6,9	0,6	0,4719
FRQ1	45,8	29,7	44,4	41,3	1,0	0,9581
FRS1	826,1	414,0	553,7	363,4	0,7	0,3609
FRS2	81,2	20,5	59,9	49,6	0,7	0,4573
FRT1	147,1	100,4	235,3	159,1	1,6	0,3846
FRT2	3,7	1,9	99,1	191,6	27,1	0,3575
FSF1	149,0	28,9	157,9	59,8	1,1	0,7968
FSH1	76,0	14,1	36,6	26,9	0,5	0,0408
FSH2	8,8	6,6	10,7	10,1	1,2	0,7591
FSH3	56,0	58,5	74,6	54,8	1,3	0,6594
FTH1	115,7	119,9	55,4	40,5	0,5	0,3771
FTR1	70,6	28,8	32,7	29,0	0,5	0,1127
FUI1	220,0	100,7	179,3	157,7	0,8	0,6784
FUM1	107,5	30,9	241,3	165,8	2,2	0,1638
FUN12	1517,7	1208,9	2984,6	2512,2	2,0	0,3332
FUN14	48,3	49,6	48,1	44,1	1,0	0,9965

FUN19	9,1	4,6	3,2	6,3	0,3	0,1820
FUN26	17,5	19,3	7,5	12,5	0,4	0,4164
FUN30	224,8	136,5	230,1	165,1	1,0	0,9622
FUR1	64,7	16,8	41,4	34,5	0,6	0,2708
FUR4	78,2	49,7	99,7	76,1	1,3	0,6525
FUS1	15,5	11,3	107,7	79,1	7,0	0,0603
FUS2	11,7	10,7	48,9	34,5	4,2	0,0849
FUS3	36,4	10,2	61,3	44,6	1,7	0,3182
FYV1	253,6	134,6	194,3	135,6	0,8	0,5575
FYV10	106,5	84,4	22,0	17,7	0,2	0,0978
FYV4	66,5	13,6	49,0	33,5	0,7	0,3704
FYV6	54,8	51,4	19,0	18,4	0,3	0,2375
FYV8	57,6	21,5	20,5	14,5	0,4	0,0285
FZF1	81,1	30,1	175,2	130,7	2,2	0,2105
FZO1	33,0	30,1	40,5	35,2	1,2	0,7547
GAA1	37,5	17,2	27,2	21,0	0,7	0,4793
GAB1	25,1	25,9	16,0	18,7	0,6	0,5889
GAC1	233,9	71,2	121,8	138,7	0,5	0,2006
GAD1	28,4	19,7	121,5	88,5	4,3	0,0859
GAL1	66,2	16,5	176,7	122,3	2,7	0,1236
GAL10	32,5	7,0	129,8	105,8	4,0	0,1163
GAL11	22,9	19,8	20,0	17,0	0,9	0,8308
GAL2	48,5	45,6	125,7	90,8	2,6	0,1794
GAL3	3,0	5,9	2,2	2,5	0,7	0,8132
GAL4	170,9	35,8	160,5	113,7	0,9	0,8673
GAL7	1,7	3,4	2,3	2,6	1,3	0,8050
GAL80	78,5	60,9	181,8	196,0	2,3	0,3531
GAL83	144,2	77,7	119,7	60,3	0,8	0,6356
GAP1	49,8	47,7	4,6	3,9	0,1	0,1077
GAR1	872,5	898,2	1345,4	1146,1	1,5	0,5400
GAS2	3094,4	2447,5	3062,9	2556,1	1,0	0,9864
GAS3	58,1	19,6	19,5	14,7	0,3	0,0199
GAS4	46,3	32,7	22,7	32,7	0,5	0,3466
GAS5	31,2	16,4	23,9	24,0	0,8	0,6313
GAT1	177,2	20,9	86,5	81,9	0,5	0,0756
GAT2	25,4	19,6	21,6	17,3	0,9	0,7810
GAT3	1,7	2,1	2,0	4,0	1,2	0,8895
GAT4	2,7	5,5	0,0	0,0	0,0	0,3559
GBP2	106,8	96,6	38,8	34,5	0,4	0,2331
GCD1	35,9	11,6	18,4	14,4	0,5	0,1066
GCD10	70,5	13,7	51,8	37,1	0,7	0,3825
GCD11	82,1	67,3	60,4	59,7	0,7	0,6456
GCD14	19,9	16,2	2,6	5,3	0,1	0,0892
GCD2	73,7	62,0	114,3	113,5	1,6	0,5536
GCD6	214,1	57,6	515,6	430,5	2,4	0,2143
GCD7	77,1	38,2	69,7	62,4	0,9	0,8457
GCN1	72,2	47,7	52,2	53,8	0,7	0,5988
GCN2	31,1	16,3	15,1	11,5	0,5	0,1614
GCN20	160,7	105,0	100,8	80,4	0,6	0,3998
GCN3	13,1	17,1	6,9	9,1	0,5	0,5454

GCN4	583,8	264,3	1024,3	503,8	1,8	0,1725
GCN5	37,1	5,1	18,1	12,3	0,5	0,0286
GCR1	27,7	26,4	31,1	24,6	1,1	0,8553
GCR2	69,5	35,9	73,6	64,0	1,1	0,9147
GCS1	26,6	26,4	6,7	8,1	0,3	0,1991
GCV1	245,1	256,7	105,9	107,2	0,4	0,3552
GCV2	423,5	283,9	95,6	80,0	0,2	0,0679
GCV3	156,7	137,8	132,3	153,1	0,8	0,8210
GCY1	100,2	55,9	52,2	39,9	0,5	0,2117
GDA1	175,5	68,8	136,3	68,9	0,8	0,4523
GDB1	14,1	11,6	13,0	10,8	0,9	0,8881
GDE1	27,5	18,0	72,5	100,7	2,6	0,4125
GDH1	286,0	189,7	54,4	55,9	0,2	0,0577
GDH2	30,7	18,7	55,0	49,5	1,8	0,3939
GDH3	458,5	251,9	164,5	172,8	0,4	0,1026
GDI1	283,4	29,7	215,1	158,9	0,8	0,4310
GDS1	527,0	136,9	400,7	214,7	0,8	0,3596
GDT1	152,9	81,8	40,3	27,5	0,3	0,0400
GEA1	8,5	7,9	7,9	5,8	0,9	0,9003
GEA2	52,4	26,0	30,5	22,0	0,6	0,2467
GEF1	15,9	10,8	7,5	6,4	0,5	0,2279
GEM1	2,1	2,8	3,1	2,3	1,5	0,5909
GEP3	48,7	23,7	42,7	34,7	0,9	0,7851
GEP4	117,6	58,7	123,1	81,3	1,0	0,9162
GEP5	63,6	61,4	16,2	26,5	0,3	0,2064
GEP7	49,2	25,5	23,8	19,9	0,5	0,1682
GET2	42,5	27,6	32,9	24,2	0,8	0,6203
GET3	94,0	85,0	80,7	81,0	0,9	0,8284
GET4	48,5	30,8	22,6	23,9	0,5	0,2321
GFA1	78,2	45,1	116,1	81,8	1,5	0,4481
GFD1	94,7	48,1	168,3	159,0	1,8	0,4094
GFD2	5,9	4,1	9,0	13,1	1,5	0,6665
GGA1	12,8	5,4	14,0	10,8	1,1	0,8408
GGA2	8,1	2,3	16,4	14,1	2,0	0,2912
GGC1	172,0	129,4	159,7	131,6	0,9	0,8981
GIC1	37,3	51,1	28,5	22,3	0,8	0,7629
GIC2	66,6	49,9	60,2	72,2	0,9	0,8896
GID7	10,5	5,2	15,5	12,3	1,5	0,4833
GIM3	185,1	50,7	323,4	84,4	1,7	0,0307
GIM4	170,3	151,4	118,2	112,1	0,7	0,6008
GIN4	144,9	112,6	26,9	20,1	0,2	0,0846
GIP1	12,4	9,3	44,9	76,7	3,6	0,4327
GIP2	16,0	15,0	13,7	11,2	0,9	0,8141
GIP3	26,6	9,7	12,8	9,0	0,5	0,0818
GIP4	3,4	3,9	3,0	5,9	0,9	0,9100
GIR2	128,7	32,8	98,5	74,5	0,8	0,4859
GIS1	51,9	36,8	84,9	90,9	1,6	0,5264
GIS2	266,7	96,3	333,4	240,2	1,3	0,6246
GIS3	17,5	14,1	38,4	36,0	2,2	0,3215
GIS4	43,0	6,3	36,3	30,2	0,8	0,6758

GIT1	6,4	5,3	8,5	6,6	1,3	0,6293
GLC3	67,6	36,0	60,8	41,2	0,9	0,8120
GLC7	201,8	121,8	394,6	381,7	2,0	0,3730
GLC8	141,8	86,2	357,6	43,9	2,5	0,0043
GLE1	165,6	111,9	156,7	106,1	0,9	0,9114
GLE2	41,3	27,2	17,5	15,9	0,4	0,1817
GLG1	0,0	0,0	1,3	2,5	#DIV/0!	0,3559
GLG2	52,8	19,9	67,6	84,1	1,3	0,7446
GLK1	123,0	78,8	527,9	580,7	4,3	0,2163
GLN1	5543,5	2588,9	2453,6	1275,3	0,4	0,0760
GLN3	12,3	13,3	15,8	17,9	1,3	0,7647
GLN4	23,3	22,7	18,9	17,0	0,8	0,7673
GLO1	22,5	8,2	28,1	19,1	1,2	0,6116
GLO2	94,6	38,7	118,2	99,9	1,2	0,6748
GLO3	75,5	45,0	35,5	28,7	0,5	0,1848
GLO4	1420,2	1349,1	1131,0	789,9	0,8	0,7240
GLR1	173,9	84,0	172,5	90,3	1,0	0,9822
GLT1	82,3	19,4	17,6	14,2	0,2	0,0017
GLY1	55,3	23,5	68,2	46,1	1,2	0,6360
GNA1	204,0	242,3	216,5	226,6	1,1	0,9423
GND1	172,4	130,0	127,2	109,8	0,7	0,6149
GND2	3,5	4,9	3,0	5,9	0,9	0,8997
GNP1	632,2	371,6	168,4	64,1	0,3	0,0491
GNT1	9,6	7,3	9,0	8,4	0,9	0,9158
GON7	90,4	33,7	114,7	138,0	1,3	0,7437
GOR1	10,4	1,2	57,7	39,0	5,5	0,0517
GOS1	81,0	44,5	58,8	43,0	0,7	0,5004
GOT1	41,7	58,4	57,5	72,6	1,4	0,7457
GPA1	104,7	55,0	339,4	289,1	3,2	0,1618
GPA2	130,4	82,9	279,7	71,8	2,1	0,0345
GPB1	23,1	7,9	26,3	21,5	1,1	0,7889
GPB2	39,5	22,3	32,3	27,1	0,8	0,6976
GPD1	92,3	8,9	61,0	50,0	0,7	0,2636
GPD2	139,9	80,4	136,2	127,3	1,0	0,9629
GPG1	41,1	6,7	67,9	93,8	1,7	0,5893
GPH1	30,1	14,9	308,6	456,8	10,3	0,2687
GPI10	74,1	17,6	196,6	54,7	2,7	0,0053
GPI11	86,7	107,7	60,1	72,9	0,7	0,6961
GPI12	17,9	7,6	17,0	16,1	0,9	0,9197
GPI13	122,6	49,4	67,8	49,1	0,6	0,1672
GPI14	79,8	9,0	50,5	33,8	0,6	0,1449
GPI15	57,6	60,4	35,0	34,9	0,6	0,5406
GPI16	42,0	22,5	30,3	23,3	0,7	0,4988
GPI17	61,2	38,4	126,6	173,3	2,1	0,4892
GPI18	88,5	46,4	101,1	84,1	1,1	0,8012
GPI19	25,1	31,3	15,3	14,2	0,6	0,5891
GPI2	38,3	13,2	332,1	451,7	8,7	0,2411
GPI8	11,5	7,0	12,8	10,8	1,1	0,8543
GPM2	31,5	18,0	86,6	84,4	2,7	0,2486
GPM3	21,8	10,1	26,7	19,4	1,2	0,6662

GPR1	161,0	46,7	255,2	106,1	1,6	0,1552
GPT2	22,4	19,6	124,8	68,4	5,6	0,0281
GPX1	20,4	13,4	108,3	121,3	5,3	0,1997
GPX2	35,3	17,3	20,5	18,5	0,6	0,2849
GRC3	10,3	4,3	8,2	11,6	0,8	0,7467
GRE1	64,9	55,4	192,8	173,3	3,0	0,2095
GRE2	40,8	27,8	21,6	17,5	0,5	0,2894
GRE3	255,7	96,5	1306,2	648,1	5,1	0,0185
GRH1	36,8	29,8	49,7	57,6	1,4	0,7049
GRR1	67,6	50,3	61,9	56,3	0,9	0,8850
GRS1	1283,1	652,1	1118,3	557,4	0,9	0,7139
GRS2	8,2	5,4	100,3	163,4	12,2	0,3029
GRX1	85,6	40,6	260,3	240,3	3,0	0,2016
GRX3	261,0	72,2	176,7	138,7	0,7	0,3223
GRX4	18,5	10,0	5,6	5,5	0,3	0,0643
GRX5	225,7	71,6	179,7	120,6	0,8	0,5363
GRX7	64,7	30,0	44,2	34,7	0,7	0,4058
GSC2	36,7	40,9	9,6	11,2	0,3	0,2487
GSF2	13,1	13,4	13,4	14,7	1,0	0,9776
GSG1	9,2	4,9	5,5	6,8	0,6	0,4096
GSH1	19,1	18,3	87,1	65,6	4,6	0,0928
GSH2	54,8	18,4	117,3	120,3	2,1	0,3432
GSM1	7,1	2,5	10,9	16,9	1,5	0,6706
GSP1	5257,1	2095,7	4960,1	1728,5	0,9	0,8342
GSP2	70,8	13,8	91,7	62,7	1,3	0,5387
GSY1	6,1	5,2	21,0	21,5	3,4	0,2281
GSY2	63,4	19,6	105,6	77,7	1,7	0,3333
GTB1	44,4	24,0	33,3	23,3	0,7	0,5302
GTO1	13,2	9,3	9,3	8,2	0,7	0,5446
GTO3	34,6	29,6	158,7	231,5	4,6	0,3284
GTR1	15,3	9,5	12,1	8,9	0,8	0,6446
GTR2	0,0	0,0	0,0	0,0	#DIV/0!	#DIV/0!
GTS1	32,6	14,0	30,0	25,7	0,9	0,8627
GTT2	7,9	5,5	46,9	45,3	5,9	0,1380
GUA1	196,6	179,0	187,6	185,9	1,0	0,9464
GUD1	2,3	4,5	5,1	6,2	2,3	0,4841
GUF1	15,9	5,8	8,5	7,3	0,5	0,1626
GUK1	172,5	100,3	86,4	64,2	0,5	0,1983
GUP1	25,8	17,4	11,5	8,9	0,4	0,1950
GUP2	63,1	27,3	20,7	17,5	0,3	0,0399
GUS1	115,7	132,0	91,8	99,1	0,8	0,7818
GUT1	16,6	9,0	9,8	7,2	0,6	0,2778
GUT2	68,5	46,2	75,8	52,1	1,1	0,8403
GVP36	37,2	26,8	43,7	48,4	1,2	0,8205
GWT1	24,6	13,4	51,1	17,9	2,1	0,0556
GYL1	85,2	27,0	76,5	52,5	0,9	0,7773
GYP1	76,5	17,1	104,8	79,6	1,4	0,5126
GYP5	76,7	30,8	89,3	63,9	1,2	0,7334
GYP6	389,3	116,7	386,4	105,9	1,0	0,9724
GYP7	15,3	12,2	19,5	15,5	1,3	0,6835

GYP8	29,7	26,7	39,9	41,3	1,3	0,6937
GZF3	108,3	76,6	55,3	39,3	0,5	0,2650
HAA1	91,3	50,3	136,9	91,7	1,5	0,4163
HAC1	25,4	17,2	23,0	31,1	0,9	0,8964
HAL1	129,3	47,2	83,3	56,8	0,6	0,2596
HAL5	42,8	17,5	42,9	32,6	1,0	0,9960
HAL9	16,8	12,2	31,8	27,1	1,9	0,3547
HAM1	24,0	11,9	14,5	12,6	0,6	0,3136
HAP1	344,5	149,8	2175,5	1660,7	6,3	0,0705
HAP2	67,3	35,4	48,6	47,3	0,7	0,5494
HAP3	19,5	22,1	96,7	165,7	5,0	0,3914
HAP4	151,5	21,8	188,8	126,5	1,2	0,5826
HAP5	19,7	8,7	107,4	159,3	5,5	0,3137
HAS1	34,8	33,8	78,0	74,8	2,2	0,3323
HAT2	36,3	27,1	37,4	35,6	1,0	0,9645
HBN1	6,2	7,5	4,2	5,0	0,7	0,6762
HBS1	54,6	30,0	62,7	58,5	1,1	0,8131
HBT1	51,9	36,4	71,2	109,6	1,4	0,7483
HCA4	78,9	42,0	110,0	97,7	1,4	0,5807
HCH1	43,3	16,6	30,8	27,6	0,7	0,4647
HCM1	16,1	11,9	4,4	3,3	0,3	0,1068
HCR1	828,0	844,6	622,8	594,4	0,8	0,7048
HCS1	19,2	7,3	12,4	9,4	0,6	0,2938
HDA1	44,6	9,7	67,7	45,4	1,5	0,3592
HDA2	92,5	50,1	102,9	48,4	1,1	0,7755
HDA3	12,6	7,8	6,2	6,7	0,5	0,2616
HED1	0,0	0,0	0,0	0,0	#DIV/0!	#DIV/0!
HEF3	15,7	4,7	18,7	18,7	1,2	0,7658
HEH2	62,3	25,0	57,1	40,5	0,9	0,8315
HEK2	58,2	8,1	20,2	21,9	0,3	0,0173
HEM1	396,8	408,5	254,3	61,4	0,6	0,5159
HEM12	53,0	23,7	18,1	13,9	0,3	0,0438
HEM13	85,1	42,9	138,8	144,2	1,6	0,5018
HEM14	0,0	0,0	0,7	1,3	#DIV/0!	0,3559
HEM15	29,2	21,3	8,4	5,8	0,3	0,1096
HEM2	56,2	30,5	52,8	42,0	0,9	0,9026
HEM3	8,4	9,7	26,4	28,0	3,1	0,2702
HEM4	15,2	13,0	5,3	4,3	0,3	0,1972
HER1	17,3	5,5	17,3	12,1	1,0	0,9982
HER2	0,0	0,0	1,3	2,6	#DIV/0!	0,3559
HES1	4,9	1,4	0,0	0,0	0,0	0,0004
HFA1	73,6	53,1	45,3	30,7	0,6	0,3913
HFD1	16,2	10,5	24,8	18,0	1,5	0,4417
HFI1	12,5	9,0	6,0	10,3	0,5	0,3811
HFM1	102,1	32,2	261,3	161,0	2,6	0,1004
HGH1	65,8	23,9	102,6	89,4	1,6	0,4576
HHO1	256,8	235,0	59,2	51,9	0,2	0,1518
HHT2	516,4	444,1	185,0	129,3	0,4	0,2018
HHY1	0,0	0,0	7,0	5,7	#DIV/0!	0,0508
HIF1	25,0	14,7	4,9	5,1	0,2	0,0412

HIM1	103,2	54,4	196,9	139,0	1,9	0,2560
HIP1	93,0	70,9	150,5	160,4	1,6	0,5363
HIR1	9,2	15,6	2,5	5,1	0,3	0,4451
HIR2	8,4	8,8	8,3	11,2	1,0	0,9942
HIR3	35,8	23,1	42,2	37,0	1,2	0,7788
HIS1	570,0	143,3	397,5	106,1	0,7	0,1011
HIS2	105,1	34,8	135,7	101,3	1,3	0,5890
HIS3	34,0	25,0	56,0	52,1	1,6	0,4752
HIS4	280,5	191,2	109,8	91,0	0,4	0,1580
HIS5	212,4	134,1	359,0	395,0	1,7	0,5086
HIS6	23,3	8,2	109,8	202,9	4,7	0,4268
HIS7	178,0	90,6	289,8	185,7	1,6	0,3208
HIT1	12,0	10,6	65,0	104,1	5,4	0,3496
HKR1	41,3	11,9	40,3	31,1	1,0	0,9522
HLJ1	31,5	26,0	25,7	25,5	0,8	0,7603
HLR1	28,0	7,5	7,4	6,0	0,3	0,0051
HMF1	77,8	37,8	80,1	77,6	1,0	0,9581
HMG1	110,9	39,6	140,9	58,8	1,3	0,4299
HMG2	120,4	38,9	132,8	107,4	1,1	0,8359
HMI1	8,5	4,1	7,2	8,9	0,9	0,8093
HMLALPHA1	0,0	0,0	0,0	0,0	#DIV/0!	#DIV/0!
HMO1	879,9	633,0	760,2	520,2	0,9	0,7800
HMRA1	47,5	27,1	21,5	22,5	0,5	0,1903
HMS1	10,3	9,6	10,1	8,1	1,0	0,9682
HMS2	19,3	11,4	25,4	23,0	1,3	0,6506
HMT1	30,4	21,8	173,9	163,3	5,7	0,1322
HMX1	15,8	15,4	15,1	21,8	1,0	0,9587
HNM1	363,8	292,7	112,0	92,2	0,3	0,1519
HNT1	748,0	76,0	491,1	23,5	0,7	0,0007
HNT2	53,9	54,4	12,6	11,5	0,2	0,1873
HO	40,6	20,9	2,4	2,8	0,1	0,0111
HOC1	202,7	66,8	295,2	155,4	1,5	0,3164
HOF1	12,4	8,4	12,3	10,3	1,0	0,9880
HOG1	184,0	73,7	123,3	84,2	0,7	0,3195
HOL1	304,8	203,4	283,6	113,3	0,9	0,8619
HOM2	195,9	142,2	450,5	399,1	2,3	0,2747
HOM3	204,7	110,0	304,3	160,0	1,5	0,3444
HOM6	133,7	102,4	140,1	159,8	1,0	0,9486
HOP2	0,0	0,0	1,0	2,0	#DIV/0!	0,3559
HOR2	789,1	591,8	295,7	109,9	0,4	0,1523
HOS1	69,0	42,1	46,5	53,2	0,7	0,5308
HOS2	99,1	68,1	96,6	110,0	1,0	0,9705
HOS3	42,2	20,4	10,7	10,7	0,3	0,0340
HOS4	480,1	315,6	1594,4	874,5	3,3	0,0535
HOT1	2,3	3,2	1,6	3,3	0,7	0,7906
HOT13	11,0	5,9	10,5	10,9	1,0	0,9395
HPA2	13,2	7,2	6,1	9,4	0,5	0,2783
HPA3	34,3	28,1	17,8	18,1	0,5	0,3589
HPC2	142,7	120,4	236,3	174,2	1,7	0,4112
HPF1	17,1	6,5	68,6	47,1	4,0	0,0736

HPM1	69,7	31,3	20,7	16,5	0,3	0,0324
HPR1	12,9	12,4	24,4	19,5	1,9	0,3572
HPT1	1541,0	604,6	155,7	125,5	0,1	0,0042
HRB1	34,4	27,3	58,3	55,4	1,7	0,4676
HRD1	16,7	13,2	16,5	12,3	1,0	0,9868
HRD3	10,8	11,8	2,5	5,1	0,2	0,2431
HRK1	81,6	44,9	131,0	91,2	1,6	0,3693
HRP1	237,6	118,3	52,6	35,2	0,2	0,0241
HRQ1	26,0	10,4	67,4	111,5	2,6	0,4873
HRR25	38,1	27,3	31,4	28,9	0,8	0,7469
HRT1	66,4	59,9	150,6	95,1	2,3	0,1843
HRT3	13,4	8,2	8,7	7,7	0,7	0,4394
HSC82	2058,3	531,1	2925,7	1711,1	1,4	0,3703
HSE1	85,0	57,0	102,2	78,6	1,2	0,7351
HSF1	11,1	6,9	67,5	94,3	6,1	0,2776
HSH155	23,5	8,4	11,2	19,1	0,5	0,2807
HSH49	79,7	20,9	13,1	14,5	0,2	0,0019
HSK3	45,1	30,9	27,7	26,3	0,6	0,4235
HSL1	168,5	102,2	87,4	90,6	0,5	0,2799
HSL7	55,8	5,5	14,7	15,9	0,3	0,0028
HSM3	110,5	25,6	240,9	229,9	2,2	0,3022
HSP104	17,1	13,4	181,4	98,5	10,6	0,0163
HSP12	1942,5	1839,9	1157,5	813,7	0,6	0,4648
HSP26	24,5	15,4	36,7	39,6	1,5	0,5861
HSP30	50,9	27,7	321,0	357,5	6,3	0,1827
HSP31	152,6	55,9	115,5	111,3	0,8	0,5741
HSP33	92,5	96,1	21,3	17,0	0,2	0,1944
HSP42	29,5	23,5	144,2	129,3	4,9	0,1315
HSP60	192,8	99,9	449,6	203,2	2,3	0,0638
HSP78	63,6	24,2	237,2	191,9	3,7	0,1228
HSP82	202,2	161,0	1564,5	1162,6	7,7	0,0593
HST1	223,6	138,9	199,3	52,1	0,9	0,7547
HST2	59,5	26,6	136,7	140,2	2,3	0,3209
HST3	2,8	5,7	0,0	0,0	0,0	0,3559
HST4	4,3	3,3	7,9	8,5	1,8	0,4602
HSV2	3,5	4,9	0,7	1,3	0,2	0,3088
HTA1	6806,9	3169,5	958,9	449,3	0,1	0,0107
HTA2	8,3	6,7	5,7	5,5	0,7	0,5847
HTB1	1547,0	1356,3	195,9	202,9	0,1	0,0963
HTB2	1169,6	656,1	121,2	68,7	0,1	0,0191
HTD2	111,9	47,0	62,4	72,0	0,6	0,2934
HTS1	77,6	40,4	81,3	67,6	1,0	0,9276
HTZ1	69,1	46,8	28,2	20,0	0,4	0,1587
HUA1	65,1	24,0	308,6	21,0	4,7	0,0000
HUA2	7,6	6,1	0,0	0,0	0,0	0,0456
HUG1	16,5	11,6	11,2	8,0	0,7	0,4793
HUL4	11,7	10,4	18,4	13,1	1,6	0,4578
HUL5	24,1	14,2	54,4	40,9	2,3	0,2110
HUT1	75,0	72,0	65,3	73,6	0,9	0,8565
HVG1	5,1	4,3	4,2	5,6	0,8	0,8168

HXK1	126,3	117,3	453,0	227,0	3,6	0,0431
HXK2	383,6	314,1	531,6	273,8	1,4	0,5042
HXT1	122,5	51,9	85,1	77,8	0,7	0,4540
HXT10	9,2	4,1	1,8	3,6	0,2	0,0341
HXT11	13,5	9,7	15,2	11,0	1,1	0,8319
HXT12	23,6	12,5	36,4	24,6	1,5	0,3901
HXT13	2,9	3,8	9,1	8,0	3,1	0,2081
HXT14	1,2	2,4	0,0	0,0	0,0	0,3559
HXT15	148,2	61,0	202,0	142,8	1,4	0,5140
HXT17	19,0	11,5	34,7	25,7	1,8	0,3083
HXT2	174,4	159,7	62,7	62,7	0,4	0,2407
HXT3	1155,0	522,8	1020,5	517,3	0,9	0,7270
HXT4	30,2	16,3	294,3	248,2	9,8	0,0778
HXT5	20,3	9,7	119,6	83,5	5,9	0,0561
HXT6	1,5	3,0	2,5	5,1	1,7	0,7337
HXT7	333,5	194,4	673,6	388,3	2,0	0,1683
HXT8	3,3	2,2	78,9	149,9	23,9	0,3524
HXT9	4,5	4,1	1,3	2,6	0,3	0,2409
HYM1	26,2	14,1	28,2	19,2	1,1	0,8697
HYR1	65,1	48,0	65,3	68,5	1,0	0,9981
IAH1	22,8	11,1	110,0	111,8	4,8	0,1717
IBA57	2,5	3,2	1,2	2,4	0,5	0,5467
IBD2	19,5	7,2	8,0	7,8	0,4	0,0738
ICE2	44,0	24,7	52,5	52,8	1,2	0,7815
ICL1	381,0	207,4	105,1	70,7	0,3	0,0454
ICL2	36,4	12,1	229,8	254,0	6,3	0,1789
ICP55	32,3	18,9	33,7	29,0	1,0	0,9409
ICS2	198,2	63,0	223,9	59,8	1,1	0,5765
ICS3	10,5	3,1	4,7	3,4	0,4	0,0454
ICT1	15,4	7,6	9,5	9,1	0,6	0,3583
ICY1	130,6	47,9	176,4	34,0	1,4	0,1700
ICY2	44,6	39,4	117,4	121,2	2,6	0,2969
IDH1	131,4	101,8	40,5	37,5	0,3	0,1446
IDH2	346,0	228,3	178,9	83,3	0,5	0,2182
IDI1	189,1	59,2	83,3	57,6	0,4	0,0429
IDP1	133,0	94,6	264,1	139,4	2,0	0,1708
IDP2	16,9	5,2	19,8	13,6	1,2	0,7075
IDP3	7,9	10,6	84,5	109,8	10,7	0,2139
IDS2	72,5	37,9	86,6	67,1	1,2	0,7273
IES1	1,2	2,4	4,2	6,8	3,5	0,4381
IES2	0,6	1,2	0,0	0,0	0,0	0,3559
IES3	10,1	5,0	6,2	7,5	0,6	0,4160
IFA38	348,6	14,4	387,7	130,4	1,1	0,5728
IFH1	165,1	125,4	285,7	256,8	1,7	0,4313
IFM1	8,2	7,1	15,5	11,7	1,9	0,3258
IGO1	26,6	29,6	30,1	33,9	1,1	0,8827
IGO2	22,7	22,8	18,6	19,8	0,8	0,7940
IKI1	26,9	5,7	9,5	11,0	0,4	0,0310
IKI3	98,2	62,7	80,0	55,8	0,8	0,6800
IKS1	32,2	28,5	55,9	32,0	1,7	0,3116

ILM1	241,0	101,1	64,9	44,1	0,3	0,0188
ILS1	113,2	81,2	84,2	69,7	0,7	0,6075
ILV1	207,1	83,0	407,1	493,3	2,0	0,4544
ILV2	783,4	233,6	828,5	88,0	1,1	0,7300
ILV3	105,2	76,2	105,6	112,9	1,0	0,9956
ILV5	846,9	475,5	461,2	324,4	0,5	0,2287
ILV6	247,8	108,6	496,3	240,0	2,0	0,1082
IMA1	3,3	2,3	0,7	1,3	0,2	0,0862
IMA2	0,0	0,0	0,0	0,0	#DIV/0!	#DIV/0!
IMA3	36,9	28,7	103,9	133,4	2,8	0,3645
IMA5	7,1	5,4	7,9	12,6	1,1	0,9163
IMD1	64,6	56,9	22,9	32,8	0,4	0,2505
IMD2	4,8	5,0	3,8	7,6	0,8	0,8370
IMD3	80,4	34,8	24,5	22,3	0,3	0,0355
IMD4	106,6	74,1	107,0	132,1	1,0	0,9952
IME1	42,7	19,4	46,2	53,1	1,1	0,9055
IME2	1,1	2,1	1,2	2,4	1,1	0,9442
IME4	282,8	72,7	274,0	224,6	1,0	0,9424
IMG1	42,3	19,3	23,0	18,6	0,5	0,1998
IMG2	2,5	5,0	2,2	2,5	0,9	0,9170
IMH1	131,5	109,2	161,0	109,5	1,2	0,7165
IML1	49,4	37,7	44,6	30,5	0,9	0,8504
IML2	3,5	2,5	68,5	120,0	19,4	0,3203
IML3	244,3	219,7	110,8	127,5	0,5	0,3338
IMP1	11,2	5,4	14,6	16,1	1,3	0,7048
IMP2'	9,9	3,0	89,8	90,5	9,0	0,1283
IMP2	6,8	3,2	3,8	5,0	0,6	0,3592
IMP3	32,2	6,4	69,9	74,3	2,2	0,3503
IMP4	37,2	26,2	20,7	14,3	0,6	0,3104
INH1	168,0	140,8	198,7	236,7	1,2	0,8308
INM1	165,4	88,6	267,7	153,2	1,6	0,2914
INM2	7,2	3,5	16,8	12,0	2,3	0,1764
INN1	44,0	32,4	37,6	34,7	0,9	0,7957
INO1	11,1	15,6	2,4	2,8	0,2	0,3162
INO2	1,1	2,1	1,0	2,0	0,9	0,9590
INO4	40,3	27,8	49,0	50,9	1,2	0,7746
INO80	79,4	42,0	91,0	28,2	1,1	0,6640
INP1	2,2	2,5	1,9	2,4	0,9	0,8809
INP2	45,3	17,1	16,4	12,3	0,4	0,0338
INP51	14,0	9,0	14,4	13,8	1,0	0,9589
INP52	5,3	4,7	9,2	8,3	1,7	0,4423
INP53	61,1	27,8	124,7	175,5	2,0	0,5007
INP54	11,4	10,2	12,1	9,4	1,1	0,9277
IOC2	273,2	84,4	281,8	130,0	1,0	0,9151
IOC3	55,8	19,4	44,8	31,8	0,8	0,5746
IOC4	67,2	35,8	60,2	42,9	0,9	0,8123
IPI1	8,5	9,9	4,9	4,4	0,6	0,5325
IPI3	35,8	17,6	18,1	16,8	0,5	0,1974
IPK1	24,8	15,2	10,9	9,2	0,4	0,1696
IPL1	7,2	5,5	0,0	0,0	0,0	0,0386

IPP1	1090,5	350,0	1264,9	356,4	1,2	0,5113
IPT1	163,5	162,6	140,1	94,1	0,9	0,8116
IQG1	8,3	4,7	13,4	9,3	1,6	0,3619
IRA1	14,3	6,3	31,8	26,3	2,2	0,2441
IRA2	109,9	23,1	138,6	63,2	1,3	0,4259
IRC10	8,4	8,4	2,3	4,6	0,3	0,2500
IRC11	9,9	13,1	66,8	44,7	6,7	0,0505
IRC15	286,7	316,7	876,4	915,2	3,1	0,2690
IRC18	12,4	9,5	22,0	20,1	1,8	0,4189
IRC19	148,9	121,7	27,4	23,6	0,2	0,0978
IRC20	25,6	8,4	26,6	17,8	1,0	0,9218
IRC21	0,0	0,0	0,7	1,3	#DIV/0!	0,3559
IRC22	234,7	168,4	263,9	212,9	1,1	0,8368
IRC23	49,3	24,9	164,2	113,2	3,3	0,0948
IRC24	12,2	10,5	3,7	3,2	0,3	0,1730
IRC3	189,4	149,0	95,5	141,1	0,5	0,3956
IRC4	11,5	22,1	8,6	17,2	0,7	0,8428
IRC5	60,4	37,2	39,0	23,0	0,6	0,3633
IRC6	165,8	78,3	410,8	221,0	2,5	0,0816
IRC7	26,9	18,1	27,2	24,4	1,0	0,9848
IRC8	168,7	101,1	103,8	102,5	0,6	0,4021
IRC9	14,4	4,4	16,6	11,9	1,2	0,7401
IRE1	75,7	6,8	72,6	53,4	1,0	0,9128
IRR1	95,0	54,8	69,1	75,3	0,7	0,5985
IRS4	21,7	10,4	26,4	24,6	1,2	0,7356
ISA1	239,3	50,7	163,0	163,8	0,7	0,4080
ISA2	0,3	0,6	0,0	0,0	0,0	0,3559
ISC1	39,3	26,2	24,0	21,4	0,6	0,4018
ISC10	0,0	0,0	6,4	6,0	#DIV/0!	0,0768
ISD11	131,7	57,1	150,3	37,4	1,1	0,6054
ISF1	41,8	21,1	134,8	90,2	3,2	0,0916
ISM1	28,0	15,4	26,3	17,8	0,9	0,8898
ISN1	28,0	20,3	33,5	32,3	1,2	0,7841
ISR1	11,7	2,6	4,2	5,6	0,4	0,0536
IST1	27,3	7,3	8,1	6,8	0,3	0,0084
IST2	87,1	66,7	127,0	87,2	1,5	0,4946
IST3	25,4	4,8	15,7	11,2	0,6	0,1604
ISU1	30,7	24,1	24,9	30,3	0,8	0,7730
ISU2	12,5	12,7	8,0	9,3	0,6	0,5876
ISW1	66,8	79,1	27,2	28,2	0,4	0,3826
ISW2	108,3	31,7	82,0	56,3	0,8	0,4471
ISY1	62,1	40,0	87,9	72,0	1,4	0,5537
ITC1	288,1	202,7	244,4	251,4	0,8	0,7954
ITR1	397,8	85,8	284,5	74,0	0,7	0,0925
ITR2	194,2	53,9	230,5	87,1	1,2	0,5042
ITT1	20,2	16,2	8,4	9,7	0,4	0,2573
IVY1	2,0	2,6	2,6	5,3	1,3	0,8253
IWR1	18,7	16,7	47,1	42,4	2,5	0,2600
IXR1	180,1	47,1	122,6	87,9	0,7	0,2925
IZH1	11,7	8,0	3,2	3,9	0,3	0,1080

IZH2	75,2	44,7	71,1	65,0	0,9	0,9215
IZH3	47,9	40,8	112,7	97,4	2,4	0,2656
IZH4	18,1	5,7	15,1	10,4	0,8	0,6315
JAC1	15,1	13,0	33,4	35,1	2,2	0,3667
JEM1	14,5	13,2	2,8	3,4	0,2	0,1371
JEN1	199,1	112,7	199,3	118,0	1,0	0,9980
JHD1	9,5	4,6	76,5	114,7	8,0	0,2874
JHD2	18,8	13,2	9,4	6,7	0,5	0,2528
JID1	10,5	9,7	6,5	5,9	0,6	0,5011
JIP4	139,8	73,0	147,7	86,7	1,1	0,8935
JIP5	402,2	229,2	530,7	173,0	1,3	0,4053
JJJ1	34,9	30,8	61,9	56,5	1,8	0,4341
JJJ2	39,8	2,9	47,7	32,0	1,2	0,6399
JJJ3	24,9	11,2	21,2	21,0	0,9	0,7674
JLP1	8,0	8,9	15,1	15,4	1,9	0,4573
JLP2	51,5	24,8	18,1	14,6	0,4	0,0591
JNM1	11,6	5,4	15,3	17,8	1,3	0,7008
JSN1	11,8	9,3	19,1	14,8	1,6	0,4357
KAE1	33,3	16,3	10,4	8,1	0,3	0,0457
KAP104	53,6	32,4	59,0	40,8	1,1	0,8436
KAP114	77,3	77,1	32,0	23,2	0,4	0,3030
KAP120	107,6	44,6	57,2	40,3	0,5	0,1442
KAP122	26,2	20,4	91,4	80,8	3,5	0,1688
KAP123	85,2	41,5	30,0	26,0	0,4	0,0654
KAP95	29,2	14,6	21,5	18,8	0,7	0,5406
KAR1	169,8	34,7	91,5	64,8	0,5	0,0769
KAR2	643,6	318,4	1094,2	486,8	1,7	0,1722
KAR3	44,4	22,0	27,9	23,1	0,6	0,3427
KAR4	22,8	10,8	131,6	107,6	5,8	0,0908
KAR5	38,9	24,0	232,9	207,5	6,0	0,1126
KAR9	8,6	8,5	16,6	11,8	1,9	0,3126
KCC4	33,1	29,3	3,4	4,0	0,1	0,0916
KCS1	29,8	17,6	299,7	164,2	10,0	0,0170
KDX1	6,0	4,9	4,3	5,0	0,7	0,6288
KEG1	9,0	6,0	1,3	2,5	0,1	0,0540
KEI1	76,8	58,5	50,4	38,1	0,7	0,4781
KEL1	52,5	11,2	97,7	137,5	1,9	0,5364
KEL2	40,5	28,1	180,2	294,1	4,4	0,3810
KEL3	109,1	112,6	103,7	79,8	1,0	0,9408
KEM1	155,8	40,0	77,9	54,9	0,5	0,0616
KES1	155,2	89,0	61,9	49,5	0,4	0,1164
KEX1	201,6	137,3	128,7	97,0	0,6	0,4189
KEX2	25,9	22,2	7,8	11,2	0,3	0,1956
KGD1	94,1	46,4	75,2	58,4	0,8	0,6319
KGD2	68,8	19,9	93,3	84,8	1,4	0,5939
KHA1	18,9	9,9	145,5	169,8	7,7	0,1869
KIC1	29,6	10,4	17,1	13,1	0,6	0,1842
KIN1	76,4	26,9	65,0	51,5	0,9	0,7103
KIN2	18,0	7,6	25,4	22,6	1,4	0,5588
KIN28	19,3	5,8	17,5	13,6	0,9	0,8081

KIN3	7,8	2,7	9,0	7,8	1,2	0,7788
KIN4	82,3	35,1	41,9	31,0	0,5	0,1343
KIN82	127,3	55,8	88,0	117,5	0,7	0,5686
KIP1	6,9	5,3	5,2	6,0	0,8	0,6858
KIP2	3,5	3,3	4,3	5,6	1,2	0,8129
KIP3	12,7	11,4	9,4	11,4	0,7	0,6932
KKQ8	20,6	10,5	18,4	12,6	0,9	0,7991
KNH1	25,8	4,2	42,4	36,2	1,6	0,3982
KNS1	15,7	7,0	13,7	11,6	0,9	0,7836
KOG1	7,4	3,7	10,7	8,3	1,4	0,4972
KRE1	51,8	30,8	37,6	30,2	0,7	0,5353
KRE11	62,0	28,7	135,1	87,6	2,2	0,1640
KRE2	29,3	15,4	64,2	54,0	2,2	0,2602
KRE27	56,0	33,1	39,4	26,5	0,7	0,4639
KRE28	30,5	19,9	18,4	19,3	0,6	0,4147
KRE33	26,7	16,5	22,3	25,4	0,8	0,7817
KRE5	55,0	12,4	91,7	62,9	1,7	0,2963
KRE6	165,5	66,9	57,4	48,4	0,3	0,0395
KRE9	66,3	32,2	131,9	120,2	2,0	0,3326
KRI1	864,8	713,4	1363,3	1059,5	1,6	0,4647
KRR1	104,2	49,3	99,2	87,6	1,0	0,9242
KRS1	633,5	368,2	949,3	809,6	1,5	0,5043
KSH1	50,1	30,7	27,6	27,6	0,6	0,3175
KSP1	75,2	26,8	88,0	62,2	1,2	0,7182
KSS1	58,0	65,0	44,3	53,8	0,8	0,7568
KTI12	10,6	6,5	40,6	37,3	3,8	0,1643
KTR1	45,4	19,2	15,1	13,4	0,3	0,0411
KTR2	146,6	84,3	65,0	43,9	0,4	0,1371
KTR3	110,4	75,8	89,7	64,4	0,8	0,6910
KTR4	63,1	11,8	59,6	40,8	0,9	0,8741
KTR5	7,1	3,5	3,0	5,9	0,4	0,2798
KTR6	68,9	23,6	45,7	34,5	0,7	0,3081
KTR7	30,6	17,1	120,5	159,2	3,9	0,3044
KXD1	117,4	33,6	85,2	80,3	0,7	0,4878
LAA1	94,4	59,9	66,7	33,6	0,7	0,4508
LAC1	43,1	20,4	22,2	16,8	0,5	0,1650
LAG1	140,5	68,9	78,8	54,4	0,6	0,2101
LAG2	2,5	3,0	3,2	3,9	1,2	0,8071
LAP2	236,5	147,4	148,9	142,3	0,6	0,4253
LAP3	166,9	103,3	478,9	243,1	2,9	0,0562
LAP4	227,7	223,9	651,2	467,7	2,9	0,1535
LAS1	40,1	15,0	23,0	17,9	0,6	0,1926
LAS17	83,0	35,6	64,8	59,1	0,8	0,6173
LAS21	114,2	28,9	213,4	163,5	1,9	0,2773
LAT1	285,5	196,3	309,7	253,1	1,1	0,8852
LCB1	88,1	25,2	68,1	48,2	0,8	0,4914
LCB2	588,2	445,1	420,1	218,3	0,7	0,5232
LCB3	62,1	53,9	25,6	27,0	0,4	0,2717
LCB4	0,0	0,0	2,4	4,7	#DIV/0!	0,3559
LCB5	28,8	16,9	23,5	16,8	0,8	0,6727

LCD1	25,1	3,5	109,1	194,2	4,3	0,4207
LCL1	23,9	11,9	34,9	33,8	1,5	0,5601
LCL2	1,5	3,0	0,0	0,0	0,0	0,3559
LCL3	7,0	2,3	13,5	10,6	1,9	0,2770
LCP5	22,1	15,2	25,8	18,2	1,2	0,7632
LDB16	24,9	7,0	25,3	20,1	1,0	0,9765
LDB17	77,7	29,5	52,2	37,3	0,7	0,3243
LDB18	16,7	6,8	16,0	11,0	1,0	0,9204
LDB19	55,9	15,1	52,6	35,2	0,9	0,8690
LEA1	56,6	29,6	41,4	27,7	0,7	0,4818
LEE1	99,8	78,7	91,1	119,6	0,9	0,9076
LEM3	68,7	51,0	37,8	26,8	0,6	0,3251
LEO1	343,1	174,3	183,8	153,3	0,5	0,2190
LEU1	338,1	156,3	227,4	194,6	0,7	0,4091
LEU3	40,2	32,1	157,5	218,0	3,9	0,3282
LEU4	58,9	42,9	39,5	39,9	0,7	0,5333
LEU5	36,7	25,6	88,4	162,2	2,4	0,5523
LEU9	41,5	35,7	21,2	14,5	0,5	0,3327
LGE1	83,7	60,1	48,2	36,0	0,6	0,3503
LHS1	90,2	6,2	100,5	53,8	1,1	0,7160
LIA1	79,0	40,3	67,1	67,5	0,8	0,7724
LIF1	8,2	11,2	20,0	19,5	2,4	0,3344
LIN1	86,7	39,4	120,3	43,0	1,4	0,2933
LIP2	1,7	3,4	4,9	5,9	2,9	0,3877
LIP5	367,7	213,9	58,1	40,1	0,2	0,0294
LOC1	31,6	21,3	4,7	3,4	0,1	0,0472
LOH1	9,4	6,5	7,1	5,8	0,8	0,6167
LOS1	29,3	14,3	99,5	118,0	3,4	0,2818
LOT5	29,6	14,5	15,7	13,5	0,5	0,2113
LOT6	13,0	10,1	12,3	15,1	0,9	0,9349
LPD1	151,1	85,0	195,7	172,7	1,3	0,6593
LPP1	15,0	6,3	14,4	10,0	1,0	0,9248
LPX1	8,5	9,2	3,7	3,2	0,4	0,3658
LRE1	10,4	12,3	13,0	9,9	1,3	0,7473
LRG1	6,5	7,1	5,6	7,2	0,9	0,8553
LRO1	33,0	13,9	21,8	16,3	0,7	0,3354
LRP1	197,9	112,8	221,4	169,2	1,1	0,8252
LRS4	0,5	0,9	0,0	0,0	0,0	0,3559
LSB3	124,9	138,3	112,9	120,2	0,9	0,8998
LSB5	17,8	18,4	7,9	9,3	0,4	0,3742
LSB6	25,5	15,9	207,8	367,8	8,2	0,3600
LSC1	41,4	21,9	21,6	14,8	0,5	0,1853
LSC2	53,7	16,9	24,0	19,4	0,4	0,0606
LSG1	49,7	46,7	32,7	28,2	0,7	0,5564
LSM1	417,6	185,4	577,2	115,6	1,4	0,1943
LSM12	70,0	45,2	36,3	32,2	0,5	0,2709
LSM2	287,8	98,8	372,0	209,1	1,3	0,4939
LSM6	145,5	39,4	108,9	74,1	0,7	0,4168
LSM7	285,6	78,8	917,3	791,1	3,2	0,1631
LSP1	175,0	103,6	333,6	186,7	1,9	0,1880

LST4	137,7	49,9	81,8	58,8	0,6	0,1972
LST7	31,8	31,2	18,3	13,1	0,6	0,4572
LST8	49,3	17,6	43,9	32,7	0,9	0,7820
LTE1	673,7	356,2	1142,5	515,6	1,7	0,1852
LTP1	109,3	69,9	46,6	34,1	0,4	0,1582
LTV1	171,5	56,3	228,5	50,9	1,3	0,1835
LYP1	222,0	115,8	221,7	172,0	1,0	0,9981
LYS1	277,3	43,3	152,4	111,2	0,5	0,0811
LYS12	615,7	367,2	471,3	212,7	0,8	0,5216
LYS14	281,5	62,5	288,6	202,6	1,0	0,9488
LYS2	97,7	40,5	44,8	41,0	0,5	0,1164
LYS20	98,0	81,1	33,2	33,9	0,3	0,1906
LYS21	800,3	432,4	429,4	391,0	0,5	0,2503
LYS4	57,6	50,2	41,0	43,3	0,7	0,6355
LYS5	581,3	381,7	163,8	174,2	0,3	0,0937
LYS9	1373,6	1270,6	556,4	546,8	0,4	0,2821
MAC1	42,2	11,0	22,3	15,2	0,5	0,0778
MAD1	146,0	100,5	133,1	91,1	0,9	0,8550
MAD2	20,3	14,0	26,5	18,5	1,3	0,6133
MAD3	23,4	15,3	23,1	16,9	1,0	0,9761
MAE1	79,1	53,7	49,0	39,3	0,6	0,3997
MAF1	17,9	2,8	18,3	12,6	1,0	0,9523
MAG1	1820,0	425,2	1251,9	589,8	0,7	0,1692
MAG2	18,7	8,1	18,8	16,7	1,0	0,9860
MAK10	6,7	3,8	0,0	0,0	0,0	0,0127
MAK11	38,2	14,9	40,5	30,5	1,1	0,8967
MAK16	80,5	25,4	71,2	63,6	0,9	0,7952
MAK21	244,5	132,4	225,9	163,3	0,9	0,8655
MAK31	18,1	11,4	23,0	24,4	1,3	0,7281
MAK32	4,7	5,6	4,5	7,3	1,0	0,9657
MAK5	183,9	89,7	219,9	147,2	1,2	0,6906
MAL11	2,0	4,0	8,0	7,1	4,1	0,1875
MAL12	8,0	6,9	27,9	31,2	3,5	0,2586
MAL13	8,2	9,6	63,4	43,2	7,8	0,0466
MAL31	2,1	4,3	1,2	2,4	0,6	0,7103
MAL32	19,9	14,5	27,5	23,0	1,4	0,5961
MAL33	18,8	5,2	99,9	73,7	5,3	0,0704
MAM1	1,1	2,3	0,0	0,0	0,0	0,3559
MAM3	17,1	12,5	25,8	18,6	1,5	0,4668
MAM33	152,3	90,2	333,3	341,5	2,2	0,3449
MAP1	199,5	53,1	406,1	415,0	2,0	0,3616
MAP2	241,6	134,3	284,0	68,9	1,2	0,5945
MAS1	32,1	20,2	17,4	12,1	0,5	0,2558
MAS2	28,2	21,5	11,2	10,6	0,4	0,2061
MBA1	11,4	11,2	13,5	12,8	1,2	0,8086
MBB1	5,8	7,3	1,8	2,3	0,3	0,3431
MBF1	0,0	0,0	0,0	0,0	#DIV/0!	#DIV/0!
MBP1	693,5	360,8	120,9	93,0	0,2	0,0218
MBR1	114,5	55,8	239,5	194,4	2,1	0,2626
MCA1	76,4	75,1	77,6	71,4	1,0	0,9818

MCD1	165,5	26,5	60,0	77,7	0,4	0,0423
MCD4	153,9	83,6	22,0	20,2	0,1	0,0220
MCH1	62,9	39,8	159,4	104,1	2,5	0,1340
MCH2	33,0	24,9	30,3	30,6	0,9	0,8972
MCH4	66,9	24,3	431,9	531,2	6,5	0,2189
MCH5	220,2	102,1	129,3	112,9	0,6	0,2776
MCK1	138,4	107,1	123,1	127,6	0,9	0,8598
MCM1	188,3	93,6	171,1	116,2	0,9	0,8254
MCM10	2,4	4,7	5,2	6,0	2,2	0,4864
MCM16	13,7	10,5	9,8	11,6	0,7	0,6434
MCM2	41,5	7,3	20,4	15,7	0,5	0,0498
MCM21	240,9	106,6	120,2	81,5	0,5	0,1223
MCM22	9,6	7,1	8,8	5,9	0,9	0,8639
MCM3	14,2	5,6	10,9	7,5	0,8	0,5067
MCM4	22,6	12,3	7,4	6,1	0,3	0,0682
MCM5	24,1	12,4	35,7	32,3	1,5	0,5292
MCM6	77,1	51,6	47,1	40,8	0,6	0,3969
MCM7	23,3	16,9	11,0	7,6	0,5	0,2313
MCR1	16,5	5,8	112,2	155,4	6,8	0,2643
MCT1	21,5	11,4	47,8	41,0	2,2	0,2627
MCX1	23,1	23,1	19,0	19,1	0,8	0,7961
MDE1	13,1	16,5	6,0	7,9	0,5	0,4730
MDG1	24,8	13,5	18,4	12,7	0,7	0,5205
MDH1	716,3	267,5	1139,5	430,6	1,6	0,1460
MDH2	302,8	88,7	578,1	168,6	1,9	0,0277
MDH3	56,2	50,9	32,3	30,4	0,6	0,4509
MDJ1	193,6	59,3	452,8	178,0	2,3	0,0327
MDJ2	16,7	6,0	25,8	17,9	1,5	0,3697
MDL1	27,7	33,3	30,4	28,5	1,1	0,9091
MDL2	114,9	73,8	89,6	62,2	0,8	0,6183
MDM1	54,9	15,4	26,7	18,6	0,5	0,0581
MDM10	0,0	0,0	0,0	0,0	#DIV/0!	#DIV/0!
MDM12	32,1	19,1	28,6	20,2	0,9	0,8135
MDM20	24,2	10,7	9,1	6,3	0,4	0,0502
MDM30	13,5	9,1	12,7	9,7	0,9	0,9081
MDM31	3,1	2,4	5,4	4,0	1,7	0,3686
MDM32	30,9	22,6	69,5	53,4	2,2	0,2313
MDM35	208,3	104,6	571,4	385,0	2,7	0,1185
MDM36	59,7	21,8	272,0	229,5	4,6	0,1151
MDM38	279,7	148,3	508,8	198,5	1,8	0,1139
MDN1	305,3	201,4	176,1	128,4	0,6	0,3208
MDR1	50,9	29,6	167,5	203,0	3,3	0,2990
MDS3	28,1	11,7	25,2	26,8	0,9	0,8489
MDV1	39,0	22,5	32,1	25,4	0,8	0,7012
MDY2	29,8	15,0	4,8	6,7	0,2	0,0225
MEC1	8,1	3,2	17,5	23,1	2,2	0,4519
MEC3	65,9	18,5	23,3	17,5	0,4	0,0156
MED1	9,4	7,4	20,2	14,4	2,1	0,2304
MED11	51,2	31,3	45,4	38,9	0,9	0,8247
MED2	31,1	29,3	39,1	38,7	1,3	0,7537

MED4	21,0	24,1	20,7	24,4	1,0	0,9876
MED6	8,3	10,5	9,2	10,2	1,1	0,9125
MED7	10,6	8,3	45,0	53,2	4,2	0,2491
MED8	8,0	5,5	7,1	5,8	0,9	0,8267
MEF1	16,8	10,9	14,8	15,7	0,9	0,8393
MEF2	4,4	3,4	12,7	12,7	2,9	0,2549
mei-04	9,3	7,7	73,4	98,5	7,9	0,2420
mei-05	1,5	1,8	0,7	1,3	0,4	0,4809
MEK1	30,0	28,1	10,7	12,5	0,4	0,2560
MEP1	83,4	50,1	121,3	132,9	1,5	0,6130
MEP2	46,2	32,9	11,3	7,9	0,2	0,0852
MEP3	303,8	13,1	453,7	232,3	1,5	0,2450
MER1	39,2	16,8	122,0	90,6	3,1	0,1226
MES1	861,4	214,9	1113,7	124,3	1,3	0,0884
MET1	7,8	4,7	27,1	27,3	3,5	0,2126
MET10	14,8	11,2	27,9	27,3	1,9	0,4089
MET12	34,6	19,3	21,7	22,0	0,6	0,4124
MET13	364,9	93,1	335,7	227,7	0,9	0,8205
MET14	4,6	4,0	11,3	12,1	2,5	0,3302
MET16	48,1	26,2	181,8	201,3	3,8	0,2358
MET18	47,9	10,3	25,0	20,1	0,5	0,0891
MET2	18,6	1,5	75,9	105,8	4,1	0,3203
MET22	22,5	14,3	30,0	30,9	1,3	0,6768
MET3	49,0	15,9	53,7	37,8	1,1	0,8260
MET30	14,9	14,5	14,6	16,0	1,0	0,9817
MET31	123,4	47,5	101,0	89,4	0,8	0,6730
MET32	269,0	138,3	144,0	119,3	0,5	0,2204
MET4	141,7	87,8	229,4	153,7	1,6	0,3603
MET5	29,8	12,1	145,8	122,2	4,9	0,1077
MET7	43,0	27,3	29,1	26,7	0,7	0,4927
MET8	3,3	4,5	38,6	48,4	11,5	0,1974
MEU1	24,5	13,7	13,2	14,1	0,5	0,2928
MEX67	27,0	5,1	22,1	20,0	0,8	0,6541
MF(ALPHA)1	1,2	2,4	1,2	2,4	1,0	0,9837
MF(ALPHA)2	229,9	126,6	288,2	145,0	1,3	0,5670
MFA1	527,2	286,3	2033,4	1220,9	3,9	0,0531
MFB1	107,0	80,5	59,7	47,5	0,6	0,3507
MFM1	3,3	4,1	6,3	6,4	1,9	0,4549
MFT1	62,7	40,3	129,9	84,7	2,1	0,2017
MGA1	3,7	2,6	8,7	6,5	2,4	0,1998
MGA2	55,3	16,9	18,8	15,5	0,3	0,0191
MGE1	272,9	89,6	127,3	30,7	0,5	0,0218
MGM1	11,8	7,1	5,7	4,4	0,5	0,1923
MGM101	111,8	87,5	26,7	18,1	0,2	0,1054
MGR1	20,6	8,7	12,7	10,1	0,6	0,2771
MGR2	236,6	100,6	405,4	269,3	1,7	0,2847
MGR3	42,9	16,0	28,7	22,6	0,7	0,3457
MGS1	44,2	3,1	24,3	21,2	0,5	0,1125
MGT1	150,1	128,3	221,5	331,2	1,5	0,7019
MHP1	342,1	21,7	294,0	178,2	0,9	0,6108

MHR1	77,9	12,0	60,2	43,2	0,8	0,4596
MHT1	3,7	3,0	103,3	170,5	27,9	0,2871
MIA40	89,7	53,9	109,6	73,2	1,2	0,6777
MIC14	20,2	16,3	15,9	18,4	0,8	0,7387
MIC17	153,6	73,8	210,0	212,0	1,4	0,6332
MID1	55,4	30,8	13,5	9,1	0,2	0,0404
MID2	9,9	4,6	24,4	25,8	2,5	0,3095
MIF2	72,9	38,8	40,1	29,6	0,5	0,2271
MIG1	43,0	17,0	32,8	26,4	0,8	0,5392
MIG2	123,7	77,5	127,2	97,8	1,0	0,9577
MIG3	87,6	76,5	273,0	330,6	3,1	0,3166
MIH1	0,0	0,0	1,2	2,4	#DIV/0!	0,3559
MIM1	31,8	18,0	174,1	262,3	5,5	0,3205
MIP1	37,4	33,2	35,4	28,2	0,9	0,9296
MIP6	10,3	4,4	71,9	108,6	7,0	0,3000
MIR1	809,8	424,1	747,2	534,7	0,9	0,8606
MIS1	137,2	25,2	150,9	112,1	1,1	0,8190
MKC7	56,9	3,1	95,3	105,0	1,7	0,4919
MKK1	29,7	10,0	27,4	21,8	0,9	0,8522
MKK2	69,4	33,7	54,6	59,9	0,8	0,6822
MKS1	35,8	23,3	116,4	134,3	3,2	0,2819
MKT1	134,2	109,9	54,0	55,8	0,4	0,2409
MLC1	62,2	51,7	210,2	118,4	3,4	0,0618
MLC2	30,2	19,2	23,4	24,3	0,8	0,6756
MLF3	31,7	18,1	36,9	29,5	1,2	0,7748
MLH1	129,4	37,1	83,9	48,0	0,6	0,1844
MLH2	58,2	20,6	90,2	79,2	1,5	0,4639
MLH3	7,6	6,3	16,1	12,9	2,1	0,2806
MLP1	147,9	118,6	97,7	71,1	0,7	0,4946
MLP2	352,0	268,1	392,4	296,9	1,1	0,8464
MLS1	25,5	21,1	33,6	27,4	1,3	0,6584
MMF1	270,9	46,4	314,2	55,6	1,2	0,2774
MMM1	1,1	2,3	10,1	9,9	8,9	0,1260
MMP1	17,1	2,5	33,5	27,8	2,0	0,2864
MMR1	43,1	20,0	95,5	120,5	2,2	0,4238
MMS1	37,4	9,1	65,9	54,2	1,8	0,3401
MMS2	45,3	23,4	81,8	93,8	1,8	0,4779
MMS21	2,8	2,1	6,9	5,5	2,5	0,2152
MMS22	1297,5	985,7	646,1	753,6	0,5	0,3342
MMS4	3,3	4,1	4,6	9,2	1,4	0,8048
MMT1	14,3	5,3	4,9	6,1	0,3	0,0607
MMT2	47,7	42,6	39,6	30,7	0,8	0,7685
MND1	2,6	3,1	1,9	2,4	0,7	0,7359
MND2	5,2	5,9	3,0	3,6	0,6	0,5631
MNE1	6,1	1,7	6,3	7,9	1,0	0,9750
MNL1	39,2	7,6	42,4	57,9	1,1	0,9160
MNN1	66,4	51,1	10,8	8,8	0,2	0,0755
MNN10	67,5	22,8	19,8	14,7	0,3	0,0125
MNN11	31,5	24,7	13,2	11,2	0,4	0,2285
MNN2	134,0	135,3	92,2	104,8	0,7	0,6422

MNN4	187,9	98,0	100,0	68,6	0,5	0,1924
MNN5	233,3	96,8	149,6	147,6	0,6	0,3794
MNN9	131,4	93,8	48,4	46,4	0,4	0,1638
MNP1	161,6	116,5	142,5	113,9	0,9	0,8224
MNR2	84,8	41,6	63,2	42,6	0,7	0,4958
MNS1	10,4	3,6	38,6	35,5	3,7	0,1647
MNT2	33,9	16,9	74,6	50,2	2,2	0,1752
MNT3	61,0	32,0	54,3	40,3	0,9	0,8034
MNT4	25,3	15,7	11,0	9,0	0,4	0,1657
MOB1	55,2	56,2	24,7	19,4	0,4	0,3452
MOB2	19,3	16,8	17,5	11,8	0,9	0,8717
MOD5	127,6	58,0	157,7	103,0	1,2	0,6291
MOG1	15,3	13,1	16,1	14,9	1,1	0,9347
MON1	1227,4	451,2	1658,4	418,9	1,4	0,2111
MON2	31,8	9,0	34,6	24,5	1,1	0,8371
MOT1	6,4	3,6	5,1	4,3	0,8	0,6577
MOT2	282,4	37,2	158,9	110,1	0,6	0,0778
MOT3	47,6	50,6	39,6	44,6	0,8	0,8205
MPA43	14,0	15,6	95,8	120,3	6,9	0,2258
MPC54	47,8	16,9	22,4	18,9	0,5	0,0920
MPD1	122,9	69,4	51,6	40,7	0,4	0,1268
MPD2	68,8	6,1	49,5	36,1	0,7	0,3329
MPE1	39,0	26,8	53,6	46,3	1,4	0,6051
MPH1	66,0	23,8	117,1	106,9	1,8	0,3868
MPH2	57,9	25,8	272,8	237,4	4,7	0,1219
MPM1	11,5	4,6	15,9	10,8	1,4	0,4766
MPP10	70,3	18,7	127,6	136,5	1,8	0,4373
MPP6	24,2	13,5	44,5	44,9	1,8	0,4201
MPS1	94,3	44,2	21,3	17,5	0,2	0,0218
MPS2	4,9	6,5	6,5	8,0	1,3	0,7768
MPS3	72,6	16,6	31,9	30,7	0,4	0,0589
MPT5	22,7	4,5	31,6	24,3	1,4	0,4998
MRC1	155,2	141,4	73,1	63,4	0,5	0,3303
MRD1	83,3	57,8	119,8	84,8	1,4	0,5039
MRE11	173,3	96,9	126,3	116,5	0,7	0,5575
MRF1	13,7	4,1	18,4	14,4	1,3	0,5575
MRH1	656,2	322,4	2494,9	2084,7	3,8	0,1319
MRH4	31,7	22,7	162,8	287,2	5,1	0,3979
MRI1	77,9	30,8	262,0	287,8	3,4	0,2504
MRK1	160,6	138,3	251,9	274,2	1,6	0,5736
MRL1	21,4	7,9	24,5	19,7	1,1	0,7846
MRM1	12,5	7,7	8,7	11,9	0,7	0,6072
MRM2	15,1	1,1	5,4	4,9	0,4	0,0081
MRN1	284,0	156,9	341,2	252,7	1,2	0,7136
MRP1	56,4	26,3	20,1	13,9	0,4	0,0507
MRP13	29,3	22,7	16,4	14,1	0,6	0,3714
MRP17	164,2	40,7	263,4	122,7	1,6	0,1755
MRP2	1169,6	435,1	221,3	158,7	0,2	0,0064
MRP20	39,0	16,9	19,8	13,5	0,5	0,1268
MRP21	30,4	17,9	17,3	14,5	0,6	0,2977

MRP4	4,4	3,4	9,8	11,6	2,2	0,4051
MRP49	32,4	11,3	40,9	35,0	1,3	0,6609
MRP51	228,7	141,7	223,6	106,6	1,0	0,9563
MRP7	400,1	587,4	485,4	771,7	1,2	0,8663
MRPL1	38,3	22,4	31,6	28,4	0,8	0,7242
MRPL10	20,4	17,2	11,7	9,5	0,6	0,4123
MRPL11	155,0	91,9	218,7	136,5	1,4	0,4681
MRPL13	7,0	6,6	6,2	8,4	0,9	0,8896
MRPL15	16,8	9,0	12,0	8,2	0,7	0,4574
MRPL16	60,9	24,6	67,6	53,0	1,1	0,8249
MRPL17	19,0	6,1	22,5	18,9	1,2	0,7347
MRPL20	18,1	13,1	8,1	10,8	0,4	0,2831
MRPL22	54,8	34,4	34,2	33,2	0,6	0,4222
MRPL23	155,4	77,1	106,4	86,7	0,7	0,4302
MRPL25	24,1	10,8	55,1	43,0	2,3	0,2115
MRPL27	22,4	8,9	13,4	10,6	0,6	0,2446
MRPL28	56,8	27,8	54,6	39,5	1,0	0,9325
MRPL3	20,3	13,9	24,6	28,5	1,2	0,7971
MRPL31	80,0	13,2	41,0	28,6	0,5	0,0481
MRPL32	28,7	18,6	17,0	14,8	0,6	0,3629
MRPL33	62,3	31,2	41,3	32,7	0,7	0,3891
MRPL35	27,3	25,2	19,7	22,1	0,7	0,6700
MRPL37	127,9	37,3	53,4	36,3	0,4	0,0288
MRPL38	9,4	2,1	3,5	4,4	0,4	0,0538
MRPL39	75,2	33,2	52,9	39,0	0,7	0,4170
MRPL4	21,9	10,5	17,4	13,7	0,8	0,6210
MRPL40	42,6	46,2	36,4	38,0	0,9	0,8426
MRPL44	379,1	188,7	479,3	191,0	1,3	0,4835
MRPL49	88,2	27,4	123,5	105,3	1,4	0,5415
MRPL50	105,3	43,4	82,6	60,0	0,8	0,5629
MRPL6	72,2	41,4	126,4	59,6	1,8	0,1858
MRPL7	259,5	90,3	173,4	161,3	0,7	0,3876
MRPL8	35,0	22,9	34,2	25,5	1,0	0,9662
MRPL9	0,6	1,2	1,9	3,8	3,1	0,5421
MRPS12	42,7	21,0	30,6	26,4	0,7	0,4998
MRPS16	40,3	20,8	53,8	48,2	1,3	0,6249
MRPS17	160,2	46,6	93,4	63,8	0,6	0,1422
MRPS18	84,9	43,9	90,8	69,3	1,1	0,8916
MRPS28	25,5	11,0	20,9	16,3	0,8	0,6566
MRPS35	28,7	10,6	33,8	29,1	1,2	0,7559
MRPS5	16,5	11,5	16,9	16,1	1,0	0,9720
MRPS8	35,8	8,4	18,6	12,7	0,5	0,0640
MRPS9	97,9	53,8	108,3	89,4	1,1	0,8486
MRS1	49,2	28,2	119,3	98,6	2,4	0,2207
MRS2	1257,1	975,5	123,3	83,8	0,1	0,0598
MRS3	41,7	11,5	17,1	12,1	0,4	0,0254
MRS4	8,3	7,7	11,2	12,1	1,3	0,6993
MRS6	215,9	31,2	309,2	98,1	1,4	0,1200
MRT4	838,4	266,7	308,2	244,9	0,4	0,0263
MSA1	48,5	17,2	85,9	70,5	1,8	0,3434

MSA2	0,6	1,2	2,6	3,6	4,4	0,3360
MSB1	14,3	3,6	10,8	8,7	0,8	0,4914
MSB2	182,2	119,6	64,0	56,6	0,4	0,1241
MSB3	4,4	4,9	10,8	10,3	2,4	0,3079
MSB4	33,8	23,7	10,1	7,6	0,3	0,1057
MSC1	8,5	7,4	17,4	19,3	2,1	0,4218
MSC2	34,8	14,3	35,2	26,7	1,0	0,9806
MSC3	26,0	18,9	46,7	42,3	1,8	0,4058
MSC6	60,8	21,9	15,7	10,6	0,3	0,0100
MSC7	27,6	19,1	15,7	14,7	0,6	0,3620
MSD1	6,3	6,1	2,4	2,8	0,4	0,2996
MSE1	35,0	11,2	27,3	21,8	0,8	0,5488
MSF1	27,4	10,2	26,4	17,7	1,0	0,9215
MSG5	42,8	15,2	28,6	22,5	0,7	0,3357
MSH1	33,6	17,9	205,6	359,8	6,1	0,3763
MSH2	25,3	8,4	6,2	7,3	0,2	0,0140
MSH3	40,0	10,5	117,2	154,0	2,9	0,3557
MSH4	23,0	21,3	92,5	159,5	4,0	0,4209
MSH5	9,4	6,7	12,4	9,0	1,3	0,6033
MSH6	20,3	9,7	5,3	3,6	0,3	0,0271
MSI1	21,3	11,9	98,2	110,7	4,6	0,2167
MSK1	20,8	13,9	21,9	23,6	1,1	0,9367
MSL1	22,8	9,7	30,2	25,6	1,3	0,6083
MSL5	10,8	7,3	5,3	10,7	0,5	0,4315
MSM1	397,4	182,5	218,2	118,0	0,5	0,1502
MSN1	15,1	6,7	9,3	7,0	0,6	0,2745
MSN2	143,8	179,7	28,3	21,0	0,2	0,2489
MSN4	36,3	14,0	52,7	36,9	1,4	0,4395
MSN5	165,6	48,5	249,0	40,4	1,5	0,0384
MSO1	16,4	6,8	4,3	5,6	0,3	0,0325
MSP1	18,3	11,2	27,3	20,8	1,5	0,4737
MSR1	19,5	8,5	96,8	113,7	5,0	0,2240
MSS1	85,8	84,4	60,2	53,7	0,7	0,6275
MSS11	13,3	6,1	177,9	213,0	13,3	0,1734
MSS116	35,3	15,5	43,2	44,9	1,2	0,7506
MSS18	91,6	68,1	28,2	22,2	0,3	0,1269
MSS2	205,9	63,4	190,6	105,8	0,9	0,8121
MSS4	39,0	16,5	37,2	32,3	1,0	0,9249
MSS51	19,4	19,2	12,2	14,6	0,6	0,5695
MST1	10,1	7,2	13,5	9,1	1,3	0,5750
MST27	183,3	83,2	344,2	270,4	1,9	0,2985
MST28	54,4	23,9	122,0	82,5	2,2	0,1665
MSW1	58,8	27,3	44,1	32,9	0,7	0,5162
MSY1	0,0	0,0	4,7	3,4	#DIV/0!	0,0308
MTC1	275,7	203,8	432,4	376,1	1,6	0,4914
MTC2	35,1	7,9	25,6	18,8	0,7	0,3843
MTC4	7,8	8,9	11,5	10,9	1,5	0,6184
MTC5	83,7	15,3	50,1	37,0	0,6	0,1442
MTC6	10,4	3,6	17,5	22,6	1,7	0,5551
MTC7	5,1	6,9	2,3	2,6	0,4	0,4671

MTD1	360,5	243,5	87,7	67,0	0,2	0,0740
MTF1	14,8	7,6	13,0	12,7	0,9	0,8217
MTF2	34,9	12,0	45,9	49,0	1,3	0,6770
MTG1	49,7	17,2	84,4	110,0	1,7	0,5558
MTG2	33,4	13,0	13,2	14,2	0,4	0,0809
MTH1	22,2	4,6	42,4	35,2	1,9	0,2988
MTL1	4,5	9,1	73,5	126,0	16,2	0,3166
MTM1	41,9	13,8	28,1	19,9	0,7	0,2990
MTO1	9,5	4,6	7,5	11,9	0,8	0,7706
MTQ1	11,7	10,7	3,2	3,9	0,3	0,1879
MTQ2	41,9	10,9	23,9	18,1	0,6	0,1391
MTR10	7,6	7,7	12,9	13,4	1,7	0,5254
MTR2	107,4	52,7	69,6	47,8	0,6	0,3283
MTR3	6,4	5,9	2,2	2,5	0,3	0,2287
MTR4	78,7	18,8	45,7	32,3	0,6	0,1278
MUB1	37,3	29,6	152,7	184,5	4,1	0,2631
MUC1	3,9	3,3	0,0	0,0	0,0	0,0567
MUD1	38,0	15,9	219,0	341,7	5,8	0,3308
MUD2	42,4	27,2	134,1	98,4	3,2	0,1223
MUK1	8,1	7,5	4,3	5,0	0,5	0,4270
MUM2	74,2	26,3	125,7	110,1	1,7	0,3984
MUM3	137,4	79,8	98,0	104,2	0,7	0,5706
MUP1	72,8	39,1	132,3	45,2	1,8	0,0931
MUP3	10,2	8,0	11,3	8,5	1,1	0,8584
MUS81	14,8	6,2	13,0	11,5	0,9	0,7960
MVB12	39,8	20,4	26,3	27,7	0,7	0,4592
MVD1	47,0	41,5	53,0	52,5	1,1	0,8656
MVP1	12,8	3,8	22,2	16,1	1,7	0,3011
MXR1	408,5	226,6	258,3	209,5	0,6	0,3681
MYO1	120,7	80,1	122,5	88,4	1,0	0,9767
MYO2	366,4	178,1	147,7	114,4	0,4	0,0843
MYO3	51,7	38,8	70,2	84,3	1,4	0,7030
MYO4	46,6	59,7	16,2	11,6	0,3	0,3565
MYO5	31,2	19,2	16,7	13,2	0,5	0,2594
MZM1	73,0	47,5	290,7	251,0	4,0	0,1393
NAB2	72,5	21,7	81,9	58,2	1,1	0,7731
NAB3	112,2	94,9	138,7	106,2	1,2	0,7222
NAB6	22,7	10,8	51,0	40,9	2,2	0,2299
NAF1	24,5	13,4	31,7	31,5	1,3	0,6902
NAM2	6,9	8,4	1,9	2,4	0,3	0,2980
NAM7	35,9	11,4	41,7	31,8	1,2	0,7444
NAM8	130,5	48,6	247,7	185,4	1,9	0,2674
NAM9	31,5	21,4	26,8	31,6	0,9	0,8135
NAN1	61,0	36,9	196,7	220,9	3,2	0,2713
NAP1	4,9	5,9	37,7	66,9	7,7	0,3662
NAR1	25,1	9,8	22,7	16,4	0,9	0,8101
NAS2	11,4	1,7	6,1	7,2	0,5	0,2006
NAS6	41,8	40,4	18,1	16,6	0,4	0,3192
NAT1	175,0	22,6	357,2	229,3	2,0	0,1649
NAT2	499,9	291,2	714,0	480,2	1,4	0,4747

NAT3	7,6	8,8	7,9	8,5	1,0	0,9618
NAT4	7,5	2,9	5,7	7,5	0,8	0,6769
NAT5	77,3	46,2	30,0	23,3	0,4	0,1173
NBA1	71,8	57,8	66,4	46,7	0,9	0,8901
NBP1	17,3	6,9	4,3	5,1	0,3	0,0231
NBP2	11,9	11,3	12,9	15,4	1,1	0,9155
NBP35	21,9	6,2	20,9	17,2	1,0	0,9241
NCA2	15,7	13,4	13,8	9,3	0,9	0,8299
NCA3	38,4	15,6	23,9	17,4	0,6	0,2602
NCE101	215,0	237,4	295,3	252,9	1,4	0,6598
NCE102	1220,6	219,6	2713,3	782,8	2,2	0,0104
NCE103	68,8	37,5	54,1	51,3	0,8	0,6608
NCL1	200,4	120,4	166,5	112,4	0,8	0,6950
NCP1	43,7	37,9	79,1	96,3	1,8	0,5188
NCR1	101,7	92,6	90,0	31,7	0,9	0,8198
NCS2	32,2	18,7	14,9	10,9	0,5	0,1623
NCS6	36,9	18,1	93,7	97,9	2,5	0,2974
NDC1	60,6	36,7	38,9	27,7	0,6	0,3827
NDD1	8,2	7,8	4,9	5,7	0,6	0,5211
NDE1	128,7	89,2	21,3	18,6	0,2	0,0564
NDE2	9,9	5,4	82,3	120,3	8,3	0,2743
NDI1	181,8	118,7	662,4	654,5	3,6	0,1986
NDJ1	5,8	1,8	1,8	3,6	0,3	0,0878
NDT80	120,9	97,6	99,2	145,7	0,8	0,8126
NEJ1	58,8	12,0	54,6	36,4	0,9	0,8337
NEM1	23,1	10,3	15,7	13,5	0,7	0,4142
NEO1	40,2	12,1	18,2	14,6	0,5	0,0589
NET1	12,8	7,4	5,0	3,5	0,4	0,1069
NEW1	33,2	13,6	25,9	24,2	0,8	0,6148
NFI1	57,1	8,3	56,1	63,2	1,0	0,9750
NFS1	325,3	104,8	593,6	340,3	1,8	0,1825
NFT1	9,7	7,4	9,3	7,0	1,0	0,9373
NFU1	16,7	4,5	21,5	15,0	1,3	0,5643
NGG1	114,7	67,9	81,9	57,7	0,7	0,4894
NGL1	2,9	3,5	14,8	16,3	5,1	0,2019
NGL2	31,3	16,6	82,3	57,3	2,6	0,1380
NGL3	1,9	2,2	34,5	27,6	18,1	0,0564
NGR1	101,5	68,3	50,9	37,2	0,5	0,2405
NHA1	119,2	95,5	78,0	63,4	0,7	0,4994
NHP2	0,0	0,0	0,0	0,0	#DIV/0!	#DIV/0!
NHP6A	379,3	265,4	430,7	57,2	1,1	0,7181
NHP6B	3,1	3,7	7,2	5,1	2,3	0,2464
NHX1	24,9	16,5	49,6	34,6	2,0	0,2461
NIC96	19,8	10,5	11,6	10,5	0,6	0,3082
NIF3	53,3	24,6	49,6	50,8	0,9	0,8988
NIP1	38,5	25,8	32,8	30,2	0,9	0,7861
NIP100	44,0	26,7	12,1	12,4	0,3	0,0732
NIP7	104,6	46,4	59,9	55,1	0,6	0,2609
NIS1	89,1	48,6	113,8	120,3	1,3	0,7162
NIT1	10,8	9,9	11,7	13,6	1,1	0,9182

NIT2	14,4	11,8	14,7	17,2	1,0	0,9722
NIT3	16,5	9,3	11,0	11,3	0,7	0,4880
NKP1	53,4	43,0	50,9	48,6	1,0	0,9404
NMA1	85,7	28,9	56,8	38,9	0,7	0,2773
NMA111	83,8	32,6	94,5	78,9	1,1	0,8108
NMA2	38,4	13,5	89,5	62,1	2,3	0,1589
NMD2	17,4	15,9	26,3	19,9	1,5	0,5087
NMD3	279,2	115,6	177,6	43,2	0,6	0,1509
NMD4	34,9	10,9	34,3	28,4	1,0	0,9687
NMD5	29,0	21,9	30,7	25,6	1,1	0,9233
NMT1	34,8	20,3	30,4	20,4	0,9	0,7699
NNF1	16,4	16,3	7,9	6,3	0,5	0,3660
NNF2	72,5	30,0	50,3	37,9	0,7	0,3929
NNK1	20,3	5,6	50,8	34,3	2,5	0,1299
NOB1	205,1	71,1	159,3	151,7	0,8	0,6046
NOC2	486,7	260,4	301,0	268,5	0,6	0,3592
NOC3	6,9	4,2	17,3	21,9	2,5	0,3880
NOC4	58,5	19,8	45,8	37,0	0,8	0,5668
NOG1	95,8	19,6	143,5	82,0	1,5	0,3005
NOG2	37,9	28,9	67,1	76,8	1,8	0,5032
NOP1	282,1	119,9	198,8	161,6	0,7	0,4395
NOP12	296,0	132,6	92,9	76,5	0,3	0,0378
NOP13	94,6	55,2	86,9	74,9	0,9	0,8743
NOP14	121,7	45,8	294,6	276,4	2,4	0,2632
NOP15	200,8	24,4	297,6	210,9	1,5	0,3968
NOP2	141,7	105,6	149,8	161,9	1,1	0,9357
NOP4	169,0	85,5	250,1	60,6	1,5	0,1723
NOP53	776,2	504,0	601,7	232,5	0,8	0,5526
NOP56	121,2	90,7	196,2	169,1	1,6	0,4639
NOP58	707,1	499,4	832,9	675,9	1,2	0,7748
NOP6	582,7	268,3	687,7	604,0	1,2	0,7614
NOP7	908,4	488,5	1205,1	580,7	1,3	0,4640
NOP8	83,5	67,2	86,1	75,3	1,0	0,9611
NOP9	45,8	26,5	27,2	27,5	0,6	0,3694
NOT3	29,2	25,5	27,4	26,6	0,9	0,9245
NOT5	145,9	35,2	66,2	46,8	0,5	0,0346
NPA3	93,2	58,4	71,9	61,9	0,8	0,6343
NPC2	109,0	25,8	121,7	86,1	1,1	0,7864
NPL3	192,7	164,8	986,5	819,2	5,1	0,1062
NPL4	20,4	10,4	41,2	27,9	2,0	0,2121
NPL6	55,2	28,9	31,3	25,0	0,6	0,2571
NPP1	15,0	11,3	24,4	22,6	1,6	0,4889
NPP2	22,1	10,3	7,4	8,7	0,3	0,0729
NPR1	46,5	15,3	30,4	20,3	0,7	0,2516
NPR2	11,7	7,0	87,8	153,4	7,5	0,3597
NPR3	90,4	29,6	103,7	121,8	1,1	0,8385
NPT1	218,4	244,1	120,8	97,1	0,6	0,4855
NPY1	124,1	48,2	67,9	62,9	0,5	0,2059
NQM1	6,5	5,3	272,2	435,1	41,8	0,2678
NRD1	40,7	38,3	47,9	57,5	1,2	0,8431

NRG1	96,2	65,2	51,9	37,8	0,5	0,2845
NRG2	38,0	12,0	43,6	31,2	1,1	0,7500
NRK1	64,0	40,0	51,4	39,5	0,8	0,6710
NRM1	9,9	4,3	1,0	2,0	0,1	0,0096
NRP1	15,8	12,1	23,1	17,3	1,5	0,5177
NRT1	46,1	33,6	48,9	42,2	1,1	0,9215
NSA1	33,4	15,8	27,7	31,6	0,8	0,7549
NSA2	51,1	37,5	84,1	79,5	1,6	0,4819
NSE1	577,8	319,8	295,1	331,1	0,5	0,2655
NSE3	32,7	10,2	6,7	5,5	0,2	0,0041
NSE4	17,4	8,4	9,8	11,5	0,6	0,3255
NSE5	59,8	12,9	49,2	35,1	0,8	0,5896
NSG1	339,0	81,8	409,3	509,4	1,2	0,7944
NSG2	71,5	37,6	89,4	51,3	1,2	0,5955
NSL1	61,2	39,4	65,0	77,1	1,1	0,9337
NSP1	2,9	5,8	2,3	4,6	0,8	0,8790
NSR1	26,2	15,3	19,2	15,6	0,7	0,5435
NST1	458,9	403,2	498,0	409,9	1,1	0,8963
NTA1	15,4	18,4	12,1	8,7	0,8	0,7566
NTE1	30,7	18,4	12,0	10,4	0,4	0,1271
NTF2	178,8	84,8	244,1	29,5	1,4	0,1959
NTG1	41,8	24,0	25,3	17,6	0,6	0,3085
NTG2	39,5	38,5	54,0	55,8	1,4	0,6834
NTH1	68,7	45,2	164,7	46,2	2,4	0,0249
NTH2	202,5	54,2	164,0	29,8	0,8	0,2601
NTO1	43,3	33,9	13,9	10,8	0,3	0,1493
NUC1	29,6	29,0	132,1	215,8	4,5	0,3827
NUD1	16,0	15,5	6,7	7,3	0,4	0,3217
NUF2	0,0	0,0	0,0	0,0	#DIV/0!	#DIV/0!
NUG1	74,4	45,5	68,8	46,4	0,9	0,8686
NUM1	33,0	27,6	31,6	24,1	1,0	0,9423
NUP1	24,2	6,1	25,9	20,8	1,1	0,8852
NUP100	35,0	20,5	89,5	106,2	2,6	0,3522
NUP116	185,0	77,2	311,3	382,7	1,7	0,5416
NUP120	3186,4	1374,0	1252,8	295,4	0,4	0,0332
NUP133	13,0	11,9	6,0	7,9	0,5	0,3703
NUP145	61,5	12,9	24,0	18,5	0,4	0,0161
NUP157	113,0	67,3	88,9	81,6	0,8	0,6658
NUP159	32,4	17,1	39,2	28,1	1,2	0,6947
NUP170	38,2	10,3	27,2	21,6	0,7	0,3940
NUP188	41,0	25,2	149,9	168,2	3,7	0,2475
NUP192	91,0	60,7	90,1	68,7	1,0	0,9843
NUP2	148,6	131,5	122,2	105,8	0,8	0,7653
NUP49	10,7	12,5	6,5	5,5	0,6	0,5561
NUP53	86,0	46,6	24,8	22,9	0,3	0,0566
NUP57	205,2	95,4	127,5	91,4	0,6	0,2838
NUP60	22,1	15,0	22,1	16,5	1,0	0,9974
NUP82	77,9	30,9	92,2	63,1	1,2	0,6983
NUP84	18,7	20,9	13,9	17,3	0,7	0,7363
NUP85	65,1	19,2	178,5	249,9	2,7	0,4004

NUR1	3,1	3,9	6,0	4,7	1,9	0,3727
NUS1	13,3	9,8	15,9	19,4	1,2	0,8162
NUT1	203,6	97,1	86,8	65,9	0,4	0,0938
NUT2	2,7	4,0	9,6	7,7	3,5	0,1675
NVJ1	12,3	8,5	13,8	13,9	1,1	0,8581
NYV1	109,6	39,2	230,7	20,6	2,1	0,0016
OAC1	34,1	20,7	35,6	33,6	1,0	0,9422
OAF1	18,5	14,6	14,2	11,6	0,8	0,6599
OAF3	20,4	10,1	23,1	19,0	1,1	0,8083
OAR1	14,4	7,8	24,3	16,8	1,7	0,3276
OAZ1	26,2	10,9	15,4	12,0	0,6	0,2324
OCA1	53,6	21,5	82,1	74,6	1,5	0,4911
OCA2	0,0	0,0	0,0	0,0	#DIV/0!	#DIV/0!
OCA4	392,3	212,6	284,1	121,3	0,7	0,4106
OCA5	10,1	5,4	24,6	21,5	2,4	0,2376
OCA6	89,6	9,3	124,9	73,6	1,4	0,3783
OCH1	60,6	10,8	24,1	19,1	0,4	0,0159
OCT1	38,9	19,2	28,8	25,0	0,7	0,5468
ODC1	257,7	86,5	131,1	99,7	0,5	0,1034
ODC2	446,5	146,1	1663,9	509,2	3,7	0,0037
OGG1	9,1	10,5	2,4	2,8	0,3	0,2667
OKP1	57,3	16,9	44,5	29,7	0,8	0,4813
OLA1	1019,7	304,7	2087,2	988,7	2,0	0,0846
OLE1	107,1	56,5	109,2	148,8	1,0	0,9793
OM45	133,0	46,4	457,1	256,1	3,4	0,0471
OMA1	41,8	20,7	5,2	6,4	0,1	0,0148
OMS1	36,7	28,4	61,1	41,7	1,7	0,3711
OPI1	51,3	22,8	40,3	33,5	0,8	0,6090
OPI10	11,3	13,5	35,9	34,4	3,2	0,2305
OPI3	63,1	19,5	153,7	139,4	2,4	0,2455
OPT1	36,9	5,3	48,6	32,8	1,3	0,5076
OPT2	181,6	68,5	42,7	30,8	0,2	0,0101
OPY1	6,5	4,1	5,7	5,5	0,9	0,8237
OPY2	20,4	18,5	10,2	14,5	0,5	0,4197
ORC1	152,4	64,1	46,8	32,8	0,3	0,0262
ORC2	24,2	15,6	92,1	122,8	3,8	0,3149
ORC3	20,7	13,7	13,8	12,6	0,7	0,4886
ORC4	37,5	13,1	97,4	111,0	2,6	0,3251
ORC5	218,8	66,2	105,5	82,6	0,5	0,0761
ORC6	4,7	6,2	7,4	8,9	1,6	0,6329
ORM1	51,7	26,1	14,1	10,5	0,3	0,0367
ORM2	35,4	22,7	93,9	79,8	2,6	0,2083
ORT1	2,8	3,4	2,4	4,7	0,8	0,8838
OSH2	148,2	79,2	101,4	67,8	0,7	0,4041
OSH3	8,0	5,8	14,4	11,5	1,8	0,3632
OSH6	47,4	19,8	29,3	20,0	0,6	0,2481
OSH7	22,2	12,4	31,5	23,6	1,4	0,5149
OSM1	58,0	18,2	68,0	52,2	1,2	0,7290
OST1	290,9	104,3	110,6	94,6	0,4	0,0428
OST3	24,2	21,6	21,7	22,3	0,9	0,8776

OST4	182,3	143,2	224,2	229,4	1,2	0,7671
OST6	156,9	61,6	66,5	45,0	0,4	0,0556
OSW1	0,8	1,5	0,7	1,3	0,9	0,9221
OSW2	38,4	45,4	117,7	134,1	3,1	0,3054
OSW5	99,6	55,0	119,8	49,9	1,2	0,6054
OTU1	13,2	3,6	29,7	22,9	2,3	0,2042
OTU2	17,7	13,8	5,6	6,7	0,3	0,1677
OXA1	64,6	49,2	47,3	47,5	0,7	0,6301
OXP1	14,1	13,1	1,8	3,6	0,1	0,1187
OXR1	100,6	27,1	136,7	123,2	1,4	0,5880
OYE2	88,2	69,3	107,8	118,5	1,2	0,7857
OYE3	2,7	3,4	2,3	4,6	0,9	0,9015
PAA1	84,8	53,8	116,5	112,2	1,4	0,6289
PAB1	272,8	158,9	422,8	182,8	1,5	0,2617
PAC1	2,7	3,7	7,0	6,4	2,5	0,2974
PAC11	11,3	12,7	1,3	2,5	0,1	0,1694
PAC2	90,5	28,1	60,0	43,9	0,7	0,2856
PAD1	97,8	40,2	166,7	128,1	1,7	0,3439
PAF1	102,1	71,8	159,3	115,0	1,6	0,4316
PAH1	23,9	7,9	126,5	130,4	5,3	0,1672
PAM1	92,1	51,5	54,3	39,6	0,6	0,2887
PAM16	192,6	88,6	137,2	63,4	0,7	0,3478
PAM17	27,6	6,8	17,3	18,3	0,6	0,3344
PAM18	28,6	16,4	9,2	8,3	0,3	0,0789
PAN1	45,2	12,3	39,7	27,5	0,9	0,7292
PAN2	143,9	106,5	65,9	46,9	0,5	0,2288
PAN3	30,8	14,9	51,1	38,7	1,7	0,3654
PAN5	31,6	12,3	19,3	19,1	0,6	0,3190
PAP1	66,3	16,6	74,7	63,2	1,1	0,8042
PAP2	40,7	16,8	16,2	11,6	0,4	0,0532
PAT1	17,1	13,7	8,0	8,2	0,5	0,3018
PAU13	9,4	9,0	5,0	5,8	0,5	0,4409
PAU15	0,0	0,0	0,0	0,0	#DIV/0!	#DIV/0!
PAU17	25,1	13,7	26,5	24,5	1,1	0,9270
PAU18	2,7	4,0	0,7	1,3	0,2	0,3684
PAU19	0,0	0,0	0,0	0,0	#DIV/0!	#DIV/0!
PAU24	0,0	0,0	0,0	0,0	#DIV/0!	#DIV/0!
PAU3	0,0	0,0	0,0	0,0	#DIV/0!	#DIV/0!
PAU5	2,1	2,8	0,0	0,0	0,0	0,1901
PAU7	3,2	2,2	1,6	3,3	0,5	0,4561
PBA1	18,3	12,0	8,4	7,3	0,5	0,2105
PBN1	13,8	10,1	9,2	10,7	0,7	0,5544
PBP1	154,3	60,3	286,5	17,3	1,9	0,0056
PBP2	29,3	23,0	7,6	6,4	0,3	0,1191
PBS2	55,7	24,3	60,8	42,5	1,1	0,8411
PBY1	48,2	14,2	49,5	42,7	1,0	0,9580
PCA1	7,9	6,0	5,0	7,2	0,6	0,5551
PCC1	119,6	20,4	425,2	171,7	3,6	0,0123
PCD1	60,6	9,6	88,7	67,4	1,5	0,4408
PCF11	57,5	27,2	31,3	20,9	0,5	0,1778

PCH2	151,0	112,7	34,2	37,0	0,2	0,0963
PCI8	1,6	1,9	1,8	2,3	1,2	0,8655
PCK1	19,7	14,0	4,9	4,4	0,2	0,0905
PCL1	44,7	26,9	2,4	3,4	0,1	0,0207
PCL10	29,8	14,9	94,9	84,3	3,2	0,1786
PCL2	63,2	26,7	28,3	22,2	0,4	0,0912
PCL5	693,4	347,2	1915,0	1103,0	2,8	0,0791
PCL6	1044,9	541,3	457,7	193,4	0,4	0,0871
PCL7	64,6	40,4	124,3	50,4	1,9	0,1137
PCL8	58,5	38,7	25,1	27,2	0,4	0,2075
PCL9	39,3	18,9	40,6	30,9	1,0	0,9457
PCM1	22,2	17,6	36,1	38,8	1,6	0,5376
PCP1	21,8	15,1	19,4	13,6	0,9	0,8207
PCS60	23,6	32,0	10,6	10,0	0,4	0,4676
PCT1	22,1	3,8	11,5	8,9	0,5	0,0726
PDA1	374,7	123,1	725,8	184,2	1,9	0,0193
PDB1	438,1	327,2	550,9	262,5	1,3	0,6102
PDC1	2686,7	2064,3	3108,2	2239,7	1,2	0,7913
PDC2	20,5	15,4	16,1	15,9	0,8	0,7033
PDC5	8,0	7,7	157,7	88,5	19,7	0,0150
PDC6	39,2	15,4	180,5	128,5	4,6	0,0718
PDE1	18,9	15,5	39,8	39,2	2,1	0,3591
PDE2	50,8	23,9	41,8	37,8	0,8	0,7006
PDH1	107,4	75,8	42,8	31,7	0,4	0,1669
PDI1	58,2	17,9	53,0	49,0	0,9	0,8481
PDR1	13,4	12,8	18,8	23,0	1,4	0,6942
PDR10	41,7	7,8	29,7	22,4	0,7	0,3533
PDR11	116,6	28,7	89,6	44,0	0,8	0,3432
PDR12	45,3	30,5	26,5	26,2	0,6	0,3852
PDR15	145,0	130,8	178,8	172,8	1,2	0,7656
PDR16	289,7	221,4	122,4	100,1	0,4	0,2177
PDR17	70,2	41,3	106,4	98,3	1,5	0,5226
PDR18	42,9	30,8	86,7	69,8	2,0	0,2939
PDR3	1427,5	1038,5	1851,3	1585,0	1,3	0,6703
PDR5	68,6	24,6	19,0	14,1	0,3	0,0128
PDR8	80,4	48,1	54,6	46,4	0,7	0,4704
PDS1	27,8	18,4	15,4	10,4	0,6	0,2836
PDS5	180,1	115,7	29,0	20,0	0,2	0,0421
PDX1	26,5	8,0	102,3	46,2	3,9	0,0178
PDX3	136,5	72,0	160,4	129,4	1,2	0,7582
PEF1	28,7	6,8	10,3	9,6	0,4	0,0208
PEP1	124,0	45,1	137,0	117,8	1,1	0,8443
PEP12	99,5	61,3	291,7	156,6	2,9	0,0624
PEP3	6,1	5,9	20,4	16,3	3,3	0,1518
PEP4	946,3	357,6	1124,0	742,0	1,2	0,6811
PEP5	36,9	15,0	99,9	64,8	2,7	0,1072
PEP7	9,7	6,0	10,0	10,9	1,0	0,9582
PEP8	116,5	68,1	73,3	50,8	0,6	0,3485
PER1	106,9	31,8	53,5	56,5	0,5	0,1501
PER33	72,0	66,6	37,8	37,2	0,5	0,4037

PES4	3,0	4,6	5,4	7,2	1,8	0,5924
PET111	7,1	7,4	10,5	8,2	1,5	0,5665
PET112	8,2	8,5	6,2	7,5	0,7	0,7254
PET117	24,4	15,6	35,2	28,6	1,4	0,5323
PET122	0,0	0,0	0,0	0,0	#DIV/0!	#DIV/0!
PET123	60,6	38,9	47,3	35,6	0,8	0,6331
PET127	8,2	7,5	1,9	2,4	0,2	0,1626
PET130	35,3	23,6	15,4	10,4	0,4	0,1741
PET18	15,7	4,2	11,3	7,9	0,7	0,3735
PET191	71,9	53,3	25,6	17,6	0,4	0,1504
PET20	73,3	56,0	136,3	95,4	1,9	0,2983
PET309	1,4	2,7	1,9	2,4	1,4	0,7732
PET494	20,5	11,5	13,0	11,9	0,6	0,4000
PET54	92,4	35,6	22,7	17,1	0,2	0,0124
PET8	114,5	52,1	76,2	55,8	0,7	0,3555
PET9	765,9	298,5	435,2	221,8	0,6	0,1257
PEX1	17,1	11,3	12,1	15,2	0,7	0,6151
PEX10	30,6	25,3	13,6	11,3	0,4	0,2667
PEX11	311,3	103,6	271,8	81,1	0,9	0,5694
PEX12	66,4	40,2	30,9	26,8	0,5	0,1927
PEX13	71,9	40,1	35,9	28,5	0,5	0,1937
PEX14	11,0	10,8	18,0	14,0	1,6	0,4623
PEX18	147,9	119,3	45,2	64,4	0,3	0,1805
PEX19	161,2	20,6	119,5	89,6	0,7	0,4001
PEX2	12,7	8,2	10,0	7,0	0,8	0,6360
PEX21	279,4	53,9	223,4	174,4	0,8	0,5618
PEX22	47,3	14,8	38,3	35,9	0,8	0,6600
PEX25	28,8	22,9	13,2	13,1	0,5	0,2815
PEX27	23,2	5,3	38,0	31,4	1,6	0,3896
PEX28	37,3	26,1	68,6	47,0	1,8	0,2892
PEX29	295,1	99,7	213,1	100,0	0,7	0,2894
PEX3	9,7	8,4	11,0	12,7	1,1	0,8689
PEX30	12,1	12,7	12,5	13,2	1,0	0,9690
PEX31	12,4	5,1	6,1	5,0	0,5	0,1221
PEX32	31,0	18,7	22,4	15,5	0,7	0,5097
PEX4	82,0	43,9	105,3	90,2	1,3	0,6588
PEX5	48,8	14,2	16,5	15,3	0,3	0,0212
PEX6	41,9	16,1	27,8	20,4	0,7	0,3196
PEX7	18,1	11,9	12,9	9,5	0,7	0,5155
PEX8	84,2	65,3	156,9	121,3	1,9	0,3317
PFA3	3,1	3,9	1,2	2,4	0,4	0,4261
PFA4	224,6	64,6	98,9	72,8	0,4	0,0416
PFA5	36,7	17,1	22,9	17,8	0,6	0,3050
PFD1	18,1	9,7	17,3	14,6	1,0	0,9310
PFK1	499,4	51,7	581,2	28,2	1,2	0,0319
PFK2	3461,1	1385,8	3169,7	589,4	0,9	0,7122
PFK26	35,3	14,6	41,1	29,4	1,2	0,7359
PFK27	79,6	67,3	68,8	46,3	0,9	0,8011
PFS1	1,0	2,0	0,0	0,0	0,0	0,3559
PFS2	4,3	2,1	6,6	5,2	1,5	0,4471

PFY1	296,1	232,2	529,0	397,6	1,8	0,3508
PGA1	9,4	6,2	15,5	10,9	1,6	0,3721
PGA2	0,9	1,8	1,6	3,3	1,9	0,6958
PGA3	34,7	21,3	27,5	34,1	0,8	0,7304
PGC1	298,1	166,0	567,6	263,0	1,9	0,1339
PGD1	82,7	41,9	41,6	29,8	0,5	0,1619
PGI1	646,1	611,9	586,6	536,7	0,9	0,8885
PGM1	5,1	4,2	0,0	0,0	0,0	0,0487
PGM2	16,8	12,0	32,1	22,5	1,9	0,2753
PGM3	16,7	15,8	32,8	22,6	2,0	0,2868
PGS1	685,9	444,4	346,2	180,3	0,5	0,2064
PGU1	53,6	26,1	816,2	826,6	15,2	0,1147
PHA2	29,4	9,5	78,5	104,3	2,7	0,3845
PHB1	13,9	15,9	30,6	35,4	2,2	0,4224
PHB2	74,4	50,9	122,3	53,9	1,6	0,2429
PHD1	125,7	76,0	352,1	281,7	2,8	0,1716
PHM6	18,7	11,4	64,8	60,7	3,5	0,1863
PHM7	16,2	20,1	7,2	8,3	0,4	0,4375
PHM8	397,6	113,4	459,2	365,8	1,2	0,7587
PHO11	23,0	24,6	110,2	116,2	4,8	0,1920
PHO13	17,7	17,5	19,2	21,4	1,1	0,9178
PHO2	14,5	13,6	12,8	14,8	0,9	0,8725
PHO23	1,9	2,8	1,2	2,4	0,6	0,6975
PHO3	146,8	82,1	135,5	127,4	0,9	0,8868
PHO4	25,5	14,7	10,5	9,5	0,4	0,1379
PHO5	10,6	7,3	73,5	64,6	7,0	0,1008
PHO8	56,6	9,5	226,8	83,2	4,0	0,0066
PHO80	244,0	149,0	89,3	60,2	0,4	0,1025
PHO81	24,7	15,1	44,8	41,1	1,8	0,3933
PHO84	248,5	172,8	2173,1	1777,6	8,7	0,0746
PHO85	43,0	22,9	13,5	9,0	0,3	0,0533
PHO86	275,9	56,6	239,3	160,5	0,9	0,6816
PHO87	9,5	9,0	6,7	8,5	0,7	0,6683
PHO88	105,5	65,7	54,9	50,1	0,5	0,2673
PHO89	23,8	6,1	7,1	7,9	0,3	0,0155
PHO90	138,9	46,1	94,1	76,9	0,7	0,3556
PHO91	35,9	15,3	37,2	25,7	1,0	0,9314
PHR1	0,6	1,2	5,4	4,2	9,1	0,0719
PHS1	140,2	133,9	68,7	62,0	0,5	0,3701
PIB1	118,3	144,1	55,5	37,0	0,5	0,4307
PIB2	29,5	21,6	31,7	33,0	1,1	0,9172
PIC2	130,0	43,3	165,6	130,3	1,3	0,6226
PIF1	16,7	14,5	3,5	4,1	0,2	0,1313
PIG1	5,6	4,9	5,4	4,9	1,0	0,9584
PIG2	18,2	8,4	16,4	29,6	0,9	0,9088
PIH1	9,2	9,6	0,0	0,0	0,0	0,1063
PIK1	17,5	14,7	31,6	24,9	1,8	0,3671
PIL1	1321,5	1079,6	1371,0	1236,6	1,0	0,9538
PIM1	69,7	87,3	26,4	20,8	0,4	0,3718
PIN2	37,3	9,7	26,3	18,5	0,7	0,3324

PIN4	306,3	206,4	312,6	252,8	1,0	0,9703
PIP2	18,2	15,9	20,0	16,6	1,1	0,8805
PIR1	204,6	146,5	116,3	86,9	0,6	0,3397
PIR3	34,2	17,3	106,3	78,9	3,1	0,1241
PIS1	171,6	117,0	150,5	148,3	0,9	0,8301
PKC1	32,0	21,4	24,4	19,1	0,8	0,6137
PKH1	23,9	11,6	13,7	19,9	0,6	0,4100
PKH2	19,3	10,6	24,4	19,3	1,3	0,6552
PKH3	45,6	22,1	24,2	17,5	0,5	0,1797
PKP1	6,6	8,7	3,7	3,1	0,6	0,5578
PKP2	65,1	23,9	125,2	90,8	1,9	0,2479
PKR1	17,6	23,6	15,3	20,6	0,9	0,8865
PLB1	8,7	6,7	22,8	20,7	2,6	0,2415
PLB2	6,9	4,6	0,0	0,0	0,0	0,0247
PLB3	162,3	24,9	235,1	125,5	1,4	0,2990
PLC1	42,2	6,4	29,4	23,9	0,7	0,3413
PLM2	40,8	33,3	15,0	14,0	0,4	0,2035
PLP1	6,4	2,3	4,8	7,2	0,7	0,6776
PLP2	402,2	244,2	216,9	239,6	0,5	0,3204
PMA1	3581,1	1901,1	4089,0	1277,8	1,1	0,6729
PMA2	16,6	11,3	4,2	4,9	0,3	0,0904
PMC1	66,0	38,0	117,4	81,4	1,8	0,2958
PMD1	39,9	31,8	39,0	30,6	1,0	0,9659
PMI40	60,7	34,7	68,8	120,0	1,1	0,9014
PML1	71,6	32,4	8,4	10,5	0,1	0,0100
PML39	8,0	6,5	0,0	0,0	0,0	0,0494
PMP1	1564,0	883,5	3249,7	1469,0	2,1	0,0968
PMP3	325,8	116,3	400,7	111,0	1,2	0,3871
PMR1	279,8	176,1	216,7	41,3	0,8	0,5117
PMS1	44,1	13,2	7,7	6,0	0,2	0,0024
PMT1	126,9	104,8	49,8	51,7	0,4	0,2349
PMT2	340,0	172,7	235,4	48,6	0,7	0,2875
PMT3	203,8	90,4	119,9	124,8	0,6	0,3184
PMT4	37,5	28,8	11,0	12,7	0,3	0,1434
PMT5	67,7	44,5	29,4	27,8	0,4	0,1943
PMT6	248,9	72,6	153,5	127,3	0,6	0,2408
PMU1	16,9	8,5	21,6	18,5	1,3	0,6603
PNC1	171,1	73,1	446,2	194,9	2,6	0,0384
PNG1	38,9	20,3	41,2	28,0	1,1	0,8964
PNP1	17,9	13,5	12,1	9,4	0,7	0,5077
PNS1	12,2	11,1	26,5	23,7	2,2	0,3165
PNT1	22,4	9,6	26,7	17,9	1,2	0,6922
POA1	778,0	391,7	276,8	286,6	0,4	0,0845
POB3	105,1	55,5	149,4	149,0	1,4	0,5977
POC4	114,8	101,0	172,0	253,7	1,5	0,6901
POG1	12,0	8,4	27,6	25,0	2,3	0,2791
POL1	141,0	62,7	29,8	24,5	0,2	0,0163
POL12	30,3	12,1	14,3	12,1	0,5	0,1104
POL2	115,8	37,7	22,0	16,8	0,2	0,0039
POL3	0,0	0,0	3,0	5,9	#DIV/0!	0,3559

POL31	6,0	6,9	2,4	2,8	0,4	0,3786
POL32	17,3	12,7	4,9	5,7	0,3	0,1245
POL4	34,6	11,8	10,7	7,2	0,3	0,0136
POL5	179,0	30,9	85,6	64,1	0,5	0,0393
POM152	17,4	13,9	13,6	11,8	0,8	0,6839
POM33	68,7	85,6	130,2	136,4	1,9	0,4744
POM34	23,2	21,9	12,3	11,4	0,5	0,4119
POP1	16,2	12,5	71,6	92,6	4,4	0,2803
POP2	51,0	3,7	77,9	72,1	1,5	0,4839
POP3	7,5	5,2	77,7	114,2	10,3	0,2657
POP4	111,5	42,2	131,6	115,7	1,2	0,7545
POP5	20,2	14,3	1,8	3,6	0,1	0,0465
POP6	211,0	101,5	43,8	30,3	0,2	0,0196
POP7	60,7	33,1	48,2	38,3	0,8	0,6398
POP8	44,2	20,2	73,3	99,3	1,7	0,5856
POR1	547,4	529,1	1485,6	1506,3	2,7	0,2844
POR2	3,2	4,9	2,4	4,7	0,7	0,8198
POS5	130,8	44,8	193,9	73,5	1,5	0,1931
POT1	84,0	92,3	19,0	14,2	0,2	0,2127
POX1	388,7	403,8	211,6	156,5	0,5	0,4446
PPA2	22,8	7,8	18,7	14,6	0,8	0,6381
PPE1	5,3	6,6	1,9	2,4	0,4	0,3672
PPG1	14,7	15,5	19,5	17,5	1,3	0,6950
PPH21	161,3	76,3	116,4	79,2	0,7	0,4450
PPH22	32,9	20,5	53,0	84,3	1,6	0,6602
PPH3	38,3	44,6	12,8	9,0	0,3	0,3039
PPM1	4,2	4,9	4,5	5,3	1,1	0,9442
PPM2	56,4	32,9	43,3	36,5	0,8	0,6147
PPN1	572,9	306,8	538,4	398,0	0,9	0,8953
PPQ1	193,1	84,0	69,0	46,7	0,4	0,0416
PPR1	42,8	38,2	14,7	11,9	0,3	0,2096
PPS1	137,4	92,6	66,4	61,9	0,5	0,2494
PPT1	562,7	259,8	2330,0	1369,8	4,1	0,0444
PPT2	7,3	5,0	1,6	3,3	0,2	0,1085
PPX1	30,0	25,2	22,3	17,9	0,7	0,6377
PPZ1	75,6	57,7	234,7	231,9	3,1	0,2313
PPZ2	11,2	6,1	29,0	21,1	2,6	0,1551
PRB1	79,4	16,2	93,1	24,6	1,2	0,3857
PRC1	87,3	55,0	105,6	107,4	1,2	0,7726
PRD1	11,4	8,7	6,9	5,5	0,6	0,4082
PRE1	82,8	65,1	97,8	94,8	1,2	0,8024
PRE10	162,1	127,7	280,2	145,0	1,7	0,2673
PRE2	1041,2	691,0	530,4	338,8	0,5	0,2326
PRE3	29,8	12,6	228,1	345,0	7,7	0,2942
PRE4	52,1	18,7	121,7	60,5	2,3	0,0701
PRE5	54,7	55,0	89,9	94,2	1,6	0,5425
PRE6	179,7	126,1	304,1	257,1	1,7	0,4185
PRE7	74,8	28,3	46,5	41,9	0,6	0,3060
PRE8	57,1	24,9	62,0	47,1	1,1	0,8589
PRE9	104,8	36,6	79,0	57,2	0,8	0,4770

PRI1	7,2	11,6	6,2	7,5	0,9	0,8824
PRI2	26,0	12,6	17,2	12,0	0,7	0,3536
PRK1	9,5	5,0	2,6	3,6	0,3	0,0649
PRM1	98,7	23,3	106,4	74,1	1,1	0,8494
PRM10	25,7	24,3	27,4	19,7	1,1	0,9192
PRM2	155,6	64,0	184,8	68,1	1,2	0,5553
PRM4	170,8	74,6	68,4	67,2	0,4	0,0876
PRM5	35,8	19,1	68,9	59,5	1,9	0,3314
PRM6	0,9	1,8	7,8	9,3	8,5	0,1973
PRM7	129,1	134,4	102,4	110,9	0,8	0,7693
PRM8	104,9	32,0	80,3	68,2	0,8	0,5388
PRM9	44,8	28,8	140,5	201,1	3,1	0,3826
PRO1	18,6	14,3	17,8	19,1	1,0	0,9505
PRO2	294,3	187,2	337,6	229,7	1,1	0,7800
PRO3	101,1	106,1	104,8	119,1	1,0	0,9650
PRP11	34,8	10,9	33,7	26,6	1,0	0,9397
PRP16	8,6	11,2	12,8	14,8	1,5	0,6638
PRP18	3,2	6,4	8,4	5,8	2,6	0,2718
PRP19	15,9	14,4	7,2	5,4	0,5	0,2999
PRP2	16,0	22,0	18,6	16,7	1,2	0,8572
PRP21	6,7	9,3	2,6	5,3	0,4	0,4783
PRP22	37,1	16,2	37,1	27,3	1,0	0,9973
PRP24	22,0	7,2	11,1	8,9	0,5	0,1083
PRP28	55,6	15,7	24,2	24,0	0,4	0,0710
PRP3	86,4	55,0	88,0	64,2	1,0	0,9710
PRP31	39,6	7,8	35,5	26,4	0,9	0,7724
PRP38	61,8	23,2	51,2	35,1	0,8	0,6351
PRP39	18,2	18,0	17,8	17,1	1,0	0,9734
PRP4	3,5	4,1	1,2	2,4	0,3	0,3584
PRP40	17,5	16,0	18,3	15,2	1,0	0,9438
PRP42	24,7	7,8	25,4	18,7	1,0	0,9425
PRP43	68,5	51,2	71,0	69,9	1,0	0,9558
PRP45	11,8	8,5	7,8	5,8	0,7	0,4667
PRP46	5,8	2,8	12,0	10,5	2,1	0,2914
PRP5	25,3	14,6	24,9	22,9	1,0	0,9747
PRP6	25,4	9,9	24,9	20,9	1,0	0,9625
PRP8	26,2	15,3	29,1	21,8	1,1	0,8330
PRP9	27,6	7,3	13,0	10,6	0,5	0,0633
PRR1	38,1	22,5	21,2	17,9	0,6	0,2822
PRR2	32,8	7,0	311,6	41,3	9,5	0,0000
PRS1	99,0	64,5	68,6	52,8	0,7	0,4934
PRS2	225,6	71,0	99,7	69,8	0,4	0,0448
PRS3	42,4	38,0	68,8	60,2	1,6	0,4872
PRS4	134,5	41,2	314,2	140,7	2,3	0,0498
PRS5	52,7	27,8	29,1	28,7	0,6	0,2827
PRT1	141,0	31,8	316,0	378,2	2,2	0,3918
PRX1	23,3	13,5	42,5	46,1	1,8	0,4567
PRY1	359,4	272,7	86,4	85,7	0,2	0,1048
PRY2	404,7	125,6	227,8	188,7	0,6	0,1696
PRY3	10,0	8,3	6,0	9,0	0,6	0,5358

PSA1	1616,7	130,7	407,5	87,9	0,3	0,0000
PSD1	33,6	19,8	12,6	13,5	0,4	0,1299
PSD2	97,3	65,0	108,7	99,8	1,1	0,8549
PSE1	43,5	35,3	20,2	19,7	0,5	0,2937
PSF1	115,8	17,0	289,3	304,4	2,5	0,2984
PSF2	11,2	9,4	6,1	7,1	0,5	0,4259
PSF3	24,1	20,2	7,9	9,5	0,3	0,1969
PSH1	1,7	2,2	49,5	95,8	28,8	0,3575
PSK1	28,7	14,5	46,3	35,3	1,6	0,3913
PSK2	38,7	26,0	37,9	32,5	1,0	0,9721
PSO2	16,2	16,7	12,9	10,8	0,8	0,7517
PSP1	29,3	20,6	27,6	30,3	0,9	0,9271
PSP2	46,2	38,7	211,0	55,9	4,6	0,0029
PSR1	521,2	255,7	627,0	415,1	1,2	0,6794
PSR2	54,5	28,5	20,4	13,8	0,4	0,0742
PST1	27,3	24,0	16,5	15,4	0,6	0,4761
PST2	224,5	122,7	330,2	129,9	1,5	0,2819
PSY2	11,9	6,4	6,3	4,8	0,5	0,2100
PSY3	9,6	3,8	1,3	2,6	0,1	0,0124
PSY4	24,4	14,6	16,9	22,5	0,7	0,5976
PTA1	50,2	26,4	46,5	38,1	0,9	0,8773
PTC1	109,0	43,8	40,7	32,0	0,4	0,0453
PTC2	280,5	77,6	121,9	100,7	0,4	0,0468
PTC3	215,5	54,5	121,8	84,2	0,6	0,1111
PTC4	37,6	33,9	26,1	18,3	0,7	0,5747
PTC5	41,6	17,1	62,8	41,1	1,5	0,3776
PTC6	6,8	6,1	5,1	7,9	0,8	0,7503
PTC7	48,6	22,2	20,9	15,5	0,4	0,0859
PTH1	3,9	4,8	3,5	4,1	0,9	0,9125
PTH2	21,6	13,5	24,9	25,1	1,2	0,8231
PTI1	148,9	90,8	118,9	90,2	0,8	0,6552
PTK1	111,7	105,7	36,5	28,4	0,3	0,2187
PTK2	115,2	57,7	225,8	66,5	2,0	0,0457
PTM1	108,7	75,2	174,5	104,4	1,6	0,3460
PTP1	30,5	10,8	48,2	39,7	1,6	0,4231
PTP2	12,5	6,3	17,3	15,9	1,4	0,5943
PTP3	23,8	17,8	17,6	14,6	0,7	0,6123
PTR2	165,9	47,7	53,6	39,2	0,3	0,0109
PTR3	20,4	14,5	22,6	18,7	1,1	0,8563
PUB1	307,8	114,5	314,3	211,1	1,0	0,9588
PUF2	56,6	22,0	64,2	47,1	1,1	0,7797
PUF3	1,1	2,3	3,2	4,8	2,8	0,4667
PUF4	39,2	24,0	32,0	36,1	0,8	0,7523
PUF6	77,5	69,4	59,4	55,4	0,8	0,6970
PUG1	20,8	19,1	3,9	5,0	0,2	0,1372
PUN1	48,7	31,6	141,8	99,8	2,9	0,1256
PUP1	41,2	21,8	33,0	28,2	0,8	0,6634
PUP2	109,6	33,7	155,3	65,9	1,4	0,2631
PUP3	312,3	247,6	348,3	165,0	1,1	0,8168
PUS1	21,1	10,5	25,5	25,8	1,2	0,7634

PUS2	36,7	40,8	19,1	17,5	0,5	0,4577
PUS4	29,2	7,8	52,1	16,4	1,8	0,0448
PUS5	30,0	16,7	41,3	30,6	1,4	0,5421
PUS6	10,8	2,6	10,3	8,3	1,0	0,9149
PUS7	70,7	38,3	110,0	56,7	1,6	0,2953
PUS9	56,1	3,8	59,2	48,7	1,1	0,9032
PUT1	45,6	25,5	96,4	148,0	2,1	0,5238
PUT2	55,4	29,9	48,0	38,8	0,9	0,7720
PUT3	20,7	10,7	43,3	30,0	2,1	0,2059
PUT4	36,0	17,0	14,2	11,4	0,4	0,0780
PWP1	351,9	196,2	387,4	279,6	1,1	0,8423
PWP2	121,7	64,8	215,3	35,7	1,8	0,0448
PXA1	30,2	7,3	17,8	12,0	0,6	0,1283
PXA2	144,6	83,2	136,3	112,6	0,9	0,9091
PXL1	76,7	15,6	54,7	63,7	0,7	0,5272
PXR1	775,3	644,8	992,3	910,3	1,3	0,7107
PYC1	240,2	126,7	565,8	273,7	2,4	0,0741
PYC2	982,9	326,7	1216,9	311,3	1,2	0,3396
PYK2	3,8	3,2	21,8	21,6	5,8	0,1500
PZF1	13,1	0,7	19,1	15,8	1,5	0,4766
Q0142	585,4	1169,2	4,4	3,0	0,0	0,3587
Q0144	38,7	46,1	31,0	23,9	0,8	0,7767
Q0255	54,2	103,0	0,0	0,0	0,0	0,3331
QCR10	16,8	13,9	22,5	22,3	1,3	0,6803
QCR2	1007,8	573,5	1760,5	462,7	1,7	0,0870
QCR6	186,1	96,1	353,5	83,9	1,9	0,0394
QCR7	508,5	328,3	753,9	522,5	1,5	0,4568
QCR8	68,4	45,6	71,3	73,6	1,0	0,9489
QCR9	59,3	29,9	64,7	45,2	1,1	0,8463
QDR1	4,8	6,4	3,7	3,2	0,8	0,7686
QDR2	51,0	8,9	131,5	34,2	2,6	0,0039
QDR3	94,8	31,2	45,3	32,8	0,5	0,0712
QNS1	120,4	78,7	103,6	79,5	0,9	0,7748
QRI1	78,5	14,2	35,4	26,2	0,5	0,0277
QRI5	6,6	5,3	6,8	8,2	1,0	0,9770
QRI7	90,6	29,2	197,9	63,9	2,2	0,0224
RAD1	33,2	14,5	91,4	88,2	2,7	0,2412
RAD14	81,0	15,6	77,8	52,0	1,0	0,9103
RAD16	31,5	8,8	36,1	32,3	1,1	0,7931
RAD17	54,0	35,9	50,4	44,3	0,9	0,9055
RAD18	5,4	4,1	8,6	8,0	1,6	0,5090
RAD2	105,9	50,4	128,3	102,0	1,2	0,7071
RAD23	134,9	72,0	245,7	141,3	1,8	0,2119
RAD24	12,6	5,5	5,4	9,1	0,4	0,2261
RAD26	12,5	3,6	21,3	17,0	1,7	0,3508
RAD27	121,3	51,1	29,7	20,5	0,2	0,0158
RAD28	12,8	7,9	116,3	161,7	9,1	0,2483
RAD3	39,1	11,8	22,1	16,3	0,6	0,1421
RAD30	11,2	3,1	19,1	27,3	1,7	0,5892
RAD33	64,9	43,4	33,4	25,3	0,5	0,2568

RAD34	8,3	9,9	16,0	19,7	1,9	0,5091
RAD4	21,3	14,7	16,9	11,6	0,8	0,6498
RAD5	45,2	16,0	38,0	30,4	0,8	0,6885
RAD50	32,9	9,6	12,4	9,9	0,4	0,0247
RAD51	7,7	4,7	3,0	3,6	0,4	0,1674
RAD52	45,3	60,1	104,2	179,3	2,3	0,5562
RAD53	5,0	7,5	0,0	0,0	0,0	0,2349
RAD54	223,0	92,5	644,0	475,8	2,9	0,1330
RAD55	10,7	9,2	1,6	3,3	0,2	0,1120
RAD57	62,1	36,3	40,5	34,3	0,7	0,4189
RAD59	1,9	2,2	5,3	10,7	2,8	0,5525
RAD6	70,1	56,4	156,6	87,8	2,2	0,1481
RAD61	12,4	6,7	12,6	10,3	1,0	0,9745
RAD7	6,6	6,0	6,3	12,7	1,0	0,9740
RAD9	277,6	214,4	390,1	152,2	1,4	0,4251
RAI1	18,7	7,8	30,7	32,4	1,6	0,5004
RAM1	19,8	11,9	16,3	21,4	0,8	0,7866
RAM2	11,7	13,5	9,7	10,8	0,8	0,8304
RAP1	35,8	20,2	174,9	237,2	4,9	0,2868
RAS1	37,9	27,2	23,1	28,0	0,6	0,4765
RAS2	283,7	75,9	374,1	301,9	1,3	0,5822
RAT1	77,8	29,6	315,2	31,1	4,1	0,0000
RAV1	11,3	4,0	13,1	11,9	1,2	0,7911
RAV2	32,0	17,7	21,6	18,2	0,7	0,4457
RAX1	25,9	13,7	18,4	22,7	0,7	0,5906
RAX2	25,3	9,1	41,4	27,6	1,6	0,3094
RBA50	207,4	106,6	464,0	199,9	2,2	0,0640
RBD2	94,7	58,8	154,5	63,3	1,6	0,2154
RBG1	339,3	257,7	557,1	428,6	1,6	0,4170
RBG2	198,5	20,8	175,3	47,9	0,9	0,4077
RBK1	6,8	6,0	7,4	6,1	1,1	0,8928
RBL2	84,8	77,2	43,5	45,9	0,5	0,3934
RBS1	47,6	32,0	41,1	34,6	0,9	0,7928
RCE1	12,5	4,1	59,9	89,3	4,8	0,3291
RCK1	0,0	0,0	2,4	2,8	#DIV/0!	0,1343
RCK2	50,7	25,6	63,8	54,8	1,3	0,6789
RCL1	140,2	15,6	41,6	31,8	0,3	0,0014
RCN1	42,2	27,2	10,3	8,2	0,2	0,0658
RCN2	71,9	39,6	283,3	247,0	3,9	0,1420
RCO1	6,8	8,1	9,2	8,3	1,4	0,6897
RCR1	9,8	9,8	10,1	9,9	1,0	0,9645
RCR2	14,6	5,3	14,0	16,3	1,0	0,9513
RCY1	0,0	0,0	1,8	3,6	#DIV/0!	0,3559
RDH54	49,0	25,6	14,3	11,0	0,3	0,0475
RDI1	61,0	24,3	67,6	40,1	1,1	0,7871
RDL1	213,0	85,0	474,6	139,9	2,2	0,0187
RDR1	24,6	29,0	16,0	24,4	0,7	0,6658
RDS1	18,0	15,1	13,2	11,7	0,7	0,6329
RDS2	25,2	17,4	15,2	18,0	0,6	0,4571
RDS3	16,9	16,1	11,5	8,8	0,7	0,5712

REB1	107,1	46,9	56,5	37,8	0,5	0,1436
REC102	22,9	22,4	70,6	109,4	3,1	0,4253
REC104	2,0	4,0	0,0	0,0	0,0	0,3559
REC107	51,3	50,2	70,5	85,5	1,4	0,7108
REC114	71,4	31,5	133,9	134,6	1,9	0,4008
REC8	3,2	2,2	2,4	3,4	0,8	0,7075
RED1	40,9	15,4	70,7	72,6	1,7	0,4533
REE1	29,8	31,9	205,3	226,3	6,9	0,1753
REF2	11,4	8,4	101,8	40,0	8,9	0,0045
REG1	76,5	35,1	42,0	31,7	0,5	0,1954
REG2	4,5	3,5	2,9	3,7	0,6	0,5405
REH1	19,0	9,8	11,0	11,9	0,6	0,3443
REP1	331,8	191,4	415,6	226,5	1,3	0,5925
REP2	22,6	20,1	20,5	14,9	0,9	0,8763
RER1	10,6	19,0	7,3	5,2	0,7	0,7444
RER2	98,6	10,2	84,5	71,2	0,9	0,7088
RET1	34,3	14,0	55,9	51,2	1,6	0,4465
RET2	101,5	67,0	105,7	57,5	1,0	0,9262
REV1	127,3	45,7	245,4	228,8	1,9	0,3504
REV3	13,6	6,2	8,6	11,8	0,6	0,4884
REV7	59,3	41,1	58,9	41,5	1,0	0,9880
REX2	346,0	204,0	343,6	172,8	1,0	0,9861
REX3	221,5	160,2	154,2	150,1	0,7	0,5624
REX4	12,4	14,3	3,8	4,3	0,3	0,2907
RFA1	554,1	225,8	172,5	82,1	0,3	0,0192
RFA3	0,0	0,0	0,0	0,0	#DIV/0!	#DIV/0!
RFC1	18,8	8,7	9,1	9,9	0,5	0,1911
RFC2	25,3	14,3	10,9	8,2	0,4	0,1319
RFC3	20,3	16,5	3,1	3,7	0,2	0,0870
RFC4	18,7	10,6	22,7	15,9	1,2	0,6862
RFC5	21,9	17,6	9,0	9,0	0,4	0,2390
RFM1	8,6	6,7	3,2	3,9	0,4	0,2220
RFS1	79,3	56,7	176,7	102,6	2,2	0,1474
RFT1	20,7	9,2	12,5	12,9	0,6	0,3402
RFU1	59,2	40,1	113,5	79,9	1,9	0,2702
RFX1	21,4	5,9	12,8	11,6	0,6	0,2335
RGA1	33,5	17,3	37,9	29,5	1,1	0,8080
RGA2	27,3	18,7	25,9	22,5	0,9	0,9275
RGC1	41,6	20,8	121,5	122,0	2,9	0,2444
RGD1	74,1	38,3	60,1	42,4	0,8	0,6428
RGD2	2,3	3,2	1,8	2,3	0,8	0,8209
RGI2	120,8	48,4	276,3	203,5	2,3	0,1877
RGM1	266,1	259,5	196,6	213,3	0,7	0,6931
RGP1	9,2	11,7	13,3	10,8	1,4	0,6281
RGR1	12,7	7,4	9,3	10,9	0,7	0,6275
RGS2	170,1	132,1	30,0	26,2	0,2	0,0827
RGT1	13,1	4,5	21,0	27,8	1,6	0,5939
RGT2	127,2	43,4	65,9	46,7	0,5	0,1024
RHB1	1,0	2,0	3,6	4,6	3,7	0,3290
RHO1	139,6	38,1	84,2	71,1	0,6	0,2186

RHO3	527,6	374,0	308,4	220,4	0,6	0,3517
RHO4	1,1	2,3	4,9	5,7	4,3	0,2631
RHO5	351,3	294,3	389,4	368,8	1,1	0,8772
RHR2	2115,0	1594,7	1927,7	1047,2	0,9	0,8508
RIA1	14,1	6,0	1,9	2,4	0,1	0,0097
RIB1	71,1	34,5	70,3	47,5	1,0	0,9805
RIB2	35,6	31,0	9,8	16,5	0,3	0,1919
RIB3	76,9	14,8	32,2	22,3	0,4	0,0158
RIB4	153,8	107,5	230,3	77,5	1,5	0,2919
RIB5	358,5	221,3	201,3	163,0	0,6	0,2964
RIB7	16,4	13,5	14,5	18,3	0,9	0,8731
RIC1	76,5	26,0	86,3	58,0	1,1	0,7676
RIF1	45,1	22,6	13,1	9,5	0,3	0,0400
RIF2	101,0	76,2	131,7	138,7	1,3	0,7114
RIM1	266,7	138,9	381,3	209,2	1,4	0,3967
RIM101	52,5	4,8	64,3	46,5	1,2	0,6313
RIM11	44,3	31,8	202,5	279,7	4,6	0,3042
RIM13	62,9	42,1	11,1	14,5	0,2	0,0590
RIM15	27,7	8,7	34,3	32,2	1,2	0,7067
RIM2	103,6	45,2	64,7	44,0	0,6	0,2639
RIM20	4,9	4,7	11,0	9,4	2,2	0,2910
RIM21	40,2	30,5	33,4	27,8	0,8	0,7529
RIM4	105,5	27,9	302,4	211,4	2,9	0,1142
RIM8	145,1	69,7	24,1	23,9	0,2	0,0168
RIM9	2,5	3,0	7,5	11,9	3,0	0,4484
RIO1	120,3	48,1	200,7	144,7	1,7	0,3326
RIO2	16,5	10,2	23,9	24,7	1,4	0,5996
RIP1	7,4	8,5	9,7	12,4	1,3	0,7643
RIT1	7,4	7,2	13,5	14,2	1,8	0,4758
RIX1	12,8	6,5	7,0	5,7	0,5	0,2275
RIX7	33,2	22,4	17,2	17,2	0,5	0,2979
RKI1	53,3	32,3	42,3	38,4	0,8	0,6770
RKM1	13,2	5,9	2,2	2,5	0,2	0,0143
RKM2	17,0	10,3	19,6	14,1	1,2	0,7789
RKM3	39,0	21,0	21,5	15,7	0,6	0,2301
RKM4	21,8	8,2	22,7	17,7	1,0	0,9318
RKR1	164,9	50,8	395,7	284,3	2,4	0,1610
RLF2	238,9	114,7	204,4	144,5	0,9	0,7206
RLI1	37,7	32,2	21,6	22,9	0,6	0,4477
RLM1	50,2	26,5	50,4	45,4	1,0	0,9926
RLP24	84,7	56,5	79,3	76,0	0,9	0,9130
RLP7	169,3	101,9	155,7	59,8	0,9	0,8254
RMA1	24,3	12,3	13,2	9,5	0,5	0,2029
RMD1	42,8	21,2	30,3	22,3	0,7	0,4477
RMD5	341,1	189,1	104,7	102,3	0,3	0,0702
RMD6	10,0	3,4	6,6	4,5	0,7	0,2731
RMD8	42,0	23,3	25,8	18,5	0,6	0,3185
RMD9	44,0	27,0	185,0	281,8	4,2	0,3576
RME1	80,0	32,0	291,4	231,0	3,6	0,1199
RMI1	108,6	93,9	54,3	60,6	0,5	0,3684

RML2	8,4	7,4	18,6	20,5	2,2	0,3843
RMR1	42,1	7,1	93,2	67,5	2,2	0,1830
RMT2	9,7	8,9	6,4	7,1	0,7	0,5810
RNA1	55,4	23,9	33,2	27,4	0,6	0,2670
RNA14	55,5	35,4	46,2	31,4	0,8	0,7078
RNA15	27,0	9,6	21,7	16,0	0,8	0,5922
RNH1	6,4	6,1	2,4	2,8	0,4	0,2857
RNH201	152,1	102,9	132,2	123,7	0,9	0,8129
RNH202	21,4	11,0	14,0	11,5	0,7	0,3891
RNH203	23,6	16,6	4,3	5,1	0,2	0,0688
RNH70	99,1	55,1	71,9	53,9	0,7	0,5078
RNQ1	51,0	41,3	43,0	40,3	0,8	0,7912
RNR1	202,9	167,6	4,3	5,6	0,0	0,0556
RNR2	30,3	18,3	15,0	21,0	0,5	0,3131
RNR3	4,0	2,8	5,7	5,2	1,4	0,5924
RNR4	315,8	175,6	520,9	306,2	1,6	0,2893
RNT1	81,4	22,6	61,0	54,9	0,7	0,5170
RNY1	2,9	4,3	11,5	8,8	4,0	0,1290
ROD1	2,3	4,5	3,2	6,3	1,4	0,8252
ROG1	6,4	1,7	2,0	4,0	0,3	0,0844
ROG3	9,1	8,4	15,8	17,3	1,7	0,5097
ROK1	42,2	23,3	39,6	27,8	0,9	0,8915
ROM1	25,4	6,8	53,4	40,2	2,1	0,2187
ROM2	59,7	28,6	53,6	37,5	0,9	0,8072
ROT1	121,9	128,9	40,3	37,5	0,3	0,2697
ROT2	31,6	16,3	25,1	24,6	0,8	0,6761
ROX1	64,3	59,0	37,7	37,1	0,6	0,4750
ROX3	37,6	19,9	15,0	15,7	0,4	0,1245
RPA12	177,4	55,9	167,3	27,6	0,9	0,7553
RPA135	43,9	25,7	23,5	23,6	0,5	0,2857
RPA14	52,9	37,8	56,5	58,6	1,1	0,9205
RPA190	137,9	50,9	144,2	102,2	1,0	0,9148
RPA34	170,7	92,4	164,8	121,1	1,0	0,9407
RPA43	82,3	43,1	113,5	81,8	1,4	0,5253
RPB10	271,0	100,7	290,3	146,6	1,1	0,8349
RPB11	13,0	10,6	65,6	69,3	5,1	0,1842
RPB2	121,6	81,7	79,1	66,7	0,7	0,4510
RPB3	16,9	13,6	7,4	9,6	0,4	0,2962
RPB4	186,3	57,8	85,9	57,4	0,5	0,0487
RPB5	208,8	119,2	173,4	168,5	0,8	0,7438
RPB7	130,9	48,4	266,8	282,9	2,0	0,3798
RPB8	120,0	124,7	139,7	132,7	1,2	0,8356
RPB9	65,2	35,0	33,8	25,8	0,5	0,1980
RPC11	114,4	84,1	56,0	54,5	0,5	0,2885
RPC17	65,6	23,7	31,3	23,6	0,5	0,0860
RPC25	84,6	47,3	43,3	29,2	0,5	0,1883
RPC31	84,4	53,3	76,4	52,3	0,9	0,8372
RPC34	14,1	13,2	14,1	16,2	1,0	0,9981
RPC37	23,9	14,3	14,7	13,6	0,6	0,3874
RPC40	113,5	35,9	189,7	93,2	1,7	0,1781

RPC53	44,3	6,5	80,0	103,1	1,8	0,5155
RPC82	54,9	22,3	45,3	32,9	0,8	0,6466
RPD3	85,6	31,6	73,6	52,2	0,9	0,7090
RPE1	4,9	6,2	3,6	4,6	0,7	0,7522
RPF1	67,8	18,4	64,2	46,8	0,9	0,8935
RPF2	45,5	26,1	37,1	31,8	0,8	0,6962
RPG1	215,7	154,7	51,8	43,6	0,2	0,0874
RPH1	2,7	2,0	4,5	7,3	1,6	0,6597
RPI1	154,3	70,3	274,2	209,3	1,8	0,3192
RPL11B	1276,9	512,2	862,6	258,3	0,7	0,1988
RPL12B	72,3	21,6	79,5	77,6	1,1	0,8640
RPL13A	0,0	0,0	1,0	2,0	#DIV/0!	0,3559
RPL14A	1146,7	345,6	527,2	313,1	0,5	0,0377
RPL14B	36,7	25,9	5,6	4,2	0,2	0,0558
RPL15B	27,1	25,9	42,1	41,1	1,6	0,5594
RPL16A	973,5	992,3	427,4	441,1	0,4	0,3534
RPL16B	272,4	175,7	203,0	175,0	0,7	0,5959
RPL17B	26,0	11,6	11,9	8,3	0,5	0,0959
RPL18B	2855,7	1456,8	415,9	304,9	0,1	0,0169
RPL19A	435,0	258,8	320,9	147,7	0,7	0,4728
RPL19B	391,5	258,0	203,0	199,3	0,5	0,2915
RPL1A	152,7	127,2	116,6	128,0	0,8	0,7034
RPL1B	1074,4	1000,0	649,3	332,7	0,6	0,4506
RPL20A	2271,7	588,8	862,9	335,3	0,4	0,0060
RPL20B	551,4	381,1	242,8	272,3	0,4	0,2357
RPL21A	565,0	505,0	401,3	434,2	0,7	0,6405
RPL21B	7202,2	6593,5	3719,3	2577,7	0,5	0,3631
RPL22B	385,8	209,5	143,1	106,3	0,4	0,0843
RPL23A	465,6	245,5	277,7	134,7	0,6	0,2282
RPL23B	1795,2	779,8	700,9	449,2	0,4	0,0510
RPL24A	3,8	2,6	7,1	7,9	1,9	0,4636
RPL24B	3,1	3,9	1,8	3,6	0,6	0,6278
RPL25	24784,7	13408,8	8983,4	3127,7	0,4	0,0615
RPL26A	3,8	3,2	8,5	5,8	2,3	0,2039
RPL26B	119,5	38,0	121,0	90,6	1,0	0,9772
RPL27A	1636,4	1502,8	868,0	769,9	0,5	0,3979
RPL27B	55,0	36,8	19,3	19,6	0,4	0,1374
RPL28	151,3	169,5	34,5	25,9	0,2	0,2221
RPL29	6300,9	2276,9	3050,3	994,7	0,5	0,0398
RPL2A	15,1	11,1	30,8	42,5	2,0	0,5020
RPL2B	2079,3	724,1	1544,2	465,8	0,7	0,2603
RPL3	1482,7	706,9	1125,2	789,0	0,8	0,5248
RPL30	90,6	17,6	119,9	77,6	1,3	0,4893
RPL31A	1094,2	502,6	1059,6	539,5	1,0	0,9282
RPL31B	812,5	445,3	270,8	184,5	0,3	0,0657
RPL32	1240,4	1235,9	1078,2	1095,6	0,9	0,8508
RPL33A	0,6	1,2	0,0	0,0	0,0	0,3559
RPL33B	9,8	7,4	2,6	5,3	0,3	0,1692
RPL34A	1413,5	558,7	1551,4	957,4	1,1	0,8118
RPL34B	4,4	3,0	2,5	5,1	0,6	0,5492

RPL35A	3,5	2,5	0,0	0,0	0,0	0,0302
RPL35B	19,5	9,5	6,4	7,7	0,3	0,0757
RPL36A	436,0	151,7	266,5	72,7	0,6	0,0905
RPL37A	22,2	17,8	1,8	2,3	0,1	0,0631
RPL37B	762,8	525,2	523,0	485,0	0,7	0,5273
RPL38	0,0	0,0	0,0	0,0	#DIV/0!	#DIV/0!
RPL39	2530,4	1398,1	1132,2	260,0	0,4	0,0969
RPL40A	49,8	29,4	23,2	23,8	0,5	0,2091
RPL41A	12708,7	7355,6	12836,6	6443,6	1,0	0,9800
RPL42A	26,5	6,2	27,6	18,7	1,0	0,9092
RPL42B	64,3	48,0	14,7	15,7	0,2	0,0977
RPL43A	12,6	13,9	3,5	4,4	0,3	0,2582
RPL43B	1702,1	1091,9	1015,3	886,9	0,6	0,3666
RPL4A	606,3	503,9	230,8	244,3	0,4	0,2285
RPL4B	638,1	278,0	337,6	183,7	0,5	0,1213
RPL5	1841,4	1321,9	1338,5	684,0	0,7	0,5244
RPL6A	947,3	786,7	507,9	568,7	0,5	0,4002
RPL6B	5,4	1,4	2,2	2,5	0,4	0,0641
RPL7A	963,2	300,1	498,6	222,3	0,5	0,0473
RPL7B	13,0	11,8	24,2	20,6	1,9	0,3831
RPL8A	219,6	174,6	24,7	16,9	0,1	0,0681
RPL9A	518,0	363,7	215,6	233,4	0,4	0,2112
RPL9B	531,4	264,0	307,7	294,1	0,6	0,3009
RPM2	38,3	24,2	72,0	48,6	1,9	0,2612
RPN1	99,0	65,9	124,6	112,6	1,3	0,7081
RPN11	49,9	48,5	67,9	76,9	1,4	0,7052
RPN12	15,5	3,4	14,5	10,7	0,9	0,8654
RPN13	84,0	45,5	74,0	50,2	0,9	0,7772
RPN14	43,9	20,8	89,3	71,2	2,0	0,2661
RPN2	817,5	651,7	997,0	769,5	1,2	0,7341
RPN3	214,1	93,7	312,4	53,8	1,5	0,1184
RPN4	146,5	60,1	168,8	117,1	1,2	0,7463
RPN5	197,5	84,2	273,9	166,9	1,4	0,4448
RPN6	136,9	86,3	131,5	112,0	1,0	0,9414
RPN7	206,0	98,9	397,6	97,8	1,9	0,0330
RPN8	112,0	80,4	85,5	61,8	0,8	0,6199
RPN9	115,3	25,9	191,9	150,9	1,7	0,3556
RPO21	160,5	118,3	133,5	134,3	0,8	0,7732
RPO26	170,0	103,6	112,7	68,9	0,7	0,3926
RPO31	169,1	51,4	147,7	99,9	0,9	0,7172
RPO41	6,2	3,8	20,2	14,9	3,2	0,1206
RPP0	3897,3	1450,4	2364,1	722,6	0,6	0,1073
RPP1	79,1	22,8	49,4	35,5	0,6	0,2086
RPP1A	1875,3	1559,1	2106,9	1274,9	1,1	0,8257
RPP1B	5,2	6,1	1,9	2,4	0,4	0,3571
RPP2A	2911,5	536,9	4864,5	2592,0	1,7	0,1905
RPR2	3,3	2,2	5,0	4,1	1,5	0,4880
RPS0A	791,4	458,0	630,6	454,1	0,8	0,6358
RPS0B	386,1	348,0	262,5	253,4	0,7	0,5869
RPS10A	1230,9	591,2	816,0	518,3	0,7	0,3319

RPS10B	741,9	505,4	408,2	404,8	0,6	0,3424
RPS11A	7,0	8,7	4,3	4,0	0,6	0,5979
RPS11B	0,0	0,0	1,8	3,6	#DIV/0!	0,3559
RPS13	913,7	699,2	543,7	573,8	0,6	0,4445
RPS14A	1578,9	958,8	1812,4	550,8	1,1	0,6875
RPS14B	940,4	366,5	451,4	175,6	0,5	0,0528
RPS15	951,9	523,4	642,0	434,6	0,7	0,3974
RPS16A	1056,4	390,9	530,4	220,9	0,5	0,0576
RPS16B	674,6	547,4	379,8	377,7	0,6	0,4095
RPS17A	7,1	12,8	0,0	0,0	0,0	0,3059
RPS17B	1204,6	369,0	611,7	439,6	0,5	0,0843
RPS18A	4,4	6,4	1,9	2,4	0,4	0,4996
RPS18B	481,3	523,3	267,5	299,7	0,6	0,5049
RPS19A	363,4	210,0	469,8	335,9	1,3	0,6102
RPS19B	377,3	369,7	357,4	388,7	0,9	0,9432
RPS1A	327,9	191,4	298,6	297,9	0,9	0,8737
RPS1B	4589,6	1791,2	1893,1	1152,4	0,4	0,0446
RPS2	1,6	1,9	0,0	0,0	0,0	0,1544
RPS21A	31,5	38,1	10,7	17,0	0,3	0,3575
RPS21B	3314,2	1786,4	3370,6	1180,2	1,0	0,9597
RPS22A	1255,1	640,8	688,1	485,7	0,5	0,2081
RPS22B	420,5	286,3	258,3	168,4	0,6	0,3663
RPS23A	925,5	798,2	785,1	631,6	0,8	0,7919
RPS23B	9,0	11,9	4,2	5,6	0,5	0,4934
RPS24A	25,9	13,2	13,3	10,8	0,5	0,1900
RPS25A	1262,2	553,4	1065,1	438,2	0,8	0,5967
RPS25B	28,1	35,8	4,9	6,1	0,2	0,2497
RPS26B	5083,0	3351,5	1156,5	747,5	0,2	0,0622
RPS27A	19,0	15,2	11,0	11,3	0,6	0,4345
RPS27B	3858,3	2575,0	2689,8	1547,0	0,7	0,4662
RPS28A	28,5	12,0	66,1	58,2	2,3	0,2528
RPS29A	26,1	16,2	10,9	10,0	0,4	0,1605
RPS29B	2,3	3,2	1,3	2,6	0,6	0,6622
RPS3	1,0	1,2	0,0	0,0	0,0	0,1389
RPS30A	1429,8	908,9	1327,7	693,3	0,9	0,8641
RPS30B	1403,7	161,7	886,2	89,9	0,6	0,0014
RPS4A	764,4	581,7	835,0	539,6	1,1	0,8646
RPS4B	0,5	0,9	4,4	3,2	9,6	0,0548
RPS6A	200,1	100,8	138,6	115,4	0,7	0,4522
RPS6B	10,0	5,7	6,8	4,9	0,7	0,4378
RPS7A	1221,3	843,8	709,3	678,8	0,6	0,3808
RPS7B	1508,7	1084,0	611,9	411,5	0,4	0,1728
RPS8A	67,5	75,4	12,4	9,6	0,2	0,1972
RPS8B	868,4	328,3	580,8	268,0	0,7	0,2235
RPS9A	236,5	152,6	278,9	150,4	1,2	0,7054
RPS9B	658,6	599,6	455,4	340,2	0,7	0,5769
RPT1	0,0	0,0	0,0	0,0	#DIV/0!	#DIV/0!
RPT2	153,3	31,2	128,5	86,5	0,8	0,6087
RPT3	75,4	49,3	94,2	87,6	1,2	0,7209
RPT4	81,7	61,4	91,8	76,9	1,1	0,8441

RPT5	46,4	26,9	58,5	51,8	1,3	0,6938
RPT6	20,8	21,4	11,8	14,9	0,6	0,5199
RRB1	93,2	49,3	123,9	97,7	1,3	0,5960
RRD1	5,9	7,8	3,0	5,9	0,5	0,5742
RRD2	28,9	17,9	41,7	36,7	1,4	0,5544
RRF1	10,3	15,6	2,6	5,3	0,3	0,3911
RRG1	10,2	7,4	6,6	13,2	0,6	0,6510
RRG7	84,9	70,4	78,9	74,7	0,9	0,9102
RRG8	11,3	9,3	12,2	12,2	1,1	0,9150
RRG9	3,8	0,6	4,4	5,3	1,2	0,8162
RRI1	19,0	6,5	36,7	27,3	1,9	0,2531
RRI2	6,4	7,9	26,8	25,9	4,2	0,1827
RRM3	44,7	15,2	43,3	44,2	1,0	0,9514
RRN10	27,8	3,9	25,6	18,0	0,9	0,8148
RRN11	65,1	18,8	38,6	27,1	0,6	0,1595
RRN3	48,1	31,1	36,2	28,9	0,8	0,5956
RRN5	248,2	174,5	430,7	333,9	1,7	0,3700
RRN6	17,7	8,1	18,8	15,8	1,1	0,9092
RRN7	12,5	4,2	3,6	5,6	0,3	0,0442
RRN9	25,8	17,2	15,8	15,8	0,6	0,4266
RRP1	71,7	26,2	56,6	44,6	0,8	0,5805
RRP12	18,6	10,3	22,9	20,9	1,2	0,7255
RRP14	589,3	546,5	772,2	710,5	1,3	0,6974
RRP15	609,5	374,6	525,6	367,2	0,9	0,7599
RRP3	115,0	67,6	73,4	68,4	0,6	0,4204
RRP36	53,0	30,4	46,1	32,3	0,9	0,7644
RRP4	453,7	238,4	173,7	79,2	0,4	0,0673
RRP40	2,9	2,0	5,9	4,8	2,0	0,2928
RRP42	19,9	10,4	7,4	8,9	0,4	0,1181
RRP43	10,1	6,0	4,1	2,9	0,4	0,1243
RRP45	198,1	48,0	150,1	106,2	0,8	0,4415
RRP46	8,7	2,6	15,6	16,6	1,8	0,4436
RRP5	111,7	53,8	91,1	57,5	0,8	0,6195
RRP6	62,6	33,3	34,9	24,4	0,6	0,2284
RRP7	160,0	104,1	184,6	98,6	1,2	0,7426
RRP8	42,9	33,8	37,9	42,4	0,9	0,8599
RRP9	63,8	33,1	70,5	46,4	1,1	0,8209
RRT1	40,8	57,5	17,8	15,1	0,4	0,4690
RRT12	514,4	254,8	257,3	185,0	0,5	0,1535
RRT13	49,7	15,6	197,3	136,8	4,0	0,0757
RRT14	68,3	60,0	63,4	60,4	0,9	0,9110
RRT2	65,9	20,2	81,9	83,6	1,2	0,7218
RRT5	3,4	4,1	1,0	2,0	0,3	0,3263
RRT6	8,9	11,4	5,4	7,2	0,6	0,6310
RRT7	2,3	4,6	0,0	0,0	0,0	0,3559
RRT8	20,3	10,8	24,9	22,6	1,2	0,7280
RSA1	13,1	9,4	11,7	12,4	0,9	0,8616
RSA3	86,7	73,7	43,3	41,1	0,5	0,3439
RSA4	43,7	16,7	41,8	29,8	1,0	0,9127
RSB1	72,2	44,0	177,7	121,2	2,5	0,1528

RSC1	217,1	147,8	78,1	53,0	0,4	0,1271
RSC2	10,8	1,7	7,4	7,1	0,7	0,3900
RSC3	16,3	8,1	11,4	11,5	0,7	0,5135
RSC30	3,8	4,5	3,0	5,9	0,8	0,8385
RSC4	31,8	7,1	12,9	10,0	0,4	0,0213
RSC58	163,4	50,7	95,1	67,0	0,6	0,1548
RSC6	33,2	36,3	28,8	31,7	0,9	0,8618
RSC8	16,1	13,2	10,1	9,9	0,6	0,4968
RSC9	28,5	4,3	15,5	12,8	0,5	0,1025
RSE1	26,7	22,4	33,3	24,3	1,2	0,7042
RSF1	42,2	19,0	38,7	26,7	0,9	0,8414
RSF2	50,2	12,2	30,5	26,0	0,6	0,2201
RSM10	0,0	0,0	1,3	2,5	#DIV/0!	0,3559
RSM18	120,5	63,2	229,9	163,9	1,9	0,2592
RSM19	28,6	15,1	22,0	16,4	0,8	0,5742
RSM22	151,5	34,6	88,2	71,9	0,6	0,1635
RSM23	49,4	25,0	143,3	134,6	2,9	0,2193
RSM24	22,1	12,8	13,2	9,2	0,6	0,3026
RSM25	99,3	48,9	230,9	158,2	2,3	0,1631
RSM26	46,3	27,4	15,4	10,8	0,3	0,0807
RSM27	1,5	3,0	0,0	0,0	0,0	0,3559
RSM28	32,5	18,8	11,4	11,1	0,4	0,1025
RSN1	40,5	14,5	19,7	20,8	0,5	0,1521
RSP5	205,4	124,5	108,3	95,0	0,5	0,2614
RTA1	171,5	127,8	74,7	58,4	0,4	0,2174
RTC1	36,0	7,3	74,2	30,2	2,1	0,0488
RTC2	31,4	34,8	27,6	27,2	0,9	0,8677
RTC3	57,7	17,6	510,6	290,2	8,8	0,0207
RTC4	7,9	6,4	14,3	11,2	1,8	0,3649
RTC5	73,6	23,3	113,3	90,1	1,5	0,4263
RTC6	27,5	5,4	16,6	12,5	0,6	0,1619
RTF1	67,8	14,4	41,7	28,3	0,6	0,1506
RTG1	44,1	8,4	33,2	25,9	0,8	0,4541
RTG2	4472,5	1033,5	1922,4	434,2	0,4	0,0039
RTG3	64,2	41,7	76,8	69,7	1,2	0,7661
RTK1	20,7	17,7	24,8	25,6	1,2	0,8005
RTN1	289,4	155,7	264,5	207,0	0,9	0,8540
RTN2	15,7	10,7	25,2	19,9	1,6	0,4313
RTR1	4,6	4,1	3,4	2,3	0,8	0,6492
RTR2	271,0	157,3	295,9	257,5	1,1	0,8745
RTS1	170,5	132,0	116,9	102,2	0,7	0,5440
RTS2	1,0	2,0	2,5	5,1	2,6	0,5913
RTS3	27,1	18,0	23,1	19,9	0,9	0,7752
RTT10	14,5	18,0	7,2	5,4	0,5	0,4658
RTT101	8,9	3,7	6,3	6,4	0,7	0,5033
RTT102	79,1	56,3	71,4	73,6	0,9	0,8722
RTT103	27,4	15,7	94,3	131,0	3,4	0,3492
RTT105	65,4	59,5	22,2	17,4	0,3	0,2122
RTT106	26,2	26,7	5,9	4,9	0,2	0,1841
RTT107	334,1	286,8	49,6	34,2	0,1	0,0963

RTT109	117,6	59,9	29,7	36,0	0,3	0,0455
RUD3	67,1	36,3	194,4	284,8	2,9	0,4092
RUP1	32,9	11,6	27,1	27,6	0,8	0,7089
RVB1	46,2	16,2	55,7	55,9	1,2	0,7547
RVB2	93,7	43,1	65,0	47,0	0,7	0,4024
RVS161	119,1	19,0	96,3	66,6	0,8	0,5358
RVS167	112,3	54,3	167,9	118,2	1,5	0,4252
RXT2	21,6	17,6	20,9	18,2	1,0	0,9579
RXT3	78,7	81,8	69,2	48,3	0,9	0,8483
SAC1	89,2	52,3	118,1	79,1	1,3	0,5647
SAC3	68,5	49,8	78,9	60,3	1,2	0,7989
SAC6	86,0	73,3	189,2	110,2	2,2	0,1700
SAC7	164,3	51,3	112,6	76,9	0,7	0,3059
SAD1	12,4	6,2	6,5	5,2	0,5	0,1996
SAF1	17,0	9,6	8,1	9,9	0,5	0,2416
SAG1	5,3	4,4	18,4	15,1	3,5	0,1466
SAH1	434,2	158,8	264,9	65,0	0,6	0,0960
SAK1	25,5	7,7	14,8	10,0	0,6	0,1415
SAL1	20,6	16,0	15,9	11,2	0,8	0,6444
SAM1	261,4	198,5	340,8	158,1	1,3	0,5545
SAM2	64,0	27,2	137,7	98,4	2,2	0,1987
SAM3	45,6	17,7	53,8	41,8	1,2	0,7294
SAM35	38,8	15,0	21,9	16,9	0,6	0,1847
SAM4	607,4	301,6	381,9	60,1	0,6	0,1929
SAM50	38,5	33,3	29,6	30,1	0,8	0,7057
SAN1	145,2	125,5	172,3	122,2	1,2	0,7672
SAP1	16,7	4,9	10,2	7,9	0,6	0,2105
SAP155	43,5	35,6	27,5	18,7	0,6	0,4571
SAP185	66,7	13,2	43,0	33,2	0,6	0,2323
SAP190	158,9	101,7	60,1	44,8	0,4	0,1257
SAP30	6,9	8,6	4,0	3,0	0,6	0,5511
SAP4	73,2	42,5	115,5	90,2	1,6	0,4288
SAR1	589,8	165,1	306,0	164,1	0,5	0,0505
SAS10	64,8	69,8	97,2	96,2	1,5	0,6058
SAS2	159,7	74,3	300,6	324,9	1,9	0,4303
SAS3	103,8	61,4	51,4	41,1	0,5	0,2061
SAS4	14,8	13,4	14,4	12,4	1,0	0,9688
SAS5	166,7	60,9	75,0	58,9	0,4	0,0737
SAT4	461,3	265,7	408,8	133,2	0,9	0,7362
SAW1	3,1	2,9	4,1	4,8	1,3	0,7500
SAY1	191,4	102,6	47,5	42,9	0,2	0,0413
SBA1	307,5	102,1	478,1	252,3	1,6	0,2566
SBE2	22,0	8,8	37,7	31,4	1,7	0,3727
SBE22	42,3	18,9	29,8	22,1	0,7	0,4221
SBH1	126,7	105,6	94,4	97,2	0,7	0,6681
SCC2	17,4	8,2	7,2	5,1	0,4	0,0800
SCC4	12,0	6,9	0,0	0,0	0,0	0,0131
SCD5	7,3	0,5	13,2	15,4	1,8	0,4719
SCEI	27,4	36,0	45,0	63,1	1,6	0,6444
SCH9	119,7	62,9	78,1	53,1	0,7	0,3513

SCJ1	48,2	30,6	75,7	53,6	1,6	0,4074
SCL1	112,6	61,9	88,8	83,9	0,8	0,6637
SCM3	17,4	6,6	18,3	14,8	1,1	0,9147
SCM4	152,2	101,0	669,2	707,2	4,4	0,1980
SCO1	37,0	17,5	12,9	14,0	0,3	0,0751
SCO2	37,3	16,8	51,2	36,3	1,4	0,5133
SCP1	0,0	0,0	0,0	0,0	#DIV/0!	#DIV/0!
SCP160	49,3	20,1	37,1	32,3	0,8	0,5449
SCS2	151,3	125,9	154,5	158,5	1,0	0,9760
SCS22	50,8	50,0	68,4	63,5	1,3	0,6799
SCS3	13,1	12,6	6,7	5,4	0,5	0,3825
SCS7	1055,2	529,5	1256,2	651,6	1,2	0,6490
SCT1	28,2	11,4	21,7	18,2	0,8	0,5630
SCW10	146,5	155,9	24,0	21,4	0,2	0,1705
SCW11	84,5	30,3	47,3	37,0	0,6	0,1711
SCW4	96,2	73,0	119,3	110,1	1,2	0,7384
SCY1	96,7	38,8	132,4	111,5	1,4	0,5679
SDA1	409,3	242,2	277,9	217,0	0,7	0,4499
SDC1	20,4	12,8	19,7	21,6	1,0	0,9561
SDC25	4,0	4,0	8,2	5,6	2,1	0,2682
SDH1	15,6	14,3	30,0	24,5	1,9	0,3492
SDH2	11,4	11,5	28,7	30,0	2,5	0,3232
SDH4	67,1	27,3	222,2	126,5	3,3	0,0535
SDL1	8,8	3,4	11,5	10,9	1,3	0,6506
SDO1	350,4	191,3	361,1	59,1	1,0	0,9183
SDP1	9,9	8,3	24,0	21,5	2,4	0,2649
SDS22	20,3	16,4	31,9	28,6	1,6	0,5047
SDS23	127,4	210,4	61,0	47,8	0,5	0,5612
SDS24	90,7	39,4	253,2	229,5	2,8	0,2121
SDS3	26,4	18,6	32,4	23,7	1,2	0,7072
SDT1	407,7	130,9	274,0	49,1	0,7	0,1042
SEC1	30,8	17,2	29,2	22,3	0,9	0,9103
SEC10	28,0	17,5	25,2	17,3	0,9	0,8256
SEC11	15,1	13,8	20,8	19,8	1,4	0,6490
SEC12	16,2	8,1	12,0	10,0	0,7	0,5374
SEC13	252,7	169,0	175,1	119,2	0,7	0,4810
SEC14	53,7	24,2	68,0	67,6	1,3	0,7041
SEC15	3,9	5,8	8,5	12,3	2,2	0,5266
SEC16	140,9	92,5	152,3	122,8	1,1	0,8873
SEC17	21,1	16,9	15,8	12,5	0,8	0,6357
SEC18	49,8	22,4	34,7	24,9	0,7	0,4019
SEC2	33,2	31,2	36,3	30,6	1,1	0,8934
SEC20	75,5	50,1	25,8	21,3	0,3	0,1175
SEC21	148,8	49,5	232,7	123,2	1,6	0,2534
SEC22	30,9	20,9	18,1	12,2	0,6	0,3323
SEC23	46,1	32,3	38,9	37,5	0,8	0,7803
SEC24	150,8	80,9	92,9	96,2	0,6	0,3926
SEC26	223,2	140,0	185,5	127,3	0,8	0,7048
SEC27	57,1	35,7	63,0	47,2	1,1	0,8486
SEC28	663,8	177,0	270,2	41,6	0,4	0,0049

SEC3	68,8	47,3	92,1	78,0	1,3	0,6273
SEC31	68,7	35,0	43,0	40,7	0,6	0,3748
SEC39	5,3	3,2	3,0	5,9	0,6	0,5201
SEC4	279,3	200,5	440,1	444,1	1,6	0,5338
SEC5	16,3	11,7	12,5	16,1	0,8	0,7142
SEC59	11,6	7,3	4,4	3,0	0,4	0,1159
SEC6	22,6	16,9	20,7	17,8	0,9	0,8829
SEC61	186,5	171,3	116,5	110,1	0,6	0,5174
SEC62	275,2	141,5	137,9	133,2	0,5	0,2072
SEC63	196,2	111,0	130,0	90,7	0,7	0,3913
SEC65	18,5	17,0	15,9	18,4	0,9	0,8386
SEC66	20,4	12,8	18,2	21,7	0,9	0,8692
SEC7	62,4	23,5	39,5	28,0	0,6	0,2559
SEC72	110,5	99,4	104,8	94,2	0,9	0,9365
SEC8	25,4	7,1	25,6	26,2	1,0	0,9893
SEC9	38,6	29,0	28,3	25,9	0,7	0,6157
SED1	1913,8	1194,6	3896,4	2313,4	2,0	0,1786
SED4	23,5	16,9	155,4	273,7	6,6	0,3733
SED5	28,9	16,5	24,0	17,4	0,8	0,6968
SEE1	31,5	17,6	10,4	10,3	0,3	0,0840
SEF1	20,8	6,6	24,5	23,6	1,2	0,7734
SEH1	38,2	24,6	12,4	12,6	0,3	0,1117
SEM1	206,3	87,3	368,7	153,1	1,8	0,1150
SEN1	190,7	125,8	74,9	54,4	0,4	0,1420
SEN15	9,5	1,7	9,9	8,7	1,0	0,9378
SEN2	4,4	4,1	9,1	8,0	2,1	0,3319
SEN34	33,9	6,2	9,4	10,9	0,3	0,0078
SEN54	6,7	4,6	1,8	2,3	0,3	0,1041
SEO1	22,8	6,9	11,8	11,7	0,5	0,1556
SER1	1258,9	344,1	788,9	294,2	0,6	0,0832
SER2	93,7	58,6	47,5	37,7	0,5	0,2334
SER3	107,4	99,1	192,1	198,9	1,8	0,4751
SER33	31,0	25,6	43,6	30,6	1,4	0,5516
SES1	303,5	171,2	354,1	188,1	1,2	0,7045
SET1	24,2	18,4	6,3	4,4	0,3	0,1071
SET2	17,4	31,9	43,1	81,8	2,5	0,5809
SET3	46,7	17,8	37,7	30,5	0,8	0,6281
SET4	260,8	202,9	668,1	502,5	2,6	0,1835
SET5	94,9	60,0	66,2	54,9	0,7	0,5069
SET6	279,5	150,0	883,9	623,0	3,2	0,1082
SEY1	45,6	20,2	20,6	18,8	0,5	0,1203
SFA1	174,3	31,4	96,6	67,4	0,6	0,0818
SFB2	76,4	29,9	37,7	28,2	0,5	0,1087
SFB3	55,5	22,9	34,5	24,2	0,6	0,2536
SFC1	2,1	4,3	2,4	2,8	1,1	0,9063
SFG1	33,7	28,7	44,7	37,9	1,3	0,6608
SFH1	27,3	18,6	21,6	15,2	0,8	0,6550
SFH5	158,6	108,8	87,4	71,8	0,6	0,3168
SFI1	33,6	12,2	22,3	17,3	0,7	0,3260
SFK1	254,7	101,1	755,0	690,9	3,0	0,2019

SFL1	21,0	3,8	12,0	10,8	0,6	0,1665
SFP1	55,0	10,9	248,5	349,9	4,5	0,3114
SFT1	8,2	6,4	3,1	2,3	0,4	0,1863
SFT2	396,6	159,6	198,7	150,8	0,5	0,1215
SGA1	11,3	7,8	25,8	18,1	2,3	0,1898
SGD1	19,8	6,4	17,8	12,2	0,9	0,7747
SGE1	41,4	43,1	29,4	25,2	0,7	0,6489
SGF11	357,0	147,1	277,7	90,7	0,8	0,3941
SGF29	31,6	11,9	25,2	22,4	0,8	0,6324
SGF73	40,0	28,4	27,3	27,6	0,7	0,5430
SGM1	85,6	57,8	96,5	76,7	1,1	0,8282
SGN1	54,4	37,5	106,0	88,1	1,9	0,3220
SGO1	26,1	14,9	9,2	9,1	0,4	0,1015
SGS1	11,5	9,1	7,5	5,2	0,6	0,4690
SGT1	445,1	63,9	857,7	210,3	1,9	0,0095
SGT2	235,3	76,8	365,7	111,4	1,6	0,1023
SGV1	16,2	14,1	9,6	12,2	0,6	0,5061
SHC1	7,3	4,6	4,7	9,5	0,6	0,6417
SHE1	1236,1	398,5	559,1	613,9	0,5	0,1138
SHE10	63,0	39,3	81,3	72,1	1,3	0,6703
SHE2	80,0	12,1	80,3	55,1	1,0	0,9924
SHE3	13,1	12,3	13,0	16,0	1,0	0,9864
SHE4	1,9	2,3	109,7	212,1	56,3	0,3487
SHE9	64,0	10,5	123,5	87,5	1,9	0,2255
SHG1	19,9	25,2	30,5	32,9	1,5	0,6252
SHM1	33,0	37,6	43,5	50,3	1,3	0,7500
SHM2	382,5	274,3	229,4	141,1	0,6	0,3590
SHO1	190,1	97,3	59,4	40,5	0,3	0,0478
SHP1	45,6	23,9	111,6	74,0	2,4	0,1406
SHQ1	7,0	4,5	13,1	13,5	1,9	0,4264
SHR3	132,3	97,3	96,4	99,6	0,7	0,6249
SHR5	2,6	3,3	3,0	4,5	1,2	0,8866
SHS1	89,6	59,1	97,4	66,7	1,1	0,8674
SHU1	49,2	42,2	18,8	13,1	0,4	0,2178
SHU2	62,0	2,9	119,6	82,6	1,9	0,2126
SHY1	10,4	10,4	9,3	8,2	0,9	0,8680
SIA1	20,4	16,3	50,5	40,4	2,5	0,2163
SIC1	59,4	66,1	32,1	33,7	0,5	0,4904
SIF2	67,4	40,1	93,4	95,5	1,4	0,6343
SIL1	7,2	6,6	1,3	2,6	0,2	0,1532
SIM1	111,3	67,0	28,4	28,7	0,3	0,0633
SIN3	56,2	27,4	62,6	42,2	1,1	0,8076
SIN4	5,2	4,9	25,8	28,0	5,0	0,1967
SIP1	374,0	138,1	171,4	128,0	0,5	0,0749
SIP18	4,8	7,2	13,5	19,9	2,8	0,4431
SIP2	5,7	3,3	1,6	3,3	0,3	0,1300
SIP3	56,5	24,4	47,2	32,3	0,8	0,6618
SIP4	85,1	25,2	138,3	88,2	1,6	0,2901
SIP5	13,3	10,7	11,4	9,7	0,9	0,8055
SIR1	17,1	8,5	16,4	16,4	1,0	0,9397

SIR2	13,2	5,6	9,0	10,3	0,7	0,4964
SIR3	92,9	60,2	89,1	64,2	1,0	0,9329
SIR4	66,2	57,9	47,2	34,1	0,7	0,5922
SIS1	564,5	283,7	1264,6	493,5	2,2	0,0491
SIS2	36,1	4,3	112,3	136,8	3,1	0,3080
SIT1	613,0	160,1	143,5	95,8	0,2	0,0024
SIT4	60,3	35,2	90,4	62,2	1,5	0,4316
SIW14	14417,3	3665,0	3328,6	945,3	0,2	0,0011
SIZ1	40,4	14,4	24,3	19,4	0,6	0,2319
SKG1	69,4	29,2	17,4	20,3	0,3	0,0264
SKG3	11,7	4,2	5,1	7,9	0,4	0,1932
SKG6	12,8	14,3	1,3	2,5	0,1	0,1634
SKI2	79,6	26,3	77,7	52,3	1,0	0,9497
SKI3	7,3	5,4	6,1	8,1	0,8	0,8006
SKI6	24,9	13,6	13,0	10,3	0,5	0,2146
SKI7	8,3	4,1	4,9	4,4	0,6	0,2960
SKI8	9,5	4,2	4,9	6,7	0,5	0,2833
SKM1	25,3	13,9	12,6	14,5	0,5	0,2528
SKN1	63,0	31,2	122,1	76,1	1,9	0,2005
SKN7	45,2	29,5	46,7	70,2	1,0	0,9702
SKO1	109,8	40,1	78,4	59,1	0,7	0,4120
SKP1	38,3	17,1	18,3	13,0	0,5	0,1114
SKP2	18,9	10,4	12,0	8,7	0,6	0,3489
SKS1	28,6	12,1	16,2	13,6	0,6	0,2199
SKT5	26,4	14,8	34,1	26,2	1,3	0,6253
SKY1	21,7	5,6	13,4	9,3	0,6	0,1779
SLA1	27,1	20,3	23,4	22,5	0,9	0,8183
SLA2	47,6	21,9	57,4	38,8	1,2	0,6759
SLC1	15,9	14,7	12,4	14,0	0,8	0,7450
SLD2	64,6	25,2	19,8	23,1	0,3	0,0391
SLD3	5,7	5,9	16,8	15,3	2,9	0,2263
SLD5	39,3	34,3	22,4	33,7	0,6	0,5088
SLF1	7,2	4,6	10,9	8,1	1,5	0,4630
SLG1	29,2	37,0	42,4	44,7	1,5	0,6636
SLH1	17,8	5,1	13,2	11,4	0,7	0,4874
SLI1	25,0	18,1	25,1	19,6	1,0	0,9932
SLI15	44,4	24,5	35,7	29,2	0,8	0,6624
SLK19	274,6	98,0	134,5	84,4	0,5	0,0735
SLM1	22,1	15,5	76,6	105,8	3,5	0,3472
SLM2	3,7	4,7	3,6	4,6	1,0	0,9855
SLM3	21,5	7,0	37,4	29,2	1,7	0,3300
SLM4	34,0	30,4	51,1	67,3	1,5	0,6594
SLM5	41,7	10,6	259,5	241,2	6,2	0,1212
SLN1	24,9	21,1	24,3	13,6	1,0	0,9653
SLO1	6,6	1,8	34,6	7,7	5,3	0,0004
SLP1	22,1	16,6	18,2	12,4	0,8	0,7144
SLS1	0,0	0,0	1,3	2,5	#DIV/0!	0,3559
SLT2	36,3	34,4	24,6	22,8	0,7	0,5934
SLU7	82,4	27,8	65,8	45,7	0,8	0,5574
SLX1	23,0	7,4	22,6	16,8	1,0	0,9680

SLX4	19,1	11,5	64,6	60,4	3,4	0,1900
SLX5	14,3	9,0	21,1	14,1	1,5	0,4492
SLX8	21,3	13,9	11,1	9,2	0,5	0,2660
SLX9	39,2	32,7	24,6	24,3	0,6	0,4989
SLY1	48,0	24,3	35,2	24,5	0,7	0,4858
SLY41	68,8	28,5	56,4	41,8	0,8	0,6416
SLZ1	58,4	30,3	97,9	84,5	1,7	0,4136
SMA1	2,4	4,7	0,7	1,3	0,3	0,5144
SMA2	252,8	72,2	107,5	76,7	0,4	0,0329
SMB1	152,2	66,2	62,4	43,9	0,4	0,0644
SMC1	124,7	82,2	38,0	27,1	0,3	0,0919
SMC2	60,5	44,3	24,0	30,9	0,4	0,2252
SMC3	76,8	46,4	14,7	20,5	0,2	0,0498
SMC4	243,9	77,3	101,9	74,7	0,4	0,0384
SMC5	87,2	26,6	76,7	73,5	0,9	0,7964
SMC6	138,6	82,4	38,8	27,1	0,3	0,0611
SMD2	5,9	7,6	8,1	13,3	1,4	0,7839
SMD3	34,3	20,6	21,3	23,8	0,6	0,4417
SME1	15,5	4,3	20,2	19,8	1,3	0,6536
SMF1	110,1	4,3	178,0	158,2	1,6	0,4237
SMF2	55,5	42,2	18,7	14,0	0,3	0,1487
SMF3	113,2	35,9	117,3	48,1	1,0	0,8950
SMI1	30,1	15,1	88,3	106,9	2,9	0,3228
SMK1	8,2	8,5	22,2	25,7	2,7	0,3422
SML1	345,0	288,3	383,3	370,5	1,1	0,8757
SMM1	10,5	10,6	6,2	4,2	0,6	0,4825
SMP1	18,0	9,2	4,8	3,8	0,3	0,0371
SMP3	26,3	21,5	22,1	16,0	0,8	0,7654
SMX3	0,6	1,2	0,0	0,0	0,0	0,3559
SMY1	41,5	30,6	44,3	30,0	1,1	0,8988
SMY2	199,1	121,9	304,1	208,5	1,5	0,4178
SNA2	67,3	45,4	205,5	56,1	3,1	0,0087
SNA3	98,8	60,0	205,8	105,3	2,1	0,1278
SNA4	70,3	61,7	61,1	55,7	0,9	0,8326
SNC1	34,9	27,1	26,1	24,6	0,7	0,6473
SNF1	42,7	26,2	24,3	21,8	0,6	0,3216
SNF11	843,5	443,9	972,5	572,4	1,2	0,7339
SNF12	4,3	3,3	4,2	5,0	1,0	0,9723
SNF2	150,1	107,3	149,2	100,9	1,0	0,9898
SNF3	8,4	12,7	6,9	5,5	0,8	0,8321
SNF4	305,0	110,4	573,3	165,0	1,9	0,0354
SNF5	58,8	44,8	41,5	28,0	0,7	0,5371
SNF6	52,6	13,7	234,4	151,7	4,5	0,0542
SNF7	227,7	38,8	239,7	163,2	1,1	0,8903
SNF8	36,1	16,3	22,1	20,5	0,6	0,3259
SNG1	71,6	34,6	22,8	16,2	0,3	0,0433
SNL1	0,0	0,0	0,0	0,0	#DIV/0!	#DIV/0!
SNM1	36,4	33,6	79,8	77,4	2,2	0,3429
SNN1	63,4	45,3	134,6	96,7	2,1	0,2307
SNO3	21,6	13,2	11,0	8,4	0,5	0,2244

SNO4	57,2	31,0	23,0	19,4	0,4	0,1104
SNQ2	185,7	24,5	71,1	53,6	0,4	0,0081
SNT1	43,8	24,3	46,5	36,3	1,1	0,9084
SNT2	16,3	4,9	17,4	12,1	1,1	0,8742
SNT309	6,7	5,8	10,4	14,4	1,6	0,6499
SNU114	2,9	2,3	4,2	6,8	1,4	0,7343
SNU23	10,5	7,1	1,8	3,6	0,2	0,0703
SNU56	6,2	5,2	6,7	7,0	1,1	0,9029
SNU66	73,4	47,3	66,4	44,3	0,9	0,8354
SNU71	48,9	37,5	65,5	57,5	1,3	0,6462
SNX3	52,8	37,7	43,2	47,9	0,8	0,7635
SNX4	0,9	1,1	3,7	4,8	4,2	0,2978
SNX41	54,4	31,4	96,0	78,5	1,8	0,3635
SNZ1	238,1	288,0	358,6	411,7	1,5	0,6485
SNZ2	29,3	14,6	11,2	8,2	0,4	0,0742
SNZ3	77,6	14,3	174,7	97,7	2,3	0,0966
SOD1	201,7	77,4	335,4	187,6	1,7	0,2357
SOD2	9,1	8,1	15,3	16,2	1,7	0,5174
SOF1	247,4	107,5	235,3	258,1	1,0	0,9339
SOG2	4,0	3,9	0,0	0,0	0,0	0,0853
SOH1	93,3	18,7	48,7	37,0	0,5	0,0752
SOK1	19,1	13,3	86,1	110,4	4,5	0,2734
SOK2	118,7	32,5	124,6	83,8	1,0	0,9001
SOL1	27,4	27,3	105,6	141,3	3,9	0,3187
SOL2	16,0	7,2	12,1	10,6	0,8	0,5660
SOL3	64,2	39,8	212,5	258,6	3,3	0,3001
SOL4	25,0	16,0	133,7	34,1	5,4	0,0012
SOP4	65,4	39,3	64,4	70,0	1,0	0,9812
SOR2	11,2	5,3	15,2	12,7	1,4	0,5755
SOV1	6,0	6,1	79,5	149,4	13,3	0,3633
SPA2	98,3	63,1	38,9	31,5	0,4	0,1426
SPB1	288,6	180,7	325,0	138,1	1,1	0,7594
SPB4	9,6	3,0	82,5	138,3	8,6	0,3329
SPC1	8,5	4,3	13,6	9,4	1,6	0,3685
SPC105	39,9	24,1	12,1	13,9	0,3	0,0928
SPC110	159,2	141,8	47,2	35,1	0,3	0,1760
SPC19	53,4	40,2	126,7	131,1	2,4	0,3257
SPC24	9,3	4,5	3,3	6,6	0,4	0,1842
SPC25	36,1	19,9	18,5	17,5	0,5	0,2344
SPC29	8,0	3,4	0,7	1,3	0,1	0,0069
SPC3	3,0	5,9	4,9	5,9	1,6	0,6672
SPC34	18,5	7,1	10,9	10,1	0,6	0,2675
SPC42	29,3	18,6	6,5	4,6	0,2	0,0554
SPC72	19,3	5,8	26,6	17,9	1,4	0,4630
SPC97	10,7	5,5	80,2	93,4	7,5	0,1881
SPC98	13,8	1,8	6,7	6,0	0,5	0,0637
SPE1	21,2	15,2	20,1	21,0	1,0	0,9406
SPE2	227,6	98,2	194,7	75,5	0,9	0,6139
SPE3	114,0	113,2	138,1	152,7	1,2	0,8078
SPE4	34,1	20,6	16,5	19,1	0,5	0,2563

SPF1	116,3	60,1	48,3	37,7	0,4	0,1039
SPG1	5,4	7,0	3,3	6,6	0,6	0,6737
SPG3	446,1	163,1	242,3	68,4	0,5	0,0608
SPG4	74,2	22,0	84,6	57,7	1,1	0,7463
SPG5	63,6	20,1	25,1	19,6	0,4	0,0338
SPH1	2,6	1,8	0,7	1,3	0,3	0,1266
SPI1	99,8	60,4	308,9	90,3	3,1	0,0085
SPL2	15,4	10,8	116,7	104,9	7,6	0,1030
SPN1	83,9	86,5	103,7	104,4	1,2	0,7802
SPO1	62,7	25,3	54,4	49,5	0,9	0,7753
SPO11	28,3	12,7	16,6	16,6	0,6	0,3057
SPO12	172,7	64,7	83,0	71,3	0,5	0,1115
SPO13	47,9	14,2	62,2	44,2	1,3	0,5612
SPO14	5,3	6,1	9,7	11,1	1,8	0,5120
SPO20	6,5	7,0	1,0	2,0	0,2	0,1843
SPO21	20,1	13,1	26,9	21,9	1,3	0,6119
SPO22	74,9	35,6	111,7	107,5	1,5	0,5403
SPO23	14,1	16,8	5,3	10,6	0,4	0,4053
SPO7	4,0	2,7	1,2	2,4	0,3	0,1709
SPO71	2,6	3,2	10,3	12,5	4,0	0,2736
SPO73	45,6	19,4	73,1	80,1	1,6	0,5291
SPO74	24,3	11,0	1,0	2,0	0,0	0,0059
SPO75	702,7	651,8	341,7	366,9	0,5	0,3717
SPO77	40,5	27,6	19,9	19,7	0,5	0,2706
SPP1	64,0	25,9	76,7	53,1	1,2	0,6833
SPP2	1,5	3,0	0,0	0,0	0,0	0,3559
SPP381	16,9	12,2	40,7	39,9	2,4	0,2961
SPP382	26,9	9,5	16,5	11,6	0,6	0,2149
SPP41	246,1	169,8	228,5	168,0	0,9	0,8877
SPR1	65,1	41,3	85,0	59,5	1,3	0,6027
SPR28	18,5	7,7	15,8	23,0	0,9	0,8279
SPR3	16,7	15,0	3,5	4,1	0,2	0,1403
SPR6	145,5	82,5	277,8	292,7	1,9	0,4177
SPS1	50,8	39,3	18,9	24,4	0,4	0,2184
SPS100	5,4	1,5	3,2	3,8	0,6	0,3252
SPS18	1,5	1,8	0,0	0,0	0,0	0,1445
SPS19	7,8	4,3	3,9	5,0	0,5	0,2807
SPS2	35,9	25,8	10,2	17,1	0,3	0,1478
SPS22	1,2	2,4	1,9	2,4	1,6	0,6733
SPS4	0,0	0,0	0,0	0,0	#DIV/0!	#DIV/0!
SPT10	14,5	6,3	128,4	118,4	8,9	0,1030
SPT14	42,7	27,7	16,7	12,9	0,4	0,1401
SPT15	104,4	59,7	70,4	54,6	0,7	0,4322
SPT16	210,7	214,8	180,2	161,6	0,9	0,8280
SPT2	59,4	13,3	143,9	154,2	2,4	0,3170
SPT20	60,3	34,6	65,1	45,7	1,1	0,8725
SPT21	15,6	9,9	86,3	172,5	5,5	0,4448
SPT23	24,8	38,2	10,6	10,0	0,4	0,4969
SPT3	33,2	33,5	26,0	27,1	0,8	0,7479
SPT5	19,1	12,3	23,9	22,7	1,2	0,7239

SPT6	64,0	33,5	53,0	40,1	0,8	0,6885
SPT7	79,1	50,8	88,7	74,8	1,1	0,8385
SPT8	108,9	76,9	129,2	172,1	1,2	0,8362
SQS1	111,9	55,9	177,4	121,0	1,6	0,3638
SQT1	69,4	48,7	59,5	61,4	0,9	0,8086
SRB2	131,2	31,2	78,4	77,7	0,6	0,2536
SRB4	14,2	8,5	5,9	7,1	0,4	0,1835
SRB5	37,2	13,1	34,0	38,8	0,9	0,8805
SRB6	84,2	52,9	63,3	50,9	0,8	0,5888
SRB7	25,0	17,9	9,9	8,7	0,4	0,1785
SRB8	4,4	3,5	7,5	12,5	1,7	0,6543
SRC1	13,0	7,9	11,8	10,9	0,9	0,8586
SRD1	246,8	230,3	217,8	227,4	0,9	0,8636
SRL1	1858,8	968,8	1216,3	616,3	0,7	0,3059
SRL2	10,8	11,0	10,9	9,3	1,0	0,9930
SRL3	11,7	8,6	19,4	16,5	1,7	0,4370
SRL4	2,1	2,8	2,3	2,6	1,1	0,9278
SRM1	181,0	59,5	143,0	95,8	0,8	0,5253
SRN2	22,7	10,7	31,9	25,6	1,4	0,5318
SRO7	5,2	4,8	6,6	5,2	1,3	0,6989
SRO77	37,1	22,0	49,7	49,4	1,3	0,6594
SRO9	14,9	15,2	18,4	18,6	1,2	0,7840
SRP1	178,7	54,9	184,7	217,7	1,0	0,9589
SRP101	18,3	13,5	19,1	17,0	1,0	0,9486
SRP102	88,2	43,6	37,4	29,5	0,4	0,1021
SRP14	92,2	61,9	86,0	64,3	0,9	0,8941
SRP21	7,6	9,7	5,5	6,4	0,7	0,7326
SRP40	200,0	109,4	133,4	107,7	0,7	0,4189
SRP54	18,0	19,6	58,1	90,4	3,2	0,4198
SRP68	187,9	115,8	51,6	36,4	0,3	0,0658
SRP72	115,3	94,9	102,6	87,1	0,9	0,8501
SRS2	45,8	23,7	12,3	16,7	0,3	0,0605
SRT1	4,8	4,4	6,5	5,2	1,4	0,6219
SRV2	162,8	81,6	196,4	74,6	1,2	0,5660
SRX1	4,2	4,6	14,8	18,9	3,5	0,3206
SRY1	100,7	61,3	109,9	96,4	1,1	0,8771
SSA1	802,5	575,8	2149,0	1308,2	2,7	0,1085
SSA2	239,4	204,9	353,5	191,0	1,5	0,4463
SSA3	13,4	8,4	26,2	23,9	2,0	0,3504
SSA4	16,3	11,6	46,7	41,8	2,9	0,2102
SSB1	313,6	214,9	175,6	159,9	0,6	0,3426
SSB2	253,4	40,8	237,4	67,8	0,9	0,7006
SSC1	1557,2	372,4	1848,6	404,3	1,2	0,3297
SSD1	304,0	106,5	563,4	230,0	1,9	0,0866
SSE1	1706,6	1073,1	2661,9	1585,4	1,6	0,3568
SSE2	112,9	36,0	500,1	65,1	4,4	0,0000
SSF1	102,2	85,5	81,5	79,3	0,8	0,7345
SSF2	92,9	80,5	57,2	52,6	0,6	0,4863
SSH4	4,2	5,3	13,1	10,3	3,1	0,1792
SSK1	10,3	11,8	11,2	9,3	1,1	0,9151

SSK2	12,1	10,8	15,7	13,7	1,3	0,6934
SSK22	69,8	19,0	73,9	61,0	1,1	0,9013
SSL1	22,9	17,7	12,8	13,5	0,6	0,3995
SSL2	7,9	3,1	42,2	36,0	5,4	0,1065
SSM4	35,8	26,5	18,6	17,8	0,5	0,3241
SSN2	7,4	11,9	21,2	15,3	2,9	0,2036
SSN3	13,9	9,8	10,1	7,7	0,7	0,5733
SSN8	3,4	2,5	29,1	21,5	8,6	0,0557
SSO1	43,2	31,2	30,0	30,2	0,7	0,5662
SSO2	112,3	102,3	148,8	162,0	1,3	0,7170
SSP1	18,3	36,6	0,0	0,0	0,0	0,3559
SSP120	232,7	22,2	166,2	124,4	0,7	0,3332
SSP2	7,4	7,3	8,5	6,1	1,2	0,8164
SSQ1	19,2	10,0	15,3	15,6	0,8	0,6890
SST2	26,2	4,2	296,3	393,6	11,3	0,2190
SSU1	87,8	62,9	84,0	79,2	1,0	0,9432
SSU72	43,3	16,0	178,0	269,4	4,1	0,3567
SSY1	16,1	5,6	23,4	20,3	1,5	0,5097
SSY5	44,2	19,6	56,4	50,4	1,3	0,6684
SSZ1	66,2	60,8	61,9	62,2	0,9	0,9243
STB1	11,4	16,7	8,5	10,6	0,7	0,7755
STB2	159,3	72,3	118,7	132,6	0,7	0,6107
STB3	88,2	41,2	203,9	96,2	2,3	0,0690
STB4	40,5	19,6	47,4	36,9	1,2	0,7511
STB5	55,6	44,2	10,7	9,8	0,2	0,0945
STB6	5,1	5,0	20,9	25,0	4,1	0,2599
STD1	28,6	14,8	81,5	102,2	2,9	0,3448
STE11	15,0	4,1	10,1	7,7	0,7	0,3130
STE12	60,5	26,2	189,0	161,1	3,1	0,1665
STE13	19,3	18,0	25,3	20,0	1,3	0,6684
STE14	125,6	89,2	287,4	272,6	2,3	0,3024
STE18	0,0	0,0	0,0	0,0	#DIV/0!	#DIV/0!
STE2	485,0	286,6	983,5	413,9	2,0	0,0950
STE20	40,1	43,7	62,6	53,4	1,6	0,5393
STE23	107,4	52,2	53,3	45,5	0,5	0,1693
STE24	78,3	29,4	297,1	311,3	3,8	0,2111
STE3	0,0	0,0	1,3	2,5	#DIV/0!	0,3559
STE4	171,4	69,3	386,0	387,2	2,3	0,3170
STE5	45,6	33,9	49,7	39,3	1,1	0,8793
STE50	9,4	6,5	3,2	6,3	0,3	0,2203
STE6	79,9	66,3	240,4	101,9	3,0	0,0386
STE7	2,7	3,3	0,0	0,0	0,0	0,1558
STF1	154,1	70,4	398,9	269,4	2,6	0,1292
STF2	142,3	80,0	340,1	172,5	2,4	0,0826
STH1	82,1	41,9	83,0	59,0	1,0	0,9800
STI1	259,0	95,0	959,5	496,4	3,7	0,0323
STL1	97,5	64,4	201,4	183,9	2,1	0,3272
STM1	1385,2	744,4	1637,7	764,3	1,2	0,6528
STN1	10,8	4,1	12,3	9,3	1,1	0,7755
STO1	19,0	16,9	17,2	12,7	0,9	0,8722

STP1	58,0	11,8	110,1	50,7	1,9	0,0918
STP2	8,7	7,1	12,1	14,6	1,4	0,6835
STP22	11,2	11,0	15,0	12,9	1,3	0,6710
STP3	30,2	9,6	20,2	20,8	0,7	0,4159
STP4	77,3	59,2	61,7	43,9	0,8	0,6866
STR2	17,6	8,2	6,8	5,9	0,4	0,0770
STR3	14,8	10,2	13,3	9,8	0,9	0,8435
STS1	37,1	11,5	20,5	14,9	0,6	0,1280
STT3	167,1	146,5	157,8	86,8	0,9	0,9165
STT4	16,3	8,9	134,6	259,9	8,3	0,3977
STU1	17,2	10,9	9,0	8,2	0,5	0,2722
STU2	148,0	72,2	184,7	69,2	1,2	0,4910
STV1	85,0	21,5	88,8	61,5	1,0	0,9115
SUA5	36,7	9,7	49,2	40,6	1,3	0,5726
SUA7	36,6	37,5	38,9	41,3	1,1	0,9353
SUB1	105,9	63,5	142,2	108,2	1,3	0,5842
SUB2	214,7	175,4	151,5	162,4	0,7	0,6157
SUC2	18,4	9,9	16,9	16,4	0,9	0,8743
SUE1	59,5	87,7	75,2	134,0	1,3	0,8517
SUI1	171,9	104,9	193,9	187,0	1,1	0,8438
SUI2	223,5	113,4	252,6	202,4	1,1	0,8103
SUL1	174,6	111,7	133,4	142,4	0,8	0,6646
SUL2	60,2	55,6	156,6	114,4	2,6	0,1805
SUM1	246,2	132,5	274,2	87,2	1,1	0,7364
SUN4	367,0	293,0	226,6	175,1	0,6	0,4420
SUP35	87,4	54,6	240,7	237,4	2,8	0,2551
SUP45	273,7	119,3	343,4	232,9	1,3	0,6132
SUR1	79,4	28,8	42,2	31,3	0,5	0,1312
SUR2	41,8	23,2	18,3	19,0	0,4	0,1686
SUR4	398,9	171,5	171,9	57,0	0,4	0,0458
SUR7	227,9	143,6	143,4	133,6	0,6	0,4218
SUS1	0,0	0,0	0,0	0,0	#DIV/0!	#DIV/0!
SUT1	19,6	8,4	31,3	30,8	1,6	0,4915
SUT2	131,9	107,2	15,0	21,2	0,1	0,0764
SUV3	24,2	12,5	9,9	11,5	0,4	0,1453
SVF1	215,3	166,1	194,5	185,8	0,9	0,8733
SVL3	315,9	62,3	341,8	121,0	1,1	0,7166
SVP26	187,5	104,2	210,7	172,6	1,1	0,8254
SWA2	27,4	16,9	25,0	21,4	0,9	0,8699
SWC3	79,8	57,3	94,7	81,6	1,2	0,7747
SWC4	39,7	18,2	70,1	56,0	1,8	0,3412
SWC5	15,5	6,1	19,2	15,6	1,2	0,6725
SWD1	31,4	19,7	24,5	19,7	0,8	0,6344
SWD2	8,4	4,2	11,0	12,0	1,3	0,6991
SWE1	30,1	28,4	31,4	32,2	1,0	0,9552
SWF1	115,5	144,4	432,0	582,5	3,7	0,3322
SWH1	80,1	60,1	162,7	135,2	2,0	0,3066
SWI1	65,9	25,9	45,8	63,9	0,7	0,5811
SWI3	225,6	212,5	127,5	110,4	0,6	0,4438
SWI4	383,0	119,5	69,4	50,9	0,2	0,0029

SWI5	15,2	16,4	7,8	7,7	0,5	0,4445
SWI6	31,8	15,6	103,4	179,7	3,3	0,4573
SWM1	27,4	7,9	78,4	64,1	2,9	0,1651
SWM2	67,3	25,0	136,2	140,4	2,0	0,3711
SWP1	36,0	26,7	33,9	33,7	0,9	0,9279
SWP82	10,4	5,0	6,7	7,8	0,6	0,4610
SWR1	28,9	20,4	56,7	42,0	2,0	0,2784
SWS2	17,8	12,7	18,2	21,7	1,0	0,9699
SWT1	40,9	28,6	11,8	13,8	0,3	0,1160
SWT21	4,0	4,7	12,1	9,8	3,0	0,1900
SXM1	65,8	34,1	58,5	44,8	0,9	0,8016
SYC1	9,7	9,4	13,5	13,9	1,4	0,6709
SYF1	36,8	29,8	23,2	18,7	0,6	0,4704
SYG1	238,4	72,7	132,9	96,7	0,6	0,1316
SYH1	286,5	196,3	307,0	211,4	1,1	0,8919
SYM1	61,1	32,0	42,5	28,7	0,7	0,4223
SYN8	20,9	12,1	36,0	38,0	1,7	0,4778
SYP1	17,7	5,1	16,7	13,6	0,9	0,8956
SYS1	252,4	189,6	299,3	281,6	1,2	0,7914
SYT1	4,1	1,5	1,3	2,5	0,3	0,1007
TAD1	29,4	24,1	8,3	7,1	0,3	0,1443
TAD2	7,3	5,2	0,7	1,3	0,1	0,0479
TAE1	185,0	150,7	193,8	178,7	1,0	0,9421
TAE2	591,1	470,0	619,0	420,7	1,0	0,9324
TAF1	14,7	8,1	21,4	19,2	1,5	0,5407
TAF10	64,7	45,9	59,9	63,0	0,9	0,9076
TAF11	30,2	13,7	124,4	114,7	4,1	0,1540
TAF14	17,9	15,0	10,9	8,2	0,6	0,4451
TAF2	58,9	13,9	40,9	31,2	0,7	0,3313
TAF3	29,4	14,3	30,5	34,4	1,0	0,9534
TAF4	211,8	176,2	163,0	123,2	0,8	0,6663
TAF5	250,3	50,5	289,4	109,3	1,2	0,5398
TAF6	5,8	5,8	3,4	4,5	0,6	0,5337
TAF7	44,4	28,2	60,4	45,8	1,4	0,5746
TAF8	16,0	10,8	23,7	24,0	1,5	0,5792
TAF9	132,0	68,2	102,6	85,7	0,8	0,6110
TAH1	34,1	18,9	137,0	127,8	4,0	0,1622
TAH11	13,1	6,7	12,2	14,2	0,9	0,9085
TAH18	2,1	2,5	4,5	6,7	2,1	0,5239
TAL1	75,8	77,9	50,5	61,3	0,7	0,6291
TAM41	8,8	12,8	6,7	8,1	0,8	0,7911
TAN1	93,7	57,9	50,0	34,3	0,5	0,2412
TAO3	21,8	13,2	19,4	18,9	0,9	0,8454
TAP42	15,2	7,5	11,2	10,3	0,7	0,5561
TAT1	302,1	198,2	255,1	317,9	0,8	0,8103
TAT2	547,5	209,4	175,4	128,0	0,3	0,0231
TAX4	13,1	7,7	12,7	10,0	1,0	0,9485
TAZ1	15,6	6,1	7,5	6,4	0,5	0,1186
TBF1	26,3	11,8	20,1	15,6	0,8	0,5457
TBS1	8,0	7,2	8,6	8,6	1,1	0,9197

TCB1	103,6	36,7	62,5	52,7	0,6	0,2474
TCB2	180,6	43,1	137,4	95,3	0,8	0,4404
TCB3	83,3	46,1	55,0	38,0	0,7	0,3803
TCM62	43,3	25,1	35,9	25,1	0,8	0,6927
TCO89	23,0	11,1	21,7	15,0	0,9	0,8957
TCP1	125,6	27,0	119,6	80,0	1,0	0,8922
TDH1	46,6	42,6	338,8	353,5	7,3	0,1518
TDH2	725,9	532,3	1553,2	1097,7	2,1	0,2238
TDH3	6049,2	4600,2	9807,6	6309,3	1,6	0,3729
TDP1	0,0	0,0	0,0	0,0	#DIV/0!	#DIV/0!
TEA1	37,9	27,0	182,3	241,5	4,8	0,2797
TEC1	67,9	11,2	199,5	144,7	2,9	0,1196
TED1	144,5	98,7	113,1	93,7	0,8	0,6601
TEF1	465,7	270,9	426,5	319,7	0,9	0,8578
TEF2	6788,2	5277,8	6931,7	4153,0	1,0	0,9673
TEF4	1379,3	631,5	1695,0	791,0	1,2	0,5557
TEL1	93,6	12,2	38,4	26,2	0,4	0,0087
TEL2	26,3	23,2	14,0	9,5	0,5	0,3654
TEM1	31,0	12,3	12,9	12,3	0,4	0,0824
TEP1	2,5	2,0	0,7	1,3	0,3	0,1797
TES1	3,0	4,6	4,5	6,0	1,5	0,7136
TEX1	7,4	5,0	3,4	4,5	0,5	0,2735
TFA1	776,0	611,4	647,7	535,2	0,8	0,7628
TFB1	52,1	21,9	124,6	74,6	2,4	0,1114
TFB2	24,2	8,6	11,1	8,8	0,5	0,0765
TFB3	124,5	31,4	211,6	88,2	1,7	0,1119
TFB4	151,8	74,2	249,6	59,4	1,6	0,0851
TFB5	270,8	197,5	177,9	160,2	0,7	0,4926
TFC1	7,4	4,0	8,5	7,3	1,1	0,8019
TFC3	16,7	4,2	13,2	8,9	0,8	0,5017
TFC4	57,5	35,1	33,0	22,6	0,6	0,2859
TFC6	55,8	17,2	284,2	316,0	5,1	0,1991
TFC7	80,2	56,0	50,9	34,1	0,6	0,4057
TFC8	13,8	4,0	14,2	9,6	1,0	0,9417
TFG1	213,3	18,5	231,0	30,1	1,1	0,3558
TFG2	58,3	41,6	78,1	64,7	1,3	0,6252
TFS1	75,6	25,6	114,4	93,0	1,5	0,4522
TGL1	130,4	96,0	73,5	51,4	0,6	0,3363
TGL2	19,6	15,3	26,4	21,9	1,3	0,6324
TGL3	20,1	6,2	4,3	8,6	0,2	0,0241
TGL4	33,1	11,0	57,9	40,7	1,7	0,2843
TGL5	68,0	33,6	35,3	26,1	0,5	0,1750
TGS1	33,5	29,1	13,9	10,9	0,4	0,2534
THG1	2,6	3,1	2,4	4,7	0,9	0,9321
THI11	32,3	6,4	26,2	18,6	0,8	0,5568
THI13	16,8	2,7	2,3	4,6	0,1	0,0016
THI2	23,2	29,0	104,2	134,0	4,5	0,2818
THI20	47,8	27,5	74,3	75,4	1,6	0,5342
THI21	9,1	7,6	21,3	33,2	2,3	0,5012
THI22	16,5	7,4	15,9	14,1	1,0	0,9473

THI3	53,6	31,9	47,6	46,8	0,9	0,8409
THI4	35,3	13,3	90,9	99,6	2,6	0,3114
THI5	0,0	0,0	0,0	0,0	#DIV/0!	#DIV/0!
THI6	34,1	19,7	15,6	13,5	0,5	0,1719
THI7	56,5	39,7	118,9	84,8	2,1	0,2310
THI72	8,6	14,3	5,9	4,4	0,7	0,7351
THI73	65,5	25,7	97,3	71,5	1,5	0,4350
THI74	10,1	8,9	53,0	32,3	5,3	0,0428
THI80	61,8	13,5	22,8	16,5	0,4	0,0106
THO1	2173,4	884,9	930,5	265,1	0,4	0,0360
THO2	14,5	9,7	29,0	19,9	2,0	0,2380
THP1	9,1	6,2	6,4	7,1	0,7	0,5853
THP2	3,4	3,9	1,9	3,8	0,6	0,6065
THP3	11,0	7,4	18,7	20,0	1,7	0,4942
THR1	101,6	88,5	118,1	126,6	1,2	0,8387
THR4	258,9	275,5	288,0	295,4	1,1	0,8900
THS1	199,3	166,6	302,0	321,7	1,5	0,5915
TID3	77,7	33,3	57,6	40,0	0,7	0,4681
TIF11	8,3	7,7	19,3	17,7	2,3	0,2970
TIF2	619,7	300,6	592,3	583,5	1,0	0,9363
TIF3	1054,2	493,2	445,5	307,3	0,4	0,0811
TIF34	296,3	153,5	274,5	115,3	0,9	0,8276
TIF35	369,4	327,5	380,5	371,4	1,0	0,9655
TIF4631	148,4	95,9	253,5	77,6	1,7	0,1393
TIF4632	101,7	49,1	49,4	44,6	0,5	0,1664
TIF5	225,3	72,9	340,2	419,3	1,5	0,6085
TIF6	38,0	29,4	45,6	45,7	1,2	0,7890
TIM10	172,9	63,0	121,7	93,7	0,7	0,3995
TIM12	26,4	10,1	9,3	9,7	0,4	0,0501
TIM17	95,7	47,1	149,2	99,2	1,6	0,3679
TIM18	153,4	49,4	184,5	132,4	1,2	0,6754
TIM21	68,1	55,4	49,4	48,3	0,7	0,6282
TIM22	36,0	21,0	20,9	18,2	0,6	0,3192
TIM23	551,5	129,8	238,2	179,8	0,4	0,0302
TIM44	61,4	59,2	84,2	59,7	1,4	0,6061
TIM50	402,7	146,7	490,5	73,7	1,2	0,3258
TIM54	44,1	9,1	101,2	72,3	2,3	0,1682
TIP1	117,6	102,9	364,2	310,8	3,1	0,1827
TIP20	40,0	25,1	22,3	15,2	0,6	0,2716
TIP41	35,4	11,9	22,1	18,7	0,6	0,2741
TIR1	13,5	8,8	12,4	9,7	0,9	0,8665
TIR2	0,9	1,8	0,0	0,0	0,0	0,3559
TIR3	76,0	32,3	43,3	32,5	0,6	0,2034
TIR4	6,8	8,2	0,0	0,0	0,0	0,1489
TIS11	36,8	17,9	12,7	10,0	0,3	0,0564
TKL1	384,0	166,9	312,5	251,3	0,8	0,6523
TKL2	85,8	53,1	66,5	58,4	0,8	0,6416
TLG1	35,6	18,0	90,0	97,3	2,5	0,3138
TLG2	20,7	6,2	6,1	9,6	0,3	0,0433
TMA10	25,1	10,2	25,7	25,1	1,0	0,9642

TMA108	6,1	6,8	31,3	32,4	5,2	0,1786
TMA16	2,0	4,0	0,0	0,0	0,0	0,3559
TMA17	47,1	9,6	68,5	57,3	1,5	0,4884
TMA19	1587,7	745,7	3167,7	907,1	2,0	0,0360
TMA20	192,7	136,6	319,4	347,8	1,7	0,5230
TMA22	390,8	305,9	735,2	584,4	1,9	0,3366
TMA23	96,2	61,9	149,1	75,7	1,5	0,3209
TMA46	202,3	82,6	352,9	38,8	1,7	0,0164
TMA64	40,2	15,9	66,9	56,9	1,7	0,4003
TMA7	269,5	135,4	371,6	259,2	1,4	0,5110
TMN2	19,2	2,8	20,9	15,3	1,1	0,8412
TMN3	72,0	12,3	49,4	37,8	0,7	0,2993
TMS1	92,6	41,6	105,2	69,5	1,1	0,7666
TMT1	15,1	11,1	56,8	57,6	3,8	0,2050
TNA1	43,3	29,1	40,9	36,9	0,9	0,9210
TOA1	34,4	31,4	43,5	35,0	1,3	0,7105
TOA2	39,8	9,5	57,0	43,8	1,4	0,4734
TOD6	16,8	16,9	15,2	17,7	0,9	0,9063
TOF1	54,2	28,3	18,0	18,2	0,3	0,0745
TOF2	56,0	42,9	21,9	20,3	0,4	0,2004
TOK1	8,0	6,1	4,1	8,3	0,5	0,4769
TOM1	63,5	12,2	35,7	23,9	0,6	0,0830
TOM20	66,4	51,5	38,7	40,6	0,6	0,4310
TOM22	104,1	47,1	162,8	56,7	1,6	0,1627
TOM40	56,4	29,7	53,9	41,1	1,0	0,9250
TOM6	75,8	68,8	85,6	82,2	1,1	0,8602
TOM7	1097,9	71,2	313,6	121,8	0,3	0,0000
TOM70	41,9	38,1	59,4	61,6	1,4	0,6452
TOM71	21,2	12,6	7,1	5,2	0,3	0,0854
TOP1	437,1	233,8	148,3	105,7	0,3	0,0653
TOP2	140,1	84,8	34,5	25,5	0,2	0,0544
TOP3	26,5	26,8	27,8	27,0	1,0	0,9470
TOR1	114,6	74,1	104,4	107,8	0,9	0,8809
TOR2	21,8	10,9	11,9	10,7	0,5	0,2393
TOS1	540,5	105,4	465,6	119,9	0,9	0,3843
TOS2	49,3	13,4	398,2	746,7	8,1	0,3862
TOS3	29,9	12,6	6,1	7,9	0,2	0,0184
TOS4	41,9	40,0	0,0	0,0	0,0	0,0812
TOS6	586,7	384,3	20,6	18,0	0,0	0,0258
TOS8	48,1	10,6	179,4	164,7	3,7	0,1626
TPA1	179,1	72,8	136,7	111,9	0,8	0,5492
TPC1	134,1	72,0	497,5	183,3	3,7	0,0102
TPD3	18,0	9,9	23,1	15,8	1,3	0,6101
TPK1	90,7	104,4	191,3	150,3	2,1	0,3140
TPK2	27,5	13,3	33,1	22,4	1,2	0,6833
TPK3	288,8	193,2	217,6	154,6	0,8	0,5860
TPM1	1540,3	335,0	2226,6	663,1	1,4	0,1142
TPM2	78,2	47,3	64,1	63,6	0,8	0,7342
TPN1	90,5	43,0	42,9	42,1	0,5	0,1647
TPO1	131,6	40,5	144,8	80,8	1,1	0,7801

TPO2	9,1	12,6	41,0	44,3	4,5	0,2157
TPO3	244,1	106,3	309,1	199,5	1,3	0,5863
TPO4	31,2	15,2	227,2	391,5	7,3	0,3558
TPO5	17,7	17,6	15,1	15,2	0,8	0,8265
TPP1	60,3	17,9	66,3	75,2	1,1	0,8822
TPS1	99,2	56,9	203,2	81,2	2,0	0,0808
TPS2	44,6	18,5	170,2	98,9	3,8	0,0467
TPS3	43,6	30,7	82,0	79,9	1,9	0,4040
TPT1	16,7	3,2	5,9	7,1	0,4	0,0318
TRA1	13,4	6,1	17,7	12,8	1,3	0,5693
TRE1	71,3	19,1	71,2	57,3	1,0	0,9951
TRE2	8,9	10,3	10,0	9,8	1,1	0,8810
TRF5	23,3	7,0	47,7	33,0	2,1	0,1965
TRI1	77,1	58,0	224,7	93,6	2,9	0,0364
TRK1	136,2	109,8	101,4	94,0	0,7	0,6476
TRK2	12,8	4,7	9,6	9,3	0,8	0,5678
TRL1	9,5	7,3	9,3	8,1	1,0	0,9751
TRM1	54,3	45,8	44,5	42,1	0,8	0,7637
TRM10	55,3	8,5	59,2	54,8	1,1	0,8949
TRM11	55,3	25,7	13,8	9,5	0,2	0,0231
TRM112	177,9	94,9	244,0	54,0	1,4	0,2714
TRM12	13,8	18,0	8,1	5,8	0,6	0,5704
TRM13	21,5	14,1	27,7	28,9	1,3	0,7125
TRM2	11,0	5,7	9,6	9,3	0,9	0,8044
TRM3	34,9	5,7	60,8	42,8	1,7	0,2743
TRM44	6,5	6,2	11,2	14,9	1,7	0,5777
TRM5	1,0	2,0	6,8	5,9	6,8	0,1122
TRM7	276,3	88,6	348,1	152,6	1,3	0,4472
TRM8	237,5	156,4	320,2	247,3	1,3	0,5923
TRM82	31,3	20,9	11,9	8,0	0,4	0,1333
TRM9	17,0	19,2	11,1	11,4	0,7	0,6170
TRP2	82,6	67,4	121,7	114,6	1,5	0,5776
TRP3	1332,3	550,7	1021,7	730,0	0,8	0,5223
TRP4	20,0	14,0	10,9	13,7	0,5	0,3879
TRP5	61,2	26,0	88,9	90,4	1,5	0,5777
TRR1	346,4	115,6	389,8	21,0	1,1	0,4884
TRS120	210,2	80,9	56,5	43,5	0,3	0,0155
TRS130	18,6	10,2	16,9	15,6	0,9	0,8588
TRS20	15,3	4,9	11,0	12,0	0,7	0,5264
TRS23	44,9	19,4	93,0	58,9	2,1	0,1715
TRS31	23,9	13,7	8,6	8,4	0,4	0,1056
TRS33	54,9	51,1	36,6	38,6	0,7	0,5882
TRX1	184,2	113,8	113,7	98,7	0,6	0,3858
TRX2	291,3	91,2	711,8	676,8	2,4	0,2642
TRX3	82,8	39,0	48,5	47,5	0,6	0,3062
TRZ1	14,6	3,2	10,3	11,2	0,7	0,4891
TSA1	716,6	574,2	1481,5	823,8	2,1	0,1785
TSA2	36,8	11,7	31,4	23,7	0,9	0,6960
TSC10	78,7	20,5	47,3	35,3	0,6	0,1742
TSC11	1622,0	1263,0	377,3	473,5	0,2	0,1145

TSC13	377,9	182,6	633,0	556,1	1,7	0,4168
TSC3	438,8	286,7	439,1	130,2	1,0	0,9983
TSL1	37,7	25,4	96,9	37,4	2,6	0,0397
TSR1	71,9	21,5	138,5	129,8	1,9	0,3505
TSR3	85,2	31,3	189,0	109,6	2,2	0,1184
TSR4	58,4	26,9	32,9	30,5	0,6	0,2554
TTI1	14,2	5,2	29,0	23,1	2,0	0,2559
TTI2	10,9	4,6	58,1	90,1	5,3	0,3363
TUB1	241,2	209,8	167,9	97,9	0,7	0,5502
TUB2	401,2	291,6	119,6	96,8	0,3	0,1164
TUB3	81,4	60,3	176,6	137,9	2,2	0,2528
TUB4	20,1	12,7	2,5	2,9	0,1	0,0350
TUF1	55,3	33,4	47,5	37,2	0,9	0,7660
TUL1	43,1	12,7	18,5	14,0	0,4	0,0404
TUM1	25,5	25,4	14,8	16,1	0,6	0,5038
TUS1	12,1	5,6	46,5	63,6	3,9	0,3218
TVP15	420,8	280,9	551,7	171,8	1,3	0,4568
TVP23	4,1	6,7	4,3	5,0	1,0	0,9687
TVP38	78,1	31,0	40,8	27,3	0,5	0,1215
TWF1	60,5	13,4	52,2	37,3	0,9	0,6888
TYE7	248,4	89,9	341,0	82,0	1,4	0,1786
TYR1	72,7	51,3	55,7	40,9	0,8	0,6229
TYS1	147,1	133,9	107,6	120,1	0,7	0,6764
TYW1	59,7	25,2	45,3	32,6	0,8	0,5126
TYW3	23,0	15,4	12,9	8,8	0,6	0,2953
UAF30	37,8	24,4	50,2	35,3	1,3	0,5843
UBA1	316,4	186,9	374,1	259,7	1,2	0,7309
UBA2	329,0	185,9	151,2	123,1	0,5	0,1618
UBA3	79,0	83,0	192,9	208,8	2,4	0,3496
UBA4	98,1	22,1	43,0	30,3	0,4	0,0260
UBC1	263,7	249,8	208,9	193,9	0,8	0,7403
UBC11	29,6	11,5	19,4	26,2	0,7	0,5015
UBC12	30,3	10,6	27,2	18,6	0,9	0,7809
UBC13	334,0	117,0	229,9	73,0	0,7	0,1819
UBC4	128,3	55,8	211,1	116,9	1,6	0,2481
UBC5	20,3	18,5	34,0	27,6	1,7	0,4416
UBC6	20,5	19,3	27,8	29,8	1,4	0,6979
UBC7	60,6	38,8	29,4	30,1	0,5	0,2502
UBC8	35,1	35,3	39,2	30,0	1,1	0,8647
UBC9	68,9	50,8	25,9	25,9	0,4	0,1826
UBI4	15,0	4,7	10,6	7,9	0,7	0,3734
UBP1	35,3	21,1	35,0	26,7	1,0	0,9836
UBP10	112,8	45,2	165,3	90,3	1,5	0,3387
UBP11	24,3	17,6	20,1	13,5	0,8	0,7199
UBP12	88,8	35,7	73,3	49,8	0,8	0,6293
UBP13	70,2	56,2	124,0	165,9	1,8	0,5616
UBP14	35,7	29,4	33,2	31,4	0,9	0,9115
UBP15	27,3	13,4	43,5	31,6	1,6	0,3824
UBP16	8,3	9,9	5,7	4,4	0,7	0,6424
UBP2	86,4	48,6	98,7	67,9	1,1	0,7794

UBP3	32,8	14,4	13,3	11,8	0,4	0,0815
UBP5	12,6	5,2	24,8	17,1	2,0	0,2192
UBP6	74,4	46,2	98,8	71,3	1,3	0,5863
UBP7	31,7	16,9	24,7	16,6	0,8	0,5778
UBP8	29,5	26,7	30,0	23,7	1,0	0,9774
UBP9	89,2	75,3	39,6	43,8	0,4	0,2979
UBR1	17,9	4,3	31,9	28,6	1,8	0,3703
UBR2	37,2	18,9	212,7	242,9	5,7	0,1999
UBS1	41,9	31,0	19,9	13,9	0,5	0,2430
UBX2	23,4	6,0	40,7	28,8	1,7	0,2839
UBX3	7,6	5,8	9,0	8,4	1,2	0,7914
UBX4	9,5	3,2	26,7	18,3	2,8	0,1129
UBX5	11,7	10,1	20,2	24,3	1,7	0,5400
UBX6	81,6	69,5	31,7	22,5	0,4	0,2215
UBX7	8,0	2,6	18,1	18,9	2,3	0,3304
UFD1	95,3	75,2	222,9	211,4	2,3	0,2988
UFD2	15,0	5,9	22,2	19,2	1,5	0,5031
UFD4	19,5	12,1	41,7	37,9	2,1	0,3057
UFE1	55,9	36,2	21,8	14,9	0,4	0,1328
UFO1	43,8	54,2	13,0	12,1	0,3	0,3113
UGA1	25,2	15,3	47,5	32,6	1,9	0,2625
UGA2	148,8	44,1	275,9	40,6	1,9	0,0054
UGA3	23,6	12,5	22,6	22,9	1,0	0,9431
UGA4	39,0	14,9	88,2	135,1	2,3	0,4965
UGO1	918,9	237,8	241,4	113,1	0,3	0,0021
UGP1	57,3	47,6	71,1	55,7	1,2	0,7187
UGX2	1,4	2,7	0,0	0,0	0,0	0,3559
UIP3	21,3	6,7	195,9	219,1	9,2	0,1623
UIP4	8,6	6,3	8,5	6,3	1,0	0,9866
UIP5	138,1	69,3	55,7	56,6	0,4	0,1149
ULA1	36,8	3,7	12,1	9,7	0,3	0,0030
ULI1	52,7	10,7	78,6	64,9	1,5	0,4597
ULP2	10,8	7,6	11,9	12,3	1,1	0,8804
ULS1	28,9	13,0	44,0	36,0	1,5	0,4627
UME1	70,0	21,3	95,9	75,0	1,4	0,5309
UME6	35,0	34,3	20,3	22,2	0,6	0,4974
UMP1	77,6	74,2	101,3	93,2	1,3	0,7044
UNG1	22,7	5,6	2,4	2,8	0,1	0,0007
UPC2	7,0	10,5	10,1	9,9	1,4	0,6809
UPF3	8,5	9,0	10,3	8,3	1,2	0,7696
UPS1	42,1	24,6	46,6	45,1	1,1	0,8664
UPS2	17,5	10,5	6,2	8,4	0,4	0,1427
UPS3	100,8	95,1	369,8	278,9	3,7	0,1176
URA1	341,9	328,5	411,5	399,0	1,2	0,7967
URA10	49,9	25,6	115,2	99,2	2,3	0,2500
URA2	218,2	166,8	337,7	100,7	1,5	0,2659
URA4	128,2	94,8	96,4	103,1	0,8	0,6658
URA5	2601,7	1221,9	3242,8	1277,6	1,2	0,4956
URA6	93,6	81,6	75,9	60,2	0,8	0,7386
URA7	49,3	42,7	43,2	43,2	0,9	0,8476

URA8	141,7	96,5	44,8	42,6	0,3	0,1157
URB1	18,9	13,3	24,5	19,7	1,3	0,6551
URB2	15,2	7,5	10,3	8,3	0,7	0,4174
URC2	45,2	10,6	16,8	12,5	0,4	0,0133
URE2	142,1	33,7	196,0	153,5	1,4	0,5184
URH1	18,2	3,6	15,2	12,0	0,8	0,6559
URK1	28,6	15,9	14,5	11,4	0,5	0,1983
URN1	3,0	4,6	107,4	204,5	35,5	0,3467
USA1	136,7	138,7	112,0	72,6	0,8	0,7625
USE1	15,5	9,0	17,9	13,9	1,2	0,7806
USO1	273,7	164,6	344,8	276,2	1,3	0,6738
USV1	58,3	16,1	84,4	58,9	1,4	0,4251
UTH1	485,6	310,9	1343,7	835,4	2,8	0,1025
UTP10	107,0	9,2	172,3	134,2	1,6	0,3685
UTP11	50,4	27,3	20,4	18,0	0,4	0,1166
UTP13	10,0	9,4	14,7	15,5	1,5	0,6195
UTP14	119,7	75,7	131,2	110,1	1,1	0,8684
UTP15	75,8	70,0	76,4	72,8	1,0	0,9916
UTP18	50,7	34,3	43,8	30,4	0,9	0,7719
UTP20	615,8	227,3	68,5	61,6	0,1	0,0035
UTP21	63,2	19,3	71,7	50,5	1,1	0,7646
UTP22	43,5	20,0	19,2	19,0	0,4	0,1289
UTP23	59,4	50,9	22,1	19,5	0,4	0,2197
UTP25	79,6	6,5	42,0	33,8	0,5	0,0716
UTP30	16,5	16,4	10,2	8,4	0,6	0,5221
UTP4	16,1	9,6	19,2	14,7	1,2	0,7325
UTP5	113,8	94,2	113,4	96,1	1,0	0,9949
UTP6	42,1	22,1	28,0	23,5	0,7	0,4141
UTP7	71,7	53,6	109,6	101,6	1,5	0,5335
UTP8	65,8	12,1	70,5	59,0	1,1	0,8806
UTP9	369,6	149,2	287,4	118,9	0,8	0,4221
UTR1	96,1	56,8	65,3	46,2	0,7	0,4335
UTR2	138,9	121,5	85,4	94,4	0,6	0,5134
UTR4	25,5	17,7	67,6	84,3	2,6	0,3664
UTR5	0,0	0,0	0,7	1,3	#DIV/0!	0,3559
VAB2	56,2	21,1	52,7	39,4	0,9	0,8788
VAC14	43,2	4,8	14,8	10,0	0,3	0,0022
VAC17	26,1	13,7	7,3	5,9	0,3	0,0456
VAC7	22,7	15,5	31,2	22,2	1,4	0,5504
VAC8	105,4	51,3	135,7	40,8	1,3	0,3912
VAM3	32,0	28,2	35,1	32,2	1,1	0,8909
VAM6	18,1	8,3	23,3	20,1	1,3	0,6488
VAM7	1,5	2,9	4,7	5,8	3,2	0,3536
VAN1	91,4	19,0	102,8	42,3	1,1	0,6384
VAS1	137,1	104,3	104,0	110,0	0,8	0,6773
VBA1	217,0	154,8	321,8	194,8	1,5	0,4320
VBA2	24,7	10,4	35,2	36,1	1,4	0,5954
VBA3	5,9	2,7	4,9	5,9	0,8	0,7689
VBA4	92,6	29,2	55,2	10,9	0,6	0,0530
VBA5	238,9	82,6	265,1	17,5	1,1	0,5566

VCX1	139,9	57,9	125,5	138,4	0,9	0,8542
VEL1	90,6	70,6	48,5	74,6	0,5	0,4434
VHR1	50,8	32,4	41,2	30,6	0,8	0,6836
VHS1	65,7	7,9	170,2	72,4	2,6	0,0284
VHS2	38,9	22,8	31,0	25,2	0,8	0,6587
VHS3	10,9	8,2	37,8	33,4	3,5	0,1681
VHT1	110,9	35,7	181,2	73,3	1,6	0,1353
VID22	15,4	6,6	86,1	163,5	5,6	0,4211
VID24	50,3	38,4	48,5	54,9	1,0	0,9577
VID27	5,0	7,5	0,7	1,3	0,1	0,3027
VID28	50,2	21,5	54,5	45,2	1,1	0,8690
VID30	8,4	8,9	12,6	10,9	1,5	0,5741
VIK1	236,9	91,7	96,2	114,6	0,4	0,1037
VIP1	21,3	11,8	74,9	71,6	3,5	0,1898
VMA1	437,6	295,7	621,6	303,0	1,4	0,4182
VMA10	8,5	3,7	4,7	3,4	0,6	0,1784
VMA11	259,3	135,6	183,6	132,9	0,7	0,4556
VMA13	138,2	60,8	109,0	91,9	0,8	0,6157
VMA16	144,6	89,5	63,1	58,2	0,4	0,1782
VMA2	123,5	86,9	149,0	74,1	1,2	0,6719
VMA22	8,4	10,4	10,0	9,8	1,2	0,8344
VMA5	96,9	39,9	83,6	56,5	0,9	0,7126
VMA6	92,8	83,8	66,6	71,7	0,7	0,6510
VMA7	23,1	10,6	26,1	25,3	1,1	0,8328
VMA8	9,6	5,2	3,7	3,1	0,4	0,0993
VMR1	26,4	21,6	36,6	29,2	1,4	0,5952
VMS1	34,2	15,3	31,9	31,7	0,9	0,8969
VNX1	17,0	11,3	12,2	9,4	0,7	0,5436
VOA1	756,3	398,4	735,9	186,4	1,0	0,9289
VPH1	509,4	112,5	303,9	80,7	0,6	0,0250
VPH2	56,4	23,8	28,1	18,9	0,5	0,1123
VPS1	83,6	56,8	31,3	33,6	0,4	0,1639
VPS13	29,3	24,6	16,5	11,6	0,6	0,3841
VPS15	15,2	10,2	90,0	124,7	5,9	0,2766
VPS16	22,0	9,6	34,5	23,8	1,6	0,3653
VPS17	40,6	9,9	34,6	26,5	0,9	0,6857
VPS20	56,4	27,3	59,2	41,5	1,0	0,9157
VPS21	116,7	62,0	206,7	153,4	1,8	0,3186
VPS24	121,6	53,0	117,7	104,4	1,0	0,9488
VPS25	20,0	11,2	14,9	11,9	0,7	0,5568
VPS27	11,0	4,9	13,2	10,4	1,2	0,7173
VPS28	13,6	14,5	14,2	15,1	1,0	0,9615
VPS3	29,6	17,3	56,5	45,6	1,9	0,3124
VPS30	9,2	9,6	9,4	7,3	1,0	0,9711
VPS33	40,3	12,8	57,0	55,5	1,4	0,5788
VPS34	54,2	16,8	136,3	149,8	2,5	0,3180
VPS35	234,5	187,5	343,9	367,2	1,5	0,6146
VPS36	466,1	272,6	381,6	253,6	0,8	0,6658
VPS38	26,0	16,7	26,1	18,0	1,0	0,9925
VPS4	71,2	39,4	60,0	40,9	0,8	0,7080

VPS41	62,6	37,4	81,1	54,7	1,3	0,5959
VPS45	210,8	190,3	107,8	100,3	0,5	0,3757
VPS5	58,7	21,5	49,7	45,9	0,8	0,7364
VPS52	20,8	8,9	16,8	15,2	0,8	0,6657
VPS53	53,1	20,6	30,4	23,1	0,6	0,1918
VPS54	37,7	6,0	39,4	26,4	1,0	0,9062
VPS55	45,3	31,7	22,0	17,4	0,5	0,2440
VPS60	827,1	863,5	643,5	536,3	0,8	0,7303
VPS61	0,0	0,0	0,7	1,3	#DIV/0!	0,3559
VPS62	29,7	19,1	120,1	73,3	4,0	0,0543
VPS63	21,8	24,0	29,5	34,9	1,4	0,7277
VPS64	33,0	25,4	15,6	12,9	0,5	0,2691
VPS66	400,9	96,9	340,2	68,1	0,8	0,3448
VPS68	89,4	69,7	88,5	77,6	1,0	0,9862
VPS69	35,2	15,5	27,6	21,2	0,8	0,5833
VPS70	93,5	38,6	143,7	45,4	1,5	0,1425
VPS71	49,7	31,8	44,2	38,0	0,9	0,8319
VPS72	52,8	25,6	66,5	50,3	1,3	0,6439
VPS73	27,7	6,5	27,5	28,3	1,0	0,9893
VPS74	102,9	59,6	57,9	56,2	0,6	0,3136
VPS75	278,0	180,1	352,6	152,5	1,3	0,5504
VPS8	7,8	2,3	19,3	14,8	2,5	0,1780
VPS9	64,6	26,1	54,4	51,7	0,8	0,7365
VRG4	113,6	86,9	39,4	31,7	0,3	0,1597
VRP1	26,2	19,6	112,9	155,0	4,3	0,3093
VTA1	36,7	25,5	21,4	19,2	0,6	0,3759
VTC1	876,9	276,8	1872,8	673,2	2,1	0,0339
VTC2	210,3	49,4	428,1	211,5	2,0	0,0918
VTC3	82,1	18,9	244,3	162,9	3,0	0,0954
VTC4	53,7	34,7	217,7	80,5	4,1	0,0096
VTH1	50,4	24,5	20,7	23,7	0,4	0,1322
VTI1	5,2	3,5	18,0	14,2	3,5	0,1313
VTS1	20,0	12,3	5,6	6,5	0,3	0,0828
WAR1	18,9	11,0	24,8	16,5	1,3	0,5739
WBP1	368,7	140,2	538,6	196,1	1,5	0,2084
WHI2	123,0	71,1	76,8	56,1	0,6	0,3466
WHI3	52,2	36,4	40,5	32,9	0,8	0,6502
WHI4	393,8	262,1	416,9	300,4	1,1	0,9117
WRS1	90,6	36,2	108,4	91,0	1,2	0,7286
WSC2	220,4	52,2	51,6	35,2	0,2	0,0017
WSC3	153,9	96,3	37,3	26,3	0,2	0,0581
WSC4	18,8	8,7	16,4	12,3	0,9	0,7563
WSS1	1,0	2,0	1,8	3,6	1,8	0,7126
WTM1	201,3	176,2	276,1	250,9	1,4	0,6429
WTM2	2,3	3,2	7,8	5,3	3,4	0,1259
WWM1	83,4	85,3	124,5	137,0	1,5	0,6287
XBP1	7,0	6,8	6,0	5,2	0,9	0,8300
XDJ1	13,0	7,6	13,8	13,8	1,1	0,9170
XKS1	79,6	14,9	152,0	75,1	1,9	0,1074
XPT1	35,2	20,7	31,8	26,5	0,9	0,8454

XRS2	1,0	2,0	8,5	10,1	8,6	0,1939
XYL2	68,0	49,9	84,2	83,8	1,2	0,7513
YAE1	43,8	44,6	46,3	44,0	1,1	0,9374
YAF9	5,3	4,5	0,7	1,3	0,1	0,0951
YAH1	7,7	3,2	5,4	4,2	0,7	0,4121
YAK1	7,2	3,1	9,1	6,1	1,3	0,6011
YAL018C	0,0	0,0	0,0	0,0	#DIV/0!	#DIV/0!
YAL026C-A	29,7	19,1	22,7	17,0	0,8	0,6014
YAL037C-A	52,1	38,8	24,5	24,0	0,5	0,2721
YAL063C-A	10,1	4,5	10,9	9,1	1,1	0,8806
YAL064C-A	1,8	2,1	5,5	8,1	3,1	0,4077
YAL064W	7,9	6,0	2,4	3,4	0,3	0,1630
YAL066W	4,2	5,3	1,6	3,3	0,4	0,4422
YAP1	52,4	35,7	48,2	40,1	0,9	0,8806
YAP1801	91,0	44,4	87,4	80,7	1,0	0,9397
YAP1802	23,7	6,4	23,1	19,5	1,0	0,9616
YAP3	48,4	9,6	93,3	85,4	1,9	0,3363
YAP5	2,4	3,0	13,2	11,7	5,5	0,1219
YAP6	34,1	25,3	103,6	73,8	3,0	0,1251
YAP7	102,2	64,2	195,2	267,3	1,9	0,5240
YAR009C	6440,6	4426,3	9654,7	3758,7	1,5	0,3107
YAR010C	2940,5	424,0	3803,3	500,1	1,3	0,0390
YAR023C	4,1	3,6	16,4	11,1	4,0	0,0792
YAR028W	36,9	11,5	41,6	30,2	1,1	0,7787
YAR029W	0,0	0,0	1,2	2,4	#DIV/0!	0,3559
YAR047C	1,0	2,0	1,3	2,6	1,3	0,8485
YAR053W	129,1	66,3	118,6	153,7	0,9	0,9047
YAR060C	0,9	1,8	0,0	0,0	0,0	0,3559
YAR061W	4,2	6,3	3,1	3,7	0,7	0,7646
YAR062W	1,1	2,1	0,0	0,0	0,0	0,3559
YAR068W	20,9	20,1	5,5	6,5	0,3	0,1962
YAR069C	51,9	60,1	880,0	1149,4	17,0	0,2002
YAR070C	22,5	16,8	266,7	397,0	11,9	0,2649
YAR075W	1,7	3,4	0,0	0,0	0,0	0,3559
YAR1	139,4	66,6	122,6	96,5	0,9	0,7829
YAT1	6,7	5,4	5,3	4,0	0,8	0,6922
YAT2	58,8	21,3	164,0	78,2	2,8	0,0409
YBL005W-A	285,5	140,8	532,3	160,9	1,9	0,0603
YBL005W-B	3137,6	2301,3	4256,7	2590,9	1,4	0,5423
YBL010C	6,4	4,1	5,6	5,0	0,9	0,8106
YBL028C	353,9	201,4	346,6	278,9	1,0	0,9674
YBL029C-A	64,4	12,5	49,9	39,9	0,8	0,5128
YBL029W	221,7	64,8	454,2	235,9	2,0	0,1060
YBL036C	45,7	30,4	46,6	44,7	1,0	0,9739
YBL039C-A	4,0	4,7	2,5	2,9	0,6	0,5964
YBL039W-B	59,8	30,2	158,9	144,9	2,7	0,2290
YBL044W	0,0	0,0	1,3	2,5	#DIV/0!	0,3559
YBL055C	163,6	121,4	31,7	37,6	0,2	0,0834
YBL059W	26,8	9,4	211,9	291,2	7,9	0,2510
YBL068W-A	35,8	14,7	94,0	63,7	2,6	0,1255

YBL071C	1641,1	1314,5	1879,1	1381,2	1,1	0,8112
YBL071C-B	1,0	2,0	0,0	0,0	0,0	0,3559
YBL073W	0,9	1,8	1,3	2,5	1,4	0,8118
YBL081W	83,0	27,3	31,8	22,5	0,4	0,0274
YBL086C	6,7	7,8	11,3	8,0	1,7	0,4381
YBL100W-A	538,1	86,4	979,4	208,7	1,8	0,0079
YBL100W-B	1020,9	586,3	1364,6	425,8	1,3	0,3795
YBL104C	17,1	8,6	16,2	17,1	0,9	0,9270
YBL107C	14,9	8,5	12,1	10,3	0,8	0,6930
YBL108W	1,2	2,4	1,2	2,4	1,0	0,9963
YBL109W	0,0	0,0	0,7	1,3	#DIV/0!	0,3559
YBL111C	39,7	15,3	19,3	17,3	0,5	0,1284
YBL112C	20,6	19,8	8,7	6,5	0,4	0,3004
YBL113C	18,8	14,3	6,5	8,0	0,3	0,1806
YBP1	174,9	67,8	58,5	42,6	0,3	0,0270
YBP2	17,0	17,1	20,8	15,0	1,2	0,7452
YBR012C	201,6	86,5	507,6	447,9	2,5	0,2282
YBR012W-A	2081,1	616,7	1681,0	471,3	0,8	0,3424
YBR012W-B	57,1	32,1	141,2	99,3	2,5	0,1580
YBR013C	9,2	9,4	5,5	6,9	0,6	0,5554
YBR016W	467,6	163,2	228,8	165,3	0,5	0,0855
YBR028C	31,7	22,1	26,0	17,4	0,8	0,6975
YBR032W	64,7	38,0	24,7	27,9	0,4	0,1408
YBR053C	79,2	27,3	141,1	108,5	1,8	0,3108
YBR056W	49,1	36,3	71,5	51,4	1,5	0,5038
YBR062C	171,1	44,4	324,7	89,4	1,9	0,0218
YBR063C	24,1	4,9	75,8	60,4	3,1	0,1391
YBR071W	49,8	31,3	206,2	323,5	4,1	0,3728
YBR074W	51,0	20,6	69,5	48,1	1,4	0,5073
YBR076C-A	25,4	26,0	10,5	9,2	0,4	0,3233
YBR085C-A	220,6	152,4	1098,2	896,3	5,0	0,1018
YBR096W	52,1	28,6	117,0	180,2	2,2	0,5033
YBR109W-A	5,9	11,8	12,0	10,4	2,0	0,4660
YBR116C	9,1	15,2	1,3	2,6	0,1	0,3528
YBR124W	57,0	13,3	8,6	8,1	0,2	0,0008
YBR134W	5,3	8,1	7,5	6,1	1,4	0,6717
YBR137W	55,7	37,9	37,4	38,8	0,7	0,5263
YBR138C	5,2	6,2	7,2	11,3	1,4	0,7716
YBR139W	65,2	47,6	87,8	67,4	1,3	0,6027
YBR141C	0,6	1,2	0,0	0,0	0,0	0,3559
YBR144C	82,2	51,8	36,0	29,9	0,4	0,1733
YBR182C-A	10,6	12,5	12,7	9,8	1,2	0,8081
YBR184W	2,9	3,4	0,0	0,0	0,0	0,1445
YBR190W	1,5	3,0	0,0	0,0	0,0	0,3559
YBR191W-A	168,2	111,0	131,4	154,4	0,8	0,7123
YBR196C-B	200,4	30,6	63,3	54,6	0,3	0,0046
YBR197C	33,4	16,9	40,1	26,8	1,2	0,6873
YBR200W-A	3,9	5,8	10,7	9,8	2,7	0,2801
YBR201C-A	36,1	18,3	12,7	12,0	0,4	0,0772
YBR204C	33,6	17,2	37,9	39,2	1,1	0,8473

YBR206W	1,5	3,0	1,3	2,5	0,9	0,9141
YBR209W	0,0	0,0	0,0	0,0	#DIV/0!	#DIV/0!
YBR219C	8,6	6,0	1,0	2,0	0,1	0,0510
YBR220C	4,2	1,9	4,4	3,3	1,1	0,9047
YBR221W-A	0,0	0,0	5,1	3,7	#DIV/0!	0,0328
YBR224W	1,4	2,7	0,0	0,0	0,0	0,3559
YBR225W	1,6	1,9	10,5	8,4	6,7	0,0828
YBR230W-A	100,6	8,5	158,1	110,3	1,6	0,3392
YBR235W	38,6	5,2	11,1	9,9	0,3	0,0026
YBR238C	8,7	11,9	20,0	14,8	2,3	0,2763
YBR241C	39,3	11,7	25,9	22,1	0,7	0,3261
YBR242W	28,3	15,6	16,6	11,3	0,6	0,2712
YBR255C-A	16,1	6,1	10,2	8,7	0,6	0,3098
YBR259W	5,2	3,6	10,3	8,3	2,0	0,3093
YBR271W	99,2	80,1	25,6	28,3	0,3	0,1341
YBR284W	2,6	5,3	3,6	7,3	1,4	0,8340
YBR285W	107,2	95,4	49,1	63,6	0,5	0,3500
YBR287W	33,6	11,4	37,5	29,4	1,1	0,8088
YBR292C	15,0	9,8	50,1	31,4	3,3	0,0772
YBT1	15,2	6,9	21,5	16,7	1,4	0,5165
YCF1	142,4	36,0	179,6	41,0	1,3	0,2221
YCG1	49,6	26,2	68,5	46,8	1,4	0,5083
YCH1	58,6	22,8	99,8	73,7	1,7	0,3263
YCK1	59,3	49,1	37,5	29,4	0,6	0,4760
YCK2	229,4	74,1	240,7	226,6	1,0	0,9279
YCK3	57,1	83,7	18,1	17,9	0,3	0,3979
YCL001W-B	34,8	15,3	95,2	124,3	2,7	0,3718
YCL002C	162,2	47,0	71,3	62,8	0,4	0,0595
YCL019W	12,6	6,8	190,2	108,1	15,1	0,0168
YCL021W-A	4,7	5,5	0,0	0,0	0,0	0,1346
YCL023C	0,0	0,0	0,0	0,0	#DIV/0!	#DIV/0!
YCL041C	15,2	8,5	22,7	16,7	1,5	0,4518
YCL042W	22,8	12,6	18,0	13,3	0,8	0,6207
YCL047C	19,0	10,2	36,1	34,6	1,9	0,3790
YCL048W-A	14,0	8,7	28,4	35,1	2,0	0,4532
YCL049C	87,2	74,2	137,9	97,8	1,6	0,4405
YCL057C-A	203,3	184,7	271,7	264,0	1,3	0,6857
YCL065W	0,0	0,0	0,0	0,0	#DIV/0!	#DIV/0!
YCL073C	15,2	11,3	17,5	14,0	1,2	0,8046
YCP4	355,6	247,5	211,9	153,0	0,6	0,3615
YCR001W	58,6	36,1	98,9	72,8	1,7	0,3591
YCR006C	1,0	2,0	0,0	0,0	0,0	0,3559
YCR007C	238,7	164,3	878,2	544,7	3,7	0,0656
YCR015C	10,1	5,6	21,4	20,6	2,1	0,3325
YCR018C-A	12,4	10,7	8,6	9,9	0,7	0,6229
YCR022C	1,1	2,1	0,0	0,0	0,0	0,3559
YCR023C	141,1	24,0	78,0	52,3	0,6	0,0707
YCR024C-B	3141,3	705,6	6707,7	478,5	2,1	0,0002
YCR043C	62,6	43,8	22,0	21,9	0,4	0,1490
YCR051W	105,4	70,9	124,2	105,5	1,2	0,7776

YCR061W	12,7	5,3	18,0	12,8	1,4	0,4744
YCR064C	1,9	2,2	0,0	0,0	0,0	0,1350
YCR076C	58,4	15,2	47,8	32,3	0,8	0,5736
YCR090C	26,4	17,1	14,2	10,5	0,5	0,2716
YCR095W-A	10,5	7,3	62,8	123,8	6,0	0,4317
YCR097W-A	0,0	0,0	0,0	0,0	#DIV/0!	#DIV/0!
YCR099C	1,8	3,7	4,3	6,0	2,4	0,4992
YCR100C	0,0	0,0	0,0	0,0	#DIV/0!	#DIV/0!
YCR101C	0,0	0,0	0,0	0,0	#DIV/0!	#DIV/0!
YCR102C	66,5	35,0	32,3	25,0	0,5	0,1625
YCR102W-A	0,6	1,2	0,7	1,3	1,1	0,9577
YCS4	50,7	17,3	104,1	134,2	2,1	0,4603
YCT1	9,5	4,0	36,2	34,6	3,8	0,1758
YDC1	41,9	17,7	74,2	44,5	1,8	0,2256
YDJ1	356,0	194,6	506,9	268,0	1,4	0,3974
YDL012C	7,1	8,5	1,3	2,5	0,2	0,2375
YDL016C	73,3	30,2	49,4	39,7	0,7	0,3751
YDL022C-A	0,0	0,0	0,0	0,0	#DIV/0!	#DIV/0!
YDL027C	135,7	88,9	168,7	121,8	1,2	0,6771
YDL057W	7,5	4,2	4,7	3,4	0,6	0,3358
YDL063C	47,8	27,3	53,7	42,0	1,1	0,8229
YDL073W	62,9	28,7	40,8	28,8	0,6	0,3192
YDL085C-A	41,5	46,1	44,2	48,8	1,1	0,9377
YDL086W	60,8	8,8	26,8	18,9	0,4	0,0174
YDL109C	10,1	9,1	1,9	3,8	0,2	0,1473
YDL114W	1,4	2,7	0,7	1,3	0,5	0,6563
YDL119C	21,2	5,1	31,2	23,0	1,5	0,4286
YDL121C	182,6	172,9	279,1	339,4	1,5	0,6305
YDL124W	171,0	51,5	270,7	50,8	1,6	0,0329
YDL129W	7,6	6,5	107,9	149,2	14,3	0,2276
YDL133W	75,1	43,5	56,5	50,5	0,8	0,5955
YDL144C	41,2	22,9	33,8	33,3	0,8	0,7278
YDL156W	11,7	10,4	2,0	4,0	0,2	0,1308
YDL157C	97,0	70,3	160,7	113,2	1,7	0,3764
YDL159W-A	0,0	0,0	1,8	3,6	#DIV/0!	0,3559
YDL160C-A	1168,4	748,6	1055,2	1057,2	0,9	0,8670
YDL176W	5,7	11,3	54,0	66,2	9,5	0,2005
YDL177C	15,5	12,5	15,8	18,6	1,0	0,9795
YDL180W	19,4	10,7	15,6	15,7	0,8	0,7028
YDL183C	73,6	31,7	41,8	28,5	0,6	0,1866
YDL186W	29,1	10,5	47,8	32,1	1,6	0,3101
YDL199C	21,3	12,2	232,2	261,2	10,9	0,1579
YDL206W	21,6	12,8	14,4	15,1	0,7	0,4930
YDL211C	8,7	7,1	1,3	2,5	0,1	0,0958
YDL218W	3,1	2,6	5,3	4,2	1,7	0,4147
YDL233W	23,3	18,7	23,4	17,3	1,0	0,9928
YDL240C-A	25,9	16,2	28,2	23,5	1,1	0,8780
YDL241W	30,8	15,1	21,2	14,6	0,7	0,3957
YDL242W	7,8	3,0	39,3	34,4	5,0	0,1178
YDR003W-A	95,7	18,9	93,8	65,4	1,0	0,9565

YDR018C	3,9	4,8	10,9	11,1	2,8	0,2958
YDR026C	83,5	38,6	69,2	53,5	0,8	0,6809
YDR029W	9,5	11,0	3,0	5,9	0,3	0,3341
YDR034C-A	28,4	24,6	125,3	137,5	4,4	0,2150
YDR034C-C	91,7	84,9	70,5	63,2	0,8	0,7013
YDR034C-D	3,4	4,7	2,0	4,0	0,6	0,6683
YDR034W-B	5,3	4,2	91,8	168,8	17,2	0,3453
YDR042C	19,0	15,7	5,8	7,8	0,3	0,1817
YDR056C	27,4	15,0	232,5	406,6	8,5	0,3522
YDR061W	72,5	15,6	123,4	77,6	1,7	0,2462
YDR089W	2278,7	1373,7	2531,4	1688,3	1,1	0,8242
YDR090C	17,2	9,2	11,3	11,7	0,7	0,4574
YDR098C-B	26,1	17,8	134,5	77,0	5,2	0,0336
YDR102C	69,2	49,7	381,8	484,0	5,5	0,2462
YDR109C	6,2	4,3	1,9	3,8	0,3	0,1882
YDR114C	3,4	6,7	1,6	3,3	0,5	0,6642
YDR118W-A	30,1	20,3	285,3	313,7	9,5	0,1555
YDR119W-A	40,4	36,1	37,7	32,6	0,9	0,9150
YDR124W	167,7	97,3	291,4	210,6	1,7	0,3270
YDR131C	1,8	2,3	12,3	13,0	6,9	0,1602
YDR132C	37,2	13,8	33,4	42,7	0,9	0,8713
YDR154C	924,6	877,9	1608,3	1719,4	1,7	0,5053
YDR157W	4,2	6,3	6,1	8,4	1,4	0,7375
YDR161W	20,4	20,0	19,8	26,4	1,0	0,9763
YDR169C-A	1,0	2,0	7,2	8,6	7,3	0,2079
YDR179W-A	49,1	11,5	23,6	23,4	0,5	0,0982
YDR182W-A	11,3	9,1	12,7	12,0	1,1	0,8510
YDR183C-A	69,5	24,9	32,1	21,8	0,5	0,0644
YDR186C	56,9	19,6	47,5	33,8	0,8	0,6481
YDR193W	0,0	0,0	0,0	0,0	#DIV/0!	#DIV/0!
YDR209C	2869,9	2260,1	1200,5	991,2	0,4	0,2248
YDR210W-A	145,4	120,1	118,8	59,6	0,8	0,7055
YDR210W-B	2,6	3,1	4,0	3,0	1,5	0,5345
YDR215C	40,8	43,8	29,5	36,2	0,7	0,7063
YDR222W	292,2	178,4	337,2	384,5	1,2	0,8388
YDR239C	21,9	15,5	11,4	11,1	0,5	0,3130
YDR246W-A	4,1	8,2	23,7	47,5	5,8	0,4464
YDR248C	41,0	26,2	50,5	35,2	1,2	0,6805
YDR249C	26,9	12,6	14,9	12,4	0,6	0,2262
YDR250C	0,0	0,0	3,1	3,7	#DIV/0!	0,1509
YDR261W-A	591,5	417,7	955,1	780,8	1,6	0,4428
YDR262W	81,5	93,1	54,1	47,2	0,7	0,6187
YDR266C	23,3	4,4	14,7	15,0	0,6	0,3164
YDR274C	105,9	88,6	134,6	122,1	1,3	0,7162
YDR278C	1,7	3,4	0,0	0,0	0,0	0,3559
YDR282C	0,0	0,0	3,7	3,1	#DIV/0!	0,0524
YDR286C	19,1	13,8	8,4	5,8	0,4	0,2047
YDR306C	38,7	7,7	71,8	57,8	1,9	0,2993
YDR307W	146,2	42,8	50,4	37,9	0,3	0,0153
YDR319C	21,9	13,4	23,8	19,8	1,1	0,8801

YDR327W	1,5	3,0	1,6	3,3	1,1	0,9577
YDR333C	32,2	19,7	35,3	27,8	1,1	0,8588
YDR336W	17,2	4,4	14,9	13,8	0,9	0,7652
YDR338C	9,3	5,9	15,6	14,8	1,7	0,4560
YDR340W	6,6	3,1	4,1	2,9	0,6	0,2814
YDR341C	145,1	90,2	155,3	155,1	1,1	0,9135
YDR344C	9,0	11,6	3,2	3,9	0,3	0,3752
YDR348C	71,0	32,3	45,1	43,3	0,6	0,3742
YDR352W	19,7	13,0	10,5	11,0	0,5	0,3237
YDR370C	8,2	6,1	1,9	3,8	0,2	0,1289
YDR374C	0,6	1,2	0,0	0,0	0,0	0,3559
YDR374W-A	24,7	23,9	15,3	17,7	0,6	0,5512
YDR379C-A	17,5	13,8	153,5	238,6	8,8	0,2984
YDR387C	9,8	3,7	74,1	98,9	7,5	0,2418
YDR391C	91,6	53,2	152,9	125,2	1,7	0,4023
YDR415C	34,1	28,3	38,3	27,4	1,1	0,8379
YDR433W	0,0	0,0	7,8	5,8	#DIV/0!	0,0373
YDR444W	15,5	7,0	15,0	11,4	1,0	0,9404
YDR461C-A	13,1	13,1	3,0	5,9	0,2	0,2087
YDR476C	57,6	41,0	31,9	32,4	0,6	0,3633
YDR491C	91,9	51,4	18,5	17,5	0,2	0,0354
YDR506C	42,4	30,1	36,6	30,8	0,9	0,7945
YDR514C	7,9	2,8	6,0	4,3	0,8	0,4724
YDR524W-C	70086,7	65511,8	74964,5	58970,1	1,1	0,9155
YDR535C	6,8	4,6	7,9	5,3	1,2	0,7652
YDR541C	153,1	30,3	121,3	93,8	0,8	0,5426
YDR543C	0,0	0,0	0,7	1,3	#DIV/0!	0,3559
YEA4	9,7	7,4	14,4	10,7	1,5	0,4974
YEA6	82,9	42,4	89,2	63,0	1,1	0,8752
YEF1	40,5	29,8	14,8	11,1	0,4	0,1572
YEF3	824,6	625,4	766,3	658,8	0,9	0,9020
YEH1	21,5	11,7	7,9	9,7	0,4	0,1242
YEH2	30,7	12,4	10,3	7,0	0,3	0,0287
YEL007W	443,5	79,1	449,1	241,5	1,0	0,9658
YEL008W	23,0	6,5	21,2	15,3	0,9	0,8367
YEL009C-A	0,6	1,2	0,7	1,3	1,1	0,9379
YEL020C	140,8	113,9	28,2	22,1	0,2	0,1003
YEL023C	686,5	76,3	182,0	160,2	0,3	0,0013
YEL025C	14,9	26,8	139,2	256,9	9,4	0,3728
YEL028W	77,3	32,8	301,5	73,2	3,9	0,0014
YEL043W	35,6	21,7	40,3	32,8	1,1	0,8209
YEL045C	10,6	7,9	43,7	33,0	4,1	0,0982
YEL047C	49,7	24,5	35,3	32,6	0,7	0,5042
YEL057C	13,6	8,4	8,7	7,6	0,6	0,4224
YEL067C	4,0	3,4	0,0	0,0	0,0	0,0601
YEL068C	13,4	4,7	8,7	7,3	0,6	0,3162
YEL073C	13,7	3,0	13,2	8,9	1,0	0,9032
YEL074W	12,3	6,3	6,2	8,1	0,5	0,2801
YEL077C	11,8	7,1	12,5	14,2	1,1	0,9319
YEL1	65,6	14,6	35,5	24,8	0,5	0,0820

YEN1	5,2	5,5	11,5	8,8	2,2	0,2704
YER034W	9,9	8,5	28,7	19,7	2,9	0,1307
YER039C-A	17,0	9,5	10,0	9,8	0,6	0,3434
YER053C-A	32,8	15,8	655,0	150,0	20,0	0,0002
YER064C	22,6	15,0	9,3	7,1	0,4	0,1605
YER071C	2244,7	1214,9	290,0	271,1	0,1	0,0201
YER076C	13,5	10,2	17,2	21,8	1,3	0,7685
YER077C	11,8	8,9	13,5	9,1	1,1	0,7956
YER079W	221,8	114,8	830,5	411,0	3,7	0,0291
YER088C-A	16,9	11,3	21,9	25,2	1,3	0,7272
YER107W-A	0,9	1,8	0,7	1,3	0,7	0,8468
YER119C-A	58,0	41,8	136,2	123,9	2,3	0,2771
YER121W	34,2	14,1	11,3	7,8	0,3	0,0292
YER128W	71,0	70,9	41,5	40,8	0,6	0,4982
YER130C	19,6	8,9	40,2	32,1	2,1	0,2631
YER135C	1,6	1,9	6,6	4,9	4,2	0,1053
YER137C	46,2	17,1	23,8	18,3	0,5	0,1243
YER138C	65,6	44,9	61,0	41,6	0,9	0,8851
YER138W-A	274,0	91,5	229,8	165,2	0,8	0,6563
YER140W	6,5	9,5	4,3	5,1	0,7	0,6963
YER147C-A	2,5	3,0	0,0	0,0	0,0	0,1420
YER152C	17,8	6,9	7,8	5,8	0,4	0,0681
YER156C	29,3	23,8	22,3	17,8	0,8	0,6554
YER158C	3,3	2,9	96,1	166,2	29,3	0,3071
YER158W-A	5,8	7,0	17,3	17,5	3,0	0,2698
YER160C	1325,1	1212,8	1933,6	930,4	1,5	0,4563
YER163C	17,9	8,1	29,6	20,4	1,7	0,3264
YER175W-A	74,3	52,6	100,9	89,2	1,4	0,6266
YER181C	0,8	1,5	2,6	3,6	3,4	0,3917
YER184C	50,7	31,0	22,7	32,7	0,4	0,2605
YER186C	41,3	68,4	11,6	13,4	0,3	0,4265
YER187W	1,7	3,4	0,7	1,3	0,4	0,5891
YER188C-A	1,0	2,0	0,0	0,0	0,0	0,3559
YER188W	3,7	7,3	0,0	0,0	0,0	0,3559
YER189W	15,2	9,0	14,0	13,6	0,9	0,8866
YET1	85,3	57,5	95,3	90,4	1,1	0,8579
YET2	9,4	12,2	4,3	5,6	0,5	0,4765
YET3	77,3	46,9	93,8	83,1	1,2	0,7416
YFH1	29,3	17,2	24,4	18,8	0,8	0,7139
YFH7	11,2	7,8	11,6	11,1	1,0	0,9622
YFL012W	21,5	8,9	9,6	13,7	0,4	0,1941
YFL021C-A	0,0	0,0	0,0	0,0	#DIV/0!	#DIV/0!
YFL032W	2,1	4,1	3,2	3,8	1,6	0,6942
YFL034W	70,0	32,9	41,1	34,6	0,6	0,2716
YFL040W	13,5	13,1	9,1	15,2	0,7	0,6761
YFL042C	30,6	20,8	46,3	51,2	1,5	0,5902
YFL051C	5,5	4,7	24,1	26,6	4,4	0,2179
YFL052W	1,6	1,9	1,6	3,3	1,0	0,9716
YFL054C	8,4	9,8	14,7	13,6	1,7	0,4783
YFL063W	0,0	0,0	0,0	0,0	#DIV/0!	#DIV/0!

YFL065C	2,4	2,9	0,7	1,3	0,3	0,3257
YFL066C	5,0	7,2	0,0	0,0	0,0	0,2185
YFR006W	33,2	27,8	37,4	39,6	1,1	0,8682
YFR009W-A	29,3	28,3	19,6	21,5	0,7	0,6045
YFR012W	28,1	13,1	51,6	56,4	1,8	0,4464
YFR012W-A	0,0	0,0	0,0	0,0	#DIV/0!	#DIV/0!
YFR016C	283,2	205,7	342,4	236,3	1,2	0,7186
YFR017C	12,9	3,5	36,5	24,8	2,8	0,1081
YFR018C	22,8	13,1	12,9	13,4	0,6	0,3309
YFR020W	33,1	21,5	135,4	103,5	4,1	0,1011
YFR032C-B	63,2	90,1	10,7	15,0	0,2	0,2941
YFR039C	29,3	14,0	24,2	20,0	0,8	0,6897
YFR045W	391,9	164,0	286,1	169,1	0,7	0,4034
YFR054C	0,6	1,2	0,0	0,0	0,0	0,3559
YFR056C	9,0	11,1	7,5	5,0	0,8	0,8171
YGK3	9,8	10,0	5,1	7,9	0,5	0,4871
YGL006W-A	5,7	5,7	2,0	4,0	0,3	0,3212
YGL010W	841,3	877,4	358,9	110,1	0,4	0,3171
YGL015C	1,2	1,7	4,3	6,0	3,7	0,3471
YGL034C	1,7	3,4	3,0	5,9	1,7	0,7235
YGL036W	26,5	10,1	109,1	93,6	4,1	0,1295
YGL039W	5,4	8,6	6,8	5,3	1,3	0,7925
YGL074C	1,5	2,9	4,2	5,6	2,9	0,4171
YGL081W	8,3	2,6	11,2	8,2	1,4	0,5232
YGL082W	177,5	24,3	150,3	111,8	0,8	0,6512
YGL088W	0,0	0,0	0,0	0,0	#DIV/0!	#DIV/0!
YGL101W	143,1	45,0	177,7	18,8	1,2	0,2060
YGL108C	1,9	1,7	1,0	2,0	0,5	0,5174
YGL114W	79,7	44,6	133,4	83,9	1,7	0,3014
YGL117W	51,0	26,3	59,7	42,8	1,2	0,7399
YGL118C	0,3	0,6	0,0	0,0	0,0	0,3559
YGL138C	18,4	9,6	2,0	4,0	0,1	0,0195
YGL140C	87,2	44,4	70,0	46,8	0,8	0,6126
YGL149W	170,3	137,3	430,2	258,1	2,5	0,1258
YGL159W	9,3	7,4	24,5	16,7	2,6	0,1492
YGL176C	20,2	13,3	30,9	27,8	1,5	0,5107
YGL177W	3,0	3,6	10,2	8,7	3,4	0,1778
YGL182C	0,0	0,0	2,8	3,4	#DIV/0!	0,1592
YGL185C	15,9	9,4	16,3	10,9	1,0	0,9578
YGL188C	514,5	246,7	545,5	328,4	1,1	0,8850
YGL188C-A	26,8	15,8	11,9	12,1	0,4	0,1856
YGL193C	0,0	0,0	2,0	4,0	#DIV/0!	0,3559
YGL194C-A	12,7	10,5	10,4	12,1	0,8	0,7893
YGL204C	42,4	46,0	38,7	37,7	0,9	0,9052
YGL230C	2,1	2,4	0,0	0,0	0,0	0,1344
YGL242C	51,6	10,2	97,3	100,9	1,9	0,4027
YGL262W	0,0	0,0	0,0	0,0	#DIV/0!	#DIV/0!
YGP1	42,2	24,2	591,9	515,4	14,0	0,0771
YGR001C	139,9	79,3	96,0	87,6	0,7	0,4854
YGR012W	36,0	13,7	20,5	14,3	0,6	0,1695

YGR015C	21,1	19,7	43,9	24,0	2,1	0,1923
YGR016W	8,9	6,1	5,6	4,1	0,6	0,3973
YGR017W	108,2	76,8	99,4	95,7	0,9	0,8911
YGR021W	28,8	28,4	52,5	57,2	1,8	0,4848
YGR026W	135,9	85,6	105,0	92,3	0,8	0,6412
YGR027W-A	1064,4	235,1	1165,9	244,6	1,1	0,5712
YGR031W	3,8	5,3	6,3	4,4	1,6	0,5027
YGR035C	44,4	44,2	98,8	173,4	2,2	0,5657
YGR035W-A	759,0	480,5	300,2	350,7	0,4	0,1739
YGR038C-A	307,2	106,2	248,5	185,3	0,8	0,6025
YGR042W	13,5	8,1	9,4	6,5	0,7	0,4581
YGR045C	0,0	0,0	2,8	3,4	#DIV/0!	0,1592
YGR050C	0,0	0,0	0,0	0,0	#DIV/0!	#DIV/0!
YGR051C	2476,0	1806,4	3159,9	1209,7	1,3	0,5524
YGR053C	4,5	6,3	5,5	5,3	1,2	0,8147
YGR054W	25,0	15,3	25,8	24,1	1,0	0,9580
YGR066C	65,5	22,8	46,2	40,8	0,7	0,4408
YGR067C	282,5	401,4	310,0	309,4	1,1	0,9170
YGR069W	39,2	38,0	108,1	114,1	2,8	0,2955
YGR071C	6,0	7,2	7,0	10,8	1,2	0,8832
YGR079W	22,2	24,1	13,9	9,4	0,6	0,5427
YGR093W	69,6	34,1	101,4	61,4	1,5	0,4002
YGR102C	1,8	2,3	1,9	3,8	1,1	0,9688
YGR107W	7,7	6,8	14,5	16,5	1,9	0,4801
YGR109W-A	16,2	3,9	42,5	34,5	2,6	0,1815
YGR109W-B	405,1	136,8	588,8	73,0	1,5	0,0556
YGR111W	78,3	37,0	64,9	55,2	0,8	0,7025
YGR114C	247,2	252,4	103,9	69,4	0,4	0,3156
YGR117C	6,7	5,0	17,6	13,5	2,6	0,1794
YGR121W-A	43,0	34,4	26,0	30,6	0,6	0,4865
YGR122C-A	2,1	2,4	5,5	4,9	2,7	0,2444
YGR122W	228,3	83,8	1126,4	477,7	4,9	0,0100
YGR125W	147,9	56,2	156,4	104,8	1,1	0,8906
YGR126W	2,2	3,3	12,0	8,8	5,4	0,0831
YGR127W	142,0	51,4	546,5	535,4	3,8	0,1832
YGR130C	369,4	53,6	368,7	84,3	1,0	0,9892
YGR137W	77,1	26,6	133,2	89,3	1,7	0,2739
YGR139W	36,3	36,3	154,7	241,4	4,3	0,3693
YGR149W	7,5	3,4	2,8	3,4	0,4	0,0966
YGR151C	264,5	174,6	30,0	21,0	0,1	0,0372
YGR153W	4,9	6,5	1,2	2,4	0,2	0,3185
YGR161W-C	13,3	9,3	8,2	11,6	0,6	0,5199
YGR164W	170,1	44,8	56,6	41,5	0,3	0,0099
YGR168C	90,6	51,9	48,1	35,8	0,5	0,2260
YGR169C-A	594,7	376,1	499,2	381,9	0,8	0,7339
YGR182C	73,9	21,5	181,0	115,9	2,4	0,1194
YGR201C	30,1	16,4	7,8	12,4	0,3	0,0727
YGR205W	201,8	179,8	217,7	167,7	1,1	0,9011
YGR207C	35,3	37,3	25,7	28,2	0,7	0,6957
YGR210C	111,3	55,8	78,9	58,6	0,7	0,4529

YGR235C	30,7	20,4	125,2	193,2	4,1	0,3684
YGR237C	15,4	10,9	34,4	31,9	2,2	0,3018
YGR240C-A	431,4	158,4	613,3	299,7	1,4	0,3245
YGR250C	32,5	12,7	51,9	39,2	1,6	0,3824
YGR251W	44,4	26,9	123,8	95,0	2,8	0,1588
YGR265W	15,7	12,5	33,7	25,0	2,1	0,2442
YGR266W	123,8	47,7	60,5	41,3	0,5	0,0918
YGR269W	0,0	0,0	0,0	0,0	#DIV/0!	#DIV/0!
YGR273C	0,0	0,0	0,0	0,0	#DIV/0!	#DIV/0!
YGR283C	91,1	56,6	413,1	399,2	4,5	0,1613
YGR290W	3,3	4,5	15,2	13,0	4,6	0,1355
YHB1	54,9	18,8	24,0	18,6	0,4	0,0585
YHC1	72,7	41,0	72,0	49,9	1,0	0,9832
YHC3	65,6	26,5	37,4	25,5	0,6	0,1775
YHI9	29,9	24,0	21,5	15,8	0,7	0,5792
YHK8	164,3	146,1	77,7	111,3	0,5	0,3821
YHL008C	66,7	9,1	82,6	56,1	1,2	0,5960
YHL009W-A	23,1	14,7	43,9	32,4	1,9	0,2877
YHL009W-B	121,1	61,8	228,2	42,6	1,9	0,0291
YHL012W	578,8	368,0	173,8	94,2	0,3	0,0769
YHL017W	27,1	18,1	37,7	33,8	1,4	0,6016
YHL018W	4,1	3,0	0,0	0,0	0,0	0,0356
YHL026C	59,3	18,4	30,4	22,7	0,5	0,0956
YHL037C	6,1	5,5	2,2	2,5	0,4	0,2486
YHL042W	8,6	9,5	70,7	37,0	8,2	0,0174
YHL044W	1,8	3,5	3,1	2,3	1,8	0,5481
YHL045W	1,2	2,4	0,0	0,0	0,0	0,3559
YHL050C	0,0	0,0	0,0	0,0	#DIV/0!	#DIV/0!
YHM2	241,2	152,4	84,0	74,7	0,3	0,1134
YHP1	62,0	18,1	22,8	17,6	0,4	0,0209
YHR003C	15,7	4,6	15,7	11,6	1,0	0,9936
YHR007C-A	429,6	58,1	1517,9	151,9	3,5	0,0000
YHR009C	241,6	141,9	363,5	130,1	1,5	0,2523
YHR020W	173,6	38,4	270,6	54,3	1,6	0,0268
YHR022C	126,1	77,3	64,2	66,4	0,5	0,2701
YHR022C-A	28,5	38,7	151,1	178,4	5,3	0,2279
YHR032W-A	0,0	0,0	0,0	0,0	#DIV/0!	#DIV/0!
YHR033W	23,4	5,6	18,1	13,2	0,8	0,4808
YHR035W	12,6	10,5	20,8	19,2	1,6	0,4833
YHR045W	12,1	13,8	5,7	4,4	0,5	0,4096
YHR050W-A	98,9	80,2	102,8	98,6	1,0	0,9537
YHR054C	302,7	90,3	558,8	568,1	1,8	0,4075
YHR071C-A	29,1	5,2	23,3	16,7	0,8	0,5349
YHR073W-A	0,9	1,8	1,3	2,5	1,4	0,8118
YHR078W	42,8	29,1	82,8	58,1	1,9	0,2643
YHR080C	9,7	9,8	14,7	11,9	1,5	0,5411
YHR086W-A	5,7	1,8	1,0	2,0	0,2	0,0122
YHR095W	6,3	8,6	3,4	4,5	0,5	0,5640
YHR097C	19,5	1,7	16,3	12,6	0,8	0,6333
YHR112C	33,0	14,8	47,1	40,0	1,4	0,5328

YHR113W	24,6	9,7	22,8	17,4	0,9	0,8575
YHR122W	160,1	112,4	322,6	257,2	2,0	0,2911
YHR125W	1,9	2,8	2,6	5,3	1,4	0,8193
YHR127W	57,6	11,7	20,6	18,5	0,4	0,0149
YHR130C	5,7	4,6	10,2	8,9	1,8	0,4022
YHR131C	8,0	5,1	2,0	4,0	0,2	0,1089
YHR138C	11,6	5,2	27,0	19,8	2,3	0,1834
YHR139C-A	32,8	14,1	49,6	67,5	1,5	0,6430
YHR140W	6,9	5,3	2,9	3,7	0,4	0,2609
YHR145C	0,0	0,0	0,0	0,0	#DIV/0!	#DIV/0!
YHR159W	13,9	8,0	12,3	8,9	0,9	0,7976
YHR175W-A	142,0	40,4	116,0	115,7	0,8	0,6864
YHR177W	36,7	38,4	20,4	26,4	0,6	0,5090
YHR180W	82,9	56,4	260,0	284,9	3,1	0,2684
YHR182W	31,5	8,4	86,0	117,6	2,7	0,3910
YHR192W	31,1	26,5	19,3	19,0	0,6	0,4962
YHR202W	4,2	0,6	1,6	3,3	0,4	0,1753
YHR210C	21,4	11,5	20,2	14,4	0,9	0,9056
YHR214C-B	23,4	18,6	20,0	19,5	0,9	0,8097
YHR217C	0,0	0,0	0,0	0,0	#DIV/0!	#DIV/0!
YIA6	19,1	9,0	26,1	23,9	1,4	0,6041
YIF1	30,6	10,4	17,9	20,9	0,6	0,3181
YIG1	9,9	11,0	0,7	1,3	0,1	0,1490
YIH1	76,0	18,6	45,4	30,6	0,6	0,1380
YIL001W	7,2	3,9	9,8	9,9	1,4	0,6496
YIL002W-A	0,0	0,0	0,0	0,0	#DIV/0!	#DIV/0!
YIL012W	0,9	1,8	2,3	2,6	2,6	0,4205
YIL014C-A	50,7	54,5	84,5	137,1	1,7	0,6630
YIL024C	3,5	4,0	0,0	0,0	0,0	0,1341
YIL025C	60,1	37,3	6,4	8,4	0,1	0,0309
YIL028W	3,3	2,3	1,6	3,3	0,5	0,4293
YIL029C	3,5	2,4	6,5	4,6	1,9	0,2865
YIL030W-A	0,0	0,0	0,0	0,0	#DIV/0!	#DIV/0!
YIL032C	14,2	14,1	6,8	6,9	0,5	0,3814
YIL046W-A	1,0	2,0	0,0	0,0	0,0	0,3559
YIL047C-A	96,2	68,7	44,5	36,6	0,5	0,2322
YIL054W	1,9	2,3	3,0	4,5	1,6	0,6677
YIL055C	21,6	26,9	14,2	11,9	0,7	0,6326
YIL058W	4,7	5,6	0,0	0,0	0,0	0,1486
YIL067C	1,7	2,2	4,8	6,7	2,8	0,4132
YIL077C	41,5	43,8	126,9	182,7	3,1	0,3985
YIL080W	108,5	34,7	72,2	54,8	0,7	0,3055
YIL086C	75,6	31,7	304,7	263,4	4,0	0,1349
YIL092W	30,6	25,1	117,6	206,9	3,8	0,4359
YIL096C	15,5	17,6	6,8	7,9	0,4	0,3993
YIL102C	32,9	9,1	76,7	56,1	2,3	0,1747
YIL102C-A	28,1	23,3	67,8	58,5	2,4	0,2548
YIL108W	11,2	6,0	18,7	14,8	1,7	0,3814
YIL134C-A	4,5	5,8	5,6	8,1	1,2	0,8425
YIL141W	3,8	3,4	10,7	8,5	2,8	0,1869

YIL151C	33,3	21,8	25,8	19,2	0,8	0,6231
YIL152W	32,3	13,8	21,7	17,2	0,7	0,3735
YIL156W-B	41,2	28,8	33,1	38,2	0,8	0,7456
YIL161W	70,3	64,0	101,8	100,8	1,4	0,6165
YIL163C	99,3	61,0	91,2	63,6	0,9	0,8599
YIL165C	50,4	23,3	85,1	82,5	1,7	0,4498
YIL166C	69,5	39,2	129,6	113,0	1,9	0,3538
YIL168W	0,9	1,8	1,0	2,0	1,1	0,9577
YIL169C	45,8	48,8	301,8	196,4	6,6	0,0446
YIL171W	39,7	12,9	59,0	41,2	1,5	0,4061
YIL171W-A	193,8	70,7	67,4	92,0	0,3	0,0722
YIL174W	2,6	5,2	0,0	0,0	0,0	0,3559
YIM1	11,8	15,9	9,7	11,1	0,8	0,8335
YIM2	6,6	6,0	9,7	15,0	1,5	0,7132
YIP1	105,1	111,5	191,2	305,7	1,8	0,6160
YIP3	50,2	12,4	150,5	167,8	3,0	0,2782
YIP4	59,2	22,5	57,4	39,4	1,0	0,9394
YIP5	14,3	8,7	21,2	19,8	1,5	0,5528
YIR007W	7,9	6,7	7,0	5,4	0,9	0,8411
YIR014W	29,9	14,2	37,5	27,9	1,3	0,6433
YIR016W	0,0	0,0	1,3	2,6	#DIV/0!	0,3559
YIR018C-A	426,6	210,8	185,0	125,0	0,4	0,0960
YIR020C	8,1	3,4	7,6	5,6	0,9	0,8955
YIR020W-A	6,9	6,8	14,4	13,1	2,1	0,3442
YIR021W-A	11,0	8,1	7,3	8,6	0,7	0,5516
YIR024C	17,5	12,0	15,1	10,7	0,9	0,7715
YIR035C	283,9	79,2	189,2	77,7	0,7	0,1389
YIR042C	42,9	22,5	11,2	10,4	0,3	0,0424
YIR043C	36,2	28,8	22,0	16,4	0,6	0,4236
YIR044C	2,4	4,7	0,0	0,0	0,0	0,3559
YJL007C	126,4	136,7	24,8	19,1	0,2	0,1914
YJL016W	63,1	32,6	77,6	69,0	1,2	0,7179
YJL022W	1,1	2,1	0,0	0,0	0,0	0,3559
YJL027C	0,0	0,0	0,0	0,0	#DIV/0!	#DIV/0!
YJL028W	6,3	7,8	1,2	2,4	0,2	0,2566
YJL043W	4,3	5,2	9,6	19,1	2,2	0,6160
YJL045W	46,5	12,2	27,4	19,0	0,6	0,1431
YJL047C-A	12,6	13,6	6,1	9,4	0,5	0,4642
YJL049W	9,7	6,8	5,3	5,3	0,6	0,3550
YJL052C-A	17,9	14,9	4,9	9,9	0,3	0,1976
YJL055W	92,4	104,3	105,6	117,9	1,1	0,8729
YJL068C	35,7	17,5	21,3	14,6	0,6	0,2534
YJL070C	8,1	6,6	15,9	11,0	2,0	0,2709
YJL077W-B	1,7	3,4	0,0	0,0	0,0	0,3559
YJL086C	0,0	0,0	0,0	0,0	#DIV/0!	#DIV/0!
YJL107C	2,8	3,4	0,0	0,0	0,0	0,1458
YJL113W	0,0	0,0	2,4	2,8	#DIV/0!	0,1343
YJL114W	0,0	0,0	3,1	3,7	#DIV/0!	0,1409
YJL127C-B	109,8	24,7	362,1	124,1	3,3	0,0072
YJL132W	34,1	14,5	42,9	50,1	1,3	0,7478

YJL133C-A	146,3	72,8	158,6	152,2	1,1	0,8894
YJL135W	7,2	2,5	1,3	2,6	0,2	0,0179
YJL144W	5,3	2,6	19,8	17,3	3,7	0,1502
YJL150W	41,6	46,3	104,5	124,1	2,5	0,3785
YJL152W	14,5	16,1	1,8	2,3	0,1	0,1691
YJL156W-A	163,1	147,7	286,9	174,1	1,8	0,3199
YJL160C	4,9	8,2	2,3	2,6	0,5	0,5688
YJL163C	115,3	98,8	712,6	514,4	6,2	0,0627
YJL171C	5,5	4,1	11,8	8,9	2,1	0,2448
YJL181W	5,7	4,5	0,0	0,0	0,0	0,0441
YJL185C	41,2	20,3	4,5	5,3	0,1	0,0129
YJL193W	13,3	6,9	17,9	13,0	1,3	0,5558
YJL206C	15,0	4,6	24,7	26,1	1,6	0,4920
YJL213W	14,9	13,7	113,4	95,1	7,6	0,0862
YJL218W	4,2	4,9	5,5	6,4	1,3	0,7680
YJR003C	8,2	6,3	16,2	17,0	2,0	0,4115
YJR005C-A	4,8	5,7	1,8	3,6	0,4	0,3997
YJR008W	10,7	7,4	13,1	15,5	1,2	0,7900
YJR011C	47,0	60,4	8,5	6,3	0,2	0,2514
YJR012C	201,7	101,2	279,1	171,3	1,4	0,4659
YJR015W	58,8	48,1	48,9	45,0	0,8	0,7730
YJR027W	72,0	43,8	71,0	64,7	1,0	0,9804
YJR030C	74,6	6,2	51,4	34,5	0,7	0,2320
YJR039W	36,0	30,7	297,7	307,9	8,3	0,1416
YJR054W	62,9	16,8	27,0	22,6	0,4	0,0433
YJR056C	69,8	42,5	47,4	32,9	0,7	0,4352
YJR061W	252,7	105,2	123,3	135,5	0,5	0,1821
YJR085C	146,0	104,7	221,1	208,3	1,5	0,5434
YJR087W	386,4	267,0	309,7	217,3	0,8	0,6716
YJR096W	97,4	17,5	119,9	97,0	1,2	0,6631
YJR098C	39,8	18,7	13,6	10,4	0,3	0,0495
YJR107W	313,1	145,1	652,7	445,2	2,1	0,1971
YJR111C	54,0	70,1	75,7	70,5	1,4	0,6775
YJR112W-A	31,4	30,0	9,2	11,2	0,3	0,2166
YJR115W	9,2	7,2	5,8	7,8	0,6	0,5491
YJR116W	53,2	27,7	92,1	104,1	1,7	0,4978
YJR120W	2,2	2,5	48,6	35,3	22,5	0,0391
YJR124C	51,5	7,7	35,0	27,9	0,7	0,2979
YJR128W	11,9	4,8	0,0	0,0	0,0	0,0026
YJR129C	54,1	37,6	86,2	99,9	1,6	0,5699
YJR141W	32,8	20,7	16,9	21,4	0,5	0,3238
YJR142W	36,5	28,6	32,8	32,2	0,9	0,8684
YJR146W	11,7	10,2	33,4	36,0	2,9	0,2901
YJR149W	87,8	14,4	34,3	23,7	0,4	0,0083
YJR151W-A	16,4	14,2	3,0	5,9	0,2	0,1317
YJR154W	0,9	1,8	7,8	9,0	8,5	0,1865
YJR157W	15,5	4,0	8,7	6,1	0,6	0,1113
YJR162C	4,8	5,6	1,6	3,3	0,3	0,3714
YJU2	6,1	6,8	7,4	7,3	1,2	0,8049
YJU3	34,5	27,5	34,9	34,2	1,0	0,9847

YKE4	28,8	6,0	84,8	83,3	2,9	0,2289
YKL018C-A	29,4	30,7	26,4	32,7	0,9	0,8971
YKL023W	160,8	43,9	258,4	20,3	1,6	0,0069
YKL027W	29,0	12,4	24,0	25,2	0,8	0,7309
YKL033W-A	471,0	303,9	260,5	36,2	0,6	0,2179
YKL044W	15,6	25,9	0,0	0,0	0,0	0,2721
YKL047W	170,4	45,7	50,2	44,5	0,3	0,0093
YKL050C	14,5	9,7	15,5	12,8	1,1	0,9012
YKL063C	411,3	243,6	183,4	152,6	0,4	0,1640
YKL066W	788,8	369,2	1287,4	702,5	1,6	0,2556
YKL068W-A	229,7	80,7	675,8	126,8	2,9	0,0010
YKL069W	84,8	55,1	100,0	69,6	1,2	0,7447
YKL070W	98,6	67,1	554,3	606,0	5,6	0,1856
YKL071W	5,7	1,7	10,8	9,8	1,9	0,3416
YKL075C	21,0	16,4	24,9	22,6	1,2	0,7862
YKL077W	71,8	21,1	41,5	34,6	0,6	0,1854
YKL091C	44,9	30,3	25,2	17,7	0,6	0,3057
YKL097C	2,9	5,9	1,6	3,3	0,6	0,7150
YKL100C	16,4	10,8	15,9	11,2	1,0	0,9466
YKL102C	5,3	6,2	5,3	4,2	1,0	0,9891
YKL105C	36,4	25,1	69,7	48,5	1,9	0,2681
YKL107W	23,5	23,1	12,6	10,8	0,5	0,4231
YKL118W	8,5	6,5	7,1	8,9	0,8	0,8156
YKL133C	28,6	6,1	19,0	14,2	0,7	0,2616
YKL151C	209,3	131,3	253,9	70,5	1,2	0,5713
YKL162C	1,6	1,9	110,9	211,4	70,3	0,3410
YKL187C	13,9	13,9	19,7	15,1	1,4	0,5972
YKL202W	639,0	183,1	287,9	238,9	0,5	0,0584
YKL222C	11,2	19,5	4,8	7,9	0,4	0,5657
YKL225W	85,8	48,3	12,5	18,0	0,1	0,0295
YKR005C	13,0	6,5	82,7	138,2	6,4	0,3527
YKR011C	137,6	15,7	365,2	136,7	2,7	0,0163
YKR012C	4,6	1,7	0,0	0,0	0,0	0,0016
YKR015C	74,6	43,4	102,9	68,9	1,4	0,5130
YKR017C	13,0	6,6	13,4	12,8	1,0	0,9612
YKR018C	88,2	57,2	164,9	67,1	1,9	0,1324
YKR023W	69,5	44,2	61,9	43,8	0,9	0,8150
YKR040C	1,5	1,8	5,9	7,1	3,9	0,2756
YKR043C	84,3	48,1	45,2	47,9	0,5	0,2922
YKR045C	7,6	7,0	2,0	4,0	0,3	0,2127
YKR051W	26,3	11,6	24,7	21,6	0,9	0,8982
YKR070W	86,7	24,6	36,1	24,2	0,4	0,0260
YKR073C	0,8	1,5	7,7	7,0	10,2	0,0987
YKR075C	47,1	35,9	13,2	9,2	0,3	0,1171
YKR078W	7,5	6,3	2,4	4,7	0,3	0,2433
YKR096W	59,2	21,3	70,3	56,6	1,2	0,7263
YKR104W	1,8	3,5	1,9	3,8	1,1	0,9602
YKR106W	84,5	57,1	118,2	157,9	1,4	0,7021
YKT6	114,9	66,5	62,1	58,3	0,5	0,2769
YKU70	20,2	8,4	79,2	76,6	3,9	0,1768

YKU80	4,5	3,9	7,9	9,7	1,8	0,5349
YLF2	117,6	38,7	152,7	137,7	1,3	0,6404
YLH47	4,2	3,9	0,0	0,0	0,0	0,0751
YLL007C	11,4	8,0	2,8	3,4	0,2	0,0930
YLL017W	29,3	16,7	10,8	14,4	0,4	0,1430
YLL020C	2,3	2,1	6,1	7,3	2,6	0,3615
YLL032C	8,5	8,8	3,7	3,1	0,4	0,3423
YLL053C	0,0	0,0	0,0	0,0	#DIV/0!	#DIV/0!
YLL054C	5,8	4,4	11,7	8,5	2,0	0,2642
YLL056C	35,8	8,1	19,2	13,2	0,5	0,0767
YLL058W	32,4	23,9	13,3	10,8	0,4	0,1962
YLL059C	0,6	1,2	5,9	7,6	10,0	0,2172
YLL065W	0,0	0,0	0,0	0,0	#DIV/0!	#DIV/0!
YLL066C	211,2	133,6	145,8	81,9	0,7	0,4357
YLL066W-B	0,0	0,0	0,7	1,3	#DIV/0!	0,3559
YLR001C	37,1	13,7	30,6	25,1	0,8	0,6684
YLR012C	154,5	92,7	158,5	112,6	1,0	0,9575
YLR030W	193,4	147,1	37,8	48,9	0,2	0,0915
YLR031W	17,9	8,7	3,4	2,3	0,2	0,0186
YLR035C-A	67,0	24,6	672,2	294,7	10,0	0,0064
YLR036C	12,5	12,4	20,9	28,3	1,7	0,6053
YLR040C	6,6	8,7	20,6	13,9	3,1	0,1376
YLR042C	10,4	2,3	13,7	14,6	1,3	0,6746
YLR046C	15,0	7,7	8,7	6,9	0,6	0,2634
YLR049C	47,7	8,5	12,1	10,6	0,3	0,0020
YLR050C	117,9	70,9	14,7	11,0	0,1	0,0281
YLR057W	21,2	14,2	16,3	14,0	0,8	0,6394
YLR063W	55,0	47,2	94,9	119,1	1,7	0,5564
YLR065C	50,7	35,8	28,6	22,8	0,6	0,3362
YLR072W	8,9	8,4	29,1	45,1	3,3	0,4124
YLR099W-A	18,5	9,7	9,3	7,0	0,5	0,1751
YLR101C	8,0	5,0	4,6	5,7	0,6	0,4114
YLR108C	18,2	17,1	20,8	15,3	1,1	0,8276
YLR111W	1,2	2,4	1,9	3,8	1,6	0,7738
YLR112W	120,8	49,7	69,4	46,7	0,6	0,1829
YLR118C	29,8	34,1	10,3	11,8	0,3	0,3230
YLR120W-A	73,8	51,7	88,2	85,8	1,2	0,7832
YLR122C	24,0	14,5	35,7	27,6	1,5	0,4807
YLR125W	1,6	2,1	11,5	8,8	7,2	0,0714
YLR126C	232,7	249,8	170,2	43,0	0,7	0,6396
YLR132C	4,2	3,4	9,8	8,1	2,4	0,2433
YLR137W	9,1	4,8	2,3	2,6	0,2	0,0467
YLR140W	0,0	0,0	0,0	0,0	#DIV/0!	#DIV/0!
YLR143W	8,6	10,3	12,9	12,3	1,5	0,6113
YLR149C	24,7	8,2	10,3	7,7	0,4	0,0425
YLR152C	23,3	13,9	79,5	47,3	3,4	0,0625
YLR154C-G	27,1	14,7	38,8	25,9	1,4	0,4620
YLR154C-H	71,8	27,6	26,1	23,6	0,4	0,0455
YLR154W-A	1583,7	2404,0	1569,7	2232,5	1,0	0,9935
YLR154W-B	814,8	1352,0	981,1	1378,7	1,2	0,8689

YLR154W-E	13464,9	13559,1	6876,2	7092,9	0,5	0,4222
YLR154W-F	957,7	1318,4	293,1	311,3	0,3	0,3644
YLR156W	0,9	1,8	0,7	1,3	0,7	0,8285
YLR162W	20316,7	29017,7	34728,1	41549,0	1,7	0,5902
YLR173W	4,0	7,9	7,9	5,3	2,0	0,4455
YLR177W	10,4	8,3	14,6	10,2	1,4	0,5386
YLR179C	210,5	72,4	215,5	150,0	1,0	0,9544
YLR202C	32,8	12,3	9,9	11,5	0,3	0,0350
YLR211C	0,0	0,0	0,0	0,0	#DIV/0!	#DIV/0!
YLR222C-A	49,1	31,3	113,0	73,9	2,3	0,1626
YLR224W	82,7	71,3	61,6	42,1	0,7	0,6290
YLR225C	42,0	20,4	153,9	37,8	3,7	0,0020
YLR227W-B	13,1	9,5	21,1	20,9	1,6	0,5146
YLR241W	26,6	12,2	95,7	138,8	3,6	0,3592
YLR243W	30,0	21,9	26,1	26,6	0,9	0,8262
YLR252W	51,7	15,0	121,4	102,1	2,3	0,2256
YLR253W	8,7	8,4	8,5	10,6	1,0	0,9759
YLR255C	5,3	5,6	6,5	10,0	1,2	0,8502
YLR256W-A	53,4	12,3	75,3	52,2	1,4	0,4441
YLR257W	102,7	43,1	171,8	85,4	1,7	0,1988
YLR264C-A	39169,6	14687,9	24177,8	10325,3	0,6	0,1460
YLR271W	42,2	26,5	68,6	51,2	1,6	0,3951
YLR278C	105,9	79,7	81,3	87,8	0,8	0,6927
YLR283W	8,7	8,8	2,4	2,8	0,3	0,2243
YLR285C-A	1525,8	212,4	1433,5	193,3	0,9	0,5442
YLR287C	99,5	83,4	160,0	67,7	1,6	0,3026
YLR290C	38,4	28,1	44,2	33,7	1,2	0,8009
YLR294C	14,2	10,4	143,2	172,7	10,1	0,1866
YLR296W	16,2	26,3	3,5	4,4	0,2	0,3757
YLR297W	79,7	34,0	218,4	157,4	2,7	0,1359
YLR301W	4,2	7,0	120,8	241,5	28,4	0,3721
YLR302C	0,0	0,0	0,0	0,0	#DIV/0!	#DIV/0!
YLR307C-A	0,0	0,0	0,0	0,0	#DIV/0!	#DIV/0!
YLR311C	11,0	9,4	26,4	23,3	2,4	0,2637
YLR312C	3,4	2,5	0,0	0,0	0,0	0,0333
YLR326W	47,0	16,4	41,6	34,1	0,9	0,7849
YLR342W-A	171,6	15,2	40,3	30,9	0,2	0,0003
YLR345W	13,6	7,5	23,9	16,6	1,8	0,3045
YLR346C	10,2	8,8	5,8	7,8	0,6	0,4851
YLR352W	41,0	13,9	33,4	28,6	0,8	0,6464
YLR361C-A	30,8	29,6	38,0	33,4	1,2	0,7576
YLR365W	0,3	0,6	0,7	1,3	2,2	0,6428
YLR407W	107,8	79,0	51,7	40,1	0,5	0,2521
YLR410W-A	44,8	38,9	32,0	28,0	0,7	0,6120
YLR410W-B	1,5	3,0	75,9	151,8	51,1	0,3648
YLR413W	40,7	22,8	5,9	4,8	0,1	0,0244
YLR415C	1,4	1,6	5,6	11,2	4,2	0,4807
YLR419W	52,1	23,5	14,1	9,5	0,3	0,0239
YLR422W	37,7	8,0	51,6	37,6	1,4	0,4990
YLR426W	17,4	14,8	15,3	14,1	0,9	0,8394

YLR445W	19,9	19,7	166,4	284,8	8,4	0,3441
YLR446W	59,6	32,1	67,8	53,9	1,1	0,8018
YLR455W	234,4	99,7	147,2	101,0	0,6	0,2648
YLR456W	10,2	8,4	37,7	28,3	3,7	0,1119
YLR460C	25,9	7,4	10,6	9,6	0,4	0,0453
YLR466C-B	18,3	6,8	12,7	12,9	0,7	0,4721
YMC1	8,6	5,4	8,8	10,3	1,0	0,9667
YMC2	44,3	31,4	27,8	28,7	0,6	0,4693
YMD8	218,3	124,1	77,6	68,2	0,4	0,0942
YME1	51,7	17,8	143,4	135,7	2,8	0,2284
YME2	81,3	20,5	31,1	30,8	0,4	0,0348
YML002W	151,7	26,5	115,4	85,4	0,8	0,4478
YML003W	0,0	0,0	1,6	3,3	#DIV/0!	0,3559
YML007C-A	0,9	1,8	1,6	3,3	1,9	0,6958
YML009W-B	9,5	8,7	8,7	10,2	0,9	0,9087
YML018C	236,7	112,4	234,5	180,9	1,0	0,9835
YML020W	25,4	4,3	19,6	14,7	0,8	0,4792
YML037C	11,2	2,0	4,5	5,4	0,4	0,0597
YML045W	374,2	144,1	337,1	219,6	0,9	0,7873
YML053C	258,3	140,5	206,3	139,9	0,8	0,6190
YML054C-A	17,9	5,6	9,9	8,4	0,6	0,1630
YML079W	47,3	45,2	45,2	41,8	1,0	0,9490
YML081W	39,3	24,6	16,8	13,3	0,4	0,1582
YML082W	16,3	9,4	14,2	12,0	0,9	0,7848
YML083C	90,2	57,1	18,7	25,0	0,2	0,0615
YML089C	0,6	1,2	0,7	1,3	1,1	0,9379
YML096W	173,7	148,2	304,0	293,5	1,7	0,4584
YML108W	30,7	16,2	20,1	15,7	0,7	0,3808
YML116W-A	31,0	18,8	19,7	14,7	0,6	0,3834
YML119W	70,4	96,3	275,7	160,8	3,9	0,0711
YML122C	248,0	90,5	52,4	48,2	0,2	0,0088
YML131W	16,2	8,5	21,4	15,4	1,3	0,5746
YML6	72,1	26,2	75,2	42,6	1,0	0,9079
YMR007W	962,0	863,3	1076,4	601,5	1,1	0,8351
YMR010W	214,1	81,7	198,2	81,1	0,9	0,7925
YMR013W-A	1,7	3,4	11,6	11,7	6,8	0,1570
YMR018W	119,8	73,1	104,0	106,0	0,9	0,8147
YMR027W	37,8	23,0	24,2	29,8	0,6	0,4979
YMR031C	16,0	14,4	24,8	20,8	1,5	0,5134
YMR034C	9,3	15,5	4,1	4,8	0,4	0,5481
YMR046W-A	2,1	4,1	2,0	4,0	1,0	0,9789
YMR074C	9,9	7,7	6,2	7,3	0,6	0,5088
YMR082C	5,8	8,3	1,6	3,3	0,3	0,3912
YMR084W	1,1	2,1	0,0	0,0	0,0	0,3559
YMR085W	1,5	3,0	0,0	0,0	0,0	0,3559
YMR086W	28,2	5,4	26,4	21,5	0,9	0,8759
YMR087W	30,1	13,6	15,2	11,7	0,5	0,1476
YMR090W	19,5	16,5	60,2	80,9	3,1	0,3623
YMR099C	84,6	44,1	106,9	84,2	1,3	0,6550
YMR1	22,0	17,3	13,8	15,2	0,6	0,5012

YMR102C	61,3	28,3	15,8	11,9	0,3	0,0252
YMR103C	11,3	3,5	30,1	20,2	2,7	0,1170
YMR111C	41,8	5,9	130,7	89,1	3,1	0,0937
YMR114C	14,7	11,5	78,2	72,0	5,3	0,1321
YMR119W-A	40,5	13,5	24,7	18,1	0,6	0,2109
YMR122C	0,0	0,0	0,0	0,0	#DIV/0!	#DIV/0!
YMR122W-A	1383,3	806,4	4346,4	792,4	3,1	0,0019
YMR124W	74,0	29,4	85,8	59,6	1,2	0,7335
YMR130W	33,7	2,6	25,2	19,6	0,7	0,4216
YMR134W	27,9	21,5	20,3	16,0	0,7	0,5893
YMR135W-A	0,6	1,2	0,0	0,0	0,0	0,3559
YMR144W	40,8	27,6	9,8	6,6	0,2	0,0717
YMR147W	45,3	32,7	11,7	13,7	0,3	0,1064
YMR155W	16,4	6,8	80,5	121,4	4,9	0,3318
YMR158C-A	10,2	4,2	10,4	7,1	1,0	0,9735
YMR160W	17,5	16,9	16,6	12,5	1,0	0,9388
YMR166C	14,2	7,4	13,8	12,9	1,0	0,9647
YMR175W-A	18,6	21,1	328,0	300,1	17,7	0,0854
YMR178W	80,4	8,7	22,6	17,7	0,3	0,0011
YMR181C	54,7	22,1	68,5	49,3	1,3	0,6276
YMR182W-A	72,2	82,8	45,4	38,3	0,6	0,5783
YMR185W	67,6	43,6	47,9	33,1	0,7	0,4973
YMR187C	21,8	19,4	9,8	16,5	0,4	0,3803
YMR194C-A	47,6	43,8	259,4	187,7	5,4	0,0703
YMR196W	42,6	7,5	9,4	12,0	0,2	0,0034
YMR206W	78,5	43,0	25,8	20,5	0,3	0,0689
YMR209C	20,4	8,2	13,7	10,0	0,7	0,3390
YMR210W	80,0	41,6	102,3	80,5	1,3	0,6410
YMR221C	61,2	24,9	105,1	96,2	1,7	0,4110
YMR226C	39,8	19,6	36,6	32,9	0,9	0,8712
YMR230W-A	23,1	13,9	3,6	7,3	0,2	0,0471
YMR244W	2,1	2,5	1,3	2,6	0,6	0,6833
YMR247W-A	4,5	5,3	49,3	41,7	10,9	0,0772
YMR252C	14,6	11,1	5,5	7,8	0,4	0,2282
YMR253C	42,9	15,9	110,7	78,9	2,6	0,1431
YMR254C	16,7	10,8	46,9	56,6	2,8	0,3347
YMR258C	13,1	8,3	17,6	13,6	1,3	0,5894
YMR259C	14,8	3,3	52,9	44,2	3,6	0,1367
YMR262W	6,9	5,7	1,3	2,6	0,2	0,1270
YMR265C	3,8	2,8	4,9	6,7	1,3	0,7782
YMR279C	19,5	19,5	16,4	11,6	0,8	0,7929
YMR290W-A	17,5	7,4	20,2	17,4	1,2	0,7883
YMR291W	19,3	15,7	12,8	9,3	0,7	0,4996
YMR295C	93,9	70,5	129,6	149,9	1,4	0,6816
YMR31	44,3	38,2	50,0	46,4	1,1	0,8551
YMR310C	128,9	24,0	50,2	40,1	0,4	0,0152
YMR315W	144,1	65,8	179,1	20,3	1,2	0,3480
YMR315W-A	66,6	59,2	238,1	220,0	3,6	0,1829
YMR317W	20,1	10,3	31,6	24,4	1,6	0,4184
YMR320W	6,5	5,7	9,2	7,1	1,4	0,5817

YMR321C	1374,8	625,5	1277,9	840,9	0,9	0,8594
YND1	368,5	113,3	525,7	161,3	1,4	0,1620
YNG1	360,6	257,5	70,1	49,0	0,2	0,0685
YNG2	68,9	40,0	140,5	61,1	2,0	0,0973
YNK1	440,0	121,7	470,2	215,7	1,1	0,8156
YNL010W	352,9	81,7	207,7	141,9	0,6	0,1265
YNL011C	5,4	6,6	88,3	143,7	16,4	0,2925
YNL018C	10,3	8,4	3,8	5,0	0,4	0,2363
YNL019C	46,9	6,2	118,8	58,2	2,5	0,0493
YNL022C	61,5	31,9	119,1	90,2	1,9	0,2736
YNL024C	14,3	9,6	107,2	169,0	7,5	0,3144
YNL033W	23,4	25,6	38,8	60,0	1,7	0,6527
YNL034W	2,5	2,9	4,0	3,0	1,6	0,4926
YNL035C	28,7	10,6	31,0	22,6	1,1	0,8599
YNL040W	31,3	19,5	37,3	37,9	1,2	0,7883
YNL042W-B	43,0	54,9	78,4	104,8	1,8	0,5714
YNL046W	58,7	31,4	153,0	187,4	2,6	0,3595
YNL050C	30,5	21,6	42,5	30,3	1,4	0,5412
YNL058C	119,5	26,7	325,0	111,5	2,7	0,0116
YNL067W-B	0,0	0,0	0,0	0,0	#DIV/0!	#DIV/0!
YNL092W	21,3	14,5	27,2	18,4	1,3	0,6361
YNL095C	132,1	121,9	174,4	131,5	1,3	0,6541
YNL097C-B	21,5	19,9	71,5	65,7	3,3	0,1958
YNL103W-A	96,1	87,0	263,1	171,9	2,7	0,1335
YNL108C	5,6	4,1	1,2	2,4	0,2	0,1115
YNL115C	19,4	8,3	17,4	16,3	0,9	0,8325
YNL122C	712,6	189,1	750,2	278,8	1,1	0,8312
YNL134C	53,0	10,7	215,4	122,4	4,1	0,0384
YNL143C	10,1	4,3	7,3	6,0	0,7	0,4823
YNL144C	19,1	7,2	146,0	145,7	7,6	0,1327
YNL146W	46,0	41,4	193,2	168,1	4,2	0,1401
YNL150W	2,5	3,0	0,0	0,0	0,0	0,1482
YNL155W	64,4	40,1	78,4	55,9	1,2	0,6982
YNL162W-A	54,2	20,4	10,5	7,7	0,2	0,0070
YNL165W	71,3	43,5	60,9	71,8	0,9	0,8129
YNL176C	33,5	20,9	20,0	15,7	0,6	0,3428
YNL179C	12,0	24,1	1,9	2,4	0,2	0,4352
YNL181W	20,5	16,2	0,7	1,3	0,0	0,0503
YNL184C	28,1	11,8	6,9	5,2	0,2	0,0169
YNL190W	1679,1	1157,9	2290,0	721,7	1,4	0,4051
YNL193W	4,3	3,7	4,1	2,9	1,0	0,9448
YNL194C	17,7	6,2	16,0	11,0	0,9	0,8007
YNL195C	4,5	5,4	5,1	4,3	1,1	0,8730
YNL200C	76,0	66,5	141,5	112,8	1,9	0,3553
YNL205C	5,8	4,3	2,5	2,9	0,4	0,2557
YNL208W	1,1	2,1	0,0	0,0	0,0	0,3559
YNL211C	7,5	5,4	1,8	2,3	0,2	0,1029
YNL217W	44,6	28,1	33,4	24,1	0,7	0,5674
YNL234W	13,8	1,7	6,8	5,9	0,5	0,0641
YNL247W	187,8	87,7	261,8	111,5	1,4	0,3365

YNL260C	18,2	11,3	3,6	5,6	0,2	0,0602
YNL277W-A	26,9	12,4	78,8	55,5	2,9	0,1179
YNL284C-A	4,4	3,2	2,5	2,9	0,6	0,4207
YNL284C-B	0,0	0,0	0,0	0,0	#DIV/0!	#DIV/0!
YNL285W	0,9	1,8	0,0	0,0	0,0	0,3559
YNL295W	7,9	1,4	1,3	2,6	0,2	0,0047
YNL303W	208,2	118,1	73,7	64,8	0,4	0,0927
YNL305C	7,8	6,5	14,0	16,7	1,8	0,5141
YNL320W	59,8	16,9	112,7	81,5	1,9	0,2503
YNL338W	20,3	9,0	12,8	14,9	0,6	0,4221
YNR014W	62,8	60,5	70,5	53,7	1,1	0,8542
YNR021W	226,9	48,6	144,7	97,6	0,6	0,1826
YNR029C	74,7	105,6	16,1	13,3	0,2	0,3130
YNR034W-A	16,8	7,5	40,0	39,0	2,4	0,2869
YNR040W	0,0	0,0	1,0	2,0	#DIV/0!	0,3559
YNR048W	4,0	2,9	6,7	8,1	1,7	0,5541
YNR061C	32,1	33,7	20,9	14,6	0,6	0,5614
YNR062C	5,7	6,7	12,4	9,4	2,2	0,2877
YNR063W	0,6	1,2	1,8	3,6	3,0	0,5489
YNR064C	203,7	128,6	500,2	268,4	2,5	0,0934
YNR065C	4,6	3,6	1,2	2,4	0,3	0,1613
YNR066C	0,0	0,0	5,6	8,1	#DIV/0!	0,2190
YNR068C	6,5	3,9	22,7	16,2	3,5	0,0986
YNR071C	58,1	26,2	53,0	51,6	0,9	0,8675
YNR073C	1,4	2,7	1,8	3,6	1,3	0,8633
YNR075C-A	10,5	11,3	1,0	2,0	0,1	0,1471
YOL013W-A	10,0	9,9	14,3	10,4	1,4	0,5730
YOL013W-B	10,3	7,5	10,9	15,5	1,1	0,9479
YOL014W	4,8	5,7	1,8	2,3	0,4	0,3730
YOL019W	38,1	37,9	4,8	6,7	0,1	0,1341
YOL019W-A	0,0	0,0	0,0	0,0	#DIV/0!	#DIV/0!
YOL024W	41,4	37,5	24,4	21,5	0,6	0,4594
YOL029C	8,6	7,5	12,1	15,0	1,4	0,6894
YOL035C	0,0	0,0	0,0	0,0	#DIV/0!	#DIV/0!
YOL036W	2,9	3,8	2,4	2,8	0,8	0,8562
YOL038C-A	26,6	22,3	35,5	35,5	1,3	0,6859
YOL047C	70,4	65,4	403,9	299,8	5,7	0,0727
YOL050C	1146,4	509,2	1069,5	685,4	0,9	0,8629
YOL057W	20,3	24,9	61,2	115,6	3,0	0,5146
YOL073C	34,1	11,3	73,4	61,5	2,2	0,2556
YOL075C	108,2	58,9	47,6	40,0	0,4	0,1398
YOL085C	50,7	22,7	27,2	20,2	0,5	0,1732
YOL086W-A	63,3	12,9	82,6	57,9	1,3	0,5389
YOL087C	35,0	17,8	50,3	34,1	1,4	0,4565
YOL092W	408,4	47,7	216,1	153,8	0,5	0,0542
YOL097W-A	7,5	6,6	2,3	4,6	0,3	0,2425
YOL098C	119,0	62,6	84,5	59,5	0,7	0,4552
YOL103W-A	189,0	30,0	230,5	67,9	1,2	0,3056
YOL107W	14,3	9,7	7,0	6,4	0,5	0,2546
YOL118C	27,5	29,1	45,2	40,7	1,6	0,5074

YOL131W	5,4	6,8	1,3	2,6	0,2	0,3094
YOL153C	55,6	18,7	118,9	92,0	2,1	0,2265
YOL155W-A	503,2	256,0	395,4	190,1	0,8	0,5240
YOL159C	8,7	2,9	18,2	12,9	2,1	0,2013
YOL159C-A	41,5	46,0	390,0	516,0	9,4	0,2271
YOL160W	269,2	47,3	238,7	44,9	0,9	0,3847
YOL162W	2,1	2,5	1,8	2,3	0,9	0,8837
YOL163W	0,0	0,0	1,9	3,8	#DIV/0!	0,3559
YOL164W-A	52,1	25,7	35,4	25,0	0,7	0,3870
YOL166C	13,2	9,8	4,4	5,3	0,3	0,1683
YOP1	163,8	92,7	257,0	68,9	1,6	0,1579
YOR008C-A	0,6	1,2	0,0	0,0	0,0	0,3559
YOR011W-A	59,9	37,7	47,4	72,7	0,8	0,7706
YOR012W	274,9	312,3	91,8	108,3	0,3	0,3103
YOR015W	54,3	24,4	29,4	23,3	0,5	0,1911
YOR019W	107,2	69,7	181,9	72,5	1,7	0,1874
YOR022C	33,9	13,0	35,9	43,8	1,1	0,9341
YOR032W-A	24,2	17,6	8,3	9,0	0,3	0,1588
YOR034C-A	20,5	12,4	25,1	21,2	1,2	0,7210
YOR050C	119,8	141,2	131,9	159,6	1,1	0,9129
YOR052C	83,2	42,8	129,3	118,0	1,6	0,4904
YOR059C	45,1	29,9	142,8	165,2	3,2	0,2885
YOR060C	2,3	3,2	7,1	6,0	3,1	0,2070
YOR062C	2,8	3,5	2,4	4,7	0,8	0,8833
YOR072W	7,4	10,1	22,0	28,3	3,0	0,3679
YOR072W-A	0,0	0,0	0,0	0,0	#DIV/0!	#DIV/0!
YOR072W-B	3,3	5,2	0,0	0,0	0,0	0,2486
YOR093C	39,8	8,3	30,2	20,4	0,8	0,4152
YOR097C	134,4	173,1	44,3	54,3	0,3	0,3590
YOR1	65,0	48,1	30,7	23,7	0,5	0,2489
YOR105W	15,7	8,7	17,6	18,4	1,1	0,8618
YOR111W	24,3	15,2	21,6	18,2	0,9	0,8282
YOR114W	48,6	17,8	7,9	9,3	0,2	0,0067
YOR131C	112,6	77,9	88,5	69,8	0,8	0,6616
YOR161C-C	28,4	19,7	11,2	22,4	0,4	0,2939
YOR186W	5,6	9,7	4,9	6,8	0,9	0,9068
YOR192C-A	3,0	3,6	3,2	3,8	1,1	0,9260
YOR203W	361,1	219,5	817,4	535,8	2,3	0,1661
YOR214C	3,7	4,7	0,0	0,0	0,0	0,1695
YOR223W	17,4	17,8	10,5	8,8	0,6	0,5155
YOR228C	3,5	4,1	4,3	5,1	1,3	0,7917
YOR238W	94,1	63,2	150,7	157,4	1,6	0,5291
YOR246C	39,5	36,4	28,3	20,3	0,7	0,6089
YOR262W	81,6	25,5	75,0	56,1	0,9	0,8378
YOR268C	3,5	7,1	81,9	99,5	23,2	0,1674
YOR283W	182,8	30,3	240,7	62,9	1,3	0,1482
YOR289W	39,4	11,1	93,6	67,3	2,4	0,1631
YOR292C	8,1	2,7	8,0	6,6	1,0	0,9913
YOR293C-A	44,3	57,0	6,9	13,9	0,2	0,2502
YOR296W	55,9	26,1	70,0	47,1	1,3	0,6169

YOR300W	8,0	1,0	5,5	6,5	0,7	0,4779
YOR302W	98,1	72,0	73,3	52,8	0,7	0,5993
YOR304C-A	120,3	30,7	121,4	54,3	1,0	0,9725
YOR314W-A	1,1	2,3	1,9	2,4	1,7	0,6506
YOR316C-A	5,2	4,3	4,9	9,9	0,9	0,9575
YOR318C	40,5	10,5	17,0	11,4	0,4	0,0226
YOR338W	11,0	11,3	14,0	10,0	1,3	0,7085
YOR342C	113,0	52,8	48,1	38,2	0,4	0,0933
YOR343C	4,5	3,8	14,9	10,9	3,4	0,1201
YOR343W-A	368,3	295,4	190,8	129,7	0,5	0,3133
YOR343W-B	0,0	0,0	0,0	0,0	#DIV/0!	#DIV/0!
YOR345C	5,6	6,7	4,9	9,9	0,9	0,9199
YOR352W	9,1	6,1	3,5	4,4	0,4	0,1830
YOR356W	24,7	13,8	24,7	19,4	1,0	0,9986
YOR365C	68,4	72,2	265,5	368,8	3,9	0,3346
YOR366W	2,5	1,8	1,2	2,4	0,5	0,4127
YOR376W	13,4	15,1	1,2	2,4	0,1	0,1625
YOR378W	1,6	1,9	0,0	0,0	0,0	0,1517
YOR381W-A	3,2	2,2	62,8	123,8	19,5	0,3732
YOR385W	15,6	7,9	134,4	132,0	8,6	0,1226
YOR387C	4,0	2,8	1,0	2,0	0,2	0,1337
YOR389W	27,7	9,7	40,1	29,6	1,4	0,4561
YOR390W	24,3	10,3	13,8	13,3	0,6	0,2567
YOR392W	0,0	0,0	0,0	0,0	#DIV/0!	#DIV/0!
YOR394C-A	5,2	3,9	0,0	0,0	0,0	0,0366
YOS9	8,2	4,0	9,9	7,8	1,2	0,7096
YOX1	132,3	63,7	70,5	79,0	0,5	0,2697
YPC1	17,7	11,4	11,2	8,3	0,6	0,3922
YPI1	15,8	16,8	44,6	53,8	2,8	0,3467
YPK1	71,8	32,2	50,2	35,5	0,7	0,4010
YPK2	9,6	9,3	13,5	9,7	1,4	0,5815
YPK9	11,1	9,3	22,8	22,4	2,1	0,3709
YPL014W	51,1	16,9	28,8	20,0	0,6	0,1387
YPL025C	0,0	0,0	2,8	3,4	#DIV/0!	0,1592
YPL038W-A	141,4	44,4	162,8	57,2	1,2	0,5760
YPL039W	7,3	8,4	4,5	5,4	0,6	0,6022
YPL041C	4,0	2,8	1,9	2,4	0,5	0,3122
YPL060C-A	4,0	2,0	1,3	2,6	0,3	0,1485
YPL062W	3,1	2,7	4,6	9,2	1,5	0,7659
YPL066W	35,1	12,9	23,4	16,2	0,7	0,3015
YPL067C	120,7	128,8	50,2	79,5	0,4	0,3879
YPL068C	73,3	20,8	71,5	51,4	1,0	0,9477
YPL071C	16,9	4,4	11,1	12,2	0,7	0,4061
YPL077C	3,5	7,0	1,3	2,6	0,4	0,5806
YPL088W	15,2	7,7	47,3	34,3	3,1	0,1175
YPL107W	233,8	145,0	125,0	112,1	0,5	0,2803
YPL109C	8,1	5,3	22,8	18,5	2,8	0,1766
YPL113C	87,0	58,5	52,4	57,0	0,6	0,4293
YPL119C-A	3,8	2,9	1,2	2,4	0,3	0,2087
YPL150W	3,1	2,6	13,9	11,6	4,5	0,1192

YPL152W-A	0,0	0,0	1,0	2,0	#DIV/0!	0,3559
YPL162C	28,2	13,3	10,2	8,5	0,4	0,0626
YPL168W	31,3	19,1	27,5	22,0	0,9	0,8045
YPL182C	1,1	2,1	4,1	3,1	3,8	0,1640
YPL191C	19,7	7,9	118,8	160,3	6,0	0,2629
YPL199C	27,3	13,6	14,1	14,9	0,5	0,2403
YPL205C	1,5	3,0	0,0	0,0	0,0	0,3559
YPL216W	109,6	47,1	202,5	134,2	1,8	0,2390
YPL225W	589,5	196,8	488,7	334,5	0,8	0,6221
YPL229W	59,7	31,8	149,2	98,3	2,5	0,1339
YPL236C	16,7	9,0	17,8	13,3	1,1	0,8948
YPL245W	14,4	9,8	2,2	2,5	0,2	0,0529
YPL247C	5,4	1,3	16,3	15,3	3,0	0,2051
YPL251W	91,8	48,2	103,7	80,5	1,1	0,8086
YPL257W	49,9	12,2	30,4	33,7	0,6	0,3183
YPL260W	55,6	15,6	83,1	58,0	1,5	0,3953
YPL264C	22,1	11,1	34,5	24,3	1,6	0,3873
YPL272C	17,0	12,0	13,0	18,0	0,8	0,7244
YPL276W	209,9	21,0	138,7	93,8	0,7	0,1890
YPL277C	0,0	0,0	0,0	0,0	#DIV/0!	#DIV/0!
YPL278C	5,3	4,9	0,0	0,0	0,0	0,0722
YPP1	36,2	9,9	20,6	14,4	0,6	0,1234
YPR003C	4,6	3,6	117,8	206,8	25,6	0,3157
YPR010C-A	256,1	101,2	361,7	259,8	1,4	0,4774
YPR011C	14,2	11,1	15,2	11,7	1,1	0,9049
YPR012W	51,2	26,9	42,2	29,5	0,8	0,6697
YPR013C	4,0	3,0	7,1	5,8	1,8	0,3738
YPR015C	12,6	3,8	25,1	20,3	2,0	0,2730
YPR022C	35,3	14,4	56,5	43,3	1,6	0,3897
YPR027C	102,9	30,6	156,3	106,3	1,5	0,3713
YPR036W-A	0,0	0,0	0,0	0,0	#DIV/0!	#DIV/0!
YPR059C	39,7	37,6	11,3	7,8	0,3	0,1894
YPR063C	46,9	46,3	45,8	45,9	1,0	0,9746
YPR064W	13,1	9,1	54,8	56,2	4,2	0,1937
YPR071W	17,0	17,4	21,3	19,0	1,3	0,7502
YPR074W-A	0,0	0,0	2,4	4,7	#DIV/0!	0,3559
YPR077C	0,0	0,0	33,0	60,9	#DIV/0!	0,3211
YPR078C	15,3	9,5	114,2	154,4	7,5	0,2480
YPR084W	5,1	4,6	5,1	10,1	1,0	0,9881
YPR089W	53,4	24,3	54,1	38,2	1,0	0,9757
YPR091C	29,6	21,2	133,2	137,4	4,5	0,1868
YPR097W	6,6	9,6	6,3	7,2	1,0	0,9605
YPR1	123,6	104,0	78,9	58,0	0,6	0,4816
YPR108W-A	13,6	10,5	0,0	0,0	0,0	0,0412
YPR109W	31,5	17,8	55,8	41,1	1,8	0,3185
YPR114W	171,9	68,3	273,9	254,3	1,6	0,4682
YPR117W	38,0	19,9	68,2	48,9	1,8	0,2961
YPR126C	10,3	5,9	10,8	8,7	1,0	0,9301
YPR127W	32,2	20,3	163,1	231,9	5,1	0,3035
YPR130C	40,0	25,1	29,6	25,9	0,7	0,5867

YPR145C-A	6,4	5,8	3,1	2,3	0,5	0,3300
YPR146C	106,1	42,9	60,8	44,4	0,6	0,1921
YPR147C	5,9	6,9	4,2	5,6	0,7	0,7267
YPR148C	58,5	23,5	110,3	101,7	1,9	0,3597
YPR153W	5,3	4,4	10,7	9,8	2,0	0,3595
YPR157W	0,0	0,0	6,2	6,7	#DIV/0!	0,1101
YPR158C-C	95,1	39,6	83,2	66,7	0,9	0,7705
YPR159C-A	1,9	2,3	5,3	4,0	2,7	0,1966
YPR160W-A	0,0	0,0	1,3	2,5	#DIV/0!	0,3559
YPR170C	0,6	1,2	2,3	4,6	3,8	0,5035
YPR172W	13,0	9,7	5,6	4,1	0,4	0,2062
YPR174C	13,4	9,9	10,9	7,9	0,8	0,7067
YPR196W	9,5	8,1	11,9	9,4	1,3	0,7150
YPR202W	332,1	385,4	144,0	215,6	0,4	0,4269
YPR204W	3,7	4,3	0,0	0,0	0,0	0,1358
YPS1	33,4	13,9	19,0	19,7	0,6	0,2793
YPS3	313,4	252,7	216,5	281,7	0,7	0,6266
YPS6	2,7	3,3	9,7	11,7	3,6	0,2933
YPS7	93,7	17,8	63,0	43,4	0,7	0,2379
YPT1	134,2	84,3	275,0	236,1	2,0	0,3041
YPT10	17,6	16,6	7,9	9,5	0,4	0,3494
YPT11	19,1	8,4	6,1	6,1	0,3	0,0457
YPT32	116,6	19,3	43,6	29,2	0,4	0,0059
YPT35	1,0	2,0	2,4	4,7	2,4	0,6108
YPT52	7119,8	3835,5	1365,2	1065,7	0,2	0,0277
YPT53	8,0	7,0	10,2	8,9	1,3	0,7110
YPT7	159,7	124,3	92,0	79,1	0,6	0,3936
YRA1	49,6	22,3	63,1	48,4	1,3	0,6294
YRA2	44,2	31,3	45,5	33,1	1,0	0,9560
YRB1	56,9	46,9	186,6	143,3	3,3	0,1361
YRB2	88,1	52,6	160,1	108,9	1,8	0,2785
YRB30	30,6	31,7	56,0	43,5	1,8	0,3819
YRF1-1	6,1	9,2	1,3	2,6	0,2	0,3626
YRM1	24,2	13,6	34,2	36,1	1,4	0,6252
YRO2	191,5	164,7	6135,4	6143,1	32,0	0,1012
YRR1	38,4	23,4	71,5	72,7	1,9	0,4188
YSA1	71,0	44,2	93,4	81,6	1,3	0,6471
YSC83	42,7	29,6	17,7	19,6	0,4	0,2092
YSC84	40,6	29,9	100,0	44,6	2,5	0,0688
YSF3	17,1	10,9	10,9	12,9	0,6	0,4912
YSH1	21,2	18,4	10,5	12,6	0,5	0,3733
YSP1	123,4	57,5	91,9	64,6	0,7	0,4939
YSP2	51,0	15,5	30,9	21,4	0,6	0,1788
YSP3	60,2	44,9	157,6	199,0	2,6	0,3763
YSR3	456,8	215,5	206,7	73,9	0,5	0,0706
YSW1	204,2	199,8	58,0	57,0	0,3	0,2089
YSY6	15,0	12,9	14,5	12,4	1,0	0,9512
YTA12	299,7	99,3	536,0	266,1	1,8	0,1472
YTA6	11,5	7,6	14,0	16,8	1,2	0,8001
YTA7	171,4	73,0	301,2	252,3	1,8	0,3610

YTH1	12,0	7,5	6,9	5,4	0,6	0,3138
YTM1	34,2	13,6	97,8	119,6	2,9	0,3317
YTP1	148,2	83,1	141,7	132,0	1,0	0,9363
YUR1	10,5	7,0	11,8	11,6	1,1	0,8511
YVC1	91,2	29,3	49,9	33,9	0,5	0,1147
YVH1	38,3	4,0	10,1	7,3	0,3	0,0005
ZAP1	39,3	15,8	54,8	46,3	1,4	0,5506
ZDS1	48,1	24,5	32,6	28,1	0,7	0,4395
ZDS2	63,4	19,0	22,6	18,6	0,4	0,0220
ZIM17	45,7	36,3	34,7	38,4	0,8	0,6909
ZIP1	25,5	9,2	33,2	23,7	1,3	0,5697
ZIP2	136,8	95,8	282,8	155,0	2,1	0,1602
ZPR1	24,9	20,0	117,6	92,2	4,7	0,0972
ZPS1	20,8	10,7	30,4	21,6	1,5	0,4592
ZRC1	68,5	60,1	80,2	66,5	1,2	0,8032
ZRG17	7,8	7,4	7,7	7,0	1,0	0,9869
ZRG8	186,3	90,5	85,6	61,5	0,5	0,1150
ZRT1	50,3	31,2	59,7	53,0	1,2	0,7679
ZRT2	73,8	36,9	63,8	64,5	0,9	0,7969
ZRT3	140,6	52,7	102,0	70,5	0,7	0,4148
ZTA1	4,0	3,8	17,1	17,4	4,3	0,1910
ZUO1	217,1	173,0	355,4	344,7	1,6	0,5001
ZWF1	35,0	13,9	36,0	30,3	1,0	0,9524
