# Supplementary material for: Transcriptional response of Saccharomyces cerevisiae to potassium starvation
Source: BMC Genomics. 2014 Nov 29;15(1):1040. doi: 10.1186/1471-2164-15-1040 (PMC4289377; doi:10.1186/1471-2164-15-1040)
Supplement: Supplementary file 3 — Additional file 3: Genes of which antisense transcripts are up‒ or down‒regulated more than 2‒fold upon potassium starvation (P<0.01). (PDF 82 KB) [file 12864_2014_6863_MOESM3_ESM.pdf]

Additional file 3. Genes of which antisense transcripts are up- or down-regulated more than 2-fold upon potassium starvation (P<0.01).

Up-regulated genes.

Annotation	Average 50 mM (reads per million)	SD 50 mM	Average 0 mM (reads per million)	SD 0 mM	Fold change 0 mM/50 mM	P-value
YER053C-A	32,8	15,8	655,0	150,0	20,0	0,0002
YLR035C-A	67,0	24,6	672,2	294,7	10,0	0,0064
AGA1	45,9	23,1	441,2	79,5	9,6	0,0001
PRR2	32,8	7,0	311,6	41,3	9,5	0,0000
REF2	11,4	8,4	101,8	40,0	8,9	0,0045
SOL4	25,0	16,0	133,7	34,1	5,4	0,0012
SLO1	6,6	1,8	34,6	7,7	5,3	0,0004
HUA1	65,1	24,0	308,6	21,0	4,7	0,0000
PSP2	46,2	38,7	211,0	55,9	4,6	0,0029
SSE2	112,9	36,0	500,1	65,1	4,4	0,0000
RAT1	77,8	29,6	315,2	31,1	4,1	0,0000
VTC4	53,7	34,7	217,7	80,5	4,1	0,0096
PHO8	56,6	9,5	226,8	83,2	4,0	0,0066
BTN2	107,7	78,4	427,8	148,2	4,0	0,0088
YEL028W	77,3	32,8	301,5	73,2	3,9	0,0014
ODC2	446,5	146,1	1663,9	509,2	3,7	0,0037
AIM17	90,1	17,7	331,9	48,9	3,7	0,0001
YLR225C	42,0	20,4	153,9	37,8	3,7	0,0020
ACH1	106,7	33,8	389,1	90,2	3,6	0,0011
FIG4	37,0	9,0	134,9	34,7	3,6	0,0016
YHR007C-A	429,6	58,1	1517,9	151,9	3,5	0,0000
YJL127C-B	109,8	24,7	362,1	124,1	3,3	0,0072
ESS1	74,4	16,2	243,3	34,0	3,3	0,0001
YMR122W-A	1383,3	806,4	4346,4	792,4	3,1	0,0019
SPI1	99,8	60,4	308,9	90,3	3,1	0,0085
SNA2	67,3	45,4	205,5	56,1	3,1	0,0087
YKL068W-A	229,7	80,7	675,8	126,8	2,9	0,0010
GPI10	74,1	17,6	196,6	54,7	2,7	0,0053
QDR2	51,0	8,9	131,5	34,2	2,6	0,0039
GLC8	141,8	86,2	357,6	43,9	2,5	0,0043
YCR024C-B	3141,3	705,6	6707,7	478,5	2,1	0,0002
NYV1	109,6	39,2	230,7	20,6	2,1	0,0016
FOL2	382,2	143,7	783,2	107,7	2,0	0,0043
ACP1	192,5	98,3	390,2	40,2	2,0	0,0098

Down-regulated genes

Annotation	Average 50 mM (reads per million)	SD 50 mM	Average 0 mM (reads per million)	SD 0 mM	Fold change 50 mM/0 mM	P-value
HES1	4,9	1,4	0,0	0,0		0,0004
YKR012C	4,6	1,7	0,0	0,0		0,0016
ECI1	5,9	2,4	0,0	0,0		0,0025
YJR128W	11,9	4,8	0,0	0,0		0,0026
CLB6	29,5	11,1	0,7	1,3	44,7	0,0021
SPO74	24,3	11,0	1,0	2,0	24,5	0,0059
CLN2	262,1	127,6	15,9	11,1	16,4	0,0085
ATP12	8,3	2,2	0,7	1,3	12,7	0,0010
SPC29	8,0	3,4	0,7	1,3	12,1	0,0069
COQ10	231,5	37,1	20,8	15,5	11,1	0,0000
ERP3	190,0	72,5	17,3	14,6	11,0	0,0034
NRM1	9,9	4,3	1,0	2,0	10,0	0,0096
HPT1	1541,0	604,6	155,7	125,5	9,9	0,0042
UNG1	22,7	5,6	2,4	2,8	9,3	0,0007
UTP20	615,8	227,3	68,5	61,6	9,0	0,0035
DTD1	47,2	18,0	6,0	5,2	7,9	0,0046
RIA1	14,1	6,0	1,9	2,4	7,3	0,0097
THI13	16,8	2,7	2,3	4,6	7,3	0,0016
YBR124W	57,0	13,3	8,6	8,1	6,6	0,0008
YOR114W	48,6	17,8	7,9	9,3	6,1	0,0067
HSH49	79,7	20,9	13,1	14,5	6,1	0,0019
YNL295W	7,9	1,4	1,3	2,6	6,0	0,0047
PMS1	44,1	13,2	7,7	6,0	5,7	0,0024
SWI4	383,0	119,5	69,4	50,9	5,5	0,0029
MRP2	1169,6	435,1	221,3	158,7	5,3	0,0064
POL2	115,8	37,7	22,0	16,8	5,3	0,0039
CIN1	67,8	18,6	13,0	17,0	5,2	0,0048
YNL162W-A	54,2	20,4	10,5	7,7	5,2	0,0070
CWC22	1678,7	573,2	338,5	53,2	5,0	0,0035
EMW1	11,1	3,3	2,3	2,6	4,9	0,0058
NSE3	32,7	10,2	6,7	5,5	4,9	0,0041
YML122C	248,0	90,5	52,4	48,2	4,7	0,0088
CNL1	22,9	6,6	4,9	5,9	4,7	0,0065
GLT1	82,3	19,4	17,6	14,2	4,7	0,0017
YMR196W	42,6	7,5	9,4	12,0	4,5	0,0034
DPB2	16,6	4,9	3,8	2,5	4,4	0,0035
SIW14	14417,3	3665,0	3328,6	945,3	4,3	0,0011
ELG1	30,7	9,0	7,2	8,3	4,3	0,0086
SIT1	613,0	160,1	143,5	95,8	4,3	0,0024
WSC2	220,4	52,2	51,6	35,2	4,3	0,0017
YLR342W-A	171,6	15,2	40,3	30,9	4,3	0,0003
CSF1	83,5	16,1	20,0	15,1	4,2	0,0012
PSA1	1616,7	130,7	407,5	87,9	4,0	0,0000
YLR049C	47,7	8,5	12,1	10,6	3,9	0,0020
DIA2	104,5	5,3	26,7	21,4	3,9	0,0004

UGO1	918,9	237,8	241,4	113,1	3,8	0,0021
HSL7	55,8	5,5	14,7	15,9	3,8	0,0028
YVH1	38,3	4,0	10,1	7,3	3,8	0,0005
YEL023C	686,5	76,3	182,0	160,2	3,8	0,0013
HLR1	28,0	7,5	7,4	6,0	3,8	0,0051
ARF3	221,1	72,0	59,8	41,3	3,7	0,0081
SEN34	33,9	6,2	9,4	10,9	3,6	0,0078
YMR178W	80,4	8,7	22,6	17,7	3,6	0,0011
TOM7	1097,9	71,2	313,6	121,8	3,5	0,0000
YBR235W	38,6	5,2	11,1	9,9	3,5	0,0026
YKL047W	170,4	45,7	50,2	44,5	3,4	0,0093
IST1	27,3	7,3	8,1	6,8	3,4	0,0084
RCL1	140,2	15,6	41,6	31,8	3,4	0,0014
BRO1	67,9	21,2	20,3	13,9	3,3	0,0095
YBR196C-B	200,4	30,6	63,3	54,6	3,2	0,0046
DMA1	56,0	15,0	17,7	13,6	3,2	0,0093
ADE16	552,7	82,1	179,7	120,6	3,1	0,0022
ULA1	36,8	3,7	12,1	9,7	3,1	0,0030
YGR164W	170,1	44,8	56,6	41,5	3,0	0,0099
VAC14	43,2	4,8	14,8	10,0	2,9	0,0022
MRM2	15,1	1,1	5,4	4,9	2,8	0,0081
YPT32	116,6	19,3	43,6	29,2	2,7	0,0059
ADE4	398,1	70,9	149,0	40,8	2,7	0,0009
RPL20A	2271,7	588,8	862,9	335,3	2,6	0,0060
CTI6	71,9	12,9	27,5	19,9	2,6	0,0096
SNQ2	185,7	24,5	71,1	53,6	2,6	0,0081
YJR149W	87,8	14,4	34,3	23,7	2,6	0,0083
SEC28	663,8	177,0	270,2	41,6	2,5	0,0049
ERG3	1541,6	464,9	629,0	154,4	2,5	0,0098
TEL1	93,6	12,2	38,4	26,2	2,4	0,0087
RTG2	4472,5	1033,5	1922,4	434,2	2,3	0,0039
